# Supplementary material for: Parallel evolution of methyltransferases leads to vobasine biosynthesis in Tabernaemontana elegans and Catharanthus roseus
Source: Front Plant Sci. 2024 Aug 27;15:1451298. doi: 10.3389/fpls.2024.1451298 (PMC11383786; doi:10.3389/fpls.2024.1451298)
Supplement: Supplementary file 1 [file DataSheet1.pdf]

Parallel evolution of methyltransferases leads to vobasine biosynthesis in *Tabernaemontana elegans* and *Catharanthus roseus*

Maisha Farzana, Matthew Bailey Richardson, Daniel André Ramey Deschênes, Zhan Mai, Destiny Ichechi Njoku, Ghislain Deslongchamps, and Yang Qu

Supplementary information

Table of Contents

Supplementary Figure 1. UV absorption and MS/MS profiles for MIAs 3
Supplementary Figure 2. ^{1}H NMR spectra for vobasine in $CDCl_3$. 4
Supplementary Figure 3. ^{13}C NMR spectra for vobasine in $CDCl_3$. 5
Supplementary Figure 4. HSQC NMR spectra for vobasine in $CDCl_3$.................................6
Supplementary Figure 5. HMBC NMR spectra for vobasine in $CDCl_3$................................7
Supplementary Figure 6. COSY NMR spectra for vobasine in $CDCl_3$.................................8
Supplementary Figure 7. NOESY NMR spectra for vobasine in $CDCl_3$. 9
Supplementary Figure 8. ^{1}H NMR spectra for apparicine in $CDCl_3$...................................10
Supplementary Figure 9. ^{13}C NMR spectra for apparicine in $CDCl_3$..................................11
Supplementary Figure 10. HSQC NMR spectra for apparicine in $CDCl_3$. 12
Supplementary Figure 11. HMBC NMR spectra for apparicine in $CDCl_3$. 13
Supplementary Figure 12. COSY NMR spectra for apparicine in $CDCl_3$. 14
Supplementary Figure 13. ^{1}H NMR spectra for dregamine in $CDCl_3$.................................15
Supplementary Figure 14. ^{13}C NMR spectra for dregamine in $CDCl_3$. 16
Supplementary Figure 15. HSQC NMR spectra for dregamine in $CDCl_3$. 17
Supplementary Figure 16. HMBC NMR spectra for dregamine in $CDCl_3$. 18
Supplementary Figure 17. NOESY NMR spectra for dregamine in $CDCl_3$. 19
Supplementary Figure 18. COSY NMR spectra for dregamine in $CDCl_3$. 20
Supplementary Figure 19. ^{13}C NMR spectra for tabernaemontanine in $CDCl_3$. 21
Supplementary Figure 20. ^{13}C NMR spectra for tabernaemontanine in $CDCl_3$. 22
Supplementary Figure 21. HSQC NMR spectra for tabernaemontanine in $CDCl_3$..............23
Supplementary Figure 22. HMBC NMR spectra for tabernaemontanine in $CDCl_3$. 24
Supplementary Figure 23. NOESY NMR spectra for tabernaemontanine in $CDCl_3$. 25
Supplementary Figure 24. COSY NMR spectra for tabernaemontanine in $CDCl_3$..............26
Supplementary Figure 25. SDS-PAGE gels of N-terminally 6X-Histagged TePeNMT and CrPeNMT 27
Supplementary Figure 26. 2D ligand interaction maps for *S*-adenosylmethionine (SAM) and perivine at the enzyme active sites of TePeNMT and CrPeNMT. 28
Supplementary Table 1. ^{1}H NMR chemical shifts of vobasine, dregamine, tabernaemontanine, and apparicine in $CDCl_3$..29
Supplementary Table 2. ^{13}C NMR chemical shifts of vobasine, dregamine, tabernaemontanine, and apparicine in $CDCl_3$..30
Supplementary Table 3. MIA substrates tested for TeMT1-3 in this study 31

Supplementary Data 1. Multiple alignment for NMTs used in the phylogenetic analysis of this study. 32
Supplementary Data 2. Cartesian coordinates for CrPeNMT homology model. 35
Supplementary Data 3. Cartesian coordinates for TePeNMT homology model. 131
Supplementary Data 4. Cartesian coordinates for CrPeNMT homology model with bound SAM....227
Supplementary Data 5. Cartesian coordinates for TePeNMT homology model with bound SAM....323
Supplementary Data 6. Cartesian coordinates for perivine docked into CrPeNMT+SAM.419
Supplementary Data 7. Cartesian coordinates for perivine docked into TePeNMT+SAM.515
References 610

Supplementary Figure 1. UV absorption and MS/MS profiles for MIAs

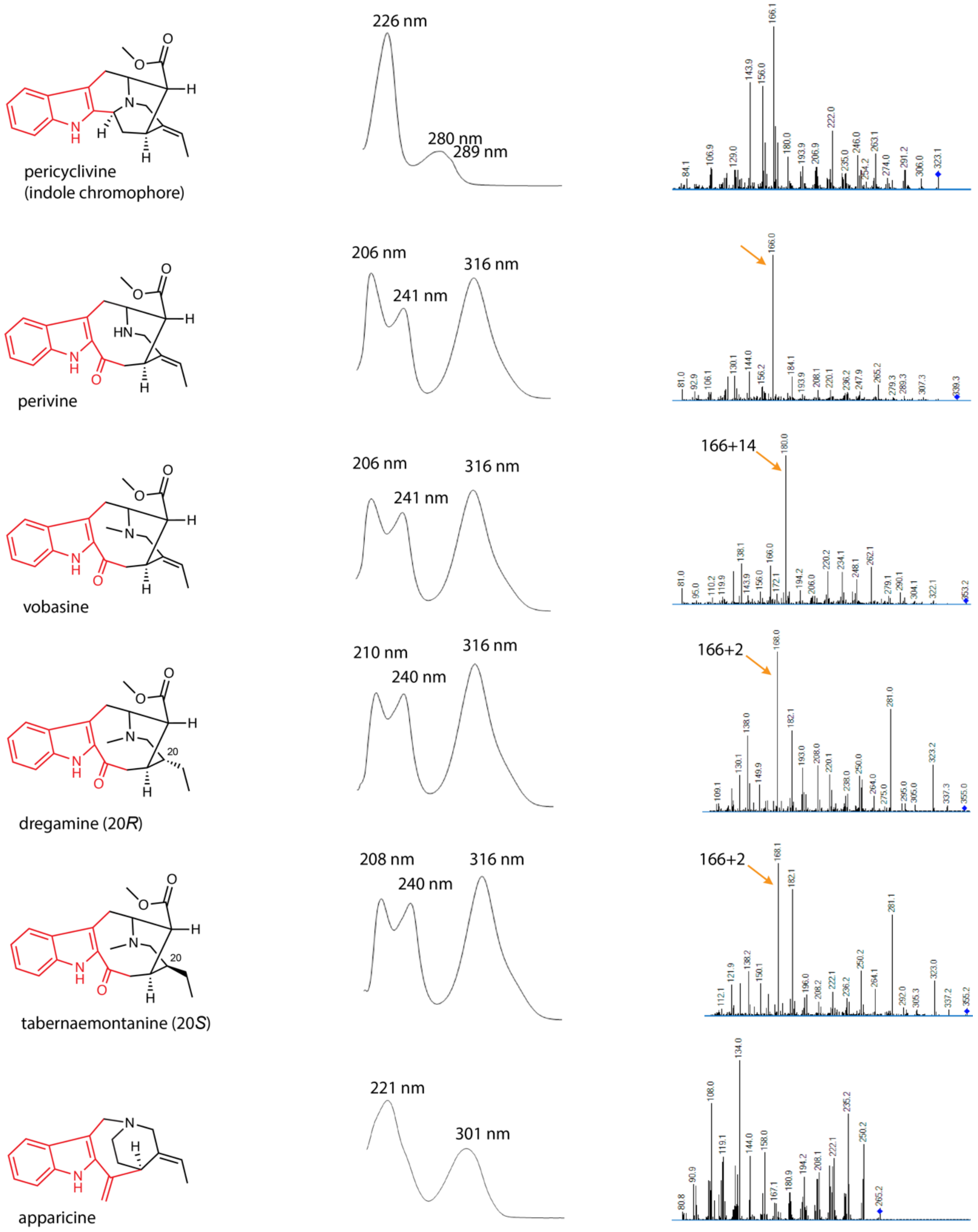

UV absorption and MS/MS profiles for pericyclivine (indole chromophore), perivine, vobasine, dregamine and tabernaemontanine, and apparicine. Chromophores are shown in red. The orange arrows indicate MS daughter ions +14 amu (methylation) from perivine for vobasine, and +2 from vobasine for dregamine and tabernaemontanine.

Supplementary Figure 2. ^{1}H NMR spectra for vobasine in $CDCl_3$.

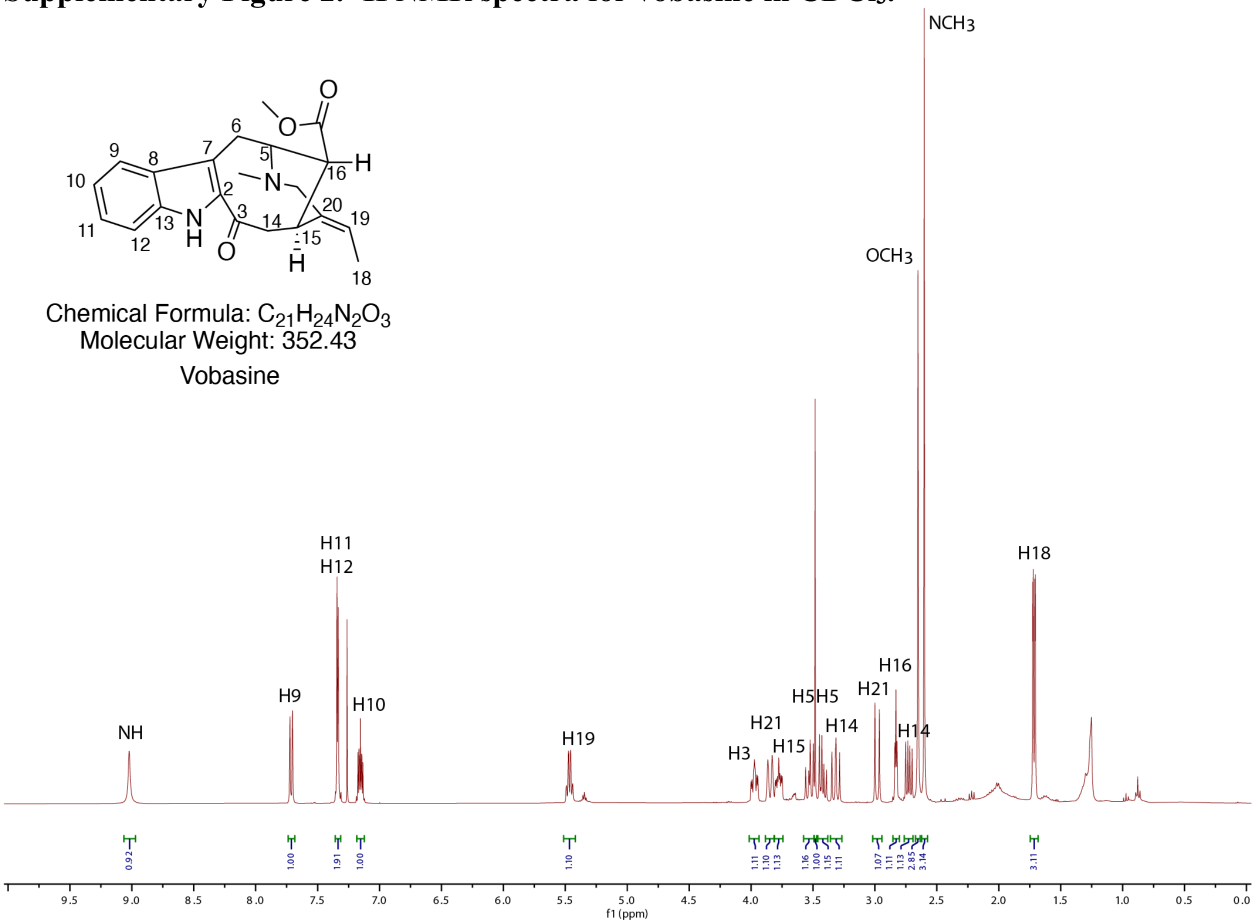

Supplementary Figure 3. ^{13}C NMR spectra for vobasine in $CDCl_3$.

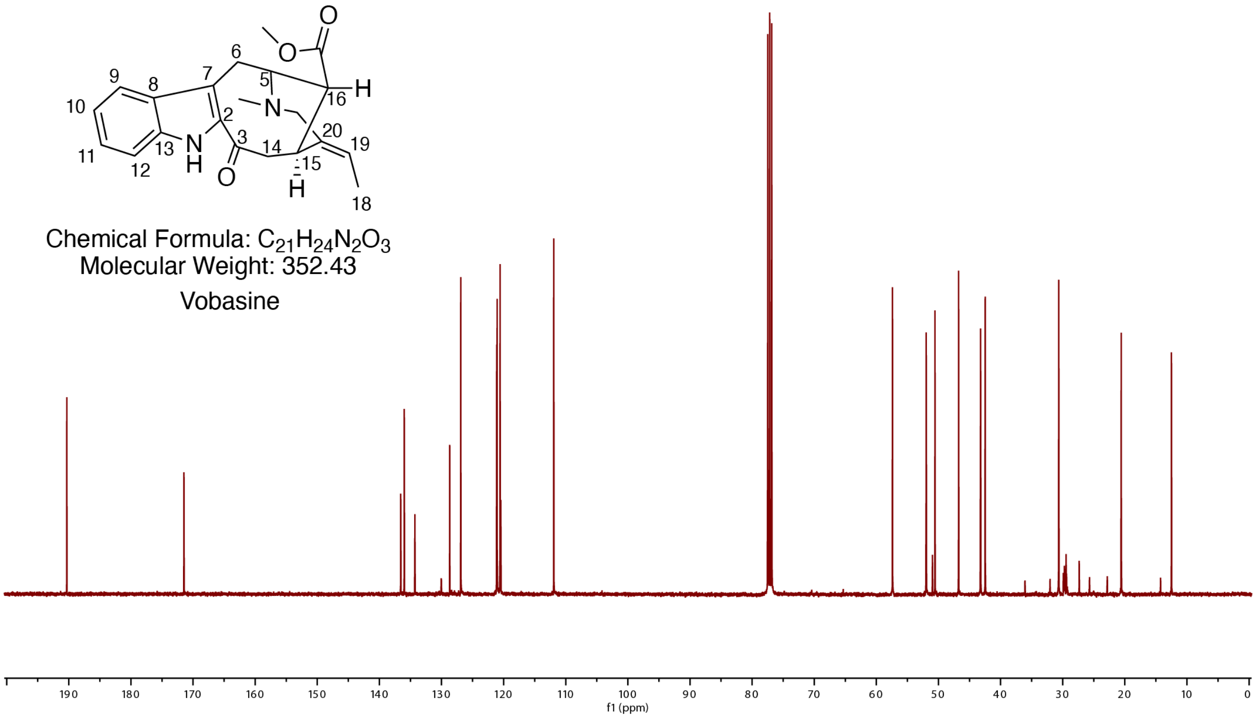

Chemical Formula: $C_{21}H_{24}N_2O_3$
Molecular Weight: 352.43
Vobasine

Supplementary Figure 4. HSQC NMR spectra for vobasine in $CDCl_3$.

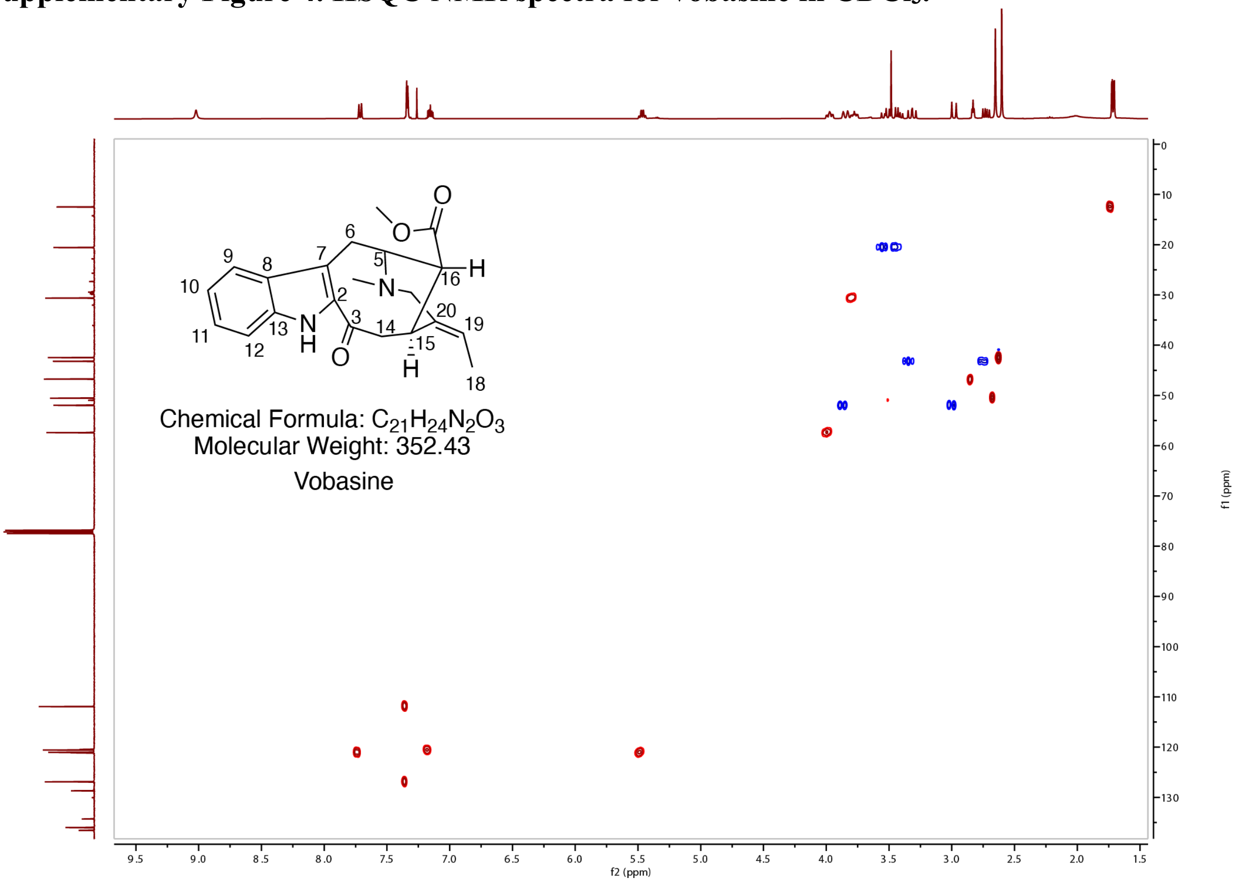

Supplementary Figure 5. HMBC NMR spectra for vobasine in $CDCl_3$.

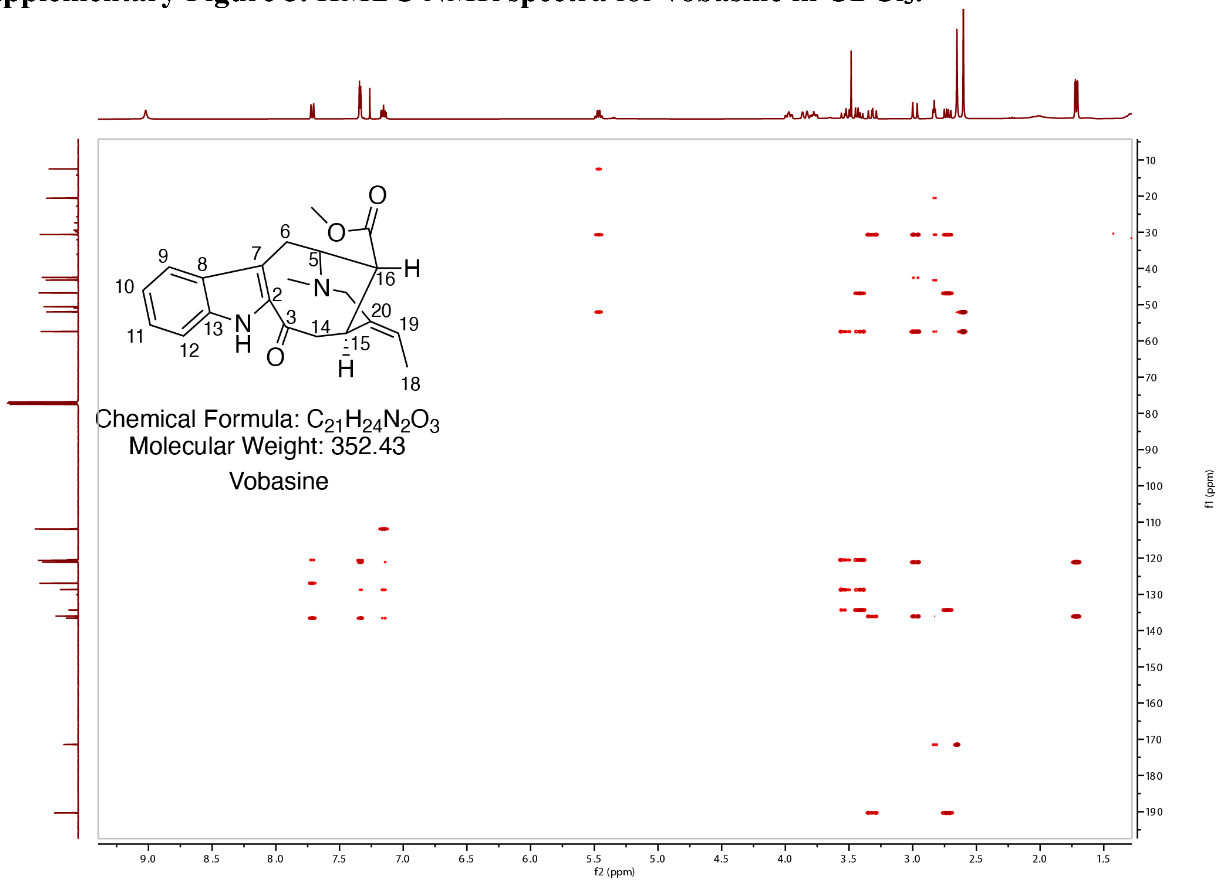

Supplementary Figure 6. COSY NMR spectra for vobasine in $CDCl_3$.

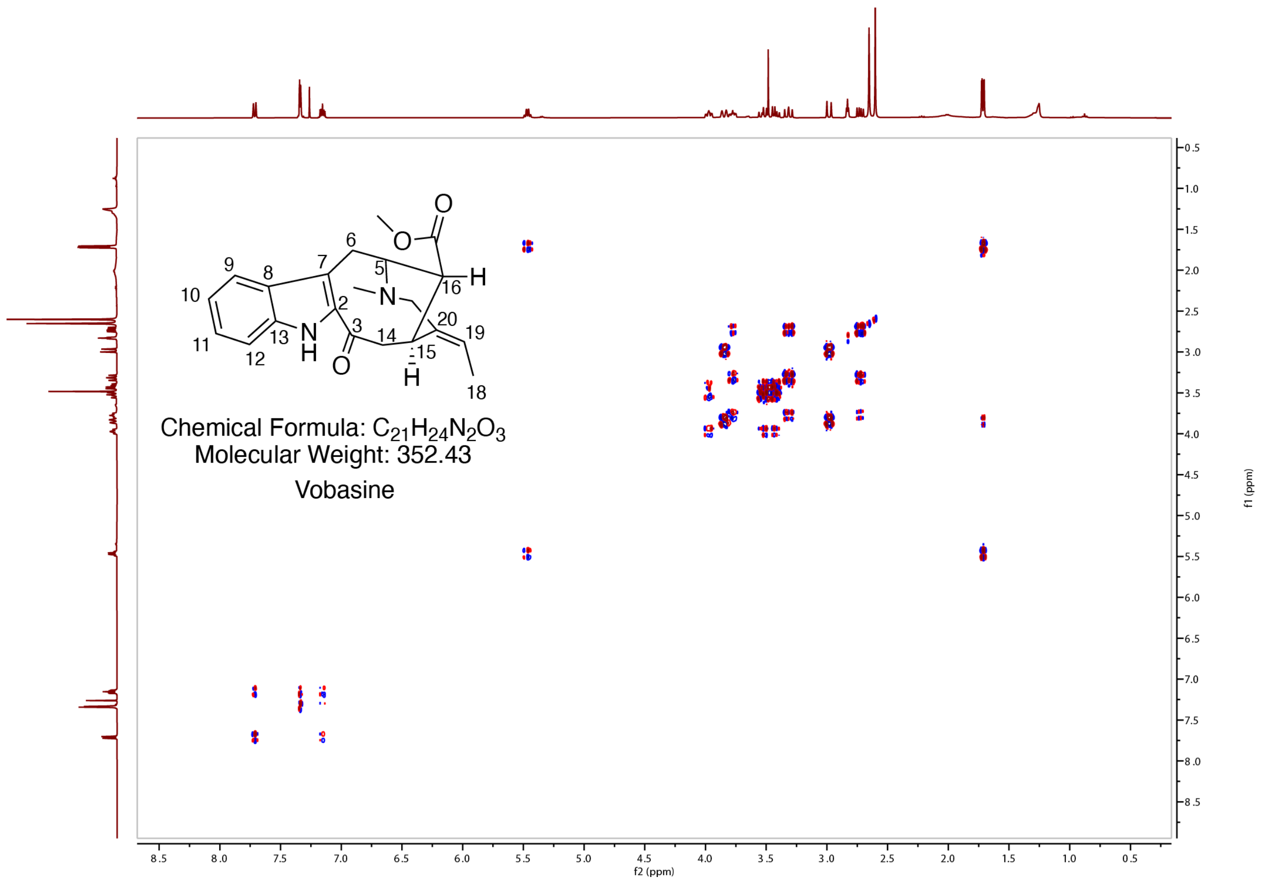

Supplementary Figure 7. NOESY NMR spectra for vobasine in $CDCl_3$.

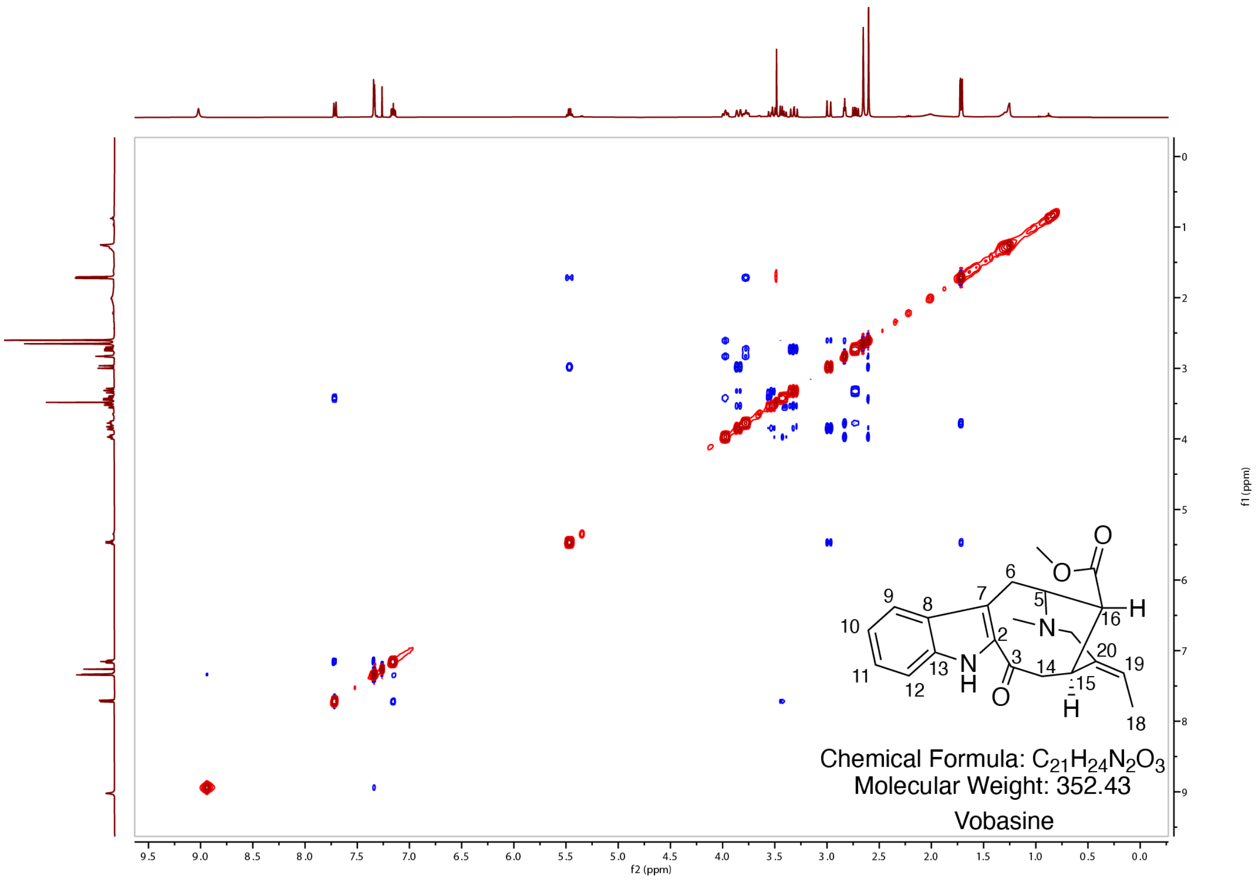

Supplementary Figure 8. ^{1}H NMR spectra for apparicine in $CDCl_3$.

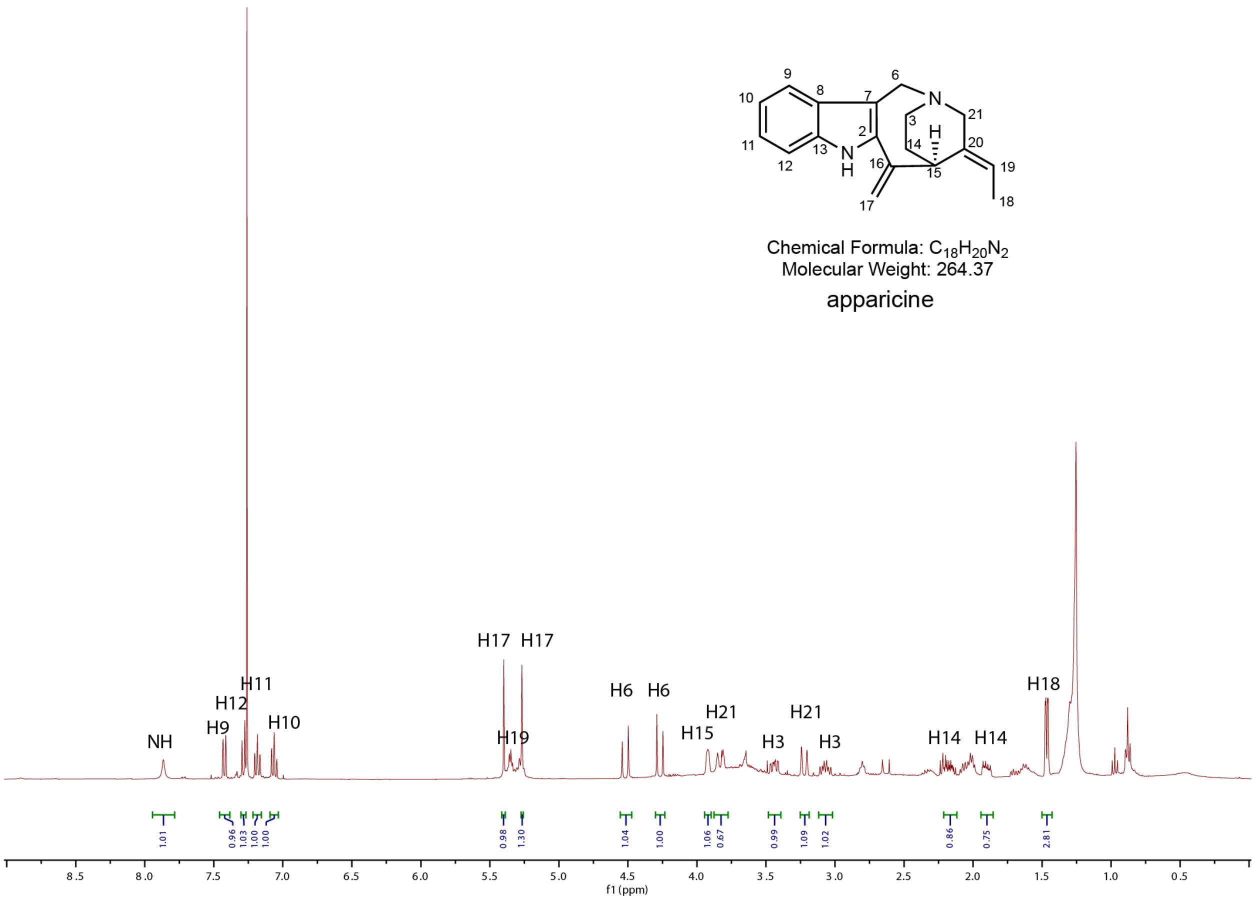

Supplementary Figure 9. ^{13}C NMR spectra for apparicine in $CDCl_3$.

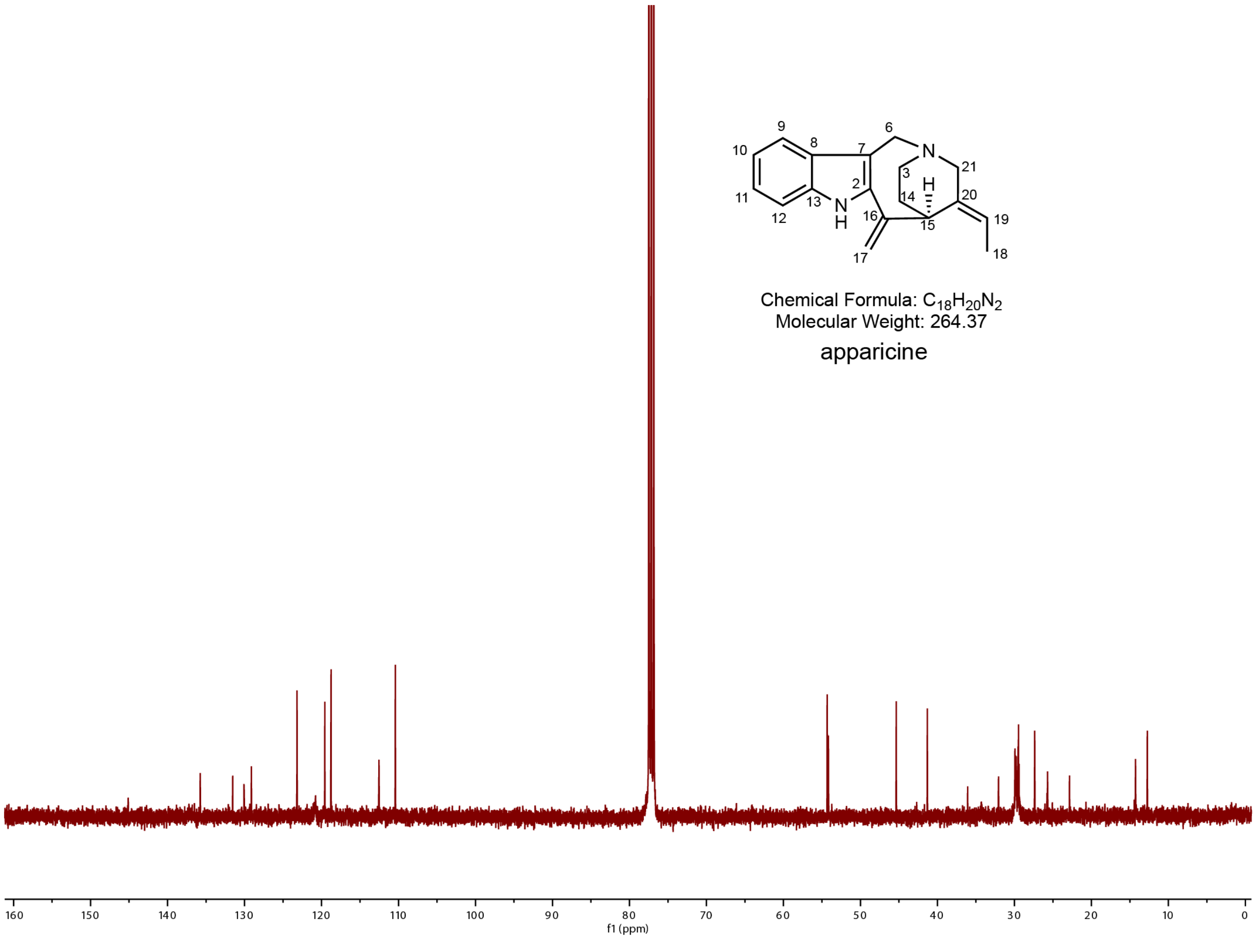

Supplementary Figure 10. HSQC NMR spectra for apparicine in $CDCl_3$.

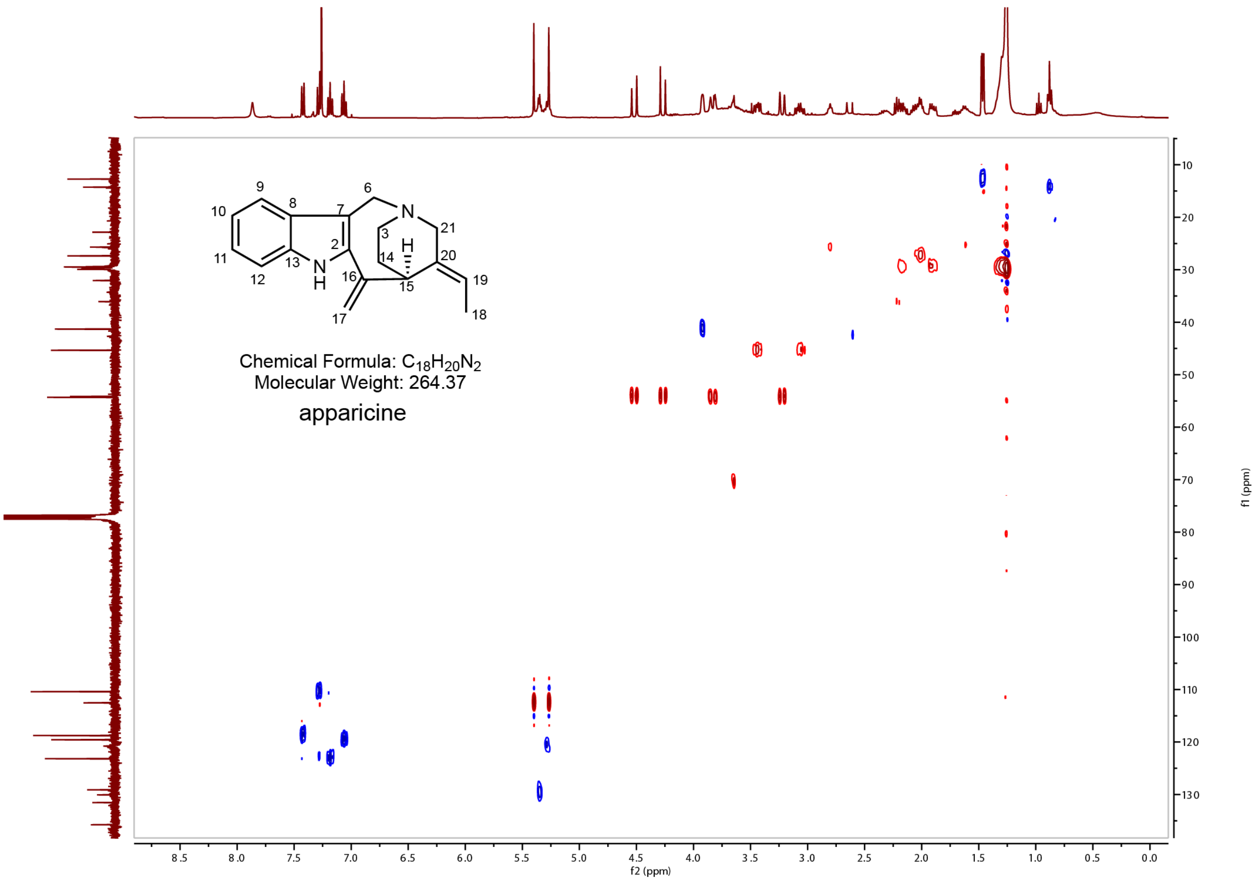

Supplementary Figure 11. HMBC NMR spectra for apparicine in $CDCl_3$.

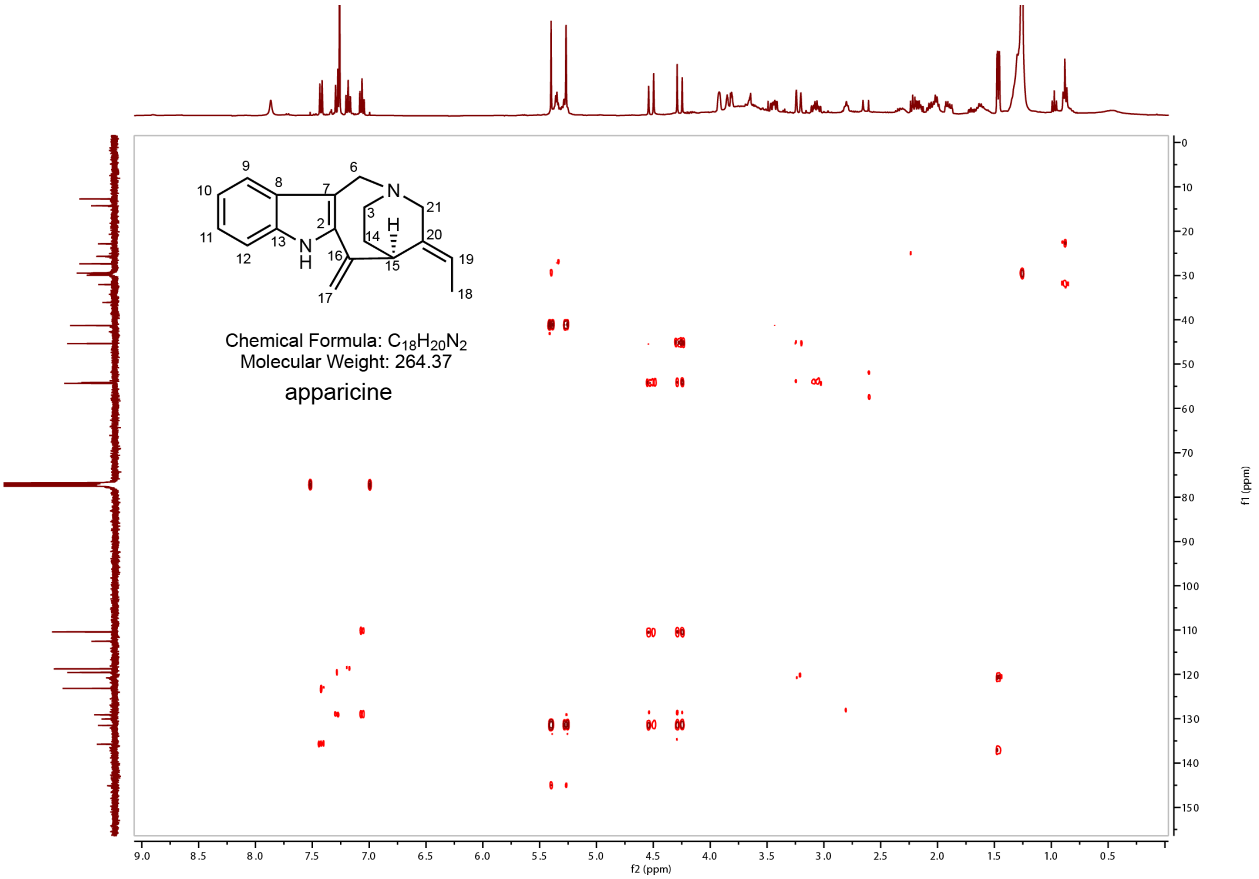

Supplementary Figure 12. COSY NMR spectra for apparicine in $CDCl_3$.

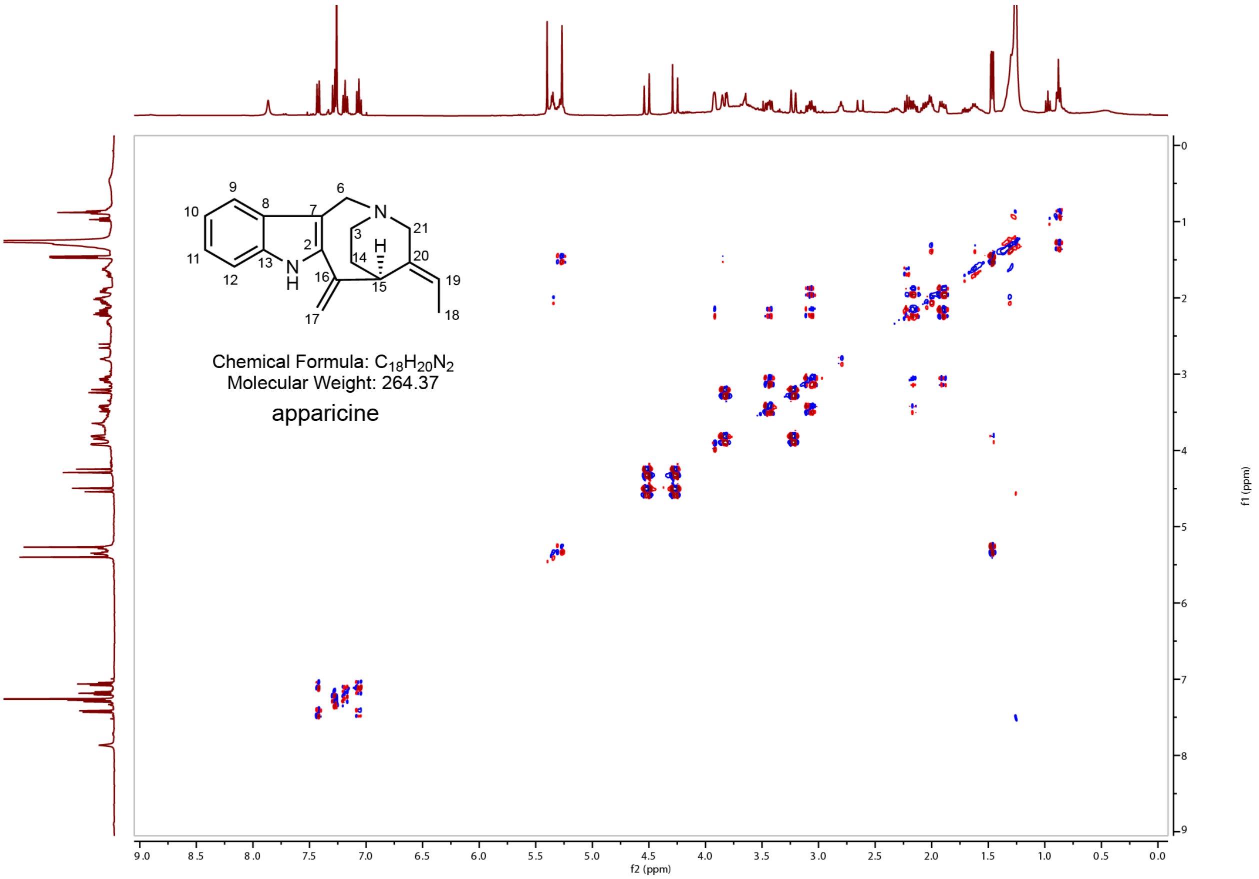

Supplementary Figure 13. ^{1}H NMR spectra for dregamine in $CDCl_3$.

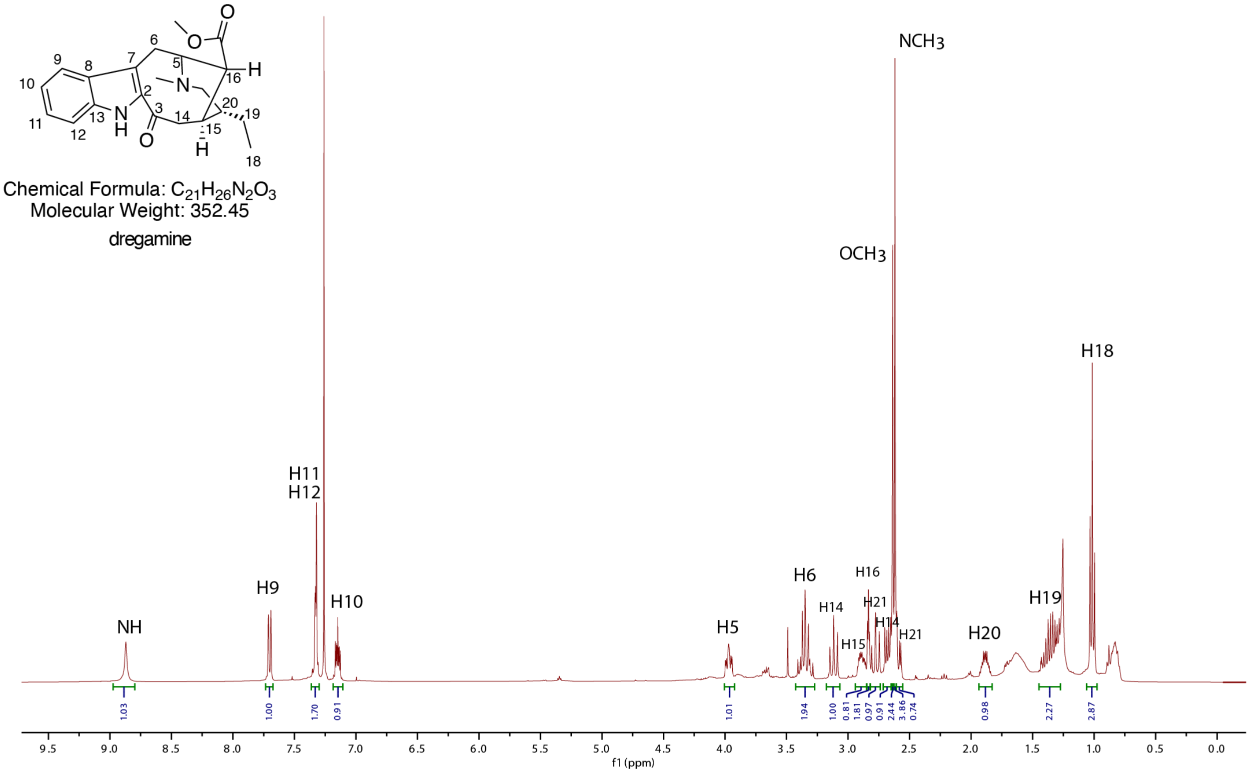

Supplementary Figure 14. ^{13}C NMR spectra for dregamine in $CDCl_3$.

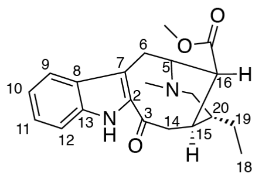

Chemical Formula: $C_{21}H_{26}N_2O_3$
Molecular Weight: 352.45
dregamine

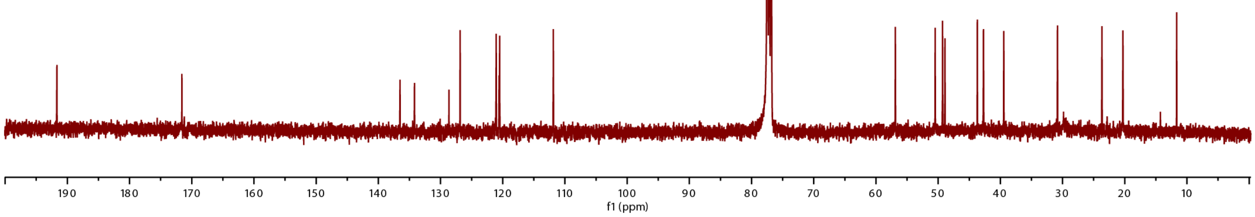

Supplementary Figure 15. HSQC NMR spectra for dregamine in $CDCl_3$.

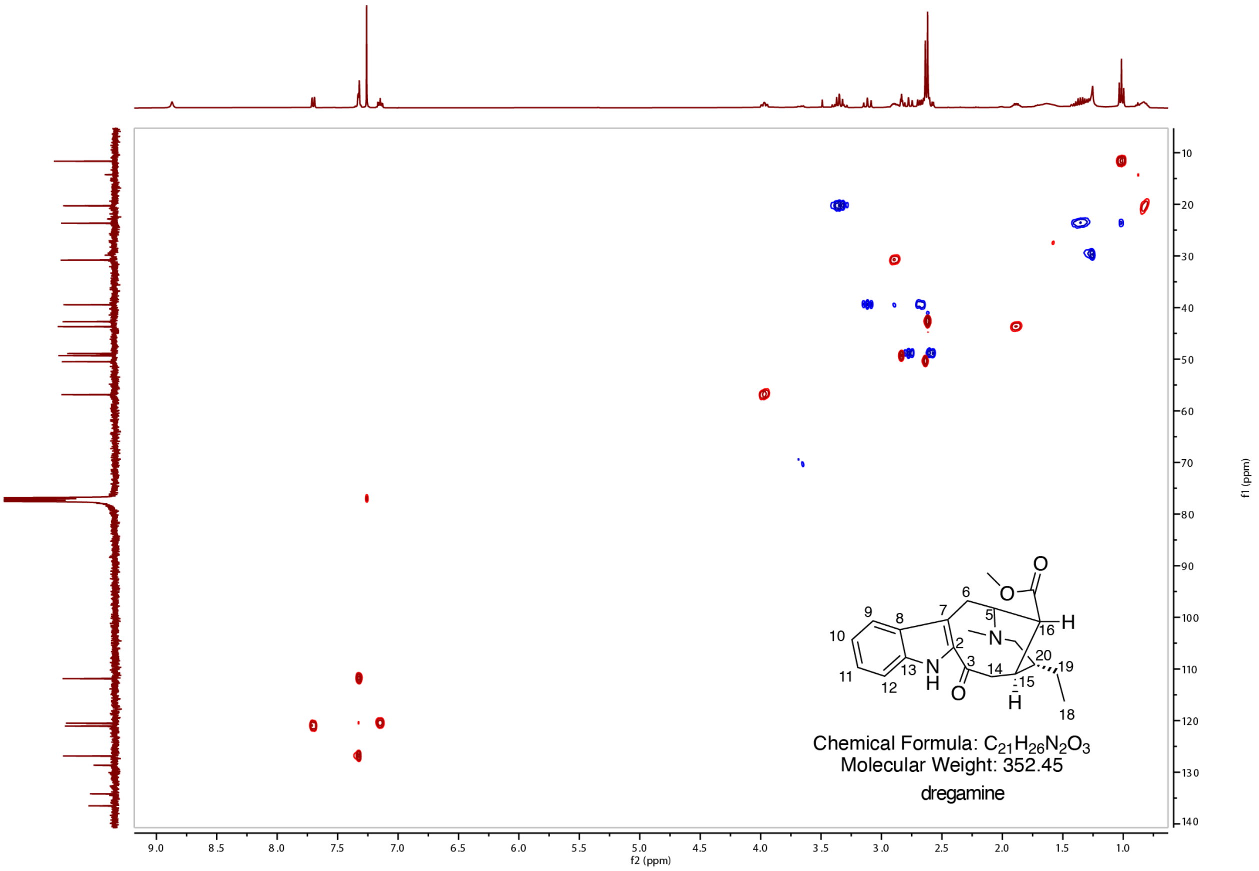

Supplementary Figure 16. HMBC NMR spectra for dregamine in $CDCl_3$.

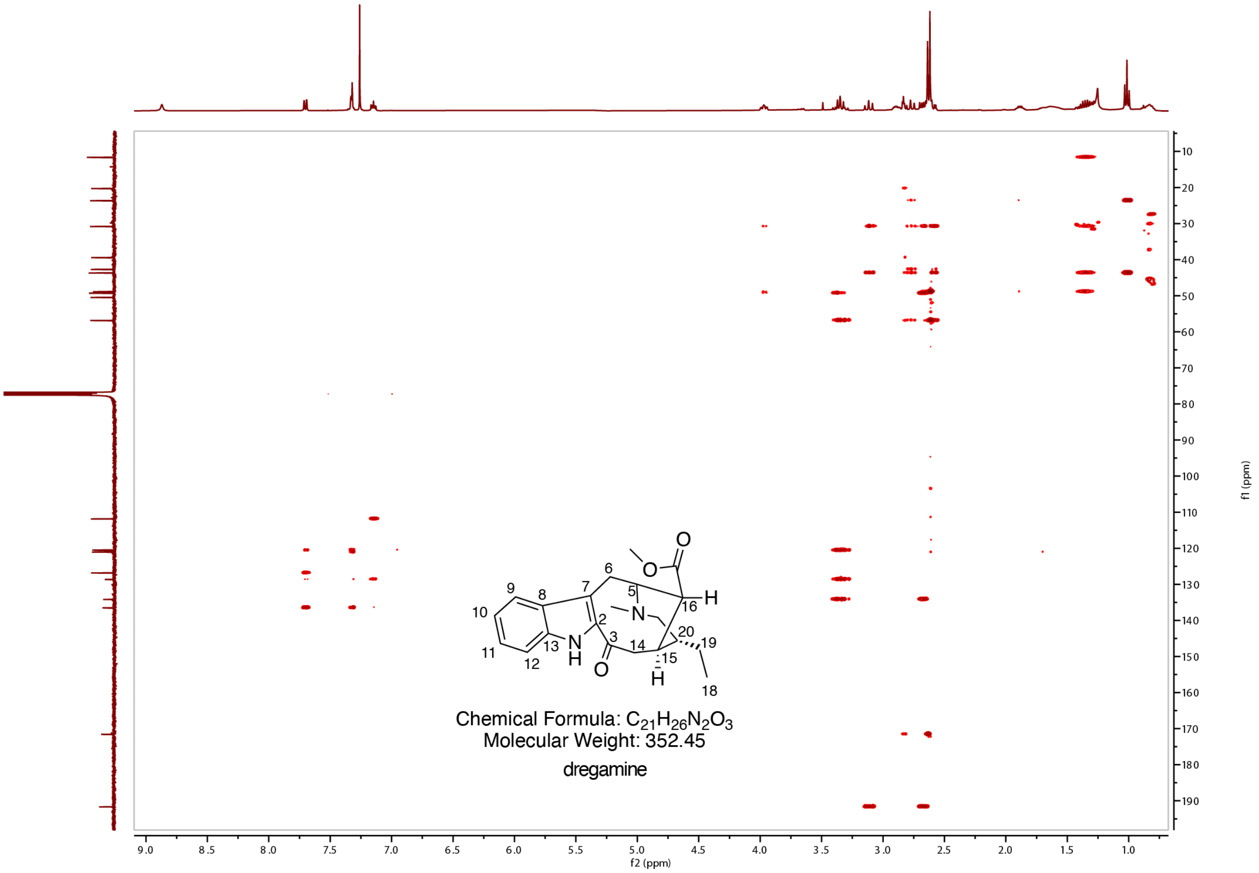

Supplementary Figure 17. NOESY NMR spectra for dregamine in $CDCl_3$.

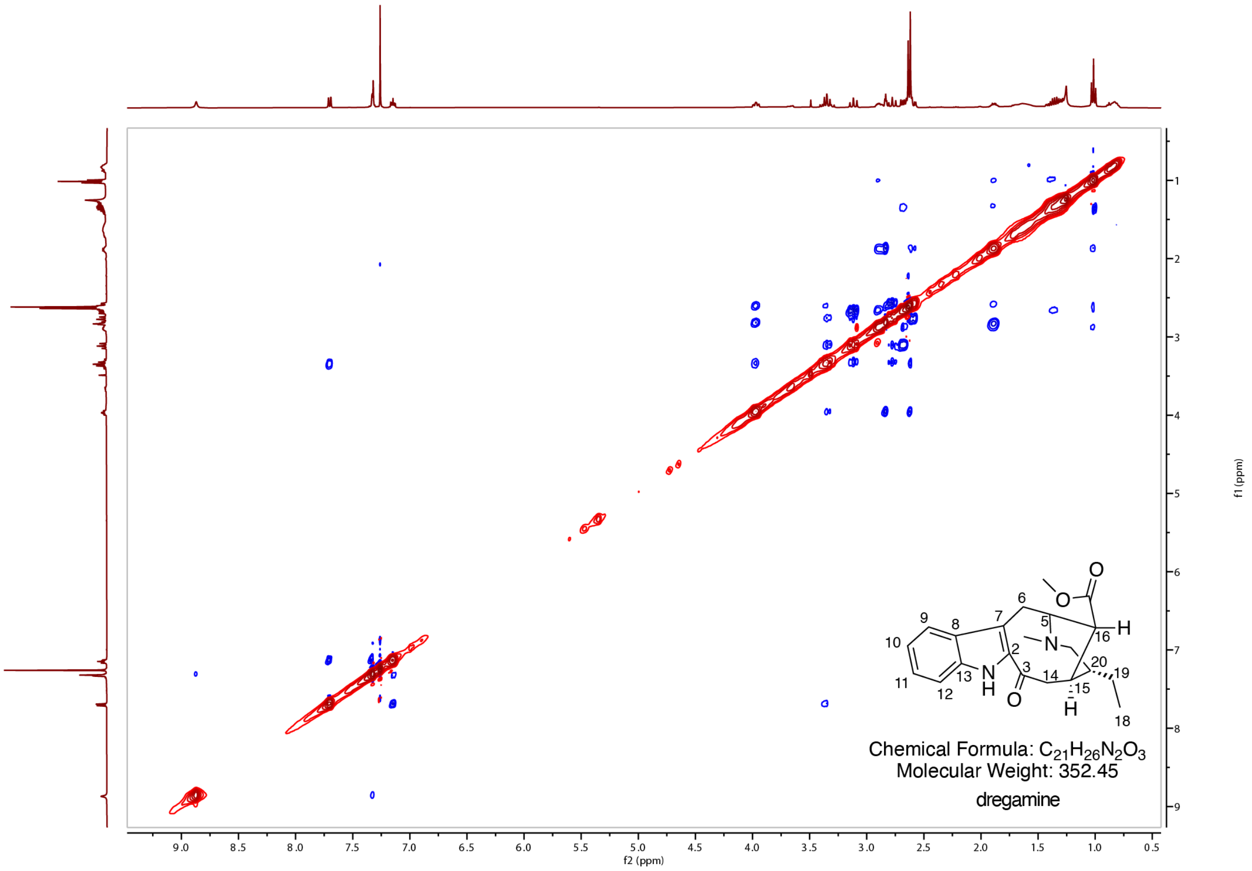

Supplementary Figure 18. COSY NMR spectra for dregamine in $CDCl_3$.

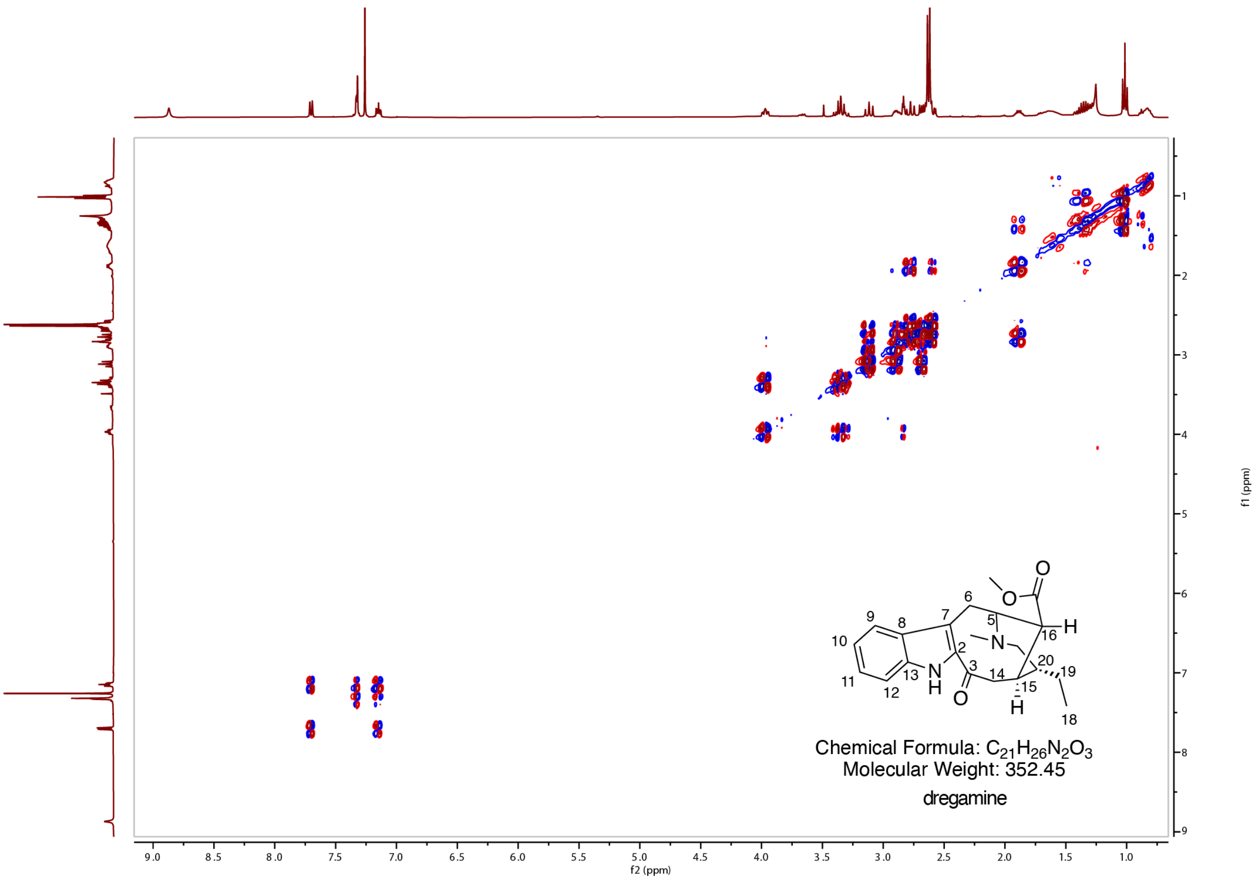

Supplementary Figure 19. ^{13}C NMR spectra for tabernaemontanine in $CDCl_3$.

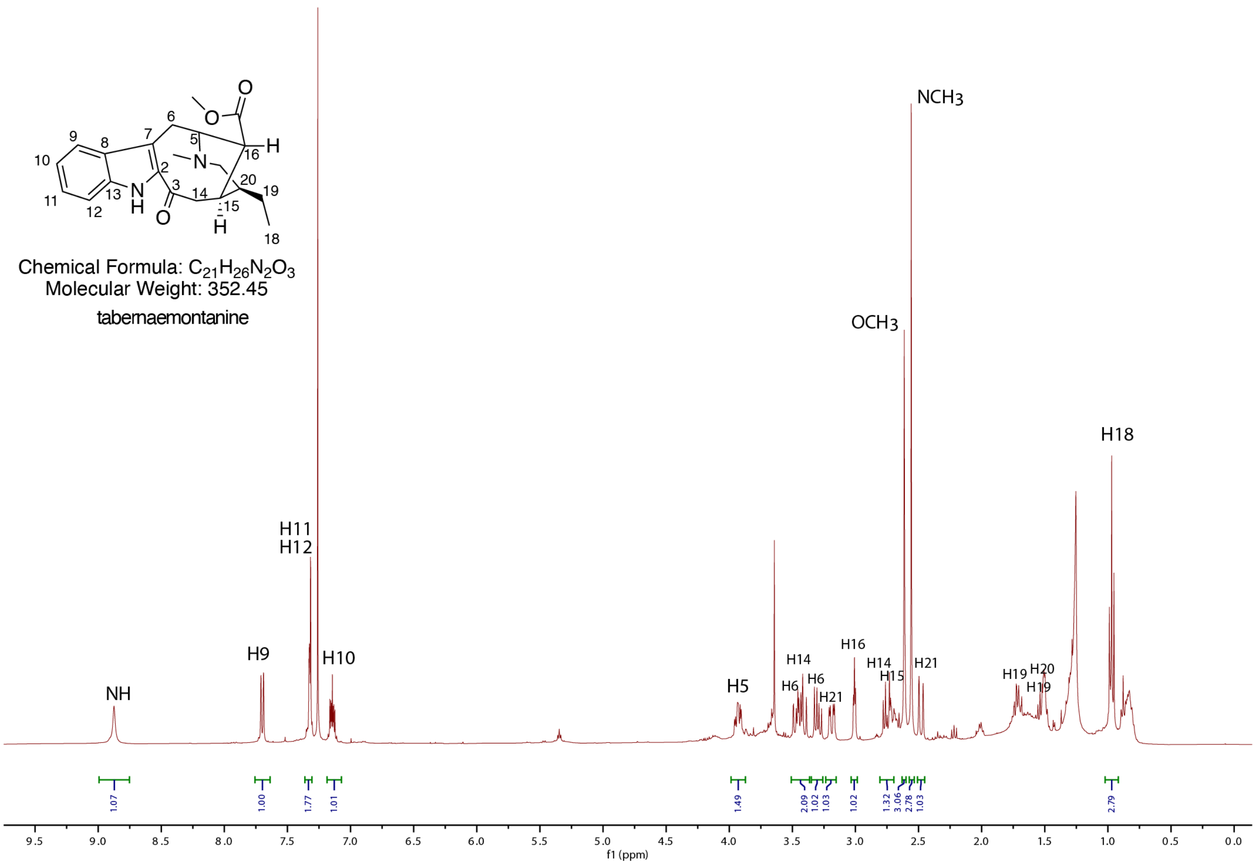

Supplementary Figure 20. ^{13}**C NMR spectra for tabernaemontanine in CDCl**$_3$**.**

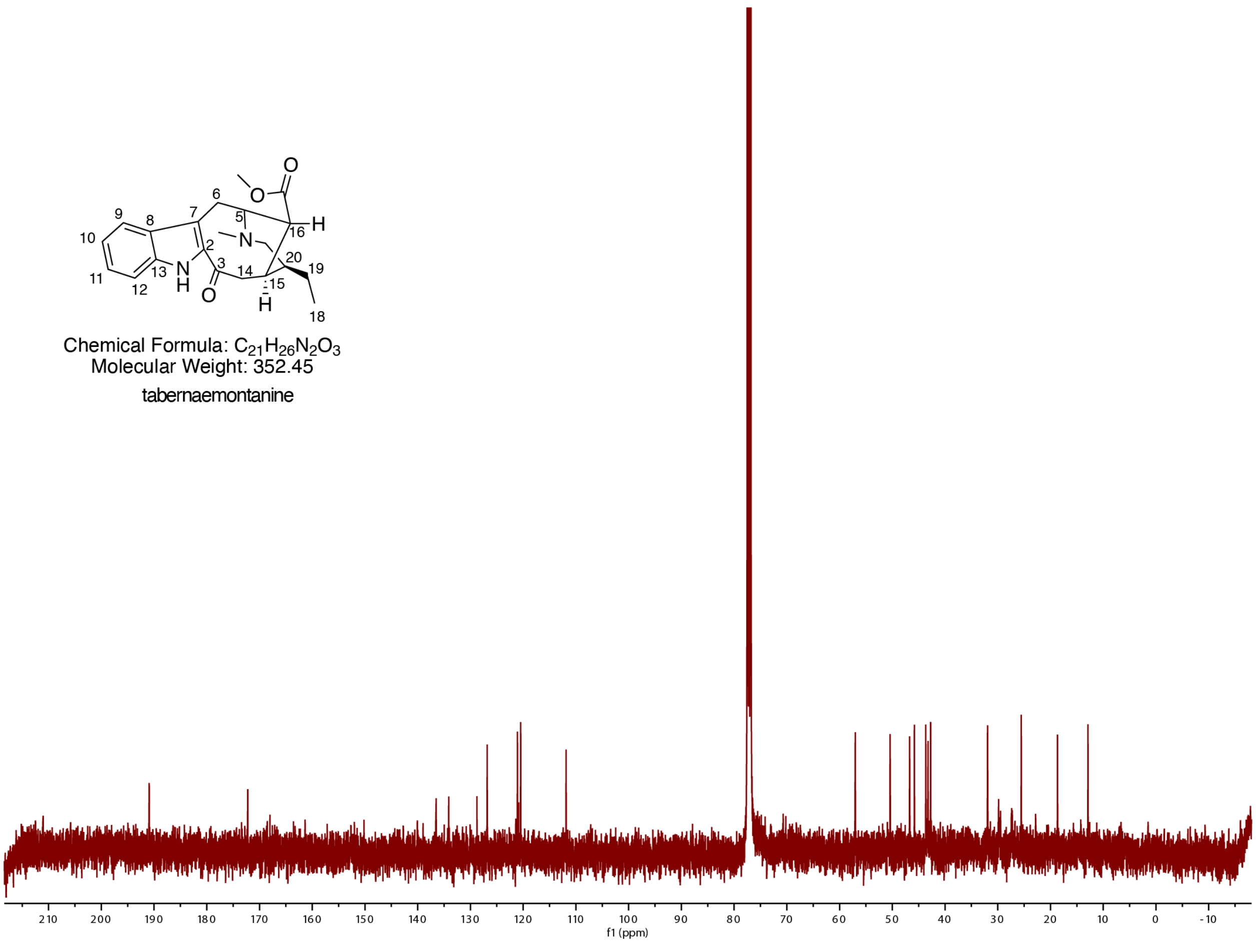

Supplementary Figure 21. HSQC NMR spectra for tabernaemontanine in $CDCl_3$.

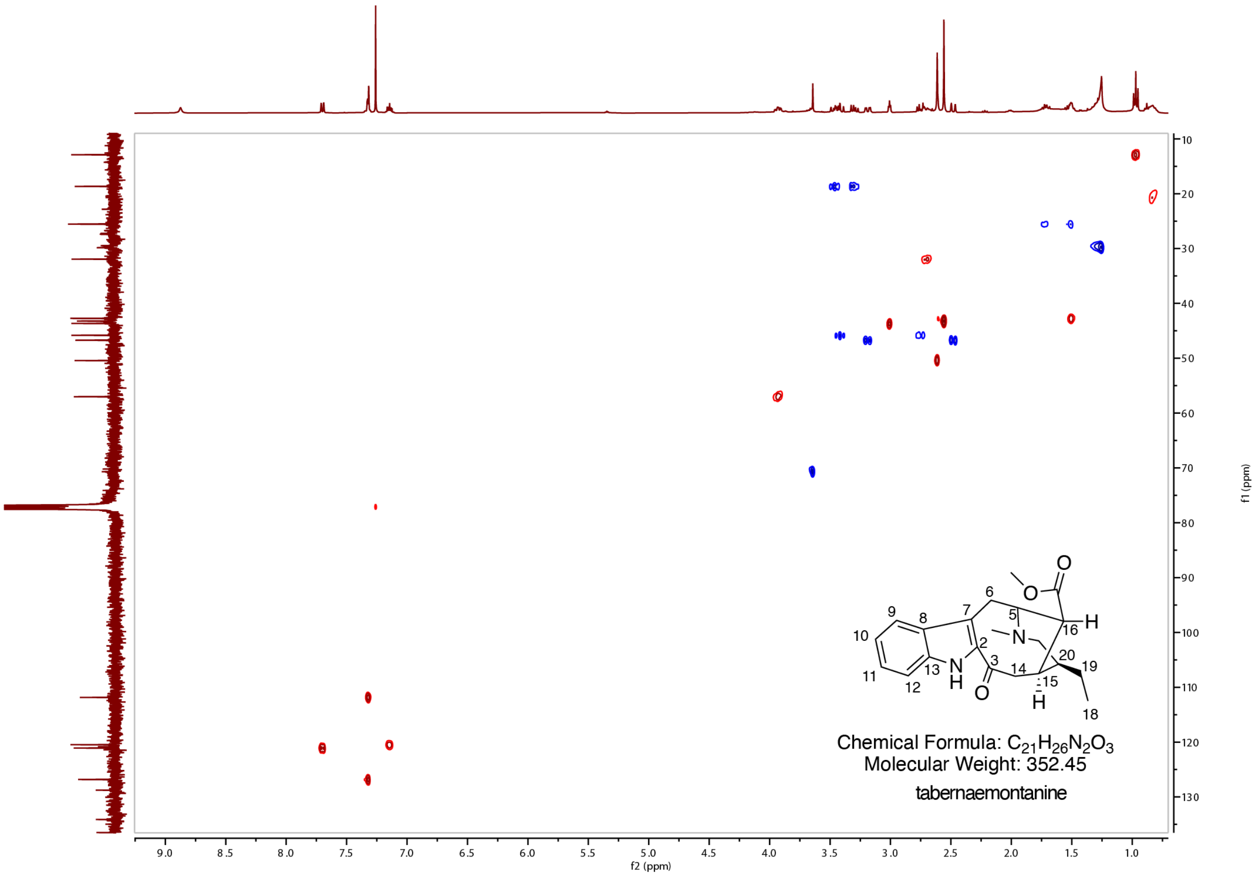

Supplementary Figure 22. HMBC NMR spectra for tabernaemontanine in $CDCl_3$.

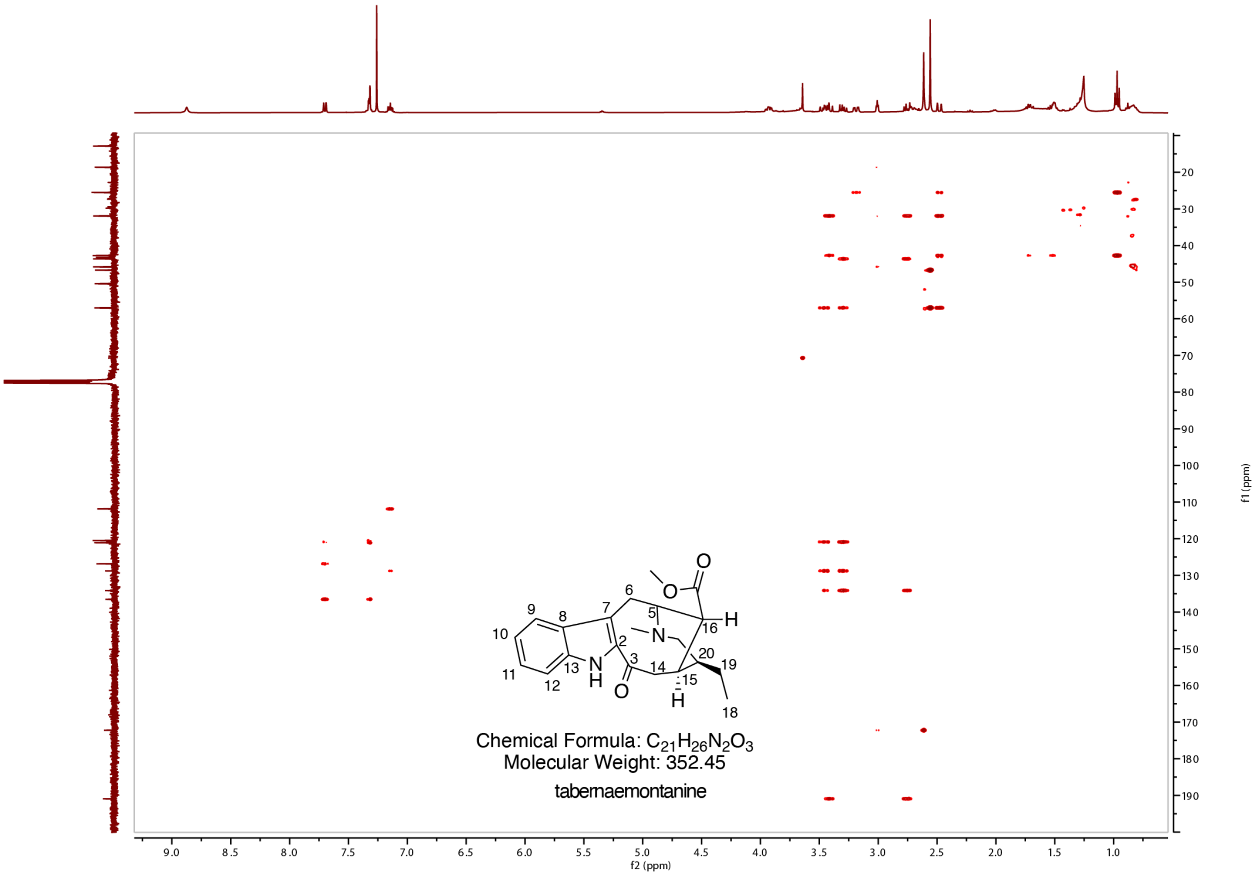

Supplementary Figure 23. NOESY NMR spectra for tabernaemontanine in $CDCl_3$.

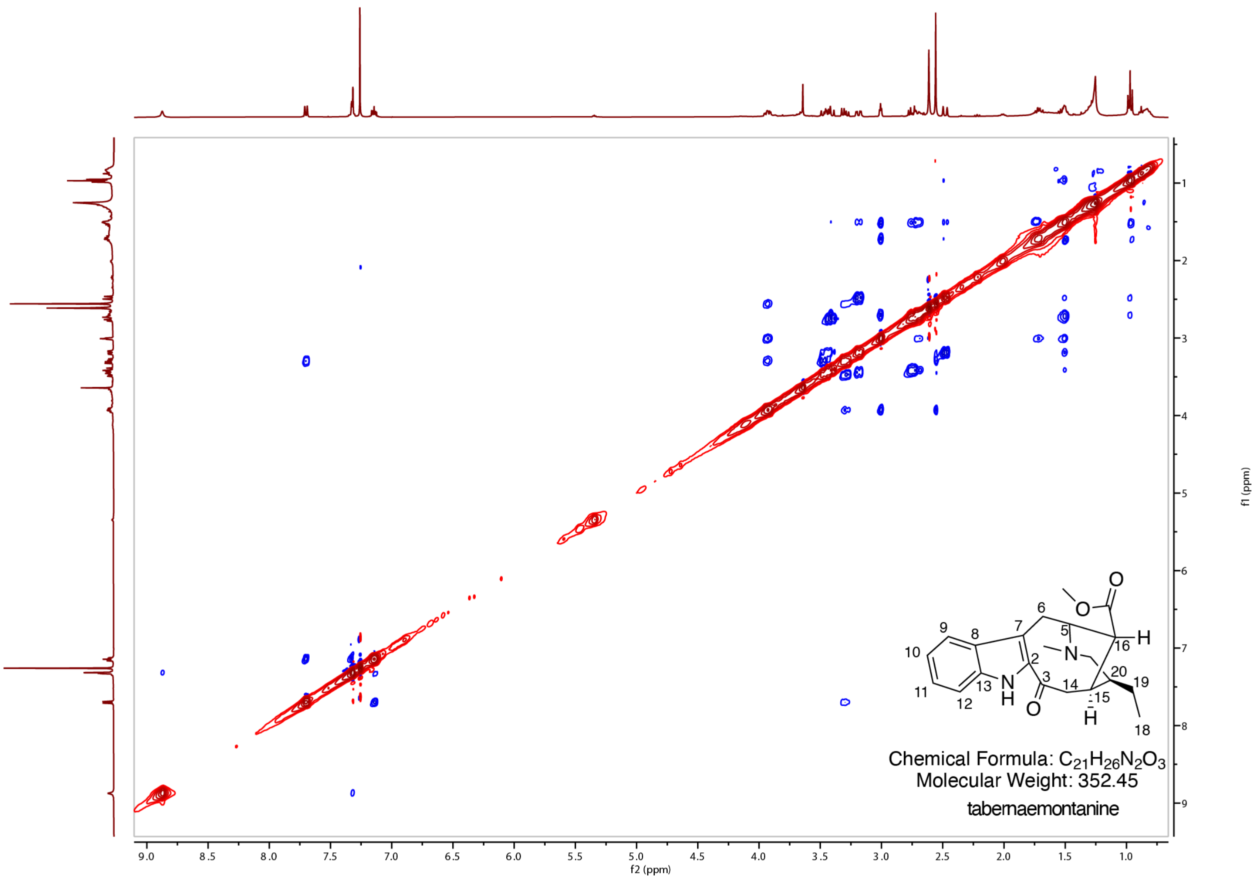

Supplementary Figure 24. COSY NMR spectra for tabernaemontanine in $CDCl_3$.

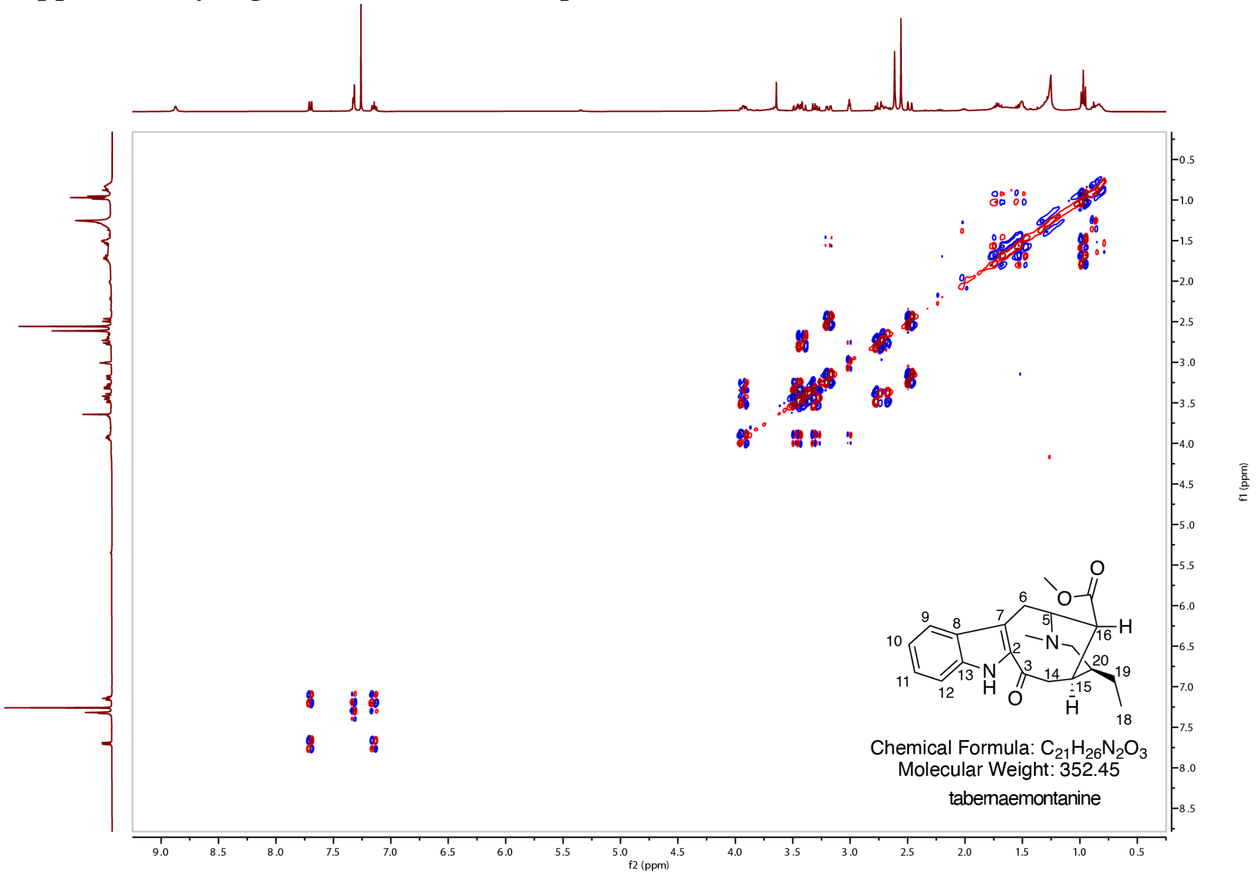

Supplementary Figure 25. SDS-PAGE gels of N-terminally 6X-Histagged TePeNMT and CrPeNMT

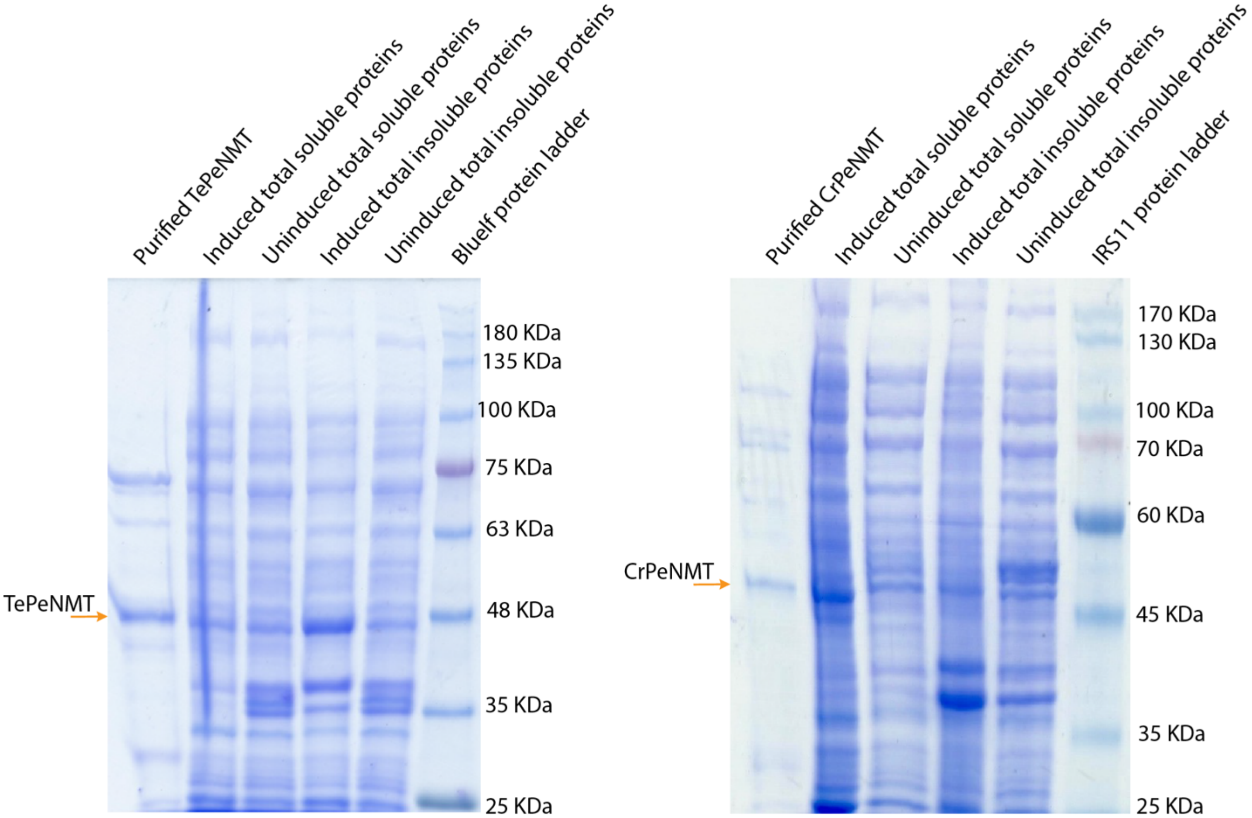

SDS-PAGE gels of *N*-terminally 6X-Histagged TePeNMT (left panel) and CrPeNMT (right panel) proteins purified by Ni-NTA affinity Chromatography. Orange arrows indicate recombinant enzymes.

Supplementary Figure 26. 2D ligand interaction maps for *S*-adenosylmethionine (SAM) and perivine at the enzyme active sites of TePeNMT and CrPeNMT.

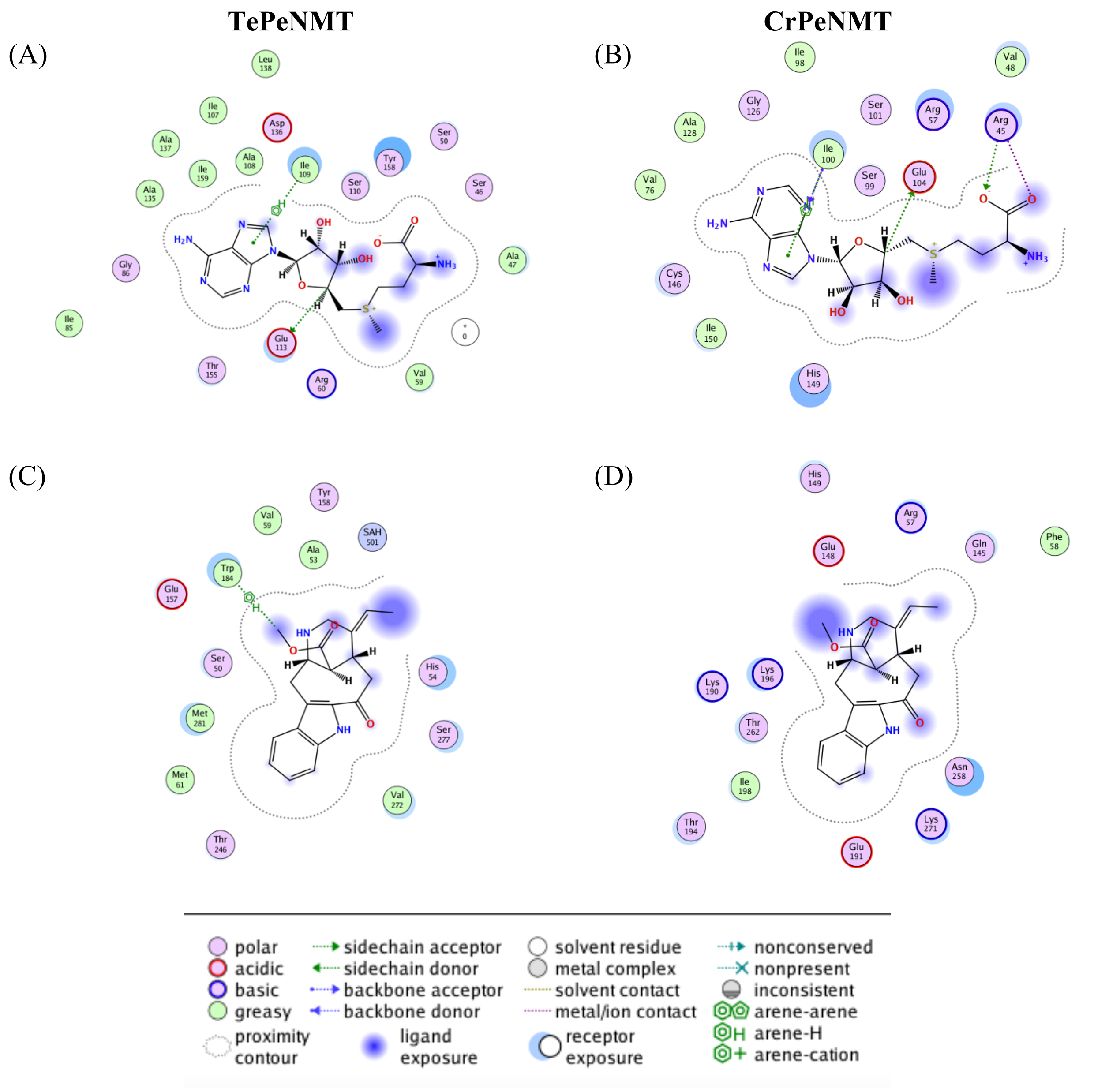

(A) and (B): active site amino acid residues interacting with SAM. (C) and (D): active site amino acid residues interaction with perivine, (A) and (C): enzyme active sites of TePeNMT. (B) and (D): enzyme active sites of CrPeNMT.

Supplementary Table 1. ^{1}H NMR chemical shifts of vobasine, dregamine, tabernaemontanine, and apparicine in $CDCl_3$.

	Vobasine This study	Vobasine Reference[1]	Dregamine This study	Tabernaemontanine This study	Apparicine This study	Apparicine Reference[2]
NH	9.02 s	-	8.87 s	8.88 s	7.87 br s	7.84 s
3	-	-	-		3.07 dddd 3.44 ddd	3.07 dddd 3.42 ddd
5	3.97 ddd	-	3.97 ddd	3.93 ddd	-	-
6	3.44 dd 3.51 dd	-	3.35 m 3.35 m	3.30 dd 3.46 m	4.27 d 4.52 d	4.28 d 4.51 d
9	7.71 d	7.71 d	7.70 d	7.70 d	7.42 d	7.42 d
10	7.15 ddd	7.15 m	7.15 ddd	7.14 ddd	7.06 ddd	7.06 ddd
11	7.34 dt	7.15 m	7.33 ddd	7.32 ddd	7.18 ddd	7.18 ddd
12	7.34 dt	7.33 d	7.32 d	7.32 d	7.28 dd	7.28 d
14	2.73 dd 3.32 dd	-	2.68 dd 3.12 t	2.75 dd 3.42 dd	1.90 dddd 2.18 dddd	1.89 ddt 2.16 dddd
15	3.78 ddd	-	2.89 dddd	2.70 m	3.92 br s	3.92 br s
16	2.83 dd	-	2.83 t	3.01 t	-	-
17	-	-	1.02 t	0.97 t	5.27 s 5.40 s	5.26 s 5.39 s
18	1.71 dd	1.71 dd	1.35 m 1.35 m	1.53 dd 1.71 dd	1.47 dd	1.46 dd
19	5.46 q	5.45 q	1.89 dddd	1.51 m	5.28 q	5.25 q
21	2.98 d 3.85 d	-	2.59 dd 2.76 d	2.48 d 3.19 dd	3.22 d 3.83 dt	3.20 d 3.82 dt
OMe	2.60 s	2.61 s	2.64 s	2.61 s	-	-
NMe	2.65 s	2.65 s	2.62 s	2.56 s	-	-

Supplementary Table 2. ^{13}C NMR chemical shifts of vobasine, dregamine, tabernaemontanine, and apparicine in $CDCl_3$.

	Vobasine This study	Vobasine Reference[3]	Dregamine This study	Tabernaemontanine This study	Apparicine This study	Apparicine Reference[2]
2	134.3	134.1	134.2	134.1	145.1	145.2
3	190.3	190.1	191.7	190.9	45.3	45.3
5	57.4	57.2	56.9	57.0	-	-
6	20.6	20.3	20.3	18.7	54.3	54.2
7	120.5	120.4	120.5	120.9	110.1	111.5
8	128.7	128.5	128.7	128.8	129.1	129.0
9	121.1	120.8	121.1	121.1	118.8	118.6
10	120.6	120.4	120.6	120.5	123.2	119.3
11	126.9	126.7	126.9	126.8	119.6	123.0
12	111.9	111.7	111.9	111.9	110.4	110.2
13	136.6	136.3	136.5	136.5	137.0	137.4
14	43.2	43.0	39.4	45.8	29.6	29.6
15	30.6	30.4	30.8	32.0	41.3	41.2
16	46.7	46.6	49.3	43.7	135.8	135.6
17	-	-	-	-	112.5	112.2
18	12.5	12.3	11.7	12.9	12.7	12.6
19	121.0	120.8	23.7	25.5	120.8	120.1
20	136.0	135.8	43.7	42.6	131.5	131.3
21	52.0	51.8	48.9	46.7	54.2	54.3
C=O	171.5	171.2	171.6	172.2	-	-
OMe	50.6	50.3	50.5	50.4	-	-
NMe	42.5	42.3	42.7	43.2	-	-

Supplementary Table 3. MIA substrates tested for TeMT1-3 in this study

MIA type	heteroyohimbine	corynanthe	yohimbe	iboga	Aspidosperma	sarpagan	akuammiline	Vincamine
MIA tested for TeMT1-3 in this study	Ajmalicine<br>Reserpiline	Corynantheidine<br>Paynantheine<br>Speciociliatine	Yohimbine	Catharanthine<br>Coronaridine<br>Heyneanine<br>Voacangine<br>Voacangarine	Tabersonine<br>Vincadifformine<br>19-hydroxytabersonine<br>2, 3-dihydrotabersonine<br>Minovincinine	Pericyclivine<br>Strictamine<br>Vomilenine<br>Ajmaline	Picrinine	Vincamine

Supplementary Data 1. Multiple alignment for NMTs used in the phylogenetic analysis of this study.

>Hp9OMT
--------------------------------MYVVISHRYICHPSKVSLSSMFSNQTHPLAMDDAQQNYLRVLEM
GCTQILHAVFTTVIE-LNVFEIIAKAGPEAQLSAAEISS--HL--PTQN--------QQAPAILERMLQLLASYSV
LKCVHVSNQDGRGTRL--YGLTPMCRYLVAD-----TMGISM-GPAML----------CYTDKAMADSWSYLKDAV
L--EGK-IPFNKANKMDLFEYFG--KSSTLNETFNQAMHSETFFVLRAVLQNY-KGFEALKELVDVGG-------G
LGITLNAIISKYPGIRGINFDLPQVIKDAPIRTGVENLPGDMFEYVPKGEAILLKNILHDWTDEHCLKLLKNCYNA
LPEH---GKVIVIEMILPTSPENDLLSRAVFFVDIMMLALTSGGRERTLKEFDALAKGAGFIACKLVCQTFGYGIL
EFYKSSTLNSSEIP
>CpOMT1_MW456557
--MATSENSTKLLRAQAHIWNQ
TFIFKNSASLKCAIQ-LGIPDVIQ--KHGKPITLSDLIS--AL--PINP--------SKALYI--DRLMRILVNNG
F---LAQEKEGY------YTLTSAGRLLLKDDETLSAREFVL----------------MVLDPALVKPWSVLTEWF
K--NDDRSPFDTAHGKSFWEYMA--DDPKLGKLFNDAMASDSQLITKVLITECRYVFEELTSLVDVGG-------G
TGTVARSIAKMLPHLNCIVFDLPHVVANQEGTENLDFVAGDMFEKVPPTNAILLKWILHDWSDEDCVKILKNCKKA
IPGRDKGGKVIVIDMVMDSQLIKDDESVEAQICFDMEMLVLFRSKERTEKELATLFWDAGFSRYKVLPVLGTRGLI
EVY----------P
>Cr16OMT
--MDVQSEEFRGAQAQIWSQ
SCSFITSASLKCAVK-LGIPDTID--NHGKPITLSELTN--ALVPPVHP--------SKAPFI--YRLMRVLAKNG
F-CSEEQLDGETEPL---YSLTPSSRILLKKEPLN-LRGIVL----------------TMADPVQLKAWESLSDWY
QNEDDSSTAFETAHGKNFWGYSS--EHMEHAEFFNEAMASDSQLISKLLIGEYKFLFEGLASLVDIGG-------G
TGTIAKAIAKNFPQLKCTVFDLPHVVANLESKENVEFVAGDMFEKIPSANAIFLKWILHDWNDEDCVKILKSCKKA
IPAK---

>TiN10OMT_MH454075
--DAMKSAELFKAQAHIFKQ
VFCFTNGASLKCAVQ-LGIPDAID--NHGKAMTLSELTD--AL--PINP--------SKAPHI--HRLMRILVTAG
F-FVEERLGNGKEEKANGYALTPSSRLLLKNKPLS-LRASAL----------------TMLDPVTVKTWNALSEWF
Q--NEDQTAFETAHGKNMWDFFA--EDPGLSKKFNESMASDSQLVTEVLVTKCKFVFEGLTSMVDVGG-------G
TGTVAGAIAKTFPSLRCTVFDLPHVVANLEPTENLDFVAGDMFGKIPPANAIFLKWVLHDWNDEDCVKILKNCKRA
IPGKEKGGKVIIVDIIMETEKHDIDEFDYAKMCMDMEMLVLCNSKERTEKELAMLVSEAGFSGYKIFPVLGIRSLI
EVY----------P
>RsANMT_KC708445

------MAENQEALA--EFYDKGV--GVWDNLSREHMHF--GYYAPGAT-ATIGG--HRASLV--RLIDEALCFAE
F-PD-DPEKKPRNMLDVGCGIGGTCLHVAKKYDIQ-CKGINI-SPEQVKIAQGLAAAQGLESKVSFDVGDALDMPY
P--DGA---FDLVLSIHCIEHLQ--DKEKFIREMVRVAASGATII---ILSHVHRDLS----------------P
SEQSLKPQEERVLRKIGSSVQ-AWFCPL------SNYV--SLLAPLP----------VEVIKIADWSRNIDPSSRL
MLKV-------AFSVKGIVSNLMKGVQGWTAIKNVLPMKLLHKALHDGLVKFVVLTCRKSN--------------
----
>CrPeNMT_TMT1_KC708453

-------MGEKEAVA--ELYDKVTSNGILEELFGEHLHD--GYYEVGTV-ATISA--HRAAVV--RIIDEALRFAD
V-FTDDQAKKPRNMLDVGCGKGGTCVHIARKYDIQ-CTGISI-SPDEIQCAKHLAASQGLENKVSFDVGDALNMRY
S--DGS---FELIFVIQCIEHIQ--DKEKFIREIVRVAAPGAQIV---IISTACRNLS----------------P
SEKSLKPKEEKTLKKICNYLHLSGFCSL------SDYS--NWLTPLP----------IEDMKIADWTQNAAPFYTL
LLRE-------AFSIKGFISLLMNG--GWTAVKVILGMKTIHEAIENDLLKIVAVTFRKTK--------------
----
>CrDhtNMT_ADP00410

------MEEKQEKVA--EFYDKVT--GAWDLFYGVHLHD--GYYEPGTT-ATMAI--SQDAVI--RMIDELLRFAG
V-SE-DPAKKPRSMLDVGSGLGGTCVYVAKKYDIQ-CTGITI-SPNQVKYAQDYAATEGVENKVSFDCGDALDMPY
S--DGK---FDVVFTINCIKHVH--DKEKFIREMVRVAAPGAAII---IASQAHPNLS----------------P
GE-SLKPRDKKILQKICDGAGAVSLCSS------DDYV--RWLTPLP----------VKEIKAADWTQNITPLYPL
LMKE-------AFTWKGFTSIVLKG--GWRAINLINAVRLVAKAANDGILKFAVVTGRKSI--------------
----
>CrPiNMT_TMT2_A0A8X8M4T9

---MAAVVEKQEAVA--EFYDNST--GAWEELFGEHLHD--GYYEPGTT-ATIPA--HRAAVV--RMIDEALRFAG
V-STDDPAKKPRNLLDVGCGLGGTCLYLAKKYDIK-CTGITI-SPEQVKCAEDLAAAQGLENKVSFDVGDALDMPY
Q--DGE---FDVVFTLQCIDHVQ--DKEKFIREMVRVGSPGAAIV---VITYTHRDLS----------------P
TEQSLKPHEIKTLKKICDNIVLSSISST------HDYV--NWMTSLS----------LKDIKTADWTQNIIPFYPL
LFKV-------SFSMKGFISLLMKG--GWSAIKVVLAVKMMSKAIDDGLLYYTAVSGRKPN--------------
----
>RsNNMT_KC708449

------MAEKQQAVT--EFYNNTSPRGAWEFLLGDHLHE--GFYDPGTT-ATISG--SQAAAA--RMIDEALRFAN
I-YD-DPSKKPKNMLDIGCGVGGTCVHVAKQYGIQ-CKGITL-SPEEVKCAQGIAKAQGLEEKVSFDVGDALNLPY
K--DGT---FDLVLTIECIEHVQ--DKEKFIREMIRVAAPGAPIV---ILSYAHRNLS-----------------P
SAESLKPDEKKVLKKICDNLALSCLCSS------ADFV--RWLTQLP----------AEDIKTADWTQNTSPFFPL
LMKE-------TFTWKGFTSLLMKG--GWTAIKELLALRMMSKAADDGLLKFVAITCRKSK---------------
----
>VmPiNMT_KC708450
---MYTCSIIIYILTFWQLSKIKKQVAAA
EKQVMTVTEKQEAVA--EFYDKST--DAWEVFFGEHLHD--GFYEPGTT-ATIPG--SKVAVV--RMIDELLRFAG
I-SD-DPEKKPKTMLDVGCGLGGTCLHVAKKYDIK-CTGITI-SPEQVKCAQDLAATQGLESKVSFDVGDALDMPY
K--DGT---FDLVFTIQCIEHIQ--DKEKFIREMVRVAAPGAPVV---IAGYAARNLS-----------------P
SEESLKPEEKMVLEKICDHIVLSWLCST------GDYV--KWLTPLP----------VQDIKVWDLTQNITPFYPL
CIKE-------AFTWKSFTSLLKMG--GWSAIKVVFAVKMMAMAAEEGLLKFAAVTCRKSK---------------
----
>CrPiNMT_TMT4_A0A8X8M501

------MAEKQQAVA--EFYDNST--GAWEVFFGDHLHD--GFYDPGTT-ATIPA--SRAAVV--RMIDEALRFAN
V-ST-DPAKKPRNMLDVGCGIGGTCLYVAKKYDIQ-CTGITI-SPEQVKCAQGFAAAQGLENKATFDCGDALNMPY
K--DGT---FDVVFTIQCIEHIQ--DKEKFIREMVRVAAPGAAIV---IVSYGHRNLS-----------------P
GEESLKPEEKKTLKKICDNIVLSWLCSS------ADYV--RWLTPLP----------VQDIKTADWTQNIQPFYPL
LFKE-------AFTWRGFTSLLMKG--GWSAIKVVLAVKVMAKAADDGLLKFMAVTCKKSK---------------
----
>RsPiNMT_KC708448

------MAEKQQAVA--EFYDNST--GAWEVFFGDHLHD--GFYDPGTT-ATIAG--SRAAVV--RMIDEALRFAN
I-SD-DPAKKPKTMLDVGCGIGGTCLHVAKKYGIQ-CKGITI-SSEQVKCAQGFAEEQGLEKKVSFDVGDALDMPY
K--DGT---FDLVFTIQCIEHIQ--DKEKFIREMVRVAAPGAPIV---IVSYAHRNLS-----------------P
SEGSLKPEEKKVLKKICDNIVLSWVCSS------ADYV--RWLTPLP----------VEDIKAADWTQNITPFYPL
LMKE-------AFTWKGFTSLLMKG--GWSAIKVVLAVRMMAKAADDGVLKFVAVTCRKSK---------------
----
>CrNMT_ADP00411
--MAKKSSV
EQLQEKDQEKFKGVA--DLYGGTS--RAWEDILGDHWHH--GYYDPGST-VSKSD--NAAALI--RMIDEVLRFGS
VFSAENQENKPKRILDIGCGIGGTCTYLARKYGAH-CTGITI-SSGEVERAQALATAQGLQEKVSFEVANALALPF
P--DGQ---FDLVWCMETAEHIP--EKEQLVKEIVRVAAPGGQII---LTSWCHRNLL-----------------P
SEQSLPLDEQKFIKKMCDLVLMHPFCSV------NEYI--NLFQSHH----------VEDMKTDDWCEFAKPFWTA
MVGS-------ACTIKGFFSLLWIG--GWNSLKFVMGSNMMIKAHKKGLVKLLVLSCRKPR---------------
----
>TePeNMT_PP067959
---MAET
SKSAKADQERVNGVA--EIYNETA--GTMERMCGEHIHH--GYYEPGSAPASMSA--HPAAQV--RMIDEVLRSAS
V-SV-DPEKRPKSILDIGCGIGGTCLHLARKYGAQ-CKGIAI-SPVEVERAQALAAAQGLQGQVSFAVADALSLPF
P--EGQ---FDLIWCMETSEYIA--DKEKLVSEFVRVAAPGGTII---LASWCHRDLS-----------------P
SEKSMKPDEEKLIKKICDLNLFPPFCSS------SDYI--KLFKSHP----------LEDIKAEEWSQYASPTWPL
LIGE-------TFTIKGFISLLWTG--GWKAVKLVTSVQTMMKAHQKGALKFAVISCRKPK---------------
----
>TeNMT3_PP067961

----------MKGTR--ESYDEKY--GRWEALWGEHLHN--GYYEPGSI-VYLSD--HLAAQV--RMIEEVLRFAS
V-SD-DPERKPRSILDVGCGVGGSSLYLASKYDAL-CKGINI-SPFEVERAQALAAAQGLQSKVSFQVADALAQPF
S--DGQ---FDLVWCLESAEYVA--DKEKLVSEMVRVAASGATIV---IASWCHRDLS-----------------P
SEQSLKPDEEKLMKKLCDSMLLSPLCST------ADYI--KLLKSHS----------FEDIKVADWSEHASPFWPV
MYRS-------TFTWKGFVSLLRTG--GWKAMKLVLAFKTMMEGQKKGLFKLAIIACQKPENAHKDFATFDVSAK-
----
>AthγTMT_AAD02882
-------------------------MKATLAAPSSLTSLPYRTNSSFGSKSSLLFRSPSSSSSVSMTTTRGNVAV
AAAATSTEALRKGIA--EFYNETS--GLWEEIWGDHMHH--GFYDPDSS-VQLSDSGHKEAQI--RMIEESLRFAG
V-TDEEEEKKIKKVVDVGCGIGGSSRYLASKFGAE-CIGITL-SPVQAKRANDLAAAQSLSHKASFQVADALDQPF
E--DGK---FDLVWSMESGEHMP--DKAKFVKELVRVAAPGGRII---IVTWCHRNLS-----------------A
GEEALQPWEQNILDKICKTFYLPAWCST------DDYV--NLLQSHS----------LQDIKCADWSENVAPFWPA
VIRT-------ALTWKGLVSLLRSG---MKSIKGALTMPLMIEGYKKGVIKFGIITCQKPL---------------
----
>HaγTMT_ABB52798
---MATTAVGVSATPMTEKLTA
ADDDQQQQKLKKGIA--EFYDESS--GMWENIWGEHMHH--GYYNSDDV-VELSD--HRSAQI--RMIEQALTFAS
V-SD-DPEKKPKTIVDVGCGIGGSSRYLARKYGAE-CHGITL-SPVQAERANALAAAQGLADKVSFQVADALNQPF
S--DGK---FDLVWSMESGEHMP--DKLKFVSELTRVAAPGATII---IVTWCHRDLN-----------------P
GEKSLRPEEEKILNKICSSFYLPAWCST------ADYV--KLLESLS----------LQDIKSADWSGNVAPFWPA
VIKT-------ALSWKGITSLLRSG---WKSIRGAMVMPLMIEGFKKDVIKFSIITCKKPE---------------

----
>PfγTMT_AAL36933
------MAEAVTPGICTTGWRRGGVHAPTYNISIKPATALLVGCTTKTKSITSFSTDSLRTRGRARRPTMSLNAAA
AEMETEMETLRKGIA--EFYDESS--GVWENIWGDHMHH--GFYEPAAD-VSISD--HRAAQI--RMIEESLRFAS
F-SPITTTEKPKNIVDVGCGIGGSSRYLARKYGAKLSRAITLSSPVQAQRAQQLADAQGLNGKVSFEVADALNQPF
P--EGK---FDLVWSMESGEHMP--DKKKFVNELVRVAAPGGRII---IVTWCHRDLS----------------P
SEESLRQEEKDLLNKICSAYYLPAWCST------ADYV--KLLDSLS----------MEDIKSADWSDHVAPFWPA
VIKS-------ALTWKGITSLLRSG---WKTIRGAMVMPLMIEGYKKGVIKFAIITCRKPAS-------------
----
>CaγTMT-like_XP_027097246
----------------------------------MLQILSTKQLSTRFIHTGARILNKKEMAVRQASLGGVDVD
LETKTNVEKLKRGIA--EFYDESS--GIWEDIWGDHMHH--GFYDPNST-VSLSD--HRAAQV--RMIEEALKFAS
V-PE-DPMKKPKSIVDVGCGIGGSSQYLARKYGTE-CTGITL-SPVQAERARALAAAQGLENKVSFEVADALNQPF
P--DGK---FDLVWSMESGEHMP--EKAKFVNELARVAAPGARII---IVTWCHRNLS----------------S
SEQSLNPDEKKLLDKICDAYYLPTWCST------DDYV--KLLQPLS----------LQDIKAADWSEYVAPFWPA
VIRS-------ALTWKGITSLILSG---WKTIKGAMVMPLMMKGYKKGLIKFAIITCRKPE--------------
----
>TeNMT2_ PP067960

------------------------------MWGDHMHH--GYYEPGTN-VSVID--HQAAQI--RLIEEVLRFAS
V-SD-DPIKKPKSIVDVGCGIGGSSRYLARKYGAK-CKGITL-SPVQAQRAQALAVAQGLQSKVSFEVADALAQPF
P--DGQ---FDLVWSMESGEHMP--DKEKFVRELARVAAPGATII---IVTWCHRDLS----------------P
SEQSLKPEEKKLLNKICDSYYLPAWCST------ADYV--KLLESVS----------LVDIKTADWSEYVAPFWPA
VIRS-------ALTWKGFASLLRSG---WKTIKGALVMPLMIEGFKKDLIKFAVITCRKPE--------------
----
>CrγTMT_TMT3_A0A8X8M4W6
MAAAPVFFPSSVVVSVYRAHGYAFSCSSLLSQLPAIKLSSYRPTRTDHTAITLLPNSNRIASRLQAMQSPTFEVNG
DGKTEEVEKLQKGIA--EFYDESS--GIWEEIWGDHMHH--GFYDPGTT-VSLAD--HRTAQI--RMIEEALRFAS
V-SD-DPMKKPKRIVDVGCGIGGSSRYFARKYDAE-CKGITL-SPVQAKRAQALAVAEGLQGTVSFEVADALAQPF
P--HGQ---FDLVWSMESGEHMP--DKEKFVRELVRVAAPGATII---IVTWCHRDLS----------------P
SEHSLKPQEQKLLNKICDSYYLPAWCST------ADYV--KILESLF----------LEDIKRADWSEYVAPFWPA
VIRS-------ALTWKGFTSLIRSG---WKTIKGAMVMPLMIEGFKKDVIKFSVITCRKPE--------------
----
>CreiNMT_XP_001695187
-----------------YNALVKTFIGAVENGYVPDFILRTGIRFLLSNRVAEPFSPSLPSDLNGQLQQTVAFVN
DLKTMPIAVNTSEAN-EQHYEIPT--PYYLLVLGSHLKYSSCLYRSPRE-------SLEQAEY--NMLDLYCERAQ
L-------RPGQRVLELGCGWGSFSLFAAARYPASTFFAVSN-SATQKAFIDGEAAKRGITN-LTVLTANMVDFEA
PTANGL---FDRVVSIEMFEHMK--NYQRLLRKVSGWLAAEGKLF---VHIFLHRTTPYHFEVQSEEDWMSKYFFT
GGTM--PSSDLLLYFQDDLVIRNHWYVN---GRHYSRTLEDWLVRH-------------DRNSREVRKILTTAYTG
SKDS-------ANVWFHR----------WRIFYIACRRLFNYKGGEEWGVGHFLFEQRG-----------------
----
>PsRNMT_KX369612
--------------STTMETTKISQQDDLWKNMELGQISDEEVRRLMKIGIEKRIKWGTKPTQQEQLAQLLDFNK
SLRGMKMATEIDTLENHKIYETPE---SFNQIIGGKESA--GLFTDETT-TTMEE-----ANT--KMMDLYCERAG
L-------KDGHTILDLGCGAGLLVLHLAKKYKKSKITGITN-TSSHKEYILKQCKNLNLSN-VEIILADVTKVDI
---EST---FDRVFVIGLIEHMK--NFELFLRKISKWMKDDGLLL---LEHLCHKSFSDHWEPLSEDDWYAKNFFP
SGTLVIPSATCLLYFQEDVTVIDHWILS---GNNFARSNEVILKRI-------------DGKIEEVKDIFMSFYGI
GREE-------AVKLINW----------WRLLCITANELFKYNNGEEWLISQLLFKKKLMTCI-------------
----
>CjCNMT_BAB71802
-------------MAVEAKQTKKAAIVELLKQLELGLVPYDDIKQLIRRELARRLQWGYKPTYEEQIAEIQNLTH
SLRQMKIATEVETLD-SQLYEIPI--EFLKIMNGSNLKGSCCYFKEDST--TLDE-----AEI--AMLDLYCERAQ
I-------QDGQSVLDLGCGQGALTLHVAQKYKNCRVTAVTN-SVSQKEYIEEESRRRNLLN-VEVKLADITTHEM
A--ET----YDRILVIELFEHMK--NYELLLRKISEWISKDGLLF---LEHICHKTFAYHYEPLDDDDWFTEYVFP
AGTMIIPSASFFLYFQDDVSVVNHWTLS---GKHFSRTNEEWLKRL-------------DANLDVI----KPMFET
LMGN----EEEAVKLINY----------WRGFCLSGMEMFGYNNGEEWMASHVLFKKK------------------
----
>PsCNMT_AAP45316
--------------------MQLKAKEELLRNMELGLIPDQEIRQLIRVELEKRLQWGYKETHEEQLSQLLDLVH
SLKGMKMATEMENLD-LKLYEAPM--EFLKIQHGSNMKQSAGYYTDEST--TLDE-----AEI--AMLDLYMERAQ
I-------KDGQSVLDLGCGLGAVALFGANKFKKCQFTGVTS-SVEQKDYIEGKCKELKLTN-VKVLLADITTYET
---EER---FDRIFAVELIEHMK--NYQLLLKKISEWMKDDGLLF---VEHVCHKTLAYHYEPVDAEDWYTNYIFP
AGTLTLSSASMLLYFQDDVSVVNQWTLS---GKHYSRSHEEWLKNM-------------DKNIVEFKEIMRSITK-
TEKE-------AIKLLNF----------WRIFCMCGAELFGYKNGEEWMLTHLLFKKK------------------
----

Supplementary Data 2. Cartesian coordinates for CrPeNMT homology model.

N -2.1385667 21.7753992 -15.9844882
H -2.8881317 22.3451992 -15.612073
H -2.4187043 21.4412796 -16.898962
H -1.952445 20.9940613 -15.3721405
C -0.8983927 22.6008534 -16.1048954
H -1.0805973 23.3575681 -16.8709468
C 0.33849 21.7897622 -16.5659561
O 1.2553163 22.327476 -17.1874333
C -0.6053818 23.3116885 -14.7771474
H 0.3668547 23.80077 -14.8479421
H -0.5567763 22.5711725 -13.9778747
C -1.639947 24.3835703 -14.4160168
H -1.396285 24.7758699 -13.426708
H -2.6273748 23.9290305 -14.3587376
S -1.7285502 25.7740286 -15.5733039
C -2.9534829 26.7844932 -14.6928753
H -3.1342375 27.7045542 -15.2488035
H -2.5798462 27.0332981 -13.6984196
H -3.8891334 26.2323095 -14.5953778
N 0.3673335 20.4886197 -16.2657559
H -0.4109708 20.1080689 -15.7405706
C 1.3938537 19.5859753 -16.7771625
H 1.3580658 19.6103407 -17.8672226
H 2.3753087 19.9422459 -16.4680576
C 1.2496557 18.1385969 -16.3224611
O 0.7986647 17.8537127 -15.2154758
N 1.7628938 17.2390447 -17.1522057
H 2.1121366 17.5432004 -18.0501647
C 2.0288584 15.8382482 -16.8338954
H 1.9028599 15.7027836 -15.7604402
C 3.4957816 15.5076051 -17.1394012
O 4.119702 16.1812749 -17.9609391
C 0.9990926 14.9223526 -17.5032517
H 1.2572628 13.8928438 -17.2749979
H 0.0423459 15.1358366 -17.026009
C 0.8149397 15.0455348 -19.020348
H 1.6157817 14.5050359 -19.5288645
H 0.8699863 16.0954136 -19.3176114
C -0.5554566 14.4754363 -19.4180847
O -1.297864 15.2053148 -20.1119062
O -0.875195 13.3478418 -18.9747065
N 4.1061184 14.7016468 -16.2632362
H 3.5442127 14.1102083 -15.6715657
C 5.5629684 14.6122266 -16.0983634

H 6.0446756 14.8186955 -17.0600251
C 5.9695982 13.2129132 -15.6736854
O 5.3463653 12.639753 -14.786269
C 6.0594875 15.5900786 -15.0182406
H 5.5966651 15.3285324 -14.0641537
H 7.1389711 15.4636292 -14.915048
C 5.7688522 17.0627619 -15.3210483
H 6.199365 17.3109927 -16.2919241
H 4.6914859 17.2284646 -15.351451
C 6.3694499 17.9615862 -14.2310701
H 7.4516987 17.8220036 -14.192695
H 5.9514537 17.6743696 -13.2638997
C 6.0413128 19.4366002 -14.4861744
H 6.4275873 20.029751 -13.6516003
H 4.9522862 19.5499365 -14.5026758
N 6.6253332 19.9107507 -15.7656037
H 7.6331593 19.818804 -15.7546612
H 6.3843249 20.8792368 -15.933991
H 6.2683797 19.3550584 -16.5356419
N 7.106455 12.7593095 -16.1705568
H 7.4956956 13.2759729 -16.9574346
C 7.5639831 11.383838 -16.027934
H 6.7220861 10.7521143 -15.7410336
C 8.6256294 11.3080201 -14.9245199
O 9.6775993 11.9537076 -14.9983873
C 8.0743326 10.8706348 -17.3888252
H 8.9570019 11.4416081 -17.6791626
H 8.3787829 9.8302572 -17.2664366
C 7.0169986 10.9370419 -18.5172246
H 6.1405201 10.371064 -18.1914962
H 7.409044 10.4393792 -19.4038394
C 6.5974706 12.3719046 -18.896483
O 7.4785217 13.2681533 -18.9084406
O 5.3745041 12.6072441 -18.9936412
N 8.3366279 10.5700175 -13.8495991
H 7.428984 10.1185224 -13.7997984
C 9.2537472 10.4628666 -12.7165948
H 9.411715 11.4704627 -12.3299929
C 10.6243327 9.9036971 -13.153139
O 10.6986872 8.9385328 -13.9069108
C 8.6037136 9.6350831 -11.6036772
H 8.4204313 8.619799 -11.9600852
H 7.655759 10.0896643 -11.3114457
H 9.2647124 9.595986 -10.737355
N 11.703524 10.5367378 -12.6721267
H 11.5296608 11.3030954 -12.0418231

C 13.1142625 10.3142897 -13.0702562
H 13.6516325 11.146085 -12.6173586
C 13.4243279 10.5119185 -14.5675031
O 14.4681525 10.0599575 -15.0339048
C 13.7765064 9.0619071 -12.4395625
H 14.845682 9.122573 -12.6445486
C 13.6265139 9.0675566 -10.9118445
H 12.5803642 8.9423089 -10.6318339
H 14.0018322 10.0070241 -10.506304
H 14.2061243 8.2486758 -10.485005
C 13.2962742 7.7038875 -12.9652798
H 12.2890587 7.4911381 -12.6080123
H 13.9637118 6.9192992 -12.6112727
H 13.2950871 7.7094786 -14.0550769
N 12.605294 11.3349009 -15.2378421
H 11.7369915 11.5814565 -14.7805248
C 12.7314974 11.833814 -16.6111805
H 11.8724211 12.4921825 -16.7495073
C 12.6154246 10.7757957 -17.7237248
O 13.1625965 9.6728211 -17.644985
C 13.9716254 12.728998 -16.7542726
H 14.876663 12.1276992 -16.6566322
H 13.9650252 13.5017634 -15.9855672
H 13.9695782 13.2008787 -17.7373648
N 11.9102878 11.1457732 -18.7984009
H 11.4901366 12.0606529 -18.8311174
C 11.6113386 10.2161504 -19.8849453
H 11.4683386 9.2575284 -19.3922061
C 12.7697246 10.041093 -20.8749029
O 13.4841971 10.9883786 -21.2103005
C 10.2956217 10.5834444 -20.6018401
H 9.5642602 10.8737195 -19.8541547
H 10.4613373 11.4376453 -21.2598264
C 9.7172444 9.4047201 -21.4196476
H 10.350257 9.2350742 -22.2929309
H 8.7362979 9.7002982 -21.7986705
C 9.5899082 8.089336 -20.6183328
O 8.4815808 7.7830257 -20.1380566
O 10.6205886 7.3866881 -20.4498584
N 12.8901662 8.8305339 -21.4204041
H 12.2017952 8.1247666 -21.1470783
C 13.7998552 8.4972353 -22.5092701
H 14.1771403 9.4236569 -22.9434863
C 13.0399482 7.741936 -23.6088019
O 12.2666108 6.8259826 -23.3460136
C 14.9936922 7.7225667 -21.9245386

H 15.5173435 8.3745069 -21.2235556
H 14.6001376 6.8863099 -21.3570609
C 16.0029798 7.189281 -22.9591753
H 15.4878043 6.568367 -23.6890142
C 16.7306364 8.3210255 -23.6896215
H 17.2504768 8.9574053 -22.9726436
H 16.0235264 8.9250527 -24.2541046
H 17.4549132 7.90003 -24.3877077
C 17.0509741 6.3236455 -22.2584349
H 17.5894864 6.9110609 -21.5149428
H 17.7563171 5.9395483 -22.9960493
H 16.5630007 5.4766885 -21.778768
N 13.2962296 8.1160457 -24.8611726
H 13.9627613 8.8548464 -25.0049569
C 12.7297022 7.4720004 -26.0467334
H 11.6552424 7.6561114 -26.0668372
C 12.963355 5.9483119 -26.0268958
O 14.0825531 5.4964434 -25.7932219
C 13.3669937 8.1475173 -27.2671617
H 13.1403425 9.2139322 -27.2266732
H 14.4513269 8.049724 -27.187207
C 12.9656811 7.6189319 -28.6307211
C 12.1494424 8.3971049 -29.4761888
H 11.769383 9.3526089 -29.1454154
C 11.8565891 7.9520163 -30.7798108
H 11.2562024 8.555452 -31.4472005
C 12.3673248 6.7178848 -31.2374061
O 12.1160781 6.2857259 -32.4969706
H 11.5991475 6.9488647 -33.0185918
C 13.1697677 5.9340538 -30.3847744
H 13.5492799 4.9896701 -30.7459872
C 13.4827043 6.3907892 -29.091865
H 14.1273567 5.791667 -28.4621886
N 11.8951892 5.1788313 -26.2599184
H 11.0304658 5.6427895 -26.4800882
C 11.8426092 3.7036502 -26.2137578
H 10.7811114 3.4599108 -26.2631227
C 12.3180866 3.0493723 -24.893297
O 12.588105 1.8486882 -24.8229072
C 12.4769853 3.1028593 -27.4818833
H 13.5610504 3.0598399 -27.3602348
H 12.2582179 3.7523311 -28.3311342
C 11.9188234 1.7098089 -27.8129528
O 10.6734795 1.5608478 -27.7641107
O 12.7303627 0.8270589 -28.169355
N 12.3786583 3.8077385 -23.7892188

H 12.1567416 4.7960764 -23.8565606
C 12.7236866 3.2518266 -22.4780661
H 13.5786598 2.5902866 -22.6181944
C 11.5733328 2.4149249 -21.9209309
O 10.5345585 2.9233827 -21.5059758
C 13.1586237 4.3717878 -21.5322934
H 14.0067916 4.8753226 -21.9927235
H 12.3398627 5.0838206 -21.4074609
C 13.5708913 3.8411374 -20.1519667
H 12.6903613 3.4453154 -19.6452956
H 14.3110593 3.0473288 -20.2626016
C 14.1595742 4.9730826 -19.3074994
H 13.477531 5.8273082 -19.3386531
H 15.1255757 5.265841 -19.7198963
C 14.3246592 4.5251755 -17.8558798
H 14.9647772 3.6393135 -17.8237746
H 13.3343658 4.2459127 -17.4769636
N 14.894404 5.6206571 -17.034387
H 15.8026652 5.9022239 -17.3743342
H 14.9585333 5.3479973 -16.0632593
H 14.281514 6.4323719 -17.0835563
N 11.8145313 1.1129027 -21.796752
H 12.636759 0.7428888 -22.2469702
C 10.915173 0.206206 -21.075752
H 9.9261211 0.3116122 -21.5233934
C 10.7879049 0.6022152 -19.5986625
O 11.7565775 1.0128309 -18.9601126
C 11.3345322 -1.270057 -21.22834
H 10.6296722 -1.8783407 -20.6606041
C 11.2418011 -1.7098514 -22.6941182
H 11.9608367 -1.1650701 -23.3087444
H 10.2395993 -1.5174528 -23.0780593
H 11.4468521 -2.7773516 -22.7753339
C 12.7458793 -1.5768076 -20.7095885
H 13.4907476 -1.0077613 -21.2656222
H 12.9570051 -2.6395233 -20.8284199
H 12.8214824 -1.3296274 -19.6506744
N 9.6090573 0.3641778 -19.0190877
H 8.8636369 0.042936 -19.61778
C 9.3030641 0.4995601 -17.5765515
H 8.2498576 0.2378134 -17.4888618
C 9.3464409 1.9102799 -16.9589934
O 9.2888891 2.018324 -15.7386553
C 10.0341763 -0.545724 -16.7016046
H 9.6693134 -0.4676256 -15.6770594
C 9.7794235 -1.9797159 -17.169852

H 10.215899 -2.1450359 -18.1540049
H 8.7079161 -2.1740235 -17.2032576
H 10.239578 -2.6699839 -16.4624216
O 11.4299275 -0.3613229 -16.691399
H 11.6667592 0.1678767 -17.4727227
N 9.2779939 2.9909397 -17.7450031
H 9.3809 2.8980378 -18.7455964
C 8.8924366 4.3186524 -17.2226461
H 9.484174 4.5459203 -16.3361664
C 7.3950813 4.3363773 -16.8420819
O 6.54833 4.1563548 -17.7157283
C 9.1737252 5.4060425 -18.2724269
H 8.8412563 6.3707971 -17.8859208
H 8.6096819 5.1895781 -19.1816057
O 10.5517975 5.4957348 -18.5958262
H 10.5894134 6.1754387 -19.3178923
N 7.0367162 4.4791013 -15.5515566
H 7.7637049 4.4744686 -14.8485576
C 5.6335974 4.372326 -15.0850094
H 5.0070584 4.6318068 -15.9400818
C 5.0839267 5.3356751 -13.9496445
O 4.1802507 4.8921545 -13.2441249
C 5.3501324 2.8742188 -14.7954481
H 5.6601937 2.2734319 -15.6495461
H 4.2770818 2.7385852 -14.6680957
C 6.0481724 2.3356799 -13.5563096
O 6.9355157 2.9397106 -12.9802825
N 5.6839717 1.1536912 -13.1130988
H 6.1721809 0.8278604 -12.2984982
H 4.9434468 0.6468731 -13.5561149
N 5.4209234 6.6581869 -13.7954432
H 6.1313111 7.0377768 -14.4037847
C 4.6727308 7.6690857 -12.8730958
H 3.6722803 7.2716519 -12.7031805
H 5.1665915 7.6425688 -11.902207
C 4.4605241 9.2667223 -13.2399755
O 5.4811072 9.6663177 -13.812121
N 3.2536409 10.1109433 -12.9872607
H 2.6474233 9.6614132 -12.3206553
C 2.3771526 11.4343133 -13.6922496
H 3.1658733 12.0715482 -14.0792353
C 1.0505375 12.8198719 -12.978889
O 0.738086 12.1140384 -12.0071596
C 1.9540762 10.4352165 -14.9544058
H 0.9329988 10.1097298 -14.746017
C 2.7417697 9.1177858 -15.5124699

H 3.7821969 9.3932306 -15.6797871
H 2.6711718 8.3582525 -14.7352443
C 1.9181844 11.302737 -16.1856385
H 2.9290002 11.590086 -16.4899791
H 1.3394983 12.1952761 -15.9637221
H 1.4171187 10.8031861 -17.0150758
C 2.3461044 8.3062146 -16.7841532
H 1.2838958 8.043668 -16.7411571
H 2.9411495 7.3919643 -16.8207085
H 2.5328117 8.8854332 -17.695951
N 0.2333932 14.4003043 -13.1834479
H 0.7437184 14.7653437 -13.9704046
C -1.2123799 15.955379 -12.9597387
H -1.0028175 16.3292374 -13.960482
C -2.5312399 15.1695335 -13.1010222
H -2.3258053 14.1092915 -13.2484796
H -3.0618878 15.2689053 -12.1555772
C -3.4175086 15.6977339 -14.2585728
H -3.2432872 16.7657087 -14.3985759
C -3.0783778 14.9714564 -15.5675978
H -3.2811317 13.903087 -15.4809576
H -2.0269104 15.1037593 -15.8111044
H -3.6757972 15.3801559 -16.3812018
C -4.9110054 15.512951 -13.9555661
H -5.1400087 14.4586235 -13.8201664
H -5.5040244 15.9052164 -14.7807417
H -5.172629 16.0607214 -13.0540348
C -2.0596333 17.9254177 -12.3430272
O -1.3832877 18.2475852 -11.374691
N -3.271554 19.2249776 -12.5340521
H -3.8017849 18.9469697 -13.3433045
C -4.1853409 20.9390259 -11.9780666
H -4.1201797 20.8378689 -10.8941409
C -5.8142116 21.9798425 -12.0685304
O -6.4577917 21.990306 -13.1238488
C -3.3553264 22.2522131 -12.3368714
H -2.7508435 21.9810248 -13.199742
H -3.9431639 23.1195955 -12.6376299
C -2.3935577 22.6639575 -11.2141059
H -1.5875927 23.2551301 -11.6539897
H -1.9417954 21.7737149 -10.7710014
C -3.1027948 23.4921229 -10.1365626
O -4.1338848 23.0043489 -9.6189633
O -2.6283562 24.6174618 -9.8712537
N -6.4475046 23.0719258 -11.2698861
H -5.8681384 23.316696 -10.4654281

C -7.9445496 23.8116708 -11.2481781
H -8.5313975 23.1447577 -11.8786809
C -8.4505552 25.3183528 -11.7282815
O -7.9562339 26.3541144 -11.2608184
C -8.5827225 23.7319299 -9.8142739
H -8.3098143 22.7644516 -9.3910452
H -8.1237068 24.4929663 -9.1820639
C -10.1128287 23.8553926 -9.699581
H -10.4295997 24.8665053 -9.9608487
H -10.3884539 23.7049408 -8.6526486
C -10.8425833 22.8299823 -10.5754585
O -10.9583405 23.1023724 -11.793048
O -11.2574013 21.7859035 -10.0265513
N -9.602907 25.5151302 -12.4609812
H -10.1970189 24.6947314 -12.6038089
C -10.0093518 26.7968804 -13.139427
H -9.6033406 27.6153906 -12.5465371
C -11.5373224 27.0770783 -13.3129338
O -12.3430523 26.1660776 -13.4651874
C -9.3158021 26.7963794 -14.5218177
H -8.2652178 26.5407625 -14.3723088
H -9.7616841 25.9988297 -15.1187812
C -9.3656862 28.1006173 -15.3396433
H -10.3980501 28.3527717 -15.5746801
C -8.7151959 29.2767986 -14.6053596
H -7.6855865 29.0272619 -14.3458923
H -9.2616884 29.5103425 -13.6946444
H -8.721151 30.1580805 -15.2468748
C -8.6202634 27.902131 -16.6598573
H -7.5714266 27.6723762 -16.4704162
H -8.6904418 28.808201 -17.2612677
H -9.0736317 27.0808258 -17.214682
N -11.9412789 28.3572898 -13.4297045
H -11.2467902 29.0828588 -13.3712445
C -13.3321225 28.7589476 -13.708797
H -13.9922764 28.0787705 -13.1669539
C -13.6973559 28.668285 -15.20173
O -13.2212429 29.4599915 -16.0166546
C -13.5976231 30.1744778 -13.1694646
H -13.3339687 30.2032292 -12.1112647
H -12.9526566 30.8857408 -13.6884859
C -15.0439728 30.6091374 -13.3172145
C -16.0007793 30.2075446 -12.3654889
H -15.7026398 29.6095012 -11.5164327
C -17.3497155 30.5734271 -12.5229045
H -18.084102 30.2600018 -11.7942258

C -17.7459906 31.3401123 -13.632773
H -18.784204 31.6156339 -13.7569758
C -16.7930155 31.742311 -14.5848431
H -17.0984245 32.326439 -15.4416514
C -15.4440982 31.3771052 -14.4276294
H -14.7165289 31.6734974 -15.1713742
N -14.6326318 27.780819 -15.5396291
H -14.9521194 27.1150618 -14.8471413
C -15.2390964 27.6886577 -16.8666391
H -15.735075 28.6312197 -17.0994544
H -14.4624243 27.5134405 -17.6111349
C -16.2666536 26.5596064 -16.957317
O -16.3953891 25.7524785 -16.0401148
N -16.9764725 26.4878918 -18.0819378
H -16.8289854 27.176356 -18.8044725
C -17.9575033 25.4408391 -18.3831007
H -17.7899232 24.5827215 -17.7304426
C -17.7792012 24.9927546 -19.8397808
O -17.7959146 25.8188285 -20.750926
C -19.3742105 25.9740925 -18.1133224
H -19.4072767 26.3626889 -17.0943744
H -19.5911057 26.7896238 -18.8053619
C -20.4418095 24.8784784 -18.2588574
H -20.2170614 24.0779836 -17.5490798
H -20.3816661 24.4589508 -19.2655484
C -21.873747 25.3850101 -18.0153021
O -22.0329252 26.4946208 -17.4568844
O -22.8044396 24.6367793 -18.3888393
N -17.5576422 23.6953514 -20.0649298
H -17.6282623 23.0479037 -19.293315
C -17.3269048 23.1256682 -21.396853
H -17.6295227 23.8524687 -22.1539443
C -18.173276 21.8705255 -21.6133325
O -18.4492201 21.1344351 -20.6665456
C -15.8303326 22.833808 -21.5971231
H -15.5031065 22.1074362 -20.8521036
H -15.2664698 23.755845 -21.4523805
C -15.5235885 22.3038595 -22.9769314
N -16.0573017 22.8083599 -24.1656877
C -15.6125519 22.0028993 -25.140073
H -15.854249 22.1181749 -26.1888167
N -14.8449621 21.02673 -24.6277346
H -14.429788 20.2755673 -25.1579691
C -14.7816493 21.1953241 -23.2621255
H -14.2791179 20.561661 -22.5447457
N -18.5185024 21.5818415 -22.8701808

H -18.1432063 22.1738025 -23.6009505
C -19.2288161 20.3661674 -23.257331
H -19.9116933 20.1067755 -22.4457962
C -18.2431347 19.2015468 -23.4437471
O -17.5034608 19.1367934 -24.4310812
C -20.0753688 20.6536823 -24.5101802
H -20.7622502 21.4721288 -24.2876809
H -19.4111275 20.9769363 -25.3134978
C -20.8899068 19.4416545 -25.0031907
H -20.2061397 18.632868 -25.252551
C -21.8900833 18.9305006 -23.9653095
H -22.5664824 19.7301243 -23.6638583
H -21.3716057 18.543429 -23.0898099
H -22.4721546 18.1090986 -24.387707
C -21.6558409 19.8178908 -26.2704486
H -22.3670983 20.6156827 -26.0549008
H -22.1966695 18.9460329 -26.6408658
H -20.9593117 20.149503 -27.0399621
N -18.305586 18.2264973 -22.5363312
H -18.9848484 18.3084584 -21.7879638
C -17.4987295 17.0022879 -22.56464
H -16.4445225 17.2767669 -22.5352472
C -17.7136686 16.1851165 -23.8479637
O -16.7324037 15.7061853 -24.4089253
C -17.8183622 16.1676056 -21.3118248
H -17.6392762 16.7679502 -20.4189096
H -17.1541192 15.3031549 -21.279748
C -19.2469521 15.6902349 -21.2993199
N -20.3555822 16.5291505 -21.1770985
C -21.409785 15.8002387 -21.5780685
H -22.404937 16.1941265 -21.7444035
N -21.0189659 14.5690479 -21.940586
H -21.5461555 13.999855 -22.611493
C -19.6585309 14.4792161 -21.7690455
H -19.0246639 13.6887216 -22.1457658
N -18.9342631 16.1416122 -24.3967885
H -19.7139306 16.4659898 -23.8383305
C -19.2306073 15.4622993 -25.6698397
H -18.9191364 14.4191048 -25.5943322
C -18.4740459 16.0915514 -26.8531044
O -17.994577 15.3816032 -27.732199
C -20.7422481 15.4827005 -25.9605596
H -20.9140981 15.0341016 -26.9402223
H -21.0845436 16.5175317 -26.0055241
C -21.5715667 14.7083567 -24.9337949
O -21.1731487 13.5796127 -24.57134

O -22.5721805 15.2767049 -24.4473625
N -18.2496141 17.4112082 -26.8323886
H -18.5716361 17.9329869 -26.0288096
C -17.4531297 18.1047217 -27.8489587
H -17.7977212 17.8135617 -28.8424323
H -17.5879439 19.1789559 -27.7304308
C -15.956921 17.7942072 -27.7454374
O -15.2888172 17.6280449 -28.7627028
N -15.439025 17.6201509 -26.5247796
H -16.0609943 17.7043393 -25.7294654
C -14.0743246 17.1266898 -26.3031017
H -13.3826248 17.7038566 -26.9186169
C -13.9256189 15.6534948 -26.724003
O -12.9564171 15.2990751 -27.3953963
C -13.702554 17.3422062 -24.8291262
H -13.7609572 18.4063683 -24.6038623
H -14.431762 16.8391893 -24.1953113
C -12.3186415 16.8515113 -24.4543145
C -11.189853 17.6482056 -24.7279804
H -11.3040053 18.609059 -25.2098121
C -9.9051194 17.1911998 -24.3777829
H -9.0312386 17.792005 -24.5808524
C -9.7460546 15.9368061 -23.7535044
O -8.5019852 15.5094553 -23.4137942
H -8.5099345 14.6569228 -22.9779248
C -10.8775819 15.1370117 -23.4808996
H -10.7593813 14.1747477 -23.0085044
C -12.161335 15.5959257 -23.8337612
H -13.0275541 14.9793858 -23.6353157
N -14.9268359 14.8201123 -26.423841
H -15.7014839 15.176822 -25.8741997
C -14.9828394 13.4197593 -26.8408227
H -14.0769896 12.9181517 -26.4986893
C -15.0310294 13.2711907 -28.3685764
O -14.316041 12.4302505 -28.9112801
C -16.1878629 12.7409781 -26.1724631
H -16.0987507 12.8425083 -25.0898935
H -17.1015123 13.2506904 -26.4752072
C -16.3194695 11.2716038 -26.5208461
C -15.679925 10.2960364 -25.7306851
H -15.1296517 10.5903453 -24.8487413
C -15.7427155 8.9385754 -26.1018896
H -15.2470437 8.1817352 -25.5143806
C -16.4327316 8.5582085 -27.2723703
O -16.4601685 7.2539103 -27.6505408
H -16.8895214 7.1591939 -28.5014005

C -17.087672 9.5346431 -28.0532165
H -17.603424 9.2540987 -28.9595907
C -17.035941 10.887789 -27.6716777
H -17.5222426 11.6367727 -28.2851494
N -15.7781968 14.1209507 -29.0785084
H -16.4213335 14.735133 -28.58639
C -15.8302485 14.0991621 -30.5458371
H -16.023302 13.0723458 -30.857743
C -14.4935853 14.5210283 -31.1785911
O -13.967663 13.81329 -32.0333803
C -16.994066 14.9750367 -31.0424011
H -17.90762 14.6865268 -30.519727
H -16.7819786 16.0183928 -30.8031416
C -17.238316 14.8508303 -32.5555673
H -16.3290961 15.1177248 -33.0992785
H -18.0038016 15.5779021 -32.8375267
C -17.6986865 13.4395358 -32.9540066
O -18.9264795 13.2539991 -33.097649
O -16.8225467 12.5548429 -33.0888592
N -13.837982 15.5729289 -30.6701081
H -14.2892367 16.1201696 -29.9440593
C -12.4898729 15.9648231 -31.1351682
H -12.5346458 16.1337827 -32.2113024
C -11.4694612 14.8360737 -30.9194148
O -10.6875397 14.5295424 -31.8238719
C -12.0510367 17.2817301 -30.4618222
H -12.2135606 17.2075124 -29.385962
C -10.5745282 17.6261432 -30.7001145
H -10.360449 17.6415137 -31.7693089
H -9.9326989 16.8899406 -30.2164525
H -10.3471552 18.6032195 -30.2737645
C -12.8768666 18.454303 -31.009272
H -12.6844 18.5843449 -32.0745506
H -12.6143381 19.3701809 -30.4802161
H -13.9403605 18.2682826 -30.8668087
N -11.5418689 14.1369177 -29.7810758
H -12.2068198 14.4377171 -29.0741423
C -10.7457172 12.934077 -29.5229422
H -9.6880299 13.1653715 -29.6509979
H -10.9128238 12.6231638 -28.4919327
C -11.1015855 11.7534031 -30.4368387
O -10.2052046 11.0525872 -30.9091058
N -12.3806552 11.5703557 -30.7665667
H -13.0669852 12.1981408 -30.3596135
C -12.8830576 10.4904933 -31.6335773
H -12.4536585 9.5465298 -31.299618

C -12.4546029 10.6930136 -33.0856704
O -11.8379689 9.7967565 -33.662114
C -14.4115139 10.3664975 -31.5108397
H -14.8837716 11.3195435 -31.7430037
C -15.0075931 9.281508 -32.405127
H -14.497612 8.3332624 -32.2385487
H -14.9184434 9.5764776 -33.4497101
H -16.0681325 9.1738969 -32.1753859
O -14.7191285 9.9990169 -30.1858573
H -14.6611214 10.8041515 -29.6445041
N -12.6061399 11.9050074 -33.6278135
H -13.1277568 12.5991368 -33.0943308
C -12.1108057 12.2899864 -34.96069
H -12.5626445 11.6330904 -35.7039491
C -10.5924574 12.1086371 -35.0606404
O -10.1118234 11.5017037 -36.0194828
C -12.5253388 13.7419652 -35.2846723
H -12.2047179 14.3950538 -34.4716908
C -11.9014053 14.2583629 -36.5896139
H -12.1485647 13.587198 -37.4129321
H -10.8182307 14.3273054 -36.4922998
H -12.2846337 15.2548982 -36.8119629
C -14.0473735 13.8569901 -35.4397281
H -14.390893 13.2481522 -36.2760117
H -14.3288191 14.8958381 -35.6094773
H -14.5551193 13.5168148 -34.5364626
N -9.8327613 12.5183483 -34.0376294
H -10.2691124 13.0158676 -33.2677802
C -8.3866883 12.2947826 -33.9964652
H -7.94803 12.713808 -34.9032965
C -8.0253687 10.7951613 -33.964585
O -7.1750255 10.3535353 -34.7385592
C -7.808095 13.0498299 -32.7940855
H -8.2322251 12.6640796 -31.8665257
H -8.0425363 14.1119177 -32.8768195
H -6.7249933 12.9258191 -32.7693519
N -8.7383063 9.9928143 -33.1690346
H -9.4399586 10.4216383 -32.5757625
C -8.5261026 8.5373236 -33.0519002
H -7.476906 8.3564513 -32.8190178
C -8.836892 7.7959867 -34.3557814
O -8.0721417 6.9214099 -34.7582541
C -9.3671494 7.960609 -31.9009351
H -10.4270834 8.1175921 -32.1029293
C -9.1210937 6.4714297 -31.6602582
H -8.0569718 6.2924769 -31.5037895

H -9.4663804 5.8883818 -32.5134126
H -9.6722286 6.1524028 -30.7752524
O -9.0287859 8.6085484 -30.6970051
H -9.4300372 9.4946216 -30.711502
N -9.8765834 8.2071453 -35.0894348
H -10.4751493 8.9342993 -34.7066042
C -10.218649 7.6484065 -36.4087586
H -10.3078933 6.565189 -36.3149444
C -9.0961985 7.9031619 -37.4291875
O -8.8107229 7.0319327 -38.2457573
C -11.5883493 8.2058925 -36.8724934
H -11.5800876 9.2895272 -36.7418409
C -11.8649231 7.9080697 -38.3605028
H -11.8182208 6.8336763 -38.5433383
H -11.1330206 8.4106419 -38.9928817
H -12.8465962 8.2794081 -38.6492608
C -12.7194695 7.6055139 -36.002287
H -12.8725628 6.5606242 -36.275951
H -12.4291906 7.6247014 -34.9527788
C -14.0547499 8.3529914 -36.1158443
H -14.469557 8.2558855 -37.1180749
H -13.9111854 9.4081356 -35.8800284
H -14.765007 7.9268607 -35.4065953
N -8.3937191 9.0388911 -37.3367375
H -8.6846259 9.7176446 -36.6427142
C -7.265239 9.3812153 -38.2229573
H -7.5533014 9.1179589 -39.2408252
C -5.9497281 8.6286735 -37.9283472
O -4.9741865 8.7465544 -38.6840646
C -7.0390962 10.899473 -38.1899562
H -8.0014138 11.4136505 -38.2093944
H -6.4800146 11.1924024 -39.0771632
O -6.2990547 11.3175345 -37.05805
H -6.6658562 10.914714 -36.2473093
N -5.8900761 7.8921141 -36.8100548
H -6.7249579 7.8243468 -36.2397125
C -4.6565738 7.3302429 -36.256341
H -3.8362244 8.0007177 -36.5123651
C -4.2800623 5.9408626 -36.8074929
O -3.1352983 5.5205919 -36.6491747
C -4.7709993 7.3240925 -34.7269169
H -5.5236596 6.6026775 -34.4107831
H -5.0474193 8.3167635 -34.3677705
H -3.8122191 7.0434425 -34.2890576
N -5.1800992 5.2659515 -37.5310566
H -6.0659794 5.7270073 -37.7010793

C -4.9863126 3.9392569 -38.1512939
H -5.0038412 3.1815968 -37.3671395
C -3.6467069 3.7571432 -38.8886477
O -2.9731921 2.7386014 -38.7264792
C -6.1697208 3.6740353 -39.1048049
H -7.0402153 3.4328757 -38.4935022
H -5.9499646 2.7970377 -39.7150907
C -6.5637775 4.8197352 -40.0201111
N -5.7699764 5.9207787 -40.3641479
C -6.5674691 6.752385 -41.049963
H -6.277414 7.7318812 -41.4028678
N -7.7926363 6.2244398 -41.1826337
H -8.5854946 6.6964794 -41.5940588
C -7.8129371 5.0107498 -40.5332027
H -8.6720512 4.3747877 -40.3736965
N -3.1845526 4.7824287 -39.6094995
H -3.8397485 5.5490973 -39.7402001
C -1.9094941 4.7790629 -40.3503428
H -1.9058746 3.9210138 -41.0259016
C -0.6755178 4.6000095 -39.4590681
O 0.3500762 4.1523355 -39.9634635
C -1.8108003 6.0655976 -41.1818839
H -1.9802055 6.9149351 -40.5182176
H -0.8101594 6.155225 -41.6088882
C -2.8366963 6.0737128 -42.3295833
H -3.7805566 5.6467642 -41.9998432
H -2.4675716 5.4691415 -43.158521
C -3.090666 7.5062857 -42.7975055
H -2.21557 7.8834898 -43.328123
H -3.2604295 8.1357064 -41.9227019
N -4.2898586 7.6146451 -43.6353146
H -5.0985667 8.0752973 -43.2163844
C -4.3632322 7.5927761 -44.9465972
N -3.4118636 7.0991039 -45.680707
H -3.2172373 7.5904414 -46.5573116
H -2.6560279 6.644648 -45.2205878
N -5.4028833 8.1065841 -45.5234574
H -6.0523492 8.6180683 -44.9202338
H -5.4423454 8.1830156 -46.5185052
N -0.7546542 4.8641941 -38.1528525
H -1.6478001 5.1514024 -37.7665882
C 0.3441051 4.6164829 -37.2169392
H 1.2323201 5.1264964 -37.5885598
C 0.6996833 3.1210416 -37.0906775
O 1.8637562 2.7984179 -36.8587498
C -0.0133202 5.2297426 -35.858726

H -0.9017486 4.748926 -35.4486878
H -0.2065543 6.2971976 -35.9704315
H 0.8169899 5.0909097 -35.1647239
N -0.2423564 2.2117153 -37.3765593
H -1.1831707 2.5200405 -37.5942453
C 0.0441412 0.7786215 -37.4595675
H 0.5673747 0.4724402 -36.5515695
C 0.9552045 0.4341165 -38.6550826
O 1.7906982 -0.4590169 -38.5465271
C -1.2872208 0.0221727 -37.522223
H -1.8167315 0.2672544 -38.4447155
H -1.9079066 0.2944651 -36.668599
H -1.1024213 -1.0504495 -37.4916568
N 0.8781965 1.1977947 -39.7544587
H 0.2102942 1.9555281 -39.7611379
C 1.8112123 1.0873253 -40.8905783
H 2.0039892 0.0301488 -41.0804667
C 3.1532747 1.7347408 -40.5479506
O 4.1918602 1.1211358 -40.7786454
C 1.2242149 1.7004207 -42.1796402
H 1.0712918 2.7691655 -42.0362597
C 2.1680245 1.5163423 -43.3761676
H 2.3959256 0.458749 -43.5151264
H 3.0968456 2.0616945 -43.2111974
H 1.7047064 1.9050683 -44.2829471
C -0.1270984 1.0684292 -42.5415657
H -0.0134202 -0.0094525 -42.6618157
H -0.4995357 1.4948188 -43.4727939
H -0.8607535 1.2658962 -41.7604837
N 3.1497115 2.9075864 -39.8983128
H 2.2584563 3.3646896 -39.7477886
C 4.3829764 3.5769137 -39.4271644
H 5.0316617 3.7532182 -40.287047
C 5.1714679 2.6870662 -38.4572727
O 6.3914124 2.6466122 -38.5342007
C 4.0854947 4.9447377 -38.7734971
H 3.4492565 4.7868126 -37.9038288
C 5.3579813 5.667574 -38.3077099
H 6.0473034 5.7700222 -39.1479344
H 5.8520541 5.0988091 -37.5198768
H 5.1091859 6.6515776 -37.9116408
C 3.3657123 5.9094878 -39.7292705
H 4.0142422 6.146524 -40.5715909
H 3.1117469 6.8290265 -39.2030367
H 2.4524687 5.4645623 -40.1099666
N 4.5099699 1.8691594 -37.6261136

H 3.501018 1.9661057 -37.5679173
C 5.1768327 0.8861925 -36.7517641
H 5.9189905 1.4157616 -36.1524491
C 5.9655105 -0.1805465 -37.5278367
O 6.9655235 -0.6580613 -37.005066
C 4.1151826 0.2654933 -35.821984
H 3.6872668 1.0584642 -35.2070059
H 3.3115988 -0.1409395 -36.4366679
C 4.6026419 -0.8663175 -34.8987054
H 4.9701494 -1.6946528 -35.5041773
H 3.7425868 -1.2253198 -34.3369632
C 5.691348 -0.4487287 -33.8979481
H 5.305648 0.3329929 -33.2411631
H 6.5347315 -0.0299299 -34.4515579
N 6.1852786 -1.5988657 -33.1065998
H 7.0302672 -2.023504 -33.4565587
C 5.6931212 -2.0929878 -31.9796119
N 4.6137617 -1.6238221 -31.4121932
H 4.2496792 -2.0315972 -30.5644291
H 4.1500759 -0.8388203 -31.8268032
N 6.2826626 -3.0960025 -31.392016
H 7.158683 -3.4460252 -31.7437805
H 5.9220268 -3.4384356 -30.5210906
N 5.5130667 -0.5786026 -38.7196586
H 4.6989518 -0.1098716 -39.0946508
C 6.155844 -1.6215128 -39.5477068
H 6.7171868 -2.2989085 -38.9044692
C 7.1870424 -1.0471198 -40.5141252
O 8.1672071 -1.7115576 -40.8379074
C 5.0729423 -2.429844 -40.2970744
H 4.4719049 -1.7392024 -40.8919679
C 5.6505611 -3.4778044 -41.2668926
H 6.3652394 -4.1194898 -40.7503349
H 6.1577817 -2.9777053 -42.0922651
H 4.8537515 -4.0898952 -41.6888069
C 4.1272498 -3.1381766 -39.3001663
H 3.4521743 -2.3981917 -38.8745679
H 3.519856 -3.8567523 -39.8472324
C 4.8022508 -3.8790266 -38.1377464
H 5.1620025 -3.1674568 -37.3956499
H 5.6444629 -4.4611102 -38.5038849
H 4.0797422 -4.5405924 -37.6627072
N 6.9634562 0.1827065 -40.9660073
H 6.1059518 0.645179 -40.6903566
C 7.8918005 0.9019409 -41.8307362
H 8.3560989 0.1968688 -42.5199967

C 9.0541694 1.5083209 -41.0392823
O 10.1751688 1.4175308 -41.5113396
C 7.0910835 1.9372281 -42.6425295
H 6.4755174 2.5141194 -41.9494156
C 8.015055 2.9405889 -43.3353853
H 8.8170607 2.4130698 -43.8517664
H 8.4436482 3.6079035 -42.5859543
H 7.4537262 3.5413332 -44.0376991
C 6.1329575 1.2547909 -43.6511673
H 5.3388 0.7519175 -43.1009127
H 5.6559528 2.0240523 -44.2518133
C 6.7607953 0.2206656 -44.5975052
H 7.0017951 -0.6915304 -44.0477814
H 7.6692296 0.6123557 -45.0487245
H 6.0481605 -0.0291608 -45.3837918
N 8.798992 1.9819869 -39.8195457
H 7.8269675 1.9509433 -39.5448266
C 9.6149082 2.8267209 -38.9312898
H 9.2170332 2.5797365 -37.9459199
C 9.3250434 4.3409519 -39.0306445
O 8.5238253 4.7993794 -39.8542185
C 11.099113 2.4020543 -38.8279642
H 11.7082873 3.1408157 -39.3434734
H 11.2510327 1.4269978 -39.2904088
C 11.5965131 2.3038259 -37.3833269
O 11.4492724 3.3205405 -36.6703128
O 12.1249463 1.2330349 -37.0094698
N 9.8588958 5.1058401 -38.0754761
H 10.516748 4.6466534 -37.4396315
C 9.3325992 6.4041998 -37.6512809
H 8.3526531 6.2042132 -37.2199303
C 9.1395831 7.4234129 -38.7896545
O 10.0494829 7.7492656 -39.5515223
C 10.2024572 6.9760756 -36.5200309
H 10.3373002 6.2059069 -35.7593743
H 11.1814432 7.2466722 -36.9189787
C 9.5601829 8.2096529 -35.8657287
H 8.5933201 7.9247972 -35.443284
H 9.3792816 8.9717041 -36.6261681
C 10.4478246 8.8008571 -34.7647909
O 10.8020372 8.0456266 -33.8314376
O 10.7428137 10.012847 -34.8410345
N 7.9356494 8.0039627 -38.8202964
H 7.2747294 7.6949909 -38.1276181
C 7.4979327 9.066309 -39.7246913
H 6.4475177 9.2219085 -39.4784392

C 7.4607978 8.7250306 -41.2328113
O 7.2182063 9.6195186 -42.0501456
C 8.189382 10.3821855 -39.339387
H 9.2533861 10.3291555 -39.5703739
H 8.0772996 10.5585665 -38.2684137
H 7.7299772 11.2135753 -39.8729389
N 7.5456185 7.4423073 -41.6155564
H 7.7541 6.7354562 -40.9135248
C 7.344726 6.9801426 -42.997353
H 6.9802142 7.8289962 -43.5714683
C 6.2228111 5.9398072 -43.1423331
O 5.8136015 5.298277 -42.1811172
C 8.7040589 6.5417867 -43.5937623
H 9.3769214 6.2789443 -42.781539
H 8.5857406 5.6690598 -44.2357264
C 9.2911549 7.7100855 -44.425368
H 9.1907816 8.6410518 -43.8675988
C 10.7676023 7.5379856 -44.7641775
H 10.9094523 6.6899523 -45.4326336
H 11.347273 7.4053009 -43.8535032
H 11.1408564 8.4293545 -45.2677901
C 8.5546316 7.8484516 -45.7653518
H 8.6226347 6.9085588 -46.3141622
H 9.0244539 8.6267692 -46.3653165
H 7.5122898 8.121164 -45.6183874
N 5.6747873 5.8134553 -44.3622881
H 6.051826 6.3868802 -45.1032308
C 4.5891328 4.8649351 -44.6980872
H 4.5631062 4.1139725 -43.9051263
C 4.8447935 4.0459821 -45.973334
O 3.9017478 3.6498304 -46.6520897
C 3.2105171 5.5603428 -44.666108
H 2.4533252 4.7738203 -44.6831793
H 3.1054279 6.0864467 -43.7153432
C 2.9161759 6.5454592 -45.8179066
H 3.1261552 6.0697121 -46.7735315
H 3.5448542 7.4315362 -45.7241825
C 1.4308664 6.9484155 -45.7776237
H 1.2590212 7.5693356 -44.8966521
H 0.8212512 6.0482975 -45.6740764
N 0.981203 7.6811794 -46.9780385
H 0.7524881 8.6673509 -46.8923419
C 0.6228584 7.177358 -48.1443321
N 0.8186153 5.929421 -48.4685675
H 0.5752193 5.6132175 -49.3870961
H 1.388143 5.3577715 -47.8677134

N 0.0406873 7.949872 -49.0092621
H -0.2933387 8.855375 -48.6610897
H -0.2623347 7.6163466 -49.9005672
N 6.1190758 3.8578277 -46.3209588
H 6.8301691 4.1363819 -45.6635574
C 6.5958218 3.2428633 -47.5626859
H 5.830005 2.5719029 -47.9542416
C 7.8916732 2.4367347 -47.343168
O 8.5668267 2.64766 -46.3401455
C 6.8674228 4.3491416 -48.5851797
H 7.7970464 4.8464069 -48.304794
H 7.032437 3.8770175 -49.5533758
C 5.793543 5.4093771 -48.7466088
C 4.5694425 5.071977 -49.3430479
H 4.3617759 4.0434651 -49.5976387
C 3.6563468 6.0786253 -49.6922932
H 2.7837729 5.8310304 -50.2772817
C 3.9157208 7.4151039 -49.3467661
H 3.2349086 8.196869 -49.6514519
C 5.0976506 7.7468283 -48.6644446
H 5.3156042 8.7854837 -48.4512446
C 6.0470079 6.7471097 -48.3865945
H 6.9997683 7.0245668 -47.966154
N 8.2414517 1.5676536 -48.3001291
H 7.6597218 1.5224626 -49.118932
C 9.3979198 0.6578697 -48.2914649
H 9.1016447 -0.2646165 -47.791358
C 10.6602547 1.1969945 -47.5742744
O 11.1786285 2.245083 -47.9427162
C 9.7087369 0.3224341 -49.75895
H 9.967396 1.234773 -50.3010873
H 8.8483582 -0.1504811 -50.2328802
H 10.5608699 -0.3531544 -49.8204047
N 11.1058984 0.4683517 -46.5404187
H 10.6020477 -0.3756197 -46.3181145
C 12.3498292 0.6411544 -45.7620579
H 12.0842348 0.2321049 -44.78769
C 12.7677959 2.0582833 -45.3295639
O 13.9257997 2.2966503 -44.965395
C 13.488643 -0.2542563 -46.2966031
H 14.4106292 0.3125754 -46.3885813
H 13.233996 -0.5667301 -47.3062372
C 13.7404825 -1.5012609 -45.4212878
O 12.782663 -1.9446545 -44.7479203
O 14.8558588 -2.0616043 -45.4835954
N 11.7676098 2.9221305 -45.1175289

H 10.8663841 2.6705479 -45.5032949
C 11.8434355 4.1137479 -44.2565654
H 10.9702733 4.7131008 -44.4953178
C 13.0474841 5.0013958 -44.5690478
O 13.2566867 5.4146228 -45.7033659
C 11.6839069 3.7215239 -42.7654516
H 10.7042933 3.2772 -42.7433744
C 12.7172875 2.6995277 -42.243958
H 13.7411237 3.021697 -42.4103113
H 12.5627012 1.7344082 -42.7242798
H 12.5786212 2.5252549 -41.1832717
C 11.6078686 4.8907969 -41.7666777
H 12.5088229 4.963613 -41.1569353
H 10.7748965 4.7152793 -41.0967139
H 11.4403098 5.837781 -42.2673589
N 13.7997814 5.3551081 -43.5357305
H 13.5427582 4.9757027 -42.6392564
C 14.9722896 6.1999847 -43.5773004
H 14.7513394 7.1316738 -44.1038787
C 16.1325397 5.5277022 -44.3124657
O 16.971215 6.2121722 -44.8791594
C 15.3363167 6.5059358 -42.1167684
H 14.5993989 7.1982355 -41.7073855
H 15.2826306 5.5845827 -41.5328418
C 16.7149966 7.0988042 -41.9408267
C 16.9780411 8.3984896 -42.403626
H 16.1908624 8.9809401 -42.8654804
C 17.7536204 6.3218351 -41.3924162
H 17.5600729 5.3123153 -41.0611771
C 18.2759221 8.9240865 -42.299878
H 18.4802673 9.9218835 -42.6403416
C 19.0546992 6.8452967 -41.3078177
H 19.8555147 6.2433117 -40.90308
C 19.314826 8.149128 -41.7601691
H 20.3159865 8.5514257 -41.6963891
N 16.2058513 4.196207 -44.3268838
H 15.3924863 3.6449569 -44.0809636
C 17.3365519 3.5317345 -44.9880782
H 18.247824 4.0148928 -44.6328091
C 17.3362498 3.7162578 -46.5202865
O 18.411362 3.6889561 -47.1212283
C 17.4139431 2.0551039 -44.544089
H 17.2640812 2.0186564 -43.4640312
C 18.7685605 1.4097662 -44.8475106
H 18.7772956 0.3945679 -44.4540022
H 19.5699411 1.9849547 -44.3903993

H 18.9200973 1.3641078 -45.9264033
O 16.4573901 1.2286298 -45.1599023
H 15.5577683 1.5969757 -45.0914068
N 16.1818698 4.0690221 -47.1078517
H 15.3465728 4.1192239 -46.5422669
C 16.0387294 4.3659216 -48.539581
H 16.9320402 3.9392428 -48.9945931
C 16.0907108 5.8966401 -49.0803814
O 16.0794458 5.9767522 -50.2908172
C 14.8556147 3.4957892 -49.0773819
H 13.9798023 4.1366509 -49.1933825
H 14.5968292 2.7346661 -48.3378472
C 15.1275989 2.7525254 -50.4024108
O 14.1400315 2.2286328 -50.965854
O 16.307982 2.6299838 -50.8096799
N 16.2900343 7.1238414 -48.4203981
H 16.4497442 7.0131143 -47.4277553
C 16.1613041 8.688626 -48.8951202
H 15.5643498 8.6270493 -49.8051702
C 17.4543756 9.8556596 -49.3389581
O 18.4787587 9.2741032 -49.0634247
C 15.1839224 9.2297703 -47.7945255
H 14.6070166 8.4267525 -47.3302822
H 14.4664933 9.8869038 -48.2885934
C 15.9020718 10.0680588 -46.713811
O 16.3872989 11.1886415 -47.009515
O 15.9809798 9.6471257 -45.537687
N 17.6582507 11.2710311 -50.0187799
H 16.7432841 11.6473397 -50.215925
C 18.9196021 12.4999217 -50.4996121
H 19.4239745 12.7072473 -49.557024
C 19.8910586 11.6284874 -51.3214552
H 19.3040143 10.926469 -51.916006
H 20.4598406 12.2416738 -52.0211191
C 20.8996192 10.8577388 -50.4511839
H 20.4908512 10.6758339 -49.4595413
H 21.799341 11.4600084 -50.3269145
C 21.2793501 9.5187183 -51.0747255
O 22.2065281 9.4215954 -51.8603748
N 20.5592735 8.459898 -50.7803827
H 20.794535 7.5910496 -51.2218482
H 19.7724068 8.5434667 -50.1355936
C 19.092366 14.182846 -51.3913657
O 18.0971829 14.350783 -52.0856275
N 20.1085432 15.3856543 -51.5493382
H 20.9260687 15.212857 -50.986138

C 20.2948739 16.9461427 -52.3131094
H 20.2227503 16.7196393 -53.3770916
C 21.4078328 18.3179152 -52.2406162
O 22.5468109 18.183726 -51.7988277
C 19.0686253 17.7464106 -51.9697146
H 19.2338989 18.2728335 -51.0391949
H 18.2005998 17.0932304 -51.9204808
H 18.8667864 18.4802587 -52.7438545
N 21.1702238 19.6749261 -52.6514902
H 20.2345851 19.8379585 -52.9935248
C 22.0024176 21.0115075 -52.4479166
H 22.8683875 20.7431257 -51.8441611
C 21.2832516 22.1579803 -51.6588977
O 20.098186 22.3880363 -51.8856444
C 22.5113401 21.7661131 -53.7349204
H 21.6390627 22.0773934 -54.3111731
H 23.0283835 22.6774737 -53.4283394
C 23.4507222 21.0203275 -54.6889031
H 22.9115143 20.1707727 -55.1106251
H 23.7236569 21.6915994 -55.5045992
C 24.7266258 20.5101083 -54.0066815
H 24.4530278 19.8220364 -53.2042071
H 25.2791937 21.3544152 -53.5879837
C 25.5984771 19.7681775 -55.0268131
H 25.909443 20.4728439 -55.8051529
H 24.9888066 18.9920389 -55.5006992
N 26.7854235 19.1515691 -54.3842775
H 27.3653918 19.8580064 -53.9512649
H 27.3408378 18.6543916 -55.0695705
H 26.4940335 18.4923601 -53.6723818
N 22.0141776 22.9790716 -50.8776924
H 22.9848334 22.7561847 -50.7481297
C 21.4363151 24.0595014 -50.0406279
H 20.6651534 23.5880877 -49.4217957
C 20.6932301 25.1571828 -50.8434591
O 21.1123859 25.4928957 -51.9501055
C 22.4954675 24.6021902 -49.0576117
H 21.9681595 25.0697176 -48.2281703
H 23.0446883 23.7611733 -48.6298199
C 23.4967813 25.6136439 -49.6395768
H 24.1308012 25.1185841 -50.3761876
H 22.9616084 26.426131 -50.1294037
C 24.3600993 26.2058559 -48.5122502
H 25.0695733 25.4548231 -48.159759
H 23.7129745 26.4715583 -47.6719088
C 25.1007745 27.47991 -48.9414417

H 25.6487684 27.8643149 -48.0751497
H 24.3538345 28.231996 -49.2207631
N 26.033051 27.2429212 -50.0717632
H 26.729265 26.5557402 -49.8134378
H 26.5058074 28.1019389 -50.3270792
H 25.5216444 26.9054137 -50.8778729
N 19.6653497 25.7902966 -50.2502701
C 18.5260935 26.4199977 -50.9302971
H 17.683172 26.2611953 -50.25573
C 17.9666354 25.9230463 -52.2825018
O 17.2120042 26.6858309 -52.8855268
C 18.8245867 27.9163968 -50.8994079
H 19.6064095 28.1600803 -51.6210341
H 17.9311673 28.5198536 -51.0658878
C 19.9982801 26.7186724 -49.1631419
H 19.6497361 26.3469128 -48.204326
H 21.0706298 26.8855675 -49.103357
C 19.3411503 28.0678737 -49.4726293
H 18.5032263 28.2293829 -48.7937525
H 20.0551531 28.8885143 -49.395854
N 18.1444302 24.6585658 -52.7170806
H 18.7575415 24.0313991 -52.2081714
C 17.2497154 24.0944877 -53.7603078
H 17.1809009 24.8305569 -54.5644565
C 15.8108259 23.9536811 -53.2184155
O 15.6116559 23.7733122 -52.0168994
C 17.7905208 22.7718561 -54.367871
H 18.2164828 22.1394345 -53.5915213
H 16.9504542 22.2274091 -54.799801
C 18.8275157 22.9966047 -55.490398
H 19.7490875 23.3914824 -55.0618205
H 18.4292468 23.7618966 -56.1540239
C 19.1471149 21.7322105 -56.331149
H 18.3107319 21.0347568 -56.2604309
H 20.0375569 21.2460875 -55.9308658
N 19.3493069 22.0762304 -57.7577649
H 19.2235844 23.0639289 -58.0004847
C 19.3902273 21.3013719 -58.8329725
N 19.5688706 20.0105413 -58.789513
H 19.5868354 19.4947518 -59.6476506
H 19.6328429 19.5190873 -57.9186205
N 19.2411043 21.8219077 -60.01574
H 18.7989704 22.7503794 -60.07748
H 19.2658129 21.2691169 -60.8598066
N 14.821947 23.9635607 -54.1171727
H 15.0640462 24.1295175 -55.0795782

C 13.4070457 23.7053603 -53.8023649
H 13.2318405 23.8314889 -52.7303655
C 13.0148305 22.2690653 -54.1901151
O 13.2650034 21.8554559 -55.3268438
C 12.5042714 24.70102 -54.554355
H 11.4657565 24.4018961 -54.4176821
H 12.7363243 24.6603459 -55.6166903
C 12.5918951 26.14774 -54.1111584
O 13.1869008 26.5106661 -53.1113018
N 11.9857542 27.0281791 -54.8750885
H 12.0663734 27.990201 -54.6088372
H 11.474667 26.7304553 -55.7012412
N 12.3104354 21.5618859 -53.3039725
H 12.1319232 21.9882772 -52.3959269
C 11.7798987 20.2088839 -53.5218785
H 12.1354009 19.8236533 -54.4750295
C 10.2499869 20.2006078 -53.5304971
O 9.636071 20.6928764 -52.5837385
C 12.2939867 19.2814334 -52.4104324
H 12.0003572 19.7111831 -51.4550013
H 13.3811827 19.2399207 -52.459261
C 11.7331357 17.8531895 -52.451972
H 10.6473428 17.8812916 -52.5280063
H 11.9562163 17.3702826 -51.5054196
S 12.3827862 16.813984 -53.7779941
C 13.8931229 16.2105975 -52.9728887
H 14.4627188 15.602152 -53.6752431
H 13.6285852 15.6018071 -52.1075691
H 14.5036321 17.0516099 -52.6454799
N 9.6561252 19.476022 -54.4810838
H 10.2356146 19.1014098 -55.228018
C 8.2618632 19.0294284 -54.4110718
H 7.7432147 19.5950109 -53.6411374
C 8.2012683 17.5367186 -54.0391612
O 8.6091906 16.6916482 -54.8315145
C 7.5848191 19.3377223 -55.7563497
H 7.6533027 20.404727 -55.9472126
H 8.1407942 18.8235216 -56.5354205
C 6.1094319 18.9161937 -55.8471269
H 6.0223168 17.8621598 -55.5987058
C 5.2008171 19.7105303 -54.9111302
H 5.2842959 20.7773402 -55.1160026
H 5.4789999 19.5191252 -53.8783082
H 4.1706631 19.3882301 -55.0433692
C 5.5958432 19.1157678 -57.2735893
H 5.6369121 20.1715894 -57.5396105

H 4.5696247 18.7588489 -57.3505647
H 6.2159663 18.5490014 -57.9673902
N 7.650806 17.207511 -52.8705797
H 7.2809473 17.9542867 -52.2913677
C 7.3710433 15.8279812 -52.4464654
H 8.0060151 15.1508264 -53.0126345
C 5.9027388 15.4793458 -52.7611271
O 4.9709185 16.075372 -52.2065813
C 7.75986 15.6405981 -50.9667627
H 7.1203425 16.2573012 -50.3370099
H 8.7862314 15.9905422 -50.8370111
C 7.6824443 14.1863395 -50.4760692
O 6.8708591 13.4090086 -51.019936
O 8.4087115 13.8653462 -49.5055914
N 5.6864177 14.5711102 -53.7189983
H 6.4974395 14.0713565 -54.0777662
C 4.3447916 14.1842873 -54.1843736
H 3.6345714 14.9349283 -53.8406842
C 3.9351687 12.8544228 -53.5605881
O 4.3232699 11.7831128 -54.0192536
C 4.2336867 14.1575708 -55.7201635
H 4.8568936 13.3737071 -56.1278179
C 2.7974381 13.8629456 -56.1479142
H 2.1426664 14.6095088 -55.703318
H 2.4969621 12.8683791 -55.8186253
H 2.7229068 13.8922377 -57.2343704
C 4.6461988 15.4961607 -56.3443168
H 4.0765308 16.3124174 -55.897042
H 4.4772019 15.482627 -57.4198195
H 5.7094427 15.6583488 -56.1699027
N 3.0257182 12.9205146 -52.5909151
H 2.717312 13.8404901 -52.2952782
C 2.6511322 11.7941466 -51.7378099
H 1.5919382 11.8736081 -51.495824
H 2.826334 10.8515972 -52.2505211
C 3.4378837 11.764253 -50.4292475
O 3.7783718 10.6859284 -49.9559292
N 3.6725557 12.9262538 -49.8125931
H 3.3218807 13.7634739 -50.2589932
C 4.5639542 13.1282033 -48.6574106
H 5.5763021 12.8435635 -48.9560907
C 4.2394702 12.317265 -47.379758
O 4.9944892 12.3378076 -46.4012804
C 4.557135 14.6320349 -48.3562011
H 4.9178299 15.1702423 -49.2356233
H 5.2327209 14.8285762 -47.5267004

S 2.8894722 15.2074225 -47.9074698
H 2.7061254 14.4032963 -46.8479299
N 3.0995178 11.6227276 -47.3420058
H 2.5537956 11.6005599 -48.1895828
C 2.6973692 10.7695608 -46.2284903
H 1.7296098 10.3306215 -46.4382888
H 3.4252333 9.964224 -46.1301078
C 2.5964225 11.5335775 -44.9096616
O 2.0756425 12.6458024 -44.8617237
N 3.1475683 10.9405853 -43.8409104
H 3.5995258 10.050913 -43.9813798
C 3.2644505 11.5676352 -42.5110625
H 2.5363332 12.3721322 -42.4922009
C 4.6024855 12.3215117 -42.2989558
O 4.9330438 12.7035656 -41.1837733
C 2.7685976 10.6219858 -41.3876643
H 3.329892 9.6866489 -41.3966698
H 2.9110664 11.1133101 -40.4234859
C 1.2482459 10.3497499 -41.5881163
H 0.7919812 11.3134804 -41.7977461
H 1.1127473 9.7337692 -42.4784474
C 0.4227669 9.7161131 -40.4391496
H 0.7314226 10.1512193 -39.4901884
H 0.5960085 8.639396 -40.4025918
C -1.0758004 10.0217816 -40.7095582
H -1.41602 9.4220592 -41.5561837
H -1.1388727 11.0716698 -41.0127608
N -2.0107233 9.8625097 -39.5598994
H -2.0996365 8.9127489 -39.2334567
H -2.9406933 10.1949484 -39.8135329
H -1.7309402 10.4529809 -38.7839952
N 5.3030928 12.6456133 -43.3932679
H 4.9509657 12.3158047 -44.2839729
C 6.263606 13.7539353 -43.4685514
H 6.339384 14.0373157 -44.5168193
H 5.8664254 14.6025745 -42.9108326
C 7.6966603 13.5217271 -42.9844836
O 8.5229441 14.4189115 -43.1446022
N 8.052298 12.3272621 -42.5032093
H 7.3432049 11.6119669 -42.3818921
C 9.4128024 12.0200965 -42.040962
H 9.6646061 12.6654394 -41.1981862
H 9.4474178 10.9834761 -41.7123813
C 10.481707 12.1834795 -43.1192423
O 11.5222019 12.7773601 -42.8556136
N 10.1750316 11.7843984 -44.3589289

H 9.2846575 11.3332788 -44.4955374
C 11.0186339 12.0413754 -45.5425943
H 11.9980506 11.588156 -45.3790969
C 11.2701527 13.5389522 -45.7486745
O 12.4236188 13.9518113 -45.8260238
C 10.4258214 11.3832015 -46.8083101
H 10.5905208 10.3099959 -46.7269139
C 8.9312833 11.6033541 -47.0463739
H 8.7099762 12.6632397 -47.1442538
H 8.3393666 11.1827496 -46.2344072
H 8.6361123 11.1131843 -47.9758533
O 11.0895781 11.8306891 -47.9597574
H 10.484578 12.3844576 -48.4701324
N 10.2252136 14.371186 -45.663101
H 9.3074535 13.9722939 -45.5446904
C 10.2857366 15.8236971 -45.8547703
H 10.7235008 16.0265557 -46.8268567
C 11.1106349 16.537419 -44.7650356
O 11.8863183 17.4526382 -45.0535196
C 8.8657689 16.3970441 -45.8856242
H 8.8986116 17.4030429 -46.3069571
H 8.4867539 16.4648006 -44.8668808
S 7.7163401 15.3759861 -46.8510702
H 8.4533831 15.1086649 -47.9512639
N 10.9632892 16.0999518 -43.508704
H 10.2825801 15.3646959 -43.3383485
C 11.7666912 16.5933009 -42.3777083
H 11.7210304 17.682167 -42.3558897
C 13.2337723 16.2149077 -42.5646649
O 14.1041332 17.0794444 -42.4631432
C 11.2181531 16.0596538 -41.0385742
H 11.0788588 14.9803609 -41.1088303
C 12.1583111 16.3454275 -39.8608403
H 12.413162 17.4044263 -39.8365978
H 13.0713173 15.7583224 -39.9544092
H 11.6747709 16.0658247 -38.9240883
C 9.8619149 16.7101419 -40.7311641
H 9.9738266 17.7906836 -40.6408162
H 9.465485 16.313601 -39.7953368
H 9.147258 16.4904463 -41.5236473
N 13.5086787 14.9554922 -42.9189931
H 12.7374487 14.300883 -43.0121126
C 14.8616324 14.4600437 -43.1763215
H 15.4605874 14.5795315 -42.2714439
C 15.5717593 15.257738 -44.2626702
O 16.6984701 15.6980688 -44.0520232

C 14.8119003 12.9696725 -43.5454865
H 15.47017 12.778969 -44.3885111
H 13.8159203 12.6800994 -43.8734669
C 15.2350372 12.1081566 -42.3887418
N 16.5503927 11.9810507 -41.9354251
C 16.4753185 11.2196844 -40.8299497
H 17.3253826 10.9192111 -40.2306378
N 15.2029599 10.8627996 -40.5836198
H 14.890693 10.2906702 -39.8085624
C 14.4037633 11.4233475 -41.5538219
H 13.3257702 11.3738953 -41.6274226
N 14.9078435 15.5075468 -45.387301
H 13.989292 15.091668 -45.5152898
C 15.5112271 16.2350314 -46.5011762
H 16.4944499 15.8005517 -46.6810838
C 15.788866 17.702243 -46.1455768
O 16.9395239 18.1374501 -46.2012692
C 14.6897345 16.0179734 -47.7882799
H 15.1327128 16.6236197 -48.5603665
C 14.7908711 14.5234165 -48.182398
H 14.1962195 13.8987777 -47.5198436
H 15.8220997 14.1781548 -48.1262874
H 14.4372692 14.3716629 -49.2011021
C 13.2366266 16.4975189 -47.7320752
H 12.7646062 16.0195718 -46.8976035
H 13.2059957 17.5711979 -47.5585925
C 12.4407379 16.1837724 -49.0033466
H 12.2244151 15.1164961 -49.062049
H 13.0189562 16.48687 -49.8741816
H 11.4970485 16.7230964 -48.9888032
N 14.8187234 18.4245126 -45.5793613
H 13.9008259 18.0120409 -45.4473034
C 15.0314858 19.8172963 -45.1857533
H 15.4248899 20.3646793 -46.0422485
C 16.0654803 19.9815299 -44.052507
O 16.775709 20.9872676 -44.0197683
C 13.6739593 20.4215669 -44.8288207
H 13.2487701 19.8884107 -43.9775321
H 12.9997992 20.3346726 -45.682005
H 13.7950387 21.4777533 -44.588073
N 16.1945128 19.0031707 -43.1417661
H 15.5550477 18.2125874 -43.1960906
C 17.1426889 19.0652184 -42.0165262
H 17.2682905 20.115006 -41.7540704
C 18.5555244 18.5806105 -42.3633946
O 19.5022617 19.2022987 -41.8920977

C 16.5356863 18.3408313 -40.8014493
H 15.5546198 18.7709715 -40.5929376
H 16.3868088 17.2899072 -41.0560764
C 17.3783728 18.4041283 -39.5137768
H 18.2761985 17.8007418 -39.655826
H 16.803368 17.9420041 -38.7110996
C 17.8159365 19.8120997 -39.0658109
H 18.4907804 20.2290409 -39.816017
H 18.3932875 19.7217441 -38.1440225
N 16.6897875 20.7491832 -38.8609668
H 16.5665419 21.4502419 -39.5716769
C 15.8807065 20.8193544 -37.8169131
N 14.9853566 21.7589121 -37.7435133
H 14.3645694 21.8061651 -36.956886
H 14.8726153 22.4170928 -38.4946404
N 15.9472023 19.9843426 -36.8189475
H 16.6241288 19.2455779 -36.8502505
H 15.2949912 20.0477172 -36.0589826
N 18.7300829 17.5290883 -43.1764286
H 17.9068943 17.0488226 -43.5306661
C 20.0684893 17.0390531 -43.5746573
H 20.7065707 16.9291063 -42.6964889
C 20.7865705 18.0002679 -44.5366372
O 22.0134416 18.0053269 -44.5586972
C 19.9813346 15.699411 -44.3219547
H 19.304658 15.8747004 -45.1616404
H 20.9671976 15.5209637 -44.7569981
C 19.5628823 14.3578569 -43.6784448
H 20.3290906 14.0366348 -42.971993
H 18.6129878 14.4437749 -43.1562454
C 19.4543242 13.3380914 -44.844005
H 20.418487 13.3271226 -45.354375
H 18.7167883 13.694725 -45.5669937
C 19.1362141 11.8730891 -44.4980398
H 19.490443 11.6480878 -43.4892399
H 19.686472 11.2395421 -45.2001729
N 17.6955314 11.5486037 -44.6343123
H 17.1272324 12.0169392 -43.9401613
H 17.4806865 10.5531329 -44.5600195
H 17.3202713 11.7308229 -45.5705452
N 20.0478271 18.6624719 -45.4364741
H 19.046923 18.5093921 -45.436498
C 20.6358853 19.3309775 -46.6085273
H 21.6749389 19.5607893 -46.3684505
C 20.0541746 20.7091617 -46.9622581
O 20.3152965 21.2210728 -48.0515675

C 20.6868561 18.354292 -47.7957786
H 21.0473326 18.879063 -48.6814314
H 21.4566581 17.6177888 -47.5613333
C 19.4373287 17.602644 -48.1667651
C 19.394892 16.1940631 -48.1271936
H 20.196105 15.6434021 -47.6560693
C 18.3451897 15.5113149 -48.7728262
H 18.2923324 14.4372359 -48.7484014
C 17.3896775 16.2497781 -49.5044677
O 16.5031357 15.6745726 -50.3589183
H 16.8175994 14.8393012 -50.723428
C 17.3695605 17.6457992 -49.4114359
H 16.6398896 18.1806343 -49.9827419
C 18.3848271 18.32481 -48.7295873
H 18.4231516 19.3990316 -48.7319403
N 19.3517895 21.3539171 -46.024161
H 19.1651048 20.8775033 -45.1539639
C 19.0384741 22.7911583 -46.0750765
H 18.3539854 22.9771147 -45.2457682
C 18.2660689 23.2374827 -47.3416898
O 18.3498463 24.3844502 -47.7803859
C 20.3254224 23.5929801 -45.7682459
H 21.0190659 23.4837652 -46.6030665
H 20.8007875 23.1682607 -44.8823023
C 20.0831759 25.0814136 -45.500918
O 19.0655738 25.4065578 -44.8420731
O 20.8655128 25.9146396 -46.0124582
N 17.4548563 22.3556176 -47.9295338
H 17.4238769 21.424189 -47.5465261
C 16.4823397 22.7281121 -48.9710723
H 16.9464183 23.4477338 -49.6473765
C 15.24281 23.3977265 -48.3725054
O 14.9522023 23.2457856 -47.1836795
C 16.0422905 21.5162724 -49.8130752
H 15.2603537 21.8499514 -50.4920367
C 17.2011516 21.0558416 -50.7125561
H 18.1143336 20.9147098 -50.1404907
H 17.4026366 21.8357731 -51.4439333
H 16.943333 20.1436236 -51.2459316
C 15.398919 20.4182088 -48.9405732
H 16.1665696 19.8238888 -48.4571155
H 14.7955406 20.8598812 -48.151009
C 14.4493492 19.5350595 -49.7478417
H 14.8862927 19.2531794 -50.7036183
H 13.5268763 20.0795328 -49.9194258
H 14.2201392 18.6408149 -49.1834476

N 14.4360211 24.010578 -49.2375354
H 14.7402083 24.0446147 -50.2085445
C 12.9993631 24.158699 -49.0076405
H 12.8175786 24.2573771 -47.9397338
C 12.2662528 22.9046082 -49.5103378
O 12.6107608 22.3634176 -50.5604784
C 12.5234879 25.4517901 -49.6859483
H 13.0406288 26.283309 -49.2048236
H 12.830272 25.44303 -50.731898
C 11.0112581 25.7338392 -49.6079951
H 10.8575365 26.7986213 -49.7828324
H 10.658697 25.5126149 -48.6002629
C 10.1424015 24.9839405 -50.6247752
O 10.5934761 24.3664138 -51.5757024
N 8.8434225 24.9954737 -50.4307784
H 8.2650384 24.4828781 -51.0906918
H 8.4331033 25.5005896 -49.6701504
N 11.2359635 22.4621827 -48.7844512
H 11.0578115 22.9049529 -47.8891113
C 10.3429926 21.383246 -49.214284
H 10.5949631 21.0920705 -50.235015
C 8.8711568 21.8264889 -49.2272665
O 8.3637704 22.3460571 -48.2286926
C 10.5732244 20.1618264 -48.309774
H 10.3332883 20.4190762 -47.2777976
H 11.6227969 19.8672222 -48.3609254
S 9.5400098 18.7650291 -48.8429061
H 8.3402798 19.3259352 -48.6493615
N 8.1565084 21.4877271 -50.299685
H 8.6580875 21.1050509 -51.0997332
C 6.6915622 21.4723115 -50.3796701
H 6.2622286 21.8728319 -49.4665579
C 6.2201094 20.031677 -50.5303368
O 6.4399128 19.4158451 -51.5664035
C 6.1943466 22.3420754 -51.5434893
H 6.7074003 22.060723 -52.4640156
C 4.6826819 22.2560971 -51.7615391
H 4.1511206 22.4652032 -50.8329504
H 4.408085 21.2625488 -52.117419
H 4.3832472 22.9787312 -52.5207642
O 6.4597851 23.693421 -51.2481149
H 6.0035097 23.908674 -50.423727
N 5.5750189 19.4858299 -49.5003679
H 5.4489048 20.0421054 -48.6609263
C 4.8934822 18.1915638 -49.5707935
H 5.3805521 17.5583986 -50.3075649

H 4.9561912 17.6920084 -48.6044006
C 3.4178491 18.3381219 -49.9451647
O 2.7499778 19.2457229 -49.4508098
N 2.8759735 17.4192718 -50.7447628
H 3.489359 16.728848 -51.172119
C 1.4218424 17.2768095 -50.9304672
H 0.9352328 17.8212694 -50.1261785
C 0.9560668 15.8305307 -50.7697592
O 1.6406314 14.8966804 -51.1867693
C 0.9136146 17.8905378 -52.2565359
H -0.1448096 17.6360649 -52.3435319
C 1.6387051 17.3248981 -53.5004349
H 2.6843131 17.6363338 -53.4916327
H 1.6106378 16.2363657 -53.4623999
C 0.9961788 19.4249524 -52.1858548
H 2.0365676 19.7521869 -52.1926341
H 0.5247676 19.7783184 -51.266841
H 0.4642133 19.8755679 -53.0218273
C 1.0052803 17.7519525 -54.8316522
H 1.1088672 18.8260369 -54.9781292
H -0.0515642 17.481976 -54.8436029
H 1.510511 17.2397663 -55.65089
N -0.2477491 15.6495421 -50.2260329
H -0.7465903 16.4668413 -49.8863459
C -0.9656272 14.3704334 -50.212057
H -0.6470092 13.784003 -51.0743246
C -2.4736961 14.6046495 -50.3361702
O -2.9923013 15.6108318 -49.855645
C -0.6345655 13.5626088 -48.9469876
H -1.035592 14.063649 -48.0667914
H 0.4483293 13.4718768 -48.8486547
O -1.2039403 12.2647033 -49.0661658
H -0.8230701 11.6633302 -48.3915009
N -3.189996 13.6624306 -50.9545782
H -2.693636 12.8593069 -51.3085801
C -4.6570956 13.7038033 -51.1109514
H -4.9453094 14.6681594 -51.5324737
C -5.3659256 13.5807981 -49.7505522
O -6.4750307 14.0756237 -49.5707605
C -5.0838402 12.5810381 -52.0946599
H -4.5868338 11.656628 -51.7934535
C -6.60297 12.3174183 -52.0927149
H -7.1427278 13.2367823 -52.3255887
H -6.9278242 11.951214 -51.1188279
H -6.8636599 11.5560362 -52.8261546
C -4.6265523 12.9470809 -53.5247128

H -5.2699191 13.744181 -53.8956398
H -3.6030739 13.3215718 -53.5000957
C -4.6555746 11.788384 -54.5300713
H -5.6777838 11.458972 -54.7113472
H -4.0625365 10.9539342 -54.1543668
H -4.2321241 12.1260136 -55.4769672
N -4.7271601 12.913471 -48.788543
H -3.775143 12.6272275 -48.9723444
C -5.2999645 12.5558781 -47.4919011
H -6.3581933 12.3426238 -47.6238231
C -5.12327 13.6827605 -46.4548777
O -3.9915082 13.9538372 -46.0403971
C -4.6452439 11.2446981 -47.0565748
H -3.587913 11.2809028 -47.3174804
H -5.1004324 10.4159779 -47.6001787
O -4.7366918 11.0128571 -45.6723862
H -5.6758702 11.0025864 -45.3613888
N -6.2181431 14.2734505 -45.9265974
C -6.1448261 15.2535177 -44.8392723
H -5.5037262 16.0768951 -45.1469994
C -5.5994107 14.6593758 -43.5365247
O -4.990551 15.3639187 -42.7414758
C -7.5767455 15.7683423 -44.6357585
H -8.056633 15.2366621 -43.8112928
H -7.5919532 16.8425494 -44.4489233
C -7.6005661 14.1249832 -46.3585443
H -7.6892693 14.0050037 -47.4364082
H -8.051605 13.2737373 -45.845865
C -8.2932888 15.4153499 -45.9362787
H -8.111043 16.1900229 -46.680453
H -9.3633916 15.2698172 -45.7853592
N -5.798928 13.3603836 -43.3244477
H -6.3334307 12.8472974 -44.0199305
C -5.3819279 12.5909009 -42.1533687
H -5.5086175 13.2194111 -41.2710266
C -3.889537 12.1953296 -42.1819728
O -3.2267231 12.2061379 -41.1417984
C -6.3412377 11.3905832 -41.9922851
H -7.3113502 11.7703061 -41.6675729
H -5.9575394 10.7498656 -41.1971473
C -6.5663179 10.5388814 -43.2538189
O -6.908747 11.078591 -44.3329274
O -6.3508923 9.3107568 -43.1812859
N -3.3008267 11.9683216 -43.3619274
H -3.8866016 11.9470974 -44.1933319
C -1.8430178 11.9831623 -43.5618261

H -1.3723892 11.257173 -42.9150736
C -1.2618897 13.3502526 -43.1982805
O -0.4067809 13.4546671 -42.3135894
C -1.4776396 11.6187214 -45.0103698
H -2.1218696 12.1563712 -45.7031098
H -0.4492902 11.9202012 -45.2048059
C -1.5826308 10.1109484 -45.2530965
H -2.542401 9.7508069 -44.8866851
H -0.8029225 9.6052696 -44.6769365
C -1.4265517 9.7523301 -46.7342513
O -2.1887154 8.9014817 -47.2378949
O -0.4387212 10.1870761 -47.3704002
N -1.8238071 14.3981459 -43.7998905
H -2.5652821 14.2183388 -44.4716944
C -1.3629664 15.7743681 -43.644951
H -0.2966211 15.7971953 -43.8619811
C -1.5011144 16.2671664 -42.1931932
O -0.5669668 16.8774303 -41.6842395
C -2.0937697 16.6432986 -44.6956286
H -3.1616424 16.4331521 -44.6208048
C -1.6453572 16.3062192 -46.1415492
H -1.8060816 15.2459139 -46.3228022
H -2.2857106 16.8442621 -46.8391984
C -1.9169274 18.1412204 -44.4519846
H -0.8624135 18.3648704 -44.4776171
H -2.351247 18.4235725 -43.4927015
H -2.4173335 18.7036014 -45.2397571
C -0.1850993 16.6247081 -46.5047295
H 0.502714 16.0852862 -45.8550906
H 0.005957 17.6948585 -46.4390428
H -0.0012286 16.3148047 -47.5317343
N -2.5671139 15.9256739 -41.4689525
H -3.3267024 15.4447242 -41.9411177
C -2.7735512 16.3191514 -40.0675664
H -2.7786111 17.4067291 -40.0129945
C -1.6436206 15.8404344 -39.1348382
O -1.1884872 16.6049745 -38.2795479
C -4.1712491 15.8244989 -39.6523631
H -4.8964345 16.3669418 -40.2600162
H -4.276831 14.7663523 -39.8970248
C -4.5547229 16.0502673 -38.1793378
H -5.6347657 16.1922723 -38.1337433
H -4.0852274 16.9595111 -37.8033673
C -4.2106249 14.87867 -37.2602295
O -3.478791 13.9624486 -37.5900405
N -4.7524033 14.838826 -36.0651956

H -4.5608134 14.0108061 -35.5281391
H -5.3835633 15.5549238 -35.7602172
N -1.1004638 14.6424705 -39.3708469
H -1.5013084 14.0764096 -40.106404
C 0.0418472 14.1322373 -38.6116889
H -0.0436926 14.4501244 -37.5713118
C 1.3850099 14.6518156 -39.1239408
O 2.2660308 14.8908551 -38.3035319
C 0.0435491 12.6104591 -38.6527647
H 1.0465421 12.2488474 -38.4138007
H -0.2262749 12.3017169 -39.6598596
S -1.1117245 11.9445062 -37.4241918
H -2.0986728 12.845829 -37.6105998
N 1.5575739 14.8170369 -40.4417058
H 0.8054631 14.5690666 -41.0762493
C 2.7701594 15.422793 -40.987119
H 3.6444812 14.9045317 -40.5883586
C 2.8696392 16.8863013 -40.5636503
O 3.5847836 17.2487937 -39.6283336
C 2.7907183 15.3258836 -42.5238653
H 1.9156234 15.7985274 -42.9702768
H 2.7822393 14.2994419 -42.8465516
H 3.7036186 15.7788831 -42.9060338
N 2.1002966 17.7180647 -41.2791163
H 1.1970245 17.3057366 -41.4945638
C 2.6117307 18.5517644 -42.3861192
H 3.1341734 17.8723516 -43.0516016
C 1.3970127 19.0600589 -43.2242848
H 1.358215 18.4262558 -44.0974844
H 0.5018309 18.6692998 -42.7733033
C 1.1166488 20.5727843 -43.5444475
H 1.2915895 21.151315 -42.6635274
H 1.8411256 20.9728381 -44.2381746
C -0.3230066 20.9360951 -43.9914732
H -1.0227159 20.3720331 -43.3859128
H -0.4699022 20.6178342 -45.0179328
C -0.6963571 22.4298742 -43.8025919
H 0.1995555 22.9755746 -43.4898631
H -1.4260614 22.4979107 -42.9928553
N -1.2635441 23.0865246 -45.012343
H -0.5895041 23.0906335 -45.7804691
H -1.4992876 24.0597524 -44.8902245
H -2.0834365 22.6400856 -45.4285437
C 3.7642777 19.4606364 -41.9383207
O 4.6934606 18.9814363 -41.3095185
N 3.6311882 20.7771193 -41.9989438

H 2.8876535 21.1417414 -42.5604266
C 4.0788258 21.6000937 -40.8865894
H 5.1237588 21.3834285 -40.6673247
C 3.9999911 23.0820359 -41.3327063
H 3.0493725 23.2473995 -41.8409777
H 4.7944901 23.2717171 -42.0553756
C 4.1026739 24.1179475 -40.2399803
N 3.0918861 25.0331692 -39.9409579
C 3.4223691 25.5589047 -38.7527186
H 2.8010558 26.2448663 -38.192991
N 4.5752577 25.0358408 -38.3042514
H 4.8930887 25.1113105 -37.3429746
C 5.0364284 24.1390869 -39.2428601
H 5.8685618 23.4582498 -39.1352582
C 3.2420288 21.199852 -39.6385361
O 3.0797663 22.025223 -38.7500341
N 2.530232 20.0383135 -39.640792
H 2.865367 19.2416506 -40.1623869
C 1.4823966 19.8353562 -38.7041374
H 1.2481924 20.7922532 -38.2384218
C 2.0858318 19.0933861 -37.5361898
O 3.067777 19.6165891 -37.0050026
C 0.1413169 19.4928001 -39.3442083
H 0.1442248 18.4588372 -39.6817178
H -0.6141341 19.569777 -38.5611991
C -0.267998 20.4123699 -40.5175301
H 0.1183068 19.9509151 -41.3678312
C -1.7776922 20.4353344 -40.70414
H -2.2492346 20.8376265 -39.8066394
H -2.1313518 19.4171078 -40.8620015
H -2.0806715 21.041301 -41.5528518
C 0.1944014 21.8747087 -40.5369619
H 1.2344464 22.003532 -40.7787416
H 0.035205 22.3050513 -39.5470494
H -0.3843236 22.4742456 -41.2283432
N 1.4878269 18.020571 -37.0275607
H 0.680745 17.619781 -37.490007
C 1.8370793 17.567107 -35.682473
H 1.6480608 18.3923588 -34.994206
C 3.3255548 17.1909504 -35.5105984
O 3.975249 17.6990186 -34.5966676
C 0.897452 16.4265003 -35.2938252
H 1.0844826 15.5580817 -35.9233497
H -0.1416476 16.7397962 -35.4098183
H 1.0723946 16.150676 -34.2533891
N 3.895938 16.3935091 -36.4204664

H 3.3273487 16.0340792 -37.1822979
C 5.2891756 15.9425756 -36.3358269
H 5.4806631 15.6036171 -35.3165186
C 6.3276383 17.0397494 -36.6405866
O 7.4910534 16.9063308 -36.2670223
C 5.4618095 14.744717 -37.274927
H 5.2733635 15.0489023 -38.3063018
H 4.7617187 13.9547081 -37.0015401
H 6.47971 14.3605827 -37.1961688
N 5.9285687 18.1328717 -37.2982429
H 4.962632 18.1795496 -37.6016175
C 6.8314934 19.2418052 -37.635999
H 7.8429605 18.8505207 -37.6832907
C 6.844892 20.3829303 -36.6121256
O 7.5699517 21.3645417 -36.8076774
C 6.4883151 19.7654549 -39.0249614
H 5.4763035 20.1549587 -38.9918722
H 6.5452826 18.940506 -39.7349417
O 7.3909096 20.7802827 -39.4271918
H 7.5897174 21.3108912 -38.6363837
N 6.0301942 20.3258281 -35.5583977
H 5.4633655 19.4990513 -35.4131305
C 5.9102691 21.4436743 -34.6242259
H 5.8569889 22.3420848 -35.2375565
C 7.1639527 21.593333 -33.7492034
O 7.6308825 20.6298769 -33.149975
C 4.6035516 21.3149437 -33.8160339
H 3.9610644 20.5586151 -34.2705869
H 4.8261419 20.983383 -32.8007128
C 3.8146552 22.6339158 -33.7630863
H 4.3761121 23.361526 -33.1761685
H 2.8597389 22.454182 -33.2696363
C 3.5503843 23.2164719 -35.1512038
O 4.0029425 24.3035214 -35.4804174
N 2.9488639 22.4629638 -36.0411139
H 2.950617 22.7735293 -37.0102779
H 2.7918841 21.4817724 -35.8719266
N 7.7210904 22.8081187 -33.6914249
H 7.2987774 23.5567177 -34.2190106
C 8.9972279 23.0788989 -33.0131334
H 9.0701395 24.1482844 -32.8145984
H 9.0214978 22.5526099 -32.0586359
C 10.2493908 22.6746477 -33.805537
O 11.3286693 22.5814544 -33.2268775
N 10.1271567 22.3925806 -35.1093067
H 9.2142375 22.4390954 -35.5407744

C 11.2828804 22.1739636 -35.9765919
H 12.1470555 21.9182832 -35.3639204
C 11.6311631 23.4425166 -36.7703543
O 10.8468171 23.8739616 -37.6062429
C 11.0248787 21.0051895 -36.9419587
H 10.2282802 21.2982624 -37.627223
H 11.9359448 20.8776523 -37.5163873
C 10.6521745 19.6372191 -36.3484215
H 9.6886744 19.7031776 -35.8480046
C 10.554009 18.6256642 -37.4922608
H 11.5421073 18.4392663 -37.908598
H 9.9033941 19.0025286 -38.2786216
H 10.1402736 17.6890333 -37.1141196
C 11.700169 19.1190278 -35.363519
H 12.6761902 19.0615675 -35.8409176
H 11.4049478 18.1266817 -35.0192001
H 11.7434391 19.7788958 -34.4960605
N 12.8948299 23.8661966 -36.7091582
H 13.3352564 23.7065076 -35.812184
C 13.4155394 25.0179326 -37.4790076
H 12.6681205 25.8145446 -37.4282954
C 13.6732908 24.7387133 -38.9836209
O 14.3679382 25.4938641 -39.6582383
C 14.7076514 25.5493859 -36.8171534
H 15.5330047 24.8870948 -37.0856383
H 14.9254605 26.5347873 -37.2302733
C 14.686988 25.6625318 -35.2845744
H 15.5866248 26.1902592 -34.9596257
H 14.7320807 24.6561241 -34.8586402
C 13.4378158 26.3886491 -34.7758366
O 13.4280188 27.6368107 -34.819975
O 12.4900572 25.6633847 -34.4019596
N 13.2648524 23.5772005 -39.5105643
H 12.5354699 23.085907 -39.0068225
C 13.5570177 23.1795007 -40.8976494
H 14.5254876 23.6009869 -41.1761233
C 12.542468 23.7627457 -41.8860466
O 11.3445727 23.7642826 -41.6133993
C 13.6717175 21.6505914 -41.0511484
H 14.6737933 21.3409764 -40.7633607
H 13.5409867 21.3814689 -42.0977122
C 12.6807254 20.8409029 -40.234541
O 13.0819143 19.9571612 -39.4998137
N 11.4063889 21.1652132 -40.2593715
H 10.7663929 20.6171536 -39.7086994
H 11.0835354 21.9909526 -40.7547637

N 13.0133136 24.1123106 -43.0888082
H 14.003369 23.9861169 -43.252692
C 12.2559559 24.8063065 -44.14478
H 11.634147 25.5703974 -43.6725806
C 11.2900693 23.8636724 -44.8836361
O 11.4794884 23.5296958 -46.0528063
C 13.2402964 25.5207442 -45.0935189
H 13.7974318 24.7585126 -45.6299273
H 12.6747639 26.1075278 -45.8195301
C 14.2367 26.4457212 -44.363787
H 13.6782029 27.2076148 -43.8182901
H 14.8081668 25.8651489 -43.6389009
C 15.2530414 27.1445263 -45.2802328
H 15.9662655 27.6665972 -44.6388995
H 14.7410762 27.8844194 -45.8974409
C 16.0017683 26.1590706 -46.1895801
H 15.4383225 26.0365783 -47.1185131
H 16.0485386 25.1791219 -45.7028254
N 17.3833513 26.6013149 -46.4784672
H 17.4501689 27.5257558 -46.8646949
H 17.8394313 25.9286781 -47.0934372
H 17.9672793 26.5200239 -45.6378488
N 10.2586788 23.4106932 -44.1767312
H 10.1841574 23.7178249 -43.2124954
C 9.2046284 22.5279462 -44.6902692
H 9.4646982 22.2318761 -45.7066837
C 7.8664666 23.2561846 -44.773848
O 7.5173305 24.0732073 -43.9268036
C 9.0728533 21.2239852 -43.8778855
H 8.316634 20.6091786 -44.3642886
C 10.3766276 20.4230278 -43.8853184
H 11.1563743 20.9672999 -43.3545985
H 10.6901088 20.2389319 -44.9118237
H 10.2204614 19.4614051 -43.3955968
C 8.6444678 21.4437578 -42.4225151
H 9.3296493 22.1190396 -41.9159669
H 8.6253233 20.491972 -41.8909169
H 7.6444986 21.8751438 -42.3818422
N 7.0848025 22.9020958 -45.7835561
H 7.4542566 22.2468272 -46.4660422
C 5.688535 23.2901281 -45.9603944
H 5.2592379 23.5642908 -44.9971347
C 4.9368154 22.090787 -46.5162855
O 5.5175932 21.2799915 -47.2402207
C 5.572196 24.4861637 -46.9093264
H 6.1315988 25.3254024 -46.4927753

H 4.5253632 24.7775338 -47.0057321
O 6.0920421 24.166681 -48.1936715
H 6.9019288 23.6412406 -48.069159
N 3.6649475 21.9328174 -46.1572492
H 3.1924113 22.6509743 -45.6290061
C 2.8540294 20.891737 -46.7718369
H 3.2603949 20.7420939 -47.768523
C 1.4232672 21.3469321 -46.9993605
O 0.9655742 22.2461308 -46.2958749
C 2.8723023 19.5311039 -46.0727033
H 1.8998189 19.3593649 -45.6756641
H 2.831625 18.8431272 -46.9167144
C 4.0238134 18.9905664 -45.2169582
C 4.3071869 17.6253821 -45.3860567
H 3.6888653 17.0224533 -46.0309537
C 4.8919789 19.744078 -44.3979068
H 4.7530479 20.7996534 -44.2590226
C 5.3992881 17.0235059 -44.752768
H 5.5957283 15.9801922 -44.9285487
C 5.9619852 19.1264943 -43.7177794
H 6.5828412 19.700868 -43.0462872
C 6.2154413 17.761582 -43.8888187
H 7.0295482 17.2877361 -43.3583586
N 0.7049211 20.6843602 -47.9071269
H 1.1580329 19.9917536 -48.4999263
C -0.7426341 20.8387991 -48.0115702
H -1.0824204 20.9641876 -46.9898499
C -1.5361428 19.617803 -48.4852348
O -0.9924186 18.6595985 -49.0384563
C -1.11455 22.1121096 -48.8003637
H -1.7026334 21.8618073 -49.6844732
H -0.2187602 22.6483505 -49.119513
C -1.9431406 22.9948283 -47.8556361
O -2.8487189 22.4398679 -47.1824651
O -1.4945848 24.1167787 -47.540539
N -2.8489941 19.6723543 -48.2388324
H -3.2118883 20.5386921 -47.8451067
C -3.8093695 18.7281656 -48.8164363
H -3.3973647 17.7249245 -48.7310293
C -3.9734597 19.0752603 -50.2936677
O -4.3973474 20.175993 -50.6415666
C -5.1732061 18.7435119 -48.0992768
H -5.6730347 19.6950951 -48.2825975
C -6.0637773 17.6068255 -48.6193746
H -5.6052813 16.639463 -48.4108445
H -6.2151491 17.7031978 -49.6940242

H -7.0395154 17.6576898 -48.1394967
C -5.0359728 18.5600012 -46.5826229
H -4.4763312 17.6507279 -46.373113
H -6.0235974 18.4938287 -46.1264681
H -4.5200769 19.4177832 -46.1531065
N -3.6215795 18.1436875 -51.1741785
H -3.2879382 17.2507786 -50.8282856
C -3.6115106 18.3903012 -52.6077545
H -4.5005105 18.9612831 -52.879691
H -2.7342712 18.9845956 -52.864576
C -3.5764643 17.1062873 -53.4192378
O -2.760003 16.2192524 -53.1788795
N -4.4347018 17.0516582 -54.4326559
H -5.0732818 17.8204822 -54.5581563
C -4.3864658 16.05468 -55.4956002
H -4.0915978 15.0912157 -55.0883004
C -3.3390088 16.4662952 -56.5310674
O -3.4495113 17.5140222 -57.164842
C -5.7890218 15.9175663 -56.0942179
H -6.1264015 16.9003061 -56.4288134
H -6.4684305 15.5787826 -55.3090581
C -5.884318 14.9468145 -57.2730016
O -4.8383242 14.4918336 -57.7921059
O -7.0322555 14.7422459 -57.7062923
N -2.3237965 15.6294866 -56.7222922
H -2.3348741 14.7515165 -56.2290766
C -1.1959614 15.917138 -57.5968154
H -0.8042434 16.9026035 -57.3349828
C -1.5477935 15.9520276 -59.0940706
O -0.7986989 16.5414193 -59.8719076
C -0.1261968 14.8713849 -57.2991538
H -0.4332321 13.8964363 -57.6832249
H 0.0211865 14.8034629 -56.2217764
H 0.8044678 15.171372 -57.7763393
N -2.6894224 15.3899282 -59.5099429
H -3.3057519 14.9434825 -58.8268714
C -3.1680469 15.5081525 -60.8889374
H -2.2977069 15.4764396 -61.5426236
C -3.8714604 16.8578481 -61.1353749
O -3.8881519 17.3427345 -62.2717246
C -4.0589673 14.2899356 -61.2019821
H -3.5800509 13.3944349 -60.8022451
H -5.0094949 14.4145144 -60.6792547
C -4.3336983 14.054691 -62.7013104
H -4.8066073 14.9365606 -63.1323418
C -3.0607759 13.732287 -63.4923323

H -2.5395826 12.8897156 -63.037984
H -2.3987076 14.5952794 -63.5160859
H -3.3198034 13.4669686 -64.5171777
C -5.2831101 12.8667808 -62.868187
H -4.8269732 11.9632398 -62.4652122
H -5.5106869 12.7195113 -63.9246257
H -6.2135557 13.0676634 -62.3361046
N -4.3438173 17.5317856 -60.0767708
H -4.239743 17.0913676 -59.1689351
C -5.1392953 18.7670931 -60.1508346
H -5.3101043 19.014173 -61.1954728
C -4.4652452 20.0192241 -59.553186
O -4.7247452 21.1197762 -60.036646
C -6.5275032 18.4864972 -59.5562995
H -7.1041324 19.4099779 -59.5149897
H -6.4332717 18.0886791 -58.546576
C -7.2894454 17.5033969 -60.4354308
O -7.5563357 17.7986585 -61.5883171
N -7.5704357 16.2932746 -60.0008698
H -8.0536807 15.6856149 -60.632247
H -7.3300548 15.9419432 -59.0695682
N -3.5291447 19.8666245 -58.6129897
H -3.3778296 18.9328279 -58.2480178
C -2.7161973 20.948246 -58.0426512
H -3.3974747 21.6988151 -57.6398965
C -1.8381347 21.6244684 -59.108357
O -1.3603305 20.9679296 -60.0376461
C -1.8708616 20.3801509 -56.8857777
H -1.2155043 19.5960797 -57.2688558
H -2.5403692 19.9339514 -56.1488536
C -1.0113161 21.4298223 -56.1713507
H -0.2443703 21.7912918 -56.8567282
H -0.5059511 20.9491715 -55.3327881
S -1.9402459 22.8506813 -55.5394919
C -0.5701828 23.8292679 -54.8690719
H -0.9613234 24.74235 -54.4197375
H 0.1193026 24.0926016 -55.6715556
H -0.0410525 23.252321 -54.1098706
N -1.6015408 22.9369568 -58.9811233
H -1.9423219 23.4054305 -58.1486339
C -0.8192637 23.7390643 -59.9395053
H -0.2576772 23.064157 -60.5851844
C 0.2282464 24.5957342 -59.2270807
O 0.004776 25.0706294 -58.1183271
C -1.7509353 24.6002576 -60.822006
H -2.2485006 25.3361505 -60.1875853

H -1.1470927 25.1432575 -61.5512182
C -2.8355023 23.8041089 -61.576147
H -3.4692309 24.5095959 -62.1148217
H -3.4670243 23.293783 -60.85025
C -2.285634 22.7794283 -62.5838831
H -1.469829 22.2166229 -62.1362081
H -1.8727529 23.3160784 -63.440655
N -3.3480238 21.8600974 -63.0454371
H -4.1592275 22.2924263 -63.4494333
C -3.3712553 20.539867 -62.9367282
N -4.3632524 19.8552986 -63.436589
H -4.394121 18.8635073 -63.2050823
H -5.2093213 20.3214733 -63.7056537
N -2.4470361 19.8412767 -62.3456859
H -1.8072108 20.3142319 -61.7116381
H -2.5589807 18.8412342 -62.2636225
N 1.3477479 24.8147152 -59.9086339
H 1.3980397 24.4714973 -60.8561952
C 2.4801481 25.6382775 -59.4817666
H 2.171737 26.310206 -58.6796175
C 2.9540436 26.4820188 -60.6742369
O 2.5493109 26.2124622 -61.8048482
C 3.6101897 24.7291168 -58.9715134
H 3.9556652 24.0994097 -59.7921348
H 4.4519802 25.3525014 -58.6710504
C 3.2414508 23.8502424 -57.790578
C 3.2255769 24.3988016 -56.4940977
H 3.4578802 25.4439975 -56.3432923
C 2.944317 22.484486 -57.9805326
H 2.957988 22.0603603 -58.9749413
C 2.9118273 23.5871558 -55.3886793
H 2.893409 23.9989979 -54.390721
C 2.6376701 21.6672874 -56.8746023
H 2.4174114 20.6212776 -57.0198648
C 2.6196309 22.2218223 -55.5761608
O 2.3179465 21.4526177 -54.4997302
H 2.18435 20.5319836 -54.724826
N 3.8334992 27.4628261 -60.4509498
H 4.2082822 27.584594 -59.5219213
C 4.4939213 28.1620494 -61.5616058
H 3.7309422 28.5488971 -62.2384471
C 5.3882736 27.2020676 -62.3485732
O 6.0176283 26.3114741 -61.7616727
C 5.3217809 29.3477141 -61.0436929
H 6.091829 28.9980095 -60.3569351
H 4.6612574 30.029898 -60.5071673

O 5.9393037 30.0538829 -62.1032955
H 6.7243398 29.5613144 -62.4093461
N 5.5820288 27.4940815 -63.6317522
H 5.0106585 28.1958685 -64.0746187
C 6.7307802 26.9874338 -64.3760478
H 6.6366855 25.9085016 -64.4254029
C 8.028407 27.3558119 -63.6304401
O 8.1187891 28.4400932 -63.0417593
C 6.7479946 27.5450176 -65.8100117
H 7.719706 27.3149662 -66.2505881
H 6.6485008 28.6315395 -65.7731873
C 5.6672195 26.9697377 -66.7358325
O 4.772257 26.2412801 -66.2508656
O 5.7522914 27.2543315 -67.9504335
N 9.0046514 26.4438149 -63.5827792
H 8.8767 25.5757138 -64.0999271
C 10.2928452 26.6834173 -62.9168859
H 10.9628395 25.863104 -63.1596956
H 10.726691 27.5994604 -63.3176861
C 10.2581107 26.8168125 -61.3836782
O 11.1929088 27.3706979 -60.812188
N 9.2068629 26.3463782 -60.7000323
H 8.4713915 25.8971 -61.2280263
C 9.0731089 26.4374344 -59.2283582
H 9.1851045 27.4791121 -58.9258279
C 10.1069491 25.6136815 -58.4432941
O 10.4836519 25.994552 -57.3288833
C 7.6897912 25.9495096 -58.7796666
H 7.6527518 25.9341602 -57.6888603
H 7.5223822 24.9374057 -59.148062
O 6.6580577 26.7898418 -59.2502181
H 6.5249451 26.5892104 -60.1998395
N 10.5092653 24.4553591 -58.9775317
H 10.148396 24.2047745 -59.8925566
C 11.2703098 23.4359545 -58.2511685
H 11.53296 23.831252 -57.2725145
C 12.5826537 23.0539197 -58.9343996
O 12.7237566 23.0892687 -60.153064
C 10.4016771 22.1945978 -58.0069762
H 10.1678104 21.7295215 -58.9665107
H 10.9725225 21.4720044 -57.4234963
C 9.1174901 22.4918067 -57.2616053
C 9.1440574 22.7882535 -55.8861933
H 10.0770687 22.7663086 -55.3446341
C 7.8965917 22.5106125 -57.9559699
H 7.8822044 22.2996273 -59.0114969

C 7.9532069 23.1130515 -55.2127899
H 7.9771022 23.3415634 -54.1554797
C 6.7052395 22.8277978 -57.284616
H 5.7704859 22.841606 -57.8236995
C 6.7337942 23.135257 -55.9133376
H 5.8189192 23.3828901 -55.3936062
N 13.517583 22.5839715 -58.1188466
H 13.3072881 22.5350507 -57.1278463
C 14.8179288 22.0601079 -58.5444263
H 15.0314648 22.3351386 -59.5799209
C 14.8820061 20.5283609 -58.4571595
O 15.7122647 19.8905221 -59.1081171
C 15.861854 22.6970743 -57.6264017
H 15.4958654 22.6849266 -56.6000881
H 16.7664742 22.1004187 -57.6568063
C 16.1785143 24.1454572 -58.0345006
H 16.4935678 24.6931106 -57.143149
H 15.2907343 24.6425045 -58.4312527
C 17.305602 24.1476694 -59.067901
O 17.1190339 23.5958259 -60.1692975
O 18.44763 24.4781666 -58.6847995
N 14.0008482 19.9431131 -57.6415347
H 13.375073 20.5442361 -57.11928
C 13.8557196 18.5135042 -57.3961515
H 14.3000178 17.9505765 -58.2132188
C 12.3562485 18.1809218 -57.2916426
O 11.6055412 18.9392478 -56.6695756
C 14.5613807 18.1437631 -56.0764476
H 13.9703084 18.5856413 -55.2756477
H 14.5331341 17.0604963 -55.9537592
C 16.0075358 18.6382877 -55.8749913
H 16.0774433 19.7022195 -56.0961422
C 16.4272853 18.4705187 -54.4170553
H 16.4803628 17.4129 -54.1558347
H 15.7119752 18.972793 -53.7650525
H 17.3987076 18.9361346 -54.2814031
C 17.0207935 17.8886108 -56.7338335
H 16.9321508 16.814618 -56.5697651
H 18.0321975 18.2073195 -56.4892937
H 16.8337156 18.1120355 -57.7796173
N 11.9268476 17.0254245 -57.802589
H 12.5667476 16.4679935 -58.3580903
C 10.6134922 16.4408486 -57.4727742
H 10.200647 16.9817986 -56.6240596
C 10.8460628 14.9974708 -57.0338666
O 11.4968357 14.2196056 -57.7343886

C 9.584129 16.5453119 -58.623281
H 9.8891 15.8453796 -59.384544
C 9.5544193 17.9798466 -59.2141413
H 9.2019235 18.6905423 -58.4697116
H 10.5636784 18.2729503 -59.500348
C 8.1844474 16.0604285 -58.2049168
H 7.7911132 16.6907992 -57.4099126
H 8.2346799 15.0332518 -57.8434264
H 7.5072373 16.083636 -59.0554359
C 8.7221937 18.1222675 -60.4791129
H 7.6661354 18.1114382 -60.2270789
H 8.9737782 17.3097286 -61.1536164
H 8.9770545 19.0592215 -60.9698104
N 10.3564225 14.6574374 -55.8469035
H 9.7941865 15.3397182 -55.3446681
C 10.5610505 13.361721 -55.2088406
H 11.1527113 12.721799 -55.8623133
C 9.2265463 12.6740554 -54.9607565
O 8.2716464 13.3041168 -54.5053329
C 11.33476 13.5316808 -53.9000093
H 12.3374437 13.8914798 -54.1303004
H 10.8329836 14.2807638 -53.2847405
C 11.4560679 12.2414929 -53.107116
C 12.2584577 11.1893237 -53.5913009
H 12.8093437 11.3079028 -54.5115507
C 12.3285074 9.9712448 -52.892086
H 12.935959 9.1595666 -53.2719667
C 11.6002187 9.80203 -51.7022888
H 11.6597189 8.8679635 -51.1608939
C 10.7885537 10.8448729 -51.2227153
H 10.2173261 10.7141468 -50.3149552
C 10.7115946 12.0603749 -51.9250526
H 10.0647863 12.8469327 -51.5572318
N 9.1602094 11.3777451 -55.2613215
H 9.967209 10.9333375 -55.6892098
C 7.9328766 10.5948167 -55.1189933
H 7.301889 11.0701356 -54.3646456
C 8.255057 9.1831792 -54.6477118
O 9.0891373 8.509012 -55.2435789
C 7.1597415 10.5440188 -56.4471444
H 7.7114147 9.9222563 -57.1494066
C 5.8095002 9.8629732 -56.2113163
H 5.4896513 10.0212756 -55.1882349
H 5.9140703 8.7885214 -56.3956002
H 5.0585275 10.2781186 -56.8705113
C 7.0334598 11.9183157 -57.1168015

H 6.7794035 12.6694729 -56.3779092
H 6.2773547 11.9109892 -57.8926942
H 7.9869524 12.1928344 -57.5734035
N 7.5304832 8.6922801 -53.6430702
H 6.8760449 9.3036927 -53.1726615
C 7.6681163 7.3252057 -53.1358409
H 8.2068826 6.7406371 -53.8801015
C 6.2876119 6.6673452 -52.9862792
O 5.4097723 7.1751835 -52.2984167
C 8.5623854 7.3067255 -51.8731256
H 9.5579647 7.6240205 -52.1890165
C 8.094785 8.2976582 -50.7891014
H 7.0661867 8.0877915 -50.5071326
H 8.1462975 9.3195321 -51.1659953
H 8.7354529 8.2405992 -49.9112774
C 8.6896144 5.865025 -51.3446308
H 7.7397634 5.5693468 -50.9079072
H 8.8984866 5.198318 -52.1818034
C 9.804598 5.6643442 -50.3096542
H 9.5898272 6.2220642 -49.3977556
H 10.7584064 5.9929064 -50.7211156
H 9.8773923 4.6049462 -50.0589894
N 6.0735872 5.5791504 -53.7322323
H 6.8322139 5.3057652 -54.3480851
C 4.8587987 4.7465541 -53.7745885
H 4.9169297 4.1923917 -54.7089597
C 3.516118 5.5057891 -53.8614048
O 2.5191161 5.1118456 -53.2532807
C 4.8607276 3.7088065 -52.6438668
H 4.6254463 4.2273965 -51.7263386
H 4.0580549 2.9937707 -52.8329263
C 6.1573545 2.9237375 -52.4338794
H 5.9667513 2.1123083 -51.7318169
H 6.9089625 3.5794581 -51.9982039
C 6.7097361 2.3381834 -53.7201912
O 7.8204849 2.6367907 -54.124086
N 5.9596121 1.5408023 -54.4434662
H 6.3860314 1.1601171 -55.2659575
H 4.9443976 1.5731702 -54.3602218
N 3.4922299 6.609794 -54.6151327
H 4.3734201 6.9183661 -54.9925454
C 2.3102518 7.4619524 -54.7883182
H 1.523502 7.1422596 -54.1042785
C 1.7272705 7.3825057 -56.209906
O 0.5213259 7.2247492 -56.3878293
C 2.7044591 8.890572 -54.3960494

H 3.4554001 9.2689719 -55.0839756
H 3.1257726 8.8895343 -53.3873539
S 1.267988 9.9956678 -54.455393
H 1.9581013 11.1116626 -54.1764562
N 2.5785643 7.3956426 -57.2408166
H 3.5667076 7.4519611 -57.0465178
C 2.1412431 7.4998195 -58.6453044
H 1.3706166 8.2684934 -58.6961616
C 1.455654 6.224998 -59.1639819
O 0.6846533 6.2812467 -60.1185169
C 3.3271499 7.9979964 -59.498981
H 4.2271993 7.4604443 -59.1935633
C 3.5120954 9.5030321 -59.2234801
H 2.7433385 10.070109 -59.7525158
H 3.3888159 9.7067547 -58.1618482
C 3.1360484 7.7830679 -61.0060698
H 2.1735433 8.1823495 -61.3276595
H 3.2063335 6.7193164 -61.2171017
H 3.9295564 8.2650958 -61.5743967
C 4.8911054 10.0097662 -59.6504475
H 5.0099305 9.9628237 -60.7314375
H 5.6735898 9.4236341 -59.1685517
H 4.9797716 11.0455733 -59.3443017
N 1.5717582 5.1041732 -58.4564899
H 2.1827045 5.1206104 -57.6529454
C 0.7328439 3.9149034 -58.651388
H 0.8947388 3.5462889 -59.6639284
C -0.7823536 4.1812482 -58.5331897
O -1.5480582 3.5308124 -59.2486143
C 1.1982313 2.8047712 -57.6992863
H 0.5310809 1.9524817 -57.8022436
H 2.1807305 2.4791663 -58.0396875
C 1.2976814 3.1948038 -56.2119626
H 0.3012642 3.1688227 -55.7657597
H 1.6944627 4.205053 -56.0979221
C 2.2384522 2.2254211 -55.4939914
O 3.4720441 2.4194285 -55.590334
O 1.7426194 1.2099946 -54.9540701
N -1.2002262 5.2068174 -57.7761104
H -0.5129244 5.7123035 -57.2254629
C -2.598939 5.6605511 -57.6700557
H -3.2650489 4.7962077 -57.7183515
C -3.0249164 6.6303356 -58.7861373
O -4.212663 6.7435325 -59.0897442
C -2.8084363 6.3407486 -56.3074555
H -3.8525083 6.6461592 -56.2269157

H -2.1951503 7.2411331 -56.2540386
C -2.494111 5.4648105 -55.1222584
N -3.2748369 4.3995001 -54.6813414
C -1.402258 5.5724542 -54.3123075
H -0.6083186 6.2963122 -54.4092074
C -2.6439835 3.8919381 -53.61534
H -2.9894624 3.0311948 -53.0587202
N -1.5076655 4.5702295 -53.3754836
H -0.8044458 4.3204002 -52.6953078
N -2.0794667 7.3391896 -59.4136958
H -1.1158875 7.1556197 -59.1719981
C -2.3616997 8.3287098 -60.4667779
H -3.2276859 8.9173499 -60.1602128
C -2.7464924 7.5875773 -61.7548098
O -2.001621 6.7304906 -62.2333305
C -1.169923 9.3091347 -60.6337605
H -0.2399088 8.7535995 -60.5132491
C -1.1908147 10.461597 -59.5993055
H -0.3002541 11.0742561 -59.749503
H -2.0610367 11.0934907 -59.7853356
C -1.1248122 9.968303 -62.0256172
H -2.0519598 10.5058078 -62.2273489
H -0.9656117 9.221869 -62.8030621
H -0.2929621 10.6711498 -62.0761009
C -1.2165483 10.0459058 -58.1225757
H -0.4028053 9.3553923 -57.9105932
H -2.1699243 9.5788426 -57.8756608
H -1.0975652 10.9321865 -57.4986943
N -3.9191623 7.9002296 -62.3131057
H -4.4943561 8.5906527 -61.8567087
C -4.4811881 7.1647627 -63.4544377
H -4.2521094 6.1048541 -63.3300885
C -3.8496852 7.5951677 -64.7889643
O -3.2727482 6.7640869 -65.4867586
C -6.0132403 7.3088003 -63.4796484
H -6.2694267 8.3537053 -63.6622205
H -6.4039576 6.7184002 -64.3096062
C -6.7166646 6.8608593 -62.1861027
H -7.7887787 7.0203155 -62.3018223
H -6.3872767 7.4812947 -61.3521355
C -6.4970237 5.3889815 -61.8459751
O -6.9995508 4.4963847 -62.5114945
N -5.7572628 5.0902401 -60.8042215
H -5.833909 4.14822 -60.4307188
H -5.3482376 5.8097848 -60.2156199
N -3.8569681 8.8981386 -65.0946658

H -4.3202539 9.5439063 -64.4785248
C -3.1401507 9.4583446 -66.2477274
H -3.0411782 8.6833109 -67.0107969
C -1.7236527 9.8698317 -65.8282051
O -1.4618108 10.9868417 -65.364135
C -3.9305454 10.5951731 -66.9132344
H -4.0322571 11.4261952 -66.2131145
H -4.9304066 10.2295566 -67.1538309
C -3.2659619 11.0967388 -68.2087631
O -2.0957811 10.7292587 -68.4770125
O -3.9030893 11.919111 -68.8986723
N -0.803794 8.9111941 -65.9612056
H -1.116004 8.0117478 -66.302686
C 0.6148988 9.1220086 -65.6716635
H 0.701243 9.6227611 -64.7065084
C 1.2656105 10.0741342 -66.6689175
O 2.098196 10.8648421 -66.2441968
C 1.3529267 7.7757301 -65.5926566
H 1.1126767 7.1689474 -66.4679944
H 2.4297305 7.955249 -65.5762918
C 0.9574017 7.0366377 -64.3062496
H 1.2856483 7.6316669 -63.4540904
H -0.1280695 6.9419732 -64.2625537
C 1.5717334 5.633152 -64.2128231
H 2.6407791 5.6991469 -64.4166036
H 1.1128656 4.9710833 -64.9484462
C 1.3819517 5.0770151 -62.7971826
H 1.7874912 5.8138986 -62.1066302
H 1.9538723 4.1524789 -62.6816544
N -0.0351696 4.8430507 -62.4374898
H -0.6226182 5.656999 -62.6128357
H -0.1159741 4.6848988 -61.4413045
H -0.410129 4.0257107 -62.9061462
N 0.857613 10.1083443 -67.9375912
H 0.0306158 9.5949737 -68.2147783
C 1.4627276 11.0419099 -68.8908006
H 2.5495178 10.9536548 -68.8456466
C 1.0955327 12.4971915 -68.5945729
O 1.9570158 13.368256 -68.6695282
C 1.0240044 10.7936445 -70.3246517
H -0.0383867 11.0268319 -70.4208665
H 1.5987591 11.5133366 -70.9013528
C 1.2870139 9.4334093 -70.9591885
H 0.6207275 8.6875652 -70.5204886
H 2.321262 9.1555211 -70.7495981
C 1.0670261 9.5267931 -72.4818624

O 1.5837226 8.6346019 -73.1863309
O 0.449561 10.5278113 -72.9382914
N -0.1651418 12.7909911 -68.2489103
H -0.8538143 12.0316837 -68.2624408
C -0.597046 14.1400191 -67.8500139
H -0.3606733 14.8368522 -68.6493023
C 0.1788132 14.6128788 -66.6331437
O 0.6875716 15.7306213 -66.6664212
C -2.1126775 14.1466946 -67.5941196
H -2.6227997 14.0571914 -68.5530931
H -2.3576696 13.272807 -66.9910062
C -2.6896842 15.3612165 -66.8459386
H -3.7652708 15.2087699 -66.7430743
H -2.2667664 15.3935762 -65.8416272
C -2.4521123 16.7168604 -67.5216241
H -2.901966 16.7154243 -68.5155877
H -1.3820194 16.9074539 -67.6060141
C -3.0951271 17.8042082 -66.6541248
H -2.7560098 17.6658543 -65.6223159
H -4.1813943 17.6762267 -66.6795216
N -2.71647 19.159312 -67.1134731
H -1.700728 19.2804625 -67.0277463
H -3.1123092 19.8764223 -66.5258363
H -2.9676407 19.3212056 -68.075571
N 0.3204954 13.766832 -65.6119406
H -0.1301998 12.8600258 -65.6626692
C 1.1372004 14.0988161 -64.4489276
H 0.7681291 15.0311553 -64.0186157
C 2.6010132 14.3488103 -64.8440332
O 3.1203401 15.4231067 -64.5515805
C 1.0048208 13.0052398 -63.3833035
H -0.0274297 12.9715737 -63.0338871
H 1.2416141 12.0362933 -63.8251743
C 1.9196494 13.2544928 -62.2026704
C 1.5902525 14.2330469 -61.2449909
H 0.6507624 14.7645696 -61.3091331
C 2.5054578 14.5589645 -60.2283914
H 2.2645 15.3361693 -59.5185997
C 3.7477584 13.9046379 -60.163313
H 4.4610224 14.1635952 -59.3939693
C 4.075634 12.9324819 -61.1222156
H 5.0420339 12.4538226 -61.0965527
C 3.1634757 12.6011592 -62.1355357
H 3.4384687 11.8742898 -62.8856969
N 3.2219607 13.4431795 -65.6129092
H 2.7236004 12.5935768 -65.8627977

C 4.6285742 13.5613066 -66.0272132
H 5.2190656 13.6913037 -65.136371
C 4.8554892 14.8103973 -66.9071792
O 5.7538651 15.5982238 -66.6248052
C 5.1469566 12.2423806 -66.6375441
H 4.5264298 12.0049949 -67.503892
C 6.6112232 12.3569321 -67.0848273
H 7.294004 12.1712733 -66.2581363
H 6.8394987 13.3368053 -67.4929083
H 6.78462 11.6171695 -67.8603109
C 5.0988351 11.0728698 -65.6156212
H 5.8917595 11.1882859 -64.8758281
H 4.1709873 11.0890031 -65.0529447
C 5.2309691 9.6913749 -66.2729081
H 6.195366 9.5950969 -66.7695518
H 4.4343643 9.5515472 -67.0036455
H 5.1519131 8.9179254 -65.5089451
N 3.9636351 15.1292163 -67.8520424
H 3.2265652 14.4600239 -68.0547949
C 4.0218868 16.3871815 -68.6226211
H 5.0042765 16.4596209 -69.0901891
C 3.8797531 17.6361744 -67.751329
O 4.3853571 18.6900604 -68.1393617
C 2.9423549 16.3991231 -69.7126516
H 1.9845357 16.1193492 -69.2705717
H 2.8543825 17.4158555 -70.0989016
C 3.2701847 15.4763868 -70.8965743
H 3.2834725 14.434007 -70.5792135
H 4.2600221 15.7274747 -71.2810722
C 2.2462379 15.6651832 -72.0260354
H 2.5661878 15.0769262 -72.8877034
H 2.2537104 16.7118842 -72.3344896
N 0.873631 15.2917612 -71.613203
H 0.3736673 15.9475456 -71.0440993
C 0.2606047 14.1451865 -71.8617678
N 0.8324717 13.2109762 -72.562001
H 0.4215544 12.2750905 -72.7048818
H 1.7817286 13.3170273 -72.8573687
N -0.9394563 13.9204432 -71.4047869
H -1.4254517 14.5628763 -70.815964
H -1.3235025 12.9947997 -71.5411287
N 3.1468849 17.5726836 -66.640555
H 2.7147858 16.6893069 -66.3908726
C 3.0422072 18.6861717 -65.6891141
H 2.9536692 19.6115987 -66.2544648
C 4.3172776 18.8331359 -64.8542892

O 4.818539 19.9522103 -64.7316767
C 1.7891423 18.5422215 -64.8007602
H 1.0555546 17.9044931 -65.2924164
H 2.0612306 18.0606817 -63.8602092
C 1.1195113 19.8957002 -64.5028273
H 1.8473703 20.5638183 -64.0369374
H 0.3048766 19.7346467 -63.7929669
C 0.5491297 20.5310302 -65.7790337
O -0.2282403 19.8629453 -66.4986209
O 0.9457575 21.6510811 -66.1609826
N 4.94132 17.725144 -64.4234338
H 4.4998686 16.8199543 -64.5638902
C 6.2140599 17.8019982 -63.6909489
H 6.0524835 18.5815138 -62.9562327
C 7.3890477 18.3481159 -64.518059
O 8.2732855 18.9822581 -63.9445966
C 6.5604161 16.5360761 -62.8697259
H 7.6036421 16.612572 -62.5826921
C 5.687129 16.5745079 -61.5928064
H 4.6438896 16.3754152 -61.8451636
H 5.7360653 17.5498829 -61.1123979
H 6.0256541 15.83006 -60.8757306
C 6.3611885 15.1622226 -63.5084856
H 5.298497 14.9474787 -63.5301493
H 6.74844 15.1843201 -64.5161972
C 7.0610066 14.0140301 -62.7739185
H 6.6684855 13.8981371 -61.7667884
H 8.122155 14.2216184 -62.7084251
H 6.9110273 13.0840706 -63.3228865
N 7.30301 18.2991724 -65.852446
H 6.594574 17.6978391 -66.2541671
C 8.2430857 18.9945263 -66.7521705
H 9.256973 18.7089679 -66.4899572
C 8.1690656 20.51075 -66.5818082
O 9.1990222 21.1611148 -66.460308
C 7.977772 18.6399987 -68.229651
H 6.9892592 19.0220523 -68.484297
C 8.9845327 19.2914653 -69.1874602
H 10.0014391 18.9979181 -68.9194354
H 8.9126701 20.3778295 -69.1491886
H 8.7886603 18.966429 -70.2109033
C 7.96589 17.1408008 -68.5243393
H 8.943109 16.8146886 -68.8779901
H 7.2357508 16.9438971 -69.3046957
H 7.7171778 16.551408 -67.6508645
N 6.9615765 21.0893303 -66.532496

H 6.1488449 20.486938 -66.5022801
C 6.780615 22.5474602 -66.4135413
H 7.4330732 23.0541204 -67.1286945
C 7.2202667 23.0717325 -65.0569848
O 7.912679 24.0815246 -64.9821131
C 5.3159302 22.9327156 -66.6760503
H 4.6831159 22.049465 -66.5844177
H 4.9780527 23.653042 -65.9286919
C 5.1585947 23.5769347 -68.0589042
H 5.5905341 22.9257093 -68.8152424
H 5.7275276 24.5057287 -68.0837541
C 3.6905468 23.8966561 -68.3952165
H 3.6380314 24.2920585 -69.4108383
H 3.3606373 24.6917945 -67.7218206
N 2.7656957 22.7454938 -68.2527494
H 2.0283473 22.845749 -67.5579178
C 2.88091 21.5274568 -68.7454514
N 3.8693124 21.140862 -69.4991559
H 4.0949378 20.1565639 -69.4957941
H 4.5709032 21.8214323 -69.7161739
N 1.9903342 20.6334323 -68.4586807
H 1.2955849 20.8980357 -67.7519289
H 2.1477213 19.6774308 -68.6872359
N 6.8385406 22.3850171 -63.9793491
H 6.2678414 21.5594362 -64.1188139
C 7.0762139 22.8882716 -62.6154744
H 6.7536171 23.9302454 -62.5989197
C 8.5529194 22.901266 -62.209056
O 8.8936518 23.5075295 -61.1960446
C 6.2302789 22.1490035 -61.5659052
H 6.3002834 22.7158685 -60.6375336
C 4.7406745 22.1370212 -61.9312552
H 4.5608851 21.5072413 -62.7992222
H 4.4104188 23.1524728 -62.155835
H 4.1599278 21.7513496 -61.0951958
C 6.7187884 20.740197 -61.2639461
H 6.946144 20.2365441 -62.1944734
H 5.9697426 20.1820863 -60.7022476
H 7.6299397 20.7966649 -60.6732389
N 9.4330305 22.2598839 -62.9727844
H 9.1190088 21.8290885 -63.831774
C 10.8586508 22.2157185 -62.6987607
H 11.0205601 22.3896924 -61.6385775
C 11.6587618 23.2613323 -63.4943908
O 11.250341 23.686733 -64.571033
C 11.3162717 20.8126336 -63.009183

H 11.2222436 20.673128 -64.0870845
H 10.6948542 20.0962774 -62.4706286
H 12.34619 20.6925253 -62.698318
N 12.8048385 23.6814376 -62.9622398
H 13.0708624 23.3246349 -62.0518115
C 13.7495271 24.5578181 -63.6509041
H 13.1778 25.2862108 -64.2238724
C 14.6719354 23.7794981 -64.6223379
O 14.975169 22.6149417 -64.3663321
C 14.5534374 25.3133525 -62.584314
H 15.1349368 24.6050227 -61.9900518
H 13.8784704 25.8645982 -61.9278724
H 15.2402197 26.0126728 -63.0612602
N 15.2562672 24.4346856 -65.6428965
C 16.3457623 23.8565942 -66.4329207
H 15.9645131 23.0084207 -66.9972777
C 17.5084646 23.3942187 -65.5437926
O 17.9666931 24.1420647 -64.6815619
C 16.7604415 24.9539896 -67.41675
H 17.5396302 25.5804047 -66.9808329
H 17.0916402 24.5356973 -68.368371
C 15.4800201 25.7739039 -67.5748403
H 14.8102338 25.2746548 -68.2775496
H 15.6876092 26.7949285 -67.8961341
C 14.8755827 25.7311526 -66.1737705
H 13.7917232 25.8368128 -66.2352599
H 15.3069372 26.5190763 -65.555338
N 17.9761557 22.1537945 -65.7184936
H 17.5578649 21.5750566 -66.4370437
C 18.9691881 21.5330127 -64.828799
H 19.4528712 20.7173447 -65.3653614
H 19.7268063 22.271958 -64.5676816
C 18.3945524 20.9579177 -63.5231969
O 19.1431088 20.4234825 -62.6929978
N 17.0763422 20.9995032 -63.325819
H 16.4871733 21.5126475 -63.9742723
C 16.4200515 20.282351 -62.2438022
H 16.8992307 20.5608404 -61.3042546
C 16.5430015 18.7673145 -62.4295642
O 16.5979498 18.2344241 -63.5433115
C 14.9511917 20.6876016 -62.1594518
H 14.4759675 20.4721613 -63.1160417
H 14.876111 21.7519627 -61.935609
H 14.4412873 20.1255796 -61.3775577
N 16.4551063 18.0541021 -61.3125826
H 16.2855776 18.5690471 -60.4511779

C 16.3207142 16.6066863 -61.2672556
H 16.667364 16.1436285 -62.1925943
C 14.8430527 16.4876811 -61.1714432
O 14.2703098 16.4918358 -60.0760996
C 17.1025975 16.0055349 -60.0967373
H 16.7267382 16.4254048 -59.1674263
H 16.9332383 14.927969 -60.0696969
C 18.6151246 16.2789271 -60.2035818
H 18.7934009 17.3000794 -60.5397025
H 19.0498173 15.6006874 -60.9384452
C 19.3309473 16.1098317 -58.8681021
O 19.8209138 17.063498 -58.2767724
N 19.4280626 14.9016131 -58.3528361
H 19.9160454 14.8206799 -57.47876
H 19.0269415 14.1074667 -58.8221429
N 14.2900884 16.7928196 -62.3621341
H 14.9082487 16.9578584 -63.1449686
C 12.8660165 16.8921841 -62.5786515
H 12.50313 17.8129391 -62.118628
C 12.3945187 16.8195691 -64.0583579
H 11.5182164 16.1806944 -64.1205188
C 11.927267 18.2080647 -64.4477732
H 12.7140434 18.9289267 -64.2293846
H 11.0102521 18.4103486 -63.8975376
H 11.6871596 18.2879955 -65.5028421
C 13.3973882 16.3601632 -65.1198995
H 13.1242213 16.743578 -66.1014841
H 14.3632553 16.7589904 -64.8585091
C 13.4802412 14.8541873 -65.2635705
H 12.5137973 14.4524802 -65.5470749
H 13.7913259 14.4259101 -64.3196536
H 14.1809111 14.6061911 -66.0551717
C 12.2832975 15.764129 -61.7971147
O 11.2494559 16.0014477 -61.1925983
N 12.9953431 14.6160479 -61.6922139
H 13.8487719 14.4634771 -62.2140222
C 12.4409562 13.5648977 -60.9104669
H 11.9661348 14.0429408 -60.056893
C 13.3950247 12.5287825 -60.3251963
O 14.3898634 12.1321749 -60.9337501
C 11.3641755 12.9163869 -61.7760491
H 11.69877 11.9263678 -62.0871113
C 10.1448767 12.7675339 -60.8818847
H 9.7797381 13.7482455 -60.5753726
H 10.389334 12.200937 -59.985975
H 9.3515675 12.2659959 -61.4236928

C 10.8142608 13.647437 -63.0167973
H 10.2651247 14.5505195 -62.7809551
H 10.1246692 12.9691349 -63.5158578
H 11.6027534 13.8396563 -63.7244018
N 12.992268 12.0624941 -59.1407394
H 12.2290494 12.5696896 -58.7005972
C 13.3517678 10.7991302 -58.4875107
H 13.8691189 10.1454328 -59.1901295
C 12.022295 10.1409558 -58.0722694
O 11.2124517 10.7801555 -57.3905926
C 14.2732964 11.0619765 -57.2714117
H 13.7556098 11.7467959 -56.5983282
C 14.5539489 9.7552096 -56.5040559
H 15.098085 9.0547942 -57.1393494
H 13.6255968 9.2720965 -56.1977078
H 15.1326487 9.9473315 -55.6028041
C 15.5967541 11.7351388 -57.7104154
H 16.1322448 11.0671286 -58.3834904
H 15.3704105 12.6510699 -58.2557262
C 16.5299331 12.1281646 -56.5575202
H 16.9353557 11.2398739 -56.0737671
H 15.9862613 12.7290053 -55.8275692
H 17.3598219 12.7148318 -56.9523203
N 11.7580335 8.9046503 -58.5018071
H 12.4971308 8.4049395 -59.0006782
C 10.4983895 8.1867901 -58.2471085
H 9.9675434 8.6814518 -57.4396159
C 10.7986262 6.7492106 -57.8252037
O 11.102295 5.9443846 -58.7003625
C 9.5576584 8.1569015 -59.488233
H 10.0657489 7.6118162 -60.2850846
C 9.2280089 9.5527545 -60.0531933
H 8.7489912 10.1650888 -59.2892799
H 10.1684049 10.018405 -60.3410172
C 8.2538598 7.3944316 -59.1507915
H 7.707404 7.913199 -58.3643917
H 8.4738782 6.3822778 -58.8197286
H 7.6197814 7.2880813 -60.0276344
C 8.3419087 9.5094014 -61.311817
H 7.3388524 9.1658198 -61.0701747
H 8.7868381 8.8466241 -62.055071
H 8.2449684 10.4990386 -61.7479271
N 10.3778145 6.3647875 -56.6173089
H 10.0867171 7.0875804 -55.969359
C 10.0771669 4.965996 -56.2829504
H 10.6161371 4.3342597 -56.978693

C 8.5735844 4.707958 -56.4325046
O 7.7547345 5.5273144 -56.0055968
C 10.6004787 4.5910992 -54.8968489
H 10.1061673 5.1945444 -54.1366728
H 11.6735419 4.7889482 -54.8607758
O 10.3828632 3.2140938 -54.6418497
H 9.4472056 3.0366627 -54.4338089
N 8.1607001 3.6309068 -57.1055167
H 8.8769937 2.9804449 -57.4273788
C 6.7371884 3.300688 -57.3620578
H 6.1596131 3.5083897 -56.4621738
C 6.5491433 1.8064488 -57.6597888
O 7.4787363 1.1227106 -58.0834072
C 6.1822375 4.201798 -58.4902398
H 6.9381585 4.3099942 -59.2694911
C 4.8723056 3.766805 -59.1432156
H 4.1171477 3.6139869 -58.3752925
H 5.0235349 2.8460121 -59.7068042
H 4.5357146 4.5394803 -59.8340945
O 5.8902408 5.4700017 -57.9372644
H 6.6083844 5.6589023 -57.3062582
N 5.3715908 1.2506011 -57.3572631
H 4.6553821 1.8125038 -56.8983762
C 5.0533527 -0.1448405 -57.6414244
H 5.9175855 -0.7539901 -57.3692877
C 4.7686066 -0.3651727 -59.1348527
O 3.9382459 0.3149866 -59.7426248
C 3.8790949 -0.6020956 -56.7719286
H 2.9852171 -0.0491227 -57.0590648
H 4.0992285 -0.4156114 -55.7198245
H 3.7121333 -1.6687299 -56.9224242
N 5.384449 -1.4132231 -59.6710028
H 5.9615485 -1.978817 -59.0585404
C 5.274073 -1.8707675 -61.0481607
H 4.52063 -1.2819514 -61.5709594
C 4.819635 -3.3368143 -61.111532
O 4.71484 -4.0327033 -60.0985856
C 6.6290324 -1.6468094 -61.7413333
H 6.7247861 -2.2962304 -62.6122492
H 7.4480343 -1.8571985 -61.0513851
S 6.7161264 0.0744788 -62.2988248
H 5.7073454 -0.0156779 -63.1767116
N 4.5804398 -3.8163232 -62.3306187
H 4.6770828 -3.1691214 -63.1064769
C 4.346574 -5.2294842 -62.6551505
H 4.459447 -5.8264837 -61.7510928

C 5.4032849 -5.7429896 -63.6329606
O 6.1897972 -4.9572822 -64.1649364
C 2.9059161 -5.4309908 -63.1662228
H 2.7672556 -6.4849372 -63.4143421
H 2.2061259 -5.1920836 -62.3647088
C 2.5629917 -4.5759187 -64.3962885
H 3.3538419 -4.6507815 -65.1381943
H 2.4774119 -3.5317996 -64.1082129
C 1.239358 -5.0199314 -65.0200982
H 0.4409992 -4.8935195 -64.2851521
H 1.2911458 -6.0804497 -65.2787751
N 0.9258565 -4.2126199 -66.212061
H 0.4081314 -3.3590145 -66.0351975
C 1.2259418 -4.5210722 -67.4631425
N 1.9688723 -5.5468659 -67.7776155
H 2.1152903 -5.8721481 -68.7219343
H 2.3570204 -6.1389378 -67.0480665
N 0.7801215 -3.779182 -68.4391839
H 0.1596895 -3.0162654 -68.2328983
H 0.9754474 -4.0537471 -69.3841574
N 5.3899017 -7.0384504 -63.929528
H 4.7383203 -7.6344439 -63.4363782
C 6.0942102 -7.5596355 -65.1036889
H 7.0977374 -7.1300138 -65.1006469
C 5.4232967 -7.0925274 -66.4226341
O 4.3354509 -6.5106139 -66.4170726
C 6.2677109 -9.0838745 -64.9594063
H 6.6936781 -9.2945219 -63.9792089
H 6.9785677 -9.4402086 -65.7029615
C 4.9912565 -9.885612 -65.1365704
O 4.2025192 -9.6370408 -66.0222303
N 4.7808317 -10.9165378 -64.3629691
H 3.901636 -11.390675 -64.4666077
H 5.4341878 -11.1196392 -63.6117621
N 6.087026 -7.301176 -67.5654737
H 6.97492 -7.7768733 -67.5279906
C 5.5686416 -6.8960675 -68.8824775
H 5.3268371 -5.8354456 -68.847767
C 4.2717675 -7.636028 -69.2757134
O 3.3960169 -7.0361793 -69.9063768
C 6.6966829 -7.1057223 -69.9118019
H 7.5465829 -6.4850685 -69.622889
H 7.0144693 -8.1489414 -69.8602459
C 6.3428838 -6.7968035 -71.3816833
H 5.5274226 -7.4439008 -71.7033055
C 5.9359276 -5.3387014 -71.5982807

H 6.7312629 -4.6712647 -71.263949
H 5.0185384 -5.1252485 -71.0539862
H 5.7487945 -5.1681951 -72.6580208
C 7.5553389 -7.0841982 -72.2684394
H 8.3866897 -6.4341584 -71.9947723
H 7.2949337 -6.9136733 -73.313153
H 7.8578121 -8.1251123 -72.1530906
N 4.1334798 -8.8979656 -68.8636108
H 4.8250579 -9.2749777 -68.2335885
C 3.0282568 -9.7990911 -69.2066128
H 2.994302 -9.8630138 -70.2915907
C 1.6602333 -9.3119172 -68.6927965
O 1.5945623 -8.516802 -67.7502102
C 3.3072741 -11.2092038 -68.6610331
H 2.5364612 -11.8929918 -69.0166306
H 3.2739869 -11.2045757 -67.5732222
O 4.5717364 -11.6737482 -69.1032695
H 4.7435367 -12.5260357 -68.6915725
N 0.5433211 -9.7783737 -69.2905212
C -0.7985269 -9.4881579 -68.7920022
H -0.974344 -8.4156383 -68.878059
C -0.9754439 -9.9212677 -67.3331253
O -0.527133 -10.9988821 -66.9390965
C -1.7659872 -10.2366409 -69.7171588
H -1.976375 -11.2318292 -69.3196156
H -2.693753 -9.6827823 -69.8650191
C 0.4554123 -10.5666366 -70.5124142
H 1.1583618 -10.2258453 -71.2721388
H 0.6242898 -11.6193873 -70.280253
C -0.973233 -10.3730318 -71.0152853
H -1.0393598 -9.4455678 -71.586019
H -1.3115491 -11.2200922 -71.6130074
N -1.6717958 -9.0991233 -66.547554
H -1.9774122 -8.2113845 -66.9104634
C -1.9426282 -9.3712957 -65.1363939
H -0.9869589 -9.4906116 -64.6280691
C -2.7599268 -10.6578647 -64.9656591
O -3.8400503 -10.7898855 -65.5414905
C -2.701309 -8.1901542 -64.5041285
H -2.7718945 -8.3380187 -63.4261289
H -3.7101012 -8.1606276 -64.9151862
O -2.0772418 -6.945159 -64.7763668
H -2.4140318 -6.2669389 -64.1668781
N -2.292544 -11.5662814 -64.1043462
H -1.3627723 -11.44079 -63.7365719
C -3.0207712 -12.7921241 -63.7457168

H -3.2244875 -13.323451 -64.6781217
C -4.3966937 -12.4684447 -63.1343671
O -4.6230048 -11.3825377 -62.579092
C -2.1096583 -13.72106 -62.9038127
H -1.910148 -14.6234283 -63.4834728
H -1.1418491 -13.2284864 -62.8004951
C -2.5148567 -14.1252411 -61.4840284
H -2.7946617 -13.2267165 -60.9280213
H -1.6416138 -14.553518 -60.9864657
C -3.6505368 -15.1492933 -61.4302847
O -4.2452488 -15.22482 -60.332582
O -4.0275463 -15.7087837 -62.4791995
N -5.3275999 -13.4242844 -63.2208885
H -5.0081732 -14.3620026 -63.4746751
C -6.7373853 -13.2558538 -62.8708644
H -7.1275333 -12.4809128 -63.5271843
C -6.946757 -12.7679238 -61.4410492
O -7.9117529 -12.0322895 -61.2615722
C -7.4812086 -14.5788163 -63.1367407
H -7.2969487 -14.8900304 -64.1668939
H -7.0568127 -15.3459934 -62.4846586
C -8.9995942 -14.5392484 -62.9048429
H -9.3885239 -15.5543231 -62.9959171
H -9.1947796 -14.2219061 -61.8805407
C -9.7553918 -13.6445641 -63.9063237
H -10.1326223 -14.2766743 -64.7127237
H -9.0952164 -12.9031723 -64.3565096
C -10.9343519 -12.935928 -63.2296966
H -11.4774207 -13.6713244 -62.6279936
H -11.6147183 -12.563259 -64.0023007
N -10.4659423 -11.8111498 -62.3804404
H -9.7675482 -12.1340372 -61.710333
H -11.2343898 -11.3862407 -61.8810379
H -10.0067443 -11.1094506 -62.9439105
N -6.0816248 -13.0886981 -60.4718269
H -5.3969747 -13.8110332 -60.6825751
C -6.0968226 -12.5397581 -59.1017552
H -7.1172031 -12.2357542 -58.8791371
C -5.2212816 -11.2922417 -58.8815129
O -5.4720267 -10.5585664 -57.9262851
C -5.7549487 -13.6167123 -58.0640669
H -6.3952429 -14.4869167 -58.2181221
H -5.9482444 -13.2182487 -57.0671658
O -4.3992676 -14.0098058 -58.1432093
H -4.343102 -14.5866227 -58.960205
N -4.2784466 -10.9636136 -59.7796263

H -4.191226 -11.5356199 -60.6116894
C -3.5645761 -9.675878 -59.7459851
H -3.2982635 -9.4612025 -58.710529
C -4.4764878 -8.5391038 -60.2170946
O -4.6424323 -7.5619692 -59.492832
C -2.2637066 -9.7208699 -60.5782299
H -1.5860132 -10.4576694 -60.1459091
H -2.5060962 -10.0411425 -61.5913691
C -1.54592 -8.348949 -60.6594275
H -2.2252017 -7.6004366 -61.0640231
C -1.049715 -7.869789 -59.2926931
H -0.3925492 -8.6162577 -58.8481829
H -1.895953 -7.6778135 -58.6345231
H -0.5004847 -6.9352805 -59.4146506
C -0.3461376 -8.4080241 -61.5969519
H 0.3865714 -9.1103831 -61.226032
H 0.1108929 -7.4212003 -61.6794127
H -0.6523785 -8.7230191 -62.5882788
N -5.1169134 -8.689943 -61.3849318
H -4.9457027 -9.5459942 -61.90868
C -6.0412299 -7.6860189 -61.9416269
H -5.4551489 -6.7857532 -62.1437637
C -7.1240799 -7.231914 -60.9375162
O -7.2297424 -6.0346225 -60.7273625
C -6.5934143 -8.1928127 -63.2907024
H -5.7661369 -8.2380254 -63.9984981
H -6.9541082 -9.2147834 -63.16253
C -7.7329938 -7.3712305 -63.9310273
H -7.8971692 -7.7500898 -64.9408023
H -8.6442331 -7.5659483 -63.3636415
C -7.5575872 -5.8442729 -64.0094301
H -7.4980538 -5.4352241 -63.0077227
H -8.4615509 -5.4163366 -64.4482493
C -6.3614685 -5.3390559 -64.8205827
H -6.662531 -5.2804452 -65.8695286
H -5.5195257 -6.0309988 -64.7300999
N -5.9571078 -4.0034871 -64.3263948
H -6.7679802 -3.395136 -64.17414
H -5.2630821 -3.5587778 -64.9038722
H -5.4837897 -4.0957187 -63.4295262
N -7.871645 -8.0931999 -60.2239082
C -8.873936 -7.6616363 -59.2573345
H -9.5234761 -6.9139405 -59.7159941
C -8.2649088 -7.0320422 -57.9992117
O -8.9337415 -6.198365 -57.3998078
C -9.7099499 -8.8997193 -58.9176722

H -10.0869565 -8.8805224 -57.8938592
H -10.5345133 -8.9914458 -59.6264198
C -7.9351771 -9.5147877 -60.3439147
H -8.4774898 -9.7379089 -61.262144
H -6.9490279 -9.9651637 -60.3563997
C -8.720291 -10.0246495 -59.1344312
H -9.2281338 -10.9685741 -59.3297095
H -8.0659944 -10.10908 -58.2666461
N -7.021786 -7.3606245 -57.6043131
H -6.4998668 -8.0322734 -58.1535313
C -6.3056253 -6.6353359 -56.5348897
H -6.9728801 -6.5111878 -55.6824392
C -5.9322881 -5.2270172 -57.0036012
O -6.1602659 -4.2700824 -56.2681912
C -5.0687729 -7.4390979 -56.0860549
H -5.3895788 -8.424192 -55.7418211
H -4.4305065 -7.5948128 -56.9556649
C -4.1992388 -6.7757217 -54.993123
H -3.2115868 -7.2341931 -55.0481208
H -4.0668317 -5.7140503 -55.2046993
C -4.7169933 -6.9550987 -53.5545555
H -4.8133604 -8.0239603 -53.351868
H -5.6999526 -6.4874112 -53.4551922
C -3.7314371 -6.3211879 -52.5553124
H -3.9467867 -5.2535484 -52.4822222
H -2.7197203 -6.4281609 -52.9491867
N -3.7880118 -6.9492504 -51.2117734
H -4.7008678 -6.8646728 -50.7950431
H -3.1294465 -6.5095671 -50.5629086
H -3.5314121 -7.9249485 -51.2652042
N -5.4101965 -5.0928218 -58.2204721
H -5.2513331 -5.9347831 -58.7634563
C -5.1139113 -3.8079675 -58.8626221
H -4.4172266 -3.2441876 -58.2414818
C -6.3827766 -2.9486339 -58.9977917
O -6.424878 -1.8461333 -58.4601641
C -4.4306352 -4.0925634 -60.2147685
H -3.4889227 -4.612916 -60.0251308
H -5.0681263 -4.750292 -60.8032861
C -4.1402352 -2.8329003 -61.0402102
H -3.5223312 -2.1630568 -60.4391233
H -5.0881717 -2.3339607 -61.2560169
C -3.4537445 -3.1310734 -62.3813936
O -3.6879035 -4.2298107 -62.9487496
O -2.7668995 -2.224337 -62.9038172
N -7.4664462 -3.5002571 -59.5499567

H -7.3734285 -4.4240226 -59.9575726
C -8.7116426 -2.7639545 -59.7964255
H -8.4353106 -1.8095062 -60.2480485
C -9.4638687 -2.4089424 -58.5074308
O -9.9528278 -1.288221 -58.3755535
C -9.6118555 -3.5466591 -60.7821039
H -9.0607949 -4.3693492 -61.2330581
H -10.4668038 -3.9705644 -60.2532929
C -10.1111674 -2.6392604 -61.9230753
H -10.7214885 -1.8416841 -61.4932013
H -10.7558622 -3.2309231 -62.5768496
C -8.9667387 -2.0313695 -62.7636765
O -8.2371866 -2.7994259 -63.4391515
O -8.8066446 -0.7847794 -62.7441526
N -9.47042 -3.2962327 -57.4999447
H -9.0417097 -4.20374 -57.6524768
C -10.1010431 -3.0170332 -56.1973756
H -11.0288266 -2.4764864 -56.3973745
C -9.2769901 -2.0761537 -55.3142795
O -9.8498703 -1.4157787 -54.4554675
C -10.4660358 -4.334596 -55.4944689
H -11.0058283 -4.9560219 -56.2112972
H -9.5566922 -4.8544857 -55.1890845
C -11.3704398 -4.1053174 -54.2696322
H -10.7558442 -3.807233 -53.4188719
H -12.0692675 -3.2953097 -54.4855386
C -12.2007088 -5.3376816 -53.8874421
H -12.8839548 -5.5640901 -54.7082912
H -12.7938671 -5.0865768 -53.0059178
C -11.3301939 -6.5636396 -53.5898349
H -10.6293076 -6.3072624 -52.7892185
H -10.7480368 -6.8020651 -54.4859351
N -12.1634946 -7.726805 -53.1936491
H -12.701381 -7.5095402 -52.3633894
H -11.5843428 -8.534113 -52.9993255
H -12.8073944 -7.9650457 -53.9371857
N -7.9716141 -1.9444161 -55.5661454
H -7.5758423 -2.5040646 -56.3110106
C -7.1488087 -0.8533673 -55.0040867
H -7.6222074 -0.5067725 -54.0856447
C -7.1064176 0.3871534 -55.9041949
O -6.4532318 1.363562 -55.5514579
C -5.7327836 -1.3132419 -54.6059369
H -5.1704231 -0.4493004 -54.2512706
C -5.7686133 -2.3392788 -53.4714731
H -6.3200593 -3.2274988 -53.7777969

H -6.2540956 -1.8980821 -52.6008782
H -4.7497511 -2.6174525 -53.2046511
O -5.0121386 -1.9015539 -55.6648057
H -5.5081724 -2.690785 -55.9458878
N -7.8166632 0.3654646 -57.0417343
H -8.3462446 -0.4726061 -57.2427193
C -7.8502309 1.3899778 -58.0923619
H -8.3461036 0.901136 -58.9292334
C -6.4594769 1.7892235 -58.633805
O -6.3001095 2.8344891 -59.2640218
C -8.7526115 2.5574542 -57.6322694
H -9.6187161 2.1305136 -57.1235023
H -8.1999693 3.1478449 -56.8997247
C -9.2803135 3.5032468 -58.7320415
H -8.4579137 4.0436594 -59.1952806
C -10.0659324 2.771923 -59.8264709
H -10.8596408 2.1741863 -59.3761475
H -9.4021933 2.1159166 -60.3858728
H -10.5024011 3.4948423 -60.5147045
C -10.2132689 4.5376623 -58.0986059
H -11.0746174 4.0426197 -57.649767
H -10.5543428 5.2422816 -58.85601
H -9.6730952 5.0878871 -57.3275553
N -5.4426723 0.9434196 -58.4481881
H -5.6678236 0.0355238 -58.0548346
C -4.0798935 1.185912 -58.9357536
H -3.8150141 2.2211407 -58.7113507
C -4.0051982 1.032849 -60.4518383
O -4.7678273 0.27695 -61.0434649
C -3.0949649 0.2568287 -58.2130025
H -3.5138127 -0.7490093 -58.1422002
H -2.1678002 0.2159683 -58.784929
C -2.795052 0.8141 -56.8097441
H -2.3119699 1.7866437 -56.9208895
H -3.7301621 0.9656837 -56.2690902
C -1.8893985 -0.0773955 -55.9502131
H -2.4545609 -0.9480272 -55.6154601
H -1.5950424 0.5033683 -55.0736083
C -0.6439782 -0.5463608 -56.7108241
H -0.3018319 0.268945 -57.3493463
H -0.9215663 -1.3830817 -57.3566641
N 0.4467047 -0.9339892 -55.7916971
H 0.7465728 -0.112789 -55.2498367
H 1.2748543 -1.2105249 -56.2979961
H 0.1611309 -1.6486113 -55.1440047
N -3.0487572 1.7262357 -61.0774219

H -2.4915023 2.3509615 -60.5015827
C -2.7384924 1.6213578 -62.5167704
H -3.2764944 0.767828 -62.9341887
C -1.2710996 1.2723612 -62.7035448
O -0.4066791 2.1504201 -62.6343866
C -3.1835379 2.9095297 -63.2545982
H -3.4195988 3.6894009 -62.5300726
H -2.3764105 3.2816492 -63.8842477
C -4.4157079 2.6815394 -64.1627795
H -4.7723658 3.6569256 -64.4963477
H -4.1117891 2.1108096 -65.0407656
C -5.5713926 1.9356428 -63.4710507
H -5.670715 2.3288555 -62.460506
H -5.3424209 0.8725846 -63.4125411
C -6.9189723 2.0674559 -64.1837241
H -6.9312356 1.427326 -65.0694601
H -7.0421595 3.108668 -64.4916204
N -8.0097774 1.7033435 -63.2510456
H -7.9605382 0.719054 -62.960874
H -8.919042 1.7965361 -63.6756161
H -7.9574248 2.2846703 -62.4272009
N -0.9851324 -0.0152724 -62.8685472
H -1.7507671 -0.6921228 -62.9343551
C 0.3622951 -0.5630426 -62.7563147
H 0.9805693 0.166144 -62.2307181
C 0.9623679 -0.7635467 -64.1569443
O 0.4536773 -1.5034089 -65.0035394
C 0.3662204 -1.8384641 -61.8795023
H -0.0630003 -2.6610733 -62.4548948
C 1.819163 -2.1735927 -61.5191769
H 2.2062048 -1.4454116 -60.8056847
H 2.4410637 -2.152216 -62.4072739
H 1.8767011 -3.1699076 -61.08193
C -0.4305112 -1.6738873 -60.5577608
H 0.0351023 -0.9039866 -59.9410057
H -1.442414 -1.3440835 -60.7786126
C -0.5557201 -2.9669112 -59.7395202
H 0.4082644 -3.2467547 -59.3150915
H -0.9233073 -3.7726035 -60.3764866
H -1.265722 -2.8133331 -58.927048
N 2.0820627 -0.0851478 -64.4029156
H 2.4655832 0.475942 -63.6575331
C 2.9068083 -0.2770199 -65.5962123
H 2.3018658 -0.6952979 -66.4020926
C 4.0396154 -1.2643317 -65.292497
O 4.4307098 -1.4269569 -64.1332412

C 3.4495725 1.0814523 -66.061202
H 4.0958151 0.9525892 -66.9322466
H 4.0432311 1.5292122 -65.261646
S 2.0709612 2.1846743 -66.4876026
H 1.3003227 1.8921115 -65.4342484
N 4.6448883 -1.8495044 -66.3268338
H 4.3466771 -1.6176306 -67.261478
C 5.9932657 -2.3856146 -66.1595621
H 6.0491473 -2.8706147 -65.1886549
C 7.0088041 -1.2347704 -66.1249314
O 6.737833 -0.132612 -66.6086652
C 6.3073177 -3.4524623 -67.2163512
H 5.4957053 -4.1766962 -67.2517385
H 7.2191217 -3.9815768 -66.9375472
C 6.5230252 -2.8364934 -68.5773457
O 7.5678347 -2.280934 -68.8635157
N 5.5086591 -2.8222407 -69.403895
H 5.6807276 -2.3748843 -70.3032807
H 4.6772054 -3.3460722 -69.2191437
N 8.1801732 -1.5131329 -65.5618081
H 8.3470506 -2.4508453 -65.2320084
C 9.2452561 -0.5331003 -65.3748175
H 8.8589516 0.286887 -64.7647094
C 9.6957767 0.098359 -66.7072817
O 9.7789872 1.3222783 -66.8014925
C 10.3873618 -1.2029495 -64.5780293
H 11.2574185 -0.549599 -64.5671677
H 10.0590078 -1.2929866 -63.5414857
C 10.8177317 -2.5865022 -65.0490904
C 11.9181375 -2.7365552 -65.9169673
H 12.4819757 -1.8755661 -66.2390297
C 12.3053206 -4.0136478 -66.363435
H 13.1452796 -4.1187607 -67.0335377
C 11.597087 -5.1547648 -65.9319127
O 11.9592972 -6.3881262 -66.3699191
H 12.7274721 -6.3595775 -66.9421564
C 10.5129368 -5.0118722 -65.040923
H 9.9908157 -5.8930459 -64.6965091
C 10.1282528 -3.7322316 -64.5973171
H 9.308215 -3.6379075 -63.8983316
N 9.914116 -0.6953223 -67.7660937
H 9.7185 -1.6813804 -67.6780474
C 10.4258322 -0.1969978 -69.0526211
H 11.3531195 0.3334907 -68.8684677
C 9.4744436 0.8147866 -69.7068052
O 9.9050969 1.903903 -70.0918449

C 10.7555701 -1.3866897 -69.9704476
H 11.4799712 -2.0264374 -69.4643361
H 9.8468928 -1.968167 -70.1290859
C 11.319076 -0.9912917 -71.3499645
H 10.5886703 -0.3825456 -71.8825983
C 12.633967 -0.2142643 -71.2488822
H 13.3625122 -0.779567 -70.6682336
H 12.462991 0.7532524 -70.7772734
H 13.0319279 -0.0350601 -72.2476385
C 11.5735498 -2.2547509 -72.1719436
H 12.3160219 -2.8816896 -71.6783682
H 11.9344282 -1.9816748 -73.1636924
H 10.6437644 -2.8131246 -72.2827093
N 8.1721243 0.5219023 -69.7300654
H 7.8781855 -0.3931025 -69.4008782
C 7.1627271 1.4694778 -70.216607
H 7.465206 1.8258281 -71.2019721
C 7.0485684 2.7218684 -69.344085
O 6.7977963 3.7974389 -69.8845881
C 5.8037654 0.7721156 -70.3591403
H 5.0142317 1.5249066 -70.3314356
H 5.6424419 0.0910384 -69.5229777
C 5.6722935 0.0300481 -71.6647418
N 5.9532087 -1.3209948 -71.8725283
C 5.7499517 -1.545272 -73.178947
H 5.897551 -2.4993019 -73.6695688
N 5.3500598 -0.4176791 -73.790252
H 5.1471925 -0.3273254 -74.7758818
C 5.2980715 0.5887433 -72.851667
H 5.0341347 1.6258243 -73.0140616
N 7.3164396 2.6332608 -68.0360767
H 7.5599259 1.7314697 -67.6420576
C 7.2614763 3.7968527 -67.1509479
H 6.3620564 4.3393293 -67.4291629
C 8.4261241 4.7738874 -67.3880553
O 8.1695259 5.9696373 -67.5199989
C 7.0948289 3.3363918 -65.6891448
H 6.3288467 2.5601209 -65.6683109
H 8.0271337 2.8928056 -65.3390609
C 6.6681347 4.4549309 -64.7117129
H 7.5189438 5.109165 -64.5258907
C 5.4997377 5.3156355 -65.2074584
H 4.6757884 4.679795 -65.5306189
H 5.8301918 5.9439793 -66.0348046
H 5.1677863 5.9791257 -64.4099764
C 6.2145039 3.8387536 -63.3851094

H 5.3016056 3.2618588 -63.5260214
H 6.0445249 4.6237662 -62.6487135
H 6.9882306 3.1722436 -63.0041928
N 9.6480168 4.2850584 -67.6533181
H 9.7997559 3.2846604 -67.5630426
C 10.7262591 5.1507473 -68.1801541
H 10.8557976 5.9933419 -67.499146
C 10.3952471 5.6900104 -69.5631661
O 10.7418516 6.824877 -69.8713061
C 12.0605472 4.4240518 -68.3016877
H 12.1166467 3.8610945 -69.2350101
H 12.121121 3.7293563 -67.4803064
O 13.1491082 5.3247477 -68.1948499
H 13.269569 5.8510611 -69.0056855
N 9.6793356 4.9112594 -70.3797769
H 9.4739327 3.9631262 -70.084374
C 9.1529553 5.3651188 -71.6612905
H 9.9800387 5.6169965 -72.3253587
H 8.5721268 4.5597261 -72.1096179
C 8.2436502 6.5889815 -71.5206075
O 8.4964877 7.5881455 -72.1829673
N 7.271716 6.5802667 -70.6017955
H 7.1008963 5.7179054 -70.0934582
C 6.3753098 7.7248641 -70.3564864
H 5.913338 8.0175862 -71.2986071
C 7.1064366 8.967594 -69.8279953
O 6.7901523 10.0960769 -70.2162708
C 5.2706065 7.3106618 -69.3736671
H 5.7289851 6.9026424 -68.4718638
H 4.7203521 8.2063148 -69.0832355
C 4.2527546 6.3259864 -69.9274463
C 3.9569052 5.1377987 -69.2312677
H 4.470828 4.90886 -68.3126257
C 2.9868958 4.2463839 -69.7247382
H 2.7656118 3.3367646 -69.1875547
C 2.2932897 4.5473782 -70.9100298
H 1.5351606 3.8746796 -71.2849157
C 2.5749518 5.7375725 -71.6014384
H 2.0297721 5.995317 -72.5016579
C 3.5527959 6.6201892 -71.1146242
H 3.7383022 7.5375435 -71.6559592
N 8.1333138 8.7500029 -69.0098282
H 8.2811018 7.8069615 -68.6678179
C 9.07562 9.7716512 -68.5730746
H 8.5547353 10.5922679 -68.0912188
C 9.8605926 10.3853852 -69.7513712

O 9.7566054 11.5847949 -70.0065046
C 9.9597189 9.1076336 -67.5219712
H 10.8339721 9.7058895 -67.3759088
H 10.2895408 8.1279684 -67.8552867
S 9.0625811 8.9471298 -65.9503501
H 9.8351265 9.8474364 -65.3019252
N 10.4805271 9.5504548 -70.5898632
H 10.5030738 8.5688311 -70.3340113
C 11.2121939 9.9579073 -71.8004392
H 12.0155281 10.6371849 -71.5108146
C 10.3189662 10.6842638 -72.8220458
O 10.6706195 11.7496569 -73.3218699
C 11.8287769 8.7012708 -72.4308687
H 11.0787146 8.1829872 -73.0301747
H 12.1581597 8.0264929 -71.641054
O 12.9535704 9.016732 -73.2287619
H 13.2151123 8.2260534 -73.7115009
N 9.0917743 10.1931924 -73.0341901
H 8.8462209 9.3288767 -72.56349
C 8.0758771 10.7997598 -73.908273
H 8.5230395 10.9933282 -74.8827677
C 7.5601152 12.1572996 -73.4059811
O 6.9709603 12.9057875 -74.1813275
C 6.8980951 9.8177845 -74.0665242
H 6.5963806 9.4797408 -73.0746319
H 6.0469815 10.3468555 -74.4990079
C 7.2010356 8.596359 -74.9559661
H 8.1359891 8.1311667 -74.6495739
C 6.078985 7.5667695 -74.8172612
H 5.1265064 8.0041828 -75.1217947
H 6.002105 7.244648 -73.7790986
H 6.2918725 6.6974123 -75.4373065
C 7.3195826 8.9722824 -76.4354788
H 6.4050387 9.4644876 -76.7684964
H 7.4802548 8.0744735 -77.031481
H 8.1643764 9.6422728 -76.586479
N 7.8083199 12.5056056 -72.1428918
H 8.3267403 11.8644839 -71.5540116
C 7.4700463 13.8201725 -71.5844624
H 6.6249218 14.2248713 -72.140381
C 8.5965015 14.8537852 -71.7283394
O 8.4412966 15.9715581 -71.2569983
C 7.0096966 13.6702722 -70.1392581
H 6.7278152 14.646009 -69.7435907
H 7.8241777 13.2648047 -69.5378654
O 5.8818799 12.8084382 -70.0899695

H 6.1934362 11.8939971 -70.2199382
N 9.6527789 14.5194905 -72.4726668
H 9.6609405 13.583641 -72.8564542
C 10.8450282 15.3307806 -72.7823003
H 11.3215018 14.7681748 -73.5857787
C 11.9223128 15.3449434 -71.6844862
O 12.7208264 16.2703293 -71.5460031
C 10.4885248 16.6983789 -73.4123986
H 10.3052822 17.4216129 -72.6154718
H 9.5630553 16.5886969 -73.9810148
C 11.5483248 17.2610639 -74.3780375
O 12.3958398 16.4764423 -74.8659835
O 11.4371665 18.4657644 -74.7049311
N 11.9834183 14.26542 -70.8998863
H 11.310854 13.5258562 -71.0438426
C 13.0028759 14.0912496 -69.8714545
H 13.306885 15.0728525 -69.5156226
C 14.2829326 13.4339092 -70.3807713
O 14.2769208 12.3465484 -70.963321
C 12.4106606 13.3349922 -68.6923727
H 12.0504773 12.3665639 -69.0383334
H 13.215576 13.1470367 -67.9823596
C 11.3089208 14.0511588 -67.9372688
C 10.4143586 13.2895275 -67.1652494
H 10.4207411 12.2129036 -67.2452246
C 9.622269 13.9020251 -66.183757
H 9.0420531 13.2912381 -65.5123696
C 9.6896914 15.2939169 -66.0117228
O 9.0688287 15.8810816 -64.9659394
H 8.6517885 15.2197967 -64.4135997
C 10.476267 16.0688853 -66.8777947
H 10.5008894 17.1334103 -66.7732363
C 11.2869235 15.4568867 -67.8389113
H 11.9309688 16.084664 -68.4366982
N 15.413457 14.0666489 -70.0648519
H 15.3470006 14.8954175 -69.4865486
C 16.7466287 13.6072225 -70.4479379
H 16.6439033 13.0148765 -71.3578561
C 17.3845581 12.7089696 -69.3861672
O 17.0498046 12.785335 -68.2019845
C 17.6340751 14.8021464 -70.8021027
H 17.0888067 15.4656922 -71.4759625
H 18.5250258 14.4458703 -71.3186039
O 18.0387931 15.5263655 -69.6592734
H 17.3011331 15.5682376 -69.0191956
N 18.3085085 11.8526167 -69.839663

H 18.540374 11.8840546 -70.8182419
C 19.0346307 10.8940314 -69.0057114
H 19.4675616 10.1472293 -69.6713046
C 18.1121144 10.1118048 -68.0476025
O 18.3664568 10.0593789 -66.8463913
C 20.2060694 11.6112569 -68.3167241
H 19.8233584 12.3411857 -67.6012615
H 20.822286 12.1241624 -69.055329
H 20.8190484 10.8836954 -67.7848943
N 17.0082147 9.5547067 -68.5680509
H 16.8205954 9.6340163 -69.5538031
C 16.1258953 8.7216485 -67.7571872
H 16.0455573 9.2076642 -66.7871337
C 16.7726785 7.3640399 -67.4612841
O 17.037384 6.5613184 -68.3551552
C 14.6792575 8.6592675 -68.293561
H 14.2122908 9.6269136 -68.1101076
H 14.1203773 7.931987 -67.704949
C 14.479784 8.3334479 -69.7606206
O 14.1690558 7.2130685 -70.1375173
N 14.5042034 9.3331716 -70.6191546
H 14.1908225 9.1433714 -71.5603483
H 14.5384129 10.2922772 -70.2963576
N 17.0392665 7.1424113 -66.177116
H 16.830673 7.8928146 -65.5251381
C 17.5157217 5.8978433 -65.5954028
H 17.9177789 5.2616605 -66.384763
C 16.3357946 5.207524 -64.9288189
O 15.6159122 5.8225069 -64.1466295
C 18.6212937 6.1737034 -64.5672921
H 18.1711266 6.6156535 -63.6769974
H 19.0702786 5.2249355 -64.2716113
C 19.7093446 7.0913046 -65.0380035
C 19.7931824 8.4044157 -64.7311366
H 19.0720161 8.9404149 -64.124824
N 20.8504143 8.9783652 -65.4059943
H 20.9953366 9.9773354 -65.4322483
C 21.4912532 8.0630289 -66.2111008
C 22.5607112 8.1678796 -67.1126483
H 23.0547657 9.1163029 -67.2628505
C 22.976687 7.0234162 -67.8144749
H 23.7994497 7.0845023 -68.5131482
C 22.3164877 5.7977862 -67.6094385
H 22.6337933 4.9207785 -68.1562422
C 21.2368826 5.7069224 -66.707767
H 20.7248034 4.766601 -66.5741431

C 20.7952953 6.8365602 -65.9837824
N 16.1848563 3.912817 -65.176291
H 16.821799 3.469261 -65.8205527
C 15.0796393 3.1104436 -64.6775838
H 14.54308 3.6333506 -63.8833365
C 15.6247004 1.7985474 -64.1325112
O 16.0577958 0.9402323 -64.9020713
C 14.131694 2.8887033 -65.8487103
H 13.64995 3.8395378 -66.0623428
H 14.7289034 2.6128478 -66.7202805
C 13.0901348 1.8002141 -65.5654381
H 13.4487714 1.0489044 -64.8679001
C 11.8698551 2.4330043 -64.9143906
H 11.3898081 3.1481945 -65.575671
H 12.1699034 2.9421886 -63.9979327
H 11.1508884 1.6662489 -64.6483251
C 12.8433832 1.0456084 -66.8611406
H 12.3756177 1.6885177 -67.5980209
H 12.2143535 0.1981272 -66.6495824
H 13.7858171 0.6635186 -67.2549475
N 15.5022455 1.6123315 -62.8227831
H 15.0176951 2.3212374 -62.2749974
C 15.9819101 0.4156113 -62.1354336
H 16.5767406 -0.1688088 -62.8309248
C 14.7948239 -0.412656 -61.638592
O 14.0877462 0.0314062 -60.7298387
C 16.9228566 0.7713672 -60.9737509
H 16.3487296 1.1324943 -60.119959
C 17.7625409 -0.435787 -60.5611816
H 18.3692173 -0.7740933 -61.4018301
H 17.1180753 -1.2500821 -60.2345302
H 18.4224701 -0.1512396 -59.7425426
O 17.83844 1.7718527 -61.3585353
H 17.3314862 2.5714806 -61.5236629
N 14.5757734 -1.6381667 -62.1522123
C 13.7832369 -2.6243789 -61.4326093
H 12.8078798 -2.2156713 -61.1651937
C 14.5459745 -3.0172483 -60.1610187
O 15.6270337 -3.5983735 -60.2304277
C 13.5987399 -3.7952014 -62.405327
H 13.5382697 -4.7567764 -61.8952256
H 12.6985773 -3.6284725 -62.9974839
C 15.1494966 -2.2202696 -63.3610773
H 14.6833669 -1.7644417 -64.236083
H 16.2312348 -2.0961457 -63.4033807
C 14.823558 -3.7137458 -63.3158808

H 14.6132543 -4.1093829 -64.3096971
H 15.6551597 -4.2543635 -62.8618106
N 13.9662979 -2.746838 -58.9908767
H 13.0661721 -2.2754632 -59.0090904
C 14.5182136 -3.1102436 -57.6789229
H 15.4998097 -3.5591397 -57.8229977
C 13.7014467 -4.2044761 -56.9428997
O 13.6238671 -4.1604091 -55.7146881
C 14.714941 -1.81596 -56.8514598
H 13.7756341 -1.2643591 -56.8431172
H 14.9534945 -2.0737327 -55.819355
C 15.8229594 -0.873091 -57.3541501
H 15.6404761 -0.6146515 -58.3959849
C 15.8120489 0.4127376 -56.5303236
H 16.028931 0.1980959 -55.4826737
H 14.8309305 0.8833728 -56.593059
H 16.5554212 1.1109018 -56.9145674
C 17.2136708 -1.5018124 -57.2217831
H 17.3755269 -1.8389114 -56.1975999
H 17.9750578 -0.7631874 -57.470298
H 17.3151205 -2.3433035 -57.9050428
N 13.0948036 -5.214763 -57.6151661
C 12.2053006 -6.1721577 -56.9514439
H 11.3888186 -5.644905 -56.4617025
C 12.9249789 -7.0386965 -55.9075064
O 12.3195788 -7.4085346 -54.9111168
C 11.6162382 -7.0363963 -58.0682371
H 11.3764525 -8.0460584 -57.7312578
H 10.7270492 -6.5549761 -58.4702904
C 13.2765494 -5.6211828 -59.0043124
H 12.7053484 -4.9577645 -59.6524598
H 14.33053 -5.6213029 -59.2835882
C 12.7120005 -7.0340446 -59.1282169
H 12.3119336 -7.2235629 -60.1252091
H 13.4796364 -7.7662537 -58.8743209
N 14.2193922 -7.3309605 -56.0875591
H 14.6758111 -6.9951267 -56.9187628
C 15.0103271 -8.0990672 -55.112049
H 14.434692 -8.9910839 -54.8655987
C 15.2070223 -7.332927 -53.7964256
O 15.0766997 -7.9116657 -52.717904
C 16.3497863 -8.552893 -55.7413196
H 16.1067111 -9.1238207 -56.6393262
C 17.2457079 -7.3784555 -56.1790683
H 17.6104389 -6.833258 -55.3064419
H 16.7096291 -6.695959 -56.8363059

H 18.1109972 -7.7622999 -56.7213065
C 17.1758962 -9.4774166 -54.8192359
H 18.0864798 -9.7670527 -55.3455807
H 17.4716894 -8.935527 -53.9193684
C 16.4479887 -10.7630839 -54.4053729
H 16.0929498 -11.2919093 -55.2902479
H 15.605773 -10.5327737 -53.7532482
H 17.1389073 -11.4070984 -53.8607548
N 15.3935547 -6.0141343 -53.87863
H 15.4044782 -5.57695 -54.785056
C 15.4989416 -5.1573755 -52.7005961
H 16.1260958 -5.6549509 -51.9609007
C 14.1275042 -4.979588 -52.0493859
O 14.0041547 -5.2410058 -50.8625342
C 16.1529791 -3.8128398 -53.0612849
H 15.5453294 -3.3096024 -53.8136461
H 16.1749842 -3.1849001 -52.1697456
C 17.5888162 -3.9565456 -53.5969701
H 17.9604463 -2.9636573 -53.8583359
H 17.5825816 -4.5500643 -54.513358
C 18.5239075 -4.5941443 -52.560969
O 18.5957494 -5.8428049 -52.4662396
O 19.14147 -3.8633975 -51.7551244
N 13.0718701 -4.7526557 -52.834066
H 13.246795 -4.5814834 -53.8192455
C 11.6674641 -4.7573474 -52.3915481
H 11.5110988 -3.9424476 -51.6838844
C 11.2638044 -6.055375 -51.6705936
O 10.7000723 -5.9916155 -50.5815018
C 10.8225721 -4.4658085 -53.6373247
H 11.2703018 -4.9725609 -54.4847155
H 10.8980451 -3.3996945 -53.8162933
C 9.3400869 -4.8422281 -53.6032018
O 8.5960027 -4.4336681 -52.6834481
O 8.8625046 -5.4577929 -54.5871172
N 11.6917214 -7.2238873 -52.1577427
H 12.1378338 -7.2256039 -53.0701622
C 11.4803921 -8.5084706 -51.4804795
H 10.4192842 -8.5902366 -51.2439924
C 12.2581264 -8.616735 -50.1576347
O 11.7173718 -9.1125472 -49.1729519
C 11.8590517 -9.6523727 -52.4351888
H 12.9441046 -9.6885066 -52.5437256
H 11.4310558 -9.4628766 -53.4176041
C 11.3583046 -11.0199242 -51.9562242
H 11.7482812 -11.2164564 -50.9570363

H 11.7634607 -11.7769659 -52.6281464
S 9.5513028 -11.2041661 -51.9296248
C 9.4280423 -12.9497713 -51.463499
H 8.3802362 -13.2482151 -51.4521366
H 9.8585708 -13.0948903 -50.4720088
H 9.9653893 -13.5614913 -52.1881289
N 13.4992124 -8.1155487 -50.1013757
H 13.8751445 -7.7085546 -50.9531837
C 14.3462607 -8.0992723 -48.8917458
H 14.3261654 -9.0880359 -48.4302966
C 13.8414349 -7.109273 -47.8321094
O 13.8696885 -7.4088854 -46.6438106
C 15.7803139 -7.7771225 -49.3386629
H 16.0728206 -8.4953921 -50.1072518
H 15.78842 -6.7853287 -49.7877521
C 16.8146607 -7.8333564 -48.2019743
H 16.5459291 -7.1366515 -47.4067893
H 16.8280532 -8.8437172 -47.7908348
C 18.2187971 -7.4873246 -48.7193819
H 18.4184365 -8.0829343 -49.6128579
H 18.9536731 -7.7499724 -47.9570584
C 18.3487833 -5.992413 -49.0458139
H 18.4319706 -5.4217815 -48.1165061
H 17.4451803 -5.6561907 -49.5619324
N 19.5031294 -5.7293883 -49.9309239
H 20.3954323 -5.9966135 -49.5549232
H 19.5174901 -4.749167 -50.2346865
H 19.3464276 -6.1609146 -50.848426
N 13.3322727 -5.9630881 -48.2710839
H 13.3916351 -5.7966786 -49.2700395
C 12.6752334 -4.9259791 -47.4652083
H 13.3066394 -4.639705 -46.6204001
C 11.3514425 -5.4659331 -46.907204
O 11.0801258 -5.3692947 -45.708615
C 12.463281 -3.6936637 -48.3766892
H 12.0638674 -4.0566426 -49.3241162
C 11.3939495 -2.735481 -47.8251092
H 11.5699158 -2.5482408 -46.7698325
H 10.4027027 -3.1721763 -47.9439616
H 11.4167651 -1.795219 -48.3641339
C 13.8090055 -3.0011842 -48.7056933
H 14.1943818 -2.5034339 -47.8276267
H 14.5553025 -3.7476582 -48.9678682
C 13.7415405 -1.9940272 -49.8643963
H 13.1388901 -1.128049 -49.5957665
H 13.3172396 -2.4665957 -50.7493734

H 14.7489847 -1.6485579 -50.0979748
N 10.5569209 -6.1251239 -47.7575478
H 10.8058184 -6.1502801 -48.7417588
C 9.3406062 -6.8155937 -47.3499945
H 8.687436 -6.0980664 -46.8515223
C 9.6451199 -7.9320667 -46.3426887
O 8.9789309 -7.9857565 -45.3181334
C 8.6090851 -7.3439192 -48.5900398
H 9.2347282 -8.0646011 -49.1166765
H 8.3743305 -6.5167723 -49.2610325
H 7.6825766 -7.8315087 -48.286617
N 10.6967849 -8.7327052 -46.5459848
H 11.1877227 -8.6812436 -47.4322827
C 11.1410652 -9.7413254 -45.5766499
H 10.3095113 -10.4188421 -45.3765601
C 11.5417736 -9.1181235 -44.2316211
O 11.0027769 -9.5243077 -43.2065835
C 12.2879134 -10.5821685 -46.1574739
H 13.1383181 -9.9410307 -46.3900585
H 11.9539012 -11.054893 -47.0827182
C 12.7241101 -11.6616207 -45.1622198
O 13.5224159 -11.3337599 -44.252894
O 12.2018212 -12.793503 -45.265959
N 12.3829782 -8.0769071 -44.2118859
H 12.8033958 -7.7736289 -45.0842588
C 12.8418208 -7.4756884 -42.9545116
H 13.20788 -8.2795145 -42.315361
C 11.7105569 -6.7843906 -42.179033
O 11.5105007 -7.0762136 -40.995856
C 14.0107534 -6.5158331 -43.2086705
H 14.8199205 -7.0620265 -43.6953675
H 13.6867322 -5.7238326 -43.8863098
C 14.5352889 -5.889822 -41.9505213
C 15.3513898 -6.4920806 -41.0560262
H 15.7532765 -7.4912347 -41.1718495
N 15.5335855 -5.6654725 -39.9629168
H 16.0897329 -5.9158626 -39.160673
C 14.8148192 -4.49376 -40.0872147
C 14.6321954 -3.3866221 -39.242576
H 15.1032791 -3.3500841 -38.2735638
C 13.819259 -2.3236695 -39.676404
H 13.6551516 -1.4597462 -39.0434416
C 13.2231146 -2.3758491 -40.9490175
H 12.61592 -1.5480294 -41.2963996
C 13.413713 -3.4925454 -41.785488
H 12.9690569 -3.4997165 -42.7718436

C 14.1985453 -4.5894945 -41.3712467
N 10.9043925 -5.9527161 -42.8492866
H 11.1134384 -5.7630804 -43.8240366
C 9.7438538 -5.2921988 -42.2212211
H 10.0844229 -4.773692 -41.3238945
C 8.6828352 -6.2978319 -41.7797415
O 8.1475363 -6.1684389 -40.6765001
C 9.0796136 -4.2487583 -43.1304959
H 8.177974 -3.8895772 -42.6371761
C 9.9720763 -3.0467225 -43.4104666
H 10.8669468 -3.3593763 -43.9445606
H 10.2661708 -2.5763767 -42.4714854
H 9.4314004 -2.3171273 -44.0117579
O 8.7181591 -4.8066588 -44.3726664
H 9.5496823 -4.9479732 -44.8577988
N 8.4358032 -7.3581704 -42.5633335
H 8.8963046 -7.4314515 -43.466883
C 7.5723311 -8.4495715 -42.1268535
H 6.6350766 -8.0155664 -41.7795424
C 8.1700975 -9.1517994 -40.9247922
O 7.4758181 -9.174459 -39.9330543
C 7.2448461 -9.4620492 -43.2330206
H 8.1639771 -9.7685181 -43.7320877
H 6.8107539 -10.3548548 -42.779457
C 6.239774 -8.9211213 -44.2597927
H 6.2060887 -9.6067299 -45.1067549
H 6.5649357 -7.9487869 -44.6272582
C 4.8301142 -8.7907822 -43.6962677
O 4.0185217 -9.6936336 -43.7800733
N 4.4373901 -7.6329512 -43.2092787
H 3.4549186 -7.5555601 -42.9669857
H 5.0762198 -6.8618807 -43.140931
N 9.4273277 -9.6006942 -40.9284614
H 9.9686587 -9.4839231 -41.780828
C 10.0214635 -10.412134 -39.8567427
H 9.3084547 -11.1990181 -39.609992
C 10.253828 -9.6601678 -38.5291229
O 10.2250454 -10.2893742 -37.4666527
C 11.2944858 -11.0964365 -40.3945858
H 11.8618845 -11.5134649 -39.5643313
H 11.9244235 -10.3674132 -40.904203
C 10.9694015 -12.2521054 -41.3360884
O 10.0165345 -12.9890257 -41.1245311
N 11.7728694 -12.4937022 -42.343769
H 11.4995019 -13.1733068 -43.0362986
H 12.499172 -11.8467642 -42.6449554

N 10.2929531 -8.3240194 -38.537167
H 10.3624872 -7.8437779 -39.4304161
C 10.2075646 -7.5261957 -37.311539
H 10.9370343 -7.9083139 -36.5955343
C 8.8173012 -7.6078404 -36.6366541
O 8.7224682 -7.6976754 -35.4119872
C 10.5911494 -6.0827475 -37.6571958
H 9.8848154 -5.6694188 -38.3793169
H 11.5923603 -6.0574983 -38.0909011
H 10.5793001 -5.471428 -36.7549126
N 7.7281362 -7.648853 -37.4093658
H 7.8464977 -7.6620426 -38.4175533
C 6.3615845 -7.7065981 -36.8837774
H 6.2525433 -6.8732437 -36.1891368
C 5.9991926 -8.9928891 -36.0905116
O 5.3798625 -8.8602907 -35.0312186
C 5.3875801 -7.4605357 -38.0417105
H 5.317507 -8.3466364 -38.6717451
H 5.7360643 -6.6360806 -38.6607618
H 4.3964641 -7.2320804 -37.651936
N 6.3537059 -10.227742 -36.5201682
C 6.0921003 -11.4546801 -35.7992746
H 5.0595046 -11.4728627 -35.4552056
C 7.0141917 -11.5868196 -34.5825291
O 6.5442037 -12.022288 -33.5387212
C 6.2944393 -12.5878507 -36.8162142
H 6.5796654 -13.5239315 -36.3340225
H 5.3790469 -12.7278175 -37.3944718
C 6.9710996 -10.6108314 -37.7595143
H 6.2311127 -10.4906961 -38.55036
H 7.8621115 -10.0271148 -37.9027458
C 7.3852351 -12.0701274 -37.7139111
H 7.3632158 -12.5333561 -38.7012058
H 8.3603036 -12.1870356 -37.2408137
N 8.2600128 -11.0993172 -34.6473682
H 8.5974122 -10.7351963 -35.529587
C 9.1638845 -11.0324667 -33.493428
H 9.2559856 -12.0264496 -33.0531509
C 8.6246533 -10.1030979 -32.3918366
O 8.5898426 -10.4804776 -31.2234502
C 10.5463395 -10.5906214 -33.9929249
H 10.9400176 -11.3588206 -34.6604347
H 10.4349591 -9.6795107 -34.5821898
C 11.5645544 -10.3336599 -32.8982995
C 12.1676299 -11.4139774 -32.226674
H 11.9007571 -12.4282594 -32.4853161

C 13.115578 -11.1764532 -31.2147571
H 13.5750579 -12.007349 -30.6985902
C 13.464218 -9.8578058 -30.873536
H 14.194 -9.6750549 -30.0974929
C 12.8624226 -8.7769348 -31.541472
H 13.1324783 -7.7636193 -31.281157
C 11.9107974 -9.0133361 -32.5496745
H 11.4482941 -8.1777601 -33.0568941
N 8.0603085 -8.9495652 -32.7631566
H 8.1434311 -8.665565 -33.7337643
C 7.4168677 -8.0211198 -31.8271501
H 7.9214617 -8.1082216 -30.8649975
C 5.9315139 -8.326438 -31.5456683
O 5.2535744 -7.4801421 -30.961817
C 7.5941536 -6.574922 -32.324633
H 7.124499 -6.4941971 -33.3064549
H 7.0538484 -5.9068789 -31.6550215
C 9.0136829 -6.0386909 -32.3987931
C 9.4463363 -5.359517 -33.5561945
H 8.7912065 -5.2787379 -34.4129759
C 10.7433545 -4.8150995 -33.6203321
H 11.075635 -4.3075551 -34.5142454
C 11.6148391 -4.9453172 -32.5180674
O 12.8717441 -4.435554 -32.5709646
H 13.0345853 -3.9454995 -33.3800472
C 11.174962 -5.6029107 -31.3511048
H 11.8453005 -5.6829437 -30.507972
C 9.8769556 -6.144496 -31.2893049
H 9.5563376 -6.6474406 -30.3872537
N 5.3871766 -9.481238 -31.956426
H 6.0033601 -10.1538206 -32.4006634
C 3.9274016 -9.6951489 -32.0875758
H 3.5788743 -9.0167038 -32.8656313
C 3.101727 -9.3795924 -30.8315989
O 1.9946272 -8.8568 -30.9597617
C 3.6184426 -11.1199467 -32.5819641
H 4.1373467 -11.2775613 -33.5237867
C 4.0262775 -12.229422 -31.6076246
H 3.4924745 -12.148523 -30.6645446
H 5.0959923 -12.1726016 -31.405786
H 3.820187 -13.1996915 -32.0601955
O 2.2341245 -11.2757929 -32.8256162
H 2.1031661 -11.0808149 -33.7675107
N 3.6312656 -9.6287895 -29.6257628
H 4.5649006 -10.0097299 -29.5872965
C 2.9405287 -9.3177676 -28.3687039

H 1.9221669 -9.6969241 -28.445873
C 2.8276563 -7.8017421 -28.1507249
O 1.7167741 -7.2833488 -28.1555535
C 3.6362754 -10.0368499 -27.1973246
H 3.6353806 -11.1098669 -27.3948457
H 4.6745595 -9.7054275 -27.1474315
C 2.978396 -9.7839054 -25.8233671
H 2.9966347 -8.7178673 -25.6005677
C 1.5310448 -10.2812004 -25.7697653
H 1.4817257 -11.3377576 -26.0293787
H 0.9096754 -9.6938931 -26.4446131
H 1.1425322 -10.1385847 -24.7604909
C 3.7720642 -10.514862 -24.7416685
H 3.7563083 -11.5903824 -24.9153329
H 3.3368301 -10.2987848 -23.7649057
H 4.8026104 -10.1599394 -24.7388168
N 3.9616382 -7.0915666 -28.1298216
H 4.8305866 -7.5964343 -28.1841824
C 4.0327294 -5.6286734 -27.9990707
H 3.5475118 -5.3373421 -27.066531
C 3.3223149 -4.9052738 -29.1588863
O 2.7887892 -3.8112358 -29.0000078
C 5.522688 -5.2420034 -27.9073454
H 5.9417744 -5.7125461 -27.0159418
H 6.0387796 -5.6508386 -28.77724
C 5.8148372 -3.7290084 -27.839953
H 5.4302001 -3.2507583 -28.740477
C 5.2052008 -3.0369301 -26.6265279
H 5.5270028 -3.5308572 -25.7079944
H 4.1165442 -3.0902357 -26.6670599
H 5.5002705 -1.9890255 -26.5938106
C 7.3263404 -3.5054407 -27.8182893
H 7.7487717 -3.9367071 -26.9075049
H 7.5460048 -2.4366408 -27.8259105
H 7.7977514 -3.9704279 -28.681937
N 3.3240981 -5.4922525 -30.3565642
H 3.8200164 -6.3736162 -30.4481514
C 2.6217824 -4.9657189 -31.5266407
H 2.8835245 -3.9148142 -31.6417372
C 1.0943839 -5.029999 -31.3514762
O 0.4108227 -4.0473604 -31.6319395
C 3.127867 -5.7370606 -32.7582961
H 4.209855 -5.607543 -32.8206349
H 2.9292394 -6.7975108 -32.6056175
C 2.5111026 -5.3318787 -34.1077532
H 1.4532277 -5.5947615 -34.1197899

C 2.6686143 -3.8435188 -34.4097948
H 3.7203921 -3.5715168 -34.3494023
H 2.0853599 -3.2554452 -33.704076
H 2.2981698 -3.6320755 -35.4139741
C 3.226156 -6.095398 -35.2194507
H 4.2560045 -5.7514367 -35.3113525
H 2.7083162 -5.937219 -36.1650524
H 3.2404669 -7.1588567 -34.9842874
N 0.5617721 -6.1321914 -30.8100106
H 1.1854655 -6.8955181 -30.565335
C -0.8774119 -6.2932057 -30.5294087
H -1.4450707 -5.8133694 -31.3273322
C -1.3387857 -5.5989339 -29.2472027
O -2.4972833 -5.2107496 -29.1799714
C -1.2259879 -7.7857952 -30.5059816
H -0.5395652 -8.3161481 -29.8425807
H -2.2423577 -7.9080194 -30.1304602
C -1.1550235 -8.3653732 -31.9235204
H -0.1595575 -8.2283015 -32.3457716
H -1.8592111 -7.825137 -32.5503861
C -1.517416 -9.8499764 -31.9535904
H -1.7789138 -10.1101266 -32.9786297
H -2.3926915 -10.0247935 -31.3275004
N -0.3815104 -10.6888054 -31.5472774
H 0.4686067 -10.5891001 -32.0922565
C -0.2819976 -11.5284697 -30.5397689
N 0.7987728 -12.2399241 -30.4453776
H 0.9213052 -12.9191248 -29.7195572
H 1.4926744 -12.1243647 -31.1788985
N -1.2140364 -11.691179 -29.6413828
H -2.0524106 -11.1461476 -29.7056909
H -1.0837421 -12.3425586 -28.8909044
N -0.4359914 -5.4137744 -28.291648
H 0.4565919 -5.878876 -28.4106519
C -0.6121594 -4.6637386 -27.0405899
H -1.5484464 -4.9448234 -26.5574101
C -0.6419177 -3.1442925 -27.2699944
O -1.4719593 -2.454789 -26.6849815
C 0.5604857 -5.0831334 -26.1452238
H 0.4619065 -6.1502756 -25.9371324
H 1.4675594 -4.941078 -26.7285774
C 0.7664583 -4.3591921 -24.8161269
H -0.1072872 -4.5150072 -24.1788573
H 0.8875321 -3.2879945 -24.9917974
C 2.035639 -4.9226683 -24.1581527
O 1.8929473 -5.6917077 -23.1831504

O 3.1384206 -4.6239782 -24.6732221
N 0.1917402 -2.6286023 -28.1822229
H 0.9142711 -3.2259607 -28.5641261
C 0.1832659 -1.2150978 -28.5658876
H 0.0037262 -0.6137299 -27.6722889
C -0.9246664 -0.8558517 -29.5768898
O -1.3427934 0.2986789 -29.6289793
C 1.5712314 -0.8568396 -29.111109
H 1.7755088 -1.4407739 -30.0082925
H 2.3275326 -1.0683264 -28.3535512
H 1.5991124 0.2052893 -29.3567835
N -1.3690744 -1.8151607 -30.399589
H -0.9570034 -2.7323259 -30.3107115
C -2.4122034 -1.6197109 -31.4130676
H -2.8401389 -0.6237023 -31.2850044
C -3.5838278 -2.5891446 -31.1989809
O -4.4984085 -2.2893246 -30.4387788
C -1.8158924 -1.6685056 -32.833723
H -1.3145361 -2.624073 -32.987596
H -2.63415 -1.6141187 -33.553035
C -0.8409829 -0.5505884 -33.1512774
C -1.3096435 0.649862 -33.7150379
H -2.3647469 0.7833783 -33.9051926
C 0.531843 -0.7025696 -32.8802269
H 0.8864084 -1.6149005 -32.4260483
C -0.4110316 1.6879453 -34.01348
H -0.7806818 2.6123909 -34.4308109
C 1.4300197 0.3388147 -33.1757978
H 2.4760809 0.2347951 -32.9481857
C 0.9602995 1.5313944 -33.7512922
H 1.6466328 2.3356076 -33.9776088
N -3.6205628 -3.7132496 -31.9236746
H -2.8479066 -3.9034345 -32.5436994
C -4.6046085 -4.7861899 -31.7255437
H -4.6477881 -5.0219769 -30.6606922
C -4.2176418 -6.0541322 -32.4919207
O -3.4466607 -6.006089 -33.4557384
C -6.0082382 -4.3507814 -32.1811528
H -6.3304691 -3.4699273 -31.624698
H -6.7175425 -5.152142 -31.9705056
O -6.0325689 -4.0692292 -33.5679786
H -5.899994 -3.1163384 -33.6518697
N -4.8741413 -7.1737827 -32.163381
H -5.4716506 -7.1487112 -31.3494907
C -4.8665038 -8.4037354 -32.9751168
H -3.8421984 -8.7622554 -33.024553

C -5.296619 -8.1459118 -34.4291164
O -4.6251069 -8.5946963 -35.3565651
C -5.7191938 -9.4992887 -32.2851475
H -5.2617698 -9.6962688 -31.3135578
C -7.1749464 -9.0643969 -32.0189038
H -7.7180113 -8.9252308 -32.9539572
H -7.2157387 -8.1483551 -31.4309694
H -7.6857446 -9.8419113 -31.4478146
C -5.7591062 -10.8389807 -33.0521625
H -6.3486961 -11.5529072 -32.4750014
H -6.2623358 -10.6958331 -34.0075932
C -4.3868889 -11.4732058 -33.3065014
H -3.86465 -11.6184485 -32.3614838
H -3.7929475 -10.8453952 -33.9693974
H -4.5241808 -12.4437534 -33.7840225
N -6.3197801 -7.3081212 -34.6459337
H -6.7867577 -6.9174776 -33.8407924
C -6.8108968 -6.9429317 -35.9822531
H -6.9295796 -7.8697976 -36.5438468
C -5.8010042 -6.0946841 -36.7703672
O -5.7534856 -6.2049544 -37.9993011
C -8.1929232 -6.2678168 -35.828368
H -8.8936588 -6.9935093 -35.4113768
H -8.1035006 -5.4462128 -35.1147961
C -8.795135 -5.6932521 -37.1238768
H -9.7559562 -5.2332397 -36.8894435
H -8.1411857 -4.8942535 -37.4703831
C -9.0162358 -6.7421972 -38.2346595
H -9.9843958 -7.2211919 -38.076221
H -8.2596339 -7.5233973 -38.1830747
C -8.9707821 -6.1540308 -39.6519625
H -9.7832162 -5.4324325 -39.7803459
H -9.1312743 -6.9749907 -40.3575952
N -7.6582374 -5.5172426 -39.9201159
H -7.5238449 -4.6979612 -39.3466649
H -7.5232324 -5.260878 -40.8933727
H -6.8930147 -6.1433565 -39.6825251
N -4.9831557 -5.2914209 -36.0868715
H -5.1114733 -5.2316311 -35.0833861
C -3.8621657 -4.5557308 -36.6755995
H -4.2125805 -3.9476157 -37.5101316
H -3.4303927 -3.8987074 -35.9202956
C -2.7652385 -5.4947951 -37.177779
O -2.4814048 -5.5085537 -38.373279
N -2.2677174 -6.3806718 -36.3070245
H -2.5945535 -6.342489 -35.3479413

C -1.2607822 -7.3958332 -36.6512525
H -0.3445124 -6.8925585 -36.961776
C -1.6939054 -8.2984128 -37.8176158
O -0.9311845 -8.4717807 -38.7673839
C -0.9587692 -8.2239316 -35.3945171
H -0.5126998 -7.5591821 -34.6545479
H -1.9003645 -8.5918536 -34.9852958
C -0.0205586 -9.403772 -35.5957312
C -0.476939 -10.7237244 -35.4080804
H -1.509788 -10.9114615 -35.1526382
C 0.4018223 -11.8087914 -35.5828742
H 0.0411055 -12.8215519 -35.4605813
C 1.7396798 -11.5814567 -35.9510346
H 2.4073452 -12.4177367 -36.1126058
C 2.197147 -10.2669613 -36.1448257
H 3.2167878 -10.0866761 -36.4561706
C 1.3158525 -9.1847377 -35.975059
H 1.6594669 -8.1812815 -36.1674815
N -2.9508894 -8.7602365 -37.8162996
H -3.5180262 -8.6013591 -36.9897401
C -3.5164419 -9.5705426 -38.9055008
H -2.8830213 -10.4489155 -39.0412821
C -3.4884375 -8.8191223 -40.2441416
O -3.0854456 -9.4032043 -41.2444796
C -4.9353343 -10.0606912 -38.5229828
H -5.4930771 -9.2156787 -38.1147458
C -5.7199256 -10.6008875 -39.7362459
H -5.1748012 -11.428586 -40.1934969
H -5.8704642 -9.8203191 -40.4808494
H -6.7059161 -10.950001 -39.4339102
C -4.8187313 -11.1576382 -37.4374046
H -4.3660146 -12.0503218 -37.8713733
H -4.1598068 -10.814504 -36.6407699
C -6.1553173 -11.5460725 -36.7923586
H -6.7955629 -12.0580817 -37.5094256
H -6.6616767 -10.6550823 -36.4198857
H -5.9674939 -12.2254155 -35.9610021
N -3.8323925 -7.5248185 -40.2879394
H -4.0656676 -7.0556824 -39.4218744
C -3.7691065 -6.745843 -41.5391746
H -4.3005425 -7.2994218 -42.3139513
C -2.3393446 -6.5519942 -42.0526116
O -2.1145754 -6.6247537 -43.2621331
C -4.4332741 -5.3718712 -41.3703427
H -3.8537605 -4.6109815 -41.8970195
H -4.4733346 -5.1006908 -40.3138927

O -5.7412933 -5.3987764 -41.9212897
H -5.6390313 -5.3843889 -42.8827614
N -1.3861787 -6.3126124 -41.1495095
H -1.6547347 -6.2826085 -40.1714205
C 0.0158965 -6.0425797 -41.481886
H 0.0577829 -5.3112942 -42.2894989
C 0.740751 -7.3012983 -41.9896076
O 1.4825599 -7.2298511 -42.9719905
C 0.6993255 -5.4537121 -40.2332656
H 0.5759614 -6.161702 -39.4119538
H 1.768021 -5.3583632 -40.4270006
C 0.1460188 -4.0790425 -39.7977291
H -0.9410754 -4.0968931 -39.8068985
C 0.5881962 -3.7787531 -38.3651539
H 1.6688825 -3.8436922 -38.2817429
H 0.1358835 -4.4987693 -37.6855946
H 0.2728934 -2.7773283 -38.0815491
C 0.5838749 -2.9467471 -40.7300837
H 1.6679734 -2.8669288 -40.751094
H 0.1714852 -2.0035095 -40.3724266
H 0.2150466 -3.1247907 -41.7383577
N 0.4550266 -8.4611916 -41.3890336
H -0.1379686 -8.4416052 -40.564342
C 0.9646168 -9.7595951 -41.8364496
H 2.0196674 -9.6433663 -42.0892642
C 0.2485841 -10.2596498 -43.1035624
O 0.8987943 -10.7616169 -44.0158858
C 0.8455888 -10.7511466 -40.6638834
H 1.3523783 -10.326376 -39.7955445
H -0.210257 -10.8640917 -40.4110157
C 1.4357562 -12.1477194 -40.9424942
H 0.9147976 -12.6038002 -41.7839374
C 2.9345315 -12.1074372 -41.2461209
H 3.4682867 -11.5756565 -40.4587843
H 3.1048157 -11.6059846 -42.1990644
H 3.3258448 -13.1209115 -41.3293987
C 1.228386 -13.036948 -39.7157139
H 1.7534838 -12.6171902 -38.8578697
H 1.6078131 -14.0384338 -39.9180822
H 0.1647686 -13.1057515 -39.4881301
N -1.0698244 -10.0583188 -43.2209953
H -1.5784126 -9.6705319 -42.4337335
C -1.8288138 -10.4543792 -44.4133578
H -1.6406428 -11.512455 -44.6026739
C -1.3632127 -9.6903915 -45.6534104
O -1.0336879 -10.3147171 -46.6612399

C -3.3368109 -10.2801862 -44.1613091
H -3.5384081 -9.2595103 -43.8397539
H -3.6148275 -10.9493552 -43.3461491
C -4.2354917 -10.6192243 -45.3629775
H -5.2387681 -10.8120393 -44.981554
H -3.8764807 -11.5469091 -45.8110439
S -4.390968 -9.3657168 -46.6781462
C -5.1799099 -8.0079527 -45.7729399
H -5.4383667 -7.2116522 -46.4701612
H -4.4931662 -7.6138861 -45.0242914
H -6.0870682 -8.3705426 -45.2881865
N -1.2836702 -8.3547994 -45.597801
H -1.5351508 -7.8696527 -44.7427603
C -0.9788531 -7.5879528 -46.8043397
H -1.457325 -8.1034868 -47.6360249
C 0.521404 -7.5756896 -47.1451478
O 0.8637811 -7.5102657 -48.3264741
C -1.6430715 -6.2032923 -46.748782
H -1.1355764 -5.5871202 -46.0089988
H -2.6802938 -6.3144597 -46.4385527
C -1.6394516 -5.5151375 -48.1094585
N -0.9709235 -4.389742 -48.2157069
H -0.4685428 -4.0199304 -47.4270163
H -0.9501478 -3.9346266 -49.1103779
O -2.2486604 -5.9590541 -49.0785727
N 1.416904 -7.7516498 -46.1678263
H 1.0980991 -7.8154289 -45.2078644
C 2.8375119 -7.9866019 -46.4363406
H 3.1969886 -7.2710589 -47.1758242
H 3.3911814 -7.8211393 -45.5225839
C 3.1279278 -9.4043741 -46.945777
O 3.8298141 -9.5493483 -47.9453507
N 2.447086 -10.4225332 -46.4101552
H 1.9315207 -10.2624063 -45.5490469
C 2.4854058 -11.7979805 -46.9139969
H 3.5152832 -12.1562071 -46.9087821
H 1.8931677 -12.4278665 -46.2504679
C 1.9239234 -11.9295401 -48.3328251
O 2.5932635 -12.4826701 -49.2022689
N 0.7929469 -11.2777152 -48.628854
H 0.267872 -10.8632915 -47.8641561
C 0.2683398 -11.1228987 -49.9938683
H 0.0573556 -12.1067264 -50.4128921
C 1.2826057 -10.4351154 -50.919821
O 1.4660857 -10.8427349 -52.0678355
C -1.03995 -10.3126457 -49.9511539

H -1.8060414 -10.9034882 -49.4476874
H -0.8743164 -9.4136616 -49.3588149
C -1.5543657 -9.8836418 -51.2950987
C -1.1187229 -8.7991881 -51.9758444
H -0.3563124 -8.1147227 -51.6133052
N -1.6791194 -8.7882528 -53.2363242
H -1.3736528 -8.1625098 -53.9676252
C -2.5438482 -9.8457334 -53.4186151
C -3.3411695 -10.2526051 -54.4992947
H -3.3476281 -9.6931163 -55.422582
C -4.1539985 -11.3894695 -54.358604
H -4.801853 -11.6994674 -55.1695271
C -4.1314936 -12.1172815 -53.1550939
H -4.7589197 -12.9925633 -53.044334
C -3.3101688 -11.7105559 -52.0842815
H -3.3046579 -12.2804389 -51.1652787
C -2.499787 -10.5592805 -52.1831049
N 1.96428 -9.3993704 -50.4253499
H 1.7809878 -9.1156849 -49.4709382
C 2.9636726 -8.654149 -51.2011413
H 2.5137098 -8.3680451 -52.1510126
C 4.1751778 -9.5252683 -51.5380323
O 4.584636 -9.5468988 -52.6991712
C 3.3562456 -7.3613529 -50.4714756
H 3.7076416 -7.5770001 -49.4645344
C 4.4220107 -6.5580398 -51.2039862
H 4.0784879 -6.3762013 -52.2179027
H 5.3645424 -7.1059384 -51.2128172
H 4.574357 -5.6062331 -50.6915057
O 2.2203371 -6.5288334 -50.4111686
H 1.6945377 -6.8265429 -49.6442254
N 4.6517677 -10.3483214 -50.5991615
H 4.2697926 -10.2894075 -49.6593257
C 5.6772822 -11.3580867 -50.8568759
H 6.5451977 -10.8700353 -51.3041686
C 5.1923812 -12.4341222 -51.8490049
O 5.8968642 -12.7354913 -52.8110289
C 6.1067911 -11.9647842 -49.5145566
H 5.2580115 -12.4435631 -49.0255728
H 6.499734 -11.1841373 -48.8617151
H 6.8863492 -12.7084471 -49.6781218
N 3.9534366 -12.9249302 -51.7086818
H 3.4267844 -12.6496469 -50.8836684
C 3.334672 -13.8997941 -52.6291921
H 3.9737587 -14.7791788 -52.6745089
C 3.2311635 -13.3581777 -54.0601941

O 3.501972 -14.0879971 -55.0132972
C 1.9611062 -14.3607064 -52.0920771
H 1.4005589 -13.4924383 -51.7508018
C 1.0987965 -15.094003 -53.1308964
H 1.6582642 -15.9270397 -53.5595551
H 0.8015677 -14.4086898 -53.9226587
H 0.1946828 -15.4764729 -52.6554527
C 2.1541222 -15.3188572 -50.9094302
H 2.6359165 -16.2380737 -51.2437664
H 1.18814 -15.559973 -50.4658729
H 2.7765934 -14.8574841 -50.1444956
N 2.9105554 -12.073782 -54.2549198
H 2.6628376 -11.510579 -53.4444772
C 2.8382215 -11.4848562 -55.603466
H 2.5477263 -12.2731829 -56.294507
C 4.1932573 -11.0523229 -56.1735234
O 4.3050569 -11.0014922 -57.3976465
C 1.7500749 -10.3972881 -55.6684607
H 1.973677 -9.6224873 -54.932472
H 1.7766916 -9.9369756 -56.657886
C 0.3181127 -10.9321047 -55.4242165
H 0.2277581 -11.265099 -54.390261
H -0.3748739 -10.1024204 -55.5677658
C -0.0987512 -12.0894509 -56.3560436
H 0.544746 -12.9544503 -56.17014
H 0.0153415 -11.7777132 -57.39766
C -1.5577073 -12.5187353 -56.1029819
H -2.2269318 -11.6802266 -56.3325773
H -1.6736438 -12.7561101 -55.0366384
N -1.920309 -13.7026939 -56.930816
H -1.8494992 -13.5031153 -57.9228767
H -2.903941 -13.9547134 -56.8042702
H -1.3493329 -14.5134468 -56.71273
N 5.239108 -10.8811623 -55.358782
H 5.0832237 -10.9063656 -54.3572045
C 6.6252368 -10.7490509 -55.8568326
H 6.6136699 -10.1725473 -56.7835268
C 7.23075 -12.111499 -56.2120809
O 7.8163342 -12.237301 -57.2830009
C 7.5248285 -9.9796415 -54.8686851
H 7.4639628 -10.4501565 -53.8877875
C 8.9905939 -9.944826 -55.3227672
H 9.0568977 -9.5997377 -56.3555774
H 9.4372889 -10.9357012 -55.2428839
H 9.5579733 -9.2635562 -54.6896495
C 7.0793244 -8.5186274 -54.7449023

H 7.2214826 -8.0117064 -55.6995769
H 7.6884329 -8.0243809 -53.9867521
H 6.0354079 -8.4688468 -54.4529373
N 7.0278598 -13.1595392 -55.4024952
H 6.5409429 -13.011564 -54.5230944
C 7.6271667 -14.4858677 -55.6559459
H 8.6634613 -14.3031979 -55.9438593
C 6.9906641 -15.2279986 -56.8419336
O 7.6716931 -15.9879257 -57.5225639
C 7.6575082 -15.3317962 -54.3638388
H 7.9124323 -14.6589608 -53.5431137
C 6.2996944 -15.9707711 -54.0400428
H 6.0816508 -16.7851927 -54.7321286
H 5.5262014 -15.2171777 -54.1221082
H 6.2972443 -16.3543042 -53.0201916
C 8.777694 -16.3911983 -54.4438692
H 8.5331507 -17.1303691 -55.2074872
H 9.7068405 -15.8989223 -54.7355244
C 9.0351373 -17.1189259 -53.1182629
H 8.173149 -17.7267722 -52.8436591
H 9.2387219 -16.3944539 -52.3289923
H 9.8999306 -17.7735446 -53.2299662
N 5.7387061 -14.9039989 -57.1868191
H 5.2127234 -14.3222172 -56.5485866
C 5.1062204 -15.3009733 -58.4534771
H 5.3874356 -16.3318692 -58.6680816
C 5.6030433 -14.4874707 -59.672003
O 5.0849158 -14.6599443 -60.773668
C 3.5735904 -15.2270815 -58.2905951
H 3.3168007 -14.2373792 -57.910768
H 3.1057086 -15.3358337 -59.269651
C 2.9775319 -16.3018904 -57.3585176
H 3.4899355 -16.2918303 -56.3990126
C 1.4957103 -15.9962792 -57.1210999
H 0.9610944 -15.9934025 -58.0710052
H 1.4152946 -15.0272253 -56.6348142
H 1.0717324 -16.755584 -56.4636815
C 3.0815974 -17.7101515 -57.947436
H 2.616133 -17.7444842 -58.9324024
H 2.5847047 -18.4241014 -57.2906702
H 4.1275589 -18.0041337 -58.0324987
N 6.5584693 -13.5695965 -59.4840437
H 6.9635998 -13.4805897 -58.559394
C 7.1419561 -12.7294521 -60.533019
H 8.0300305 -12.2428227 -60.1296168
H 7.4428545 -13.3604889 -61.3694312

C 6.2113295 -11.6372743 -61.0664694
O 6.4668133 -11.1007314 -62.1414297
N 5.0989407 -11.3521821 -60.3807085
H 4.9813281 -11.7997437 -59.4822739
C 4.011173 -10.5016823 -60.8819061
H 4.0372055 -10.488733 -61.9692609
C 4.1458654 -9.0360507 -60.4550618
O 4.0311999 -8.1487806 -61.2999311
C 2.6640571 -11.1114873 -60.4638078
H 1.8926477 -10.351034 -60.4842377
H 2.730934 -11.4831747 -59.4414438
C 2.2535011 -12.2657448 -61.3868724
H 1.4591474 -12.8339579 -60.902344
H 3.1069454 -12.932034 -61.5231857
S 1.6498686 -11.7327541 -63.0139356
C 1.6214996 -13.3280329 -63.8756168
H 1.2174771 -13.1904539 -64.8799789
H 0.9970689 -14.0358844 -63.3303468
H 2.6341481 -13.7267153 -63.9450518
N 4.4403578 -8.7747006 -59.1755103
H 4.5741277 -9.5745046 -58.565276
C 4.7721047 -7.4432695 -58.6461105
H 4.2329189 -6.6929209 -59.2287529
C 6.2628089 -7.1513591 -58.8255713
O 7.1132365 -8.0286716 -58.7190631
C 4.3124856 -7.3225627 -57.1776623
H 3.2218766 -7.3201566 -57.1538076
H 4.664482 -8.1940433 -56.6248092
C 4.8413101 -6.0586761 -56.4669405
H 5.9269408 -6.1201353 -56.3940316
H 4.5835999 -5.1738691 -57.0510368
C 4.2767258 -5.8952971 -55.0518431
H 4.2724669 -6.8599056 -54.5417714
H 3.2469937 -5.5465683 -55.1283597
C 5.0870934 -4.8828455 -54.2236432
H 4.4787623 -4.580427 -53.3658758
H 5.2958632 -3.991815 -54.8235343
N 6.3573003 -5.466491 -53.7188249
H 6.2395348 -6.3113772 -53.1868018
H 6.9070721 -4.8226841 -53.1439003
H 7.0461657 -5.6510471 -54.4521823
N 6.5831059 -5.8796803 -59.0166479
H 5.8235267 -5.2105063 -59.113986
C 7.9375657 -5.3227778 -59.0310328
H 8.6143732 -5.9856498 -58.4939189
C 7.9299697 -3.9753104 -58.3016906

O 6.8849011 -3.3299543 -58.2056804
C 8.3940536 -5.2114097 -60.498702
H 7.5989185 -4.7461053 -61.083704
C 9.6867695 -4.4393521 -60.7534294
H 10.461601 -4.7634189 -60.0624864
H 9.5092652 -3.3712309 -60.637248
H 10.0261984 -4.6212524 -61.7728531
O 8.6164189 -6.5095718 -60.9963761
H 8.1255677 -7.1366062 -60.4489059
N 9.0868513 -3.5097747 -57.8288001
H 9.9133854 -4.0836863 -57.9019445
C 9.2842826 -2.0970461 -57.484942
H 8.3344924 -1.5820776 -57.6283965
C 10.2397767 -1.4588863 -58.488381
O 11.1822076 -2.0878065 -58.9748673
C 9.6723067 -1.8988096 -55.9998239
H 9.4745394 -2.8358148 -55.4851521
C 11.1589555 -1.5434698 -55.8111287
H 11.3819425 -0.5661663 -56.2441685
H 11.7740098 -2.3086833 -56.2769605
H 11.4226248 -1.4985324 -54.7557414
C 8.7636256 -0.8305709 -55.3496209
H 8.9602225 0.1445593 -55.7991688
H 7.7226656 -1.0931424 -55.5430241
C 8.9349449 -0.7232145 -53.8274753
H 9.9006709 -0.2819557 -53.5826193
H 8.8562241 -1.7099889 -53.3708842
H 8.1532925 -0.0869728 -53.4164581
N 9.9517901 -0.2103244 -58.8094482
H 9.1682783 0.231535 -58.3373574
C 10.654951 0.6360141 -59.7608788
H 11.4393476 0.0802286 -60.2761536
C 11.3137825 1.7707113 -58.9834636
O 10.6744127 2.3280577 -58.0948429
C 9.5994166 1.0937026 -60.7929687
H 8.6089785 1.028997 -60.3467226
H 9.6158493 0.3827572 -61.6188321
C 9.6756245 2.4856417 -61.3678588
N 9.6560584 2.7683857 -62.7304314
C 9.6304442 4.1041051 -62.8320208
H 9.6677495 4.647484 -63.7669766
N 9.599696 4.6686446 -61.6145544
H 9.6754936 5.6576488 -61.4159251
C 9.6037052 3.6646771 -60.6808175
H 9.5692624 3.7963986 -59.6106405
N 12.5267095 2.1509732 -59.3842939

H 13.0058659 1.5797279 -60.0714761
C 13.1036285 3.4786336 -59.1419166
H 12.3791542 4.0801703 -58.6065819
C 13.4003518 4.137936 -60.4980748
O 13.9804095 3.4895145 -61.3751488
C 14.3486196 3.3947989 -58.2404563
H 14.0355429 3.0345998 -57.2602812
H 15.0443904 2.6671256 -58.661884
C 15.0998792 4.7290328 -58.0561606
H 15.5972644 4.9762276 -58.9978091
H 15.8861092 4.5829474 -57.3125827
C 14.2028056 5.9034645 -57.6227446
O 14.1451209 6.8963968 -58.3833069
O 13.5576779 5.8054743 -56.5593021
N 13.0453446 5.4126846 -60.6712948
H 12.6425479 5.9066621 -59.8759317
C 13.3632583 6.2062371 -61.8533931
H 14.1139036 5.6662568 -62.4167197
C 13.9496037 7.5757594 -61.5001384
O 13.3110616 8.3851175 -60.8299668
C 12.1357501 6.3519771 -62.752082
H 11.3121412 6.7960327 -62.1946084
H 11.8710934 5.3727228 -63.1411174
H 12.3807769 6.9994993 -63.5956822
N 15.109189 7.8745898 -62.0873356
H 15.4773862 7.2053572 -62.7563538
C 15.8432781 9.1316037 -61.9126432
H 15.2448618 9.8123746 -61.30726
C 16.0561624 9.7692783 -63.2824116
O 16.4188304 9.0839867 -64.2349318
C 17.1881392 8.8969502 -61.188147
H 17.8076121 8.2561194 -61.8169794
C 16.9764192 8.1765845 -59.8366834
H 16.3622953 8.7964619 -59.1838709
H 16.4432474 7.2414061 -60.0042166
C 17.9261826 10.2389898 -60.9959842
H 17.3004281 10.929227 -60.4298202
H 18.1645092 10.6855659 -61.9607414
H 18.8686102 10.0894265 -60.4738383
C 18.2749524 7.8099776 -59.110778
H 18.7708445 8.7035771 -58.7336403
H 18.9412565 7.2714474 -59.7855677
H 18.0370505 7.1684199 -58.2616609
N 15.8801458 11.0823937 -63.3805037
H 15.5331748 11.5872138 -62.5688742
C 15.9623305 11.817571 -64.6442913

H 16.7952455 11.4316431 -65.2351664
C 16.2306405 13.3158319 -64.4223685
O 16.001996 13.8407697 -63.3256233
C 14.6780471 11.5721577 -65.4405948
H 14.5614297 12.3592306 -66.1828294
H 14.8143697 10.6397859 -65.9771394
C 13.3981278 11.4233831 -64.5967226
H 13.2409175 12.3213791 -64.0054631
H 13.4843556 10.5777558 -63.912386
C 12.2010246 11.1445692 -65.486179
O 12.4220538 10.9836834 -66.7062945
O 11.0711677 11.0577901 -64.952999
N 16.7127389 14.0282412 -65.4533237
H 16.8577185 13.566903 -66.349382
C 16.9273486 15.4814323 -65.3898288
H 16.3871383 15.8476574 -64.5193038
C 16.3447654 16.2645858 -66.5817988
O 16.1558116 15.7419953 -67.6835179
C 18.4109936 15.8096423 -65.1287159
H 18.8261429 15.0930979 -64.4212154
H 18.4804463 16.7986531 -64.677849
C 19.2719235 15.817902 -66.3805842
O 19.9812442 14.8726451 -66.670495
N 19.2629312 16.8803734 -67.1552989
H 19.7115882 16.7659892 -68.0471681
H 18.6938739 17.6945154 -66.9492704
N 16.1171737 17.5560555 -66.3461034
H 16.3244087 17.9218972 -65.4216912
C 15.6409335 18.5213459 -67.3311437
H 15.1563779 17.9880668 -68.1515813
C 16.8207639 19.3019001 -67.9050657
O 17.814395 19.5196466 -67.2156087
C 14.6025547 19.470826 -66.7156745
H 15.0457816 19.9951369 -65.8663646
H 13.7532703 18.8943344 -66.3687152
C 14.1049765 20.4776596 -67.7549781
O 14.0497 21.6808959 -67.4357038
O 13.8939207 20.0301459 -68.9036003
N 16.6932574 19.7127438 -69.1587056
H 15.7361606 19.6535177 -69.5221682
C 17.6417525 20.4891035 -69.9664808
H 18.1709276 19.7718774 -70.5775385
C 18.7397635 21.2279062 -69.1911158
O 19.6163392 20.5303848 -68.6231238
O 18.7905354 22.4280729 -69.0989548
C 16.8603575 21.4153103 -70.9236658

H 16.3055296 22.1240006 -70.3041713
H 17.5800612 21.9809911 -71.516076
C 15.8643249 20.7213124 -71.8783228
H 15.1037594 20.1933291 -71.2956912
C 15.1458514 21.7861056 -72.7164382
H 15.8579213 22.3362843 -73.3312233
H 14.6219113 22.4788679 -72.0561909
H 14.4058411 21.3071409 -73.3609419
C 16.5435476 19.7199673 -72.8232848
H 17.329409 20.2094041 -73.398468
H 15.8002973 19.3095617 -73.5106352
H 16.9602655 18.8885803 -72.2557724

Supplementary Data 3. Cartesian coordinates for TePeNMT homology model.

N -2.1385667 21.7753992 -15.9844882
H -2.8881317 22.3451992 -15.612073
H -2.4187043 21.4412796 -16.898962
H -1.952445 20.9940613 -15.3721405
C -0.8983927 22.6008534 -16.1048954
H -1.0805973 23.3575681 -16.8709468
C 0.33849 21.7897622 -16.5659561
O 1.2553163 22.327476 -17.1874333
C -0.6053818 23.3116885 -14.7771474
H 0.3668547 23.80077 -14.8479421
H -0.5567763 22.5711725 -13.9778747
C -1.639947 24.3835703 -14.4160168
H -1.396285 24.7758699 -13.426708
H -2.6273748 23.9290305 -14.3587376
S -1.7285502 25.7740286 -15.5733039
C -2.9534829 26.7844932 -14.6928753
H -3.1342375 27.7045542 -15.2488035
H -2.5798462 27.0332981 -13.6984196
H -3.8891334 26.2323095 -14.5953778
N 0.3673335 20.4886197 -16.2657559
H -0.4109708 20.1080689 -15.7405706
C 1.3938537 19.5859753 -16.7771625
H 1.3580658 19.6103407 -17.8672226
H 2.3753087 19.9422459 -16.4680576
C 1.2496557 18.1385969 -16.3224611
O 0.7986647 17.8537127 -15.2154758
N 1.7628938 17.2390447 -17.1522057
H 2.1121366 17.5432004 -18.0501647
C 2.0288584 15.8382482 -16.8338954
H 1.9028599 15.7027836 -15.7604402
C 3.4957816 15.5076051 -17.1394012
O 4.119702 16.1812749 -17.9609391
C 0.9990926 14.9223526 -17.5032517
H 1.2572628 13.8928438 -17.2749979
H 0.0423459 15.1358366 -17.026009
C 0.8149397 15.0455348 -19.020348
H 1.6157817 14.5050359 -19.5288645
H 0.8699863 16.0954136 -19.3176114
C -0.5554566 14.4754363 -19.4180847
O -1.297864 15.2053148 -20.1119062
O -0.875195 13.3478418 -18.9747065
N 4.1061184 14.7016468 -16.2632362
H 3.5442127 14.1102083 -15.6715657
C 5.5629684 14.6122266 -16.0983634

H 6.0446756 14.8186955 -17.0600251
C 5.9695982 13.2129132 -15.6736854
O 5.3463653 12.639753 -14.786269
C 6.0594875 15.5900786 -15.0182406
H 5.5966651 15.3285324 -14.0641537
H 7.1389711 15.4636292 -14.915048
C 5.7688522 17.0627619 -15.3210483
H 6.199365 17.3109927 -16.2919241
H 4.6914859 17.2284646 -15.351451
C 6.3694499 17.9615862 -14.2310701
H 7.4516987 17.8220036 -14.192695
H 5.9514537 17.6743696 -13.2638997
C 6.0413128 19.4366002 -14.4861744
H 6.4275873 20.029751 -13.6516003
H 4.9522862 19.5499365 -14.5026758
N 6.6253332 19.9107507 -15.7656037
H 7.6331593 19.818804 -15.7546612
H 6.3843249 20.8792368 -15.933991
H 6.2683797 19.3550584 -16.5356419
N 7.106455 12.7593095 -16.1705568
H 7.4956956 13.2759729 -16.9574346
C 7.5639831 11.383838 -16.027934
H 6.7220861 10.7521143 -15.7410336
C 8.6256294 11.3080201 -14.9245199
O 9.6775993 11.9537076 -14.9983873
C 8.0743326 10.8706348 -17.3888252
H 8.9570019 11.4416081 -17.6791626
H 8.3787829 9.8302572 -17.2664366
C 7.0169986 10.9370419 -18.5172246
H 6.1405201 10.371064 -18.1914962
H 7.409044 10.4393792 -19.4038394
C 6.5974706 12.3719046 -18.896483
O 7.4785217 13.2681533 -18.9084406
O 5.3745041 12.6072441 -18.9936412
N 8.3366279 10.5700175 -13.8495991
H 7.428984 10.1185224 -13.7997984
C 9.2537472 10.4628666 -12.7165948
H 9.411715 11.4704627 -12.3299929
C 10.6243327 9.9036971 -13.153139
O 10.6986872 8.9385328 -13.9069108
C 8.6037136 9.6350831 -11.6036772
H 8.4204313 8.619799 -11.9600852
H 7.655759 10.0896643 -11.3114457
H 9.2647124 9.595986 -10.737355
N 11.703524 10.5367378 -12.6721267
H 11.5296608 11.3030954 -12.0418231

C 13.1142625 10.3142897 -13.0702562
H 13.6516325 11.146085 -12.6173586
C 13.4243279 10.5119185 -14.5675031
O 14.4681525 10.0599575 -15.0339048
C 13.7765064 9.0619071 -12.4395625
H 14.845682 9.122573 -12.6445486
C 13.6265139 9.0675566 -10.9118445
H 12.5803642 8.9423089 -10.6318339
H 14.0018322 10.0070241 -10.506304
H 14.2061243 8.2486758 -10.485005
C 13.2962742 7.7038875 -12.9652798
H 12.2890587 7.4911381 -12.6080123
H 13.9637118 6.9192992 -12.6112727
H 13.2950871 7.7094786 -14.0550769
N 12.605294 11.3349009 -15.2378421
H 11.7369915 11.5814565 -14.7805248
C 12.7314974 11.833814 -16.6111805
H 11.8724211 12.4921825 -16.7495073
C 12.6154246 10.7757957 -17.7237248
O 13.1625965 9.6728211 -17.644985
C 13.9716254 12.728998 -16.7542726
H 14.876663 12.1276992 -16.6566322
H 13.9650252 13.5017634 -15.9855672
H 13.9695782 13.2008787 -17.7373648
N 11.9102878 11.1457732 -18.7984009
H 11.4901366 12.0606529 -18.8311174
C 11.6113386 10.2161504 -19.8849453
H 11.4683386 9.2575284 -19.3922061
C 12.7697246 10.041093 -20.8749029
O 13.4841971 10.9883786 -21.2103005
C 10.2956217 10.5834444 -20.6018401
H 9.5642602 10.8737195 -19.8541547
H 10.4613373 11.4376453 -21.2598264
C 9.7172444 9.4047201 -21.4196476
H 10.350257 9.2350742 -22.2929309
H 8.7362979 9.7002982 -21.7986705
C 9.5899082 8.089336 -20.6183328
O 8.4815808 7.7830257 -20.1380566
O 10.6205886 7.3866881 -20.4498584
N 12.8901662 8.8305339 -21.4204041
H 12.2017952 8.1247666 -21.1470783
C 13.7998552 8.4972353 -22.5092701
H 14.1771403 9.4236569 -22.9434863
C 13.0399482 7.741936 -23.6088019
O 12.2666108 6.8259826 -23.3460136
C 14.9936922 7.7225667 -21.9245386

H 15.5173435 8.3745069 -21.2235556
H 14.6001376 6.8863099 -21.3570609
C 16.0029798 7.189281 -22.9591753
H 15.4878043 6.568367 -23.6890142
C 16.7306364 8.3210255 -23.6896215
H 17.2504768 8.9574053 -22.9726436
H 16.0235264 8.9250527 -24.2541046
H 17.4549132 7.90003 -24.3877077
C 17.0509741 6.3236455 -22.2584349
H 17.5894864 6.9110609 -21.5149428
H 17.7563171 5.9395483 -22.9960493
H 16.5630007 5.4766885 -21.778768
N 13.2962296 8.1160457 -24.8611726
H 13.9627613 8.8548464 -25.0049569
C 12.7297022 7.4720004 -26.0467334
H 11.6552424 7.6561114 -26.0668372
C 12.963355 5.9483119 -26.0268958
O 14.0825531 5.4964434 -25.7932219
C 13.3669937 8.1475173 -27.2671617
H 13.1403425 9.2139322 -27.2266732
H 14.4513269 8.049724 -27.187207
C 12.9656811 7.6189319 -28.6307211
C 12.1494424 8.3971049 -29.4761888
H 11.769383 9.3526089 -29.1454154
C 11.8565891 7.9520163 -30.7798108
H 11.2562024 8.555452 -31.4472005
C 12.3673248 6.7178848 -31.2374061
O 12.1160781 6.2857259 -32.4969706
H 11.5991475 6.9488647 -33.0185918
C 13.1697677 5.9340538 -30.3847744
H 13.5492799 4.9896701 -30.7459872
C 13.4827043 6.3907892 -29.091865
H 14.1273567 5.791667 -28.4621886
N 11.8951892 5.1788313 -26.2599184
H 11.0304658 5.6427895 -26.4800882
C 11.8426092 3.7036502 -26.2137578
H 10.7811114 3.4599108 -26.2631227
C 12.3180866 3.0493723 -24.893297
O 12.588105 1.8486882 -24.8229072
C 12.4769853 3.1028593 -27.4818833
H 13.5610504 3.0598399 -27.3602348
H 12.2582179 3.7523311 -28.3311342
C 11.9188234 1.7098089 -27.8129528
O 10.6734795 1.5608478 -27.7641107
O 12.7303627 0.8270589 -28.169355
N 12.3786583 3.8077385 -23.7892188

H 12.1567416 4.7960764 -23.8565606
C 12.7236866 3.2518266 -22.4780661
H 13.5786598 2.5902866 -22.6181944
C 11.5733328 2.4149249 -21.9209309
O 10.5345585 2.9233827 -21.5059758
C 13.1586237 4.3717878 -21.5322934
H 14.0067916 4.8753226 -21.9927235
H 12.3398627 5.0838206 -21.4074609
C 13.5708913 3.8411374 -20.1519667
H 12.6903613 3.4453154 -19.6452956
H 14.3110593 3.0473288 -20.2626016
C 14.1595742 4.9730826 -19.3074994
H 13.477531 5.8273082 -19.3386531
H 15.1255757 5.265841 -19.7198963
C 14.3246592 4.5251755 -17.8558798
H 14.9647772 3.6393135 -17.8237746
H 13.3343658 4.2459127 -17.4769636
N 14.894404 5.6206571 -17.034387
H 15.8026652 5.9022239 -17.3743342
H 14.9585333 5.3479973 -16.0632593
H 14.281514 6.4323719 -17.0835563
N 11.8145313 1.1129027 -21.796752
H 12.636759 0.7428888 -22.2469702
C 10.915173 0.206206 -21.075752
H 9.9261211 0.3116122 -21.5233934
C 10.7879049 0.6022152 -19.5986625
O 11.7565775 1.0128309 -18.9601126
C 11.3345322 -1.270057 -21.22834
H 10.6296722 -1.8783407 -20.6606041
C 11.2418011 -1.7098514 -22.6941182
H 11.9608367 -1.1650701 -23.3087444
H 10.2395993 -1.5174528 -23.0780593
H 11.4468521 -2.7773516 -22.7753339
C 12.7458793 -1.5768076 -20.7095885
H 13.4907476 -1.0077613 -21.2656222
H 12.9570051 -2.6395233 -20.8284199
H 12.8214824 -1.3296274 -19.6506744
N 9.6090573 0.3641778 -19.0190877
H 8.8636369 0.042936 -19.61778
C 9.3030641 0.4995601 -17.5765515
H 8.2498576 0.2378134 -17.4888618
C 9.3464409 1.9102799 -16.9589934
O 9.2888891 2.018324 -15.7386553
C 10.0341763 -0.545724 -16.7016046
H 9.6693134 -0.4676256 -15.6770594
C 9.7794235 -1.9797159 -17.169852

H 10.215899 -2.1450359 -18.1540049
H 8.7079161 -2.1740235 -17.2032576
H 10.239578 -2.6699839 -16.4624216
O 11.4299275 -0.3613229 -16.691399
H 11.6667592 0.1678767 -17.4727227
N 9.2779939 2.9909397 -17.7450031
H 9.3809 2.8980378 -18.7455964
C 8.8924366 4.3186524 -17.2226461
H 9.484174 4.5459203 -16.3361664
C 7.3950813 4.3363773 -16.8420819
O 6.54833 4.1563548 -17.7157283
C 9.1737252 5.4060425 -18.2724269
H 8.8412563 6.3707971 -17.8859208
H 8.6096819 5.1895781 -19.1816057
O 10.5517975 5.4957348 -18.5958262
H 10.5894134 6.1754387 -19.3178923
N 7.0367162 4.4791013 -15.5515566
H 7.7637049 4.4744686 -14.8485576
C 5.6335974 4.372326 -15.0850094
H 5.0070584 4.6318068 -15.9400818
C 5.0839267 5.3356751 -13.9496445
O 4.1802507 4.8921545 -13.2441249
C 5.3501324 2.8742188 -14.7954481
H 5.6601937 2.2734319 -15.6495461
H 4.2770818 2.7385852 -14.6680957
C 6.0481724 2.3356799 -13.5563096
O 6.9355157 2.9397106 -12.9802825
N 5.6839717 1.1536912 -13.1130988
H 6.1721809 0.8278604 -12.2984982
H 4.9434468 0.6468731 -13.5561149
N 5.4209234 6.6581869 -13.7954432
H 6.1313111 7.0377768 -14.4037847
C 4.6727308 7.6690857 -12.8730958
H 3.6722803 7.2716519 -12.7031805
H 5.1665915 7.6425688 -11.902207
C 4.4605241 9.2667223 -13.2399755
O 5.4811072 9.6663177 -13.812121
N 3.2536409 10.1109433 -12.9872607
H 2.6474233 9.6614132 -12.3206553
C 2.3771526 11.4343133 -13.6922496
H 3.1658733 12.0715482 -14.0792353
C 1.0505375 12.8198719 -12.978889
O 0.738086 12.1140384 -12.0071596
C 1.9540762 10.4352165 -14.9544058
H 0.9329988 10.1097298 -14.746017
C 2.7417697 9.1177858 -15.5124699

H 3.7821969 9.3932306 -15.6797871
H 2.6711718 8.3582525 -14.7352443
C 1.9181844 11.302737 -16.1856385
H 2.9290002 11.590086 -16.4899791
H 1.3394983 12.1952761 -15.9637221
H 1.4171187 10.8031861 -17.0150758
C 2.3461044 8.3062146 -16.7841532
H 1.2838958 8.043668 -16.7411571
H 2.9411495 7.3919643 -16.8207085
H 2.5328117 8.8854332 -17.695951
N 0.2333932 14.4003043 -13.1834479
H 0.7437184 14.7653437 -13.9704046
C -1.2123799 15.955379 -12.9597387
H -1.0028175 16.3292374 -13.960482
C -2.5312399 15.1695335 -13.1010222
H -2.3258053 14.1092915 -13.2484796
H -3.0618878 15.2689053 -12.1555772
C -3.4175086 15.6977339 -14.2585728
H -3.2432872 16.7657087 -14.3985759
C -3.0783778 14.9714564 -15.5675978
H -3.2811317 13.903087 -15.4809576
H -2.0269104 15.1037593 -15.8111044
H -3.6757972 15.3801559 -16.3812018
C -4.9110054 15.512951 -13.9555661
H -5.1400087 14.4586235 -13.8201664
H -5.5040244 15.9052164 -14.7807417
H -5.172629 16.0607214 -13.0540348
C -2.0596333 17.9254177 -12.3430272
O -1.3832877 18.2475852 -11.374691
N -3.271554 19.2249776 -12.5340521
H -3.8017849 18.9469697 -13.3433045
C -4.1853409 20.9390259 -11.9780666
H -4.1201797 20.8378689 -10.8941409
C -5.8142116 21.9798425 -12.0685304
O -6.4577917 21.990306 -13.1238488
C -3.3553264 22.2522131 -12.3368714
H -2.7508435 21.9810248 -13.199742
H -3.9431639 23.1195955 -12.6376299
C -2.3935577 22.6639575 -11.2141059
H -1.5875927 23.2551301 -11.6539897
H -1.9417954 21.7737149 -10.7710014
C -3.1027948 23.4921229 -10.1365626
O -4.1338848 23.0043489 -9.6189633
O -2.6283562 24.6174618 -9.8712537
N -6.4475046 23.0719258 -11.2698861
H -5.8681384 23.316696 -10.4654281

C -7.9445496 23.8116708 -11.2481781
H -8.5313975 23.1447577 -11.8786809
C -8.4505552 25.3183528 -11.7282815
O -7.9562339 26.3541144 -11.2608184
C -8.5827225 23.7319299 -9.8142739
H -8.3098143 22.7644516 -9.3910452
H -8.1237068 24.4929663 -9.1820639
C -10.1128287 23.8553926 -9.699581
H -10.4295997 24.8665053 -9.9608487
H -10.3884539 23.7049408 -8.6526486
C -10.8425833 22.8299823 -10.5754585
O -10.9583405 23.1023724 -11.793048
O -11.2574013 21.7859035 -10.0265513
N -9.602907 25.5151302 -12.4609812
H -10.1970189 24.6947314 -12.6038089
C -10.0093518 26.7968804 -13.139427
H -9.6033406 27.6153906 -12.5465371
C -11.5373224 27.0770783 -13.3129338
O -12.3430523 26.1660776 -13.4651874
C -9.3158021 26.7963794 -14.5218177
H -8.2652178 26.5407625 -14.3723088
H -9.7616841 25.9988297 -15.1187812
C -9.3656862 28.1006173 -15.3396433
H -10.3980501 28.3527717 -15.5746801
C -8.7151959 29.2767986 -14.6053596
H -7.6855865 29.0272619 -14.3458923
H -9.2616884 29.5103425 -13.6946444
H -8.721151 30.1580805 -15.2468748
C -8.6202634 27.902131 -16.6598573
H -7.5714266 27.6723762 -16.4704162
H -8.6904418 28.808201 -17.2612677
H -9.0736317 27.0808258 -17.214682
N -11.9412789 28.3572898 -13.4297045
H -11.2467902 29.0828588 -13.3712445
C -13.3321225 28.7589476 -13.708797
H -13.9922764 28.0787705 -13.1669539
C -13.6973559 28.668285 -15.20173
O -13.2212429 29.4599915 -16.0166546
C -13.5976231 30.1744778 -13.1694646
H -13.3339687 30.2032292 -12.1112647
H -12.9526566 30.8857408 -13.6884859
C -15.0439728 30.6091374 -13.3172145
C -16.0007793 30.2075446 -12.3654889
H -15.7026398 29.6095012 -11.5164327
C -17.3497155 30.5734271 -12.5229045
H -18.084102 30.2600018 -11.7942258

C -17.7459906 31.3401123 -13.632773
H -18.784204 31.6156339 -13.7569758
C -16.7930155 31.742311 -14.5848431
H -17.0984245 32.326439 -15.4416514
C -15.4440982 31.3771052 -14.4276294
H -14.7165289 31.6734974 -15.1713742
N -14.6326318 27.780819 -15.5396291
H -14.9521194 27.1150618 -14.8471413
C -15.2390964 27.6886577 -16.8666391
H -15.735075 28.6312197 -17.0994544
H -14.4624243 27.5134405 -17.6111349
C -16.2666536 26.5596064 -16.957317
O -16.3953891 25.7524785 -16.0401148
N -16.9764725 26.4878918 -18.0819378
H -16.8289854 27.176356 -18.8044725
C -17.9575033 25.4408391 -18.3831007
H -17.7899232 24.5827215 -17.7304426
C -17.7792012 24.9927546 -19.8397808
O -17.7959146 25.8188285 -20.750926
C -19.3742105 25.9740925 -18.1133224
H -19.4072767 26.3626889 -17.0943744
H -19.5911057 26.7896238 -18.8053619
C -20.4418095 24.8784784 -18.2588574
H -20.2170614 24.0779836 -17.5490798
H -20.3816661 24.4589508 -19.2655484
C -21.873747 25.3850101 -18.0153021
O -22.0329252 26.4946208 -17.4568844
O -22.8044396 24.6367793 -18.3888393
N -17.5576422 23.6953514 -20.0649298
H -17.6282623 23.0479037 -19.293315
C -17.3269048 23.1256682 -21.396853
H -17.6295227 23.8524687 -22.1539443
C -18.173276 21.8705255 -21.6133325
O -18.4492201 21.1344351 -20.6665456
C -15.8303326 22.833808 -21.5971231
H -15.5031065 22.1074362 -20.8521036
H -15.2664698 23.755845 -21.4523805
C -15.5235885 22.3038595 -22.9769314
N -16.0573017 22.8083599 -24.1656877
C -15.6125519 22.0028993 -25.140073
H -15.854249 22.1181749 -26.1888167
N -14.8449621 21.02673 -24.6277346
H -14.429788 20.2755673 -25.1579691
C -14.7816493 21.1953241 -23.2621255
H -14.2791179 20.561661 -22.5447457
N -18.5185024 21.5818415 -22.8701808

H -18.1432063 22.1738025 -23.6009505
C -19.2288161 20.3661674 -23.257331
H -19.9116933 20.1067755 -22.4457962
C -18.2431347 19.2015468 -23.4437471
O -17.5034608 19.1367934 -24.4310812
C -20.0753688 20.6536823 -24.5101802
H -20.7622502 21.4721288 -24.2876809
H -19.4111275 20.9769363 -25.3134978
C -20.8899068 19.4416545 -25.0031907
H -20.2061397 18.632868 -25.252551
C -21.8900833 18.9305006 -23.9653095
H -22.5664824 19.7301243 -23.6638583
H -21.3716057 18.543429 -23.0898099
H -22.4721546 18.1090986 -24.387707
C -21.6558409 19.8178908 -26.2704486
H -22.3670983 20.6156827 -26.0549008
H -22.1966695 18.9460329 -26.6408658
H -20.9593117 20.149503 -27.0399621
N -18.305586 18.2264973 -22.5363312
H -18.9848484 18.3084584 -21.7879638
C -17.4987295 17.0022879 -22.56464
H -16.4445225 17.2767669 -22.5352472
C -17.7136686 16.1851165 -23.8479637
O -16.7324037 15.7061853 -24.4089253
C -17.8183622 16.1676056 -21.3118248
H -17.6392762 16.7679502 -20.4189096
H -17.1541192 15.3031549 -21.279748
C -19.2469521 15.6902349 -21.2993199
N -20.3555822 16.5291505 -21.1770985
C -21.409785 15.8002387 -21.5780685
H -22.404937 16.1941265 -21.7444035
N -21.0189659 14.5690479 -21.940586
H -21.5461555 13.999855 -22.611493
C -19.6585309 14.4792161 -21.7690455
H -19.0246639 13.6887216 -22.1457658
N -18.9342631 16.1416122 -24.3967885
H -19.7139306 16.4659898 -23.8383305
C -19.2306073 15.4622993 -25.6698397
H -18.9191364 14.4191048 -25.5943322
C -18.4740459 16.0915514 -26.8531044
O -17.994577 15.3816032 -27.732199
C -20.7422481 15.4827005 -25.9605596
H -20.9140981 15.0341016 -26.9402223
H -21.0845436 16.5175317 -26.0055241
C -21.5715667 14.7083567 -24.9337949
O -21.1731487 13.5796127 -24.57134

O -22.5721805 15.2767049 -24.4473625
N -18.2496141 17.4112082 -26.8323886
H -18.5716361 17.9329869 -26.0288096
C -17.4531297 18.1047217 -27.8489587
H -17.7977212 17.8135617 -28.8424323
H -17.5879439 19.1789559 -27.7304308
C -15.956921 17.7942072 -27.7454374
O -15.2888172 17.6280449 -28.7627028
N -15.439025 17.6201509 -26.5247796
H -16.0609943 17.7043393 -25.7294654
C -14.0743246 17.1266898 -26.3031017
H -13.3826248 17.7038566 -26.9186169
C -13.9256189 15.6534948 -26.724003
O -12.9564171 15.2990751 -27.3953963
C -13.702554 17.3422062 -24.8291262
H -13.7609572 18.4063683 -24.6038623
H -14.431762 16.8391893 -24.1953113
C -12.3186415 16.8515113 -24.4543145
C -11.189853 17.6482056 -24.7279804
H -11.3040053 18.609059 -25.2098121
C -9.9051194 17.1911998 -24.3777829
H -9.0312386 17.792005 -24.5808524
C -9.7460546 15.9368061 -23.7535044
O -8.5019852 15.5094553 -23.4137942
H -8.5099345 14.6569228 -22.9779248
C -10.8775819 15.1370117 -23.4808996
H -10.7593813 14.1747477 -23.0085044
C -12.161335 15.5959257 -23.8337612
H -13.0275541 14.9793858 -23.6353157
N -14.9268359 14.8201123 -26.423841
H -15.7014839 15.176822 -25.8741997
C -14.9828394 13.4197593 -26.8408227
H -14.0769896 12.9181517 -26.4986893
C -15.0310294 13.2711907 -28.3685764
O -14.316041 12.4302505 -28.9112801
C -16.1878629 12.7409781 -26.1724631
H -16.0987507 12.8425083 -25.0898935
H -17.1015123 13.2506904 -26.4752072
C -16.3194695 11.2716038 -26.5208461
C -15.679925 10.2960364 -25.7306851
H -15.1296517 10.5903453 -24.8487413
C -15.7427155 8.9385754 -26.1018896
H -15.2470437 8.1817352 -25.5143806
C -16.4327316 8.5582085 -27.2723703
O -16.4601685 7.2539103 -27.6505408
H -16.8895214 7.1591939 -28.5014005

C -17.087672 9.5346431 -28.0532165
H -17.603424 9.2540987 -28.9595907
C -17.035941 10.887789 -27.6716777
H -17.5222426 11.6367727 -28.2851494
N -15.7781968 14.1209507 -29.0785084
H -16.4213335 14.735133 -28.58639
C -15.8302485 14.0991621 -30.5458371
H -16.023302 13.0723458 -30.857743
C -14.4935853 14.5210283 -31.1785911
O -13.967663 13.81329 -32.0333803
C -16.994066 14.9750367 -31.0424011
H -17.90762 14.6865268 -30.519727
H -16.7819786 16.0183928 -30.8031416
C -17.238316 14.8508303 -32.5555673
H -16.3290961 15.1177248 -33.0992785
H -18.0038016 15.5779021 -32.8375267
C -17.6986865 13.4395358 -32.9540066
O -18.9264795 13.2539991 -33.097649
O -16.8225467 12.5548429 -33.0888592
N -13.837982 15.5729289 -30.6701081
H -14.2892367 16.1201696 -29.9440593
C -12.4898729 15.9648231 -31.1351682
H -12.5346458 16.1337827 -32.2113024
C -11.4694612 14.8360737 -30.9194148
O -10.6875397 14.5295424 -31.8238719
C -12.0510367 17.2817301 -30.4618222
H -12.2135606 17.2075124 -29.385962
C -10.5745282 17.6261432 -30.7001145
H -10.360449 17.6415137 -31.7693089
H -9.9326989 16.8899406 -30.2164525
H -10.3471552 18.6032195 -30.2737645
C -12.8768666 18.454303 -31.009272
H -12.6844 18.5843449 -32.0745506
H -12.6143381 19.3701809 -30.4802161
H -13.9403605 18.2682826 -30.8668087
N -11.5418689 14.1369177 -29.7810758
H -12.2068198 14.4377171 -29.0741423
C -10.7457172 12.934077 -29.5229422
H -9.6880299 13.1653715 -29.6509979
H -10.9128238 12.6231638 -28.4919327
C -11.1015855 11.7534031 -30.4368387
O -10.2052046 11.0525872 -30.9091058
N -12.3806552 11.5703557 -30.7665667
H -13.0669852 12.1981408 -30.3596135
C -12.8830576 10.4904933 -31.6335773
H -12.4536585 9.5465298 -31.299618

C -12.4546029 10.6930136 -33.0856704
O -11.8379689 9.7967565 -33.662114
C -14.4115139 10.3664975 -31.5108397
H -14.8837716 11.3195435 -31.7430037
C -15.0075931 9.281508 -32.405127
H -14.497612 8.3332624 -32.2385487
H -14.9184434 9.5764776 -33.4497101
H -16.0681325 9.1738969 -32.1753859
O -14.7191285 9.9990169 -30.1858573
H -14.6611214 10.8041515 -29.6445041
N -12.6061399 11.9050074 -33.6278135
H -13.1277568 12.5991368 -33.0943308
C -12.1108057 12.2899864 -34.96069
H -12.5626445 11.6330904 -35.7039491
C -10.5924574 12.1086371 -35.0606404
O -10.1118234 11.5017037 -36.0194828
C -12.5253388 13.7419652 -35.2846723
H -12.2047179 14.3950538 -34.4716908
C -11.9014053 14.2583629 -36.5896139
H -12.1485647 13.587198 -37.4129321
H -10.8182307 14.3273054 -36.4922998
H -12.2846337 15.2548982 -36.8119629
C -14.0473735 13.8569901 -35.4397281
H -14.390893 13.2481522 -36.2760117
H -14.3288191 14.8958381 -35.6094773
H -14.5551193 13.5168148 -34.5364626
N -9.8327613 12.5183483 -34.0376294
H -10.2691124 13.0158676 -33.2677802
C -8.3866883 12.2947826 -33.9964652
H -7.94803 12.713808 -34.9032965
C -8.0253687 10.7951613 -33.964585
O -7.1750255 10.3535353 -34.7385592
C -7.808095 13.0498299 -32.7940855
H -8.2322251 12.6640796 -31.8665257
H -8.0425363 14.1119177 -32.8768195
H -6.7249933 12.9258191 -32.7693519
N -8.7383063 9.9928143 -33.1690346
H -9.4399586 10.4216383 -32.5757625
C -8.5261026 8.5373236 -33.0519002
H -7.476906 8.3564513 -32.8190178
C -8.836892 7.7959867 -34.3557814
O -8.0721417 6.9214099 -34.7582541
C -9.3671494 7.960609 -31.9009351
H -10.4270834 8.1175921 -32.1029293
C -9.1210937 6.4714297 -31.6602582
H -8.0569718 6.2924769 -31.5037895

H -9.4663804 5.8883818 -32.5134126
H -9.6722286 6.1524028 -30.7752524
O -9.0287859 8.6085484 -30.6970051
H -9.4300372 9.4946216 -30.711502
N -9.8765834 8.2071453 -35.0894348
H -10.4751493 8.9342993 -34.7066042
C -10.218649 7.6484065 -36.4087586
H -10.3078933 6.565189 -36.3149444
C -9.0961985 7.9031619 -37.4291875
O -8.8107229 7.0319327 -38.2457573
C -11.5883493 8.2058925 -36.8724934
H -11.5800876 9.2895272 -36.7418409
C -11.8649231 7.9080697 -38.3605028
H -11.8182208 6.8336763 -38.5433383
H -11.1330206 8.4106419 -38.9928817
H -12.8465962 8.2794081 -38.6492608
C -12.7194695 7.6055139 -36.002287
H -12.8725628 6.5606242 -36.275951
H -12.4291906 7.6247014 -34.9527788
C -14.0547499 8.3529914 -36.1158443
H -14.469557 8.2558855 -37.1180749
H -13.9111854 9.4081356 -35.8800284
H -14.765007 7.9268607 -35.4065953
N -8.3937191 9.0388911 -37.3367375
H -8.6846259 9.7176446 -36.6427142
C -7.265239 9.3812153 -38.2229573
H -7.5533014 9.1179589 -39.2408252
C -5.9497281 8.6286735 -37.9283472
O -4.9741865 8.7465544 -38.6840646
C -7.0390962 10.899473 -38.1899562
H -8.0014138 11.4136505 -38.2093944
H -6.4800146 11.1924024 -39.0771632
O -6.2990547 11.3175345 -37.05805
H -6.6658562 10.914714 -36.2473093
N -5.8900761 7.8921141 -36.8100548
H -6.7249579 7.8243468 -36.2397125
C -4.6565738 7.3302429 -36.256341
H -3.8362244 8.0007177 -36.5123651
C -4.2800623 5.9408626 -36.8074929
O -3.1352983 5.5205919 -36.6491747
C -4.7709993 7.3240925 -34.7269169
H -5.5236596 6.6026775 -34.4107831
H -5.0474193 8.3167635 -34.3677705
H -3.8122191 7.0434425 -34.2890576
N -5.1800992 5.2659515 -37.5310566
H -6.0659794 5.7270073 -37.7010793

C -4.9863126 3.9392569 -38.1512939
H -5.0038412 3.1815968 -37.3671395
C -3.6467069 3.7571432 -38.8886477
O -2.9731921 2.7386014 -38.7264792
C -6.1697208 3.6740353 -39.1048049
H -7.0402153 3.4328757 -38.4935022
H -5.9499646 2.7970377 -39.7150907
C -6.5637775 4.8197352 -40.0201111
N -5.7699764 5.9207787 -40.3641479
C -6.5674691 6.752385 -41.049963
H -6.277414 7.7318812 -41.4028678
N -7.7926363 6.2244398 -41.1826337
H -8.5854946 6.6964794 -41.5940588
C -7.8129371 5.0107498 -40.5332027
H -8.6720512 4.3747877 -40.3736965
N -3.1845526 4.7824287 -39.6094995
H -3.8397485 5.5490973 -39.7402001
C -1.9094941 4.7790629 -40.3503428
H -1.9058746 3.9210138 -41.0259016
C -0.6755178 4.6000095 -39.4590681
O 0.3500762 4.1523355 -39.9634635
C -1.8108003 6.0655976 -41.1818839
H -1.9802055 6.9149351 -40.5182176
H -0.8101594 6.155225 -41.6088882
C -2.8366963 6.0737128 -42.3295833
H -3.7805566 5.6467642 -41.9998432
H -2.4675716 5.4691415 -43.158521
C -3.090666 7.5062857 -42.7975055
H -2.21557 7.8834898 -43.328123
H -3.2604295 8.1357064 -41.9227019
N -4.2898586 7.6146451 -43.6353146
H -5.0985667 8.0752973 -43.2163844
C -4.3632322 7.5927761 -44.9465972
N -3.4118636 7.0991039 -45.680707
H -3.2172373 7.5904414 -46.5573116
H -2.6560279 6.644648 -45.2205878
N -5.4028833 8.1065841 -45.5234574
H -6.0523492 8.6180683 -44.9202338
H -5.4423454 8.1830156 -46.5185052
N -0.7546542 4.8641941 -38.1528525
H -1.6478001 5.1514024 -37.7665882
C 0.3441051 4.6164829 -37.2169392
H 1.2323201 5.1264964 -37.5885598
C 0.6996833 3.1210416 -37.0906775
O 1.8637562 2.7984179 -36.8587498
C -0.0133202 5.2297426 -35.858726

H -0.9017486 4.748926 -35.4486878
H -0.2065543 6.2971976 -35.9704315
H 0.8169899 5.0909097 -35.1647239
N -0.2423564 2.2117153 -37.3765593
H -1.1831707 2.5200405 -37.5942453
C 0.0441412 0.7786215 -37.4595675
H 0.5673747 0.4724402 -36.5515695
C 0.9552045 0.4341165 -38.6550826
O 1.7906982 -0.4590169 -38.5465271
C -1.2872208 0.0221727 -37.522223
H -1.8167315 0.2672544 -38.4447155
H -1.9079066 0.2944651 -36.668599
H -1.1024213 -1.0504495 -37.4916568
N 0.8781965 1.1977947 -39.7544587
H 0.2102942 1.9555281 -39.7611379
C 1.8112123 1.0873253 -40.8905783
H 2.0039892 0.0301488 -41.0804667
C 3.1532747 1.7347408 -40.5479506
O 4.1918602 1.1211358 -40.7786454
C 1.2242149 1.7004207 -42.1796402
H 1.0712918 2.7691655 -42.0362597
C 2.1680245 1.5163423 -43.3761676
H 2.3959256 0.458749 -43.5151264
H 3.0968456 2.0616945 -43.2111974
H 1.7047064 1.9050683 -44.2829471
C -0.1270984 1.0684292 -42.5415657
H -0.0134202 -0.0094525 -42.6618157
H -0.4995357 1.4948188 -43.4727939
H -0.8607535 1.2658962 -41.7604837
N 3.1497115 2.9075864 -39.8983128
H 2.2584563 3.3646896 -39.7477886
C 4.3829764 3.5769137 -39.4271644
H 5.0316617 3.7532182 -40.287047
C 5.1714679 2.6870662 -38.4572727
O 6.3914124 2.6466122 -38.5342007
C 4.0854947 4.9447377 -38.7734971
H 3.4492565 4.7868126 -37.9038288
C 5.3579813 5.667574 -38.3077099
H 6.0473034 5.7700222 -39.1479344
H 5.8520541 5.0988091 -37.5198768
H 5.1091859 6.6515776 -37.9116408
C 3.3657123 5.9094878 -39.7292705
H 4.0142422 6.146524 -40.5715909
H 3.1117469 6.8290265 -39.2030367
H 2.4524687 5.4645623 -40.1099666
N 4.5099699 1.8691594 -37.6261136

H 3.501018 1.9661057 -37.5679173
C 5.1768327 0.8861925 -36.7517641
H 5.9189905 1.4157616 -36.1524491
C 5.9655105 -0.1805465 -37.5278367
O 6.9655235 -0.6580613 -37.005066
C 4.1151826 0.2654933 -35.821984
H 3.6872668 1.0584642 -35.2070059
H 3.3115988 -0.1409395 -36.4366679
C 4.6026419 -0.8663175 -34.8987054
H 4.9701494 -1.6946528 -35.5041773
H 3.7425868 -1.2253198 -34.3369632
C 5.691348 -0.4487287 -33.8979481
H 5.305648 0.3329929 -33.2411631
H 6.5347315 -0.0299299 -34.4515579
N 6.1852786 -1.5988657 -33.1065998
H 7.0302672 -2.023504 -33.4565587
C 5.6931212 -2.0929878 -31.9796119
N 4.6137617 -1.6238221 -31.4121932
H 4.2496792 -2.0315972 -30.5644291
H 4.1500759 -0.8388203 -31.8268032
N 6.2826626 -3.0960025 -31.392016
H 7.158683 -3.4460252 -31.7437805
H 5.9220268 -3.4384356 -30.5210906
N 5.5130667 -0.5786026 -38.7196586
H 4.6989518 -0.1098716 -39.0946508
C 6.155844 -1.6215128 -39.5477068
H 6.7171868 -2.2989085 -38.9044692
C 7.1870424 -1.0471198 -40.5141252
O 8.1672071 -1.7115576 -40.8379074
C 5.0729423 -2.429844 -40.2970744
H 4.4719049 -1.7392024 -40.8919679
C 5.6505611 -3.4778044 -41.2668926
H 6.3652394 -4.1194898 -40.7503349
H 6.1577817 -2.9777053 -42.0922651
H 4.8537515 -4.0898952 -41.6888069
C 4.1272498 -3.1381766 -39.3001663
H 3.4521743 -2.3981917 -38.8745679
H 3.519856 -3.8567523 -39.8472324
C 4.8022508 -3.8790266 -38.1377464
H 5.1620025 -3.1674568 -37.3956499
H 5.6444629 -4.4611102 -38.5038849
H 4.0797422 -4.5405924 -37.6627072
N 6.9634562 0.1827065 -40.9660073
H 6.1059518 0.645179 -40.6903566
C 7.8918005 0.9019409 -41.8307362
H 8.3560989 0.1968688 -42.5199967

C 9.0541694 1.5083209 -41.0392823
O 10.1751688 1.4175308 -41.5113396
C 7.0910835 1.9372281 -42.6425295
H 6.4755174 2.5141194 -41.9494156
C 8.015055 2.9405889 -43.3353853
H 8.8170607 2.4130698 -43.8517664
H 8.4436482 3.6079035 -42.5859543
H 7.4537262 3.5413332 -44.0376991
C 6.1329575 1.2547909 -43.6511673
H 5.3388 0.7519175 -43.1009127
H 5.6559528 2.0240523 -44.2518133
C 6.7607953 0.2206656 -44.5975052
H 7.0017951 -0.6915304 -44.0477814
H 7.6692296 0.6123557 -45.0487245
H 6.0481605 -0.0291608 -45.3837918
N 8.798992 1.9819869 -39.8195457
H 7.8269675 1.9509433 -39.5448266
C 9.6149082 2.8267209 -38.9312898
H 9.2170332 2.5797365 -37.9459199
C 9.3250434 4.3409519 -39.0306445
O 8.5238253 4.7993794 -39.8542185
C 11.099113 2.4020543 -38.8279642
H 11.7082873 3.1408157 -39.3434734
H 11.2510327 1.4269978 -39.2904088
C 11.5965131 2.3038259 -37.3833269
O 11.4492724 3.3205405 -36.6703128
O 12.1249463 1.2330349 -37.0094698
N 9.8588958 5.1058401 -38.0754761
H 10.516748 4.6466534 -37.4396315
C 9.3325992 6.4041998 -37.6512809
H 8.3526531 6.2042132 -37.2199303
C 9.1395831 7.4234129 -38.7896545
O 10.0494829 7.7492656 -39.5515223
C 10.2024572 6.9760756 -36.5200309
H 10.3373002 6.2059069 -35.7593743
H 11.1814432 7.2466722 -36.9189787
C 9.5601829 8.2096529 -35.8657287
H 8.5933201 7.9247972 -35.443284
H 9.3792816 8.9717041 -36.6261681
C 10.4478246 8.8008571 -34.7647909
O 10.8020372 8.0456266 -33.8314376
O 10.7428137 10.012847 -34.8410345
N 7.9356494 8.0039627 -38.8202964
H 7.2747294 7.6949909 -38.1276181
C 7.4979327 9.066309 -39.7246913
H 6.4475177 9.2219085 -39.4784392

C 7.4607978 8.7250306 -41.2328113
O 7.2182063 9.6195186 -42.0501456
C 8.189382 10.3821855 -39.339387
H 9.2533861 10.3291555 -39.5703739
H 8.0772996 10.5585665 -38.2684137
H 7.7299772 11.2135753 -39.8729389
N 7.5456185 7.4423073 -41.6155564
H 7.7541 6.7354562 -40.9135248
C 7.344726 6.9801426 -42.997353
H 6.9802142 7.8289962 -43.5714683
C 6.2228111 5.9398072 -43.1423331
O 5.8136015 5.298277 -42.1811172
C 8.7040589 6.5417867 -43.5937623
H 9.3769214 6.2789443 -42.781539
H 8.5857406 5.6690598 -44.2357264
C 9.2911549 7.7100855 -44.425368
H 9.1907816 8.6410518 -43.8675988
C 10.7676023 7.5379856 -44.7641775
H 10.9094523 6.6899523 -45.4326336
H 11.347273 7.4053009 -43.8535032
H 11.1408564 8.4293545 -45.2677901
C 8.5546316 7.8484516 -45.7653518
H 8.6226347 6.9085588 -46.3141622
H 9.0244539 8.6267692 -46.3653165
H 7.5122898 8.121164 -45.6183874
N 5.6747873 5.8134553 -44.3622881
H 6.051826 6.3868802 -45.1032308
C 4.5891328 4.8649351 -44.6980872
H 4.5631062 4.1139725 -43.9051263
C 4.8447935 4.0459821 -45.973334
O 3.9017478 3.6498304 -46.6520897
C 3.2105171 5.5603428 -44.666108
H 2.4533252 4.7738203 -44.6831793
H 3.1054279 6.0864467 -43.7153432
C 2.9161759 6.5454592 -45.8179066
H 3.1261552 6.0697121 -46.7735315
H 3.5448542 7.4315362 -45.7241825
C 1.4308664 6.9484155 -45.7776237
H 1.2590212 7.5693356 -44.8966521
H 0.8212512 6.0482975 -45.6740764
N 0.981203 7.6811794 -46.9780385
H 0.7524881 8.6673509 -46.8923419
C 0.6228584 7.177358 -48.1443321
N 0.8186153 5.929421 -48.4685675
H 0.5752193 5.6132175 -49.3870961
H 1.388143 5.3577715 -47.8677134

N 0.0406873 7.949872 -49.0092621
H -0.2933387 8.855375 -48.6610897
H -0.2623347 7.6163466 -49.9005672
N 6.1190758 3.8578277 -46.3209588
H 6.8301691 4.1363819 -45.6635574
C 6.5958218 3.2428633 -47.5626859
H 5.830005 2.5719029 -47.9542416
C 7.8916732 2.4367347 -47.343168
O 8.5668267 2.64766 -46.3401455
C 6.8674228 4.3491416 -48.5851797
H 7.7970464 4.8464069 -48.304794
H 7.032437 3.8770175 -49.5533758
C 5.793543 5.4093771 -48.7466088
C 4.5694425 5.071977 -49.3430479
H 4.3617759 4.0434651 -49.5976387
C 3.6563468 6.0786253 -49.6922932
H 2.7837729 5.8310304 -50.2772817
C 3.9157208 7.4151039 -49.3467661
H 3.2349086 8.196869 -49.6514519
C 5.0976506 7.7468283 -48.6644446
H 5.3156042 8.7854837 -48.4512446
C 6.0470079 6.7471097 -48.3865945
H 6.9997683 7.0245668 -47.966154
N 8.2414517 1.5676536 -48.3001291
H 7.6597218 1.5224626 -49.118932
C 9.3979198 0.6578697 -48.2914649
H 9.1016447 -0.2646165 -47.791358
C 10.6602547 1.1969945 -47.5742744
O 11.1786285 2.245083 -47.9427162
C 9.7087369 0.3224341 -49.75895
H 9.967396 1.234773 -50.3010873
H 8.8483582 -0.1504811 -50.2328802
H 10.5608699 -0.3531544 -49.8204047
N 11.1058984 0.4683517 -46.5404187
H 10.6020477 -0.3756197 -46.3181145
C 12.3498292 0.6411544 -45.7620579
H 12.0842348 0.2321049 -44.78769
C 12.7677959 2.0582833 -45.3295639
O 13.9257997 2.2966503 -44.965395
C 13.488643 -0.2542563 -46.2966031
H 14.4106292 0.3125754 -46.3885813
H 13.233996 -0.5667301 -47.3062372
C 13.7404825 -1.5012609 -45.4212878
O 12.782663 -1.9446545 -44.7479203
O 14.8558588 -2.0616043 -45.4835954
N 11.7676098 2.9221305 -45.1175289

H 10.8663841 2.6705479 -45.5032949
C 11.8434355 4.1137479 -44.2565654
H 10.9702733 4.7131008 -44.4953178
C 13.0474841 5.0013958 -44.5690478
O 13.2566867 5.4146228 -45.7033659
C 11.6839069 3.7215239 -42.7654516
H 10.7042933 3.2772 -42.7433744
C 12.7172875 2.6995277 -42.243958
H 13.7411237 3.021697 -42.4103113
H 12.5627012 1.7344082 -42.7242798
H 12.5786212 2.5252549 -41.1832717
C 11.6078686 4.8907969 -41.7666777
H 12.5088229 4.963613 -41.1569353
H 10.7748965 4.7152793 -41.0967139
H 11.4403098 5.837781 -42.2673589
N 13.7997814 5.3551081 -43.5357305
H 13.5427582 4.9757027 -42.6392564
C 14.9722896 6.1999847 -43.5773004
H 14.7513394 7.1316738 -44.1038787
C 16.1325397 5.5277022 -44.3124657
O 16.971215 6.2121722 -44.8791594
C 15.3363167 6.5059358 -42.1167684
H 14.5993989 7.1982355 -41.7073855
H 15.2826306 5.5845827 -41.5328418
C 16.7149966 7.0988042 -41.9408267
C 16.9780411 8.3984896 -42.403626
H 16.1908624 8.9809401 -42.8654804
C 17.7536204 6.3218351 -41.3924162
H 17.5600729 5.3123153 -41.0611771
C 18.2759221 8.9240865 -42.299878
H 18.4802673 9.9218835 -42.6403416
C 19.0546992 6.8452967 -41.3078177
H 19.8555147 6.2433117 -40.90308
C 19.314826 8.149128 -41.7601691
H 20.3159865 8.5514257 -41.6963891
N 16.2058513 4.196207 -44.3268838
H 15.3924863 3.6449569 -44.0809636
C 17.3365519 3.5317345 -44.9880782
H 18.247824 4.0148928 -44.6328091
C 17.3362498 3.7162578 -46.5202865
O 18.411362 3.6889561 -47.1212283
C 17.4139431 2.0551039 -44.544089
H 17.2640812 2.0186564 -43.4640312
C 18.7685605 1.4097662 -44.8475106
H 18.7772956 0.3945679 -44.4540022
H 19.5699411 1.9849547 -44.3903993

H 18.9200973 1.3641078 -45.9264033
O 16.4573901 1.2286298 -45.1599023
H 15.5577683 1.5969757 -45.0914068
N 16.1818698 4.0690221 -47.1078517
H 15.3465728 4.1192239 -46.5422669
C 16.0387294 4.3659216 -48.539581
H 16.9320402 3.9392428 -48.9945931
C 16.0907108 5.8966401 -49.0803814
O 16.0794458 5.9767522 -50.2908172
C 14.8556147 3.4957892 -49.0773819
H 13.9798023 4.1366509 -49.1933825
H 14.5968292 2.7346661 -48.3378472
C 15.1275989 2.7525254 -50.4024108
O 14.1400315 2.2286328 -50.965854
O 16.307982 2.6299838 -50.8096799
N 16.2900343 7.1238414 -48.4203981
H 16.4497442 7.0131143 -47.4277553
C 16.1613041 8.688626 -48.8951202
H 15.5643498 8.6270493 -49.8051702
C 17.4543756 9.8556596 -49.3389581
O 18.4787587 9.2741032 -49.0634247
C 15.1839224 9.2297703 -47.7945255
H 14.6070166 8.4267525 -47.3302822
H 14.4664933 9.8869038 -48.2885934
C 15.9020718 10.0680588 -46.713811
O 16.3872989 11.1886415 -47.009515
O 15.9809798 9.6471257 -45.537687
N 17.6582507 11.2710311 -50.0187799
H 16.7432841 11.6473397 -50.215925
C 18.9196021 12.4999217 -50.4996121
H 19.4239745 12.7072473 -49.557024
C 19.8910586 11.6284874 -51.3214552
H 19.3040143 10.926469 -51.916006
H 20.4598406 12.2416738 -52.0211191
C 20.8996192 10.8577388 -50.4511839
H 20.4908512 10.6758339 -49.4595413
H 21.799341 11.4600084 -50.3269145
C 21.2793501 9.5187183 -51.0747255
O 22.2065281 9.4215954 -51.8603748
N 20.5592735 8.459898 -50.7803827
H 20.794535 7.5910496 -51.2218482
H 19.7724068 8.5434667 -50.1355936
C 19.092366 14.182846 -51.3913657
O 18.0971829 14.350783 -52.0856275
N 20.1085432 15.3856543 -51.5493382
H 20.9260687 15.212857 -50.986138

C 20.2948739 16.9461427 -52.3131094
H 20.2227503 16.7196393 -53.3770916
C 21.4078328 18.3179152 -52.2406162
O 22.5468109 18.183726 -51.7988277
C 19.0686253 17.7464106 -51.9697146
H 19.2338989 18.2728335 -51.0391949
H 18.2005998 17.0932304 -51.9204808
H 18.8667864 18.4802587 -52.7438545
N 21.1702238 19.6749261 -52.6514902
H 20.2345851 19.8379585 -52.9935248
C 22.0024176 21.0115075 -52.4479166
H 22.8683875 20.7431257 -51.8441611
C 21.2832516 22.1579803 -51.6588977
O 20.098186 22.3880363 -51.8856444
C 22.5113401 21.7661131 -53.7349204
H 21.6390627 22.0773934 -54.3111731
H 23.0283835 22.6774737 -53.4283394
C 23.4507222 21.0203275 -54.6889031
H 22.9115143 20.1707727 -55.1106251
H 23.7236569 21.6915994 -55.5045992
C 24.7266258 20.5101083 -54.0066815
H 24.4530278 19.8220364 -53.2042071
H 25.2791937 21.3544152 -53.5879837
C 25.5984771 19.7681775 -55.0268131
H 25.909443 20.4728439 -55.8051529
H 24.9888066 18.9920389 -55.5006992
N 26.7854235 19.1515691 -54.3842775
H 27.3653918 19.8580064 -53.9512649
H 27.3408378 18.6543916 -55.0695705
H 26.4940335 18.4923601 -53.6723818
N 22.0141776 22.9790716 -50.8776924
H 22.9848334 22.7561847 -50.7481297
C 21.4363151 24.0595014 -50.0406279
H 20.6651534 23.5880877 -49.4217957
C 20.6932301 25.1571828 -50.8434591
O 21.1123859 25.4928957 -51.9501055
C 22.4954675 24.6021902 -49.0576117
H 21.9681595 25.0697176 -48.2281703
H 23.0446883 23.7611733 -48.6298199
C 23.4967813 25.6136439 -49.6395768
H 24.1308012 25.1185841 -50.3761876
H 22.9616084 26.426131 -50.1294037
C 24.3600993 26.2058559 -48.5122502
H 25.0695733 25.4548231 -48.159759
H 23.7129745 26.4715583 -47.6719088
C 25.1007745 27.47991 -48.9414417

H 25.6487684 27.8643149 -48.0751497
H 24.3538345 28.231996 -49.2207631
N 26.033051 27.2429212 -50.0717632
H 26.729265 26.5557402 -49.8134378
H 26.5058074 28.1019389 -50.3270792
H 25.5216444 26.9054137 -50.8778729
N 19.6653497 25.7902966 -50.2502701
C 18.5260935 26.4199977 -50.9302971
H 17.683172 26.2611953 -50.25573
C 17.9666354 25.9230463 -52.2825018
O 17.2120042 26.6858309 -52.8855268
C 18.8245867 27.9163968 -50.8994079
H 19.6064095 28.1600803 -51.6210341
H 17.9311673 28.5198536 -51.0658878
C 19.9982801 26.7186724 -49.1631419
H 19.6497361 26.3469128 -48.204326
H 21.0706298 26.8855675 -49.103357
C 19.3411503 28.0678737 -49.4726293
H 18.5032263 28.2293829 -48.7937525
H 20.0551531 28.8885143 -49.395854
N 18.1444302 24.6585658 -52.7170806
H 18.7575415 24.0313991 -52.2081714
C 17.2497154 24.0944877 -53.7603078
H 17.1809009 24.8305569 -54.5644565
C 15.8108259 23.9536811 -53.2184155
O 15.6116559 23.7733122 -52.0168994
C 17.7905208 22.7718561 -54.367871
H 18.2164828 22.1394345 -53.5915213
H 16.9504542 22.2274091 -54.799801
C 18.8275157 22.9966047 -55.490398
H 19.7490875 23.3914824 -55.0618205
H 18.4292468 23.7618966 -56.1540239
C 19.1471149 21.7322105 -56.331149
H 18.3107319 21.0347568 -56.2604309
H 20.0375569 21.2460875 -55.9308658
N 19.3493069 22.0762304 -57.7577649
H 19.2235844 23.0639289 -58.0004847
C 19.3902273 21.3013719 -58.8329725
N 19.5688706 20.0105413 -58.789513
H 19.5868354 19.4947518 -59.6476506
H 19.6328429 19.5190873 -57.9186205
N 19.2411043 21.8219077 -60.01574
H 18.7989704 22.7503794 -60.07748
H 19.2658129 21.2691169 -60.8598066
N 14.821947 23.9635607 -54.1171727
H 15.0640462 24.1295175 -55.0795782

C 13.4070457 23.7053603 -53.8023649
H 13.2318405 23.8314889 -52.7303655
C 13.0148305 22.2690653 -54.1901151
O 13.2650034 21.8554559 -55.3268438
C 12.5042714 24.70102 -54.554355
H 11.4657565 24.4018961 -54.4176821
H 12.7363243 24.6603459 -55.6166903
C 12.5918951 26.14774 -54.1111584
O 13.1869008 26.5106661 -53.1113018
N 11.9857542 27.0281791 -54.8750885
H 12.0663734 27.990201 -54.6088372
H 11.474667 26.7304553 -55.7012412
N 12.3104354 21.5618859 -53.3039725
H 12.1319232 21.9882772 -52.3959269
C 11.7798987 20.2088839 -53.5218785
H 12.1354009 19.8236533 -54.4750295
C 10.2499869 20.2006078 -53.5304971
O 9.636071 20.6928764 -52.5837385
C 12.2939867 19.2814334 -52.4104324
H 12.0003572 19.7111831 -51.4550013
H 13.3811827 19.2399207 -52.459261
C 11.7331357 17.8531895 -52.451972
H 10.6473428 17.8812916 -52.5280063
H 11.9562163 17.3702826 -51.5054196
S 12.3827862 16.813984 -53.7779941
C 13.8931229 16.2105975 -52.9728887
H 14.4627188 15.602152 -53.6752431
H 13.6285852 15.6018071 -52.1075691
H 14.5036321 17.0516099 -52.6454799
N 9.6561252 19.476022 -54.4810838
H 10.2356146 19.1014098 -55.228018
C 8.2618632 19.0294284 -54.4110718
H 7.7432147 19.5950109 -53.6411374
C 8.2012683 17.5367186 -54.0391612
O 8.6091906 16.6916482 -54.8315145
C 7.5848191 19.3377223 -55.7563497
H 7.6533027 20.404727 -55.9472126
H 8.1407942 18.8235216 -56.5354205
C 6.1094319 18.9161937 -55.8471269
H 6.0223168 17.8621598 -55.5987058
C 5.2008171 19.7105303 -54.9111302
H 5.2842959 20.7773402 -55.1160026
H 5.4789999 19.5191252 -53.8783082
H 4.1706631 19.3882301 -55.0433692
C 5.5958432 19.1157678 -57.2735893
H 5.6369121 20.1715894 -57.5396105

H 4.5696247 18.7588489 -57.3505647
H 6.2159663 18.5490014 -57.9673902
N 7.650806 17.207511 -52.8705797
H 7.2809473 17.9542867 -52.2913677
C 7.3710433 15.8279812 -52.4464654
H 8.0060151 15.1508264 -53.0126345
C 5.9027388 15.4793458 -52.7611271
O 4.9709185 16.075372 -52.2065813
C 7.75986 15.6405981 -50.9667627
H 7.1203425 16.2573012 -50.3370099
H 8.7862314 15.9905422 -50.8370111
C 7.6824443 14.1863395 -50.4760692
O 6.8708591 13.4090086 -51.019936
O 8.4087115 13.8653462 -49.5055914
N 5.6864177 14.5711102 -53.7189983
H 6.4974395 14.0713565 -54.0777662
C 4.3447916 14.1842873 -54.1843736
H 3.6345714 14.9349283 -53.8406842
C 3.9351687 12.8544228 -53.5605881
O 4.3232699 11.7831128 -54.0192536
C 4.2336867 14.1575708 -55.7201635
H 4.8568936 13.3737071 -56.1278179
C 2.7974381 13.8629456 -56.1479142
H 2.1426664 14.6095088 -55.703318
H 2.4969621 12.8683791 -55.8186253
H 2.7229068 13.8922377 -57.2343704
C 4.6461988 15.4961607 -56.3443168
H 4.0765308 16.3124174 -55.897042
H 4.4772019 15.482627 -57.4198195
H 5.7094427 15.6583488 -56.1699027
N 3.0257182 12.9205146 -52.5909151
H 2.717312 13.8404901 -52.2952782
C 2.6511322 11.7941466 -51.7378099
H 1.5919382 11.8736081 -51.495824
H 2.826334 10.8515972 -52.2505211
C 3.4378837 11.764253 -50.4292475
O 3.7783718 10.6859284 -49.9559292
N 3.6725557 12.9262538 -49.8125931
H 3.3218807 13.7634739 -50.2589932
C 4.5639542 13.1282033 -48.6574106
H 5.5763021 12.8435635 -48.9560907
C 4.2394702 12.317265 -47.379758
O 4.9944892 12.3378076 -46.4012804
C 4.557135 14.6320349 -48.3562011
H 4.9178299 15.1702423 -49.2356233
H 5.2327209 14.8285762 -47.5267004

S 2.8894722 15.2074225 -47.9074698
H 2.7061254 14.4032963 -46.8479299
N 3.0995178 11.6227276 -47.3420058
H 2.5537956 11.6005599 -48.1895828
C 2.6973692 10.7695608 -46.2284903
H 1.7296098 10.3306215 -46.4382888
H 3.4252333 9.964224 -46.1301078
C 2.5964225 11.5335775 -44.9096616
O 2.0756425 12.6458024 -44.8617237
N 3.1475683 10.9405853 -43.8409104
H 3.5995258 10.050913 -43.9813798
C 3.2644505 11.5676352 -42.5110625
H 2.5363332 12.3721322 -42.4922009
C 4.6024855 12.3215117 -42.2989558
O 4.9330438 12.7035656 -41.1837733
C 2.7685976 10.6219858 -41.3876643
H 3.329892 9.6866489 -41.3966698
H 2.9110664 11.1133101 -40.4234859
C 1.2482459 10.3497499 -41.5881163
H 0.7919812 11.3134804 -41.7977461
H 1.1127473 9.7337692 -42.4784474
C 0.4227669 9.7161131 -40.4391496
H 0.7314226 10.1512193 -39.4901884
H 0.5960085 8.639396 -40.4025918
C -1.0758004 10.0217816 -40.7095582
H -1.41602 9.4220592 -41.5561837
H -1.1388727 11.0716698 -41.0127608
N -2.0107233 9.8625097 -39.5598994
H -2.0996365 8.9127489 -39.2334567
H -2.9406933 10.1949484 -39.8135329
H -1.7309402 10.4529809 -38.7839952
N 5.3030928 12.6456133 -43.3932679
H 4.9509657 12.3158047 -44.2839729
C 6.263606 13.7539353 -43.4685514
H 6.339384 14.0373157 -44.5168193
H 5.8664254 14.6025745 -42.9108326
C 7.6966603 13.5217271 -42.9844836
O 8.5229441 14.4189115 -43.1446022
N 8.052298 12.3272621 -42.5032093
H 7.3432049 11.6119669 -42.3818921
C 9.4128024 12.0200965 -42.040962
H 9.6646061 12.6654394 -41.1981862
H 9.4474178 10.9834761 -41.7123813
C 10.481707 12.1834795 -43.1192423
O 11.5222019 12.7773601 -42.8556136
N 10.1750316 11.7843984 -44.3589289

H 9.2846575 11.3332788 -44.4955374
C 11.0186339 12.0413754 -45.5425943
H 11.9980506 11.588156 -45.3790969
C 11.2701527 13.5389522 -45.7486745
O 12.4236188 13.9518113 -45.8260238
C 10.4258214 11.3832015 -46.8083101
H 10.5905208 10.3099959 -46.7269139
C 8.9312833 11.6033541 -47.0463739
H 8.7099762 12.6632397 -47.1442538
H 8.3393666 11.1827496 -46.2344072
H 8.6361123 11.1131843 -47.9758533
O 11.0895781 11.8306891 -47.9597574
H 10.484578 12.3844576 -48.4701324
N 10.2252136 14.371186 -45.663101
H 9.3074535 13.9722939 -45.5446904
C 10.2857366 15.8236971 -45.8547703
H 10.7235008 16.0265557 -46.8268567
C 11.1106349 16.537419 -44.7650356
O 11.8863183 17.4526382 -45.0535196
C 8.8657689 16.3970441 -45.8856242
H 8.8986116 17.4030429 -46.3069571
H 8.4867539 16.4648006 -44.8668808
S 7.7163401 15.3759861 -46.8510702
H 8.4533831 15.1086649 -47.9512639
N 10.9632892 16.0999518 -43.508704
H 10.2825801 15.3646959 -43.3383485
C 11.7666912 16.5933009 -42.3777083
H 11.7210304 17.682167 -42.3558897
C 13.2337723 16.2149077 -42.5646649
O 14.1041332 17.0794444 -42.4631432
C 11.2181531 16.0596538 -41.0385742
H 11.0788588 14.9803609 -41.1088303
C 12.1583111 16.3454275 -39.8608403
H 12.413162 17.4044263 -39.8365978
H 13.0713173 15.7583224 -39.9544092
H 11.6747709 16.0658247 -38.9240883
C 9.8619149 16.7101419 -40.7311641
H 9.9738266 17.7906836 -40.6408162
H 9.465485 16.313601 -39.7953368
H 9.147258 16.4904463 -41.5236473
N 13.5086787 14.9554922 -42.9189931
H 12.7374487 14.300883 -43.0121126
C 14.8616324 14.4600437 -43.1763215
H 15.4605874 14.5795315 -42.2714439
C 15.5717593 15.257738 -44.2626702
O 16.6984701 15.6980688 -44.0520232

C 14.8119003 12.9696725 -43.5454865
H 15.47017 12.778969 -44.3885111
H 13.8159203 12.6800994 -43.8734669
C 15.2350372 12.1081566 -42.3887418
N 16.5503927 11.9810507 -41.9354251
C 16.4753185 11.2196844 -40.8299497
H 17.3253826 10.9192111 -40.2306378
N 15.2029599 10.8627996 -40.5836198
H 14.890693 10.2906702 -39.8085624
C 14.4037633 11.4233475 -41.5538219
H 13.3257702 11.3738953 -41.6274226
N 14.9078435 15.5075468 -45.387301
H 13.989292 15.091668 -45.5152898
C 15.5112271 16.2350314 -46.5011762
H 16.4944499 15.8005517 -46.6810838
C 15.788866 17.702243 -46.1455768
O 16.9395239 18.1374501 -46.2012692
C 14.6897345 16.0179734 -47.7882799
H 15.1327128 16.6236197 -48.5603665
C 14.7908711 14.5234165 -48.182398
H 14.1962195 13.8987777 -47.5198436
H 15.8220997 14.1781548 -48.1262874
H 14.4372692 14.3716629 -49.2011021
C 13.2366266 16.4975189 -47.7320752
H 12.7646062 16.0195718 -46.8976035
H 13.2059957 17.5711979 -47.5585925
C 12.4407379 16.1837724 -49.0033466
H 12.2244151 15.1164961 -49.062049
H 13.0189562 16.48687 -49.8741816
H 11.4970485 16.7230964 -48.9888032
N 14.8187234 18.4245126 -45.5793613
H 13.9008259 18.0120409 -45.4473034
C 15.0314858 19.8172963 -45.1857533
H 15.4248899 20.3646793 -46.0422485
C 16.0654803 19.9815299 -44.052507
O 16.775709 20.9872676 -44.0197683
C 13.6739593 20.4215669 -44.8288207
H 13.2487701 19.8884107 -43.9775321
H 12.9997992 20.3346726 -45.682005
H 13.7950387 21.4777533 -44.588073
N 16.1945128 19.0031707 -43.1417661
H 15.5550477 18.2125874 -43.1960906
C 17.1426889 19.0652184 -42.0165262
H 17.2682905 20.115006 -41.7540704
C 18.5555244 18.5806105 -42.3633946
O 19.5022617 19.2022987 -41.8920977

C 16.5356863 18.3408313 -40.8014493
H 15.5546198 18.7709715 -40.5929376
H 16.3868088 17.2899072 -41.0560764
C 17.3783728 18.4041283 -39.5137768
H 18.2761985 17.8007418 -39.655826
H 16.803368 17.9420041 -38.7110996
C 17.8159365 19.8120997 -39.0658109
H 18.4907804 20.2290409 -39.816017
H 18.3932875 19.7217441 -38.1440225
N 16.6897875 20.7491832 -38.8609668
H 16.5665419 21.4502419 -39.5716769
C 15.8807065 20.8193544 -37.8169131
N 14.9853566 21.7589121 -37.7435133
H 14.3645694 21.8061651 -36.956886
H 14.8726153 22.4170928 -38.4946404
N 15.9472023 19.9843426 -36.8189475
H 16.6241288 19.2455779 -36.8502505
H 15.2949912 20.0477172 -36.0589826
N 18.7300829 17.5290883 -43.1764286
H 17.9068943 17.0488226 -43.5306661
C 20.0684893 17.0390531 -43.5746573
H 20.7065707 16.9291063 -42.6964889
C 20.7865705 18.0002679 -44.5366372
O 22.0134416 18.0053269 -44.5586972
C 19.9813346 15.699411 -44.3219547
H 19.304658 15.8747004 -45.1616404
H 20.9671976 15.5209637 -44.7569981
C 19.5628823 14.3578569 -43.6784448
H 20.3290906 14.0366348 -42.971993
H 18.6129878 14.4437749 -43.1562454
C 19.4543242 13.3380914 -44.844005
H 20.418487 13.3271226 -45.354375
H 18.7167883 13.694725 -45.5669937
C 19.1362141 11.8730891 -44.4980398
H 19.490443 11.6480878 -43.4892399
H 19.686472 11.2395421 -45.2001729
N 17.6955314 11.5486037 -44.6343123
H 17.1272324 12.0169392 -43.9401613
H 17.4806865 10.5531329 -44.5600195
H 17.3202713 11.7308229 -45.5705452
N 20.0478271 18.6624719 -45.4364741
H 19.046923 18.5093921 -45.436498
C 20.6358853 19.3309775 -46.6085273
H 21.6749389 19.5607893 -46.3684505
C 20.0541746 20.7091617 -46.9622581
O 20.3152965 21.2210728 -48.0515675

C 20.6868561 18.354292 -47.7957786
H 21.0473326 18.879063 -48.6814314
H 21.4566581 17.6177888 -47.5613333
C 19.4373287 17.602644 -48.1667651
C 19.394892 16.1940631 -48.1271936
H 20.196105 15.6434021 -47.6560693
C 18.3451897 15.5113149 -48.7728262
H 18.2923324 14.4372359 -48.7484014
C 17.3896775 16.2497781 -49.5044677
O 16.5031357 15.6745726 -50.3589183
H 16.8175994 14.8393012 -50.723428
C 17.3695605 17.6457992 -49.4114359
H 16.6398896 18.1806343 -49.9827419
C 18.3848271 18.32481 -48.7295873
H 18.4231516 19.3990316 -48.7319403
N 19.3517895 21.3539171 -46.024161
H 19.1651048 20.8775033 -45.1539639
C 19.0384741 22.7911583 -46.0750765
H 18.3539854 22.9771147 -45.2457682
C 18.2660689 23.2374827 -47.3416898
O 18.3498463 24.3844502 -47.7803859
C 20.3254224 23.5929801 -45.7682459
H 21.0190659 23.4837652 -46.6030665
H 20.8007875 23.1682607 -44.8823023
C 20.0831759 25.0814136 -45.500918
O 19.0655738 25.4065578 -44.8420731
O 20.8655128 25.9146396 -46.0124582
N 17.4548563 22.3556176 -47.9295338
H 17.4238769 21.424189 -47.5465261
C 16.4823397 22.7281121 -48.9710723
H 16.9464183 23.4477338 -49.6473765
C 15.24281 23.3977265 -48.3725054
O 14.9522023 23.2457856 -47.1836795
C 16.0422905 21.5162724 -49.8130752
H 15.2603537 21.8499514 -50.4920367
C 17.2011516 21.0558416 -50.7125561
H 18.1143336 20.9147098 -50.1404907
H 17.4026366 21.8357731 -51.4439333
H 16.943333 20.1436236 -51.2459316
C 15.398919 20.4182088 -48.9405732
H 16.1665696 19.8238888 -48.4571155
H 14.7955406 20.8598812 -48.151009
C 14.4493492 19.5350595 -49.7478417
H 14.8862927 19.2531794 -50.7036183
H 13.5268763 20.0795328 -49.9194258
H 14.2201392 18.6408149 -49.1834476

N 14.4360211 24.010578 -49.2375354
H 14.7402083 24.0446147 -50.2085445
C 12.9993631 24.158699 -49.0076405
H 12.8175786 24.2573771 -47.9397338
C 12.2662528 22.9046082 -49.5103378
O 12.6107608 22.3634176 -50.5604784
C 12.5234879 25.4517901 -49.6859483
H 13.0406288 26.283309 -49.2048236
H 12.830272 25.44303 -50.731898
C 11.0112581 25.7338392 -49.6079951
H 10.8575365 26.7986213 -49.7828324
H 10.658697 25.5126149 -48.6002629
C 10.1424015 24.9839405 -50.6247752
O 10.5934761 24.3664138 -51.5757024
N 8.8434225 24.9954737 -50.4307784
H 8.2650384 24.4828781 -51.0906918
H 8.4331033 25.5005896 -49.6701504
N 11.2359635 22.4621827 -48.7844512
H 11.0578115 22.9049529 -47.8891113
C 10.3429926 21.383246 -49.214284
H 10.5949631 21.0920705 -50.235015
C 8.8711568 21.8264889 -49.2272665
O 8.3637704 22.3460571 -48.2286926
C 10.5732244 20.1618264 -48.309774
H 10.3332883 20.4190762 -47.2777976
H 11.6227969 19.8672222 -48.3609254
S 9.5400098 18.7650291 -48.8429061
H 8.3402798 19.3259352 -48.6493615
N 8.1565084 21.4877271 -50.299685
H 8.6580875 21.1050509 -51.0997332
C 6.6915622 21.4723115 -50.3796701
H 6.2622286 21.8728319 -49.4665579
C 6.2201094 20.031677 -50.5303368
O 6.4399128 19.4158451 -51.5664035
C 6.1943466 22.3420754 -51.5434893
H 6.7074003 22.060723 -52.4640156
C 4.6826819 22.2560971 -51.7615391
H 4.1511206 22.4652032 -50.8329504
H 4.408085 21.2625488 -52.117419
H 4.3832472 22.9787312 -52.5207642
O 6.4597851 23.693421 -51.2481149
H 6.0035097 23.908674 -50.423727
N 5.5750189 19.4858299 -49.5003679
H 5.4489048 20.0421054 -48.6609263
C 4.8934822 18.1915638 -49.5707935
H 5.3805521 17.5583986 -50.3075649

H 4.9561912 17.6920084 -48.6044006
C 3.4178491 18.3381219 -49.9451647
O 2.7499778 19.2457229 -49.4508098
N 2.8759735 17.4192718 -50.7447628
H 3.489359 16.728848 -51.172119
C 1.4218424 17.2768095 -50.9304672
H 0.9352328 17.8212694 -50.1261785
C 0.9560668 15.8305307 -50.7697592
O 1.6406314 14.8966804 -51.1867693
C 0.9136146 17.8905378 -52.2565359
H -0.1448096 17.6360649 -52.3435319
C 1.6387051 17.3248981 -53.5004349
H 2.6843131 17.6363338 -53.4916327
H 1.6106378 16.2363657 -53.4623999
C 0.9961788 19.4249524 -52.1858548
H 2.0365676 19.7521869 -52.1926341
H 0.5247676 19.7783184 -51.266841
H 0.4642133 19.8755679 -53.0218273
C 1.0052803 17.7519525 -54.8316522
H 1.1088672 18.8260369 -54.9781292
H -0.0515642 17.481976 -54.8436029
H 1.510511 17.2397663 -55.65089
N -0.2477491 15.6495421 -50.2260329
H -0.7465903 16.4668413 -49.8863459
C -0.9656272 14.3704334 -50.212057
H -0.6470092 13.784003 -51.0743246
C -2.4736961 14.6046495 -50.3361702
O -2.9923013 15.6108318 -49.855645
C -0.6345655 13.5626088 -48.9469876
H -1.035592 14.063649 -48.0667914
H 0.4483293 13.4718768 -48.8486547
O -1.2039403 12.2647033 -49.0661658
H -0.8230701 11.6633302 -48.3915009
N -3.189996 13.6624306 -50.9545782
H -2.693636 12.8593069 -51.3085801
C -4.6570956 13.7038033 -51.1109514
H -4.9453094 14.6681594 -51.5324737
C -5.3659256 13.5807981 -49.7505522
O -6.4750307 14.0756237 -49.5707605
C -5.0838402 12.5810381 -52.0946599
H -4.5868338 11.656628 -51.7934535
C -6.60297 12.3174183 -52.0927149
H -7.1427278 13.2367823 -52.3255887
H -6.9278242 11.951214 -51.1188279
H -6.8636599 11.5560362 -52.8261546
C -4.6265523 12.9470809 -53.5247128

H -5.2699191 13.744181 -53.8956398
H -3.6030739 13.3215718 -53.5000957
C -4.6555746 11.788384 -54.5300713
H -5.6777838 11.458972 -54.7113472
H -4.0625365 10.9539342 -54.1543668
H -4.2321241 12.1260136 -55.4769672
N -4.7271601 12.913471 -48.788543
H -3.775143 12.6272275 -48.9723444
C -5.2999645 12.5558781 -47.4919011
H -6.3581933 12.3426238 -47.6238231
C -5.12327 13.6827605 -46.4548777
O -3.9915082 13.9538372 -46.0403971
C -4.6452439 11.2446981 -47.0565748
H -3.587913 11.2809028 -47.3174804
H -5.1004324 10.4159779 -47.6001787
O -4.7366918 11.0128571 -45.6723862
H -5.6758702 11.0025864 -45.3613888
N -6.2181431 14.2734505 -45.9265974
C -6.1448261 15.2535177 -44.8392723
H -5.5037262 16.0768951 -45.1469994
C -5.5994107 14.6593758 -43.5365247
O -4.990551 15.3639187 -42.7414758
C -7.5767455 15.7683423 -44.6357585
H -8.056633 15.2366621 -43.8112928
H -7.5919532 16.8425494 -44.4489233
C -7.6005661 14.1249832 -46.3585443
H -7.6892693 14.0050037 -47.4364082
H -8.051605 13.2737373 -45.845865
C -8.2932888 15.4153499 -45.9362787
H -8.111043 16.1900229 -46.680453
H -9.3633916 15.2698172 -45.7853592
N -5.798928 13.3603836 -43.3244477
H -6.3334307 12.8472974 -44.0199305
C -5.3819279 12.5909009 -42.1533687
H -5.5086175 13.2194111 -41.2710266
C -3.889537 12.1953296 -42.1819728
O -3.2267231 12.2061379 -41.1417984
C -6.3412377 11.3905832 -41.9922851
H -7.3113502 11.7703061 -41.6675729
H -5.9575394 10.7498656 -41.1971473
C -6.5663179 10.5388814 -43.2538189
O -6.908747 11.078591 -44.3329274
O -6.3508923 9.3107568 -43.1812859
N -3.3008267 11.9683216 -43.3619274
H -3.8866016 11.9470974 -44.1933319
C -1.8430178 11.9831623 -43.5618261

H -1.3723892 11.257173 -42.9150736
C -1.2618897 13.3502526 -43.1982805
O -0.4067809 13.4546671 -42.3135894
C -1.4776396 11.6187214 -45.0103698
H -2.1218696 12.1563712 -45.7031098
H -0.4492902 11.9202012 -45.2048059
C -1.5826308 10.1109484 -45.2530965
H -2.542401 9.7508069 -44.8866851
H -0.8029225 9.6052696 -44.6769365
C -1.4265517 9.7523301 -46.7342513
O -2.1887154 8.9014817 -47.2378949
O -0.4387212 10.1870761 -47.3704002
N -1.8238071 14.3981459 -43.7998905
H -2.5652821 14.2183388 -44.4716944
C -1.3629664 15.7743681 -43.644951
H -0.2966211 15.7971953 -43.8619811
C -1.5011144 16.2671664 -42.1931932
O -0.5669668 16.8774303 -41.6842395
C -2.0937697 16.6432986 -44.6956286
H -3.1616424 16.4331521 -44.6208048
C -1.6453572 16.3062192 -46.1415492
H -1.8060816 15.2459139 -46.3228022
H -2.2857106 16.8442621 -46.8391984
C -1.9169274 18.1412204 -44.4519846
H -0.8624135 18.3648704 -44.4776171
H -2.351247 18.4235725 -43.4927015
H -2.4173335 18.7036014 -45.2397571
C -0.1850993 16.6247081 -46.5047295
H 0.502714 16.0852862 -45.8550906
H 0.005957 17.6948585 -46.4390428
H -0.0012286 16.3148047 -47.5317343
N -2.5671139 15.9256739 -41.4689525
H -3.3267024 15.4447242 -41.9411177
C -2.7735512 16.3191514 -40.0675664
H -2.7786111 17.4067291 -40.0129945
C -1.6436206 15.8404344 -39.1348382
O -1.1884872 16.6049745 -38.2795479
C -4.1712491 15.8244989 -39.6523631
H -4.8964345 16.3669418 -40.2600162
H -4.276831 14.7663523 -39.8970248
C -4.5547229 16.0502673 -38.1793378
H -5.6347657 16.1922723 -38.1337433
H -4.0852274 16.9595111 -37.8033673
C -4.2106249 14.87867 -37.2602295
O -3.478791 13.9624486 -37.5900405
N -4.7524033 14.838826 -36.0651956

H -4.5608134 14.0108061 -35.5281391
H -5.3835633 15.5549238 -35.7602172
N -1.1004638 14.6424705 -39.3708469
H -1.5013084 14.0764096 -40.106404
C 0.0418472 14.1322373 -38.6116889
H -0.0436926 14.4501244 -37.5713118
C 1.3850099 14.6518156 -39.1239408
O 2.2660308 14.8908551 -38.3035319
C 0.0435491 12.6104591 -38.6527647
H 1.0465421 12.2488474 -38.4138007
H -0.2262749 12.3017169 -39.6598596
S -1.1117245 11.9445062 -37.4241918
H -2.0986728 12.845829 -37.6105998
N 1.5575739 14.8170369 -40.4417058
H 0.8054631 14.5690666 -41.0762493
C 2.7701594 15.422793 -40.987119
H 3.6444812 14.9045317 -40.5883586
C 2.8696392 16.8863013 -40.5636503
O 3.5847836 17.2487937 -39.6283336
C 2.7907183 15.3258836 -42.5238653
H 1.9156234 15.7985274 -42.9702768
H 2.7822393 14.2994419 -42.8465516
H 3.7036186 15.7788831 -42.9060338
N 2.1002966 17.7180647 -41.2791163
H 1.1970245 17.3057366 -41.4945638
C 2.6117307 18.5517644 -42.3861192
H 3.1341734 17.8723516 -43.0516016
C 1.3970127 19.0600589 -43.2242848
H 1.358215 18.4262558 -44.0974844
H 0.5018309 18.6692998 -42.7733033
C 1.1166488 20.5727843 -43.5444475
H 1.2915895 21.151315 -42.6635274
H 1.8411256 20.9728381 -44.2381746
C -0.3230066 20.9360951 -43.9914732
H -1.0227159 20.3720331 -43.3859128
H -0.4699022 20.6178342 -45.0179328
C -0.6963571 22.4298742 -43.8025919
H 0.1995555 22.9755746 -43.4898631
H -1.4260614 22.4979107 -42.9928553
N -1.2635441 23.0865246 -45.012343
H -0.5895041 23.0906335 -45.7804691
H -1.4992876 24.0597524 -44.8902245
H -2.0834365 22.6400856 -45.4285437
C 3.7642777 19.4606364 -41.9383207
O 4.6934606 18.9814363 -41.3095185
N 3.6311882 20.7771193 -41.9989438

H 2.8876535 21.1417414 -42.5604266
C 4.0788258 21.6000937 -40.8865894
H 5.1237588 21.3834285 -40.6673247
C 3.9999911 23.0820359 -41.3327063
H 3.0493725 23.2473995 -41.8409777
H 4.7944901 23.2717171 -42.0553756
C 4.1026739 24.1179475 -40.2399803
N 3.0918861 25.0331692 -39.9409579
C 3.4223691 25.5589047 -38.7527186
H 2.8010558 26.2448663 -38.192991
N 4.5752577 25.0358408 -38.3042514
H 4.8930887 25.1113105 -37.3429746
C 5.0364284 24.1390869 -39.2428601
H 5.8685618 23.4582498 -39.1352582
C 3.2420288 21.199852 -39.6385361
O 3.0797663 22.025223 -38.7500341
N 2.530232 20.0383135 -39.640792
H 2.865367 19.2416506 -40.1623869
C 1.4823966 19.8353562 -38.7041374
H 1.2481924 20.7922532 -38.2384218
C 2.0858318 19.0933861 -37.5361898
O 3.067777 19.6165891 -37.0050026
C 0.1413169 19.4928001 -39.3442083
H 0.1442248 18.4588372 -39.6817178
H -0.6141341 19.569777 -38.5611991
C -0.267998 20.4123699 -40.5175301
H 0.1183068 19.9509151 -41.3678312
C -1.7776922 20.4353344 -40.70414
H -2.2492346 20.8376265 -39.8066394
H -2.1313518 19.4171078 -40.8620015
H -2.0806715 21.041301 -41.5528518
C 0.1944014 21.8747087 -40.5369619
H 1.2344464 22.003532 -40.7787416
H 0.035205 22.3050513 -39.5470494
H -0.3843236 22.4742456 -41.2283432
N 1.4878269 18.020571 -37.0275607
H 0.680745 17.619781 -37.490007
C 1.8370793 17.567107 -35.682473
H 1.6480608 18.3923588 -34.994206
C 3.3255548 17.1909504 -35.5105984
O 3.975249 17.6990186 -34.5966676
C 0.897452 16.4265003 -35.2938252
H 1.0844826 15.5580817 -35.9233497
H -0.1416476 16.7397962 -35.4098183
H 1.0723946 16.150676 -34.2533891
N 3.895938 16.3935091 -36.4204664

H 3.3273487 16.0340792 -37.1822979
C 5.2891756 15.9425756 -36.3358269
H 5.4806631 15.6036171 -35.3165186
C 6.3276383 17.0397494 -36.6405866
O 7.4910534 16.9063308 -36.2670223
C 5.4618095 14.744717 -37.274927
H 5.2733635 15.0489023 -38.3063018
H 4.7617187 13.9547081 -37.0015401
H 6.47971 14.3605827 -37.1961688
N 5.9285687 18.1328717 -37.2982429
H 4.962632 18.1795496 -37.6016175
C 6.8314934 19.2418052 -37.635999
H 7.8429605 18.8505207 -37.6832907
C 6.844892 20.3829303 -36.6121256
O 7.5699517 21.3645417 -36.8076774
C 6.4883151 19.7654549 -39.0249614
H 5.4763035 20.1549587 -38.9918722
H 6.5452826 18.940506 -39.7349417
O 7.3909096 20.7802827 -39.4271918
H 7.5897174 21.3108912 -38.6363837
N 6.0301942 20.3258281 -35.5583977
H 5.4633655 19.4990513 -35.4131305
C 5.9102691 21.4436743 -34.6242259
H 5.8569889 22.3420848 -35.2375565
C 7.1639527 21.593333 -33.7492034
O 7.6308825 20.6298769 -33.149975
C 4.6035516 21.3149437 -33.8160339
H 3.9610644 20.5586151 -34.2705869
H 4.8261419 20.983383 -32.8007128
C 3.8146552 22.6339158 -33.7630863
H 4.3761121 23.361526 -33.1761685
H 2.8597389 22.454182 -33.2696363
C 3.5503843 23.2164719 -35.1512038
O 4.0029425 24.3035214 -35.4804174
N 2.9488639 22.4629638 -36.0411139
H 2.950617 22.7735293 -37.0102779
H 2.7918841 21.4817724 -35.8719266
N 7.7210904 22.8081187 -33.6914249
H 7.2987774 23.5567177 -34.2190106
C 8.9972279 23.0788989 -33.0131334
H 9.0701395 24.1482844 -32.8145984
H 9.0214978 22.5526099 -32.0586359
C 10.2493908 22.6746477 -33.805537
O 11.3286693 22.5814544 -33.2268775
N 10.1271567 22.3925806 -35.1093067
H 9.2142375 22.4390954 -35.5407744

C 11.2828804 22.1739636 -35.9765919
H 12.1470555 21.9182832 -35.3639204
C 11.6311631 23.4425166 -36.7703543
O 10.8468171 23.8739616 -37.6062429
C 11.0248787 21.0051895 -36.9419587
H 10.2282802 21.2982624 -37.627223
H 11.9359448 20.8776523 -37.5163873
C 10.6521745 19.6372191 -36.3484215
H 9.6886744 19.7031776 -35.8480046
C 10.554009 18.6256642 -37.4922608
H 11.5421073 18.4392663 -37.908598
H 9.9033941 19.0025286 -38.2786216
H 10.1402736 17.6890333 -37.1141196
C 11.700169 19.1190278 -35.363519
H 12.6761902 19.0615675 -35.8409176
H 11.4049478 18.1266817 -35.0192001
H 11.7434391 19.7788958 -34.4960605
N 12.8948299 23.8661966 -36.7091582
H 13.3352564 23.7065076 -35.812184
C 13.4155394 25.0179326 -37.4790076
H 12.6681205 25.8145446 -37.4282954
C 13.6732908 24.7387133 -38.9836209
O 14.3679382 25.4938641 -39.6582383
C 14.7076514 25.5493859 -36.8171534
H 15.5330047 24.8870948 -37.0856383
H 14.9254605 26.5347873 -37.2302733
C 14.686988 25.6625318 -35.2845744
H 15.5866248 26.1902592 -34.9596257
H 14.7320807 24.6561241 -34.8586402
C 13.4378158 26.3886491 -34.7758366
O 13.4280188 27.6368107 -34.819975
O 12.4900572 25.6633847 -34.4019596
N 13.2648524 23.5772005 -39.5105643
H 12.5354699 23.085907 -39.0068225
C 13.5570177 23.1795007 -40.8976494
H 14.5254876 23.6009869 -41.1761233
C 12.542468 23.7627457 -41.8860466
O 11.3445727 23.7642826 -41.6133993
C 13.6717175 21.6505914 -41.0511484
H 14.6737933 21.3409764 -40.7633607
H 13.5409867 21.3814689 -42.0977122
C 12.6807254 20.8409029 -40.234541
O 13.0819143 19.9571612 -39.4998137
N 11.4063889 21.1652132 -40.2593715
H 10.7663929 20.6171536 -39.7086994
H 11.0835354 21.9909526 -40.7547637

N 13.0133136 24.1123106 -43.0888082
H 14.003369 23.9861169 -43.252692
C 12.2559559 24.8063065 -44.14478
H 11.634147 25.5703974 -43.6725806
C 11.2900693 23.8636724 -44.8836361
O 11.4794884 23.5296958 -46.0528063
C 13.2402964 25.5207442 -45.0935189
H 13.7974318 24.7585126 -45.6299273
H 12.6747639 26.1075278 -45.8195301
C 14.2367 26.4457212 -44.363787
H 13.6782029 27.2076148 -43.8182901
H 14.8081668 25.8651489 -43.6389009
C 15.2530414 27.1445263 -45.2802328
H 15.9662655 27.6665972 -44.6388995
H 14.7410762 27.8844194 -45.8974409
C 16.0017683 26.1590706 -46.1895801
H 15.4383225 26.0365783 -47.1185131
H 16.0485386 25.1791219 -45.7028254
N 17.3833513 26.6013149 -46.4784672
H 17.4501689 27.5257558 -46.8646949
H 17.8394313 25.9286781 -47.0934372
H 17.9672793 26.5200239 -45.6378488
N 10.2586788 23.4106932 -44.1767312
H 10.1841574 23.7178249 -43.2124954
C 9.2046284 22.5279462 -44.6902692
H 9.4646982 22.2318761 -45.7066837
C 7.8664666 23.2561846 -44.773848
O 7.5173305 24.0732073 -43.9268036
C 9.0728533 21.2239852 -43.8778855
H 8.316634 20.6091786 -44.3642886
C 10.3766276 20.4230278 -43.8853184
H 11.1563743 20.9672999 -43.3545985
H 10.6901088 20.2389319 -44.9118237
H 10.2204614 19.4614051 -43.3955968
C 8.6444678 21.4437578 -42.4225151
H 9.3296493 22.1190396 -41.9159669
H 8.6253233 20.491972 -41.8909169
H 7.6444986 21.8751438 -42.3818422
N 7.0848025 22.9020958 -45.7835561
H 7.4542566 22.2468272 -46.4660422
C 5.688535 23.2901281 -45.9603944
H 5.2592379 23.5642908 -44.9971347
C 4.9368154 22.090787 -46.5162855
O 5.5175932 21.2799915 -47.2402207
C 5.572196 24.4861637 -46.9093264
H 6.1315988 25.3254024 -46.4927753

H 4.5253632 24.7775338 -47.0057321
O 6.0920421 24.166681 -48.1936715
H 6.9019288 23.6412406 -48.069159
N 3.6649475 21.9328174 -46.1572492
H 3.1924113 22.6509743 -45.6290061
C 2.8540294 20.891737 -46.7718369
H 3.2603949 20.7420939 -47.768523
C 1.4232672 21.3469321 -46.9993605
O 0.9655742 22.2461308 -46.2958749
C 2.8723023 19.5311039 -46.0727033
H 1.8998189 19.3593649 -45.6756641
H 2.831625 18.8431272 -46.9167144
C 4.0238134 18.9905664 -45.2169582
C 4.3071869 17.6253821 -45.3860567
H 3.6888653 17.0224533 -46.0309537
C 4.8919789 19.744078 -44.3979068
H 4.7530479 20.7996534 -44.2590226
C 5.3992881 17.0235059 -44.752768
H 5.5957283 15.9801922 -44.9285487
C 5.9619852 19.1264943 -43.7177794
H 6.5828412 19.700868 -43.0462872
C 6.2154413 17.761582 -43.8888187
H 7.0295482 17.2877361 -43.3583586
N 0.7049211 20.6843602 -47.9071269
H 1.1580329 19.9917536 -48.4999263
C -0.7426341 20.8387991 -48.0115702
H -1.0824204 20.9641876 -46.9898499
C -1.5361428 19.617803 -48.4852348
O -0.9924186 18.6595985 -49.0384563
C -1.11455 22.1121096 -48.8003637
H -1.7026334 21.8618073 -49.6844732
H -0.2187602 22.6483505 -49.119513
C -1.9431406 22.9948283 -47.8556361
O -2.8487189 22.4398679 -47.1824651
O -1.4945848 24.1167787 -47.540539
N -2.8489941 19.6723543 -48.2388324
H -3.2118883 20.5386921 -47.8451067
C -3.8093695 18.7281656 -48.8164363
H -3.3973647 17.7249245 -48.7310293
C -3.9734597 19.0752603 -50.2936677
O -4.3973474 20.175993 -50.6415666
C -5.1732061 18.7435119 -48.0992768
H -5.6730347 19.6950951 -48.2825975
C -6.0637773 17.6068255 -48.6193746
H -5.6052813 16.639463 -48.4108445
H -6.2151491 17.7031978 -49.6940242

H -7.0395154 17.6576898 -48.1394967
C -5.0359728 18.5600012 -46.5826229
H -4.4763312 17.6507279 -46.373113
H -6.0235974 18.4938287 -46.1264681
H -4.5200769 19.4177832 -46.1531065
N -3.6215795 18.1436875 -51.1741785
H -3.2879382 17.2507786 -50.8282856
C -3.6115106 18.3903012 -52.6077545
H -4.5005105 18.9612831 -52.879691
H -2.7342712 18.9845956 -52.864576
C -3.5764643 17.1062873 -53.4192378
O -2.760003 16.2192524 -53.1788795
N -4.4347018 17.0516582 -54.4326559
H -5.0732818 17.8204822 -54.5581563
C -4.3864658 16.05468 -55.4956002
H -4.0915978 15.0912157 -55.0883004
C -3.3390088 16.4662952 -56.5310674
O -3.4495113 17.5140222 -57.164842
C -5.7890218 15.9175663 -56.0942179
H -6.1264015 16.9003061 -56.4288134
H -6.4684305 15.5787826 -55.3090581
C -5.884318 14.9468145 -57.2730016
O -4.8383242 14.4918336 -57.7921059
O -7.0322555 14.7422459 -57.7062923
N -2.3237965 15.6294866 -56.7222922
H -2.3348741 14.7515165 -56.2290766
C -1.1959614 15.917138 -57.5968154
H -0.8042434 16.9026035 -57.3349828
C -1.5477935 15.9520276 -59.0940706
O -0.7986989 16.5414193 -59.8719076
C -0.1261968 14.8713849 -57.2991538
H -0.4332321 13.8964363 -57.6832249
H 0.0211865 14.8034629 -56.2217764
H 0.8044678 15.171372 -57.7763393
N -2.6894224 15.3899282 -59.5099429
H -3.3057519 14.9434825 -58.8268714
C -3.1680469 15.5081525 -60.8889374
H -2.2977069 15.4764396 -61.5426236
C -3.8714604 16.8578481 -61.1353749
O -3.8881519 17.3427345 -62.2717246
C -4.0589673 14.2899356 -61.2019821
H -3.5800509 13.3944349 -60.8022451
H -5.0094949 14.4145144 -60.6792547
C -4.3336983 14.054691 -62.7013104
H -4.8066073 14.9365606 -63.1323418
C -3.0607759 13.732287 -63.4923323

H -2.5395826 12.8897156 -63.037984
H -2.3987076 14.5952794 -63.5160859
H -3.3198034 13.4669686 -64.5171777
C -5.2831101 12.8667808 -62.868187
H -4.8269732 11.9632398 -62.4652122
H -5.5106869 12.7195113 -63.9246257
H -6.2135557 13.0676634 -62.3361046
N -4.3438173 17.5317856 -60.0767708
H -4.239743 17.0913676 -59.1689351
C -5.1392953 18.7670931 -60.1508346
H -5.3101043 19.014173 -61.1954728
C -4.4652452 20.0192241 -59.553186
O -4.7247452 21.1197762 -60.036646
C -6.5275032 18.4864972 -59.5562995
H -7.1041324 19.4099779 -59.5149897
H -6.4332717 18.0886791 -58.546576
C -7.2894454 17.5033969 -60.4354308
O -7.5563357 17.7986585 -61.5883171
N -7.5704357 16.2932746 -60.0008698
H -8.0536807 15.6856149 -60.632247
H -7.3300548 15.9419432 -59.0695682
N -3.5291447 19.8666245 -58.6129897
H -3.3778296 18.9328279 -58.2480178
C -2.7161973 20.948246 -58.0426512
H -3.3974747 21.6988151 -57.6398965
C -1.8381347 21.6244684 -59.108357
O -1.3603305 20.9679296 -60.0376461
C -1.8708616 20.3801509 -56.8857777
H -1.2155043 19.5960797 -57.2688558
H -2.5403692 19.9339514 -56.1488536
C -1.0113161 21.4298223 -56.1713507
H -0.2443703 21.7912918 -56.8567282
H -0.5059511 20.9491715 -55.3327881
S -1.9402459 22.8506813 -55.5394919
C -0.5701828 23.8292679 -54.8690719
H -0.9613234 24.74235 -54.4197375
H 0.1193026 24.0926016 -55.6715556
H -0.0410525 23.252321 -54.1098706
N -1.6015408 22.9369568 -58.9811233
H -1.9423219 23.4054305 -58.1486339
C -0.8192637 23.7390643 -59.9395053
H -0.2576772 23.064157 -60.5851844
C 0.2282464 24.5957342 -59.2270807
O 0.004776 25.0706294 -58.1183271
C -1.7509353 24.6002576 -60.822006
H -2.2485006 25.3361505 -60.1875853

H -1.1470927 25.1432575 -61.5512182
C -2.8355023 23.8041089 -61.576147
H -3.4692309 24.5095959 -62.1148217
H -3.4670243 23.293783 -60.85025
C -2.285634 22.7794283 -62.5838831
H -1.469829 22.2166229 -62.1362081
H -1.8727529 23.3160784 -63.440655
N -3.3480238 21.8600974 -63.0454371
H -4.1592275 22.2924263 -63.4494333
C -3.3712553 20.539867 -62.9367282
N -4.3632524 19.8552986 -63.436589
H -4.394121 18.8635073 -63.2050823
H -5.2093213 20.3214733 -63.7056537
N -2.4470361 19.8412767 -62.3456859
H -1.8072108 20.3142319 -61.7116381
H -2.5589807 18.8412342 -62.2636225
N 1.3477479 24.8147152 -59.9086339
H 1.3980397 24.4714973 -60.8561952
C 2.4801481 25.6382775 -59.4817666
H 2.171737 26.310206 -58.6796175
C 2.9540436 26.4820188 -60.6742369
O 2.5493109 26.2124622 -61.8048482
C 3.6101897 24.7291168 -58.9715134
H 3.9556652 24.0994097 -59.7921348
H 4.4519802 25.3525014 -58.6710504
C 3.2414508 23.8502424 -57.790578
C 3.2255769 24.3988016 -56.4940977
H 3.4578802 25.4439975 -56.3432923
C 2.944317 22.484486 -57.9805326
H 2.957988 22.0603603 -58.9749413
C 2.9118273 23.5871558 -55.3886793
H 2.893409 23.9989979 -54.390721
C 2.6376701 21.6672874 -56.8746023
H 2.4174114 20.6212776 -57.0198648
C 2.6196309 22.2218223 -55.5761608
O 2.3179465 21.4526177 -54.4997302
H 2.18435 20.5319836 -54.724826
N 3.8334992 27.4628261 -60.4509498
H 4.2082822 27.584594 -59.5219213
C 4.4939213 28.1620494 -61.5616058
H 3.7309422 28.5488971 -62.2384471
C 5.3882736 27.2020676 -62.3485732
O 6.0176283 26.3114741 -61.7616727
C 5.3217809 29.3477141 -61.0436929
H 6.091829 28.9980095 -60.3569351
H 4.6612574 30.029898 -60.5071673

O 5.9393037 30.0538829 -62.1032955
H 6.7243398 29.5613144 -62.4093461
N 5.5820288 27.4940815 -63.6317522
H 5.0106585 28.1958685 -64.0746187
C 6.7307802 26.9874338 -64.3760478
H 6.6366855 25.9085016 -64.4254029
C 8.028407 27.3558119 -63.6304401
O 8.1187891 28.4400932 -63.0417593
C 6.7479946 27.5450176 -65.8100117
H 7.719706 27.3149662 -66.2505881
H 6.6485008 28.6315395 -65.7731873
C 5.6672195 26.9697377 -66.7358325
O 4.772257 26.2412801 -66.2508656
O 5.7522914 27.2543315 -67.9504335
N 9.0046514 26.4438149 -63.5827792
H 8.8767 25.5757138 -64.0999271
C 10.2928452 26.6834173 -62.9168859
H 10.9628395 25.863104 -63.1596956
H 10.726691 27.5994604 -63.3176861
C 10.2581107 26.8168125 -61.3836782
O 11.1929088 27.3706979 -60.812188
N 9.2068629 26.3463782 -60.7000323
H 8.4713915 25.8971 -61.2280263
C 9.0731089 26.4374344 -59.2283582
H 9.1851045 27.4791121 -58.9258279
C 10.1069491 25.6136815 -58.4432941
O 10.4836519 25.994552 -57.3288833
C 7.6897912 25.9495096 -58.7796666
H 7.6527518 25.9341602 -57.6888603
H 7.5223822 24.9374057 -59.148062
O 6.6580577 26.7898418 -59.2502181
H 6.5249451 26.5892104 -60.1998395
N 10.5092653 24.4553591 -58.9775317
H 10.148396 24.2047745 -59.8925566
C 11.2703098 23.4359545 -58.2511685
H 11.53296 23.831252 -57.2725145
C 12.5826537 23.0539197 -58.9343996
O 12.7237566 23.0892687 -60.153064
C 10.4016771 22.1945978 -58.0069762
H 10.1678104 21.7295215 -58.9665107
H 10.9725225 21.4720044 -57.4234963
C 9.1174901 22.4918067 -57.2616053
C 9.1440574 22.7882535 -55.8861933
H 10.0770687 22.7663086 -55.3446341
C 7.8965917 22.5106125 -57.9559699
H 7.8822044 22.2996273 -59.0114969

C 7.9532069 23.1130515 -55.2127899
H 7.9771022 23.3415634 -54.1554797
C 6.7052395 22.8277978 -57.284616
H 5.7704859 22.841606 -57.8236995
C 6.7337942 23.135257 -55.9133376
H 5.8189192 23.3828901 -55.3936062
N 13.517583 22.5839715 -58.1188466
H 13.3072881 22.5350507 -57.1278463
C 14.8179288 22.0601079 -58.5444263
H 15.0314648 22.3351386 -59.5799209
C 14.8820061 20.5283609 -58.4571595
O 15.7122647 19.8905221 -59.1081171
C 15.861854 22.6970743 -57.6264017
H 15.4958654 22.6849266 -56.6000881
H 16.7664742 22.1004187 -57.6568063
C 16.1785143 24.1454572 -58.0345006
H 16.4935678 24.6931106 -57.143149
H 15.2907343 24.6425045 -58.4312527
C 17.305602 24.1476694 -59.067901
O 17.1190339 23.5958259 -60.1692975
O 18.44763 24.4781666 -58.6847995
N 14.0008482 19.9431131 -57.6415347
H 13.375073 20.5442361 -57.11928
C 13.8557196 18.5135042 -57.3961515
H 14.3000178 17.9505765 -58.2132188
C 12.3562485 18.1809218 -57.2916426
O 11.6055412 18.9392478 -56.6695756
C 14.5613807 18.1437631 -56.0764476
H 13.9703084 18.5856413 -55.2756477
H 14.5331341 17.0604963 -55.9537592
C 16.0075358 18.6382877 -55.8749913
H 16.0774433 19.7022195 -56.0961422
C 16.4272853 18.4705187 -54.4170553
H 16.4803628 17.4129 -54.1558347
H 15.7119752 18.972793 -53.7650525
H 17.3987076 18.9361346 -54.2814031
C 17.0207935 17.8886108 -56.7338335
H 16.9321508 16.814618 -56.5697651
H 18.0321975 18.2073195 -56.4892937
H 16.8337156 18.1120355 -57.7796173
N 11.9268476 17.0254245 -57.802589
H 12.5667476 16.4679935 -58.3580903
C 10.6134922 16.4408486 -57.4727742
H 10.200647 16.9817986 -56.6240596
C 10.8460628 14.9974708 -57.0338666
O 11.4968357 14.2196056 -57.7343886

C 9.584129 16.5453119 -58.623281
H 9.8891 15.8453796 -59.384544
C 9.5544193 17.9798466 -59.2141413
H 9.2019235 18.6905423 -58.4697116
H 10.5636784 18.2729503 -59.500348
C 8.1844474 16.0604285 -58.2049168
H 7.7911132 16.6907992 -57.4099126
H 8.2346799 15.0332518 -57.8434264
H 7.5072373 16.083636 -59.0554359
C 8.7221937 18.1222675 -60.4791129
H 7.6661354 18.1114382 -60.2270789
H 8.9737782 17.3097286 -61.1536164
H 8.9770545 19.0592215 -60.9698104
N 10.3564225 14.6574374 -55.8469035
H 9.7941865 15.3397182 -55.3446681
C 10.5610505 13.361721 -55.2088406
H 11.1527113 12.721799 -55.8623133
C 9.2265463 12.6740554 -54.9607565
O 8.2716464 13.3041168 -54.5053329
C 11.33476 13.5316808 -53.9000093
H 12.3374437 13.8914798 -54.1303004
H 10.8329836 14.2807638 -53.2847405
C 11.4560679 12.2414929 -53.107116
C 12.2584577 11.1893237 -53.5913009
H 12.8093437 11.3079028 -54.5115507
C 12.3285074 9.9712448 -52.892086
H 12.935959 9.1595666 -53.2719667
C 11.6002187 9.80203 -51.7022888
H 11.6597189 8.8679635 -51.1608939
C 10.7885537 10.8448729 -51.2227153
H 10.2173261 10.7141468 -50.3149552
C 10.7115946 12.0603749 -51.9250526
H 10.0647863 12.8469327 -51.5572318
N 9.1602094 11.3777451 -55.2613215
H 9.967209 10.9333375 -55.6892098
C 7.9328766 10.5948167 -55.1189933
H 7.301889 11.0701356 -54.3646456
C 8.255057 9.1831792 -54.6477118
O 9.0891373 8.509012 -55.2435789
C 7.1597415 10.5440188 -56.4471444
H 7.7114147 9.9222563 -57.1494066
C 5.8095002 9.8629732 -56.2113163
H 5.4896513 10.0212756 -55.1882349
H 5.9140703 8.7885214 -56.3956002
H 5.0585275 10.2781186 -56.8705113
C 7.0334598 11.9183157 -57.1168015

H 6.7794035 12.6694729 -56.3779092
H 6.2773547 11.9109892 -57.8926942
H 7.9869524 12.1928344 -57.5734035
N 7.5304832 8.6922801 -53.6430702
H 6.8760449 9.3036927 -53.1726615
C 7.6681163 7.3252057 -53.1358409
H 8.2068826 6.7406371 -53.8801015
C 6.2876119 6.6673452 -52.9862792
O 5.4097723 7.1751835 -52.2984167
C 8.5623854 7.3067255 -51.8731256
H 9.5579647 7.6240205 -52.1890165
C 8.094785 8.2976582 -50.7891014
H 7.0661867 8.0877915 -50.5071326
H 8.1462975 9.3195321 -51.1659953
H 8.7354529 8.2405992 -49.9112774
C 8.6896144 5.865025 -51.3446308
H 7.7397634 5.5693468 -50.9079072
H 8.8984866 5.198318 -52.1818034
C 9.804598 5.6643442 -50.3096542
H 9.5898272 6.2220642 -49.3977556
H 10.7584064 5.9929064 -50.7211156
H 9.8773923 4.6049462 -50.0589894
N 6.0735872 5.5791504 -53.7322323
H 6.8322139 5.3057652 -54.3480851
C 4.8587987 4.7465541 -53.7745885
H 4.9169297 4.1923917 -54.7089597
C 3.516118 5.5057891 -53.8614048
O 2.5191161 5.1118456 -53.2532807
C 4.8607276 3.7088065 -52.6438668
H 4.6254463 4.2273965 -51.7263386
H 4.0580549 2.9937707 -52.8329263
C 6.1573545 2.9237375 -52.4338794
H 5.9667513 2.1123083 -51.7318169
H 6.9089625 3.5794581 -51.9982039
C 6.7097361 2.3381834 -53.7201912
O 7.8204849 2.6367907 -54.124086
N 5.9596121 1.5408023 -54.4434662
H 6.3860314 1.1601171 -55.2659575
H 4.9443976 1.5731702 -54.3602218
N 3.4922299 6.609794 -54.6151327
H 4.3734201 6.9183661 -54.9925454
C 2.3102518 7.4619524 -54.7883182
H 1.523502 7.1422596 -54.1042785
C 1.7272705 7.3825057 -56.209906
O 0.5213259 7.2247492 -56.3878293
C 2.7044591 8.890572 -54.3960494

H 3.4554001 9.2689719 -55.0839756
H 3.1257726 8.8895343 -53.3873539
S 1.267988 9.9956678 -54.455393
H 1.9581013 11.1116626 -54.1764562
N 2.5785643 7.3956426 -57.2408166
H 3.5667076 7.4519611 -57.0465178
C 2.1412431 7.4998195 -58.6453044
H 1.3706166 8.2684934 -58.6961616
C 1.455654 6.224998 -59.1639819
O 0.6846533 6.2812467 -60.1185169
C 3.3271499 7.9979964 -59.498981
H 4.2271993 7.4604443 -59.1935633
C 3.5120954 9.5030321 -59.2234801
H 2.7433385 10.070109 -59.7525158
H 3.3888159 9.7067547 -58.1618482
C 3.1360484 7.7830679 -61.0060698
H 2.1735433 8.1823495 -61.3276595
H 3.2063335 6.7193164 -61.2171017
H 3.9295564 8.2650958 -61.5743967
C 4.8911054 10.0097662 -59.6504475
H 5.0099305 9.9628237 -60.7314375
H 5.6735898 9.4236341 -59.1685517
H 4.9797716 11.0455733 -59.3443017
N 1.5717582 5.1041732 -58.4564899
H 2.1827045 5.1206104 -57.6529454
C 0.7328439 3.9149034 -58.651388
H 0.8947388 3.5462889 -59.6639284
C -0.7823536 4.1812482 -58.5331897
O -1.5480582 3.5308124 -59.2486143
C 1.1982313 2.8047712 -57.6992863
H 0.5310809 1.9524817 -57.8022436
H 2.1807305 2.4791663 -58.0396875
C 1.2976814 3.1948038 -56.2119626
H 0.3012642 3.1688227 -55.7657597
H 1.6944627 4.205053 -56.0979221
C 2.2384522 2.2254211 -55.4939914
O 3.4720441 2.4194285 -55.590334
O 1.7426194 1.2099946 -54.9540701
N -1.2002262 5.2068174 -57.7761104
H -0.5129244 5.7123035 -57.2254629
C -2.598939 5.6605511 -57.6700557
H -3.2650489 4.7962077 -57.7183515
C -3.0249164 6.6303356 -58.7861373
O -4.212663 6.7435325 -59.0897442
C -2.8084363 6.3407486 -56.3074555
H -3.8525083 6.6461592 -56.2269157

H -2.1951503 7.2411331 -56.2540386
C -2.494111 5.4648105 -55.1222584
N -3.2748369 4.3995001 -54.6813414
C -1.402258 5.5724542 -54.3123075
H -0.6083186 6.2963122 -54.4092074
C -2.6439835 3.8919381 -53.61534
H -2.9894624 3.0311948 -53.0587202
N -1.5076655 4.5702295 -53.3754836
H -0.8044458 4.3204002 -52.6953078
N -2.0794667 7.3391896 -59.4136958
H -1.1158875 7.1556197 -59.1719981
C -2.3616997 8.3287098 -60.4667779
H -3.2276859 8.9173499 -60.1602128
C -2.7464924 7.5875773 -61.7548098
O -2.001621 6.7304906 -62.2333305
C -1.169923 9.3091347 -60.6337605
H -0.2399088 8.7535995 -60.5132491
C -1.1908147 10.461597 -59.5993055
H -0.3002541 11.0742561 -59.749503
H -2.0610367 11.0934907 -59.7853356
C -1.1248122 9.968303 -62.0256172
H -2.0519598 10.5058078 -62.2273489
H -0.9656117 9.221869 -62.8030621
H -0.2929621 10.6711498 -62.0761009
C -1.2165483 10.0459058 -58.1225757
H -0.4028053 9.3553923 -57.9105932
H -2.1699243 9.5788426 -57.8756608
H -1.0975652 10.9321865 -57.4986943
N -3.9191623 7.9002296 -62.3131057
H -4.4943561 8.5906527 -61.8567087
C -4.4811881 7.1647627 -63.4544377
H -4.2521094 6.1048541 -63.3300885
C -3.8496852 7.5951677 -64.7889643
O -3.2727482 6.7640869 -65.4867586
C -6.0132403 7.3088003 -63.4796484
H -6.2694267 8.3537053 -63.6622205
H -6.4039576 6.7184002 -64.3096062
C -6.7166646 6.8608593 -62.1861027
H -7.7887787 7.0203155 -62.3018223
H -6.3872767 7.4812947 -61.3521355
C -6.4970237 5.3889815 -61.8459751
O -6.9995508 4.4963847 -62.5114945
N -5.7572628 5.0902401 -60.8042215
H -5.833909 4.14822 -60.4307188
H -5.3482376 5.8097848 -60.2156199
N -3.8569681 8.8981386 -65.0946658

H -4.3202539 9.5439063 -64.4785248
C -3.1401507 9.4583446 -66.2477274
H -3.0411782 8.6833109 -67.0107969
C -1.7236527 9.8698317 -65.8282051
O -1.4618108 10.9868417 -65.364135
C -3.9305454 10.5951731 -66.9132344
H -4.0322571 11.4261952 -66.2131145
H -4.9304066 10.2295566 -67.1538309
C -3.2659619 11.0967388 -68.2087631
O -2.0957811 10.7292587 -68.4770125
O -3.9030893 11.919111 -68.8986723
N -0.803794 8.9111941 -65.9612056
H -1.116004 8.0117478 -66.302686
C 0.6148988 9.1220086 -65.6716635
H 0.701243 9.6227611 -64.7065084
C 1.2656105 10.0741342 -66.6689175
O 2.098196 10.8648421 -66.2441968
C 1.3529267 7.7757301 -65.5926566
H 1.1126767 7.1689474 -66.4679944
H 2.4297305 7.955249 -65.5762918
C 0.9574017 7.0366377 -64.3062496
H 1.2856483 7.6316669 -63.4540904
H -0.1280695 6.9419732 -64.2625537
C 1.5717334 5.633152 -64.2128231
H 2.6407791 5.6991469 -64.4166036
H 1.1128656 4.9710833 -64.9484462
C 1.3819517 5.0770151 -62.7971826
H 1.7874912 5.8138986 -62.1066302
H 1.9538723 4.1524789 -62.6816544
N -0.0351696 4.8430507 -62.4374898
H -0.6226182 5.656999 -62.6128357
H -0.1159741 4.6848988 -61.4413045
H -0.410129 4.0257107 -62.9061462
N 0.857613 10.1083443 -67.9375912
H 0.0306158 9.5949737 -68.2147783
C 1.4627276 11.0419099 -68.8908006
H 2.5495178 10.9536548 -68.8456466
C 1.0955327 12.4971915 -68.5945729
O 1.9570158 13.368256 -68.6695282
C 1.0240044 10.7936445 -70.3246517
H -0.0383867 11.0268319 -70.4208665
H 1.5987591 11.5133366 -70.9013528
C 1.2870139 9.4334093 -70.9591885
H 0.6207275 8.6875652 -70.5204886
H 2.321262 9.1555211 -70.7495981
C 1.0670261 9.5267931 -72.4818624

O 1.5837226 8.6346019 -73.1863309
O 0.449561 10.5278113 -72.9382914
N -0.1651418 12.7909911 -68.2489103
H -0.8538143 12.0316837 -68.2624408
C -0.597046 14.1400191 -67.8500139
H -0.3606733 14.8368522 -68.6493023
C 0.1788132 14.6128788 -66.6331437
O 0.6875716 15.7306213 -66.6664212
C -2.1126775 14.1466946 -67.5941196
H -2.6227997 14.0571914 -68.5530931
H -2.3576696 13.272807 -66.9910062
C -2.6896842 15.3612165 -66.8459386
H -3.7652708 15.2087699 -66.7430743
H -2.2667664 15.3935762 -65.8416272
C -2.4521123 16.7168604 -67.5216241
H -2.901966 16.7154243 -68.5155877
H -1.3820194 16.9074539 -67.6060141
C -3.0951271 17.8042082 -66.6541248
H -2.7560098 17.6658543 -65.6223159
H -4.1813943 17.6762267 -66.6795216
N -2.71647 19.159312 -67.1134731
H -1.700728 19.2804625 -67.0277463
H -3.1123092 19.8764223 -66.5258363
H -2.9676407 19.3212056 -68.075571
N 0.3204954 13.766832 -65.6119406
H -0.1301998 12.8600258 -65.6626692
C 1.1372004 14.0988161 -64.4489276
H 0.7681291 15.0311553 -64.0186157
C 2.6010132 14.3488103 -64.8440332
O 3.1203401 15.4231067 -64.5515805
C 1.0048208 13.0052398 -63.3833035
H -0.0274297 12.9715737 -63.0338871
H 1.2416141 12.0362933 -63.8251743
C 1.9196494 13.2544928 -62.2026704
C 1.5902525 14.2330469 -61.2449909
H 0.6507624 14.7645696 -61.3091331
C 2.5054578 14.5589645 -60.2283914
H 2.2645 15.3361693 -59.5185997
C 3.7477584 13.9046379 -60.163313
H 4.4610224 14.1635952 -59.3939693
C 4.075634 12.9324819 -61.1222156
H 5.0420339 12.4538226 -61.0965527
C 3.1634757 12.6011592 -62.1355357
H 3.4384687 11.8742898 -62.8856969
N 3.2219607 13.4431795 -65.6129092
H 2.7236004 12.5935768 -65.8627977

C 4.6285742 13.5613066 -66.0272132
H 5.2190656 13.6913037 -65.136371
C 4.8554892 14.8103973 -66.9071792
O 5.7538651 15.5982238 -66.6248052
C 5.1469566 12.2423806 -66.6375441
H 4.5264298 12.0049949 -67.503892
C 6.6112232 12.3569321 -67.0848273
H 7.294004 12.1712733 -66.2581363
H 6.8394987 13.3368053 -67.4929083
H 6.78462 11.6171695 -67.8603109
C 5.0988351 11.0728698 -65.6156212
H 5.8917595 11.1882859 -64.8758281
H 4.1709873 11.0890031 -65.0529447
C 5.2309691 9.6913749 -66.2729081
H 6.195366 9.5950969 -66.7695518
H 4.4343643 9.5515472 -67.0036455
H 5.1519131 8.9179254 -65.5089451
N 3.9636351 15.1292163 -67.8520424
H 3.2265652 14.4600239 -68.0547949
C 4.0218868 16.3871815 -68.6226211
H 5.0042765 16.4596209 -69.0901891
C 3.8797531 17.6361744 -67.751329
O 4.3853571 18.6900604 -68.1393617
C 2.9423549 16.3991231 -69.7126516
H 1.9845357 16.1193492 -69.2705717
H 2.8543825 17.4158555 -70.0989016
C 3.2701847 15.4763868 -70.8965743
H 3.2834725 14.434007 -70.5792135
H 4.2600221 15.7274747 -71.2810722
C 2.2462379 15.6651832 -72.0260354
H 2.5661878 15.0769262 -72.8877034
H 2.2537104 16.7118842 -72.3344896
N 0.873631 15.2917612 -71.613203
H 0.3736673 15.9475456 -71.0440993
C 0.2606047 14.1451865 -71.8617678
N 0.8324717 13.2109762 -72.562001
H 0.4215544 12.2750905 -72.7048818
H 1.7817286 13.3170273 -72.8573687
N -0.9394563 13.9204432 -71.4047869
H -1.4254517 14.5628763 -70.815964
H -1.3235025 12.9947997 -71.5411287
N 3.1468849 17.5726836 -66.640555
H 2.7147858 16.6893069 -66.3908726
C 3.0422072 18.6861717 -65.6891141
H 2.9536692 19.6115987 -66.2544648
C 4.3172776 18.8331359 -64.8542892

O 4.818539 19.9522103 -64.7316767
C 1.7891423 18.5422215 -64.8007602
H 1.0555546 17.9044931 -65.2924164
H 2.0612306 18.0606817 -63.8602092
C 1.1195113 19.8957002 -64.5028273
H 1.8473703 20.5638183 -64.0369374
H 0.3048766 19.7346467 -63.7929669
C 0.5491297 20.5310302 -65.7790337
O -0.2282403 19.8629453 -66.4986209
O 0.9457575 21.6510811 -66.1609826
N 4.94132 17.725144 -64.4234338
H 4.4998686 16.8199543 -64.5638902
C 6.2140599 17.8019982 -63.6909489
H 6.0524835 18.5815138 -62.9562327
C 7.3890477 18.3481159 -64.518059
O 8.2732855 18.9822581 -63.9445966
C 6.5604161 16.5360761 -62.8697259
H 7.6036421 16.612572 -62.5826921
C 5.687129 16.5745079 -61.5928064
H 4.6438896 16.3754152 -61.8451636
H 5.7360653 17.5498829 -61.1123979
H 6.0256541 15.83006 -60.8757306
C 6.3611885 15.1622226 -63.5084856
H 5.298497 14.9474787 -63.5301493
H 6.74844 15.1843201 -64.5161972
C 7.0610066 14.0140301 -62.7739185
H 6.6684855 13.8981371 -61.7667884
H 8.122155 14.2216184 -62.7084251
H 6.9110273 13.0840706 -63.3228865
N 7.30301 18.2991724 -65.852446
H 6.594574 17.6978391 -66.2541671
C 8.2430857 18.9945263 -66.7521705
H 9.256973 18.7089679 -66.4899572
C 8.1690656 20.51075 -66.5818082
O 9.1990222 21.1611148 -66.460308
C 7.977772 18.6399987 -68.229651
H 6.9892592 19.0220523 -68.484297
C 8.9845327 19.2914653 -69.1874602
H 10.0014391 18.9979181 -68.9194354
H 8.9126701 20.3778295 -69.1491886
H 8.7886603 18.966429 -70.2109033
C 7.96589 17.1408008 -68.5243393
H 8.943109 16.8146886 -68.8779901
H 7.2357508 16.9438971 -69.3046957
H 7.7171778 16.551408 -67.6508645
N 6.9615765 21.0893303 -66.532496

H 6.1488449 20.486938 -66.5022801
C 6.780615 22.5474602 -66.4135413
H 7.4330732 23.0541204 -67.1286945
C 7.2202667 23.0717325 -65.0569848
O 7.912679 24.0815246 -64.9821131
C 5.3159302 22.9327156 -66.6760503
H 4.6831159 22.049465 -66.5844177
H 4.9780527 23.653042 -65.9286919
C 5.1585947 23.5769347 -68.0589042
H 5.5905341 22.9257093 -68.8152424
H 5.7275276 24.5057287 -68.0837541
C 3.6905468 23.8966561 -68.3952165
H 3.6380314 24.2920585 -69.4108383
H 3.3606373 24.6917945 -67.7218206
N 2.7656957 22.7454938 -68.2527494
H 2.0283473 22.845749 -67.5579178
C 2.88091 21.5274568 -68.7454514
N 3.8693124 21.140862 -69.4991559
H 4.0949378 20.1565639 -69.4957941
H 4.5709032 21.8214323 -69.7161739
N 1.9903342 20.6334323 -68.4586807
H 1.2955849 20.8980357 -67.7519289
H 2.1477213 19.6774308 -68.6872359
N 6.8385406 22.3850171 -63.9793491
H 6.2678414 21.5594362 -64.1188139
C 7.0762139 22.8882716 -62.6154744
H 6.7536171 23.9302454 -62.5989197
C 8.5529194 22.901266 -62.209056
O 8.8936518 23.5075295 -61.1960446
C 6.2302789 22.1490035 -61.5659052
H 6.3002834 22.7158685 -60.6375336
C 4.7406745 22.1370212 -61.9312552
H 4.5608851 21.5072413 -62.7992222
H 4.4104188 23.1524728 -62.155835
H 4.1599278 21.7513496 -61.0951958
C 6.7187884 20.740197 -61.2639461
H 6.946144 20.2365441 -62.1944734
H 5.9697426 20.1820863 -60.7022476
H 7.6299397 20.7966649 -60.6732389
N 9.4330305 22.2598839 -62.9727844
H 9.1190088 21.8290885 -63.831774
C 10.8586508 22.2157185 -62.6987607
H 11.0205601 22.3896924 -61.6385775
C 11.6587618 23.2613323 -63.4943908
O 11.250341 23.686733 -64.571033
C 11.3162717 20.8126336 -63.009183

H 11.2222436 20.673128 -64.0870845
H 10.6948542 20.0962774 -62.4706286
H 12.34619 20.6925253 -62.698318
N 12.8048385 23.6814376 -62.9622398
H 13.0708624 23.3246349 -62.0518115
C 13.7495271 24.5578181 -63.6509041
H 13.1778 25.2862108 -64.2238724
C 14.6719354 23.7794981 -64.6223379
O 14.975169 22.6149417 -64.3663321
C 14.5534374 25.3133525 -62.584314
H 15.1349368 24.6050227 -61.9900518
H 13.8784704 25.8645982 -61.9278724
H 15.2402197 26.0126728 -63.0612602
N 15.2562672 24.4346856 -65.6428965
C 16.3457623 23.8565942 -66.4329207
H 15.9645131 23.0084207 -66.9972777
C 17.5084646 23.3942187 -65.5437926
O 17.9666931 24.1420647 -64.6815619
C 16.7604415 24.9539896 -67.41675
H 17.5396302 25.5804047 -66.9808329
H 17.0916402 24.5356973 -68.368371
C 15.4800201 25.7739039 -67.5748403
H 14.8102338 25.2746548 -68.2775496
H 15.6876092 26.7949285 -67.8961341
C 14.8755827 25.7311526 -66.1737705
H 13.7917232 25.8368128 -66.2352599
H 15.3069372 26.5190763 -65.555338
N 17.9761557 22.1537945 -65.7184936
H 17.5578649 21.5750566 -66.4370437
C 18.9691881 21.5330127 -64.828799
H 19.4528712 20.7173447 -65.3653614
H 19.7268063 22.271958 -64.5676816
C 18.3945524 20.9579177 -63.5231969
O 19.1431088 20.4234825 -62.6929978
N 17.0763422 20.9995032 -63.325819
H 16.4871733 21.5126475 -63.9742723
C 16.4200515 20.282351 -62.2438022
H 16.8992307 20.5608404 -61.3042546
C 16.5430015 18.7673145 -62.4295642
O 16.5979498 18.2344241 -63.5433115
C 14.9511917 20.6876016 -62.1594518
H 14.4759675 20.4721613 -63.1160417
H 14.876111 21.7519627 -61.935609
H 14.4412873 20.1255796 -61.3775577
N 16.4551063 18.0541021 -61.3125826
H 16.2855776 18.5690471 -60.4511779

C 16.3207142 16.6066863 -61.2672556
H 16.667364 16.1436285 -62.1925943
C 14.8430527 16.4876811 -61.1714432
O 14.2703098 16.4918358 -60.0760996
C 17.1025975 16.0055349 -60.0967373
H 16.7267382 16.4254048 -59.1674263
H 16.9332383 14.927969 -60.0696969
C 18.6151246 16.2789271 -60.2035818
H 18.7934009 17.3000794 -60.5397025
H 19.0498173 15.6006874 -60.9384452
C 19.3309473 16.1098317 -58.8681021
O 19.8209138 17.063498 -58.2767724
N 19.4280626 14.9016131 -58.3528361
H 19.9160454 14.8206799 -57.47876
H 19.0269415 14.1074667 -58.8221429
N 14.2900884 16.7928196 -62.3621341
H 14.9082487 16.9578584 -63.1449686
C 12.8660165 16.8921841 -62.5786515
H 12.50313 17.8129391 -62.118628
C 12.3945187 16.8195691 -64.0583579
H 11.5182164 16.1806944 -64.1205188
C 11.927267 18.2080647 -64.4477732
H 12.7140434 18.9289267 -64.2293846
H 11.0102521 18.4103486 -63.8975376
H 11.6871596 18.2879955 -65.5028421
C 13.3973882 16.3601632 -65.1198995
H 13.1242213 16.743578 -66.1014841
H 14.3632553 16.7589904 -64.8585091
C 13.4802412 14.8541873 -65.2635705
H 12.5137973 14.4524802 -65.5470749
H 13.7913259 14.4259101 -64.3196536
H 14.1809111 14.6061911 -66.0551717
C 12.2832975 15.764129 -61.7971147
O 11.2494559 16.0014477 -61.1925983
N 12.9953431 14.6160479 -61.6922139
H 13.8487719 14.4634771 -62.2140222
C 12.4409562 13.5648977 -60.9104669
H 11.9661348 14.0429408 -60.056893
C 13.3950247 12.5287825 -60.3251963
O 14.3898634 12.1321749 -60.9337501
C 11.3641755 12.9163869 -61.7760491
H 11.69877 11.9263678 -62.0871113
C 10.1448767 12.7675339 -60.8818847
H 9.7797381 13.7482455 -60.5753726
H 10.389334 12.200937 -59.985975
H 9.3515675 12.2659959 -61.4236928

C 10.8142608 13.647437 -63.0167973
H 10.2651247 14.5505195 -62.7809551
H 10.1246692 12.9691349 -63.5158578
H 11.6027534 13.8396563 -63.7244018
N 12.992268 12.0624941 -59.1407394
H 12.2290494 12.5696896 -58.7005972
C 13.3517678 10.7991302 -58.4875107
H 13.8691189 10.1454328 -59.1901295
C 12.022295 10.1409558 -58.0722694
O 11.2124517 10.7801555 -57.3905926
C 14.2732964 11.0619765 -57.2714117
H 13.7556098 11.7467959 -56.5983282
C 14.5539489 9.7552096 -56.5040559
H 15.098085 9.0547942 -57.1393494
H 13.6255968 9.2720965 -56.1977078
H 15.1326487 9.9473315 -55.6028041
C 15.5967541 11.7351388 -57.7104154
H 16.1322448 11.0671286 -58.3834904
H 15.3704105 12.6510699 -58.2557262
C 16.5299331 12.1281646 -56.5575202
H 16.9353557 11.2398739 -56.0737671
H 15.9862613 12.7290053 -55.8275692
H 17.3598219 12.7148318 -56.9523203
N 11.7580335 8.9046503 -58.5018071
H 12.4971308 8.4049395 -59.0006782
C 10.4983895 8.1867901 -58.2471085
H 9.9675434 8.6814518 -57.4396159
C 10.7986262 6.7492106 -57.8252037
O 11.102295 5.9443846 -58.7003625
C 9.5576584 8.1569015 -59.488233
H 10.0657489 7.6118162 -60.2850846
C 9.2280089 9.5527545 -60.0531933
H 8.7489912 10.1650888 -59.2892799
H 10.1684049 10.018405 -60.3410172
C 8.2538598 7.3944316 -59.1507915
H 7.707404 7.913199 -58.3643917
H 8.4738782 6.3822778 -58.8197286
H 7.6197814 7.2880813 -60.0276344
C 8.3419087 9.5094014 -61.311817
H 7.3388524 9.1658198 -61.0701747
H 8.7868381 8.8466241 -62.055071
H 8.2449684 10.4990386 -61.7479271
N 10.3778145 6.3647875 -56.6173089
H 10.0867171 7.0875804 -55.969359
C 10.0771669 4.965996 -56.2829504
H 10.6161371 4.3342597 -56.978693

C 8.5735844 4.707958 -56.4325046
O 7.7547345 5.5273144 -56.0055968
C 10.6004787 4.5910992 -54.8968489
H 10.1061673 5.1945444 -54.1366728
H 11.6735419 4.7889482 -54.8607758
O 10.3828632 3.2140938 -54.6418497
H 9.4472056 3.0366627 -54.4338089
N 8.1607001 3.6309068 -57.1055167
H 8.8769937 2.9804449 -57.4273788
C 6.7371884 3.300688 -57.3620578
H 6.1596131 3.5083897 -56.4621738
C 6.5491433 1.8064488 -57.6597888
O 7.4787363 1.1227106 -58.0834072
C 6.1822375 4.201798 -58.4902398
H 6.9381585 4.3099942 -59.2694911
C 4.8723056 3.766805 -59.1432156
H 4.1171477 3.6139869 -58.3752925
H 5.0235349 2.8460121 -59.7068042
H 4.5357146 4.5394803 -59.8340945
O 5.8902408 5.4700017 -57.9372644
H 6.6083844 5.6589023 -57.3062582
N 5.3715908 1.2506011 -57.3572631
H 4.6553821 1.8125038 -56.8983762
C 5.0533527 -0.1448405 -57.6414244
H 5.9175855 -0.7539901 -57.3692877
C 4.7686066 -0.3651727 -59.1348527
O 3.9382459 0.3149866 -59.7426248
C 3.8790949 -0.6020956 -56.7719286
H 2.9852171 -0.0491227 -57.0590648
H 4.0992285 -0.4156114 -55.7198245
H 3.7121333 -1.6687299 -56.9224242
N 5.384449 -1.4132231 -59.6710028
H 5.9615485 -1.978817 -59.0585404
C 5.274073 -1.8707675 -61.0481607
H 4.52063 -1.2819514 -61.5709594
C 4.819635 -3.3368143 -61.111532
O 4.71484 -4.0327033 -60.0985856
C 6.6290324 -1.6468094 -61.7413333
H 6.7247861 -2.2962304 -62.6122492
H 7.4480343 -1.8571985 -61.0513851
S 6.7161264 0.0744788 -62.2988248
H 5.7073454 -0.0156779 -63.1767116
N 4.5804398 -3.8163232 -62.3306187
H 4.6770828 -3.1691214 -63.1064769
C 4.346574 -5.2294842 -62.6551505
H 4.459447 -5.8264837 -61.7510928

C 5.4032849 -5.7429896 -63.6329606
O 6.1897972 -4.9572822 -64.1649364
C 2.9059161 -5.4309908 -63.1662228
H 2.7672556 -6.4849372 -63.4143421
H 2.2061259 -5.1920836 -62.3647088
C 2.5629917 -4.5759187 -64.3962885
H 3.3538419 -4.6507815 -65.1381943
H 2.4774119 -3.5317996 -64.1082129
C 1.239358 -5.0199314 -65.0200982
H 0.4409992 -4.8935195 -64.2851521
H 1.2911458 -6.0804497 -65.2787751
N 0.9258565 -4.2126199 -66.212061
H 0.4081314 -3.3590145 -66.0351975
C 1.2259418 -4.5210722 -67.4631425
N 1.9688723 -5.5468659 -67.7776155
H 2.1152903 -5.8721481 -68.7219343
H 2.3570204 -6.1389378 -67.0480665
N 0.7801215 -3.779182 -68.4391839
H 0.1596895 -3.0162654 -68.2328983
H 0.9754474 -4.0537471 -69.3841574
N 5.3899017 -7.0384504 -63.929528
H 4.7383203 -7.6344439 -63.4363782
C 6.0942102 -7.5596355 -65.1036889
H 7.0977374 -7.1300138 -65.1006469
C 5.4232967 -7.0925274 -66.4226341
O 4.3354509 -6.5106139 -66.4170726
C 6.2677109 -9.0838745 -64.9594063
H 6.6936781 -9.2945219 -63.9792089
H 6.9785677 -9.4402086 -65.7029615
C 4.9912565 -9.885612 -65.1365704
O 4.2025192 -9.6370408 -66.0222303
N 4.7808317 -10.9165378 -64.3629691
H 3.901636 -11.390675 -64.4666077
H 5.4341878 -11.1196392 -63.6117621
N 6.087026 -7.301176 -67.5654737
H 6.97492 -7.7768733 -67.5279906
C 5.5686416 -6.8960675 -68.8824775
H 5.3268371 -5.8354456 -68.847767
C 4.2717675 -7.636028 -69.2757134
O 3.3960169 -7.0361793 -69.9063768
C 6.6966829 -7.1057223 -69.9118019
H 7.5465829 -6.4850685 -69.622889
H 7.0144693 -8.1489414 -69.8602459
C 6.3428838 -6.7968035 -71.3816833
H 5.5274226 -7.4439008 -71.7033055
C 5.9359276 -5.3387014 -71.5982807

H 6.7312629 -4.6712647 -71.263949
H 5.0185384 -5.1252485 -71.0539862
H 5.7487945 -5.1681951 -72.6580208
C 7.5553389 -7.0841982 -72.2684394
H 8.3866897 -6.4341584 -71.9947723
H 7.2949337 -6.9136733 -73.313153
H 7.8578121 -8.1251123 -72.1530906
N 4.1334798 -8.8979656 -68.8636108
H 4.8250579 -9.2749777 -68.2335885
C 3.0282568 -9.7990911 -69.2066128
H 2.994302 -9.8630138 -70.2915907
C 1.6602333 -9.3119172 -68.6927965
O 1.5945623 -8.516802 -67.7502102
C 3.3072741 -11.2092038 -68.6610331
H 2.5364612 -11.8929918 -69.0166306
H 3.2739869 -11.2045757 -67.5732222
O 4.5717364 -11.6737482 -69.1032695
H 4.7435367 -12.5260357 -68.6915725
N 0.5433211 -9.7783737 -69.2905212
C -0.7985269 -9.4881579 -68.7920022
H -0.974344 -8.4156383 -68.878059
C -0.9754439 -9.9212677 -67.3331253
O -0.527133 -10.9988821 -66.9390965
C -1.7659872 -10.2366409 -69.7171588
H -1.976375 -11.2318292 -69.3196156
H -2.693753 -9.6827823 -69.8650191
C 0.4554123 -10.5666366 -70.5124142
H 1.1583618 -10.2258453 -71.2721388
H 0.6242898 -11.6193873 -70.280253
C -0.973233 -10.3730318 -71.0152853
H -1.0393598 -9.4455678 -71.586019
H -1.3115491 -11.2200922 -71.6130074
N -1.6717958 -9.0991233 -66.547554
H -1.9774122 -8.2113845 -66.9104634
C -1.9426282 -9.3712957 -65.1363939
H -0.9869589 -9.4906116 -64.6280691
C -2.7599268 -10.6578647 -64.9656591
O -3.8400503 -10.7898855 -65.5414905
C -2.701309 -8.1901542 -64.5041285
H -2.7718945 -8.3380187 -63.4261289
H -3.7101012 -8.1606276 -64.9151862
O -2.0772418 -6.945159 -64.7763668
H -2.4140318 -6.2669389 -64.1668781
N -2.292544 -11.5662814 -64.1043462
H -1.3627723 -11.44079 -63.7365719
C -3.0207712 -12.7921241 -63.7457168

H -3.2244875 -13.323451 -64.6781217
C -4.3966937 -12.4684447 -63.1343671
O -4.6230048 -11.3825377 -62.579092
C -2.1096583 -13.72106 -62.9038127
H -1.910148 -14.6234283 -63.4834728
H -1.1418491 -13.2284864 -62.8004951
C -2.5148567 -14.1252411 -61.4840284
H -2.7946617 -13.2267165 -60.9280213
H -1.6416138 -14.553518 -60.9864657
C -3.6505368 -15.1492933 -61.4302847
O -4.2452488 -15.22482 -60.332582
O -4.0275463 -15.7087837 -62.4791995
N -5.3275999 -13.4242844 -63.2208885
H -5.0081732 -14.3620026 -63.4746751
C -6.7373853 -13.2558538 -62.8708644
H -7.1275333 -12.4809128 -63.5271843
C -6.946757 -12.7679238 -61.4410492
O -7.9117529 -12.0322895 -61.2615722
C -7.4812086 -14.5788163 -63.1367407
H -7.2969487 -14.8900304 -64.1668939
H -7.0568127 -15.3459934 -62.4846586
C -8.9995942 -14.5392484 -62.9048429
H -9.3885239 -15.5543231 -62.9959171
H -9.1947796 -14.2219061 -61.8805407
C -9.7553918 -13.6445641 -63.9063237
H -10.1326223 -14.2766743 -64.7127237
H -9.0952164 -12.9031723 -64.3565096
C -10.9343519 -12.935928 -63.2296966
H -11.4774207 -13.6713244 -62.6279936
H -11.6147183 -12.563259 -64.0023007
N -10.4659423 -11.8111498 -62.3804404
H -9.7675482 -12.1340372 -61.710333
H -11.2343898 -11.3862407 -61.8810379
H -10.0067443 -11.1094506 -62.9439105
N -6.0816248 -13.0886981 -60.4718269
H -5.3969747 -13.8110332 -60.6825751
C -6.0968226 -12.5397581 -59.1017552
H -7.1172031 -12.2357542 -58.8791371
C -5.2212816 -11.2922417 -58.8815129
O -5.4720267 -10.5585664 -57.9262851
C -5.7549487 -13.6167123 -58.0640669
H -6.3952429 -14.4869167 -58.2181221
H -5.9482444 -13.2182487 -57.0671658
O -4.3992676 -14.0098058 -58.1432093
H -4.343102 -14.5866227 -58.960205
N -4.2784466 -10.9636136 -59.7796263

H -4.191226 -11.5356199 -60.6116894
C -3.5645761 -9.675878 -59.7459851
H -3.2982635 -9.4612025 -58.710529
C -4.4764878 -8.5391038 -60.2170946
O -4.6424323 -7.5619692 -59.492832
C -2.2637066 -9.7208699 -60.5782299
H -1.5860132 -10.4576694 -60.1459091
H -2.5060962 -10.0411425 -61.5913691
C -1.54592 -8.348949 -60.6594275
H -2.2252017 -7.6004366 -61.0640231
C -1.049715 -7.869789 -59.2926931
H -0.3925492 -8.6162577 -58.8481829
H -1.895953 -7.6778135 -58.6345231
H -0.5004847 -6.9352805 -59.4146506
C -0.3461376 -8.4080241 -61.5969519
H 0.3865714 -9.1103831 -61.226032
H 0.1108929 -7.4212003 -61.6794127
H -0.6523785 -8.7230191 -62.5882788
N -5.1169134 -8.689943 -61.3849318
H -4.9457027 -9.5459942 -61.90868
C -6.0412299 -7.6860189 -61.9416269
H -5.4551489 -6.7857532 -62.1437637
C -7.1240799 -7.231914 -60.9375162
O -7.2297424 -6.0346225 -60.7273625
C -6.5934143 -8.1928127 -63.2907024
H -5.7661369 -8.2380254 -63.9984981
H -6.9541082 -9.2147834 -63.16253
C -7.7329938 -7.3712305 -63.9310273
H -7.8971692 -7.7500898 -64.9408023
H -8.6442331 -7.5659483 -63.3636415
C -7.5575872 -5.8442729 -64.0094301
H -7.4980538 -5.4352241 -63.0077227
H -8.4615509 -5.4163366 -64.4482493
C -6.3614685 -5.3390559 -64.8205827
H -6.662531 -5.2804452 -65.8695286
H -5.5195257 -6.0309988 -64.7300999
N -5.9571078 -4.0034871 -64.3263948
H -6.7679802 -3.395136 -64.17414
H -5.2630821 -3.5587778 -64.9038722
H -5.4837897 -4.0957187 -63.4295262
N -7.871645 -8.0931999 -60.2239082
C -8.873936 -7.6616363 -59.2573345
H -9.5234761 -6.9139405 -59.7159941
C -8.2649088 -7.0320422 -57.9992117
O -8.9337415 -6.198365 -57.3998078
C -9.7099499 -8.8997193 -58.9176722

H -10.0869565 -8.8805224 -57.8938592
H -10.5345133 -8.9914458 -59.6264198
C -7.9351771 -9.5147877 -60.3439147
H -8.4774898 -9.7379089 -61.262144
H -6.9490279 -9.9651637 -60.3563997
C -8.720291 -10.0246495 -59.1344312
H -9.2281338 -10.9685741 -59.3297095
H -8.0659944 -10.10908 -58.2666461
N -7.021786 -7.3606245 -57.6043131
H -6.4998668 -8.0322734 -58.1535313
C -6.3056253 -6.6353359 -56.5348897
H -6.9728801 -6.5111878 -55.6824392
C -5.9322881 -5.2270172 -57.0036012
O -6.1602659 -4.2700824 -56.2681912
C -5.0687729 -7.4390979 -56.0860549
H -5.3895788 -8.424192 -55.7418211
H -4.4305065 -7.5948128 -56.9556649
C -4.1992388 -6.7757217 -54.993123
H -3.2115868 -7.2341931 -55.0481208
H -4.0668317 -5.7140503 -55.2046993
C -4.7169933 -6.9550987 -53.5545555
H -4.8133604 -8.0239603 -53.351868
H -5.6999526 -6.4874112 -53.4551922
C -3.7314371 -6.3211879 -52.5553124
H -3.9467867 -5.2535484 -52.4822222
H -2.7197203 -6.4281609 -52.9491867
N -3.7880118 -6.9492504 -51.2117734
H -4.7008678 -6.8646728 -50.7950431
H -3.1294465 -6.5095671 -50.5629086
H -3.5314121 -7.9249485 -51.2652042
N -5.4101965 -5.0928218 -58.2204721
H -5.2513331 -5.9347831 -58.7634563
C -5.1139113 -3.8079675 -58.8626221
H -4.4172266 -3.2441876 -58.2414818
C -6.3827766 -2.9486339 -58.9977917
O -6.424878 -1.8461333 -58.4601641
C -4.4306352 -4.0925634 -60.2147685
H -3.4889227 -4.612916 -60.0251308
H -5.0681263 -4.750292 -60.8032861
C -4.1402352 -2.8329003 -61.0402102
H -3.5223312 -2.1630568 -60.4391233
H -5.0881717 -2.3339607 -61.2560169
C -3.4537445 -3.1310734 -62.3813936
O -3.6879035 -4.2298107 -62.9487496
O -2.7668995 -2.224337 -62.9038172
N -7.4664462 -3.5002571 -59.5499567

H -7.3734285 -4.4240226 -59.9575726
C -8.7116426 -2.7639545 -59.7964255
H -8.4353106 -1.8095062 -60.2480485
C -9.4638687 -2.4089424 -58.5074308
O -9.9528278 -1.288221 -58.3755535
C -9.6118555 -3.5466591 -60.7821039
H -9.0607949 -4.3693492 -61.2330581
H -10.4668038 -3.9705644 -60.2532929
C -10.1111674 -2.6392604 -61.9230753
H -10.7214885 -1.8416841 -61.4932013
H -10.7558622 -3.2309231 -62.5768496
C -8.9667387 -2.0313695 -62.7636765
O -8.2371866 -2.7994259 -63.4391515
O -8.8066446 -0.7847794 -62.7441526
N -9.47042 -3.2962327 -57.4999447
H -9.0417097 -4.20374 -57.6524768
C -10.1010431 -3.0170332 -56.1973756
H -11.0288266 -2.4764864 -56.3973745
C -9.2769901 -2.0761537 -55.3142795
O -9.8498703 -1.4157787 -54.4554675
C -10.4660358 -4.334596 -55.4944689
H -11.0058283 -4.9560219 -56.2112972
H -9.5566922 -4.8544857 -55.1890845
C -11.3704398 -4.1053174 -54.2696322
H -10.7558442 -3.807233 -53.4188719
H -12.0692675 -3.2953097 -54.4855386
C -12.2007088 -5.3376816 -53.8874421
H -12.8839548 -5.5640901 -54.7082912
H -12.7938671 -5.0865768 -53.0059178
C -11.3301939 -6.5636396 -53.5898349
H -10.6293076 -6.3072624 -52.7892185
H -10.7480368 -6.8020651 -54.4859351
N -12.1634946 -7.726805 -53.1936491
H -12.701381 -7.5095402 -52.3633894
H -11.5843428 -8.534113 -52.9993255
H -12.8073944 -7.9650457 -53.9371857
N -7.9716141 -1.9444161 -55.5661454
H -7.5758423 -2.5040646 -56.3110106
C -7.1488087 -0.8533673 -55.0040867
H -7.6222074 -0.5067725 -54.0856447
C -7.1064176 0.3871534 -55.9041949
O -6.4532318 1.363562 -55.5514579
C -5.7327836 -1.3132419 -54.6059369
H -5.1704231 -0.4493004 -54.2512706
C -5.7686133 -2.3392788 -53.4714731
H -6.3200593 -3.2274988 -53.7777969

H -6.2540956 -1.8980821 -52.6008782
H -4.7497511 -2.6174525 -53.2046511
O -5.0121386 -1.9015539 -55.6648057
H -5.5081724 -2.690785 -55.9458878
N -7.8166632 0.3654646 -57.0417343
H -8.3462446 -0.4726061 -57.2427193
C -7.8502309 1.3899778 -58.0923619
H -8.3461036 0.901136 -58.9292334
C -6.4594769 1.7892235 -58.633805
O -6.3001095 2.8344891 -59.2640218
C -8.7526115 2.5574542 -57.6322694
H -9.6187161 2.1305136 -57.1235023
H -8.1999693 3.1478449 -56.8997247
C -9.2803135 3.5032468 -58.7320415
H -8.4579137 4.0436594 -59.1952806
C -10.0659324 2.771923 -59.8264709
H -10.8596408 2.1741863 -59.3761475
H -9.4021933 2.1159166 -60.3858728
H -10.5024011 3.4948423 -60.5147045
C -10.2132689 4.5376623 -58.0986059
H -11.0746174 4.0426197 -57.649767
H -10.5543428 5.2422816 -58.85601
H -9.6730952 5.0878871 -57.3275553
N -5.4426723 0.9434196 -58.4481881
H -5.6678236 0.0355238 -58.0548346
C -4.0798935 1.185912 -58.9357536
H -3.8150141 2.2211407 -58.7113507
C -4.0051982 1.032849 -60.4518383
O -4.7678273 0.27695 -61.0434649
C -3.0949649 0.2568287 -58.2130025
H -3.5138127 -0.7490093 -58.1422002
H -2.1678002 0.2159683 -58.784929
C -2.795052 0.8141 -56.8097441
H -2.3119699 1.7866437 -56.9208895
H -3.7301621 0.9656837 -56.2690902
C -1.8893985 -0.0773955 -55.9502131
H -2.4545609 -0.9480272 -55.6154601
H -1.5950424 0.5033683 -55.0736083
C -0.6439782 -0.5463608 -56.7108241
H -0.3018319 0.268945 -57.3493463
H -0.9215663 -1.3830817 -57.3566641
N 0.4467047 -0.9339892 -55.7916971
H 0.7465728 -0.112789 -55.2498367
H 1.2748543 -1.2105249 -56.2979961
H 0.1611309 -1.6486113 -55.1440047
N -3.0487572 1.7262357 -61.0774219

H -2.4915023 2.3509615 -60.5015827
C -2.7384924 1.6213578 -62.5167704
H -3.2764944 0.767828 -62.9341887
C -1.2710996 1.2723612 -62.7035448
O -0.4066791 2.1504201 -62.6343866
C -3.1835379 2.9095297 -63.2545982
H -3.4195988 3.6894009 -62.5300726
H -2.3764105 3.2816492 -63.8842477
C -4.4157079 2.6815394 -64.1627795
H -4.7723658 3.6569256 -64.4963477
H -4.1117891 2.1108096 -65.0407656
C -5.5713926 1.9356428 -63.4710507
H -5.670715 2.3288555 -62.460506
H -5.3424209 0.8725846 -63.4125411
C -6.9189723 2.0674559 -64.1837241
H -6.9312356 1.427326 -65.0694601
H -7.0421595 3.108668 -64.4916204
N -8.0097774 1.7033435 -63.2510456
H -7.9605382 0.719054 -62.960874
H -8.919042 1.7965361 -63.6756161
H -7.9574248 2.2846703 -62.4272009
N -0.9851324 -0.0152724 -62.8685472
H -1.7507671 -0.6921228 -62.9343551
C 0.3622951 -0.5630426 -62.7563147
H 0.9805693 0.166144 -62.2307181
C 0.9623679 -0.7635467 -64.1569443
O 0.4536773 -1.5034089 -65.0035394
C 0.3662204 -1.8384641 -61.8795023
H -0.0630003 -2.6610733 -62.4548948
C 1.819163 -2.1735927 -61.5191769
H 2.2062048 -1.4454116 -60.8056847
H 2.4410637 -2.152216 -62.4072739
H 1.8767011 -3.1699076 -61.08193
C -0.4305112 -1.6738873 -60.5577608
H 0.0351023 -0.9039866 -59.9410057
H -1.442414 -1.3440835 -60.7786126
C -0.5557201 -2.9669112 -59.7395202
H 0.4082644 -3.2467547 -59.3150915
H -0.9233073 -3.7726035 -60.3764866
H -1.265722 -2.8133331 -58.927048
N 2.0820627 -0.0851478 -64.4029156
H 2.4655832 0.475942 -63.6575331
C 2.9068083 -0.2770199 -65.5962123
H 2.3018658 -0.6952979 -66.4020926
C 4.0396154 -1.2643317 -65.292497
O 4.4307098 -1.4269569 -64.1332412

C 3.4495725 1.0814523 -66.061202
H 4.0958151 0.9525892 -66.9322466
H 4.0432311 1.5292122 -65.261646
S 2.0709612 2.1846743 -66.4876026
H 1.3003227 1.8921115 -65.4342484
N 4.6448883 -1.8495044 -66.3268338
H 4.3466771 -1.6176306 -67.261478
C 5.9932657 -2.3856146 -66.1595621
H 6.0491473 -2.8706147 -65.1886549
C 7.0088041 -1.2347704 -66.1249314
O 6.737833 -0.132612 -66.6086652
C 6.3073177 -3.4524623 -67.2163512
H 5.4957053 -4.1766962 -67.2517385
H 7.2191217 -3.9815768 -66.9375472
C 6.5230252 -2.8364934 -68.5773457
O 7.5678347 -2.280934 -68.8635157
N 5.5086591 -2.8222407 -69.403895
H 5.6807276 -2.3748843 -70.3032807
H 4.6772054 -3.3460722 -69.2191437
N 8.1801732 -1.5131329 -65.5618081
H 8.3470506 -2.4508453 -65.2320084
C 9.2452561 -0.5331003 -65.3748175
H 8.8589516 0.286887 -64.7647094
C 9.6957767 0.098359 -66.7072817
O 9.7789872 1.3222783 -66.8014925
C 10.3873618 -1.2029495 -64.5780293
H 11.2574185 -0.549599 -64.5671677
H 10.0590078 -1.2929866 -63.5414857
C 10.8177317 -2.5865022 -65.0490904
C 11.9181375 -2.7365552 -65.9169673
H 12.4819757 -1.8755661 -66.2390297
C 12.3053206 -4.0136478 -66.363435
H 13.1452796 -4.1187607 -67.0335377
C 11.597087 -5.1547648 -65.9319127
O 11.9592972 -6.3881262 -66.3699191
H 12.7274721 -6.3595775 -66.9421564
C 10.5129368 -5.0118722 -65.040923
H 9.9908157 -5.8930459 -64.6965091
C 10.1282528 -3.7322316 -64.5973171
H 9.308215 -3.6379075 -63.8983316
N 9.914116 -0.6953223 -67.7660937
H 9.7185 -1.6813804 -67.6780474
C 10.4258322 -0.1969978 -69.0526211
H 11.3531195 0.3334907 -68.8684677
C 9.4744436 0.8147866 -69.7068052
O 9.9050969 1.903903 -70.0918449

C 10.7555701 -1.3866897 -69.9704476
H 11.4799712 -2.0264374 -69.4643361
H 9.8468928 -1.968167 -70.1290859
C 11.319076 -0.9912917 -71.3499645
H 10.5886703 -0.3825456 -71.8825983
C 12.633967 -0.2142643 -71.2488822
H 13.3625122 -0.779567 -70.6682336
H 12.462991 0.7532524 -70.7772734
H 13.0319279 -0.0350601 -72.2476385
C 11.5735498 -2.2547509 -72.1719436
H 12.3160219 -2.8816896 -71.6783682
H 11.9344282 -1.9816748 -73.1636924
H 10.6437644 -2.8131246 -72.2827093
N 8.1721243 0.5219023 -69.7300654
H 7.8781855 -0.3931025 -69.4008782
C 7.1627271 1.4694778 -70.216607
H 7.465206 1.8258281 -71.2019721
C 7.0485684 2.7218684 -69.344085
O 6.7977963 3.7974389 -69.8845881
C 5.8037654 0.7721156 -70.3591403
H 5.0142317 1.5249066 -70.3314356
H 5.6424419 0.0910384 -69.5229777
C 5.6722935 0.0300481 -71.6647418
N 5.9532087 -1.3209948 -71.8725283
C 5.7499517 -1.545272 -73.178947
H 5.897551 -2.4993019 -73.6695688
N 5.3500598 -0.4176791 -73.790252
H 5.1471925 -0.3273254 -74.7758818
C 5.2980715 0.5887433 -72.851667
H 5.0341347 1.6258243 -73.0140616
N 7.3164396 2.6332608 -68.0360767
H 7.5599259 1.7314697 -67.6420576
C 7.2614763 3.7968527 -67.1509479
H 6.3620564 4.3393293 -67.4291629
C 8.4261241 4.7738874 -67.3880553
O 8.1695259 5.9696373 -67.5199989
C 7.0948289 3.3363918 -65.6891448
H 6.3288467 2.5601209 -65.6683109
H 8.0271337 2.8928056 -65.3390609
C 6.6681347 4.4549309 -64.7117129
H 7.5189438 5.109165 -64.5258907
C 5.4997377 5.3156355 -65.2074584
H 4.6757884 4.679795 -65.5306189
H 5.8301918 5.9439793 -66.0348046
H 5.1677863 5.9791257 -64.4099764
C 6.2145039 3.8387536 -63.3851094

H 5.3016056 3.2618588 -63.5260214
H 6.0445249 4.6237662 -62.6487135
H 6.9882306 3.1722436 -63.0041928
N 9.6480168 4.2850584 -67.6533181
H 9.7997559 3.2846604 -67.5630426
C 10.7262591 5.1507473 -68.1801541
H 10.8557976 5.9933419 -67.499146
C 10.3952471 5.6900104 -69.5631661
O 10.7418516 6.824877 -69.8713061
C 12.0605472 4.4240518 -68.3016877
H 12.1166467 3.8610945 -69.2350101
H 12.121121 3.7293563 -67.4803064
O 13.1491082 5.3247477 -68.1948499
H 13.269569 5.8510611 -69.0056855
N 9.6793356 4.9112594 -70.3797769
H 9.4739327 3.9631262 -70.084374
C 9.1529553 5.3651188 -71.6612905
H 9.9800387 5.6169965 -72.3253587
H 8.5721268 4.5597261 -72.1096179
C 8.2436502 6.5889815 -71.5206075
O 8.4964877 7.5881455 -72.1829673
N 7.271716 6.5802667 -70.6017955
H 7.1008963 5.7179054 -70.0934582
C 6.3753098 7.7248641 -70.3564864
H 5.913338 8.0175862 -71.2986071
C 7.1064366 8.967594 -69.8279953
O 6.7901523 10.0960769 -70.2162708
C 5.2706065 7.3106618 -69.3736671
H 5.7289851 6.9026424 -68.4718638
H 4.7203521 8.2063148 -69.0832355
C 4.2527546 6.3259864 -69.9274463
C 3.9569052 5.1377987 -69.2312677
H 4.470828 4.90886 -68.3126257
C 2.9868958 4.2463839 -69.7247382
H 2.7656118 3.3367646 -69.1875547
C 2.2932897 4.5473782 -70.9100298
H 1.5351606 3.8746796 -71.2849157
C 2.5749518 5.7375725 -71.6014384
H 2.0297721 5.995317 -72.5016579
C 3.5527959 6.6201892 -71.1146242
H 3.7383022 7.5375435 -71.6559592
N 8.1333138 8.7500029 -69.0098282
H 8.2811018 7.8069615 -68.6678179
C 9.07562 9.7716512 -68.5730746
H 8.5547353 10.5922679 -68.0912188
C 9.8605926 10.3853852 -69.7513712

O 9.7566054 11.5847949 -70.0065046
C 9.9597189 9.1076336 -67.5219712
H 10.8339721 9.7058895 -67.3759088
H 10.2895408 8.1279684 -67.8552867
S 9.0625811 8.9471298 -65.9503501
H 9.8351265 9.8474364 -65.3019252
N 10.4805271 9.5504548 -70.5898632
H 10.5030738 8.5688311 -70.3340113
C 11.2121939 9.9579073 -71.8004392
H 12.0155281 10.6371849 -71.5108146
C 10.3189662 10.6842638 -72.8220458
O 10.6706195 11.7496569 -73.3218699
C 11.8287769 8.7012708 -72.4308687
H 11.0787146 8.1829872 -73.0301747
H 12.1581597 8.0264929 -71.641054
O 12.9535704 9.016732 -73.2287619
H 13.2151123 8.2260534 -73.7115009
N 9.0917743 10.1931924 -73.0341901
H 8.8462209 9.3288767 -72.56349
C 8.0758771 10.7997598 -73.908273
H 8.5230395 10.9933282 -74.8827677
C 7.5601152 12.1572996 -73.4059811
O 6.9709603 12.9057875 -74.1813275
C 6.8980951 9.8177845 -74.0665242
H 6.5963806 9.4797408 -73.0746319
H 6.0469815 10.3468555 -74.4990079
C 7.2010356 8.596359 -74.9559661
H 8.1359891 8.1311667 -74.6495739
C 6.078985 7.5667695 -74.8172612
H 5.1265064 8.0041828 -75.1217947
H 6.002105 7.244648 -73.7790986
H 6.2918725 6.6974123 -75.4373065
C 7.3195826 8.9722824 -76.4354788
H 6.4050387 9.4644876 -76.7684964
H 7.4802548 8.0744735 -77.031481
H 8.1643764 9.6422728 -76.586479
N 7.8083199 12.5056056 -72.1428918
H 8.3267403 11.8644839 -71.5540116
C 7.4700463 13.8201725 -71.5844624
H 6.6249218 14.2248713 -72.140381
C 8.5965015 14.8537852 -71.7283394
O 8.4412966 15.9715581 -71.2569983
C 7.0096966 13.6702722 -70.1392581
H 6.7278152 14.646009 -69.7435907
H 7.8241777 13.2648047 -69.5378654
O 5.8818799 12.8084382 -70.0899695

H 6.1934362 11.8939971 -70.2199382
N 9.6527789 14.5194905 -72.4726668
H 9.6609405 13.583641 -72.8564542
C 10.8450282 15.3307806 -72.7823003
H 11.3215018 14.7681748 -73.5857787
C 11.9223128 15.3449434 -71.6844862
O 12.7208264 16.2703293 -71.5460031
C 10.4885248 16.6983789 -73.4123986
H 10.3052822 17.4216129 -72.6154718
H 9.5630553 16.5886969 -73.9810148
C 11.5483248 17.2610639 -74.3780375
O 12.3958398 16.4764423 -74.8659835
O 11.4371665 18.4657644 -74.7049311
N 11.9834183 14.26542 -70.8998863
H 11.310854 13.5258562 -71.0438426
C 13.0028759 14.0912496 -69.8714545
H 13.306885 15.0728525 -69.5156226
C 14.2829326 13.4339092 -70.3807713
O 14.2769208 12.3465484 -70.963321
C 12.4106606 13.3349922 -68.6923727
H 12.0504773 12.3665639 -69.0383334
H 13.215576 13.1470367 -67.9823596
C 11.3089208 14.0511588 -67.9372688
C 10.4143586 13.2895275 -67.1652494
H 10.4207411 12.2129036 -67.2452246
C 9.622269 13.9020251 -66.183757
H 9.0420531 13.2912381 -65.5123696
C 9.6896914 15.2939169 -66.0117228
O 9.0688287 15.8810816 -64.9659394
H 8.6517885 15.2197967 -64.4135997
C 10.476267 16.0688853 -66.8777947
H 10.5008894 17.1334103 -66.7732363
C 11.2869235 15.4568867 -67.8389113
H 11.9309688 16.084664 -68.4366982
N 15.413457 14.0666489 -70.0648519
H 15.3470006 14.8954175 -69.4865486
C 16.7466287 13.6072225 -70.4479379
H 16.6439033 13.0148765 -71.3578561
C 17.3845581 12.7089696 -69.3861672
O 17.0498046 12.785335 -68.2019845
C 17.6340751 14.8021464 -70.8021027
H 17.0888067 15.4656922 -71.4759625
H 18.5250258 14.4458703 -71.3186039
O 18.0387931 15.5263655 -69.6592734
H 17.3011331 15.5682376 -69.0191956
N 18.3085085 11.8526167 -69.839663

H 18.540374 11.8840546 -70.8182419
C 19.0346307 10.8940314 -69.0057114
H 19.4675616 10.1472293 -69.6713046
C 18.1121144 10.1118048 -68.0476025
O 18.3664568 10.0593789 -66.8463913
C 20.2060694 11.6112569 -68.3167241
H 19.8233584 12.3411857 -67.6012615
H 20.822286 12.1241624 -69.055329
H 20.8190484 10.8836954 -67.7848943
N 17.0082147 9.5547067 -68.5680509
H 16.8205954 9.6340163 -69.5538031
C 16.1258953 8.7216485 -67.7571872
H 16.0455573 9.2076642 -66.7871337
C 16.7726785 7.3640399 -67.4612841
O 17.037384 6.5613184 -68.3551552
C 14.6792575 8.6592675 -68.293561
H 14.2122908 9.6269136 -68.1101076
H 14.1203773 7.931987 -67.704949
C 14.479784 8.3334479 -69.7606206
O 14.1690558 7.2130685 -70.1375173
N 14.5042034 9.3331716 -70.6191546
H 14.1908225 9.1433714 -71.5603483
H 14.5384129 10.2922772 -70.2963576
N 17.0392665 7.1424113 -66.177116
H 16.830673 7.8928146 -65.5251381
C 17.5157217 5.8978433 -65.5954028
H 17.9177789 5.2616605 -66.384763
C 16.3357946 5.207524 -64.9288189
O 15.6159122 5.8225069 -64.1466295
C 18.6212937 6.1737034 -64.5672921
H 18.1711266 6.6156535 -63.6769974
H 19.0702786 5.2249355 -64.2716113
C 19.7093446 7.0913046 -65.0380035
C 19.7931824 8.4044157 -64.7311366
H 19.0720161 8.9404149 -64.124824
N 20.8504143 8.9783652 -65.4059943
H 20.9953366 9.9773354 -65.4322483
C 21.4912532 8.0630289 -66.2111008
C 22.5607112 8.1678796 -67.1126483
H 23.0547657 9.1163029 -67.2628505
C 22.976687 7.0234162 -67.8144749
H 23.7994497 7.0845023 -68.5131482
C 22.3164877 5.7977862 -67.6094385
H 22.6337933 4.9207785 -68.1562422
C 21.2368826 5.7069224 -66.707767
H 20.7248034 4.766601 -66.5741431

C 20.7952953 6.8365602 -65.9837824
N 16.1848563 3.912817 -65.176291
H 16.821799 3.469261 -65.8205527
C 15.0796393 3.1104436 -64.6775838
H 14.54308 3.6333506 -63.8833365
C 15.6247004 1.7985474 -64.1325112
O 16.0577958 0.9402323 -64.9020713
C 14.131694 2.8887033 -65.8487103
H 13.64995 3.8395378 -66.0623428
H 14.7289034 2.6128478 -66.7202805
C 13.0901348 1.8002141 -65.5654381
H 13.4487714 1.0489044 -64.8679001
C 11.8698551 2.4330043 -64.9143906
H 11.3898081 3.1481945 -65.575671
H 12.1699034 2.9421886 -63.9979327
H 11.1508884 1.6662489 -64.6483251
C 12.8433832 1.0456084 -66.8611406
H 12.3756177 1.6885177 -67.5980209
H 12.2143535 0.1981272 -66.6495824
H 13.7858171 0.6635186 -67.2549475
N 15.5022455 1.6123315 -62.8227831
H 15.0176951 2.3212374 -62.2749974
C 15.9819101 0.4156113 -62.1354336
H 16.5767406 -0.1688088 -62.8309248
C 14.7948239 -0.412656 -61.638592
O 14.0877462 0.0314062 -60.7298387
C 16.9228566 0.7713672 -60.9737509
H 16.3487296 1.1324943 -60.119959
C 17.7625409 -0.435787 -60.5611816
H 18.3692173 -0.7740933 -61.4018301
H 17.1180753 -1.2500821 -60.2345302
H 18.4224701 -0.1512396 -59.7425426
O 17.83844 1.7718527 -61.3585353
H 17.3314862 2.5714806 -61.5236629
N 14.5757734 -1.6381667 -62.1522123
C 13.7832369 -2.6243789 -61.4326093
H 12.8078798 -2.2156713 -61.1651937
C 14.5459745 -3.0172483 -60.1610187
O 15.6270337 -3.5983735 -60.2304277
C 13.5987399 -3.7952014 -62.405327
H 13.5382697 -4.7567764 -61.8952256
H 12.6985773 -3.6284725 -62.9974839
C 15.1494966 -2.2202696 -63.3610773
H 14.6833669 -1.7644417 -64.236083
H 16.2312348 -2.0961457 -63.4033807
C 14.823558 -3.7137458 -63.3158808

H 14.6132543 -4.1093829 -64.3096971
H 15.6551597 -4.2543635 -62.8618106
N 13.9662979 -2.746838 -58.9908767
H 13.0661721 -2.2754632 -59.0090904
C 14.5182136 -3.1102436 -57.6789229
H 15.4998097 -3.5591397 -57.8229977
C 13.7014467 -4.2044761 -56.9428997
O 13.6238671 -4.1604091 -55.7146881
C 14.714941 -1.81596 -56.8514598
H 13.7756341 -1.2643591 -56.8431172
H 14.9534945 -2.0737327 -55.819355
C 15.8229594 -0.873091 -57.3541501
H 15.6404761 -0.6146515 -58.3959849
C 15.8120489 0.4127376 -56.5303236
H 16.028931 0.1980959 -55.4826737
H 14.8309305 0.8833728 -56.593059
H 16.5554212 1.1109018 -56.9145674
C 17.2136708 -1.5018124 -57.2217831
H 17.3755269 -1.8389114 -56.1975999
H 17.9750578 -0.7631874 -57.470298
H 17.3151205 -2.3433035 -57.9050428
N 13.0948036 -5.214763 -57.6151661
C 12.2053006 -6.1721577 -56.9514439
H 11.3888186 -5.644905 -56.4617025
C 12.9249789 -7.0386965 -55.9075064
O 12.3195788 -7.4085346 -54.9111168
C 11.6162382 -7.0363963 -58.0682371
H 11.3764525 -8.0460584 -57.7312578
H 10.7270492 -6.5549761 -58.4702904
C 13.2765494 -5.6211828 -59.0043124
H 12.7053484 -4.9577645 -59.6524598
H 14.33053 -5.6213029 -59.2835882
C 12.7120005 -7.0340446 -59.1282169
H 12.3119336 -7.2235629 -60.1252091
H 13.4796364 -7.7662537 -58.8743209
N 14.2193922 -7.3309605 -56.0875591
H 14.6758111 -6.9951267 -56.9187628
C 15.0103271 -8.0990672 -55.112049
H 14.434692 -8.9910839 -54.8655987
C 15.2070223 -7.332927 -53.7964256
O 15.0766997 -7.9116657 -52.717904
C 16.3497863 -8.552893 -55.7413196
H 16.1067111 -9.1238207 -56.6393262
C 17.2457079 -7.3784555 -56.1790683
H 17.6104389 -6.833258 -55.3064419
H 16.7096291 -6.695959 -56.8363059

H 18.1109972 -7.7622999 -56.7213065
C 17.1758962 -9.4774166 -54.8192359
H 18.0864798 -9.7670527 -55.3455807
H 17.4716894 -8.935527 -53.9193684
C 16.4479887 -10.7630839 -54.4053729
H 16.0929498 -11.2919093 -55.2902479
H 15.605773 -10.5327737 -53.7532482
H 17.1389073 -11.4070984 -53.8607548
N 15.3935547 -6.0141343 -53.87863
H 15.4044782 -5.57695 -54.785056
C 15.4989416 -5.1573755 -52.7005961
H 16.1260958 -5.6549509 -51.9609007
C 14.1275042 -4.979588 -52.0493859
O 14.0041547 -5.2410058 -50.8625342
C 16.1529791 -3.8128398 -53.0612849
H 15.5453294 -3.3096024 -53.8136461
H 16.1749842 -3.1849001 -52.1697456
C 17.5888162 -3.9565456 -53.5969701
H 17.9604463 -2.9636573 -53.8583359
H 17.5825816 -4.5500643 -54.513358
C 18.5239075 -4.5941443 -52.560969
O 18.5957494 -5.8428049 -52.4662396
O 19.14147 -3.8633975 -51.7551244
N 13.0718701 -4.7526557 -52.834066
H 13.246795 -4.5814834 -53.8192455
C 11.6674641 -4.7573474 -52.3915481
H 11.5110988 -3.9424476 -51.6838844
C 11.2638044 -6.055375 -51.6705936
O 10.7000723 -5.9916155 -50.5815018
C 10.8225721 -4.4658085 -53.6373247
H 11.2703018 -4.9725609 -54.4847155
H 10.8980451 -3.3996945 -53.8162933
C 9.3400869 -4.8422281 -53.6032018
O 8.5960027 -4.4336681 -52.6834481
O 8.8625046 -5.4577929 -54.5871172
N 11.6917214 -7.2238873 -52.1577427
H 12.1378338 -7.2256039 -53.0701622
C 11.4803921 -8.5084706 -51.4804795
H 10.4192842 -8.5902366 -51.2439924
C 12.2581264 -8.616735 -50.1576347
O 11.7173718 -9.1125472 -49.1729519
C 11.8590517 -9.6523727 -52.4351888
H 12.9441046 -9.6885066 -52.5437256
H 11.4310558 -9.4628766 -53.4176041
C 11.3583046 -11.0199242 -51.9562242
H 11.7482812 -11.2164564 -50.9570363

H 11.7634607 -11.7769659 -52.6281464
S 9.5513028 -11.2041661 -51.9296248
C 9.4280423 -12.9497713 -51.463499
H 8.3802362 -13.2482151 -51.4521366
H 9.8585708 -13.0948903 -50.4720088
H 9.9653893 -13.5614913 -52.1881289
N 13.4992124 -8.1155487 -50.1013757
H 13.8751445 -7.7085546 -50.9531837
C 14.3462607 -8.0992723 -48.8917458
H 14.3261654 -9.0880359 -48.4302966
C 13.8414349 -7.109273 -47.8321094
O 13.8696885 -7.4088854 -46.6438106
C 15.7803139 -7.7771225 -49.3386629
H 16.0728206 -8.4953921 -50.1072518
H 15.78842 -6.7853287 -49.7877521
C 16.8146607 -7.8333564 -48.2019743
H 16.5459291 -7.1366515 -47.4067893
H 16.8280532 -8.8437172 -47.7908348
C 18.2187971 -7.4873246 -48.7193819
H 18.4184365 -8.0829343 -49.6128579
H 18.9536731 -7.7499724 -47.9570584
C 18.3487833 -5.992413 -49.0458139
H 18.4319706 -5.4217815 -48.1165061
H 17.4451803 -5.6561907 -49.5619324
N 19.5031294 -5.7293883 -49.9309239
H 20.3954323 -5.9966135 -49.5549232
H 19.5174901 -4.749167 -50.2346865
H 19.3464276 -6.1609146 -50.848426
N 13.3322727 -5.9630881 -48.2710839
H 13.3916351 -5.7966786 -49.2700395
C 12.6752334 -4.9259791 -47.4652083
H 13.3066394 -4.639705 -46.6204001
C 11.3514425 -5.4659331 -46.907204
O 11.0801258 -5.3692947 -45.708615
C 12.463281 -3.6936637 -48.3766892
H 12.0638674 -4.0566426 -49.3241162
C 11.3939495 -2.735481 -47.8251092
H 11.5699158 -2.5482408 -46.7698325
H 10.4027027 -3.1721763 -47.9439616
H 11.4167651 -1.795219 -48.3641339
C 13.8090055 -3.0011842 -48.7056933
H 14.1943818 -2.5034339 -47.8276267
H 14.5553025 -3.7476582 -48.9678682
C 13.7415405 -1.9940272 -49.8643963
H 13.1388901 -1.128049 -49.5957665
H 13.3172396 -2.4665957 -50.7493734

H 14.7489847 -1.6485579 -50.0979748
N 10.5569209 -6.1251239 -47.7575478
H 10.8058184 -6.1502801 -48.7417588
C 9.3406062 -6.8155937 -47.3499945
H 8.687436 -6.0980664 -46.8515223
C 9.6451199 -7.9320667 -46.3426887
O 8.9789309 -7.9857565 -45.3181334
C 8.6090851 -7.3439192 -48.5900398
H 9.2347282 -8.0646011 -49.1166765
H 8.3743305 -6.5167723 -49.2610325
H 7.6825766 -7.8315087 -48.286617
N 10.6967849 -8.7327052 -46.5459848
H 11.1877227 -8.6812436 -47.4322827
C 11.1410652 -9.7413254 -45.5766499
H 10.3095113 -10.4188421 -45.3765601
C 11.5417736 -9.1181235 -44.2316211
O 11.0027769 -9.5243077 -43.2065835
C 12.2879134 -10.5821685 -46.1574739
H 13.1383181 -9.9410307 -46.3900585
H 11.9539012 -11.054893 -47.0827182
C 12.7241101 -11.6616207 -45.1622198
O 13.5224159 -11.3337599 -44.252894
O 12.2018212 -12.793503 -45.265959
N 12.3829782 -8.0769071 -44.2118859
H 12.8033958 -7.7736289 -45.0842588
C 12.8418208 -7.4756884 -42.9545116
H 13.20788 -8.2795145 -42.315361
C 11.7105569 -6.7843906 -42.179033
O 11.5105007 -7.0762136 -40.995856
C 14.0107534 -6.5158331 -43.2086705
H 14.8199205 -7.0620265 -43.6953675
H 13.6867322 -5.7238326 -43.8863098
C 14.5352889 -5.889822 -41.9505213
C 15.3513898 -6.4920806 -41.0560262
H 15.7532765 -7.4912347 -41.1718495
N 15.5335855 -5.6654725 -39.9629168
H 16.0897329 -5.9158626 -39.160673
C 14.8148192 -4.49376 -40.0872147
C 14.6321954 -3.3866221 -39.242576
H 15.1032791 -3.3500841 -38.2735638
C 13.819259 -2.3236695 -39.676404
H 13.6551516 -1.4597462 -39.0434416
C 13.2231146 -2.3758491 -40.9490175
H 12.61592 -1.5480294 -41.2963996
C 13.413713 -3.4925454 -41.785488
H 12.9690569 -3.4997165 -42.7718436

C 14.1985453 -4.5894945 -41.3712467
N 10.9043925 -5.9527161 -42.8492866
H 11.1134384 -5.7630804 -43.8240366
C 9.7438538 -5.2921988 -42.2212211
H 10.0844229 -4.773692 -41.3238945
C 8.6828352 -6.2978319 -41.7797415
O 8.1475363 -6.1684389 -40.6765001
C 9.0796136 -4.2487583 -43.1304959
H 8.177974 -3.8895772 -42.6371761
C 9.9720763 -3.0467225 -43.4104666
H 10.8669468 -3.3593763 -43.9445606
H 10.2661708 -2.5763767 -42.4714854
H 9.4314004 -2.3171273 -44.0117579
O 8.7181591 -4.8066588 -44.3726664
H 9.5496823 -4.9479732 -44.8577988
N 8.4358032 -7.3581704 -42.5633335
H 8.8963046 -7.4314515 -43.466883
C 7.5723311 -8.4495715 -42.1268535
H 6.6350766 -8.0155664 -41.7795424
C 8.1700975 -9.1517994 -40.9247922
O 7.4758181 -9.174459 -39.9330543
C 7.2448461 -9.4620492 -43.2330206
H 8.1639771 -9.7685181 -43.7320877
H 6.8107539 -10.3548548 -42.779457
C 6.239774 -8.9211213 -44.2597927
H 6.2060887 -9.6067299 -45.1067549
H 6.5649357 -7.9487869 -44.6272582
C 4.8301142 -8.7907822 -43.6962677
O 4.0185217 -9.6936336 -43.7800733
N 4.4373901 -7.6329512 -43.2092787
H 3.4549186 -7.5555601 -42.9669857
H 5.0762198 -6.8618807 -43.140931
N 9.4273277 -9.6006942 -40.9284614
H 9.9686587 -9.4839231 -41.780828
C 10.0214635 -10.412134 -39.8567427
H 9.3084547 -11.1990181 -39.609992
C 10.253828 -9.6601678 -38.5291229
O 10.2250454 -10.2893742 -37.4666527
C 11.2944858 -11.0964365 -40.3945858
H 11.8618845 -11.5134649 -39.5643313
H 11.9244235 -10.3674132 -40.904203
C 10.9694015 -12.2521054 -41.3360884
O 10.0165345 -12.9890257 -41.1245311
N 11.7728694 -12.4937022 -42.343769
H 11.4995019 -13.1733068 -43.0362986
H 12.499172 -11.8467642 -42.6449554

N 10.2929531 -8.3240194 -38.537167
H 10.3624872 -7.8437779 -39.4304161
C 10.2075646 -7.5261957 -37.311539
H 10.9370343 -7.9083139 -36.5955343
C 8.8173012 -7.6078404 -36.6366541
O 8.7224682 -7.6976754 -35.4119872
C 10.5911494 -6.0827475 -37.6571958
H 9.8848154 -5.6694188 -38.3793169
H 11.5923603 -6.0574983 -38.0909011
H 10.5793001 -5.471428 -36.7549126
N 7.7281362 -7.648853 -37.4093658
H 7.8464977 -7.6620426 -38.4175533
C 6.3615845 -7.7065981 -36.8837774
H 6.2525433 -6.8732437 -36.1891368
C 5.9991926 -8.9928891 -36.0905116
O 5.3798625 -8.8602907 -35.0312186
C 5.3875801 -7.4605357 -38.0417105
H 5.317507 -8.3466364 -38.6717451
H 5.7360643 -6.6360806 -38.6607618
H 4.3964641 -7.2320804 -37.651936
N 6.3537059 -10.227742 -36.5201682
C 6.0921003 -11.4546801 -35.7992746
H 5.0595046 -11.4728627 -35.4552056
C 7.0141917 -11.5868196 -34.5825291
O 6.5442037 -12.022288 -33.5387212
C 6.2944393 -12.5878507 -36.8162142
H 6.5796654 -13.5239315 -36.3340225
H 5.3790469 -12.7278175 -37.3944718
C 6.9710996 -10.6108314 -37.7595143
H 6.2311127 -10.4906961 -38.55036
H 7.8621115 -10.0271148 -37.9027458
C 7.3852351 -12.0701274 -37.7139111
H 7.3632158 -12.5333561 -38.7012058
H 8.3603036 -12.1870356 -37.2408137
N 8.2600128 -11.0993172 -34.6473682
H 8.5974122 -10.7351963 -35.529587
C 9.1638845 -11.0324667 -33.493428
H 9.2559856 -12.0264496 -33.0531509
C 8.6246533 -10.1030979 -32.3918366
O 8.5898426 -10.4804776 -31.2234502
C 10.5463395 -10.5906214 -33.9929249
H 10.9400176 -11.3588206 -34.6604347
H 10.4349591 -9.6795107 -34.5821898
C 11.5645544 -10.3336599 -32.8982995
C 12.1676299 -11.4139774 -32.226674
H 11.9007571 -12.4282594 -32.4853161

C 13.115578 -11.1764532 -31.2147571
H 13.5750579 -12.007349 -30.6985902
C 13.464218 -9.8578058 -30.873536
H 14.194 -9.6750549 -30.0974929
C 12.8624226 -8.7769348 -31.541472
H 13.1324783 -7.7636193 -31.281157
C 11.9107974 -9.0133361 -32.5496745
H 11.4482941 -8.1777601 -33.0568941
N 8.0603085 -8.9495652 -32.7631566
H 8.1434311 -8.665565 -33.7337643
C 7.4168677 -8.0211198 -31.8271501
H 7.9214617 -8.1082216 -30.8649975
C 5.9315139 -8.326438 -31.5456683
O 5.2535744 -7.4801421 -30.961817
C 7.5941536 -6.574922 -32.324633
H 7.124499 -6.4941971 -33.3064549
H 7.0538484 -5.9068789 -31.6550215
C 9.0136829 -6.0386909 -32.3987931
C 9.4463363 -5.359517 -33.5561945
H 8.7912065 -5.2787379 -34.4129759
C 10.7433545 -4.8150995 -33.6203321
H 11.075635 -4.3075551 -34.5142454
C 11.6148391 -4.9453172 -32.5180674
O 12.8717441 -4.435554 -32.5709646
H 13.0345853 -3.9454995 -33.3800472
C 11.174962 -5.6029107 -31.3511048
H 11.8453005 -5.6829437 -30.507972
C 9.8769556 -6.144496 -31.2893049
H 9.5563376 -6.6474406 -30.3872537
N 5.3871766 -9.481238 -31.956426
H 6.0033601 -10.1538206 -32.4006634
C 3.9274016 -9.6951489 -32.0875758
H 3.5788743 -9.0167038 -32.8656313
C 3.101727 -9.3795924 -30.8315989
O 1.9946272 -8.8568 -30.9597617
C 3.6184426 -11.1199467 -32.5819641
H 4.1373467 -11.2775613 -33.5237867
C 4.0262775 -12.229422 -31.6076246
H 3.4924745 -12.148523 -30.6645446
H 5.0959923 -12.1726016 -31.405786
H 3.820187 -13.1996915 -32.0601955
O 2.2341245 -11.2757929 -32.8256162
H 2.1031661 -11.0808149 -33.7675107
N 3.6312656 -9.6287895 -29.6257628
H 4.5649006 -10.0097299 -29.5872965
C 2.9405287 -9.3177676 -28.3687039

H 1.9221669 -9.6969241 -28.445873
C 2.8276563 -7.8017421 -28.1507249
O 1.7167741 -7.2833488 -28.1555535
C 3.6362754 -10.0368499 -27.1973246
H 3.6353806 -11.1098669 -27.3948457
H 4.6745595 -9.7054275 -27.1474315
C 2.978396 -9.7839054 -25.8233671
H 2.9966347 -8.7178673 -25.6005677
C 1.5310448 -10.2812004 -25.7697653
H 1.4817257 -11.3377576 -26.0293787
H 0.9096754 -9.6938931 -26.4446131
H 1.1425322 -10.1385847 -24.7604909
C 3.7720642 -10.514862 -24.7416685
H 3.7563083 -11.5903824 -24.9153329
H 3.3368301 -10.2987848 -23.7649057
H 4.8026104 -10.1599394 -24.7388168
N 3.9616382 -7.0915666 -28.1298216
H 4.8305866 -7.5964343 -28.1841824
C 4.0327294 -5.6286734 -27.9990707
H 3.5475118 -5.3373421 -27.066531
C 3.3223149 -4.9052738 -29.1588863
O 2.7887892 -3.8112358 -29.0000078
C 5.522688 -5.2420034 -27.9073454
H 5.9417744 -5.7125461 -27.0159418
H 6.0387796 -5.6508386 -28.77724
C 5.8148372 -3.7290084 -27.839953
H 5.4302001 -3.2507583 -28.740477
C 5.2052008 -3.0369301 -26.6265279
H 5.5270028 -3.5308572 -25.7079944
H 4.1165442 -3.0902357 -26.6670599
H 5.5002705 -1.9890255 -26.5938106
C 7.3263404 -3.5054407 -27.8182893
H 7.7487717 -3.9367071 -26.9075049
H 7.5460048 -2.4366408 -27.8259105
H 7.7977514 -3.9704279 -28.681937
N 3.3240981 -5.4922525 -30.3565642
H 3.8200164 -6.3736162 -30.4481514
C 2.6217824 -4.9657189 -31.5266407
H 2.8835245 -3.9148142 -31.6417372
C 1.0943839 -5.029999 -31.3514762
O 0.4108227 -4.0473604 -31.6319395
C 3.127867 -5.7370606 -32.7582961
H 4.209855 -5.607543 -32.8206349
H 2.9292394 -6.7975108 -32.6056175
C 2.5111026 -5.3318787 -34.1077532
H 1.4532277 -5.5947615 -34.1197899

C 2.6686143 -3.8435188 -34.4097948
H 3.7203921 -3.5715168 -34.3494023
H 2.0853599 -3.2554452 -33.704076
H 2.2981698 -3.6320755 -35.4139741
C 3.226156 -6.095398 -35.2194507
H 4.2560045 -5.7514367 -35.3113525
H 2.7083162 -5.937219 -36.1650524
H 3.2404669 -7.1588567 -34.9842874
N 0.5617721 -6.1321914 -30.8100106
H 1.1854655 -6.8955181 -30.565335
C -0.8774119 -6.2932057 -30.5294087
H -1.4450707 -5.8133694 -31.3273322
C -1.3387857 -5.5989339 -29.2472027
O -2.4972833 -5.2107496 -29.1799714
C -1.2259879 -7.7857952 -30.5059816
H -0.5395652 -8.3161481 -29.8425807
H -2.2423577 -7.9080194 -30.1304602
C -1.1550235 -8.3653732 -31.9235204
H -0.1595575 -8.2283015 -32.3457716
H -1.8592111 -7.825137 -32.5503861
C -1.517416 -9.8499764 -31.9535904
H -1.7789138 -10.1101266 -32.9786297
H -2.3926915 -10.0247935 -31.3275004
N -0.3815104 -10.6888054 -31.5472774
H 0.4686067 -10.5891001 -32.0922565
C -0.2819976 -11.5284697 -30.5397689
N 0.7987728 -12.2399241 -30.4453776
H 0.9213052 -12.9191248 -29.7195572
H 1.4926744 -12.1243647 -31.1788985
N -1.2140364 -11.691179 -29.6413828
H -2.0524106 -11.1461476 -29.7056909
H -1.0837421 -12.3425586 -28.8909044
N -0.4359914 -5.4137744 -28.291648
H 0.4565919 -5.878876 -28.4106519
C -0.6121594 -4.6637386 -27.0405899
H -1.5484464 -4.9448234 -26.5574101
C -0.6419177 -3.1442925 -27.2699944
O -1.4719593 -2.454789 -26.6849815
C 0.5604857 -5.0831334 -26.1452238
H 0.4619065 -6.1502756 -25.9371324
H 1.4675594 -4.941078 -26.7285774
C 0.7664583 -4.3591921 -24.8161269
H -0.1072872 -4.5150072 -24.1788573
H 0.8875321 -3.2879945 -24.9917974
C 2.035639 -4.9226683 -24.1581527
O 1.8929473 -5.6917077 -23.1831504

O 3.1384206 -4.6239782 -24.6732221
N 0.1917402 -2.6286023 -28.1822229
H 0.9142711 -3.2259607 -28.5641261
C 0.1832659 -1.2150978 -28.5658876
H 0.0037262 -0.6137299 -27.6722889
C -0.9246664 -0.8558517 -29.5768898
O -1.3427934 0.2986789 -29.6289793
C 1.5712314 -0.8568396 -29.111109
H 1.7755088 -1.4407739 -30.0082925
H 2.3275326 -1.0683264 -28.3535512
H 1.5991124 0.2052893 -29.3567835
N -1.3690744 -1.8151607 -30.399589
H -0.9570034 -2.7323259 -30.3107115
C -2.4122034 -1.6197109 -31.4130676
H -2.8401389 -0.6237023 -31.2850044
C -3.5838278 -2.5891446 -31.1989809
O -4.4984085 -2.2893246 -30.4387788
C -1.8158924 -1.6685056 -32.833723
H -1.3145361 -2.624073 -32.987596
H -2.63415 -1.6141187 -33.553035
C -0.8409829 -0.5505884 -33.1512774
C -1.3096435 0.649862 -33.7150379
H -2.3647469 0.7833783 -33.9051926
C 0.531843 -0.7025696 -32.8802269
H 0.8864084 -1.6149005 -32.4260483
C -0.4110316 1.6879453 -34.01348
H -0.7806818 2.6123909 -34.4308109
C 1.4300197 0.3388147 -33.1757978
H 2.4760809 0.2347951 -32.9481857
C 0.9602995 1.5313944 -33.7512922
H 1.6466328 2.3356076 -33.9776088
N -3.6205628 -3.7132496 -31.9236746
H -2.8479066 -3.9034345 -32.5436994
C -4.6046085 -4.7861899 -31.7255437
H -4.6477881 -5.0219769 -30.6606922
C -4.2176418 -6.0541322 -32.4919207
O -3.4466607 -6.006089 -33.4557384
C -6.0082382 -4.3507814 -32.1811528
H -6.3304691 -3.4699273 -31.624698
H -6.7175425 -5.152142 -31.9705056
O -6.0325689 -4.0692292 -33.5679786
H -5.899994 -3.1163384 -33.6518697
N -4.8741413 -7.1737827 -32.163381
H -5.4716506 -7.1487112 -31.3494907
C -4.8665038 -8.4037354 -32.9751168
H -3.8421984 -8.7622554 -33.024553

C -5.296619 -8.1459118 -34.4291164
O -4.6251069 -8.5946963 -35.3565651
C -5.7191938 -9.4992887 -32.2851475
H -5.2617698 -9.6962688 -31.3135578
C -7.1749464 -9.0643969 -32.0189038
H -7.7180113 -8.9252308 -32.9539572
H -7.2157387 -8.1483551 -31.4309694
H -7.6857446 -9.8419113 -31.4478146
C -5.7591062 -10.8389807 -33.0521625
H -6.3486961 -11.5529072 -32.4750014
H -6.2623358 -10.6958331 -34.0075932
C -4.3868889 -11.4732058 -33.3065014
H -3.86465 -11.6184485 -32.3614838
H -3.7929475 -10.8453952 -33.9693974
H -4.5241808 -12.4437534 -33.7840225
N -6.3197801 -7.3081212 -34.6459337
H -6.7867577 -6.9174776 -33.8407924
C -6.8108968 -6.9429317 -35.9822531
H -6.9295796 -7.8697976 -36.5438468
C -5.8010042 -6.0946841 -36.7703672
O -5.7534856 -6.2049544 -37.9993011
C -8.1929232 -6.2678168 -35.828368
H -8.8936588 -6.9935093 -35.4113768
H -8.1035006 -5.4462128 -35.1147961
C -8.795135 -5.6932521 -37.1238768
H -9.7559562 -5.2332397 -36.8894435
H -8.1411857 -4.8942535 -37.4703831
C -9.0162358 -6.7421972 -38.2346595
H -9.9843958 -7.2211919 -38.076221
H -8.2596339 -7.5233973 -38.1830747
C -8.9707821 -6.1540308 -39.6519625
H -9.7832162 -5.4324325 -39.7803459
H -9.1312743 -6.9749907 -40.3575952
N -7.6582374 -5.5172426 -39.9201159
H -7.5238449 -4.6979612 -39.3466649
H -7.5232324 -5.260878 -40.8933727
H -6.8930147 -6.1433565 -39.6825251
N -4.9831557 -5.2914209 -36.0868715
H -5.1114733 -5.2316311 -35.0833861
C -3.8621657 -4.5557308 -36.6755995
H -4.2125805 -3.9476157 -37.5101316
H -3.4303927 -3.8987074 -35.9202956
C -2.7652385 -5.4947951 -37.177779
O -2.4814048 -5.5085537 -38.373279
N -2.2677174 -6.3806718 -36.3070245
H -2.5945535 -6.342489 -35.3479413

C -1.2607822 -7.3958332 -36.6512525
H -0.3445124 -6.8925585 -36.961776
C -1.6939054 -8.2984128 -37.8176158
O -0.9311845 -8.4717807 -38.7673839
C -0.9587692 -8.2239316 -35.3945171
H -0.5126998 -7.5591821 -34.6545479
H -1.9003645 -8.5918536 -34.9852958
C -0.0205586 -9.403772 -35.5957312
C -0.476939 -10.7237244 -35.4080804
H -1.509788 -10.9114615 -35.1526382
C 0.4018223 -11.8087914 -35.5828742
H 0.0411055 -12.8215519 -35.4605813
C 1.7396798 -11.5814567 -35.9510346
H 2.4073452 -12.4177367 -36.1126058
C 2.197147 -10.2669613 -36.1448257
H 3.2167878 -10.0866761 -36.4561706
C 1.3158525 -9.1847377 -35.975059
H 1.6594669 -8.1812815 -36.1674815
N -2.9508894 -8.7602365 -37.8162996
H -3.5180262 -8.6013591 -36.9897401
C -3.5164419 -9.5705426 -38.9055008
H -2.8830213 -10.4489155 -39.0412821
C -3.4884375 -8.8191223 -40.2441416
O -3.0854456 -9.4032043 -41.2444796
C -4.9353343 -10.0606912 -38.5229828
H -5.4930771 -9.2156787 -38.1147458
C -5.7199256 -10.6008875 -39.7362459
H -5.1748012 -11.428586 -40.1934969
H -5.8704642 -9.8203191 -40.4808494
H -6.7059161 -10.950001 -39.4339102
C -4.8187313 -11.1576382 -37.4374046
H -4.3660146 -12.0503218 -37.8713733
H -4.1598068 -10.814504 -36.6407699
C -6.1553173 -11.5460725 -36.7923586
H -6.7955629 -12.0580817 -37.5094256
H -6.6616767 -10.6550823 -36.4198857
H -5.9674939 -12.2254155 -35.9610021
N -3.8323925 -7.5248185 -40.2879394
H -4.0656676 -7.0556824 -39.4218744
C -3.7691065 -6.745843 -41.5391746
H -4.3005425 -7.2994218 -42.3139513
C -2.3393446 -6.5519942 -42.0526116
O -2.1145754 -6.6247537 -43.2621331
C -4.4332741 -5.3718712 -41.3703427
H -3.8537605 -4.6109815 -41.8970195
H -4.4733346 -5.1006908 -40.3138927

O -5.7412933 -5.3987764 -41.9212897
H -5.6390313 -5.3843889 -42.8827614
N -1.3861787 -6.3126124 -41.1495095
H -1.6547347 -6.2826085 -40.1714205
C 0.0158965 -6.0425797 -41.481886
H 0.0577829 -5.3112942 -42.2894989
C 0.740751 -7.3012983 -41.9896076
O 1.4825599 -7.2298511 -42.9719905
C 0.6993255 -5.4537121 -40.2332656
H 0.5759614 -6.161702 -39.4119538
H 1.768021 -5.3583632 -40.4270006
C 0.1460188 -4.0790425 -39.7977291
H -0.9410754 -4.0968931 -39.8068985
C 0.5881962 -3.7787531 -38.3651539
H 1.6688825 -3.8436922 -38.2817429
H 0.1358835 -4.4987693 -37.6855946
H 0.2728934 -2.7773283 -38.0815491
C 0.5838749 -2.9467471 -40.7300837
H 1.6679734 -2.8669288 -40.751094
H 0.1714852 -2.0035095 -40.3724266
H 0.2150466 -3.1247907 -41.7383577
N 0.4550266 -8.4611916 -41.3890336
H -0.1379686 -8.4416052 -40.564342
C 0.9646168 -9.7595951 -41.8364496
H 2.0196674 -9.6433663 -42.0892642
C 0.2485841 -10.2596498 -43.1035624
O 0.8987943 -10.7616169 -44.0158858
C 0.8455888 -10.7511466 -40.6638834
H 1.3523783 -10.326376 -39.7955445
H -0.210257 -10.8640917 -40.4110157
C 1.4357562 -12.1477194 -40.9424942
H 0.9147976 -12.6038002 -41.7839374
C 2.9345315 -12.1074372 -41.2461209
H 3.4682867 -11.5756565 -40.4587843
H 3.1048157 -11.6059846 -42.1990644
H 3.3258448 -13.1209115 -41.3293987
C 1.228386 -13.036948 -39.7157139
H 1.7534838 -12.6171902 -38.8578697
H 1.6078131 -14.0384338 -39.9180822
H 0.1647686 -13.1057515 -39.4881301
N -1.0698244 -10.0583188 -43.2209953
H -1.5784126 -9.6705319 -42.4337335
C -1.8288138 -10.4543792 -44.4133578
H -1.6406428 -11.512455 -44.6026739
C -1.3632127 -9.6903915 -45.6534104
O -1.0336879 -10.3147171 -46.6612399

C -3.3368109 -10.2801862 -44.1613091
H -3.5384081 -9.2595103 -43.8397539
H -3.6148275 -10.9493552 -43.3461491
C -4.2354917 -10.6192243 -45.3629775
H -5.2387681 -10.8120393 -44.981554
H -3.8764807 -11.5469091 -45.8110439
S -4.390968 -9.3657168 -46.6781462
C -5.1799099 -8.0079527 -45.7729399
H -5.4383667 -7.2116522 -46.4701612
H -4.4931662 -7.6138861 -45.0242914
H -6.0870682 -8.3705426 -45.2881865
N -1.2836702 -8.3547994 -45.597801
H -1.5351508 -7.8696527 -44.7427603
C -0.9788531 -7.5879528 -46.8043397
H -1.457325 -8.1034868 -47.6360249
C 0.521404 -7.5756896 -47.1451478
O 0.8637811 -7.5102657 -48.3264741
C -1.6430715 -6.2032923 -46.748782
H -1.1355764 -5.5871202 -46.0089988
H -2.6802938 -6.3144597 -46.4385527
C -1.6394516 -5.5151375 -48.1094585
N -0.9709235 -4.389742 -48.2157069
H -0.4685428 -4.0199304 -47.4270163
H -0.9501478 -3.9346266 -49.1103779
O -2.2486604 -5.9590541 -49.0785727
N 1.416904 -7.7516498 -46.1678263
H 1.0980991 -7.8154289 -45.2078644
C 2.8375119 -7.9866019 -46.4363406
H 3.1969886 -7.2710589 -47.1758242
H 3.3911814 -7.8211393 -45.5225839
C 3.1279278 -9.4043741 -46.945777
O 3.8298141 -9.5493483 -47.9453507
N 2.447086 -10.4225332 -46.4101552
H 1.9315207 -10.2624063 -45.5490469
C 2.4854058 -11.7979805 -46.9139969
H 3.5152832 -12.1562071 -46.9087821
H 1.8931677 -12.4278665 -46.2504679
C 1.9239234 -11.9295401 -48.3328251
O 2.5932635 -12.4826701 -49.2022689
N 0.7929469 -11.2777152 -48.628854
H 0.267872 -10.8632915 -47.8641561
C 0.2683398 -11.1228987 -49.9938683
H 0.0573556 -12.1067264 -50.4128921
C 1.2826057 -10.4351154 -50.919821
O 1.4660857 -10.8427349 -52.0678355
C -1.03995 -10.3126457 -49.9511539

H -1.8060414 -10.9034882 -49.4476874
H -0.8743164 -9.4136616 -49.3588149
C -1.5543657 -9.8836418 -51.2950987
C -1.1187229 -8.7991881 -51.9758444
H -0.3563124 -8.1147227 -51.6133052
N -1.6791194 -8.7882528 -53.2363242
H -1.3736528 -8.1625098 -53.9676252
C -2.5438482 -9.8457334 -53.4186151
C -3.3411695 -10.2526051 -54.4992947
H -3.3476281 -9.6931163 -55.422582
C -4.1539985 -11.3894695 -54.358604
H -4.801853 -11.6994674 -55.1695271
C -4.1314936 -12.1172815 -53.1550939
H -4.7589197 -12.9925633 -53.044334
C -3.3101688 -11.7105559 -52.0842815
H -3.3046579 -12.2804389 -51.1652787
C -2.499787 -10.5592805 -52.1831049
N 1.96428 -9.3993704 -50.4253499
H 1.7809878 -9.1156849 -49.4709382
C 2.9636726 -8.654149 -51.2011413
H 2.5137098 -8.3680451 -52.1510126
C 4.1751778 -9.5252683 -51.5380323
O 4.584636 -9.5468988 -52.6991712
C 3.3562456 -7.3613529 -50.4714756
H 3.7076416 -7.5770001 -49.4645344
C 4.4220107 -6.5580398 -51.2039862
H 4.0784879 -6.3762013 -52.2179027
H 5.3645424 -7.1059384 -51.2128172
H 4.574357 -5.6062331 -50.6915057
O 2.2203371 -6.5288334 -50.4111686
H 1.6945377 -6.8265429 -49.6442254
N 4.6517677 -10.3483214 -50.5991615
H 4.2697926 -10.2894075 -49.6593257
C 5.6772822 -11.3580867 -50.8568759
H 6.5451977 -10.8700353 -51.3041686
C 5.1923812 -12.4341222 -51.8490049
O 5.8968642 -12.7354913 -52.8110289
C 6.1067911 -11.9647842 -49.5145566
H 5.2580115 -12.4435631 -49.0255728
H 6.499734 -11.1841373 -48.8617151
H 6.8863492 -12.7084471 -49.6781218
N 3.9534366 -12.9249302 -51.7086818
H 3.4267844 -12.6496469 -50.8836684
C 3.334672 -13.8997941 -52.6291921
H 3.9737587 -14.7791788 -52.6745089
C 3.2311635 -13.3581777 -54.0601941

O 3.501972 -14.0879971 -55.0132972
C 1.9611062 -14.3607064 -52.0920771
H 1.4005589 -13.4924383 -51.7508018
C 1.0987965 -15.094003 -53.1308964
H 1.6582642 -15.9270397 -53.5595551
H 0.8015677 -14.4086898 -53.9226587
H 0.1946828 -15.4764729 -52.6554527
C 2.1541222 -15.3188572 -50.9094302
H 2.6359165 -16.2380737 -51.2437664
H 1.18814 -15.559973 -50.4658729
H 2.7765934 -14.8574841 -50.1444956
N 2.9105554 -12.073782 -54.2549198
H 2.6628376 -11.510579 -53.4444772
C 2.8382215 -11.4848562 -55.603466
H 2.5477263 -12.2731829 -56.294507
C 4.1932573 -11.0523229 -56.1735234
O 4.3050569 -11.0014922 -57.3976465
C 1.7500749 -10.3972881 -55.6684607
H 1.973677 -9.6224873 -54.932472
H 1.7766916 -9.9369756 -56.657886
C 0.3181127 -10.9321047 -55.4242165
H 0.2277581 -11.265099 -54.390261
H -0.3748739 -10.1024204 -55.5677658
C -0.0987512 -12.0894509 -56.3560436
H 0.544746 -12.9544503 -56.17014
H 0.0153415 -11.7777132 -57.39766
C -1.5577073 -12.5187353 -56.1029819
H -2.2269318 -11.6802266 -56.3325773
H -1.6736438 -12.7561101 -55.0366384
N -1.920309 -13.7026939 -56.930816
H -1.8494992 -13.5031153 -57.9228767
H -2.903941 -13.9547134 -56.8042702
H -1.3493329 -14.5134468 -56.71273
N 5.239108 -10.8811623 -55.358782
H 5.0832237 -10.9063656 -54.3572045
C 6.6252368 -10.7490509 -55.8568326
H 6.6136699 -10.1725473 -56.7835268
C 7.23075 -12.111499 -56.2120809
O 7.8163342 -12.237301 -57.2830009
C 7.5248285 -9.9796415 -54.8686851
H 7.4639628 -10.4501565 -53.8877875
C 8.9905939 -9.944826 -55.3227672
H 9.0568977 -9.5997377 -56.3555774
H 9.4372889 -10.9357012 -55.2428839
H 9.5579733 -9.2635562 -54.6896495
C 7.0793244 -8.5186274 -54.7449023

H 7.2214826 -8.0117064 -55.6995769
H 7.6884329 -8.0243809 -53.9867521
H 6.0354079 -8.4688468 -54.4529373
N 7.0278598 -13.1595392 -55.4024952
H 6.5409429 -13.011564 -54.5230944
C 7.6271667 -14.4858677 -55.6559459
H 8.6634613 -14.3031979 -55.9438593
C 6.9906641 -15.2279986 -56.8419336
O 7.6716931 -15.9879257 -57.5225639
C 7.6575082 -15.3317962 -54.3638388
H 7.9124323 -14.6589608 -53.5431137
C 6.2996944 -15.9707711 -54.0400428
H 6.0816508 -16.7851927 -54.7321286
H 5.5262014 -15.2171777 -54.1221082
H 6.2972443 -16.3543042 -53.0201916
C 8.777694 -16.3911983 -54.4438692
H 8.5331507 -17.1303691 -55.2074872
H 9.7068405 -15.8989223 -54.7355244
C 9.0351373 -17.1189259 -53.1182629
H 8.173149 -17.7267722 -52.8436591
H 9.2387219 -16.3944539 -52.3289923
H 9.8999306 -17.7735446 -53.2299662
N 5.7387061 -14.9039989 -57.1868191
H 5.2127234 -14.3222172 -56.5485866
C 5.1062204 -15.3009733 -58.4534771
H 5.3874356 -16.3318692 -58.6680816
C 5.6030433 -14.4874707 -59.672003
O 5.0849158 -14.6599443 -60.773668
C 3.5735904 -15.2270815 -58.2905951
H 3.3168007 -14.2373792 -57.910768
H 3.1057086 -15.3358337 -59.269651
C 2.9775319 -16.3018904 -57.3585176
H 3.4899355 -16.2918303 -56.3990126
C 1.4957103 -15.9962792 -57.1210999
H 0.9610944 -15.9934025 -58.0710052
H 1.4152946 -15.0272253 -56.6348142
H 1.0717324 -16.755584 -56.4636815
C 3.0815974 -17.7101515 -57.947436
H 2.616133 -17.7444842 -58.9324024
H 2.5847047 -18.4241014 -57.2906702
H 4.1275589 -18.0041337 -58.0324987
N 6.5584693 -13.5695965 -59.4840437
H 6.9635998 -13.4805897 -58.559394
C 7.1419561 -12.7294521 -60.533019
H 8.0300305 -12.2428227 -60.1296168
H 7.4428545 -13.3604889 -61.3694312

C 6.2113295 -11.6372743 -61.0664694
O 6.4668133 -11.1007314 -62.1414297
N 5.0989407 -11.3521821 -60.3807085
H 4.9813281 -11.7997437 -59.4822739
C 4.011173 -10.5016823 -60.8819061
H 4.0372055 -10.488733 -61.9692609
C 4.1458654 -9.0360507 -60.4550618
O 4.0311999 -8.1487806 -61.2999311
C 2.6640571 -11.1114873 -60.4638078
H 1.8926477 -10.351034 -60.4842377
H 2.730934 -11.4831747 -59.4414438
C 2.2535011 -12.2657448 -61.3868724
H 1.4591474 -12.8339579 -60.902344
H 3.1069454 -12.932034 -61.5231857
S 1.6498686 -11.7327541 -63.0139356
C 1.6214996 -13.3280329 -63.8756168
H 1.2174771 -13.1904539 -64.8799789
H 0.9970689 -14.0358844 -63.3303468
H 2.6341481 -13.7267153 -63.9450518
N 4.4403578 -8.7747006 -59.1755103
H 4.5741277 -9.5745046 -58.565276
C 4.7721047 -7.4432695 -58.6461105
H 4.2329189 -6.6929209 -59.2287529
C 6.2628089 -7.1513591 -58.8255713
O 7.1132365 -8.0286716 -58.7190631
C 4.3124856 -7.3225627 -57.1776623
H 3.2218766 -7.3201566 -57.1538076
H 4.664482 -8.1940433 -56.6248092
C 4.8413101 -6.0586761 -56.4669405
H 5.9269408 -6.1201353 -56.3940316
H 4.5835999 -5.1738691 -57.0510368
C 4.2767258 -5.8952971 -55.0518431
H 4.2724669 -6.8599056 -54.5417714
H 3.2469937 -5.5465683 -55.1283597
C 5.0870934 -4.8828455 -54.2236432
H 4.4787623 -4.580427 -53.3658758
H 5.2958632 -3.991815 -54.8235343
N 6.3573003 -5.466491 -53.7188249
H 6.2395348 -6.3113772 -53.1868018
H 6.9070721 -4.8226841 -53.1439003
H 7.0461657 -5.6510471 -54.4521823
N 6.5831059 -5.8796803 -59.0166479
H 5.8235267 -5.2105063 -59.113986
C 7.9375657 -5.3227778 -59.0310328
H 8.6143732 -5.9856498 -58.4939189
C 7.9299697 -3.9753104 -58.3016906

O 6.8849011 -3.3299543 -58.2056804
C 8.3940536 -5.2114097 -60.498702
H 7.5989185 -4.7461053 -61.083704
C 9.6867695 -4.4393521 -60.7534294
H 10.461601 -4.7634189 -60.0624864
H 9.5092652 -3.3712309 -60.637248
H 10.0261984 -4.6212524 -61.7728531
O 8.6164189 -6.5095718 -60.9963761
H 8.1255677 -7.1366062 -60.4489059
N 9.0868513 -3.5097747 -57.8288001
H 9.9133854 -4.0836863 -57.9019445
C 9.2842826 -2.0970461 -57.484942
H 8.3344924 -1.5820776 -57.6283965
C 10.2397767 -1.4588863 -58.488381
O 11.1822076 -2.0878065 -58.9748673
C 9.6723067 -1.8988096 -55.9998239
H 9.4745394 -2.8358148 -55.4851521
C 11.1589555 -1.5434698 -55.8111287
H 11.3819425 -0.5661663 -56.2441685
H 11.7740098 -2.3086833 -56.2769605
H 11.4226248 -1.4985324 -54.7557414
C 8.7636256 -0.8305709 -55.3496209
H 8.9602225 0.1445593 -55.7991688
H 7.7226656 -1.0931424 -55.5430241
C 8.9349449 -0.7232145 -53.8274753
H 9.9006709 -0.2819557 -53.5826193
H 8.8562241 -1.7099889 -53.3708842
H 8.1532925 -0.0869728 -53.4164581
N 9.9517901 -0.2103244 -58.8094482
H 9.1682783 0.231535 -58.3373574
C 10.654951 0.6360141 -59.7608788
H 11.4393476 0.0802286 -60.2761536
C 11.3137825 1.7707113 -58.9834636
O 10.6744127 2.3280577 -58.0948429
C 9.5994166 1.0937026 -60.7929687
H 8.6089785 1.028997 -60.3467226
H 9.6158493 0.3827572 -61.6188321
C 9.6756245 2.4856417 -61.3678588
N 9.6560584 2.7683857 -62.7304314
C 9.6304442 4.1041051 -62.8320208
H 9.6677495 4.647484 -63.7669766
N 9.599696 4.6686446 -61.6145544
H 9.6754936 5.6576488 -61.4159251
C 9.6037052 3.6646771 -60.6808175
H 9.5692624 3.7963986 -59.6106405
N 12.5267095 2.1509732 -59.3842939

H 13.0058659 1.5797279 -60.0714761
C 13.1036285 3.4786336 -59.1419166
H 12.3791542 4.0801703 -58.6065819
C 13.4003518 4.137936 -60.4980748
O 13.9804095 3.4895145 -61.3751488
C 14.3486196 3.3947989 -58.2404563
H 14.0355429 3.0345998 -57.2602812
H 15.0443904 2.6671256 -58.661884
C 15.0998792 4.7290328 -58.0561606
H 15.5972644 4.9762276 -58.9978091
H 15.8861092 4.5829474 -57.3125827
C 14.2028056 5.9034645 -57.6227446
O 14.1451209 6.8963968 -58.3833069
O 13.5576779 5.8054743 -56.5593021
N 13.0453446 5.4126846 -60.6712948
H 12.6425479 5.9066621 -59.8759317
C 13.3632583 6.2062371 -61.8533931
H 14.1139036 5.6662568 -62.4167197
C 13.9496037 7.5757594 -61.5001384
O 13.3110616 8.3851175 -60.8299668
C 12.1357501 6.3519771 -62.752082
H 11.3121412 6.7960327 -62.1946084
H 11.8710934 5.3727228 -63.1411174
H 12.3807769 6.9994993 -63.5956822
N 15.109189 7.8745898 -62.0873356
H 15.4773862 7.2053572 -62.7563538
C 15.8432781 9.1316037 -61.9126432
H 15.2448618 9.8123746 -61.30726
C 16.0561624 9.7692783 -63.2824116
O 16.4188304 9.0839867 -64.2349318
C 17.1881392 8.8969502 -61.188147
H 17.8076121 8.2561194 -61.8169794
C 16.9764192 8.1765845 -59.8366834
H 16.3622953 8.7964619 -59.1838709
H 16.4432474 7.2414061 -60.0042166
C 17.9261826 10.2389898 -60.9959842
H 17.3004281 10.929227 -60.4298202
H 18.1645092 10.6855659 -61.9607414
H 18.8686102 10.0894265 -60.4738383
C 18.2749524 7.8099776 -59.110778
H 18.7708445 8.7035771 -58.7336403
H 18.9412565 7.2714474 -59.7855677
H 18.0370505 7.1684199 -58.2616609
N 15.8801458 11.0823937 -63.3805037
H 15.5331748 11.5872138 -62.5688742
C 15.9623305 11.817571 -64.6442913

H 16.7952455 11.4316431 -65.2351664
C 16.2306405 13.3158319 -64.4223685
O 16.001996 13.8407697 -63.3256233
C 14.6780471 11.5721577 -65.4405948
H 14.5614297 12.3592306 -66.1828294
H 14.8143697 10.6397859 -65.9771394
C 13.3981278 11.4233831 -64.5967226
H 13.2409175 12.3213791 -64.0054631
H 13.4843556 10.5777558 -63.912386
C 12.2010246 11.1445692 -65.486179
O 12.4220538 10.9836834 -66.7062945
O 11.0711677 11.0577901 -64.952999
N 16.7127389 14.0282412 -65.4533237
H 16.8577185 13.566903 -66.349382
C 16.9273486 15.4814323 -65.3898288
H 16.3871383 15.8476574 -64.5193038
C 16.3447654 16.2645858 -66.5817988
O 16.1558116 15.7419953 -67.6835179
C 18.4109936 15.8096423 -65.1287159
H 18.8261429 15.0930979 -64.4212154
H 18.4804463 16.7986531 -64.677849
C 19.2719235 15.817902 -66.3805842
O 19.9812442 14.8726451 -66.670495
N 19.2629312 16.8803734 -67.1552989
H 19.7115882 16.7659892 -68.0471681
H 18.6938739 17.6945154 -66.9492704
N 16.1171737 17.5560555 -66.3461034
H 16.3244087 17.9218972 -65.4216912
C 15.6409335 18.5213459 -67.3311437
H 15.1563779 17.9880668 -68.1515813
C 16.8207639 19.3019001 -67.9050657
O 17.814395 19.5196466 -67.2156087
C 14.6025547 19.470826 -66.7156745
H 15.0457816 19.9951369 -65.8663646
H 13.7532703 18.8943344 -66.3687152
C 14.1049765 20.4776596 -67.7549781
O 14.0497 21.6808959 -67.4357038
O 13.8939207 20.0301459 -68.9036003
N 16.6932574 19.7127438 -69.1587056
H 15.7361606 19.6535177 -69.5221682
C 17.6417525 20.4891035 -69.9664808
H 18.1709276 19.7718774 -70.5775385
C 18.7397635 21.2279062 -69.1911158
O 19.6163392 20.5303848 -68.6231238
O 18.7905354 22.4280729 -69.0989548
C 16.8603575 21.4153103 -70.9236658

H 16.3055296 22.1240006 -70.3041713
H 17.5800612 21.9809911 -71.516076
C 15.8643249 20.7213124 -71.8783228
H 15.1037594 20.1933291 -71.2956912
C 15.1458514 21.7861056 -72.7164382
H 15.8579213 22.3362843 -73.3312233
H 14.6219113 22.4788679 -72.0561909
H 14.4058411 21.3071409 -73.3609419
C 16.5435476 19.7199673 -72.8232848
H 17.329409 20.2094041 -73.398468
H 15.8002973 19.3095617 -73.5106352
H 16.9602655 18.8885803 -72.2557724

Supplementary Data 4. Cartesian coordinates for CrPeNMT homology model with bound SAM.

N -2.1385667 21.7753992 -15.9844882
H -2.8881317 22.3451992 -15.612073
H -2.4187043 21.4412796 -16.898962
H -1.952445 20.9940613 -15.3721405
C -0.8983927 22.6008534 -16.1048954
H -1.0805973 23.3575681 -16.8709468
C 0.33849 21.7897622 -16.5659561
O 1.2553163 22.327476 -17.1874333
C -0.6053818 23.3116885 -14.7771474
H 0.3668547 23.80077 -14.8479421
H -0.5567763 22.5711725 -13.9778747
C -1.639947 24.3835703 -14.4160168
H -1.396285 24.7758699 -13.426708
H -2.6273748 23.9290305 -14.3587376
S -1.7285502 25.7740286 -15.5733039
C -2.9534829 26.7844932 -14.6928753
H -3.1342375 27.7045542 -15.2488035
H -2.5798462 27.0332981 -13.6984196
H -3.8891334 26.2323095 -14.5953778
N 0.3673335 20.4886197 -16.2657559
H -0.4109708 20.1080689 -15.7405706
C 1.3938537 19.5859753 -16.7771625
H 1.3580658 19.6103407 -17.8672226
H 2.3753087 19.9422459 -16.4680576
C 1.2496557 18.1385969 -16.3224611
O 0.7986647 17.8537127 -15.2154758
N 1.7628938 17.2390447 -17.1522057
H 2.1121366 17.5432004 -18.0501647
C 2.0288584 15.8382482 -16.8338954
H 1.9028599 15.7027836 -15.7604402
C 3.4957816 15.5076051 -17.1394012
O 4.119702 16.1812749 -17.9609391
C 0.9990926 14.9223526 -17.5032517
H 1.2572628 13.8928438 -17.2749979
H 0.0423459 15.1358366 -17.026009
C 0.8149397 15.0455348 -19.020348
H 1.6157817 14.5050359 -19.5288645
H 0.8699863 16.0954136 -19.3176114
C -0.5554566 14.4754363 -19.4180847
O -1.297864 15.2053148 -20.1119062
O -0.875195 13.3478418 -18.9747065
N 4.1061184 14.7016468 -16.2632362
H 3.5442127 14.1102083 -15.6715657

C 5.5629684 14.6122266 -16.0983634
H 6.0446756 14.8186955 -17.0600251
C 5.9695982 13.2129132 -15.6736854
O 5.3463653 12.639753 -14.786269
C 6.0594875 15.5900786 -15.0182406
H 5.5966651 15.3285324 -14.0641537
H 7.1389711 15.4636292 -14.915048
C 5.7688522 17.0627619 -15.3210483
H 6.199365 17.3109927 -16.2919241
H 4.6914859 17.2284646 -15.351451
C 6.3694499 17.9615862 -14.2310701
H 7.4516987 17.8220036 -14.192695
H 5.9514537 17.6743696 -13.2638997
C 6.0413128 19.4366002 -14.4861744
H 6.4275873 20.029751 -13.6516003
H 4.9522862 19.5499365 -14.5026758
N 6.6253332 19.9107507 -15.7656037
H 7.6331593 19.818804 -15.7546612
H 6.3843249 20.8792368 -15.933991
H 6.2683797 19.3550584 -16.5356419
N 7.106455 12.7593095 -16.1705568
H 7.4956956 13.2759729 -16.9574346
C 7.5639831 11.383838 -16.027934
H 6.7220861 10.7521143 -15.7410336
C 8.6256294 11.3080201 -14.9245199
O 9.6775993 11.9537076 -14.9983873
C 8.0743326 10.8706348 -17.3888252
H 8.9570019 11.4416081 -17.6791626
H 8.3787829 9.8302572 -17.2664366
C 7.0169986 10.9370419 -18.5172246
H 6.1405201 10.371064 -18.1914962
H 7.409044 10.4393792 -19.4038394
C 6.5974706 12.3719046 -18.896483
O 7.4785217 13.2681533 -18.9084406
O 5.3745041 12.6072441 -18.9936412
N 8.3366279 10.5700175 -13.8495991
H 7.428984 10.1185224 -13.7997984
C 9.2537472 10.4628666 -12.7165948
H 9.411715 11.4704627 -12.3299929
C 10.6243327 9.9036971 -13.153139
O 10.6986872 8.9385328 -13.9069108
C 8.6037136 9.6350831 -11.6036772
H 8.4204313 8.619799 -11.9600852
H 7.655759 10.0896643 -11.3114457
H 9.2647124 9.595986 -10.737355
N 11.703524 10.5367378 -12.6721267

H 11.5296608 11.3030954 -12.0418231
C 13.1142625 10.3142897 -13.0702562
H 13.6516325 11.146085 -12.6173586
C 13.4243279 10.5119185 -14.5675031
O 14.4681525 10.0599575 -15.0339048
C 13.7765064 9.0619071 -12.4395625
H 14.845682 9.122573 -12.6445486
C 13.6265139 9.0675566 -10.9118445
H 12.5803642 8.9423089 -10.6318339
H 14.0018322 10.0070241 -10.506304
H 14.2061243 8.2486758 -10.485005
C 13.2962742 7.7038875 -12.9652798
H 12.2890587 7.4911381 -12.6080123
H 13.9637118 6.9192992 -12.6112727
H 13.2950871 7.7094786 -14.0550769
N 12.605294 11.3349009 -15.2378421
H 11.7369915 11.5814565 -14.7805248
C 12.7314974 11.833814 -16.6111805
H 11.8724211 12.4921825 -16.7495073
C 12.6154246 10.7757957 -17.7237248
O 13.1625965 9.6728211 -17.644985
C 13.9716254 12.728998 -16.7542726
H 14.876663 12.1276992 -16.6566322
H 13.9650252 13.5017634 -15.9855672
H 13.9695782 13.2008787 -17.7373648
N 11.9102878 11.1457732 -18.7984009
H 11.4901366 12.0606529 -18.8311174
C 11.6113386 10.2161504 -19.8849453
H 11.4683386 9.2575284 -19.3922061
C 12.7697246 10.041093 -20.8749029
O 13.4841971 10.9883786 -21.2103005
C 10.2956217 10.5834444 -20.6018401
H 9.5642602 10.8737195 -19.8541547
H 10.4613373 11.4376453 -21.2598264
C 9.7172444 9.4047201 -21.4196476
H 10.350257 9.2350742 -22.2929309
H 8.7362979 9.7002982 -21.7986705
C 9.5899082 8.089336 -20.6183328
O 8.4815808 7.7830257 -20.1380566
O 10.6205886 7.3866881 -20.4498584
N 12.8901662 8.8305339 -21.4204041
H 12.2017952 8.1247666 -21.1470783
C 13.7998552 8.4972353 -22.5092701
H 14.1771403 9.4236569 -22.9434863
C 13.0399482 7.741936 -23.6088019
O 12.2666108 6.8259826 -23.3460136

C 14.9936922 7.7225667 -21.9245386
H 15.5173435 8.3745069 -21.2235556
H 14.6001376 6.8863099 -21.3570609
C 16.0029798 7.189281 -22.9591753
H 15.4878043 6.568367 -23.6890142
C 16.7306364 8.3210255 -23.6896215
H 17.2504768 8.9574053 -22.9726436
H 16.0235264 8.9250527 -24.2541046
H 17.4549132 7.90003 -24.3877077
C 17.0509741 6.3236455 -22.2584349
H 17.5894864 6.9110609 -21.5149428
H 17.7563171 5.9395483 -22.9960493
H 16.5630007 5.4766885 -21.778768
N 13.2962296 8.1160457 -24.8611726
H 13.9627613 8.8548464 -25.0049569
C 12.7297022 7.4720004 -26.0467334
H 11.6552424 7.6561114 -26.0668372
C 12.963355 5.9483119 -26.0268958
O 14.0825531 5.4964434 -25.7932219
C 13.3669937 8.1475173 -27.2671617
H 13.1403425 9.2139322 -27.2266732
H 14.4513269 8.049724 -27.187207
C 12.9656811 7.6189319 -28.6307211
C 12.1494424 8.3971049 -29.4761888
H 11.769383 9.3526089 -29.1454154
C 11.8565891 7.9520163 -30.7798108
H 11.2562024 8.555452 -31.4472005
C 12.3673248 6.7178848 -31.2374061
O 12.1160781 6.2857259 -32.4969706
H 11.5991475 6.9488647 -33.0185918
C 13.1697677 5.9340538 -30.3847744
H 13.5492799 4.9896701 -30.7459872
C 13.4827043 6.3907892 -29.091865
H 14.1273567 5.791667 -28.4621886
N 11.8951892 5.1788313 -26.2599184
H 11.0304658 5.6427895 -26.4800882
C 11.8426092 3.7036502 -26.2137578
H 10.7811114 3.4599108 -26.2631227
C 12.3180866 3.0493723 -24.893297
O 12.588105 1.8486882 -24.8229072
C 12.4769853 3.1028593 -27.4818833
H 13.5610504 3.0598399 -27.3602348
H 12.2582179 3.7523311 -28.3311342
C 11.9188234 1.7098089 -27.8129528
O 10.6734795 1.5608478 -27.7641107
O 12.7303627 0.8270589 -28.169355

N 12.3786583 3.8077385 -23.7892188
H 12.1567416 4.7960764 -23.8565606
C 12.7236866 3.2518266 -22.4780661
H 13.5786598 2.5902866 -22.6181944
C 11.5733328 2.4149249 -21.9209309
O 10.5345585 2.9233827 -21.5059758
C 13.1586237 4.3717878 -21.5322934
H 14.0067916 4.8753226 -21.9927235
H 12.3398627 5.0838206 -21.4074609
C 13.5708913 3.8411374 -20.1519667
H 12.6903613 3.4453154 -19.6452956
H 14.3110593 3.0473288 -20.2626016
C 14.1595742 4.9730826 -19.3074994
H 13.477531 5.8273082 -19.3386531
H 15.1255757 5.265841 -19.7198963
C 14.3246592 4.5251755 -17.8558798
H 14.9647772 3.6393135 -17.8237746
H 13.3343658 4.2459127 -17.4769636
N 14.894404 5.6206571 -17.034387
H 15.8026652 5.9022239 -17.3743342
H 14.9585333 5.3479973 -16.0632593
H 14.281514 6.4323719 -17.0835563
N 11.8145313 1.1129027 -21.796752
H 12.636759 0.7428888 -22.2469702
C 10.915173 0.206206 -21.075752
H 9.9261211 0.3116122 -21.5233934
C 10.7879049 0.6022152 -19.5986625
O 11.7565775 1.0128309 -18.9601126
C 11.3345322 -1.270057 -21.22834
H 10.6296722 -1.8783407 -20.6606041
C 11.2418011 -1.7098514 -22.6941182
H 11.9608367 -1.1650701 -23.3087444
H 10.2395993 -1.5174528 -23.0780593
H 11.4468521 -2.7773516 -22.7753339
C 12.7458793 -1.5768076 -20.7095885
H 13.4907476 -1.0077613 -21.2656222
H 12.9570051 -2.6395233 -20.8284199
H 12.8214824 -1.3296274 -19.6506744
N 9.6090573 0.3641778 -19.0190877
H 8.8636369 0.042936 -19.61778
C 9.3030641 0.4995601 -17.5765515
H 8.2498576 0.2378134 -17.4888618
C 9.3464409 1.9102799 -16.9589934
O 9.2888891 2.018324 -15.7386553
C 10.0341763 -0.545724 -16.7016046
H 9.6693134 -0.4676256 -15.6770594

C 9.7794235 -1.9797159 -17.169852
H 10.215899 -2.1450359 -18.1540049
H 8.7079161 -2.1740235 -17.2032576
H 10.239578 -2.6699839 -16.4624216
O 11.4299275 -0.3613229 -16.691399
H 11.6667592 0.1678767 -17.4727227
N 9.2779939 2.9909397 -17.7450031
H 9.3809 2.8980378 -18.7455964
C 8.8924366 4.3186524 -17.2226461
H 9.484174 4.5459203 -16.3361664
C 7.3950813 4.3363773 -16.8420819
O 6.54833 4.1563548 -17.7157283
C 9.1737252 5.4060425 -18.2724269
H 8.8412563 6.3707971 -17.8859208
H 8.6096819 5.1895781 -19.1816057
O 10.5517975 5.4957348 -18.5958262
H 10.5894134 6.1754387 -19.3178923
N 7.0367162 4.4791013 -15.5515566
H 7.7637049 4.4744686 -14.8485576
C 5.6335974 4.372326 -15.0850094
H 5.0070584 4.6318068 -15.9400818
C 5.0839267 5.3356751 -13.9496445
O 4.1802507 4.8921545 -13.2441249
C 5.3501324 2.8742188 -14.7954481
H 5.6601937 2.2734319 -15.6495461
H 4.2770818 2.7385852 -14.6680957
C 6.0481724 2.3356799 -13.5563096
O 6.9355157 2.9397106 -12.9802825
N 5.6839717 1.1536912 -13.1130988
H 6.1721809 0.8278604 -12.2984982
H 4.9434468 0.6468731 -13.5561149
N 5.4209234 6.6581869 -13.7954432
H 6.1313111 7.0377768 -14.4037847
C 4.6727308 7.6690857 -12.8730958
H 3.6722803 7.2716519 -12.7031805
H 5.1665915 7.6425688 -11.902207
C 4.4605241 9.2667223 -13.2399755
O 5.4811072 9.6663177 -13.812121
N 3.2536409 10.1109433 -12.9872607
H 2.6474233 9.6614132 -12.3206553
C 2.3771526 11.4343133 -13.6922496
H 3.1658733 12.0715482 -14.0792353
C 1.0505375 12.8198719 -12.978889
O 0.738086 12.1140384 -12.0071596
C 1.9540762 10.4352165 -14.9544058
H 0.9329988 10.1097298 -14.746017

C 2.7417697 9.1177858 -15.5124699
H 3.7821969 9.3932306 -15.6797871
H 2.6711718 8.3582525 -14.7352443
C 1.9181844 11.302737 -16.1856385
H 2.9290002 11.590086 -16.4899791
H 1.3394983 12.1952761 -15.9637221
H 1.4171187 10.8031861 -17.0150758
C 2.3461044 8.3062146 -16.7841532
H 1.2838958 8.043668 -16.7411571
H 2.9411495 7.3919643 -16.8207085
H 2.5328117 8.8854332 -17.695951
N 0.2333932 14.4003043 -13.1834479
H 0.7437184 14.7653437 -13.9704046
C -1.2123799 15.955379 -12.9597387
H -1.0028175 16.3292374 -13.960482
C -2.5312399 15.1695335 -13.1010222
H -2.3258053 14.1092915 -13.2484796
H -3.0618878 15.2689053 -12.1555772
C -3.4175086 15.6977339 -14.2585728
H -3.2432872 16.7657087 -14.3985759
C -3.0783778 14.9714564 -15.5675978
H -3.2811317 13.903087 -15.4809576
H -2.0269104 15.1037593 -15.8111044
H -3.6757972 15.3801559 -16.3812018
C -4.9110054 15.512951 -13.9555661
H -5.1400087 14.4586235 -13.8201664
H -5.5040244 15.9052164 -14.7807417
H -5.172629 16.0607214 -13.0540348
C -2.0596333 17.9254177 -12.3430272
O -1.3832877 18.2475852 -11.374691
N -3.271554 19.2249776 -12.5340521
H -3.8017849 18.9469697 -13.3433045
C -4.1853409 20.9390259 -11.9780666
H -4.1201797 20.8378689 -10.8941409
C -5.8142116 21.9798425 -12.0685304
O -6.4577917 21.990306 -13.1238488
C -3.3553264 22.2522131 -12.3368714
H -2.7508435 21.9810248 -13.199742
H -3.9431639 23.1195955 -12.6376299
C -2.3935577 22.6639575 -11.2141059
H -1.5875927 23.2551301 -11.6539897
H -1.9417954 21.7737149 -10.7710014
C -3.1027948 23.4921229 -10.1365626
O -4.1338848 23.0043489 -9.6189633
O -2.6283562 24.6174618 -9.8712537
N -6.4475046 23.0719258 -11.2698861

H -5.8681384 23.316696 -10.4654281
C -7.9445496 23.8116708 -11.2481781
H -8.5313975 23.1447577 -11.8786809
C -8.4505552 25.3183528 -11.7282815
O -7.9562339 26.3541144 -11.2608184
C -8.5827225 23.7319299 -9.8142739
H -8.3098143 22.7644516 -9.3910452
H -8.1237068 24.4929663 -9.1820639
C -10.1128287 23.8553926 -9.699581
H -10.4295997 24.8665053 -9.9608487
H -10.3884539 23.7049408 -8.6526486
C -10.8425833 22.8299823 -10.5754585
O -10.9583405 23.1023724 -11.793048
O -11.2574013 21.7859035 -10.0265513
N -9.602907 25.5151302 -12.4609812
H -10.1970189 24.6947314 -12.6038089
C -10.0093518 26.7968804 -13.139427
H -9.6033406 27.6153906 -12.5465371
C -11.5373224 27.0770783 -13.3129338
O -12.3430523 26.1660776 -13.4651874
C -9.3158021 26.7963794 -14.5218177
H -8.2652178 26.5407625 -14.3723088
H -9.7616841 25.9988297 -15.1187812
C -9.3656862 28.1006173 -15.3396433
H -10.3980501 28.3527717 -15.5746801
C -8.7151959 29.2767986 -14.6053596
H -7.6855865 29.0272619 -14.3458923
H -9.2616884 29.5103425 -13.6946444
H -8.721151 30.1580805 -15.2468748
C -8.6202634 27.902131 -16.6598573
H -7.5714266 27.6723762 -16.4704162
H -8.6904418 28.808201 -17.2612677
H -9.0736317 27.0808258 -17.214682
N -11.9412789 28.3572898 -13.4297045
H -11.2467902 29.0828588 -13.3712445
C -13.3321225 28.7589476 -13.708797
H -13.9922764 28.0787705 -13.1669539
C -13.6973559 28.668285 -15.20173
O -13.2212429 29.4599915 -16.0166546
C -13.5976231 30.1744778 -13.1694646
H -13.3339687 30.2032292 -12.1112647
H -12.9526566 30.8857408 -13.6884859
C -15.0439728 30.6091374 -13.3172145
C -16.0007793 30.2075446 -12.3654889
H -15.7026398 29.6095012 -11.5164327
C -17.3497155 30.5734271 -12.5229045

H -18.084102 30.2600018 -11.7942258
C -17.7459906 31.3401123 -13.632773
H -18.784204 31.6156339 -13.7569758
C -16.7930155 31.742311 -14.5848431
H -17.0984245 32.326439 -15.4416514
C -15.4440982 31.3771052 -14.4276294
H -14.7165289 31.6734974 -15.1713742
N -14.6326318 27.780819 -15.5396291
H -14.9521194 27.1150618 -14.8471413
C -15.2390964 27.6886577 -16.8666391
H -15.735075 28.6312197 -17.0994544
H -14.4624243 27.5134405 -17.6111349
C -16.2666536 26.5596064 -16.957317
O -16.3953891 25.7524785 -16.0401148
N -16.9764725 26.4878918 -18.0819378
H -16.8289854 27.176356 -18.8044725
C -17.9575033 25.4408391 -18.3831007
H -17.7899232 24.5827215 -17.7304426
C -17.7792012 24.9927546 -19.8397808
O -17.7959146 25.8188285 -20.750926
C -19.3742105 25.9740925 -18.1133224
H -19.4072767 26.3626889 -17.0943744
H -19.5911057 26.7896238 -18.8053619
C -20.4418095 24.8784784 -18.2588574
H -20.2170614 24.0779836 -17.5490798
H -20.3816661 24.4589508 -19.2655484
C -21.873747 25.3850101 -18.0153021
O -22.0329252 26.4946208 -17.4568844
O -22.8044396 24.6367793 -18.3888393
N -17.5576422 23.6953514 -20.0649298
H -17.6282623 23.0479037 -19.293315
C -17.3269048 23.1256682 -21.396853
H -17.6295227 23.8524687 -22.1539443
C -18.173276 21.8705255 -21.6133325
O -18.4492201 21.1344351 -20.6665456
C -15.8303326 22.833808 -21.5971231
H -15.5031065 22.1074362 -20.8521036
H -15.2664698 23.755845 -21.4523805
C -15.5235885 22.3038595 -22.9769314
N -16.0573017 22.8083599 -24.1656877
C -15.6125519 22.0028993 -25.140073
H -15.854249 22.1181749 -26.1888167
N -14.8449621 21.02673 -24.6277346
H -14.429788 20.2755673 -25.1579691
C -14.7816493 21.1953241 -23.2621255
H -14.2791179 20.561661 -22.5447457

N -18.5185024 21.5818415 -22.8701808
H -18.1432063 22.1738025 -23.6009505
C -19.2288161 20.3661674 -23.257331
H -19.9116933 20.1067755 -22.4457962
C -18.2431347 19.2015468 -23.4437471
O -17.5034608 19.1367934 -24.4310812
C -20.0753688 20.6536823 -24.5101802
H -20.7622502 21.4721288 -24.2876809
H -19.4111275 20.9769363 -25.3134978
C -20.8899068 19.4416545 -25.0031907
H -20.2061397 18.632868 -25.252551
C -21.8900833 18.9305006 -23.9653095
H -22.5664824 19.7301243 -23.6638583
H -21.3716057 18.543429 -23.0898099
H -22.4721546 18.1090986 -24.387707
C -21.6558409 19.8178908 -26.2704486
H -22.3670983 20.6156827 -26.0549008
H -22.1966695 18.9460329 -26.6408658
H -20.9593117 20.149503 -27.0399621
N -18.305586 18.2264973 -22.5363312
H -18.9848484 18.3084584 -21.7879638
C -17.4987295 17.0022879 -22.56464
H -16.4445225 17.2767669 -22.5352472
C -17.7136686 16.1851165 -23.8479637
O -16.7324037 15.7061853 -24.4089253
C -17.8183622 16.1676056 -21.3118248
H -17.6392762 16.7679502 -20.4189096
H -17.1541192 15.3031549 -21.279748
C -19.2469521 15.6902349 -21.2993199
N -20.3555822 16.5291505 -21.1770985
C -21.409785 15.8002387 -21.5780685
H -22.404937 16.1941265 -21.7444035
N -21.0189659 14.5690479 -21.940586
H -21.5461555 13.999855 -22.611493
C -19.6585309 14.4792161 -21.7690455
H -19.0246639 13.6887216 -22.1457658
N -18.9342631 16.1416122 -24.3967885
H -19.7139306 16.4659898 -23.8383305
C -19.2306073 15.4622993 -25.6698397
H -18.9191364 14.4191048 -25.5943322
C -18.4740459 16.0915514 -26.8531044
O -17.994577 15.3816032 -27.732199
C -20.7422481 15.4827005 -25.9605596
H -20.9140981 15.0341016 -26.9402223
H -21.0845436 16.5175317 -26.0055241
C -21.5715667 14.7083567 -24.9337949

O -21.1731487 13.5796127 -24.57134
O -22.5721805 15.2767049 -24.4473625
N -18.2496141 17.4112082 -26.8323886
H -18.5716361 17.9329869 -26.0288096
C -17.4531297 18.1047217 -27.8489587
H -17.7977212 17.8135617 -28.8424323
H -17.5879439 19.1789559 -27.7304308
C -15.956921 17.7942072 -27.7454374
O -15.2888172 17.6280449 -28.7627028
N -15.439025 17.6201509 -26.5247796
H -16.0609943 17.7043393 -25.7294654
C -14.0743246 17.1266898 -26.3031017
H -13.3826248 17.7038566 -26.9186169
C -13.9256189 15.6534948 -26.724003
O -12.9564171 15.2990751 -27.3953963
C -13.702554 17.3422062 -24.8291262
H -13.7609572 18.4063683 -24.6038623
H -14.431762 16.8391893 -24.1953113
C -12.3186415 16.8515113 -24.4543145
C -11.189853 17.6482056 -24.7279804
H -11.3040053 18.609059 -25.2098121
C -9.9051194 17.1911998 -24.3777829
H -9.0312386 17.792005 -24.5808524
C -9.7460546 15.9368061 -23.7535044
O -8.5019852 15.5094553 -23.4137942
H -8.5099345 14.6569228 -22.9779248
C -10.8775819 15.1370117 -23.4808996
H -10.7593813 14.1747477 -23.0085044
C -12.161335 15.5959257 -23.8337612
H -13.0275541 14.9793858 -23.6353157
N -14.9268359 14.8201123 -26.423841
H -15.7014839 15.176822 -25.8741997
C -14.9828394 13.4197593 -26.8408227
H -14.0769896 12.9181517 -26.4986893
C -15.0310294 13.2711907 -28.3685764
O -14.316041 12.4302505 -28.9112801
C -16.1878629 12.7409781 -26.1724631
H -16.0987507 12.8425083 -25.0898935
H -17.1015123 13.2506904 -26.4752072
C -16.3194695 11.2716038 -26.5208461
C -15.679925 10.2960364 -25.7306851
H -15.1296517 10.5903453 -24.8487413
C -15.7427155 8.9385754 -26.1018896
H -15.2470437 8.1817352 -25.5143806
C -16.4327316 8.5582085 -27.2723703
O -16.4601685 7.2539103 -27.6505408

H -16.8895214 7.1591939 -28.5014005
C -17.087672 9.5346431 -28.0532165
H -17.603424 9.2540987 -28.9595907
C -17.035941 10.887789 -27.6716777
H -17.5222426 11.6367727 -28.2851494
N -15.7781968 14.1209507 -29.0785084
H -16.4213335 14.735133 -28.58639
C -15.8302485 14.0991621 -30.5458371
H -16.023302 13.0723458 -30.857743
C -14.4935853 14.5210283 -31.1785911
O -13.967663 13.81329 -32.0333803
C -16.994066 14.9750367 -31.0424011
H -17.90762 14.6865268 -30.519727
H -16.7819786 16.0183928 -30.8031416
C -17.238316 14.8508303 -32.5555673
H -16.3290961 15.1177248 -33.0992785
H -18.0038016 15.5779021 -32.8375267
C -17.6986865 13.4395358 -32.9540066
O -18.9264795 13.2539991 -33.097649
O -16.8225467 12.5548429 -33.0888592
N -13.837982 15.5729289 -30.6701081
H -14.2892367 16.1201696 -29.9440593
C -12.4898729 15.9648231 -31.1351682
H -12.5346458 16.1337827 -32.2113024
C -11.4694612 14.8360737 -30.9194148
O -10.6875397 14.5295424 -31.8238719
C -12.0510367 17.2817301 -30.4618222
H -12.2135606 17.2075124 -29.385962
C -10.5745282 17.6261432 -30.7001145
H -10.360449 17.6415137 -31.7693089
H -9.9326989 16.8899406 -30.2164525
H -10.3471552 18.6032195 -30.2737645
C -12.8768666 18.454303 -31.009272
H -12.6844 18.5843449 -32.0745506
H -12.6143381 19.3701809 -30.4802161
H -13.9403605 18.2682826 -30.8668087
N -11.5418689 14.1369177 -29.7810758
H -12.2068198 14.4377171 -29.0741423
C -10.7457172 12.934077 -29.5229422
H -9.6880299 13.1653715 -29.6509979
H -10.9128238 12.6231638 -28.4919327
C -11.1015855 11.7534031 -30.4368387
O -10.2052046 11.0525872 -30.9091058
N -12.3806552 11.5703557 -30.7665667
H -13.0669852 12.1981408 -30.3596135
C -12.8830576 10.4904933 -31.6335773

H -12.4536585 9.5465298 -31.299618
C -12.4546029 10.6930136 -33.0856704
O -11.8379689 9.7967565 -33.662114
C -14.4115139 10.3664975 -31.5108397
H -14.8837716 11.3195435 -31.7430037
C -15.0075931 9.281508 -32.405127
H -14.497612 8.3332624 -32.2385487
H -14.9184434 9.5764776 -33.4497101
H -16.0681325 9.1738969 -32.1753859
O -14.7191285 9.9990169 -30.1858573
H -14.6611214 10.8041515 -29.6445041
N -12.6061399 11.9050074 -33.6278135
H -13.1277568 12.5991368 -33.0943308
C -12.1108057 12.2899864 -34.96069
H -12.5626445 11.6330904 -35.7039491
C -10.5924574 12.1086371 -35.0606404
O -10.1118234 11.5017037 -36.0194828
C -12.5253388 13.7419652 -35.2846723
H -12.2047179 14.3950538 -34.4716908
C -11.9014053 14.2583629 -36.5896139
H -12.1485647 13.587198 -37.4129321
H -10.8182307 14.3273054 -36.4922998
H -12.2846337 15.2548982 -36.8119629
C -14.0473735 13.8569901 -35.4397281
H -14.390893 13.2481522 -36.2760117
H -14.3288191 14.8958381 -35.6094773
H -14.5551193 13.5168148 -34.5364626
N -9.8327613 12.5183483 -34.0376294
H -10.2691124 13.0158676 -33.2677802
C -8.3866883 12.2947826 -33.9964652
H -7.94803 12.713808 -34.9032965
C -8.0253687 10.7951613 -33.964585
O -7.1750255 10.3535353 -34.7385592
C -7.808095 13.0498299 -32.7940855
H -8.2322251 12.6640796 -31.8665257
H -8.0425363 14.1119177 -32.8768195
H -6.7249933 12.9258191 -32.7693519
N -8.7383063 9.9928143 -33.1690346
H -9.4399586 10.4216383 -32.5757625
C -8.5261026 8.5373236 -33.0519002
H -7.476906 8.3564513 -32.8190178
C -8.836892 7.7959867 -34.3557814
O -8.0721417 6.9214099 -34.7582541
C -9.3671494 7.960609 -31.9009351
H -10.4270834 8.1175921 -32.1029293
C -9.1210937 6.4714297 -31.6602582

H -8.0569718 6.2924769 -31.5037895
H -9.4663804 5.8883818 -32.5134126
H -9.6722286 6.1524028 -30.7752524
O -9.0287859 8.6085484 -30.6970051
H -9.4300372 9.4946216 -30.711502
N -9.8765834 8.2071453 -35.0894348
H -10.4751493 8.9342993 -34.7066042
C -10.218649 7.6484065 -36.4087586
H -10.3078933 6.565189 -36.3149444
C -9.0961985 7.9031619 -37.4291875
O -8.8107229 7.0319327 -38.2457573
C -11.5883493 8.2058925 -36.8724934
H -11.5800876 9.2895272 -36.7418409
C -11.8649231 7.9080697 -38.3605028
H -11.8182208 6.8336763 -38.5433383
H -11.1330206 8.4106419 -38.9928817
H -12.8465962 8.2794081 -38.6492608
C -12.7194695 7.6055139 -36.002287
H -12.8725628 6.5606242 -36.275951
H -12.4291906 7.6247014 -34.9527788
C -14.0547499 8.3529914 -36.1158443
H -14.469557 8.2558855 -37.1180749
H -13.9111854 9.4081356 -35.8800284
H -14.765007 7.9268607 -35.4065953
N -8.3937191 9.0388911 -37.3367375
H -8.6846259 9.7176446 -36.6427142
C -7.265239 9.3812153 -38.2229573
H -7.5533014 9.1179589 -39.2408252
C -5.9497281 8.6286735 -37.9283472
O -4.9741865 8.7465544 -38.6840646
C -7.0390962 10.899473 -38.1899562
H -8.0014138 11.4136505 -38.2093944
H -6.4800146 11.1924024 -39.0771632
O -6.2990547 11.3175345 -37.05805
H -6.6658562 10.914714 -36.2473093
N -5.8900761 7.8921141 -36.8100548
H -6.7249579 7.8243468 -36.2397125
C -4.6565738 7.3302429 -36.256341
H -3.8362244 8.0007177 -36.5123651
C -4.2800623 5.9408626 -36.8074929
O -3.1352983 5.5205919 -36.6491747
C -4.7709993 7.3240925 -34.7269169
H -5.5236596 6.6026775 -34.4107831
H -5.0474193 8.3167635 -34.3677705
H -3.8122191 7.0434425 -34.2890576
N -5.1800992 5.2659515 -37.5310566

H -6.0659794 5.7270073 -37.7010793
C -4.9863126 3.9392569 -38.1512939
H -5.0038412 3.1815968 -37.3671395
C -3.6467069 3.7571432 -38.8886477
O -2.9731921 2.7386014 -38.7264792
C -6.1697208 3.6740353 -39.1048049
H -7.0402153 3.4328757 -38.4935022
H -5.9499646 2.7970377 -39.7150907
C -6.5637775 4.8197352 -40.0201111
N -5.7699764 5.9207787 -40.3641479
C -6.5674691 6.752385 -41.049963
H -6.277414 7.7318812 -41.4028678
N -7.7926363 6.2244398 -41.1826337
H -8.5854946 6.6964794 -41.5940588
C -7.8129371 5.0107498 -40.5332027
H -8.6720512 4.3747877 -40.3736965
N -3.1845526 4.7824287 -39.6094995
H -3.8397485 5.5490973 -39.7402001
C -1.9094941 4.7790629 -40.3503428
H -1.9058746 3.9210138 -41.0259016
C -0.6755178 4.6000095 -39.4590681
O 0.3500762 4.1523355 -39.9634635
C -1.8108003 6.0655976 -41.1818839
H -1.9802055 6.9149351 -40.5182176
H -0.8101594 6.155225 -41.6088882
C -2.8366963 6.0737128 -42.3295833
H -3.7805566 5.6467642 -41.9998432
H -2.4675716 5.4691415 -43.158521
C -3.090666 7.5062857 -42.7975055
H -2.21557 7.8834898 -43.328123
H -3.2604295 8.1357064 -41.9227019
N -4.2898586 7.6146451 -43.6353146
H -5.0985667 8.0752973 -43.2163844
C -4.3632322 7.5927761 -44.9465972
N -3.4118636 7.0991039 -45.680707
H -3.2172373 7.5904414 -46.5573116
H -2.6560279 6.644648 -45.2205878
N -5.4028833 8.1065841 -45.5234574
H -6.0523492 8.6180683 -44.9202338
H -5.4423454 8.1830156 -46.5185052
N -0.7546542 4.8641941 -38.1528525
H -1.6478001 5.1514024 -37.7665882
C 0.3441051 4.6164829 -37.2169392
H 1.2323201 5.1264964 -37.5885598
C 0.6996833 3.1210416 -37.0906775
O 1.8637562 2.7984179 -36.8587498

C -0.0133202 5.2297426 -35.858726
H -0.9017486 4.748926 -35.4486878
H -0.2065543 6.2971976 -35.9704315
H 0.8169899 5.0909097 -35.1647239
N -0.2423564 2.2117153 -37.3765593
H -1.1831707 2.5200405 -37.5942453
C 0.0441412 0.7786215 -37.4595675
H 0.5673747 0.4724402 -36.5515695
C 0.9552045 0.4341165 -38.6550826
O 1.7906982 -0.4590169 -38.5465271
C -1.2872208 0.0221727 -37.522223
H -1.8167315 0.2672544 -38.4447155
H -1.9079066 0.2944651 -36.668599
H -1.1024213 -1.0504495 -37.4916568
N 0.8781965 1.1977947 -39.7544587
H 0.2102942 1.9555281 -39.7611379
C 1.8112123 1.0873253 -40.8905783
H 2.0039892 0.0301488 -41.0804667
C 3.1532747 1.7347408 -40.5479506
O 4.1918602 1.1211358 -40.7786454
C 1.2242149 1.7004207 -42.1796402
H 1.0712918 2.7691655 -42.0362597
C 2.1680245 1.5163423 -43.3761676
H 2.3959256 0.458749 -43.5151264
H 3.0968456 2.0616945 -43.2111974
H 1.7047064 1.9050683 -44.2829471
C -0.1270984 1.0684292 -42.5415657
H -0.0134202 -0.0094525 -42.6618157
H -0.4995357 1.4948188 -43.4727939
H -0.8607535 1.2658962 -41.7604837
N 3.1497115 2.9075864 -39.8983128
H 2.2584563 3.3646896 -39.7477886
C 4.3829764 3.5769137 -39.4271644
H 5.0316617 3.7532182 -40.287047
C 5.1714679 2.6870662 -38.4572727
O 6.3914124 2.6466122 -38.5342007
C 4.0854947 4.9447377 -38.7734971
H 3.4492565 4.7868126 -37.9038288
C 5.3579813 5.667574 -38.3077099
H 6.0473034 5.7700222 -39.1479344
H 5.8520541 5.0988091 -37.5198768
H 5.1091859 6.6515776 -37.9116408
C 3.3657123 5.9094878 -39.7292705
H 4.0142422 6.146524 -40.5715909
H 3.1117469 6.8290265 -39.2030367
H 2.4524687 5.4645623 -40.1099666

N 4.5099699 1.8691594 -37.6261136
H 3.501018 1.9661057 -37.5679173
C 5.1768327 0.8861925 -36.7517641
H 5.9189905 1.4157616 -36.1524491
C 5.9655105 -0.1805465 -37.5278367
O 6.9655235 -0.6580613 -37.005066
C 4.1151826 0.2654933 -35.821984
H 3.6872668 1.0584642 -35.2070059
H 3.3115988 -0.1409395 -36.4366679
C 4.6026419 -0.8663175 -34.8987054
H 4.9701494 -1.6946528 -35.5041773
H 3.7425868 -1.2253198 -34.3369632
C 5.691348 -0.4487287 -33.8979481
H 5.305648 0.3329929 -33.2411631
H 6.5347315 -0.0299299 -34.4515579
N 6.1852786 -1.5988657 -33.1065998
H 7.0302672 -2.023504 -33.4565587
C 5.6931212 -2.0929878 -31.9796119
N 4.6137617 -1.6238221 -31.4121932
H 4.2496792 -2.0315972 -30.5644291
H 4.1500759 -0.8388203 -31.8268032
N 6.2826626 -3.0960025 -31.392016
H 7.158683 -3.4460252 -31.7437805
H 5.9220268 -3.4384356 -30.5210906
N 5.5130667 -0.5786026 -38.7196586
H 4.6989518 -0.1098716 -39.0946508
C 6.155844 -1.6215128 -39.5477068
H 6.7171868 -2.2989085 -38.9044692
C 7.1870424 -1.0471198 -40.5141252
O 8.1672071 -1.7115576 -40.8379074
C 5.0729423 -2.429844 -40.2970744
H 4.4719049 -1.7392024 -40.8919679
C 5.6505611 -3.4778044 -41.2668926
H 6.3652394 -4.1194898 -40.7503349
H 6.1577817 -2.9777053 -42.0922651
H 4.8537515 -4.0898952 -41.6888069
C 4.1272498 -3.1381766 -39.3001663
H 3.4521743 -2.3981917 -38.8745679
H 3.519856 -3.8567523 -39.8472324
C 4.8022508 -3.8790266 -38.1377464
H 5.1620025 -3.1674568 -37.3956499
H 5.6444629 -4.4611102 -38.5038849
H 4.0797422 -4.5405924 -37.6627072
N 6.9634562 0.1827065 -40.9660073
H 6.1059518 0.645179 -40.6903566
C 7.8918005 0.9019409 -41.8307362

H 8.3560989 0.1968688 -42.5199967
C 9.0541694 1.5083209 -41.0392823
O 10.1751688 1.4175308 -41.5113396
C 7.0910835 1.9372281 -42.6425295
H 6.4755174 2.5141194 -41.9494156
C 8.015055 2.9405889 -43.3353853
H 8.8170607 2.4130698 -43.8517664
H 8.4436482 3.6079035 -42.5859543
H 7.4537262 3.5413332 -44.0376991
C 6.1329575 1.2547909 -43.6511673
H 5.3388 0.7519175 -43.1009127
H 5.6559528 2.0240523 -44.2518133
C 6.7607953 0.2206656 -44.5975052
H 7.0017951 -0.6915304 -44.0477814
H 7.6692296 0.6123557 -45.0487245
H 6.0481605 -0.0291608 -45.3837918
N 8.798992 1.9819869 -39.8195457
H 7.8269675 1.9509433 -39.5448266
C 9.6149082 2.8267209 -38.9312898
H 9.2170332 2.5797365 -37.9459199
C 9.3250434 4.3409519 -39.0306445
O 8.5238253 4.7993794 -39.8542185
C 11.099113 2.4020543 -38.8279642
H 11.7082873 3.1408157 -39.3434734
H 11.2510327 1.4269978 -39.2904088
C 11.5965131 2.3038259 -37.3833269
O 11.4492724 3.3205405 -36.6703128
O 12.1249463 1.2330349 -37.0094698
N 9.8588958 5.1058401 -38.0754761
H 10.516748 4.6466534 -37.4396315
C 9.3325992 6.4041998 -37.6512809
H 8.3526531 6.2042132 -37.2199303
C 9.1395831 7.4234129 -38.7896545
O 10.0494829 7.7492656 -39.5515223
C 10.2024572 6.9760756 -36.5200309
H 10.3373002 6.2059069 -35.7593743
H 11.1814432 7.2466722 -36.9189787
C 9.5601829 8.2096529 -35.8657287
H 8.5933201 7.9247972 -35.443284
H 9.3792816 8.9717041 -36.6261681
C 10.4478246 8.8008571 -34.7647909
O 10.8020372 8.0456266 -33.8314376
O 10.7428137 10.012847 -34.8410345
N 7.9356494 8.0039627 -38.8202964
H 7.2747294 7.6949909 -38.1276181
C 7.4979327 9.066309 -39.7246913

H 6.4475177 9.2219085 -39.4784392
C 7.4607978 8.7250306 -41.2328113
O 7.2182063 9.6195186 -42.0501456
C 8.189382 10.3821855 -39.339387
H 9.2533861 10.3291555 -39.5703739
H 8.0772996 10.5585665 -38.2684137
H 7.7299772 11.2135753 -39.8729389
N 7.5456185 7.4423073 -41.6155564
H 7.7541 6.7354562 -40.9135248
C 7.344726 6.9801426 -42.997353
H 6.9802142 7.8289962 -43.5714683
C 6.2228111 5.9398072 -43.1423331
O 5.8136015 5.298277 -42.1811172
C 8.7040589 6.5417867 -43.5937623
H 9.3769214 6.2789443 -42.781539
H 8.5857406 5.6690598 -44.2357264
C 9.2911549 7.7100855 -44.425368
H 9.1907816 8.6410518 -43.8675988
C 10.7676023 7.5379856 -44.7641775
H 10.9094523 6.6899523 -45.4326336
H 11.347273 7.4053009 -43.8535032
H 11.1408564 8.4293545 -45.2677901
C 8.5546316 7.8484516 -45.7653518
H 8.6226347 6.9085588 -46.3141622
H 9.0244539 8.6267692 -46.3653165
H 7.5122898 8.121164 -45.6183874
N 5.6747873 5.8134553 -44.3622881
H 6.051826 6.3868802 -45.1032308
C 4.5891328 4.8649351 -44.6980872
H 4.5631062 4.1139725 -43.9051263
C 4.8447935 4.0459821 -45.973334
O 3.9017478 3.6498304 -46.6520897
C 3.2105171 5.5603428 -44.666108
H 2.4533252 4.7738203 -44.6831793
H 3.1054279 6.0864467 -43.7153432
C 2.9161759 6.5454592 -45.8179066
H 3.1261552 6.0697121 -46.7735315
H 3.5448542 7.4315362 -45.7241825
C 1.4308664 6.9484155 -45.7776237
H 1.2590212 7.5693356 -44.8966521
H 0.8212512 6.0482975 -45.6740764
N 0.981203 7.6811794 -46.9780385
H 0.7524881 8.6673509 -46.8923419
C 0.6228584 7.177358 -48.1443321
N 0.8186153 5.929421 -48.4685675
H 0.5752193 5.6132175 -49.3870961

H 1.388143 5.3577715 -47.8677134
N 0.0406873 7.949872 -49.0092621
H -0.2933387 8.855375 -48.6610897
H -0.2623347 7.6163466 -49.9005672
N 6.1190758 3.8578277 -46.3209588
H 6.8301691 4.1363819 -45.6635574
C 6.5958218 3.2428633 -47.5626859
H 5.830005 2.5719029 -47.9542416
C 7.8916732 2.4367347 -47.343168
O 8.5668267 2.64766 -46.3401455
C 6.8674228 4.3491416 -48.5851797
H 7.7970464 4.8464069 -48.304794
H 7.032437 3.8770175 -49.5533758
C 5.793543 5.4093771 -48.7466088
C 4.5694425 5.071977 -49.3430479
H 4.3617759 4.0434651 -49.5976387
C 3.6563468 6.0786253 -49.6922932
H 2.7837729 5.8310304 -50.2772817
C 3.9157208 7.4151039 -49.3467661
H 3.2349086 8.196869 -49.6514519
C 5.0976506 7.7468283 -48.6644446
H 5.3156042 8.7854837 -48.4512446
C 6.0470079 6.7471097 -48.3865945
H 6.9997683 7.0245668 -47.966154
N 8.2414517 1.5676536 -48.3001291
H 7.6597218 1.5224626 -49.118932
C 9.3979198 0.6578697 -48.2914649
H 9.1016447 -0.2646165 -47.791358
C 10.6602547 1.1969945 -47.5742744
O 11.1786285 2.245083 -47.9427162
C 9.7087369 0.3224341 -49.75895
H 9.967396 1.234773 -50.3010873
H 8.8483582 -0.1504811 -50.2328802
H 10.5608699 -0.3531544 -49.8204047
N 11.1058984 0.4683517 -46.5404187
H 10.6020477 -0.3756197 -46.3181145
C 12.3498292 0.6411544 -45.7620579
H 12.0842348 0.2321049 -44.78769
C 12.7677959 2.0582833 -45.3295639
O 13.9257997 2.2966503 -44.965395
C 13.488643 -0.2542563 -46.2966031
H 14.4106292 0.3125754 -46.3885813
H 13.233996 -0.5667301 -47.3062372
C 13.7404825 -1.5012609 -45.4212878
O 12.782663 -1.9446545 -44.7479203
O 14.8558588 -2.0616043 -45.4835954

N 11.7676098 2.9221305 -45.1175289
H 10.8663841 2.6705479 -45.5032949
C 11.8434355 4.1137479 -44.2565654
H 10.9702733 4.7131008 -44.4953178
C 13.0474841 5.0013958 -44.5690478
O 13.2566867 5.4146228 -45.7033659
C 11.6839069 3.7215239 -42.7654516
H 10.7042933 3.2772 -42.7433744
C 12.7172875 2.6995277 -42.243958
H 13.7411237 3.021697 -42.4103113
H 12.5627012 1.7344082 -42.7242798
H 12.5786212 2.5252549 -41.1832717
C 11.6078686 4.8907969 -41.7666777
H 12.5088229 4.963613 -41.1569353
H 10.7748965 4.7152793 -41.0967139
H 11.4403098 5.837781 -42.2673589
N 13.7997814 5.3551081 -43.5357305
H 13.5427582 4.9757027 -42.6392564
C 14.9722896 6.1999847 -43.5773004
H 14.7513394 7.1316738 -44.1038787
C 16.1325397 5.5277022 -44.3124657
O 16.971215 6.2121722 -44.8791594
C 15.3363167 6.5059358 -42.1167684
H 14.5993989 7.1982355 -41.7073855
H 15.2826306 5.5845827 -41.5328418
C 16.7149966 7.0988042 -41.9408267
C 16.9780411 8.3984896 -42.403626
H 16.1908624 8.9809401 -42.8654804
C 17.7536204 6.3218351 -41.3924162
H 17.5600729 5.3123153 -41.0611771
C 18.2759221 8.9240865 -42.299878
H 18.4802673 9.9218835 -42.6403416
C 19.0546992 6.8452967 -41.3078177
H 19.8555147 6.2433117 -40.90308
C 19.314826 8.149128 -41.7601691
H 20.3159865 8.5514257 -41.6963891
N 16.2058513 4.196207 -44.3268838
H 15.3924863 3.6449569 -44.0809636
C 17.3365519 3.5317345 -44.9880782
H 18.247824 4.0148928 -44.6328091
C 17.3362498 3.7162578 -46.5202865
O 18.411362 3.6889561 -47.1212283
C 17.4139431 2.0551039 -44.544089
H 17.2640812 2.0186564 -43.4640312
C 18.7685605 1.4097662 -44.8475106
H 18.7772956 0.3945679 -44.4540022

H 19.5699411 1.9849547 -44.3903993
H 18.9200973 1.3641078 -45.9264033
O 16.4573901 1.2286298 -45.1599023
H 15.5577683 1.5969757 -45.0914068
N 16.1818698 4.0690221 -47.1078517
H 15.3465728 4.1192239 -46.5422669
C 16.0387294 4.3659216 -48.539581
H 16.9320402 3.9392428 -48.9945931
C 16.0907108 5.8966401 -49.0803814
O 16.0794458 5.9767522 -50.2908172
C 14.8556147 3.4957892 -49.0773819
H 13.9798023 4.1366509 -49.1933825
H 14.5968292 2.7346661 -48.3378472
C 15.1275989 2.7525254 -50.4024108
O 14.1400315 2.2286328 -50.965854
O 16.307982 2.6299838 -50.8096799
N 16.2900343 7.1238414 -48.4203981
H 16.4497442 7.0131143 -47.4277553
C 16.1613041 8.688626 -48.8951202
H 15.5643498 8.6270493 -49.8051702
C 17.4543756 9.8556596 -49.3389581
O 18.4787587 9.2741032 -49.0634247
C 15.1839224 9.2297703 -47.7945255
H 14.6070166 8.4267525 -47.3302822
H 14.4664933 9.8869038 -48.2885934
C 15.9020718 10.0680588 -46.713811
O 16.3872989 11.1886415 -47.009515
O 15.9809798 9.6471257 -45.537687
N 17.6582507 11.2710311 -50.0187799
H 16.7432841 11.6473397 -50.215925
C 18.9196021 12.4999217 -50.4996121
H 19.4239745 12.7072473 -49.557024
C 19.8910586 11.6284874 -51.3214552
H 19.3040143 10.926469 -51.916006
H 20.4598406 12.2416738 -52.0211191
C 20.8996192 10.8577388 -50.4511839
H 20.4908512 10.6758339 -49.4595413
H 21.799341 11.4600084 -50.3269145
C 21.2793501 9.5187183 -51.0747255
O 22.2065281 9.4215954 -51.8603748
N 20.5592735 8.459898 -50.7803827
H 20.794535 7.5910496 -51.2218482
H 19.7724068 8.5434667 -50.1355936
C 19.092366 14.182846 -51.3913657
O 18.0971829 14.350783 -52.0856275
N 20.1085432 15.3856543 -51.5493382

H 20.9260687 15.212857 -50.986138
C 20.2948739 16.9461427 -52.3131094
H 20.2227503 16.7196393 -53.3770916
C 21.4078328 18.3179152 -52.2406162
O 22.5468109 18.183726 -51.7988277
C 19.0686253 17.7464106 -51.9697146
H 19.2338989 18.2728335 -51.0391949
H 18.2005998 17.0932304 -51.9204808
H 18.8667864 18.4802587 -52.7438545
N 21.1702238 19.6749261 -52.6514902
H 20.2345851 19.8379585 -52.9935248
C 22.0024176 21.0115075 -52.4479166
H 22.8683875 20.7431257 -51.8441611
C 21.2832516 22.1579803 -51.6588977
O 20.098186 22.3880363 -51.8856444
C 22.5113401 21.7661131 -53.7349204
H 21.6390627 22.0773934 -54.3111731
H 23.0283835 22.6774737 -53.4283394
C 23.4507222 21.0203275 -54.6889031
H 22.9115143 20.1707727 -55.1106251
H 23.7236569 21.6915994 -55.5045992
C 24.7266258 20.5101083 -54.0066815
H 24.4530278 19.8220364 -53.2042071
H 25.2791937 21.3544152 -53.5879837
C 25.5984771 19.7681775 -55.0268131
H 25.909443 20.4728439 -55.8051529
H 24.9888066 18.9920389 -55.5006992
N 26.7854235 19.1515691 -54.3842775
H 27.3653918 19.8580064 -53.9512649
H 27.3408378 18.6543916 -55.0695705
H 26.4940335 18.4923601 -53.6723818
N 22.0141776 22.9790716 -50.8776924
H 22.9848334 22.7561847 -50.7481297
C 21.4363151 24.0595014 -50.0406279
H 20.6651534 23.5880877 -49.4217957
C 20.6932301 25.1571828 -50.8434591
O 21.1123859 25.4928957 -51.9501055
C 22.4954675 24.6021902 -49.0576117
H 21.9681595 25.0697176 -48.2281703
H 23.0446883 23.7611733 -48.6298199
C 23.4967813 25.6136439 -49.6395768
H 24.1308012 25.1185841 -50.3761876
H 22.9616084 26.426131 -50.1294037
C 24.3600993 26.2058559 -48.5122502
H 25.0695733 25.4548231 -48.159759
H 23.7129745 26.4715583 -47.6719088

C 25.1007745 27.47991 -48.9414417
H 25.6487684 27.8643149 -48.0751497
H 24.3538345 28.231996 -49.2207631
N 26.033051 27.2429212 -50.0717632
H 26.729265 26.5557402 -49.8134378
H 26.5058074 28.1019389 -50.3270792
H 25.5216444 26.9054137 -50.8778729
N 19.6653497 25.7902966 -50.2502701
C 18.5260935 26.4199977 -50.9302971
H 17.683172 26.2611953 -50.25573
C 17.9666354 25.9230463 -52.2825018
O 17.2120042 26.6858309 -52.8855268
C 18.8245867 27.9163968 -50.8994079
H 19.6064095 28.1600803 -51.6210341
H 17.9311673 28.5198536 -51.0658878
C 19.9982801 26.7186724 -49.1631419
H 19.6497361 26.3469128 -48.204326
H 21.0706298 26.8855675 -49.103357
C 19.3411503 28.0678737 -49.4726293
H 18.5032263 28.2293829 -48.7937525
H 20.0551531 28.8885143 -49.395854
N 18.1444302 24.6585658 -52.7170806
H 18.7575415 24.0313991 -52.2081714
C 17.2497154 24.0944877 -53.7603078
H 17.1809009 24.8305569 -54.5644565
C 15.8108259 23.9536811 -53.2184155
O 15.6116559 23.7733122 -52.0168994
C 17.7905208 22.7718561 -54.367871
H 18.2164828 22.1394345 -53.5915213
H 16.9504542 22.2274091 -54.799801
C 18.8275157 22.9966047 -55.490398
H 19.7490875 23.3914824 -55.0618205
H 18.4292468 23.7618966 -56.1540239
C 19.1471149 21.7322105 -56.331149
H 18.3107319 21.0347568 -56.2604309
H 20.0375569 21.2460875 -55.9308658
N 19.3493069 22.0762304 -57.7577649
H 19.2235844 23.0639289 -58.0004847
C 19.3902273 21.3013719 -58.8329725
N 19.5688706 20.0105413 -58.789513
H 19.5868354 19.4947518 -59.6476506
H 19.6328429 19.5190873 -57.9186205
N 19.2411043 21.8219077 -60.01574
H 18.7989704 22.7503794 -60.07748
H 19.2658129 21.2691169 -60.8598066
N 14.821947 23.9635607 -54.1171727

H 15.0640462 24.1295175 -55.0795782
C 13.4070457 23.7053603 -53.8023649
H 13.2318405 23.8314889 -52.7303655
C 13.0148305 22.2690653 -54.1901151
O 13.2650034 21.8554559 -55.3268438
C 12.5042714 24.70102 -54.554355
H 11.4657565 24.4018961 -54.4176821
H 12.7363243 24.6603459 -55.6166903
C 12.5918951 26.14774 -54.1111584
O 13.1869008 26.5106661 -53.1113018
N 11.9857542 27.0281791 -54.8750885
H 12.0663734 27.990201 -54.6088372
H 11.474667 26.7304553 -55.7012412
N 12.3104354 21.5618859 -53.3039725
H 12.1319232 21.9882772 -52.3959269
C 11.7798987 20.2088839 -53.5218785
H 12.1354009 19.8236533 -54.4750295
C 10.2499869 20.2006078 -53.5304971
O 9.636071 20.6928764 -52.5837385
C 12.2939867 19.2814334 -52.4104324
H 12.0003572 19.7111831 -51.4550013
H 13.3811827 19.2399207 -52.459261
C 11.7331357 17.8531895 -52.451972
H 10.6473428 17.8812916 -52.5280063
H 11.9562163 17.3702826 -51.5054196
S 12.3827862 16.813984 -53.7779941
C 13.8931229 16.2105975 -52.9728887
H 14.4627188 15.602152 -53.6752431
H 13.6285852 15.6018071 -52.1075691
H 14.5036321 17.0516099 -52.6454799
N 9.6561252 19.476022 -54.4810838
H 10.2356146 19.1014098 -55.228018
C 8.2618632 19.0294284 -54.4110718
H 7.7432147 19.5950109 -53.6411374
C 8.2012683 17.5367186 -54.0391612
O 8.6091906 16.6916482 -54.8315145
C 7.5848191 19.3377223 -55.7563497
H 7.6533027 20.404727 -55.9472126
H 8.1407942 18.8235216 -56.5354205
C 6.1094319 18.9161937 -55.8471269
H 6.0223168 17.8621598 -55.5987058
C 5.2008171 19.7105303 -54.9111302
H 5.2842959 20.7773402 -55.1160026
H 5.4789999 19.5191252 -53.8783082
H 4.1706631 19.3882301 -55.0433692
C 5.5958432 19.1157678 -57.2735893

H 5.6369121 20.1715894 -57.5396105
H 4.5696247 18.7588489 -57.3505647
H 6.2159663 18.5490014 -57.9673902
N 7.650806 17.207511 -52.8705797
H 7.2809473 17.9542867 -52.2913677
C 7.3710433 15.8279812 -52.4464654
H 8.0060151 15.1508264 -53.0126345
C 5.9027388 15.4793458 -52.7611271
O 4.9709185 16.075372 -52.2065813
C 7.75986 15.6405981 -50.9667627
H 7.1203425 16.2573012 -50.3370099
H 8.7862314 15.9905422 -50.8370111
C 7.6824443 14.1863395 -50.4760692
O 6.8708591 13.4090086 -51.019936
O 8.4087115 13.8653462 -49.5055914
N 5.6864177 14.5711102 -53.7189983
H 6.4974395 14.0713565 -54.0777662
C 4.3447916 14.1842873 -54.1843736
H 3.6345714 14.9349283 -53.8406842
C 3.9351687 12.8544228 -53.5605881
O 4.3232699 11.7831128 -54.0192536
C 4.2336867 14.1575708 -55.7201635
H 4.8568936 13.3737071 -56.1278179
C 2.7974381 13.8629456 -56.1479142
H 2.1426664 14.6095088 -55.703318
H 2.4969621 12.8683791 -55.8186253
H 2.7229068 13.8922377 -57.2343704
C 4.6461988 15.4961607 -56.3443168
H 4.0765308 16.3124174 -55.897042
H 4.4772019 15.482627 -57.4198195
H 5.7094427 15.6583488 -56.1699027
N 3.0257182 12.9205146 -52.5909151
H 2.717312 13.8404901 -52.2952782
C 2.6511322 11.7941466 -51.7378099
H 1.5919382 11.8736081 -51.495824
H 2.826334 10.8515972 -52.2505211
C 3.4378837 11.764253 -50.4292475
O 3.7783718 10.6859284 -49.9559292
N 3.6725557 12.9262538 -49.8125931
H 3.3218807 13.7634739 -50.2589932
C 4.5639542 13.1282033 -48.6574106
H 5.5763021 12.8435635 -48.9560907
C 4.2394702 12.317265 -47.379758
O 4.9944892 12.3378076 -46.4012804
C 4.557135 14.6320349 -48.3562011
H 4.9178299 15.1702423 -49.2356233

H 5.2327209 14.8285762 -47.5267004
S 2.8894722 15.2074225 -47.9074698
H 2.7061254 14.4032963 -46.8479299
N 3.0995178 11.6227276 -47.3420058
H 2.5537956 11.6005599 -48.1895828
C 2.6973692 10.7695608 -46.2284903
H 1.7296098 10.3306215 -46.4382888
H 3.4252333 9.964224 -46.1301078
C 2.5964225 11.5335775 -44.9096616
O 2.0756425 12.6458024 -44.8617237
N 3.1475683 10.9405853 -43.8409104
H 3.5995258 10.050913 -43.9813798
C 3.2644505 11.5676352 -42.5110625
H 2.5363332 12.3721322 -42.4922009
C 4.6024855 12.3215117 -42.2989558
O 4.9330438 12.7035656 -41.1837733
C 2.7685976 10.6219858 -41.3876643
H 3.329892 9.6866489 -41.3966698
H 2.9110664 11.1133101 -40.4234859
C 1.2482459 10.3497499 -41.5881163
H 0.7919812 11.3134804 -41.7977461
H 1.1127473 9.7337692 -42.4784474
C 0.4227669 9.7161131 -40.4391496
H 0.7314226 10.1512193 -39.4901884
H 0.5960085 8.639396 -40.4025918
C -1.0758004 10.0217816 -40.7095582
H -1.41602 9.4220592 -41.5561837
H -1.1388727 11.0716698 -41.0127608
N -2.0107233 9.8625097 -39.5598994
H -2.0996365 8.9127489 -39.2334567
H -2.9406933 10.1949484 -39.8135329
H -1.7309402 10.4529809 -38.7839952
N 5.3030928 12.6456133 -43.3932679
H 4.9509657 12.3158047 -44.2839729
C 6.263606 13.7539353 -43.4685514
H 6.339384 14.0373157 -44.5168193
H 5.8664254 14.6025745 -42.9108326
C 7.6966603 13.5217271 -42.9844836
O 8.5229441 14.4189115 -43.1446022
N 8.052298 12.3272621 -42.5032093
H 7.3432049 11.6119669 -42.3818921
C 9.4128024 12.0200965 -42.040962
H 9.6646061 12.6654394 -41.1981862
H 9.4474178 10.9834761 -41.7123813
C 10.481707 12.1834795 -43.1192423
O 11.5222019 12.7773601 -42.8556136

N 10.1750316 11.7843984 -44.3589289
H 9.2846575 11.3332788 -44.4955374
C 11.0186339 12.0413754 -45.5425943
H 11.9980506 11.588156 -45.3790969
C 11.2701527 13.5389522 -45.7486745
O 12.4236188 13.9518113 -45.8260238
C 10.4258214 11.3832015 -46.8083101
H 10.5905208 10.3099959 -46.7269139
C 8.9312833 11.6033541 -47.0463739
H 8.7099762 12.6632397 -47.1442538
H 8.3393666 11.1827496 -46.2344072
H 8.6361123 11.1131843 -47.9758533
O 11.0895781 11.8306891 -47.9597574
H 10.484578 12.3844576 -48.4701324
N 10.2252136 14.371186 -45.663101
H 9.3074535 13.9722939 -45.5446904
C 10.2857366 15.8236971 -45.8547703
H 10.7235008 16.0265557 -46.8268567
C 11.1106349 16.537419 -44.7650356
O 11.8863183 17.4526382 -45.0535196
C 8.8657689 16.3970441 -45.8856242
H 8.8986116 17.4030429 -46.3069571
H 8.4867539 16.4648006 -44.8668808
S 7.7163401 15.3759861 -46.8510702
H 8.4533831 15.1086649 -47.9512639
N 10.9632892 16.0999518 -43.508704
H 10.2825801 15.3646959 -43.3383485
C 11.7666912 16.5933009 -42.3777083
H 11.7210304 17.682167 -42.3558897
C 13.2337723 16.2149077 -42.5646649
O 14.1041332 17.0794444 -42.4631432
C 11.2181531 16.0596538 -41.0385742
H 11.0788588 14.9803609 -41.1088303
C 12.1583111 16.3454275 -39.8608403
H 12.413162 17.4044263 -39.8365978
H 13.0713173 15.7583224 -39.9544092
H 11.6747709 16.0658247 -38.9240883
C 9.8619149 16.7101419 -40.7311641
H 9.9738266 17.7906836 -40.6408162
H 9.465485 16.313601 -39.7953368
H 9.147258 16.4904463 -41.5236473
N 13.5086787 14.9554922 -42.9189931
H 12.7374487 14.300883 -43.0121126
C 14.8616324 14.4600437 -43.1763215
H 15.4605874 14.5795315 -42.2714439
C 15.5717593 15.257738 -44.2626702

O 16.6984701 15.6980688 -44.0520232
C 14.8119003 12.9696725 -43.5454865
H 15.47017 12.778969 -44.3885111
H 13.8159203 12.6800994 -43.8734669
C 15.2350372 12.1081566 -42.3887418
N 16.5503927 11.9810507 -41.9354251
C 16.4753185 11.2196844 -40.8299497
H 17.3253826 10.9192111 -40.2306378
N 15.2029599 10.8627996 -40.5836198
H 14.890693 10.2906702 -39.8085624
C 14.4037633 11.4233475 -41.5538219
H 13.3257702 11.3738953 -41.6274226
N 14.9078435 15.5075468 -45.387301
H 13.989292 15.091668 -45.5152898
C 15.5112271 16.2350314 -46.5011762
H 16.4944499 15.8005517 -46.6810838
C 15.788866 17.702243 -46.1455768
O 16.9395239 18.1374501 -46.2012692
C 14.6897345 16.0179734 -47.7882799
H 15.1327128 16.6236197 -48.5603665
C 14.7908711 14.5234165 -48.182398
H 14.1962195 13.8987777 -47.5198436
H 15.8220997 14.1781548 -48.1262874
H 14.4372692 14.3716629 -49.2011021
C 13.2366266 16.4975189 -47.7320752
H 12.7646062 16.0195718 -46.8976035
H 13.2059957 17.5711979 -47.5585925
C 12.4407379 16.1837724 -49.0033466
H 12.2244151 15.1164961 -49.062049
H 13.0189562 16.48687 -49.8741816
H 11.4970485 16.7230964 -48.9888032
N 14.8187234 18.4245126 -45.5793613
H 13.9008259 18.0120409 -45.4473034
C 15.0314858 19.8172963 -45.1857533
H 15.4248899 20.3646793 -46.0422485
C 16.0654803 19.9815299 -44.052507
O 16.775709 20.9872676 -44.0197683
C 13.6739593 20.4215669 -44.8288207
H 13.2487701 19.8884107 -43.9775321
H 12.9997992 20.3346726 -45.682005
H 13.7950387 21.4777533 -44.588073
N 16.1945128 19.0031707 -43.1417661
H 15.5550477 18.2125874 -43.1960906
C 17.1426889 19.0652184 -42.0165262
H 17.2682905 20.115006 -41.7540704
C 18.5555244 18.5806105 -42.3633946

O 19.5022617 19.2022987 -41.8920977
C 16.5356863 18.3408313 -40.8014493
H 15.5546198 18.7709715 -40.5929376
H 16.3868088 17.2899072 -41.0560764
C 17.3783728 18.4041283 -39.5137768
H 18.2761985 17.8007418 -39.655826
H 16.803368 17.9420041 -38.7110996
C 17.8159365 19.8120997 -39.0658109
H 18.4907804 20.2290409 -39.816017
H 18.3932875 19.7217441 -38.1440225
N 16.6897875 20.7491832 -38.8609668
H 16.5665419 21.4502419 -39.5716769
C 15.8807065 20.8193544 -37.8169131
N 14.9853566 21.7589121 -37.7435133
H 14.3645694 21.8061651 -36.956886
H 14.8726153 22.4170928 -38.4946404
N 15.9472023 19.9843426 -36.8189475
H 16.6241288 19.2455779 -36.8502505
H 15.2949912 20.0477172 -36.0589826
N 18.7300829 17.5290883 -43.1764286
H 17.9068943 17.0488226 -43.5306661
C 20.0684893 17.0390531 -43.5746573
H 20.7065707 16.9291063 -42.6964889
C 20.7865705 18.0002679 -44.5366372
O 22.0134416 18.0053269 -44.5586972
C 19.9813346 15.699411 -44.3219547
H 19.304658 15.8747004 -45.1616404
H 20.9671976 15.5209637 -44.7569981
C 19.5628823 14.3578569 -43.6784448
H 20.3290906 14.0366348 -42.971993
H 18.6129878 14.4437749 -43.1562454
C 19.4543242 13.3380914 -44.844005
H 20.418487 13.3271226 -45.354375
H 18.7167883 13.694725 -45.5669937
C 19.1362141 11.8730891 -44.4980398
H 19.490443 11.6480878 -43.4892399
H 19.686472 11.2395421 -45.2001729
N 17.6955314 11.5486037 -44.6343123
H 17.1272324 12.0169392 -43.9401613
H 17.4806865 10.5531329 -44.5600195
H 17.3202713 11.7308229 -45.5705452
N 20.0478271 18.6624719 -45.4364741
H 19.046923 18.5093921 -45.436498
C 20.6358853 19.3309775 -46.6085273
H 21.6749389 19.5607893 -46.3684505
C 20.0541746 20.7091617 -46.9622581

O 20.3152965 21.2210728 -48.0515675
C 20.6868561 18.354292 -47.7957786
H 21.0473326 18.879063 -48.6814314
H 21.4566581 17.6177888 -47.5613333
C 19.4373287 17.602644 -48.1667651
C 19.394892 16.1940631 -48.1271936
H 20.196105 15.6434021 -47.6560693
C 18.3451897 15.5113149 -48.7728262
H 18.2923324 14.4372359 -48.7484014
C 17.3896775 16.2497781 -49.5044677
O 16.5031357 15.6745726 -50.3589183
H 16.8175994 14.8393012 -50.723428
C 17.3695605 17.6457992 -49.4114359
H 16.6398896 18.1806343 -49.9827419
C 18.3848271 18.32481 -48.7295873
H 18.4231516 19.3990316 -48.7319403
N 19.3517895 21.3539171 -46.024161
H 19.1651048 20.8775033 -45.1539639
C 19.0384741 22.7911583 -46.0750765
H 18.3539854 22.9771147 -45.2457682
C 18.2660689 23.2374827 -47.3416898
O 18.3498463 24.3844502 -47.7803859
C 20.3254224 23.5929801 -45.7682459
H 21.0190659 23.4837652 -46.6030665
H 20.8007875 23.1682607 -44.8823023
C 20.0831759 25.0814136 -45.500918
O 19.0655738 25.4065578 -44.8420731
O 20.8655128 25.9146396 -46.0124582
N 17.4548563 22.3556176 -47.9295338
H 17.4238769 21.424189 -47.5465261
C 16.4823397 22.7281121 -48.9710723
H 16.9464183 23.4477338 -49.6473765
C 15.24281 23.3977265 -48.3725054
O 14.9522023 23.2457856 -47.1836795
C 16.0422905 21.5162724 -49.8130752
H 15.2603537 21.8499514 -50.4920367
C 17.2011516 21.0558416 -50.7125561
H 18.1143336 20.9147098 -50.1404907
H 17.4026366 21.8357731 -51.4439333
H 16.943333 20.1436236 -51.2459316
C 15.398919 20.4182088 -48.9405732
H 16.1665696 19.8238888 -48.4571155
H 14.7955406 20.8598812 -48.151009
C 14.4493492 19.5350595 -49.7478417
H 14.8862927 19.2531794 -50.7036183
H 13.5268763 20.0795328 -49.9194258

H 14.2201392 18.6408149 -49.1834476
N 14.4360211 24.010578 -49.2375354
H 14.7402083 24.0446147 -50.2085445
C 12.9993631 24.158699 -49.0076405
H 12.8175786 24.2573771 -47.9397338
C 12.2662528 22.9046082 -49.5103378
O 12.6107608 22.3634176 -50.5604784
C 12.5234879 25.4517901 -49.6859483
H 13.0406288 26.283309 -49.2048236
H 12.830272 25.44303 -50.731898
C 11.0112581 25.7338392 -49.6079951
H 10.8575365 26.7986213 -49.7828324
H 10.658697 25.5126149 -48.6002629
C 10.1424015 24.9839405 -50.6247752
O 10.5934761 24.3664138 -51.5757024
N 8.8434225 24.9954737 -50.4307784
H 8.2650384 24.4828781 -51.0906918
H 8.4331033 25.5005896 -49.6701504
N 11.2359635 22.4621827 -48.7844512
H 11.0578115 22.9049529 -47.8891113
C 10.3429926 21.383246 -49.214284
H 10.5949631 21.0920705 -50.235015
C 8.8711568 21.8264889 -49.2272665
O 8.3637704 22.3460571 -48.2286926
C 10.5732244 20.1618264 -48.309774
H 10.3332883 20.4190762 -47.2777976
H 11.6227969 19.8672222 -48.3609254
S 9.5400098 18.7650291 -48.8429061
H 8.3402798 19.3259352 -48.6493615
N 8.1565084 21.4877271 -50.299685
H 8.6580875 21.1050509 -51.0997332
C 6.6915622 21.4723115 -50.3796701
H 6.2622286 21.8728319 -49.4665579
C 6.2201094 20.031677 -50.5303368
O 6.4399128 19.4158451 -51.5664035
C 6.1943466 22.3420754 -51.5434893
H 6.7074003 22.060723 -52.4640156
C 4.6826819 22.2560971 -51.7615391
H 4.1511206 22.4652032 -50.8329504
H 4.408085 21.2625488 -52.117419
H 4.3832472 22.9787312 -52.5207642
O 6.4597851 23.693421 -51.2481149
H 6.0035097 23.908674 -50.423727
N 5.5750189 19.4858299 -49.5003679
H 5.4489048 20.0421054 -48.6609263
C 4.8934822 18.1915638 -49.5707935

H 5.3805521 17.5583986 -50.3075649
H 4.9561912 17.6920084 -48.6044006
C 3.4178491 18.3381219 -49.9451647
O 2.7499778 19.2457229 -49.4508098
N 2.8759735 17.4192718 -50.7447628
H 3.489359 16.728848 -51.172119
C 1.4218424 17.2768095 -50.9304672
H 0.9352328 17.8212694 -50.1261785
C 0.9560668 15.8305307 -50.7697592
O 1.6406314 14.8966804 -51.1867693
C 0.9136146 17.8905378 -52.2565359
H -0.1448096 17.6360649 -52.3435319
C 1.6387051 17.3248981 -53.5004349
H 2.6843131 17.6363338 -53.4916327
H 1.6106378 16.2363657 -53.4623999
C 0.9961788 19.4249524 -52.1858548
H 2.0365676 19.7521869 -52.1926341
H 0.5247676 19.7783184 -51.266841
H 0.4642133 19.8755679 -53.0218273
C 1.0052803 17.7519525 -54.8316522
H 1.1088672 18.8260369 -54.9781292
H -0.0515642 17.481976 -54.8436029
H 1.510511 17.2397663 -55.65089
N -0.2477491 15.6495421 -50.2260329
H -0.7465903 16.4668413 -49.8863459
C -0.9656272 14.3704334 -50.212057
H -0.6470092 13.784003 -51.0743246
C -2.4736961 14.6046495 -50.3361702
O -2.9923013 15.6108318 -49.855645
C -0.6345655 13.5626088 -48.9469876
H -1.035592 14.063649 -48.0667914
H 0.4483293 13.4718768 -48.8486547
O -1.2039403 12.2647033 -49.0661658
H -0.8230701 11.6633302 -48.3915009
N -3.189996 13.6624306 -50.9545782
H -2.693636 12.8593069 -51.3085801
C -4.6570956 13.7038033 -51.1109514
H -4.9453094 14.6681594 -51.5324737
C -5.3659256 13.5807981 -49.7505522
O -6.4750307 14.0756237 -49.5707605
C -5.0838402 12.5810381 -52.0946599
H -4.5868338 11.656628 -51.7934535
C -6.60297 12.3174183 -52.0927149
H -7.1427278 13.2367823 -52.3255887
H -6.9278242 11.951214 -51.1188279
H -6.8636599 11.5560362 -52.8261546

C -4.6265523 12.9470809 -53.5247128
H -5.2699191 13.744181 -53.8956398
H -3.6030739 13.3215718 -53.5000957
C -4.6555746 11.788384 -54.5300713
H -5.6777838 11.458972 -54.7113472
H -4.0625365 10.9539342 -54.1543668
H -4.2321241 12.1260136 -55.4769672
N -4.7271601 12.913471 -48.788543
H -3.775143 12.6272275 -48.9723444
C -5.2999645 12.5558781 -47.4919011
H -6.3581933 12.3426238 -47.6238231
C -5.12327 13.6827605 -46.4548777
O -3.9915082 13.9538372 -46.0403971
C -4.6452439 11.2446981 -47.0565748
H -3.587913 11.2809028 -47.3174804
H -5.1004324 10.4159779 -47.6001787
O -4.7366918 11.0128571 -45.6723862
H -5.6758702 11.0025864 -45.3613888
N -6.2181431 14.2734505 -45.9265974
C -6.1448261 15.2535177 -44.8392723
H -5.5037262 16.0768951 -45.1469994
C -5.5994107 14.6593758 -43.5365247
O -4.990551 15.3639187 -42.7414758
C -7.5767455 15.7683423 -44.6357585
H -8.056633 15.2366621 -43.8112928
H -7.5919532 16.8425494 -44.4489233
C -7.6005661 14.1249832 -46.3585443
H -7.6892693 14.0050037 -47.4364082
H -8.051605 13.2737373 -45.845865
C -8.2932888 15.4153499 -45.9362787
H -8.111043 16.1900229 -46.680453
H -9.3633916 15.2698172 -45.7853592
N -5.798928 13.3603836 -43.3244477
H -6.3334307 12.8472974 -44.0199305
C -5.3819279 12.5909009 -42.1533687
H -5.5086175 13.2194111 -41.2710266
C -3.889537 12.1953296 -42.1819728
O -3.2267231 12.2061379 -41.1417984
C -6.3412377 11.3905832 -41.9922851
H -7.3113502 11.7703061 -41.6675729
H -5.9575394 10.7498656 -41.1971473
C -6.5663179 10.5388814 -43.2538189
O -6.908747 11.078591 -44.3329274
O -6.3508923 9.3107568 -43.1812859
N -3.3008267 11.9683216 -43.3619274
H -3.8866016 11.9470974 -44.1933319

C -1.8430178 11.9831623 -43.5618261
H -1.3723892 11.257173 -42.9150736
C -1.2618897 13.3502526 -43.1982805
O -0.4067809 13.4546671 -42.3135894
C -1.4776396 11.6187214 -45.0103698
H -2.1218696 12.1563712 -45.7031098
H -0.4492902 11.9202012 -45.2048059
C -1.5826308 10.1109484 -45.2530965
H -2.542401 9.7508069 -44.8866851
H -0.8029225 9.6052696 -44.6769365
C -1.4265517 9.7523301 -46.7342513
O -2.1887154 8.9014817 -47.2378949
O -0.4387212 10.1870761 -47.3704002
N -1.8238071 14.3981459 -43.7998905
H -2.5652821 14.2183388 -44.4716944
C -1.3629664 15.7743681 -43.644951
H -0.2966211 15.7971953 -43.8619811
C -1.5011144 16.2671664 -42.1931932
O -0.5669668 16.8774303 -41.6842395
C -2.0937697 16.6432986 -44.6956286
H -3.1616424 16.4331521 -44.6208048
C -1.6453572 16.3062192 -46.1415492
H -1.8060816 15.2459139 -46.3228022
H -2.2857106 16.8442621 -46.8391984
C -1.9169274 18.1412204 -44.4519846
H -0.8624135 18.3648704 -44.4776171
H -2.351247 18.4235725 -43.4927015
H -2.4173335 18.7036014 -45.2397571
C -0.1850993 16.6247081 -46.5047295
H 0.502714 16.0852862 -45.8550906
H 0.005957 17.6948585 -46.4390428
H -0.0012286 16.3148047 -47.5317343
N -2.5671139 15.9256739 -41.4689525
H -3.3267024 15.4447242 -41.9411177
C -2.7735512 16.3191514 -40.0675664
H -2.7786111 17.4067291 -40.0129945
C -1.6436206 15.8404344 -39.1348382
O -1.1884872 16.6049745 -38.2795479
C -4.1712491 15.8244989 -39.6523631
H -4.8964345 16.3669418 -40.2600162
H -4.276831 14.7663523 -39.8970248
C -4.5547229 16.0502673 -38.1793378
H -5.6347657 16.1922723 -38.1337433
H -4.0852274 16.9595111 -37.8033673
C -4.2106249 14.87867 -37.2602295
O -3.478791 13.9624486 -37.5900405

N -4.7524033 14.838826 -36.0651956
H -4.5608134 14.0108061 -35.5281391
H -5.3835633 15.5549238 -35.7602172
N -1.1004638 14.6424705 -39.3708469
H -1.5013084 14.0764096 -40.106404
C 0.0418472 14.1322373 -38.6116889
H -0.0436926 14.4501244 -37.5713118
C 1.3850099 14.6518156 -39.1239408
O 2.2660308 14.8908551 -38.3035319
C 0.0435491 12.6104591 -38.6527647
H 1.0465421 12.2488474 -38.4138007
H -0.2262749 12.3017169 -39.6598596
S -1.1117245 11.9445062 -37.4241918
H -2.0986728 12.845829 -37.6105998
N 1.5575739 14.8170369 -40.4417058
H 0.8054631 14.5690666 -41.0762493
C 2.7701594 15.422793 -40.987119
H 3.6444812 14.9045317 -40.5883586
C 2.8696392 16.8863013 -40.5636503
O 3.5847836 17.2487937 -39.6283336
C 2.7907183 15.3258836 -42.5238653
H 1.9156234 15.7985274 -42.9702768
H 2.7822393 14.2994419 -42.8465516
H 3.7036186 15.7788831 -42.9060338
N 2.1002966 17.7180647 -41.2791163
H 1.1970245 17.3057366 -41.4945638
C 2.6117307 18.5517644 -42.3861192
H 3.1341734 17.8723516 -43.0516016
C 1.3970127 19.0600589 -43.2242848
H 1.358215 18.4262558 -44.0974844
H 0.5018309 18.6692998 -42.7733033
C 1.1166488 20.5727843 -43.5444475
H 1.2915895 21.151315 -42.6635274
H 1.8411256 20.9728381 -44.2381746
C -0.3230066 20.9360951 -43.9914732
H -1.0227159 20.3720331 -43.3859128
H -0.4699022 20.6178342 -45.0179328
C -0.6963571 22.4298742 -43.8025919
H 0.1995555 22.9755746 -43.4898631
H -1.4260614 22.4979107 -42.9928553
N -1.2635441 23.0865246 -45.012343
H -0.5895041 23.0906335 -45.7804691
H -1.4992876 24.0597524 -44.8902245
H -2.0834365 22.6400856 -45.4285437
C 3.7642777 19.4606364 -41.9383207
O 4.6934606 18.9814363 -41.3095185

N 3.6311882 20.7771193 -41.9989438
H 2.8876535 21.1417414 -42.5604266
C 4.0788258 21.6000937 -40.8865894
H 5.1237588 21.3834285 -40.6673247
C 3.9999911 23.0820359 -41.3327063
H 3.0493725 23.2473995 -41.8409777
H 4.7944901 23.2717171 -42.0553756
C 4.1026739 24.1179475 -40.2399803
N 3.0918861 25.0331692 -39.9409579
C 3.4223691 25.5589047 -38.7527186
H 2.8010558 26.2448663 -38.192991
N 4.5752577 25.0358408 -38.3042514
H 4.8930887 25.1113105 -37.3429746
C 5.0364284 24.1390869 -39.2428601
H 5.8685618 23.4582498 -39.1352582
C 3.2420288 21.199852 -39.6385361
O 3.0797663 22.025223 -38.7500341
N 2.530232 20.0383135 -39.640792
H 2.865367 19.2416506 -40.1623869
C 1.4823966 19.8353562 -38.7041374
H 1.2481924 20.7922532 -38.2384218
C 2.0858318 19.0933861 -37.5361898
O 3.067777 19.6165891 -37.0050026
C 0.1413169 19.4928001 -39.3442083
H 0.1442248 18.4588372 -39.6817178
H -0.6141341 19.569777 -38.5611991
C -0.267998 20.4123699 -40.5175301
H 0.1183068 19.9509151 -41.3678312
C -1.7776922 20.4353344 -40.70414
H -2.2492346 20.8376265 -39.8066394
H -2.1313518 19.4171078 -40.8620015
H -2.0806715 21.041301 -41.5528518
C 0.1944014 21.8747087 -40.5369619
H 1.2344464 22.003532 -40.7787416
H 0.035205 22.3050513 -39.5470494
H -0.3843236 22.4742456 -41.2283432
N 1.4878269 18.020571 -37.0275607
H 0.680745 17.619781 -37.490007
C 1.8370793 17.567107 -35.682473
H 1.6480608 18.3923588 -34.994206
C 3.3255548 17.1909504 -35.5105984
O 3.975249 17.6990186 -34.5966676
C 0.897452 16.4265003 -35.2938252
H 1.0844826 15.5580817 -35.9233497
H -0.1416476 16.7397962 -35.4098183
H 1.0723946 16.150676 -34.2533891

N 3.895938 16.3935091 -36.4204664
H 3.3273487 16.0340792 -37.1822979
C 5.2891756 15.9425756 -36.3358269
H 5.4806631 15.6036171 -35.3165186
C 6.3276383 17.0397494 -36.6405866
O 7.4910534 16.9063308 -36.2670223
C 5.4618095 14.744717 -37.274927
H 5.2733635 15.0489023 -38.3063018
H 4.7617187 13.9547081 -37.0015401
H 6.47971 14.3605827 -37.1961688
N 5.9285687 18.1328717 -37.2982429
H 4.962632 18.1795496 -37.6016175
C 6.8314934 19.2418052 -37.635999
H 7.8429605 18.8505207 -37.6832907
C 6.844892 20.3829303 -36.6121256
O 7.5699517 21.3645417 -36.8076774
C 6.4883151 19.7654549 -39.0249614
H 5.4763035 20.1549587 -38.9918722
H 6.5452826 18.940506 -39.7349417
O 7.3909096 20.7802827 -39.4271918
H 7.5897174 21.3108912 -38.6363837
N 6.0301942 20.3258281 -35.5583977
H 5.4633655 19.4990513 -35.4131305
C 5.9102691 21.4436743 -34.6242259
H 5.8569889 22.3420848 -35.2375565
C 7.1639527 21.593333 -33.7492034
O 7.6308825 20.6298769 -33.149975
C 4.6035516 21.3149437 -33.8160339
H 3.9610644 20.5586151 -34.2705869
H 4.8261419 20.983383 -32.8007128
C 3.8146552 22.6339158 -33.7630863
H 4.3761121 23.361526 -33.1761685
H 2.8597389 22.454182 -33.2696363
C 3.5503843 23.2164719 -35.1512038
O 4.0029425 24.3035214 -35.4804174
N 2.9488639 22.4629638 -36.0411139
H 2.950617 22.7735293 -37.0102779
H 2.7918841 21.4817724 -35.8719266
N 7.7210904 22.8081187 -33.6914249
H 7.2987774 23.5567177 -34.2190106
C 8.9972279 23.0788989 -33.0131334
H 9.0701395 24.1482844 -32.8145984
H 9.0214978 22.5526099 -32.0586359
C 10.2493908 22.6746477 -33.805537
O 11.3286693 22.5814544 -33.2268775
N 10.1271567 22.3925806 -35.1093067

H 9.2142375 22.4390954 -35.5407744
C 11.2828804 22.1739636 -35.9765919
H 12.1470555 21.9182832 -35.3639204
C 11.6311631 23.4425166 -36.7703543
O 10.8468171 23.8739616 -37.6062429
C 11.0248787 21.0051895 -36.9419587
H 10.2282802 21.2982624 -37.627223
H 11.9359448 20.8776523 -37.5163873
C 10.6521745 19.6372191 -36.3484215
H 9.6886744 19.7031776 -35.8480046
C 10.554009 18.6256642 -37.4922608
H 11.5421073 18.4392663 -37.908598
H 9.9033941 19.0025286 -38.2786216
H 10.1402736 17.6890333 -37.1141196
C 11.700169 19.1190278 -35.363519
H 12.6761902 19.0615675 -35.8409176
H 11.4049478 18.1266817 -35.0192001
H 11.7434391 19.7788958 -34.4960605
N 12.8948299 23.8661966 -36.7091582
H 13.3352564 23.7065076 -35.812184
C 13.4155394 25.0179326 -37.4790076
H 12.6681205 25.8145446 -37.4282954
C 13.6732908 24.7387133 -38.9836209
O 14.3679382 25.4938641 -39.6582383
C 14.7076514 25.5493859 -36.8171534
H 15.5330047 24.8870948 -37.0856383
H 14.9254605 26.5347873 -37.2302733
C 14.686988 25.6625318 -35.2845744
H 15.5866248 26.1902592 -34.9596257
H 14.7320807 24.6561241 -34.8586402
C 13.4378158 26.3886491 -34.7758366
O 13.4280188 27.6368107 -34.819975
O 12.4900572 25.6633847 -34.4019596
N 13.2648524 23.5772005 -39.5105643
H 12.5354699 23.085907 -39.0068225
C 13.5570177 23.1795007 -40.8976494
H 14.5254876 23.6009869 -41.1761233
C 12.542468 23.7627457 -41.8860466
O 11.3445727 23.7642826 -41.6133993
C 13.6717175 21.6505914 -41.0511484
H 14.6737933 21.3409764 -40.7633607
H 13.5409867 21.3814689 -42.0977122
C 12.6807254 20.8409029 -40.234541
O 13.0819143 19.9571612 -39.4998137
N 11.4063889 21.1652132 -40.2593715
H 10.7663929 20.6171536 -39.7086994

H 11.0835354 21.9909526 -40.7547637
N 13.0133136 24.1123106 -43.0888082
H 14.003369 23.9861169 -43.252692
C 12.2559559 24.8063065 -44.14478
H 11.634147 25.5703974 -43.6725806
C 11.2900693 23.8636724 -44.8836361
O 11.4794884 23.5296958 -46.0528063
C 13.2402964 25.5207442 -45.0935189
H 13.7974318 24.7585126 -45.6299273
H 12.6747639 26.1075278 -45.8195301
C 14.2367 26.4457212 -44.363787
H 13.6782029 27.2076148 -43.8182901
H 14.8081668 25.8651489 -43.6389009
C 15.2530414 27.1445263 -45.2802328
H 15.9662655 27.6665972 -44.6388995
H 14.7410762 27.8844194 -45.8974409
C 16.0017683 26.1590706 -46.1895801
H 15.4383225 26.0365783 -47.1185131
H 16.0485386 25.1791219 -45.7028254
N 17.3833513 26.6013149 -46.4784672
H 17.4501689 27.5257558 -46.8646949
H 17.8394313 25.9286781 -47.0934372
H 17.9672793 26.5200239 -45.6378488
N 10.2586788 23.4106932 -44.1767312
H 10.1841574 23.7178249 -43.2124954
C 9.2046284 22.5279462 -44.6902692
H 9.4646982 22.2318761 -45.7066837
C 7.8664666 23.2561846 -44.773848
O 7.5173305 24.0732073 -43.9268036
C 9.0728533 21.2239852 -43.8778855
H 8.316634 20.6091786 -44.3642886
C 10.3766276 20.4230278 -43.8853184
H 11.1563743 20.9672999 -43.3545985
H 10.6901088 20.2389319 -44.9118237
H 10.2204614 19.4614051 -43.3955968
C 8.6444678 21.4437578 -42.4225151
H 9.3296493 22.1190396 -41.9159669
H 8.6253233 20.491972 -41.8909169
H 7.6444986 21.8751438 -42.3818422
N 7.0848025 22.9020958 -45.7835561
H 7.4542566 22.2468272 -46.4660422
C 5.688535 23.2901281 -45.9603944
H 5.2592379 23.5642908 -44.9971347
C 4.9368154 22.090787 -46.5162855
O 5.5175932 21.2799915 -47.2402207
C 5.572196 24.4861637 -46.9093264

H 6.1315988 25.3254024 -46.4927753
H 4.5253632 24.7775338 -47.0057321
O 6.0920421 24.166681 -48.1936715
H 6.9019288 23.6412406 -48.069159
N 3.6649475 21.9328174 -46.1572492
H 3.1924113 22.6509743 -45.6290061
C 2.8540294 20.891737 -46.7718369
H 3.2603949 20.7420939 -47.768523
C 1.4232672 21.3469321 -46.9993605
O 0.9655742 22.2461308 -46.2958749
C 2.8723023 19.5311039 -46.0727033
H 1.8998189 19.3593649 -45.6756641
H 2.831625 18.8431272 -46.9167144
C 4.0238134 18.9905664 -45.2169582
C 4.3071869 17.6253821 -45.3860567
H 3.6888653 17.0224533 -46.0309537
C 4.8919789 19.744078 -44.3979068
H 4.7530479 20.7996534 -44.2590226
C 5.3992881 17.0235059 -44.752768
H 5.5957283 15.9801922 -44.9285487
C 5.9619852 19.1264943 -43.7177794
H 6.5828412 19.700868 -43.0462872
C 6.2154413 17.761582 -43.8888187
H 7.0295482 17.2877361 -43.3583586
N 0.7049211 20.6843602 -47.9071269
H 1.1580329 19.9917536 -48.4999263
C -0.7426341 20.8387991 -48.0115702
H -1.0824204 20.9641876 -46.9898499
C -1.5361428 19.617803 -48.4852348
O -0.9924186 18.6595985 -49.0384563
C -1.11455 22.1121096 -48.8003637
H -1.7026334 21.8618073 -49.6844732
H -0.2187602 22.6483505 -49.119513
C -1.9431406 22.9948283 -47.8556361
O -2.8487189 22.4398679 -47.1824651
O -1.4945848 24.1167787 -47.540539
N -2.8489941 19.6723543 -48.2388324
H -3.2118883 20.5386921 -47.8451067
C -3.8093695 18.7281656 -48.8164363
H -3.3973647 17.7249245 -48.7310293
C -3.9734597 19.0752603 -50.2936677
O -4.3973474 20.175993 -50.6415666
C -5.1732061 18.7435119 -48.0992768
H -5.6730347 19.6950951 -48.2825975
C -6.0637773 17.6068255 -48.6193746
H -5.6052813 16.639463 -48.4108445

H -6.2151491 17.7031978 -49.6940242
H -7.0395154 17.6576898 -48.1394967
C -5.0359728 18.5600012 -46.5826229
H -4.4763312 17.6507279 -46.373113
H -6.0235974 18.4938287 -46.1264681
H -4.5200769 19.4177832 -46.1531065
N -3.6215795 18.1436875 -51.1741785
H -3.2879382 17.2507786 -50.8282856
C -3.6115106 18.3903012 -52.6077545
H -4.5005105 18.9612831 -52.879691
H -2.7342712 18.9845956 -52.864576
C -3.5764643 17.1062873 -53.4192378
O -2.760003 16.2192524 -53.1788795
N -4.4347018 17.0516582 -54.4326559
H -5.0732818 17.8204822 -54.5581563
C -4.3864658 16.05468 -55.4956002
H -4.0915978 15.0912157 -55.0883004
C -3.3390088 16.4662952 -56.5310674
O -3.4495113 17.5140222 -57.164842
C -5.7890218 15.9175663 -56.0942179
H -6.1264015 16.9003061 -56.4288134
H -6.4684305 15.5787826 -55.3090581
C -5.884318 14.9468145 -57.2730016
O -4.8383242 14.4918336 -57.7921059
O -7.0322555 14.7422459 -57.7062923
N -2.3237965 15.6294866 -56.7222922
H -2.3348741 14.7515165 -56.2290766
C -1.1959614 15.917138 -57.5968154
H -0.8042434 16.9026035 -57.3349828
C -1.5477935 15.9520276 -59.0940706
O -0.7986989 16.5414193 -59.8719076
C -0.1261968 14.8713849 -57.2991538
H -0.4332321 13.8964363 -57.6832249
H 0.0211865 14.8034629 -56.2217764
H 0.8044678 15.171372 -57.7763393
N -2.6894224 15.3899282 -59.5099429
H -3.3057519 14.9434825 -58.8268714
C -3.1680469 15.5081525 -60.8889374
H -2.2977069 15.4764396 -61.5426236
C -3.8714604 16.8578481 -61.1353749
O -3.8881519 17.3427345 -62.2717246
C -4.0589673 14.2899356 -61.2019821
H -3.5800509 13.3944349 -60.8022451
H -5.0094949 14.4145144 -60.6792547
C -4.3336983 14.054691 -62.7013104
H -4.8066073 14.9365606 -63.1323418

C -3.0607759 13.732287 -63.4923323
H -2.5395826 12.8897156 -63.037984
H -2.3987076 14.5952794 -63.5160859
H -3.3198034 13.4669686 -64.5171777
C -5.2831101 12.8667808 -62.868187
H -4.8269732 11.9632398 -62.4652122
H -5.5106869 12.7195113 -63.9246257
H -6.2135557 13.0676634 -62.3361046
N -4.3438173 17.5317856 -60.0767708
H -4.239743 17.0913676 -59.1689351
C -5.1392953 18.7670931 -60.1508346
H -5.3101043 19.014173 -61.1954728
C -4.4652452 20.0192241 -59.553186
O -4.7247452 21.1197762 -60.036646
C -6.5275032 18.4864972 -59.5562995
H -7.1041324 19.4099779 -59.5149897
H -6.4332717 18.0886791 -58.546576
C -7.2894454 17.5033969 -60.4354308
O -7.5563357 17.7986585 -61.5883171
N -7.5704357 16.2932746 -60.0008698
H -8.0536807 15.6856149 -60.632247
H -7.3300548 15.9419432 -59.0695682
N -3.5291447 19.8666245 -58.6129897
H -3.3778296 18.9328279 -58.2480178
C -2.7161973 20.948246 -58.0426512
H -3.3974747 21.6988151 -57.6398965
C -1.8381347 21.6244684 -59.108357
O -1.3603305 20.9679296 -60.0376461
C -1.8708616 20.3801509 -56.8857777
H -1.2155043 19.5960797 -57.2688558
H -2.5403692 19.9339514 -56.1488536
C -1.0113161 21.4298223 -56.1713507
H -0.2443703 21.7912918 -56.8567282
H -0.5059511 20.9491715 -55.3327881
S -1.9402459 22.8506813 -55.5394919
C -0.5701828 23.8292679 -54.8690719
H -0.9613234 24.74235 -54.4197375
H 0.1193026 24.0926016 -55.6715556
H -0.0410525 23.252321 -54.1098706
N -1.6015408 22.9369568 -58.9811233
H -1.9423219 23.4054305 -58.1486339
C -0.8192637 23.7390643 -59.9395053
H -0.2576772 23.064157 -60.5851844
C 0.2282464 24.5957342 -59.2270807
O 0.004776 25.0706294 -58.1183271
C -1.7509353 24.6002576 -60.822006

H -2.2485006 25.3361505 -60.1875853
H -1.1470927 25.1432575 -61.5512182
C -2.8355023 23.8041089 -61.576147
H -3.4692309 24.5095959 -62.1148217
H -3.4670243 23.293783 -60.85025
C -2.285634 22.7794283 -62.5838831
H -1.469829 22.2166229 -62.1362081
H -1.8727529 23.3160784 -63.440655
N -3.3480238 21.8600974 -63.0454371
H -4.1592275 22.2924263 -63.4494333
C -3.3712553 20.539867 -62.9367282
N -4.3632524 19.8552986 -63.436589
H -4.394121 18.8635073 -63.2050823
H -5.2093213 20.3214733 -63.7056537
N -2.4470361 19.8412767 -62.3456859
H -1.8072108 20.3142319 -61.7116381
H -2.5589807 18.8412342 -62.2636225
N 1.3477479 24.8147152 -59.9086339
H 1.3980397 24.4714973 -60.8561952
C 2.4801481 25.6382775 -59.4817666
H 2.171737 26.310206 -58.6796175
C 2.9540436 26.4820188 -60.6742369
O 2.5493109 26.2124622 -61.8048482
C 3.6101897 24.7291168 -58.9715134
H 3.9556652 24.0994097 -59.7921348
H 4.4519802 25.3525014 -58.6710504
C 3.2414508 23.8502424 -57.790578
C 3.2255769 24.3988016 -56.4940977
H 3.4578802 25.4439975 -56.3432923
C 2.944317 22.484486 -57.9805326
H 2.957988 22.0603603 -58.9749413
C 2.9118273 23.5871558 -55.3886793
H 2.893409 23.9989979 -54.390721
C 2.6376701 21.6672874 -56.8746023
H 2.4174114 20.6212776 -57.0198648
C 2.6196309 22.2218223 -55.5761608
O 2.3179465 21.4526177 -54.4997302
H 2.18435 20.5319836 -54.724826
N 3.8334992 27.4628261 -60.4509498
H 4.2082822 27.584594 -59.5219213
C 4.4939213 28.1620494 -61.5616058
H 3.7309422 28.5488971 -62.2384471
C 5.3882736 27.2020676 -62.3485732
O 6.0176283 26.3114741 -61.7616727
C 5.3217809 29.3477141 -61.0436929
H 6.091829 28.9980095 -60.3569351

H 4.6612574 30.029898 -60.5071673
O 5.9393037 30.0538829 -62.1032955
H 6.7243398 29.5613144 -62.4093461
N 5.5820288 27.4940815 -63.6317522
H 5.0106585 28.1958685 -64.0746187
C 6.7307802 26.9874338 -64.3760478
H 6.6366855 25.9085016 -64.4254029
C 8.028407 27.3558119 -63.6304401
O 8.1187891 28.4400932 -63.0417593
C 6.7479946 27.5450176 -65.8100117
H 7.719706 27.3149662 -66.2505881
H 6.6485008 28.6315395 -65.7731873
C 5.6672195 26.9697377 -66.7358325
O 4.772257 26.2412801 -66.2508656
O 5.7522914 27.2543315 -67.9504335
N 9.0046514 26.4438149 -63.5827792
H 8.8767 25.5757138 -64.0999271
C 10.2928452 26.6834173 -62.9168859
H 10.9628395 25.863104 -63.1596956
H 10.726691 27.5994604 -63.3176861
C 10.2581107 26.8168125 -61.3836782
O 11.1929088 27.3706979 -60.812188
N 9.2068629 26.3463782 -60.7000323
H 8.4713915 25.8971 -61.2280263
C 9.0731089 26.4374344 -59.2283582
H 9.1851045 27.4791121 -58.9258279
C 10.1069491 25.6136815 -58.4432941
O 10.4836519 25.994552 -57.3288833
C 7.6897912 25.9495096 -58.7796666
H 7.6527518 25.9341602 -57.6888603
H 7.5223822 24.9374057 -59.148062
O 6.6580577 26.7898418 -59.2502181
H 6.5249451 26.5892104 -60.1998395
N 10.5092653 24.4553591 -58.9775317
H 10.148396 24.2047745 -59.8925566
C 11.2703098 23.4359545 -58.2511685
H 11.53296 23.831252 -57.2725145
C 12.5826537 23.0539197 -58.9343996
O 12.7237566 23.0892687 -60.153064
C 10.4016771 22.1945978 -58.0069762
H 10.1678104 21.7295215 -58.9665107
H 10.9725225 21.4720044 -57.4234963
C 9.1174901 22.4918067 -57.2616053
C 9.1440574 22.7882535 -55.8861933
H 10.0770687 22.7663086 -55.3446341
C 7.8965917 22.5106125 -57.9559699

H 7.8822044 22.2996273 -59.0114969
C 7.9532069 23.1130515 -55.2127899
H 7.9771022 23.3415634 -54.1554797
C 6.7052395 22.8277978 -57.284616
H 5.7704859 22.841606 -57.8236995
C 6.7337942 23.135257 -55.9133376
H 5.8189192 23.3828901 -55.3936062
N 13.517583 22.5839715 -58.1188466
H 13.3072881 22.5350507 -57.1278463
C 14.8179288 22.0601079 -58.5444263
H 15.0314648 22.3351386 -59.5799209
C 14.8820061 20.5283609 -58.4571595
O 15.7122647 19.8905221 -59.1081171
C 15.861854 22.6970743 -57.6264017
H 15.4958654 22.6849266 -56.6000881
H 16.7664742 22.1004187 -57.6568063
C 16.1785143 24.1454572 -58.0345006
H 16.4935678 24.6931106 -57.143149
H 15.2907343 24.6425045 -58.4312527
C 17.305602 24.1476694 -59.067901
O 17.1190339 23.5958259 -60.1692975
O 18.44763 24.4781666 -58.6847995
N 14.0008482 19.9431131 -57.6415347
H 13.375073 20.5442361 -57.11928
C 13.8557196 18.5135042 -57.3961515
H 14.3000178 17.9505765 -58.2132188
C 12.3562485 18.1809218 -57.2916426
O 11.6055412 18.9392478 -56.6695756
C 14.5613807 18.1437631 -56.0764476
H 13.9703084 18.5856413 -55.2756477
H 14.5331341 17.0604963 -55.9537592
C 16.0075358 18.6382877 -55.8749913
H 16.0774433 19.7022195 -56.0961422
C 16.4272853 18.4705187 -54.4170553
H 16.4803628 17.4129 -54.1558347
H 15.7119752 18.972793 -53.7650525
H 17.3987076 18.9361346 -54.2814031
C 17.0207935 17.8886108 -56.7338335
H 16.9321508 16.814618 -56.5697651
H 18.0321975 18.2073195 -56.4892937
H 16.8337156 18.1120355 -57.7796173
N 11.9268476 17.0254245 -57.802589
H 12.5667476 16.4679935 -58.3580903
C 10.6134922 16.4408486 -57.4727742
H 10.200647 16.9817986 -56.6240596
C 10.8460628 14.9974708 -57.0338666

O 11.4968357 14.2196056 -57.7343886
C 9.584129 16.5453119 -58.623281
H 9.8891 15.8453796 -59.384544
C 9.5544193 17.9798466 -59.2141413
H 9.2019235 18.6905423 -58.4697116
H 10.5636784 18.2729503 -59.500348
C 8.1844474 16.0604285 -58.2049168
H 7.7911132 16.6907992 -57.4099126
H 8.2346799 15.0332518 -57.8434264
H 7.5072373 16.083636 -59.0554359
C 8.7221937 18.1222675 -60.4791129
H 7.6661354 18.1114382 -60.2270789
H 8.9737782 17.3097286 -61.1536164
H 8.9770545 19.0592215 -60.9698104
N 10.3564225 14.6574374 -55.8469035
H 9.7941865 15.3397182 -55.3446681
C 10.5610505 13.361721 -55.2088406
H 11.1527113 12.721799 -55.8623133
C 9.2265463 12.6740554 -54.9607565
O 8.2716464 13.3041168 -54.5053329
C 11.33476 13.5316808 -53.9000093
H 12.3374437 13.8914798 -54.1303004
H 10.8329836 14.2807638 -53.2847405
C 11.4560679 12.2414929 -53.107116
C 12.2584577 11.1893237 -53.5913009
H 12.8093437 11.3079028 -54.5115507
C 12.3285074 9.9712448 -52.892086
H 12.935959 9.1595666 -53.2719667
C 11.6002187 9.80203 -51.7022888
H 11.6597189 8.8679635 -51.1608939
C 10.7885537 10.8448729 -51.2227153
H 10.2173261 10.7141468 -50.3149552
C 10.7115946 12.0603749 -51.9250526
H 10.0647863 12.8469327 -51.5572318
N 9.1602094 11.3777451 -55.2613215
H 9.967209 10.9333375 -55.6892098
C 7.9328766 10.5948167 -55.1189933
H 7.301889 11.0701356 -54.3646456
C 8.255057 9.1831792 -54.6477118
O 9.0891373 8.509012 -55.2435789
C 7.1597415 10.5440188 -56.4471444
H 7.7114147 9.9222563 -57.1494066
C 5.8095002 9.8629732 -56.2113163
H 5.4896513 10.0212756 -55.1882349
H 5.9140703 8.7885214 -56.3956002
H 5.0585275 10.2781186 -56.8705113

C 7.0334598 11.9183157 -57.1168015
H 6.7794035 12.6694729 -56.3779092
H 6.2773547 11.9109892 -57.8926942
H 7.9869524 12.1928344 -57.5734035
N 7.5304832 8.6922801 -53.6430702
H 6.8760449 9.3036927 -53.1726615
C 7.6681163 7.3252057 -53.1358409
H 8.2068826 6.7406371 -53.8801015
C 6.2876119 6.6673452 -52.9862792
O 5.4097723 7.1751835 -52.2984167
C 8.5623854 7.3067255 -51.8731256
H 9.5579647 7.6240205 -52.1890165
C 8.094785 8.2976582 -50.7891014
H 7.0661867 8.0877915 -50.5071326
H 8.1462975 9.3195321 -51.1659953
H 8.7354529 8.2405992 -49.9112774
C 8.6896144 5.865025 -51.3446308
H 7.7397634 5.5693468 -50.9079072
H 8.8984866 5.198318 -52.1818034
C 9.804598 5.6643442 -50.3096542
H 9.5898272 6.2220642 -49.3977556
H 10.7584064 5.9929064 -50.7211156
H 9.8773923 4.6049462 -50.0589894
N 6.0735872 5.5791504 -53.7322323
H 6.8322139 5.3057652 -54.3480851
C 4.8587987 4.7465541 -53.7745885
H 4.9169297 4.1923917 -54.7089597
C 3.516118 5.5057891 -53.8614048
O 2.5191161 5.1118456 -53.2532807
C 4.8607276 3.7088065 -52.6438668
H 4.6254463 4.2273965 -51.7263386
H 4.0580549 2.9937707 -52.8329263
C 6.1573545 2.9237375 -52.4338794
H 5.9667513 2.1123083 -51.7318169
H 6.9089625 3.5794581 -51.9982039
C 6.7097361 2.3381834 -53.7201912
O 7.8204849 2.6367907 -54.124086
N 5.9596121 1.5408023 -54.4434662
H 6.3860314 1.1601171 -55.2659575
H 4.9443976 1.5731702 -54.3602218
N 3.4922299 6.609794 -54.6151327
H 4.3734201 6.9183661 -54.9925454
C 2.3102518 7.4619524 -54.7883182
H 1.523502 7.1422596 -54.1042785
C 1.7272705 7.3825057 -56.209906
O 0.5213259 7.2247492 -56.3878293

C 2.7044591 8.890572 -54.3960494
H 3.4554001 9.2689719 -55.0839756
H 3.1257726 8.8895343 -53.3873539
S 1.267988 9.9956678 -54.455393
H 1.9581013 11.1116626 -54.1764562
N 2.5785643 7.3956426 -57.2408166
H 3.5667076 7.4519611 -57.0465178
C 2.1412431 7.4998195 -58.6453044
H 1.3706166 8.2684934 -58.6961616
C 1.455654 6.224998 -59.1639819
O 0.6846533 6.2812467 -60.1185169
C 3.3271499 7.9979964 -59.498981
H 4.2271993 7.4604443 -59.1935633
C 3.5120954 9.5030321 -59.2234801
H 2.7433385 10.070109 -59.7525158
H 3.3888159 9.7067547 -58.1618482
C 3.1360484 7.7830679 -61.0060698
H 2.1735433 8.1823495 -61.3276595
H 3.2063335 6.7193164 -61.2171017
H 3.9295564 8.2650958 -61.5743967
C 4.8911054 10.0097662 -59.6504475
H 5.0099305 9.9628237 -60.7314375
H 5.6735898 9.4236341 -59.1685517
H 4.9797716 11.0455733 -59.3443017
N 1.5717582 5.1041732 -58.4564899
H 2.1827045 5.1206104 -57.6529454
C 0.7328439 3.9149034 -58.651388
H 0.8947388 3.5462889 -59.6639284
C -0.7823536 4.1812482 -58.5331897
O -1.5480582 3.5308124 -59.2486143
C 1.1982313 2.8047712 -57.6992863
H 0.5310809 1.9524817 -57.8022436
H 2.1807305 2.4791663 -58.0396875
C 1.2976814 3.1948038 -56.2119626
H 0.3012642 3.1688227 -55.7657597
H 1.6944627 4.205053 -56.0979221
C 2.2384522 2.2254211 -55.4939914
O 3.4720441 2.4194285 -55.590334
O 1.7426194 1.2099946 -54.9540701
N -1.2002262 5.2068174 -57.7761104
H -0.5129244 5.7123035 -57.2254629
C -2.598939 5.6605511 -57.6700557
H -3.2650489 4.7962077 -57.7183515
C -3.0249164 6.6303356 -58.7861373
O -4.212663 6.7435325 -59.0897442
C -2.8084363 6.3407486 -56.3074555

H -3.8525083 6.6461592 -56.2269157
H -2.1951503 7.2411331 -56.2540386
C -2.494111 5.4648105 -55.1222584
N -3.2748369 4.3995001 -54.6813414
C -1.402258 5.5724542 -54.3123075
H -0.6083186 6.2963122 -54.4092074
C -2.6439835 3.8919381 -53.61534
H -2.9894624 3.0311948 -53.0587202
N -1.5076655 4.5702295 -53.3754836
H -0.8044458 4.3204002 -52.6953078
N -2.0794667 7.3391896 -59.4136958
H -1.1158875 7.1556197 -59.1719981
C -2.3616997 8.3287098 -60.4667779
H -3.2276859 8.9173499 -60.1602128
C -2.7464924 7.5875773 -61.7548098
O -2.001621 6.7304906 -62.2333305
C -1.169923 9.3091347 -60.6337605
H -0.2399088 8.7535995 -60.5132491
C -1.1908147 10.461597 -59.5993055
H -0.3002541 11.0742561 -59.749503
H -2.0610367 11.0934907 -59.7853356
C -1.1248122 9.968303 -62.0256172
H -2.0519598 10.5058078 -62.2273489
H -0.9656117 9.221869 -62.8030621
H -0.2929621 10.6711498 -62.0761009
C -1.2165483 10.0459058 -58.1225757
H -0.4028053 9.3553923 -57.9105932
H -2.1699243 9.5788426 -57.8756608
H -1.0975652 10.9321865 -57.4986943
N -3.9191623 7.9002296 -62.3131057
H -4.4943561 8.5906527 -61.8567087
C -4.4811881 7.1647627 -63.4544377
H -4.2521094 6.1048541 -63.3300885
C -3.8496852 7.5951677 -64.7889643
O -3.2727482 6.7640869 -65.4867586
C -6.0132403 7.3088003 -63.4796484
H -6.2694267 8.3537053 -63.6622205
H -6.4039576 6.7184002 -64.3096062
C -6.7166646 6.8608593 -62.1861027
H -7.7887787 7.0203155 -62.3018223
H -6.3872767 7.4812947 -61.3521355
C -6.4970237 5.3889815 -61.8459751
O -6.9995508 4.4963847 -62.5114945
N -5.7572628 5.0902401 -60.8042215
H -5.833909 4.14822 -60.4307188
H -5.3482376 5.8097848 -60.2156199

N -3.8569681 8.8981386 -65.0946658
H -4.3202539 9.5439063 -64.4785248
C -3.1401507 9.4583446 -66.2477274
H -3.0411782 8.6833109 -67.0107969
C -1.7236527 9.8698317 -65.8282051
O -1.4618108 10.9868417 -65.364135
C -3.9305454 10.5951731 -66.9132344
H -4.0322571 11.4261952 -66.2131145
H -4.9304066 10.2295566 -67.1538309
C -3.2659619 11.0967388 -68.2087631
O -2.0957811 10.7292587 -68.4770125
O -3.9030893 11.919111 -68.8986723
N -0.803794 8.9111941 -65.9612056
H -1.116004 8.0117478 -66.302686
C 0.6148988 9.1220086 -65.6716635
H 0.701243 9.6227611 -64.7065084
C 1.2656105 10.0741342 -66.6689175
O 2.098196 10.8648421 -66.2441968
C 1.3529267 7.7757301 -65.5926566
H 1.1126767 7.1689474 -66.4679944
H 2.4297305 7.955249 -65.5762918
C 0.9574017 7.0366377 -64.3062496
H 1.2856483 7.6316669 -63.4540904
H -0.1280695 6.9419732 -64.2625537
C 1.5717334 5.633152 -64.2128231
H 2.6407791 5.6991469 -64.4166036
H 1.1128656 4.9710833 -64.9484462
C 1.3819517 5.0770151 -62.7971826
H 1.7874912 5.8138986 -62.1066302
H 1.9538723 4.1524789 -62.6816544
N -0.0351696 4.8430507 -62.4374898
H -0.6226182 5.656999 -62.6128357
H -0.1159741 4.6848988 -61.4413045
H -0.410129 4.0257107 -62.9061462
N 0.857613 10.1083443 -67.9375912
H 0.0306158 9.5949737 -68.2147783
C 1.4627276 11.0419099 -68.8908006
H 2.5495178 10.9536548 -68.8456466
C 1.0955327 12.4971915 -68.5945729
O 1.9570158 13.368256 -68.6695282
C 1.0240044 10.7936445 -70.3246517
H -0.0383867 11.0268319 -70.4208665
H 1.5987591 11.5133366 -70.9013528
C 1.2870139 9.4334093 -70.9591885
H 0.6207275 8.6875652 -70.5204886
H 2.321262 9.1555211 -70.7495981

C 1.0670261 9.5267931 -72.4818624
O 1.5837226 8.6346019 -73.1863309
O 0.449561 10.5278113 -72.9382914
N -0.1651418 12.7909911 -68.2489103
H -0.8538143 12.0316837 -68.2624408
C -0.597046 14.1400191 -67.8500139
H -0.3606733 14.8368522 -68.6493023
C 0.1788132 14.6128788 -66.6331437
O 0.6875716 15.7306213 -66.6664212
C -2.1126775 14.1466946 -67.5941196
H -2.6227997 14.0571914 -68.5530931
H -2.3576696 13.272807 -66.9910062
C -2.6896842 15.3612165 -66.8459386
H -3.7652708 15.2087699 -66.7430743
H -2.2667664 15.3935762 -65.8416272
C -2.4521123 16.7168604 -67.5216241
H -2.901966 16.7154243 -68.5155877
H -1.3820194 16.9074539 -67.6060141
C -3.0951271 17.8042082 -66.6541248
H -2.7560098 17.6658543 -65.6223159
H -4.1813943 17.6762267 -66.6795216
N -2.71647 19.159312 -67.1134731
H -1.700728 19.2804625 -67.0277463
H -3.1123092 19.8764223 -66.5258363
H -2.9676407 19.3212056 -68.075571
N 0.3204954 13.766832 -65.6119406
H -0.1301998 12.8600258 -65.6626692
C 1.1372004 14.0988161 -64.4489276
H 0.7681291 15.0311553 -64.0186157
C 2.6010132 14.3488103 -64.8440332
O 3.1203401 15.4231067 -64.5515805
C 1.0048208 13.0052398 -63.3833035
H -0.0274297 12.9715737 -63.0338871
H 1.2416141 12.0362933 -63.8251743
C 1.9196494 13.2544928 -62.2026704
C 1.5902525 14.2330469 -61.2449909
H 0.6507624 14.7645696 -61.3091331
C 2.5054578 14.5589645 -60.2283914
H 2.2645 15.3361693 -59.5185997
C 3.7477584 13.9046379 -60.163313
H 4.4610224 14.1635952 -59.3939693
C 4.075634 12.9324819 -61.1222156
H 5.0420339 12.4538226 -61.0965527
C 3.1634757 12.6011592 -62.1355357
H 3.4384687 11.8742898 -62.8856969
N 3.2219607 13.4431795 -65.6129092

H 2.7236004 12.5935768 -65.8627977
C 4.6285742 13.5613066 -66.0272132
H 5.2190656 13.6913037 -65.136371
C 4.8554892 14.8103973 -66.9071792
O 5.7538651 15.5982238 -66.6248052
C 5.1469566 12.2423806 -66.6375441
H 4.5264298 12.0049949 -67.503892
C 6.6112232 12.3569321 -67.0848273
H 7.294004 12.1712733 -66.2581363
H 6.8394987 13.3368053 -67.4929083
H 6.78462 11.6171695 -67.8603109
C 5.0988351 11.0728698 -65.6156212
H 5.8917595 11.1882859 -64.8758281
H 4.1709873 11.0890031 -65.0529447
C 5.2309691 9.6913749 -66.2729081
H 6.195366 9.5950969 -66.7695518
H 4.4343643 9.5515472 -67.0036455
H 5.1519131 8.9179254 -65.5089451
N 3.9636351 15.1292163 -67.8520424
H 3.2265652 14.4600239 -68.0547949
C 4.0218868 16.3871815 -68.6226211
H 5.0042765 16.4596209 -69.0901891
C 3.8797531 17.6361744 -67.751329
O 4.3853571 18.6900604 -68.1393617
C 2.9423549 16.3991231 -69.7126516
H 1.9845357 16.1193492 -69.2705717
H 2.8543825 17.4158555 -70.0989016
C 3.2701847 15.4763868 -70.8965743
H 3.2834725 14.434007 -70.5792135
H 4.2600221 15.7274747 -71.2810722
C 2.2462379 15.6651832 -72.0260354
H 2.5661878 15.0769262 -72.8877034
H 2.2537104 16.7118842 -72.3344896
N 0.873631 15.2917612 -71.613203
H 0.3736673 15.9475456 -71.0440993
C 0.2606047 14.1451865 -71.8617678
N 0.8324717 13.2109762 -72.562001
H 0.4215544 12.2750905 -72.7048818
H 1.7817286 13.3170273 -72.8573687
N -0.9394563 13.9204432 -71.4047869
H -1.4254517 14.5628763 -70.815964
H -1.3235025 12.9947997 -71.5411287
N 3.1468849 17.5726836 -66.640555
H 2.7147858 16.6893069 -66.3908726
C 3.0422072 18.6861717 -65.6891141
H 2.9536692 19.6115987 -66.2544648

C 4.3172776 18.8331359 -64.8542892
O 4.818539 19.9522103 -64.7316767
C 1.7891423 18.5422215 -64.8007602
H 1.0555546 17.9044931 -65.2924164
H 2.0612306 18.0606817 -63.8602092
C 1.1195113 19.8957002 -64.5028273
H 1.8473703 20.5638183 -64.0369374
H 0.3048766 19.7346467 -63.7929669
C 0.5491297 20.5310302 -65.7790337
O -0.2282403 19.8629453 -66.4986209
O 0.9457575 21.6510811 -66.1609826
N 4.94132 17.725144 -64.4234338
H 4.4998686 16.8199543 -64.5638902
C 6.2140599 17.8019982 -63.6909489
H 6.0524835 18.5815138 -62.9562327
C 7.3890477 18.3481159 -64.518059
O 8.2732855 18.9822581 -63.9445966
C 6.5604161 16.5360761 -62.8697259
H 7.6036421 16.612572 -62.5826921
C 5.687129 16.5745079 -61.5928064
H 4.6438896 16.3754152 -61.8451636
H 5.7360653 17.5498829 -61.1123979
H 6.0256541 15.83006 -60.8757306
C 6.3611885 15.1622226 -63.5084856
H 5.298497 14.9474787 -63.5301493
H 6.74844 15.1843201 -64.5161972
C 7.0610066 14.0140301 -62.7739185
H 6.6684855 13.8981371 -61.7667884
H 8.122155 14.2216184 -62.7084251
H 6.9110273 13.0840706 -63.3228865
N 7.30301 18.2991724 -65.852446
H 6.594574 17.6978391 -66.2541671
C 8.2430857 18.9945263 -66.7521705
H 9.256973 18.7089679 -66.4899572
C 8.1690656 20.51075 -66.5818082
O 9.1990222 21.1611148 -66.460308
C 7.977772 18.6399987 -68.229651
H 6.9892592 19.0220523 -68.484297
C 8.9845327 19.2914653 -69.1874602
H 10.0014391 18.9979181 -68.9194354
H 8.9126701 20.3778295 -69.1491886
H 8.7886603 18.966429 -70.2109033
C 7.96589 17.1408008 -68.5243393
H 8.943109 16.8146886 -68.8779901
H 7.2357508 16.9438971 -69.3046957
H 7.7171778 16.551408 -67.6508645

N 6.9615765 21.0893303 -66.532496
H 6.1488449 20.486938 -66.5022801
C 6.780615 22.5474602 -66.4135413
H 7.4330732 23.0541204 -67.1286945
C 7.2202667 23.0717325 -65.0569848
O 7.912679 24.0815246 -64.9821131
C 5.3159302 22.9327156 -66.6760503
H 4.6831159 22.049465 -66.5844177
H 4.9780527 23.653042 -65.9286919
C 5.1585947 23.5769347 -68.0589042
H 5.5905341 22.9257093 -68.8152424
H 5.7275276 24.5057287 -68.0837541
C 3.6905468 23.8966561 -68.3952165
H 3.6380314 24.2920585 -69.4108383
H 3.3606373 24.6917945 -67.7218206
N 2.7656957 22.7454938 -68.2527494
H 2.0283473 22.845749 -67.5579178
C 2.88091 21.5274568 -68.7454514
N 3.8693124 21.140862 -69.4991559
H 4.0949378 20.1565639 -69.4957941
H 4.5709032 21.8214323 -69.7161739
N 1.9903342 20.6334323 -68.4586807
H 1.2955849 20.8980357 -67.7519289
H 2.1477213 19.6774308 -68.6872359
N 6.8385406 22.3850171 -63.9793491
H 6.2678414 21.5594362 -64.1188139
C 7.0762139 22.8882716 -62.6154744
H 6.7536171 23.9302454 -62.5989197
C 8.5529194 22.901266 -62.209056
O 8.8936518 23.5075295 -61.1960446
C 6.2302789 22.1490035 -61.5659052
H 6.3002834 22.7158685 -60.6375336
C 4.7406745 22.1370212 -61.9312552
H 4.5608851 21.5072413 -62.7992222
H 4.4104188 23.1524728 -62.155835
H 4.1599278 21.7513496 -61.0951958
C 6.7187884 20.740197 -61.2639461
H 6.946144 20.2365441 -62.1944734
H 5.9697426 20.1820863 -60.7022476
H 7.6299397 20.7966649 -60.6732389
N 9.4330305 22.2598839 -62.9727844
H 9.1190088 21.8290885 -63.831774
C 10.8586508 22.2157185 -62.6987607
H 11.0205601 22.3896924 -61.6385775
C 11.6587618 23.2613323 -63.4943908
O 11.250341 23.686733 -64.571033

C 11.3162717 20.8126336 -63.009183
H 11.2222436 20.673128 -64.0870845
H 10.6948542 20.0962774 -62.4706286
H 12.34619 20.6925253 -62.698318
N 12.8048385 23.6814376 -62.9622398
H 13.0708624 23.3246349 -62.0518115
C 13.7495271 24.5578181 -63.6509041
H 13.1778 25.2862108 -64.2238724
C 14.6719354 23.7794981 -64.6223379
O 14.975169 22.6149417 -64.3663321
C 14.5534374 25.3133525 -62.584314
H 15.1349368 24.6050227 -61.9900518
H 13.8784704 25.8645982 -61.9278724
H 15.2402197 26.0126728 -63.0612602
N 15.2562672 24.4346856 -65.6428965
C 16.3457623 23.8565942 -66.4329207
H 15.9645131 23.0084207 -66.9972777
C 17.5084646 23.3942187 -65.5437926
O 17.9666931 24.1420647 -64.6815619
C 16.7604415 24.9539896 -67.41675
H 17.5396302 25.5804047 -66.9808329
H 17.0916402 24.5356973 -68.368371
C 15.4800201 25.7739039 -67.5748403
H 14.8102338 25.2746548 -68.2775496
H 15.6876092 26.7949285 -67.8961341
C 14.8755827 25.7311526 -66.1737705
H 13.7917232 25.8368128 -66.2352599
H 15.3069372 26.5190763 -65.555338
N 17.9761557 22.1537945 -65.7184936
H 17.5578649 21.5750566 -66.4370437
C 18.9691881 21.5330127 -64.828799
H 19.4528712 20.7173447 -65.3653614
H 19.7268063 22.271958 -64.5676816
C 18.3945524 20.9579177 -63.5231969
O 19.1431088 20.4234825 -62.6929978
N 17.0763422 20.9995032 -63.325819
H 16.4871733 21.5126475 -63.9742723
C 16.4200515 20.282351 -62.2438022
H 16.8992307 20.5608404 -61.3042546
C 16.5430015 18.7673145 -62.4295642
O 16.5979498 18.2344241 -63.5433115
C 14.9511917 20.6876016 -62.1594518
H 14.4759675 20.4721613 -63.1160417
H 14.876111 21.7519627 -61.935609
H 14.4412873 20.1255796 -61.3775577
N 16.4551063 18.0541021 -61.3125826

H 16.2855776 18.5690471 -60.4511779
C 16.3207142 16.6066863 -61.2672556
H 16.667364 16.1436285 -62.1925943
C 14.8430527 16.4876811 -61.1714432
O 14.2703098 16.4918358 -60.0760996
C 17.1025975 16.0055349 -60.0967373
H 16.7267382 16.4254048 -59.1674263
H 16.9332383 14.927969 -60.0696969
C 18.6151246 16.2789271 -60.2035818
H 18.7934009 17.3000794 -60.5397025
H 19.0498173 15.6006874 -60.9384452
C 19.3309473 16.1098317 -58.8681021
O 19.8209138 17.063498 -58.2767724
N 19.4280626 14.9016131 -58.3528361
H 19.9160454 14.8206799 -57.47876
H 19.0269415 14.1074667 -58.8221429
N 14.2900884 16.7928196 -62.3621341
H 14.9082487 16.9578584 -63.1449686
C 12.8660165 16.8921841 -62.5786515
H 12.50313 17.8129391 -62.118628
C 12.3945187 16.8195691 -64.0583579
H 11.5182164 16.1806944 -64.1205188
C 11.927267 18.2080647 -64.4477732
H 12.7140434 18.9289267 -64.2293846
H 11.0102521 18.4103486 -63.8975376
H 11.6871596 18.2879955 -65.5028421
C 13.3973882 16.3601632 -65.1198995
H 13.1242213 16.743578 -66.1014841
H 14.3632553 16.7589904 -64.8585091
C 13.4802412 14.8541873 -65.2635705
H 12.5137973 14.4524802 -65.5470749
H 13.7913259 14.4259101 -64.3196536
H 14.1809111 14.6061911 -66.0551717
C 12.2832975 15.764129 -61.7971147
O 11.2494559 16.0014477 -61.1925983
N 12.9953431 14.6160479 -61.6922139
H 13.8487719 14.4634771 -62.2140222
C 12.4409562 13.5648977 -60.9104669
H 11.9661348 14.0429408 -60.056893
C 13.3950247 12.5287825 -60.3251963
O 14.3898634 12.1321749 -60.9337501
C 11.3641755 12.9163869 -61.7760491
H 11.69877 11.9263678 -62.0871113
C 10.1448767 12.7675339 -60.8818847
H 9.7797381 13.7482455 -60.5753726
H 10.389334 12.200937 -59.985975

H 9.3515675 12.2659959 -61.4236928
C 10.8142608 13.647437 -63.0167973
H 10.2651247 14.5505195 -62.7809551
H 10.1246692 12.9691349 -63.5158578
H 11.6027534 13.8396563 -63.7244018
N 12.992268 12.0624941 -59.1407394
H 12.2290494 12.5696896 -58.7005972
C 13.3517678 10.7991302 -58.4875107
H 13.8691189 10.1454328 -59.1901295
C 12.022295 10.1409558 -58.0722694
O 11.2124517 10.7801555 -57.3905926
C 14.2732964 11.0619765 -57.2714117
H 13.7556098 11.7467959 -56.5983282
C 14.5539489 9.7552096 -56.5040559
H 15.098085 9.0547942 -57.1393494
H 13.6255968 9.2720965 -56.1977078
H 15.1326487 9.9473315 -55.6028041
C 15.5967541 11.7351388 -57.7104154
H 16.1322448 11.0671286 -58.3834904
H 15.3704105 12.6510699 -58.2557262
C 16.5299331 12.1281646 -56.5575202
H 16.9353557 11.2398739 -56.0737671
H 15.9862613 12.7290053 -55.8275692
H 17.3598219 12.7148318 -56.9523203
N 11.7580335 8.9046503 -58.5018071
H 12.4971308 8.4049395 -59.0006782
C 10.4983895 8.1867901 -58.2471085
H 9.9675434 8.6814518 -57.4396159
C 10.7986262 6.7492106 -57.8252037
O 11.102295 5.9443846 -58.7003625
C 9.5576584 8.1569015 -59.488233
H 10.0657489 7.6118162 -60.2850846
C 9.2280089 9.5527545 -60.0531933
H 8.7489912 10.1650888 -59.2892799
H 10.1684049 10.018405 -60.3410172
C 8.2538598 7.3944316 -59.1507915
H 7.707404 7.913199 -58.3643917
H 8.4738782 6.3822778 -58.8197286
H 7.6197814 7.2880813 -60.0276344
C 8.3419087 9.5094014 -61.311817
H 7.3388524 9.1658198 -61.0701747
H 8.7868381 8.8466241 -62.055071
H 8.2449684 10.4990386 -61.7479271
N 10.3778145 6.3647875 -56.6173089
H 10.0867171 7.0875804 -55.969359
C 10.0771669 4.965996 -56.2829504

H 10.6161371 4.3342597 -56.978693
C 8.5735844 4.707958 -56.4325046
O 7.7547345 5.5273144 -56.0055968
C 10.6004787 4.5910992 -54.8968489
H 10.1061673 5.1945444 -54.1366728
H 11.6735419 4.7889482 -54.8607758
O 10.3828632 3.2140938 -54.6418497
H 9.4472056 3.0366627 -54.4338089
N 8.1607001 3.6309068 -57.1055167
H 8.8769937 2.9804449 -57.4273788
C 6.7371884 3.300688 -57.3620578
H 6.1596131 3.5083897 -56.4621738
C 6.5491433 1.8064488 -57.6597888
O 7.4787363 1.1227106 -58.0834072
C 6.1822375 4.201798 -58.4902398
H 6.9381585 4.3099942 -59.2694911
C 4.8723056 3.766805 -59.1432156
H 4.1171477 3.6139869 -58.3752925
H 5.0235349 2.8460121 -59.7068042
H 4.5357146 4.5394803 -59.8340945
O 5.8902408 5.4700017 -57.9372644
H 6.6083844 5.6589023 -57.3062582
N 5.3715908 1.2506011 -57.3572631
H 4.6553821 1.8125038 -56.8983762
C 5.0533527 -0.1448405 -57.6414244
H 5.9175855 -0.7539901 -57.3692877
C 4.7686066 -0.3651727 -59.1348527
O 3.9382459 0.3149866 -59.7426248
C 3.8790949 -0.6020956 -56.7719286
H 2.9852171 -0.0491227 -57.0590648
H 4.0992285 -0.4156114 -55.7198245
H 3.7121333 -1.6687299 -56.9224242
N 5.384449 -1.4132231 -59.6710028
H 5.9615485 -1.978817 -59.0585404
C 5.274073 -1.8707675 -61.0481607
H 4.52063 -1.2819514 -61.5709594
C 4.819635 -3.3368143 -61.111532
O 4.71484 -4.0327033 -60.0985856
C 6.6290324 -1.6468094 -61.7413333
H 6.7247861 -2.2962304 -62.6122492
H 7.4480343 -1.8571985 -61.0513851
S 6.7161264 0.0744788 -62.2988248
H 5.7073454 -0.0156779 -63.1767116
N 4.5804398 -3.8163232 -62.3306187
H 4.6770828 -3.1691214 -63.1064769
C 4.346574 -5.2294842 -62.6551505

H 4.459447 -5.8264837 -61.7510928
C 5.4032849 -5.7429896 -63.6329606
O 6.1897972 -4.9572822 -64.1649364
C 2.9059161 -5.4309908 -63.1662228
H 2.7672556 -6.4849372 -63.4143421
H 2.2061259 -5.1920836 -62.3647088
C 2.5629917 -4.5759187 -64.3962885
H 3.3538419 -4.6507815 -65.1381943
H 2.4774119 -3.5317996 -64.1082129
C 1.239358 -5.0199314 -65.0200982
H 0.4409992 -4.8935195 -64.2851521
H 1.2911458 -6.0804497 -65.2787751
N 0.9258565 -4.2126199 -66.212061
H 0.4081314 -3.3590145 -66.0351975
C 1.2259418 -4.5210722 -67.4631425
N 1.9688723 -5.5468659 -67.7776155
H 2.1152903 -5.8721481 -68.7219343
H 2.3570204 -6.1389378 -67.0480665
N 0.7801215 -3.779182 -68.4391839
H 0.1596895 -3.0162654 -68.2328983
H 0.9754474 -4.0537471 -69.3841574
N 5.3899017 -7.0384504 -63.929528
H 4.7383203 -7.6344439 -63.4363782
C 6.0942102 -7.5596355 -65.1036889
H 7.0977374 -7.1300138 -65.1006469
C 5.4232967 -7.0925274 -66.4226341
O 4.3354509 -6.5106139 -66.4170726
C 6.2677109 -9.0838745 -64.9594063
H 6.6936781 -9.2945219 -63.9792089
H 6.9785677 -9.4402086 -65.7029615
C 4.9912565 -9.885612 -65.1365704
O 4.2025192 -9.6370408 -66.0222303
N 4.7808317 -10.9165378 -64.3629691
H 3.901636 -11.390675 -64.4666077
H 5.4341878 -11.1196392 -63.6117621
N 6.087026 -7.301176 -67.5654737
H 6.97492 -7.7768733 -67.5279906
C 5.5686416 -6.8960675 -68.8824775
H 5.3268371 -5.8354456 -68.847767
C 4.2717675 -7.636028 -69.2757134
O 3.3960169 -7.0361793 -69.9063768
C 6.6966829 -7.1057223 -69.9118019
H 7.5465829 -6.4850685 -69.622889
H 7.0144693 -8.1489414 -69.8602459
C 6.3428838 -6.7968035 -71.3816833
H 5.5274226 -7.4439008 -71.7033055

C 5.9359276 -5.3387014 -71.5982807
H 6.7312629 -4.6712647 -71.263949
H 5.0185384 -5.1252485 -71.0539862
H 5.7487945 -5.1681951 -72.6580208
C 7.5553389 -7.0841982 -72.2684394
H 8.3866897 -6.4341584 -71.9947723
H 7.2949337 -6.9136733 -73.313153
H 7.8578121 -8.1251123 -72.1530906
N 4.1334798 -8.8979656 -68.8636108
H 4.8250579 -9.2749777 -68.2335885
C 3.0282568 -9.7990911 -69.2066128
H 2.994302 -9.8630138 -70.2915907
C 1.6602333 -9.3119172 -68.6927965
O 1.5945623 -8.516802 -67.7502102
C 3.3072741 -11.2092038 -68.6610331
H 2.5364612 -11.8929918 -69.0166306
H 3.2739869 -11.2045757 -67.5732222
O 4.5717364 -11.6737482 -69.1032695
H 4.7435367 -12.5260357 -68.6915725
N 0.5433211 -9.7783737 -69.2905212
C -0.7985269 -9.4881579 -68.7920022
H -0.974344 -8.4156383 -68.878059
C -0.9754439 -9.9212677 -67.3331253
O -0.527133 -10.9988821 -66.9390965
C -1.7659872 -10.2366409 -69.7171588
H -1.976375 -11.2318292 -69.3196156
H -2.693753 -9.6827823 -69.8650191
C 0.4554123 -10.5666366 -70.5124142
H 1.1583618 -10.2258453 -71.2721388
H 0.6242898 -11.6193873 -70.280253
C -0.973233 -10.3730318 -71.0152853
H -1.0393598 -9.4455678 -71.586019
H -1.3115491 -11.2200922 -71.6130074
N -1.6717958 -9.0991233 -66.547554
H -1.9774122 -8.2113845 -66.9104634
C -1.9426282 -9.3712957 -65.1363939
H -0.9869589 -9.4906116 -64.6280691
C -2.7599268 -10.6578647 -64.9656591
O -3.8400503 -10.7898855 -65.5414905
C -2.701309 -8.1901542 -64.5041285
H -2.7718945 -8.3380187 -63.4261289
H -3.7101012 -8.1606276 -64.9151862
O -2.0772418 -6.945159 -64.7763668
H -2.4140318 -6.2669389 -64.1668781
N -2.292544 -11.5662814 -64.1043462
H -1.3627723 -11.44079 -63.7365719

C -3.0207712 -12.7921241 -63.7457168
H -3.2244875 -13.323451 -64.6781217
C -4.3966937 -12.4684447 -63.1343671
O -4.6230048 -11.3825377 -62.579092
C -2.1096583 -13.72106 -62.9038127
H -1.910148 -14.6234283 -63.4834728
H -1.1418491 -13.2284864 -62.8004951
C -2.5148567 -14.1252411 -61.4840284
H -2.7946617 -13.2267165 -60.9280213
H -1.6416138 -14.553518 -60.9864657
C -3.6505368 -15.1492933 -61.4302847
O -4.2452488 -15.22482 -60.332582
O -4.0275463 -15.7087837 -62.4791995
N -5.3275999 -13.4242844 -63.2208885
H -5.0081732 -14.3620026 -63.4746751
C -6.7373853 -13.2558538 -62.8708644
H -7.1275333 -12.4809128 -63.5271843
C -6.946757 -12.7679238 -61.4410492
O -7.9117529 -12.0322895 -61.2615722
C -7.4812086 -14.5788163 -63.1367407
H -7.2969487 -14.8900304 -64.1668939
H -7.0568127 -15.3459934 -62.4846586
C -8.9995942 -14.5392484 -62.9048429
H -9.3885239 -15.5543231 -62.9959171
H -9.1947796 -14.2219061 -61.8805407
C -9.7553918 -13.6445641 -63.9063237
H -10.1326223 -14.2766743 -64.7127237
H -9.0952164 -12.9031723 -64.3565096
C -10.9343519 -12.935928 -63.2296966
H -11.4774207 -13.6713244 -62.6279936
H -11.6147183 -12.563259 -64.0023007
N -10.4659423 -11.8111498 -62.3804404
H -9.7675482 -12.1340372 -61.710333
H -11.2343898 -11.3862407 -61.8810379
H -10.0067443 -11.1094506 -62.9439105
N -6.0816248 -13.0886981 -60.4718269
H -5.3969747 -13.8110332 -60.6825751
C -6.0968226 -12.5397581 -59.1017552
H -7.1172031 -12.2357542 -58.8791371
C -5.2212816 -11.2922417 -58.8815129
O -5.4720267 -10.5585664 -57.9262851
C -5.7549487 -13.6167123 -58.0640669
H -6.3952429 -14.4869167 -58.2181221
H -5.9482444 -13.2182487 -57.0671658
O -4.3992676 -14.0098058 -58.1432093
H -4.343102 -14.5866227 -58.960205

N -4.2784466 -10.9636136 -59.7796263
H -4.191226 -11.5356199 -60.6116894
C -3.5645761 -9.675878 -59.7459851
H -3.2982635 -9.4612025 -58.710529
C -4.4764878 -8.5391038 -60.2170946
O -4.6424323 -7.5619692 -59.492832
C -2.2637066 -9.7208699 -60.5782299
H -1.5860132 -10.4576694 -60.1459091
H -2.5060962 -10.0411425 -61.5913691
C -1.54592 -8.348949 -60.6594275
H -2.2252017 -7.6004366 -61.0640231
C -1.049715 -7.869789 -59.2926931
H -0.3925492 -8.6162577 -58.8481829
H -1.895953 -7.6778135 -58.6345231
H -0.5004847 -6.9352805 -59.4146506
C -0.3461376 -8.4080241 -61.5969519
H 0.3865714 -9.1103831 -61.226032
H 0.1108929 -7.4212003 -61.6794127
H -0.6523785 -8.7230191 -62.5882788
N -5.1169134 -8.689943 -61.3849318
H -4.9457027 -9.5459942 -61.90868
C -6.0412299 -7.6860189 -61.9416269
H -5.4551489 -6.7857532 -62.1437637
C -7.1240799 -7.231914 -60.9375162
O -7.2297424 -6.0346225 -60.7273625
C -6.5934143 -8.1928127 -63.2907024
H -5.7661369 -8.2380254 -63.9984981
H -6.9541082 -9.2147834 -63.16253
C -7.7329938 -7.3712305 -63.9310273
H -7.8971692 -7.7500898 -64.9408023
H -8.6442331 -7.5659483 -63.3636415
C -7.5575872 -5.8442729 -64.0094301
H -7.4980538 -5.4352241 -63.0077227
H -8.4615509 -5.4163366 -64.4482493
C -6.3614685 -5.3390559 -64.8205827
H -6.662531 -5.2804452 -65.8695286
H -5.5195257 -6.0309988 -64.7300999
N -5.9571078 -4.0034871 -64.3263948
H -6.7679802 -3.395136 -64.17414
H -5.2630821 -3.5587778 -64.9038722
H -5.4837897 -4.0957187 -63.4295262
N -7.871645 -8.0931999 -60.2239082
C -8.873936 -7.6616363 -59.2573345
H -9.5234761 -6.9139405 -59.7159941
C -8.2649088 -7.0320422 -57.9992117
O -8.9337415 -6.198365 -57.3998078

C -9.7099499 -8.8997193 -58.9176722
H -10.0869565 -8.8805224 -57.8938592
H -10.5345133 -8.9914458 -59.6264198
C -7.9351771 -9.5147877 -60.3439147
H -8.4774898 -9.7379089 -61.262144
H -6.9490279 -9.9651637 -60.3563997
C -8.720291 -10.0246495 -59.1344312
H -9.2281338 -10.9685741 -59.3297095
H -8.0659944 -10.10908 -58.2666461
N -7.021786 -7.3606245 -57.6043131
H -6.4998668 -8.0322734 -58.1535313
C -6.3056253 -6.6353359 -56.5348897
H -6.9728801 -6.5111878 -55.6824392
C -5.9322881 -5.2270172 -57.0036012
O -6.1602659 -4.2700824 -56.2681912
C -5.0687729 -7.4390979 -56.0860549
H -5.3895788 -8.424192 -55.7418211
H -4.4305065 -7.5948128 -56.9556649
C -4.1992388 -6.7757217 -54.993123
H -3.2115868 -7.2341931 -55.0481208
H -4.0668317 -5.7140503 -55.2046993
C -4.7169933 -6.9550987 -53.5545555
H -4.8133604 -8.0239603 -53.351868
H -5.6999526 -6.4874112 -53.4551922
C -3.7314371 -6.3211879 -52.5553124
H -3.9467867 -5.2535484 -52.4822222
H -2.7197203 -6.4281609 -52.9491867
N -3.7880118 -6.9492504 -51.2117734
H -4.7008678 -6.8646728 -50.7950431
H -3.1294465 -6.5095671 -50.5629086
H -3.5314121 -7.9249485 -51.2652042
N -5.4101965 -5.0928218 -58.2204721
H -5.2513331 -5.9347831 -58.7634563
C -5.1139113 -3.8079675 -58.8626221
H -4.4172266 -3.2441876 -58.2414818
C -6.3827766 -2.9486339 -58.9977917
O -6.424878 -1.8461333 -58.4601641
C -4.4306352 -4.0925634 -60.2147685
H -3.4889227 -4.612916 -60.0251308
H -5.0681263 -4.750292 -60.8032861
C -4.1402352 -2.8329003 -61.0402102
H -3.5223312 -2.1630568 -60.4391233
H -5.0881717 -2.3339607 -61.2560169
C -3.4537445 -3.1310734 -62.3813936
O -3.6879035 -4.2298107 -62.9487496
O -2.7668995 -2.224337 -62.9038172

N -7.4664462 -3.5002571 -59.5499567
H -7.3734285 -4.4240226 -59.9575726
C -8.7116426 -2.7639545 -59.7964255
H -8.4353106 -1.8095062 -60.2480485
C -9.4638687 -2.4089424 -58.5074308
O -9.9528278 -1.288221 -58.3755535
C -9.6118555 -3.5466591 -60.7821039
H -9.0607949 -4.3693492 -61.2330581
H -10.4668038 -3.9705644 -60.2532929
C -10.1111674 -2.6392604 -61.9230753
H -10.7214885 -1.8416841 -61.4932013
H -10.7558622 -3.2309231 -62.5768496
C -8.9667387 -2.0313695 -62.7636765
O -8.2371866 -2.7994259 -63.4391515
O -8.8066446 -0.7847794 -62.7441526
N -9.47042 -3.2962327 -57.4999447
H -9.0417097 -4.20374 -57.6524768
C -10.1010431 -3.0170332 -56.1973756
H -11.0288266 -2.4764864 -56.3973745
C -9.2769901 -2.0761537 -55.3142795
O -9.8498703 -1.4157787 -54.4554675
C -10.4660358 -4.334596 -55.4944689
H -11.0058283 -4.9560219 -56.2112972
H -9.5566922 -4.8544857 -55.1890845
C -11.3704398 -4.1053174 -54.2696322
H -10.7558442 -3.807233 -53.4188719
H -12.0692675 -3.2953097 -54.4855386
C -12.2007088 -5.3376816 -53.8874421
H -12.8839548 -5.5640901 -54.7082912
H -12.7938671 -5.0865768 -53.0059178
C -11.3301939 -6.5636396 -53.5898349
H -10.6293076 -6.3072624 -52.7892185
H -10.7480368 -6.8020651 -54.4859351
N -12.1634946 -7.726805 -53.1936491
H -12.701381 -7.5095402 -52.3633894
H -11.5843428 -8.534113 -52.9993255
H -12.8073944 -7.9650457 -53.9371857
N -7.9716141 -1.9444161 -55.5661454
H -7.5758423 -2.5040646 -56.3110106
C -7.1488087 -0.8533673 -55.0040867
H -7.6222074 -0.5067725 -54.0856447
C -7.1064176 0.3871534 -55.9041949
O -6.4532318 1.363562 -55.5514579
C -5.7327836 -1.3132419 -54.6059369
H -5.1704231 -0.4493004 -54.2512706
C -5.7686133 -2.3392788 -53.4714731

H -6.3200593 -3.2274988 -53.7777969
H -6.2540956 -1.8980821 -52.6008782
H -4.7497511 -2.6174525 -53.2046511
O -5.0121386 -1.9015539 -55.6648057
H -5.5081724 -2.690785 -55.9458878
N -7.8166632 0.3654646 -57.0417343
H -8.3462446 -0.4726061 -57.2427193
C -7.8502309 1.3899778 -58.0923619
H -8.3461036 0.901136 -58.9292334
C -6.4594769 1.7892235 -58.633805
O -6.3001095 2.8344891 -59.2640218
C -8.7526115 2.5574542 -57.6322694
H -9.6187161 2.1305136 -57.1235023
H -8.1999693 3.1478449 -56.8997247
C -9.2803135 3.5032468 -58.7320415
H -8.4579137 4.0436594 -59.1952806
C -10.0659324 2.771923 -59.8264709
H -10.8596408 2.1741863 -59.3761475
H -9.4021933 2.1159166 -60.3858728
H -10.5024011 3.4948423 -60.5147045
C -10.2132689 4.5376623 -58.0986059
H -11.0746174 4.0426197 -57.649767
H -10.5543428 5.2422816 -58.85601
H -9.6730952 5.0878871 -57.3275553
N -5.4426723 0.9434196 -58.4481881
H -5.6678236 0.0355238 -58.0548346
C -4.0798935 1.185912 -58.9357536
H -3.8150141 2.2211407 -58.7113507
C -4.0051982 1.032849 -60.4518383
O -4.7678273 0.27695 -61.0434649
C -3.0949649 0.2568287 -58.2130025
H -3.5138127 -0.7490093 -58.1422002
H -2.1678002 0.2159683 -58.784929
C -2.795052 0.8141 -56.8097441
H -2.3119699 1.7866437 -56.9208895
H -3.7301621 0.9656837 -56.2690902
C -1.8893985 -0.0773955 -55.9502131
H -2.4545609 -0.9480272 -55.6154601
H -1.5950424 0.5033683 -55.0736083
C -0.6439782 -0.5463608 -56.7108241
H -0.3018319 0.268945 -57.3493463
H -0.9215663 -1.3830817 -57.3566641
N 0.4467047 -0.9339892 -55.7916971
H 0.7465728 -0.112789 -55.2498367
H 1.2748543 -1.2105249 -56.2979961
H 0.1611309 -1.6486113 -55.1440047

N -3.0487572 1.7262357 -61.0774219
H -2.4915023 2.3509615 -60.5015827
C -2.7384924 1.6213578 -62.5167704
H -3.2764944 0.767828 -62.9341887
C -1.2710996 1.2723612 -62.7035448
O -0.4066791 2.1504201 -62.6343866
C -3.1835379 2.9095297 -63.2545982
H -3.4195988 3.6894009 -62.5300726
H -2.3764105 3.2816492 -63.8842477
C -4.4157079 2.6815394 -64.1627795
H -4.7723658 3.6569256 -64.4963477
H -4.1117891 2.1108096 -65.0407656
C -5.5713926 1.9356428 -63.4710507
H -5.670715 2.3288555 -62.460506
H -5.3424209 0.8725846 -63.4125411
C -6.9189723 2.0674559 -64.1837241
H -6.9312356 1.427326 -65.0694601
H -7.0421595 3.108668 -64.4916204
N -8.0097774 1.7033435 -63.2510456
H -7.9605382 0.719054 -62.960874
H -8.919042 1.7965361 -63.6756161
H -7.9574248 2.2846703 -62.4272009
N -0.9851324 -0.0152724 -62.8685472
H -1.7507671 -0.6921228 -62.9343551
C 0.3622951 -0.5630426 -62.7563147
H 0.9805693 0.166144 -62.2307181
C 0.9623679 -0.7635467 -64.1569443
O 0.4536773 -1.5034089 -65.0035394
C 0.3662204 -1.8384641 -61.8795023
H -0.0630003 -2.6610733 -62.4548948
C 1.819163 -2.1735927 -61.5191769
H 2.2062048 -1.4454116 -60.8056847
H 2.4410637 -2.152216 -62.4072739
H 1.8767011 -3.1699076 -61.08193
C -0.4305112 -1.6738873 -60.5577608
H 0.0351023 -0.9039866 -59.9410057
H -1.442414 -1.3440835 -60.7786126
C -0.5557201 -2.9669112 -59.7395202
H 0.4082644 -3.2467547 -59.3150915
H -0.9233073 -3.7726035 -60.3764866
H -1.265722 -2.8133331 -58.927048
N 2.0820627 -0.0851478 -64.4029156
H 2.4655832 0.475942 -63.6575331
C 2.9068083 -0.2770199 -65.5962123
H 2.3018658 -0.6952979 -66.4020926
C 4.0396154 -1.2643317 -65.292497

O 4.4307098 -1.4269569 -64.1332412
C 3.4495725 1.0814523 -66.061202
H 4.0958151 0.9525892 -66.9322466
H 4.0432311 1.5292122 -65.261646
S 2.0709612 2.1846743 -66.4876026
H 1.3003227 1.8921115 -65.4342484
N 4.6448883 -1.8495044 -66.3268338
H 4.3466771 -1.6176306 -67.261478
C 5.9932657 -2.3856146 -66.1595621
H 6.0491473 -2.8706147 -65.1886549
C 7.0088041 -1.2347704 -66.1249314
O 6.737833 -0.132612 -66.6086652
C 6.3073177 -3.4524623 -67.2163512
H 5.4957053 -4.1766962 -67.2517385
H 7.2191217 -3.9815768 -66.9375472
C 6.5230252 -2.8364934 -68.5773457
O 7.5678347 -2.280934 -68.8635157
N 5.5086591 -2.8222407 -69.403895
H 5.6807276 -2.3748843 -70.3032807
H 4.6772054 -3.3460722 -69.2191437
N 8.1801732 -1.5131329 -65.5618081
H 8.3470506 -2.4508453 -65.2320084
C 9.2452561 -0.5331003 -65.3748175
H 8.8589516 0.286887 -64.7647094
C 9.6957767 0.098359 -66.7072817
O 9.7789872 1.3222783 -66.8014925
C 10.3873618 -1.2029495 -64.5780293
H 11.2574185 -0.549599 -64.5671677
H 10.0590078 -1.2929866 -63.5414857
C 10.8177317 -2.5865022 -65.0490904
C 11.9181375 -2.7365552 -65.9169673
H 12.4819757 -1.8755661 -66.2390297
C 12.3053206 -4.0136478 -66.363435
H 13.1452796 -4.1187607 -67.0335377
C 11.597087 -5.1547648 -65.9319127
O 11.9592972 -6.3881262 -66.3699191
H 12.7274721 -6.3595775 -66.9421564
C 10.5129368 -5.0118722 -65.040923
H 9.9908157 -5.8930459 -64.6965091
C 10.1282528 -3.7322316 -64.5973171
H 9.308215 -3.6379075 -63.8983316
N 9.914116 -0.6953223 -67.7660937
H 9.7185 -1.6813804 -67.6780474
C 10.4258322 -0.1969978 -69.0526211
H 11.3531195 0.3334907 -68.8684677
C 9.4744436 0.8147866 -69.7068052

O 9.9050969 1.903903 -70.0918449
C 10.7555701 -1.3866897 -69.9704476
H 11.4799712 -2.0264374 -69.4643361
H 9.8468928 -1.968167 -70.1290859
C 11.319076 -0.9912917 -71.3499645
H 10.5886703 -0.3825456 -71.8825983
C 12.633967 -0.2142643 -71.2488822
H 13.3625122 -0.779567 -70.6682336
H 12.462991 0.7532524 -70.7772734
H 13.0319279 -0.0350601 -72.2476385
C 11.5735498 -2.2547509 -72.1719436
H 12.3160219 -2.8816896 -71.6783682
H 11.9344282 -1.9816748 -73.1636924
H 10.6437644 -2.8131246 -72.2827093
N 8.1721243 0.5219023 -69.7300654
H 7.8781855 -0.3931025 -69.4008782
C 7.1627271 1.4694778 -70.216607
H 7.465206 1.8258281 -71.2019721
C 7.0485684 2.7218684 -69.344085
O 6.7977963 3.7974389 -69.8845881
C 5.8037654 0.7721156 -70.3591403
H 5.0142317 1.5249066 -70.3314356
H 5.6424419 0.0910384 -69.5229777
C 5.6722935 0.0300481 -71.6647418
N 5.9532087 -1.3209948 -71.8725283
C 5.7499517 -1.545272 -73.178947
H 5.897551 -2.4993019 -73.6695688
N 5.3500598 -0.4176791 -73.790252
H 5.1471925 -0.3273254 -74.7758818
C 5.2980715 0.5887433 -72.851667
H 5.0341347 1.6258243 -73.0140616
N 7.3164396 2.6332608 -68.0360767
H 7.5599259 1.7314697 -67.6420576
C 7.2614763 3.7968527 -67.1509479
H 6.3620564 4.3393293 -67.4291629
C 8.4261241 4.7738874 -67.3880553
O 8.1695259 5.9696373 -67.5199989
C 7.0948289 3.3363918 -65.6891448
H 6.3288467 2.5601209 -65.6683109
H 8.0271337 2.8928056 -65.3390609
C 6.6681347 4.4549309 -64.7117129
H 7.5189438 5.109165 -64.5258907
C 5.4997377 5.3156355 -65.2074584
H 4.6757884 4.679795 -65.5306189
H 5.8301918 5.9439793 -66.0348046
H 5.1677863 5.9791257 -64.4099764

C 6.2145039 3.8387536 -63.3851094
H 5.3016056 3.2618588 -63.5260214
H 6.0445249 4.6237662 -62.6487135
H 6.9882306 3.1722436 -63.0041928
N 9.6480168 4.2850584 -67.6533181
H 9.7997559 3.2846604 -67.5630426
C 10.7262591 5.1507473 -68.1801541
H 10.8557976 5.9933419 -67.499146
C 10.3952471 5.6900104 -69.5631661
O 10.7418516 6.824877 -69.8713061
C 12.0605472 4.4240518 -68.3016877
H 12.1166467 3.8610945 -69.2350101
H 12.121121 3.7293563 -67.4803064
O 13.1491082 5.3247477 -68.1948499
H 13.269569 5.8510611 -69.0056855
N 9.6793356 4.9112594 -70.3797769
H 9.4739327 3.9631262 -70.084374
C 9.1529553 5.3651188 -71.6612905
H 9.9800387 5.6169965 -72.3253587
H 8.5721268 4.5597261 -72.1096179
C 8.2436502 6.5889815 -71.5206075
O 8.4964877 7.5881455 -72.1829673
N 7.271716 6.5802667 -70.6017955
H 7.1008963 5.7179054 -70.0934582
C 6.3753098 7.7248641 -70.3564864
H 5.913338 8.0175862 -71.2986071
C 7.1064366 8.967594 -69.8279953
O 6.7901523 10.0960769 -70.2162708
C 5.2706065 7.3106618 -69.3736671
H 5.7289851 6.9026424 -68.4718638
H 4.7203521 8.2063148 -69.0832355
C 4.2527546 6.3259864 -69.9274463
C 3.9569052 5.1377987 -69.2312677
H 4.470828 4.90886 -68.3126257
C 2.9868958 4.2463839 -69.7247382
H 2.7656118 3.3367646 -69.1875547
C 2.2932897 4.5473782 -70.9100298
H 1.5351606 3.8746796 -71.2849157
C 2.5749518 5.7375725 -71.6014384
H 2.0297721 5.995317 -72.5016579
C 3.5527959 6.6201892 -71.1146242
H 3.7383022 7.5375435 -71.6559592
N 8.1333138 8.7500029 -69.0098282
H 8.2811018 7.8069615 -68.6678179
C 9.07562 9.7716512 -68.5730746
H 8.5547353 10.5922679 -68.0912188

C 9.8605926 10.3853852 -69.7513712
O 9.7566054 11.5847949 -70.0065046
C 9.9597189 9.1076336 -67.5219712
H 10.8339721 9.7058895 -67.3759088
H 10.2895408 8.1279684 -67.8552867
S 9.0625811 8.9471298 -65.9503501
H 9.8351265 9.8474364 -65.3019252
N 10.4805271 9.5504548 -70.5898632
H 10.5030738 8.5688311 -70.3340113
C 11.2121939 9.9579073 -71.8004392
H 12.0155281 10.6371849 -71.5108146
C 10.3189662 10.6842638 -72.8220458
O 10.6706195 11.7496569 -73.3218699
C 11.8287769 8.7012708 -72.4308687
H 11.0787146 8.1829872 -73.0301747
H 12.1581597 8.0264929 -71.641054
O 12.9535704 9.016732 -73.2287619
H 13.2151123 8.2260534 -73.7115009
N 9.0917743 10.1931924 -73.0341901
H 8.8462209 9.3288767 -72.56349
C 8.0758771 10.7997598 -73.908273
H 8.5230395 10.9933282 -74.8827677
C 7.5601152 12.1572996 -73.4059811
O 6.9709603 12.9057875 -74.1813275
C 6.8980951 9.8177845 -74.0665242
H 6.5963806 9.4797408 -73.0746319
H 6.0469815 10.3468555 -74.4990079
C 7.2010356 8.596359 -74.9559661
H 8.1359891 8.1311667 -74.6495739
C 6.078985 7.5667695 -74.8172612
H 5.1265064 8.0041828 -75.1217947
H 6.002105 7.244648 -73.7790986
H 6.2918725 6.6974123 -75.4373065
C 7.3195826 8.9722824 -76.4354788
H 6.4050387 9.4644876 -76.7684964
H 7.4802548 8.0744735 -77.031481
H 8.1643764 9.6422728 -76.586479
N 7.8083199 12.5056056 -72.1428918
H 8.3267403 11.8644839 -71.5540116
C 7.4700463 13.8201725 -71.5844624
H 6.6249218 14.2248713 -72.140381
C 8.5965015 14.8537852 -71.7283394
O 8.4412966 15.9715581 -71.2569983
C 7.0096966 13.6702722 -70.1392581
H 6.7278152 14.646009 -69.7435907
H 7.8241777 13.2648047 -69.5378654

O 5.8818799 12.8084382 -70.0899695
H 6.1934362 11.8939971 -70.2199382
N 9.6527789 14.5194905 -72.4726668
H 9.6609405 13.583641 -72.8564542
C 10.8450282 15.3307806 -72.7823003
H 11.3215018 14.7681748 -73.5857787
C 11.9223128 15.3449434 -71.6844862
O 12.7208264 16.2703293 -71.5460031
C 10.4885248 16.6983789 -73.4123986
H 10.3052822 17.4216129 -72.6154718
H 9.5630553 16.5886969 -73.9810148
C 11.5483248 17.2610639 -74.3780375
O 12.3958398 16.4764423 -74.8659835
O 11.4371665 18.4657644 -74.7049311
N 11.9834183 14.26542 -70.8998863
H 11.310854 13.5258562 -71.0438426
C 13.0028759 14.0912496 -69.8714545
H 13.306885 15.0728525 -69.5156226
C 14.2829326 13.4339092 -70.3807713
O 14.2769208 12.3465484 -70.963321
C 12.4106606 13.3349922 -68.6923727
H 12.0504773 12.3665639 -69.0383334
H 13.215576 13.1470367 -67.9823596
C 11.3089208 14.0511588 -67.9372688
C 10.4143586 13.2895275 -67.1652494
H 10.4207411 12.2129036 -67.2452246
C 9.622269 13.9020251 -66.183757
H 9.0420531 13.2912381 -65.5123696
C 9.6896914 15.2939169 -66.0117228
O 9.0688287 15.8810816 -64.9659394
H 8.6517885 15.2197967 -64.4135997
C 10.476267 16.0688853 -66.8777947
H 10.5008894 17.1334103 -66.7732363
C 11.2869235 15.4568867 -67.8389113
H 11.9309688 16.084664 -68.4366982
N 15.413457 14.0666489 -70.0648519
H 15.3470006 14.8954175 -69.4865486
C 16.7466287 13.6072225 -70.4479379
H 16.6439033 13.0148765 -71.3578561
C 17.3845581 12.7089696 -69.3861672
O 17.0498046 12.785335 -68.2019845
C 17.6340751 14.8021464 -70.8021027
H 17.0888067 15.4656922 -71.4759625
H 18.5250258 14.4458703 -71.3186039
O 18.0387931 15.5263655 -69.6592734
H 17.3011331 15.5682376 -69.0191956

N 18.3085085 11.8526167 -69.839663
H 18.540374 11.8840546 -70.8182419
C 19.0346307 10.8940314 -69.0057114
H 19.4675616 10.1472293 -69.6713046
C 18.1121144 10.1118048 -68.0476025
O 18.3664568 10.0593789 -66.8463913
C 20.2060694 11.6112569 -68.3167241
H 19.8233584 12.3411857 -67.6012615
H 20.822286 12.1241624 -69.055329
H 20.8190484 10.8836954 -67.7848943
N 17.0082147 9.5547067 -68.5680509
H 16.8205954 9.6340163 -69.5538031
C 16.1258953 8.7216485 -67.7571872
H 16.0455573 9.2076642 -66.7871337
C 16.7726785 7.3640399 -67.4612841
O 17.037384 6.5613184 -68.3551552
C 14.6792575 8.6592675 -68.293561
H 14.2122908 9.6269136 -68.1101076
H 14.1203773 7.931987 -67.704949
C 14.479784 8.3334479 -69.7606206
O 14.1690558 7.2130685 -70.1375173
N 14.5042034 9.3331716 -70.6191546
H 14.1908225 9.1433714 -71.5603483
H 14.5384129 10.2922772 -70.2963576
N 17.0392665 7.1424113 -66.177116
H 16.830673 7.8928146 -65.5251381
C 17.5157217 5.8978433 -65.5954028
H 17.9177789 5.2616605 -66.384763
C 16.3357946 5.207524 -64.9288189
O 15.6159122 5.8225069 -64.1466295
C 18.6212937 6.1737034 -64.5672921
H 18.1711266 6.6156535 -63.6769974
H 19.0702786 5.2249355 -64.2716113
C 19.7093446 7.0913046 -65.0380035
C 19.7931824 8.4044157 -64.7311366
H 19.0720161 8.9404149 -64.124824
N 20.8504143 8.9783652 -65.4059943
H 20.9953366 9.9773354 -65.4322483
C 21.4912532 8.0630289 -66.2111008
C 22.5607112 8.1678796 -67.1126483
H 23.0547657 9.1163029 -67.2628505
C 22.976687 7.0234162 -67.8144749
H 23.7994497 7.0845023 -68.5131482
C 22.3164877 5.7977862 -67.6094385
H 22.6337933 4.9207785 -68.1562422
C 21.2368826 5.7069224 -66.707767

H 20.7248034 4.766601 -66.5741431
C 20.7952953 6.8365602 -65.9837824
N 16.1848563 3.912817 -65.176291
H 16.821799 3.469261 -65.8205527
C 15.0796393 3.1104436 -64.6775838
H 14.54308 3.6333506 -63.8833365
C 15.6247004 1.7985474 -64.1325112
O 16.0577958 0.9402323 -64.9020713
C 14.131694 2.8887033 -65.8487103
H 13.64995 3.8395378 -66.0623428
H 14.7289034 2.6128478 -66.7202805
C 13.0901348 1.8002141 -65.5654381
H 13.4487714 1.0489044 -64.8679001
C 11.8698551 2.4330043 -64.9143906
H 11.3898081 3.1481945 -65.575671
H 12.1699034 2.9421886 -63.9979327
H 11.1508884 1.6662489 -64.6483251
C 12.8433832 1.0456084 -66.8611406
H 12.3756177 1.6885177 -67.5980209
H 12.2143535 0.1981272 -66.6495824
H 13.7858171 0.6635186 -67.2549475
N 15.5022455 1.6123315 -62.8227831
H 15.0176951 2.3212374 -62.2749974
C 15.9819101 0.4156113 -62.1354336
H 16.5767406 -0.1688088 -62.8309248
C 14.7948239 -0.412656 -61.638592
O 14.0877462 0.0314062 -60.7298387
C 16.9228566 0.7713672 -60.9737509
H 16.3487296 1.1324943 -60.119959
C 17.7625409 -0.435787 -60.5611816
H 18.3692173 -0.7740933 -61.4018301
H 17.1180753 -1.2500821 -60.2345302
H 18.4224701 -0.1512396 -59.7425426
O 17.83844 1.7718527 -61.3585353
H 17.3314862 2.5714806 -61.5236629
N 14.5757734 -1.6381667 -62.1522123
C 13.7832369 -2.6243789 -61.4326093
H 12.8078798 -2.2156713 -61.1651937
C 14.5459745 -3.0172483 -60.1610187
O 15.6270337 -3.5983735 -60.2304277
C 13.5987399 -3.7952014 -62.405327
H 13.5382697 -4.7567764 -61.8952256
H 12.6985773 -3.6284725 -62.9974839
C 15.1494966 -2.2202696 -63.3610773
H 14.6833669 -1.7644417 -64.236083
H 16.2312348 -2.0961457 -63.4033807

C 14.823558 -3.7137458 -63.3158808
H 14.6132543 -4.1093829 -64.3096971
H 15.6551597 -4.2543635 -62.8618106
N 13.9662979 -2.746838 -58.9908767
H 13.0661721 -2.2754632 -59.0090904
C 14.5182136 -3.1102436 -57.6789229
H 15.4998097 -3.5591397 -57.8229977
C 13.7014467 -4.2044761 -56.9428997
O 13.6238671 -4.1604091 -55.7146881
C 14.714941 -1.81596 -56.8514598
H 13.7756341 -1.2643591 -56.8431172
H 14.9534945 -2.0737327 -55.819355
C 15.8229594 -0.873091 -57.3541501
H 15.6404761 -0.6146515 -58.3959849
C 15.8120489 0.4127376 -56.5303236
H 16.028931 0.1980959 -55.4826737
H 14.8309305 0.8833728 -56.593059
H 16.5554212 1.1109018 -56.9145674
C 17.2136708 -1.5018124 -57.2217831
H 17.3755269 -1.8389114 -56.1975999
H 17.9750578 -0.7631874 -57.470298
H 17.3151205 -2.3433035 -57.9050428
N 13.0948036 -5.214763 -57.6151661
C 12.2053006 -6.1721577 -56.9514439
H 11.3888186 -5.644905 -56.4617025
C 12.9249789 -7.0386965 -55.9075064
O 12.3195788 -7.4085346 -54.9111168
C 11.6162382 -7.0363963 -58.0682371
H 11.3764525 -8.0460584 -57.7312578
H 10.7270492 -6.5549761 -58.4702904
C 13.2765494 -5.6211828 -59.0043124
H 12.7053484 -4.9577645 -59.6524598
H 14.33053 -5.6213029 -59.2835882
C 12.7120005 -7.0340446 -59.1282169
H 12.3119336 -7.2235629 -60.1252091
H 13.4796364 -7.7662537 -58.8743209
N 14.2193922 -7.3309605 -56.0875591
H 14.6758111 -6.9951267 -56.9187628
C 15.0103271 -8.0990672 -55.112049
H 14.434692 -8.9910839 -54.8655987
C 15.2070223 -7.332927 -53.7964256
O 15.0766997 -7.9116657 -52.717904
C 16.3497863 -8.552893 -55.7413196
H 16.1067111 -9.1238207 -56.6393262
C 17.2457079 -7.3784555 -56.1790683
H 17.6104389 -6.833258 -55.3064419

H 16.7096291 -6.695959 -56.8363059
H 18.1109972 -7.7622999 -56.7213065
C 17.1758962 -9.4774166 -54.8192359
H 18.0864798 -9.7670527 -55.3455807
H 17.4716894 -8.935527 -53.9193684
C 16.4479887 -10.7630839 -54.4053729
H 16.0929498 -11.2919093 -55.2902479
H 15.605773 -10.5327737 -53.7532482
H 17.1389073 -11.4070984 -53.8607548
N 15.3935547 -6.0141343 -53.87863
H 15.4044782 -5.57695 -54.785056
C 15.4989416 -5.1573755 -52.7005961
H 16.1260958 -5.6549509 -51.9609007
C 14.1275042 -4.979588 -52.0493859
O 14.0041547 -5.2410058 -50.8625342
C 16.1529791 -3.8128398 -53.0612849
H 15.5453294 -3.3096024 -53.8136461
H 16.1749842 -3.1849001 -52.1697456
C 17.5888162 -3.9565456 -53.5969701
H 17.9604463 -2.9636573 -53.8583359
H 17.5825816 -4.5500643 -54.513358
C 18.5239075 -4.5941443 -52.560969
O 18.5957494 -5.8428049 -52.4662396
O 19.14147 -3.8633975 -51.7551244
N 13.0718701 -4.7526557 -52.834066
H 13.246795 -4.5814834 -53.8192455
C 11.6674641 -4.7573474 -52.3915481
H 11.5110988 -3.9424476 -51.6838844
C 11.2638044 -6.055375 -51.6705936
O 10.7000723 -5.9916155 -50.5815018
C 10.8225721 -4.4658085 -53.6373247
H 11.2703018 -4.9725609 -54.4847155
H 10.8980451 -3.3996945 -53.8162933
C 9.3400869 -4.8422281 -53.6032018
O 8.5960027 -4.4336681 -52.6834481
O 8.8625046 -5.4577929 -54.5871172
N 11.6917214 -7.2238873 -52.1577427
H 12.1378338 -7.2256039 -53.0701622
C 11.4803921 -8.5084706 -51.4804795
H 10.4192842 -8.5902366 -51.2439924
C 12.2581264 -8.616735 -50.1576347
O 11.7173718 -9.1125472 -49.1729519
C 11.8590517 -9.6523727 -52.4351888
H 12.9441046 -9.6885066 -52.5437256
H 11.4310558 -9.4628766 -53.4176041
C 11.3583046 -11.0199242 -51.9562242

H 11.7482812 -11.2164564 -50.9570363
H 11.7634607 -11.7769659 -52.6281464
S 9.5513028 -11.2041661 -51.9296248
C 9.4280423 -12.9497713 -51.463499
H 8.3802362 -13.2482151 -51.4521366
H 9.8585708 -13.0948903 -50.4720088
H 9.9653893 -13.5614913 -52.1881289
N 13.4992124 -8.1155487 -50.1013757
H 13.8751445 -7.7085546 -50.9531837
C 14.3462607 -8.0992723 -48.8917458
H 14.3261654 -9.0880359 -48.4302966
C 13.8414349 -7.109273 -47.8321094
O 13.8696885 -7.4088854 -46.6438106
C 15.7803139 -7.7771225 -49.3386629
H 16.0728206 -8.4953921 -50.1072518
H 15.78842 -6.7853287 -49.7877521
C 16.8146607 -7.8333564 -48.2019743
H 16.5459291 -7.1366515 -47.4067893
H 16.8280532 -8.8437172 -47.7908348
C 18.2187971 -7.4873246 -48.7193819
H 18.4184365 -8.0829343 -49.6128579
H 18.9536731 -7.7499724 -47.9570584
C 18.3487833 -5.992413 -49.0458139
H 18.4319706 -5.4217815 -48.1165061
H 17.4451803 -5.6561907 -49.5619324
N 19.5031294 -5.7293883 -49.9309239
H 20.3954323 -5.9966135 -49.5549232
H 19.5174901 -4.749167 -50.2346865
H 19.3464276 -6.1609146 -50.848426
N 13.3322727 -5.9630881 -48.2710839
H 13.3916351 -5.7966786 -49.2700395
C 12.6752334 -4.9259791 -47.4652083
H 13.3066394 -4.639705 -46.6204001
C 11.3514425 -5.4659331 -46.907204
O 11.0801258 -5.3692947 -45.708615
C 12.463281 -3.6936637 -48.3766892
H 12.0638674 -4.0566426 -49.3241162
C 11.3939495 -2.735481 -47.8251092
H 11.5699158 -2.5482408 -46.7698325
H 10.4027027 -3.1721763 -47.9439616
H 11.4167651 -1.795219 -48.3641339
C 13.8090055 -3.0011842 -48.7056933
H 14.1943818 -2.5034339 -47.8276267
H 14.5553025 -3.7476582 -48.9678682
C 13.7415405 -1.9940272 -49.8643963
H 13.1388901 -1.128049 -49.5957665

H 13.3172396 -2.4665957 -50.7493734
H 14.7489847 -1.6485579 -50.0979748
N 10.5569209 -6.1251239 -47.7575478
H 10.8058184 -6.1502801 -48.7417588
C 9.3406062 -6.8155937 -47.3499945
H 8.687436 -6.0980664 -46.8515223
C 9.6451199 -7.9320667 -46.3426887
O 8.9789309 -7.9857565 -45.3181334
C 8.6090851 -7.3439192 -48.5900398
H 9.2347282 -8.0646011 -49.1166765
H 8.3743305 -6.5167723 -49.2610325
H 7.6825766 -7.8315087 -48.286617
N 10.6967849 -8.7327052 -46.5459848
H 11.1877227 -8.6812436 -47.4322827
C 11.1410652 -9.7413254 -45.5766499
H 10.3095113 -10.4188421 -45.3765601
C 11.5417736 -9.1181235 -44.2316211
O 11.0027769 -9.5243077 -43.2065835
C 12.2879134 -10.5821685 -46.1574739
H 13.1383181 -9.9410307 -46.3900585
H 11.9539012 -11.054893 -47.0827182
C 12.7241101 -11.6616207 -45.1622198
O 13.5224159 -11.3337599 -44.252894
O 12.2018212 -12.793503 -45.265959
N 12.3829782 -8.0769071 -44.2118859
H 12.8033958 -7.7736289 -45.0842588
C 12.8418208 -7.4756884 -42.9545116
H 13.20788 -8.2795145 -42.315361
C 11.7105569 -6.7843906 -42.179033
O 11.5105007 -7.0762136 -40.995856
C 14.0107534 -6.5158331 -43.2086705
H 14.8199205 -7.0620265 -43.6953675
H 13.6867322 -5.7238326 -43.8863098
C 14.5352889 -5.889822 -41.9505213
C 15.3513898 -6.4920806 -41.0560262
H 15.7532765 -7.4912347 -41.1718495
N 15.5335855 -5.6654725 -39.9629168
H 16.0897329 -5.9158626 -39.160673
C 14.8148192 -4.49376 -40.0872147
C 14.6321954 -3.3866221 -39.242576
H 15.1032791 -3.3500841 -38.2735638
C 13.819259 -2.3236695 -39.676404
H 13.6551516 -1.4597462 -39.0434416
C 13.2231146 -2.3758491 -40.9490175
H 12.61592 -1.5480294 -41.2963996
C 13.413713 -3.4925454 -41.785488

H 12.9690569 -3.4997165 -42.7718436
C 14.1985453 -4.5894945 -41.3712467
N 10.9043925 -5.9527161 -42.8492866
H 11.1134384 -5.7630804 -43.8240366
C 9.7438538 -5.2921988 -42.2212211
H 10.0844229 -4.773692 -41.3238945
C 8.6828352 -6.2978319 -41.7797415
O 8.1475363 -6.1684389 -40.6765001
C 9.0796136 -4.2487583 -43.1304959
H 8.177974 -3.8895772 -42.6371761
C 9.9720763 -3.0467225 -43.4104666
H 10.8669468 -3.3593763 -43.9445606
H 10.2661708 -2.5763767 -42.4714854
H 9.4314004 -2.3171273 -44.0117579
O 8.7181591 -4.8066588 -44.3726664
H 9.5496823 -4.9479732 -44.8577988
N 8.4358032 -7.3581704 -42.5633335
H 8.8963046 -7.4314515 -43.466883
C 7.5723311 -8.4495715 -42.1268535
H 6.6350766 -8.0155664 -41.7795424
C 8.1700975 -9.1517994 -40.9247922
O 7.4758181 -9.174459 -39.9330543
C 7.2448461 -9.4620492 -43.2330206
H 8.1639771 -9.7685181 -43.7320877
H 6.8107539 -10.3548548 -42.779457
C 6.239774 -8.9211213 -44.2597927
H 6.2060887 -9.6067299 -45.1067549
H 6.5649357 -7.9487869 -44.6272582
C 4.8301142 -8.7907822 -43.6962677
O 4.0185217 -9.6936336 -43.7800733
N 4.4373901 -7.6329512 -43.2092787
H 3.4549186 -7.5555601 -42.9669857
H 5.0762198 -6.8618807 -43.140931
N 9.4273277 -9.6006942 -40.9284614
H 9.9686587 -9.4839231 -41.780828
C 10.0214635 -10.412134 -39.8567427
H 9.3084547 -11.1990181 -39.609992
C 10.253828 -9.6601678 -38.5291229
O 10.2250454 -10.2893742 -37.4666527
C 11.2944858 -11.0964365 -40.3945858
H 11.8618845 -11.5134649 -39.5643313
H 11.9244235 -10.3674132 -40.904203
C 10.9694015 -12.2521054 -41.3360884
O 10.0165345 -12.9890257 -41.1245311
N 11.7728694 -12.4937022 -42.343769
H 11.4995019 -13.1733068 -43.0362986

H 12.499172 -11.8467642 -42.6449554
N 10.2929531 -8.3240194 -38.537167
H 10.3624872 -7.8437779 -39.4304161
C 10.2075646 -7.5261957 -37.311539
H 10.9370343 -7.9083139 -36.5955343
C 8.8173012 -7.6078404 -36.6366541
O 8.7224682 -7.6976754 -35.4119872
C 10.5911494 -6.0827475 -37.6571958
H 9.8848154 -5.6694188 -38.3793169
H 11.5923603 -6.0574983 -38.0909011
H 10.5793001 -5.471428 -36.7549126
N 7.7281362 -7.648853 -37.4093658
H 7.8464977 -7.6620426 -38.4175533
C 6.3615845 -7.7065981 -36.8837774
H 6.2525433 -6.8732437 -36.1891368
C 5.9991926 -8.9928891 -36.0905116
O 5.3798625 -8.8602907 -35.0312186
C 5.3875801 -7.4605357 -38.0417105
H 5.317507 -8.3466364 -38.6717451
H 5.7360643 -6.6360806 -38.6607618
H 4.3964641 -7.2320804 -37.651936
N 6.3537059 -10.227742 -36.5201682
C 6.0921003 -11.4546801 -35.7992746
H 5.0595046 -11.4728627 -35.4552056
C 7.0141917 -11.5868196 -34.5825291
O 6.5442037 -12.022288 -33.5387212
C 6.2944393 -12.5878507 -36.8162142
H 6.5796654 -13.5239315 -36.3340225
H 5.3790469 -12.7278175 -37.3944718
C 6.9710996 -10.6108314 -37.7595143
H 6.2311127 -10.4906961 -38.55036
H 7.8621115 -10.0271148 -37.9027458
C 7.3852351 -12.0701274 -37.7139111
H 7.3632158 -12.5333561 -38.7012058
H 8.3603036 -12.1870356 -37.2408137
N 8.2600128 -11.0993172 -34.6473682
H 8.5974122 -10.7351963 -35.529587
C 9.1638845 -11.0324667 -33.493428
H 9.2559856 -12.0264496 -33.0531509
C 8.6246533 -10.1030979 -32.3918366
O 8.5898426 -10.4804776 -31.2234502
C 10.5463395 -10.5906214 -33.9929249
H 10.9400176 -11.3588206 -34.6604347
H 10.4349591 -9.6795107 -34.5821898
C 11.5645544 -10.3336599 -32.8982995
C 12.1676299 -11.4139774 -32.226674

H 11.9007571 -12.4282594 -32.4853161
C 13.115578 -11.1764532 -31.2147571
H 13.5750579 -12.007349 -30.6985902
C 13.464218 -9.8578058 -30.873536
H 14.194 -9.6750549 -30.0974929
C 12.8624226 -8.7769348 -31.541472
H 13.1324783 -7.7636193 -31.281157
C 11.9107974 -9.0133361 -32.5496745
H 11.4482941 -8.1777601 -33.0568941
N 8.0603085 -8.9495652 -32.7631566
H 8.1434311 -8.665565 -33.7337643
C 7.4168677 -8.0211198 -31.8271501
H 7.9214617 -8.1082216 -30.8649975
C 5.9315139 -8.326438 -31.5456683
O 5.2535744 -7.4801421 -30.961817
C 7.5941536 -6.574922 -32.324633
H 7.124499 -6.4941971 -33.3064549
H 7.0538484 -5.9068789 -31.6550215
C 9.0136829 -6.0386909 -32.3987931
C 9.4463363 -5.359517 -33.5561945
H 8.7912065 -5.2787379 -34.4129759
C 10.7433545 -4.8150995 -33.6203321
H 11.075635 -4.3075551 -34.5142454
C 11.6148391 -4.9453172 -32.5180674
O 12.8717441 -4.435554 -32.5709646
H 13.0345853 -3.9454995 -33.3800472
C 11.174962 -5.6029107 -31.3511048
H 11.8453005 -5.6829437 -30.507972
C 9.8769556 -6.144496 -31.2893049
H 9.5563376 -6.6474406 -30.3872537
N 5.3871766 -9.481238 -31.956426
H 6.0033601 -10.1538206 -32.4006634
C 3.9274016 -9.6951489 -32.0875758
H 3.5788743 -9.0167038 -32.8656313
C 3.101727 -9.3795924 -30.8315989
O 1.9946272 -8.8568 -30.9597617
C 3.6184426 -11.1199467 -32.5819641
H 4.1373467 -11.2775613 -33.5237867
C 4.0262775 -12.229422 -31.6076246
H 3.4924745 -12.148523 -30.6645446
H 5.0959923 -12.1726016 -31.405786
H 3.820187 -13.1996915 -32.0601955
O 2.2341245 -11.2757929 -32.8256162
H 2.1031661 -11.0808149 -33.7675107
N 3.6312656 -9.6287895 -29.6257628
H 4.5649006 -10.0097299 -29.5872965

C 2.9405287 -9.3177676 -28.3687039
H 1.9221669 -9.6969241 -28.445873
C 2.8276563 -7.8017421 -28.1507249
O 1.7167741 -7.2833488 -28.1555535
C 3.6362754 -10.0368499 -27.1973246
H 3.6353806 -11.1098669 -27.3948457
H 4.6745595 -9.7054275 -27.1474315
C 2.978396 -9.7839054 -25.8233671
H 2.9966347 -8.7178673 -25.6005677
C 1.5310448 -10.2812004 -25.7697653
H 1.4817257 -11.3377576 -26.0293787
H 0.9096754 -9.6938931 -26.4446131
H 1.1425322 -10.1385847 -24.7604909
C 3.7720642 -10.514862 -24.7416685
H 3.7563083 -11.5903824 -24.9153329
H 3.3368301 -10.2987848 -23.7649057
H 4.8026104 -10.1599394 -24.7388168
N 3.9616382 -7.0915666 -28.1298216
H 4.8305866 -7.5964343 -28.1841824
C 4.0327294 -5.6286734 -27.9990707
H 3.5475118 -5.3373421 -27.066531
C 3.3223149 -4.9052738 -29.1588863
O 2.7887892 -3.8112358 -29.0000078
C 5.522688 -5.2420034 -27.9073454
H 5.9417744 -5.7125461 -27.0159418
H 6.0387796 -5.6508386 -28.77724
C 5.8148372 -3.7290084 -27.839953
H 5.4302001 -3.2507583 -28.740477
C 5.2052008 -3.0369301 -26.6265279
H 5.5270028 -3.5308572 -25.7079944
H 4.1165442 -3.0902357 -26.6670599
H 5.5002705 -1.9890255 -26.5938106
C 7.3263404 -3.5054407 -27.8182893
H 7.7487717 -3.9367071 -26.9075049
H 7.5460048 -2.4366408 -27.8259105
H 7.7977514 -3.9704279 -28.681937
N 3.3240981 -5.4922525 -30.3565642
H 3.8200164 -6.3736162 -30.4481514
C 2.6217824 -4.9657189 -31.5266407
H 2.8835245 -3.9148142 -31.6417372
C 1.0943839 -5.029999 -31.3514762
O 0.4108227 -4.0473604 -31.6319395
C 3.127867 -5.7370606 -32.7582961
H 4.209855 -5.607543 -32.8206349
H 2.9292394 -6.7975108 -32.6056175
C 2.5111026 -5.3318787 -34.1077532

H 1.4532277 -5.5947615 -34.1197899
C 2.6686143 -3.8435188 -34.4097948
H 3.7203921 -3.5715168 -34.3494023
H 2.0853599 -3.2554452 -33.704076
H 2.2981698 -3.6320755 -35.4139741
C 3.226156 -6.095398 -35.2194507
H 4.2560045 -5.7514367 -35.3113525
H 2.7083162 -5.937219 -36.1650524
H 3.2404669 -7.1588567 -34.9842874
N 0.5617721 -6.1321914 -30.8100106
H 1.1854655 -6.8955181 -30.565335
C -0.8774119 -6.2932057 -30.5294087
H -1.4450707 -5.8133694 -31.3273322
C -1.3387857 -5.5989339 -29.2472027
O -2.4972833 -5.2107496 -29.1799714
C -1.2259879 -7.7857952 -30.5059816
H -0.5395652 -8.3161481 -29.8425807
H -2.2423577 -7.9080194 -30.1304602
C -1.1550235 -8.3653732 -31.9235204
H -0.1595575 -8.2283015 -32.3457716
H -1.8592111 -7.825137 -32.5503861
C -1.517416 -9.8499764 -31.9535904
H -1.7789138 -10.1101266 -32.9786297
H -2.3926915 -10.0247935 -31.3275004
N -0.3815104 -10.6888054 -31.5472774
H 0.4686067 -10.5891001 -32.0922565
C -0.2819976 -11.5284697 -30.5397689
N 0.7987728 -12.2399241 -30.4453776
H 0.9213052 -12.9191248 -29.7195572
H 1.4926744 -12.1243647 -31.1788985
N -1.2140364 -11.691179 -29.6413828
H -2.0524106 -11.1461476 -29.7056909
H -1.0837421 -12.3425586 -28.8909044
N -0.4359914 -5.4137744 -28.291648
H 0.4565919 -5.878876 -28.4106519
C -0.6121594 -4.6637386 -27.0405899
H -1.5484464 -4.9448234 -26.5574101
C -0.6419177 -3.1442925 -27.2699944
O -1.4719593 -2.454789 -26.6849815
C 0.5604857 -5.0831334 -26.1452238
H 0.4619065 -6.1502756 -25.9371324
H 1.4675594 -4.941078 -26.7285774
C 0.7664583 -4.3591921 -24.8161269
H -0.1072872 -4.5150072 -24.1788573
H 0.8875321 -3.2879945 -24.9917974
C 2.035639 -4.9226683 -24.1581527

O 1.8929473 -5.6917077 -23.1831504
O 3.1384206 -4.6239782 -24.6732221
N 0.1917402 -2.6286023 -28.1822229
H 0.9142711 -3.2259607 -28.5641261
C 0.1832659 -1.2150978 -28.5658876
H 0.0037262 -0.6137299 -27.6722889
C -0.9246664 -0.8558517 -29.5768898
O -1.3427934 0.2986789 -29.6289793
C 1.5712314 -0.8568396 -29.111109
H 1.7755088 -1.4407739 -30.0082925
H 2.3275326 -1.0683264 -28.3535512
H 1.5991124 0.2052893 -29.3567835
N -1.3690744 -1.8151607 -30.399589
H -0.9570034 -2.7323259 -30.3107115
C -2.4122034 -1.6197109 -31.4130676
H -2.8401389 -0.6237023 -31.2850044
C -3.5838278 -2.5891446 -31.1989809
O -4.4984085 -2.2893246 -30.4387788
C -1.8158924 -1.6685056 -32.833723
H -1.3145361 -2.624073 -32.987596
H -2.63415 -1.6141187 -33.553035
C -0.8409829 -0.5505884 -33.1512774
C -1.3096435 0.649862 -33.7150379
H -2.3647469 0.7833783 -33.9051926
C 0.531843 -0.7025696 -32.8802269
H 0.8864084 -1.6149005 -32.4260483
C -0.4110316 1.6879453 -34.01348
H -0.7806818 2.6123909 -34.4308109
C 1.4300197 0.3388147 -33.1757978
H 2.4760809 0.2347951 -32.9481857
C 0.9602995 1.5313944 -33.7512922
H 1.6466328 2.3356076 -33.9776088
N -3.6205628 -3.7132496 -31.9236746
H -2.8479066 -3.9034345 -32.5436994
C -4.6046085 -4.7861899 -31.7255437
H -4.6477881 -5.0219769 -30.6606922
C -4.2176418 -6.0541322 -32.4919207
O -3.4466607 -6.006089 -33.4557384
C -6.0082382 -4.3507814 -32.1811528
H -6.3304691 -3.4699273 -31.624698
H -6.7175425 -5.152142 -31.9705056
O -6.0325689 -4.0692292 -33.5679786
H -5.899994 -3.1163384 -33.6518697
N -4.8741413 -7.1737827 -32.163381
H -5.4716506 -7.1487112 -31.3494907
C -4.8665038 -8.4037354 -32.9751168

H -3.8421984 -8.7622554 -33.024553
C -5.296619 -8.1459118 -34.4291164
O -4.6251069 -8.5946963 -35.3565651
C -5.7191938 -9.4992887 -32.2851475
H -5.2617698 -9.6962688 -31.3135578
C -7.1749464 -9.0643969 -32.0189038
H -7.7180113 -8.9252308 -32.9539572
H -7.2157387 -8.1483551 -31.4309694
H -7.6857446 -9.8419113 -31.4478146
C -5.7591062 -10.8389807 -33.0521625
H -6.3486961 -11.5529072 -32.4750014
H -6.2623358 -10.6958331 -34.0075932
C -4.3868889 -11.4732058 -33.3065014
H -3.86465 -11.6184485 -32.3614838
H -3.7929475 -10.8453952 -33.9693974
H -4.5241808 -12.4437534 -33.7840225
N -6.3197801 -7.3081212 -34.6459337
H -6.7867577 -6.9174776 -33.8407924
C -6.8108968 -6.9429317 -35.9822531
H -6.9295796 -7.8697976 -36.5438468
C -5.8010042 -6.0946841 -36.7703672
O -5.7534856 -6.2049544 -37.9993011
C -8.1929232 -6.2678168 -35.828368
H -8.8936588 -6.9935093 -35.4113768
H -8.1035006 -5.4462128 -35.1147961
C -8.795135 -5.6932521 -37.1238768
H -9.7559562 -5.2332397 -36.8894435
H -8.1411857 -4.8942535 -37.4703831
C -9.0162358 -6.7421972 -38.2346595
H -9.9843958 -7.2211919 -38.076221
H -8.2596339 -7.5233973 -38.1830747
C -8.9707821 -6.1540308 -39.6519625
H -9.7832162 -5.4324325 -39.7803459
H -9.1312743 -6.9749907 -40.3575952
N -7.6582374 -5.5172426 -39.9201159
H -7.5238449 -4.6979612 -39.3466649
H -7.5232324 -5.260878 -40.8933727
H -6.8930147 -6.1433565 -39.6825251
N -4.9831557 -5.2914209 -36.0868715
H -5.1114733 -5.2316311 -35.0833861
C -3.8621657 -4.5557308 -36.6755995
H -4.2125805 -3.9476157 -37.5101316
H -3.4303927 -3.8987074 -35.9202956
C -2.7652385 -5.4947951 -37.177779
O -2.4814048 -5.5085537 -38.373279
N -2.2677174 -6.3806718 -36.3070245

H -2.5945535 -6.342489 -35.3479413
C -1.2607822 -7.3958332 -36.6512525
H -0.3445124 -6.8925585 -36.961776
C -1.6939054 -8.2984128 -37.8176158
O -0.9311845 -8.4717807 -38.7673839
C -0.9587692 -8.2239316 -35.3945171
H -0.5126998 -7.5591821 -34.6545479
H -1.9003645 -8.5918536 -34.9852958
C -0.0205586 -9.403772 -35.5957312
C -0.476939 -10.7237244 -35.4080804
H -1.509788 -10.9114615 -35.1526382
C 0.4018223 -11.8087914 -35.5828742
H 0.0411055 -12.8215519 -35.4605813
C 1.7396798 -11.5814567 -35.9510346
H 2.4073452 -12.4177367 -36.1126058
C 2.197147 -10.2669613 -36.1448257
H 3.2167878 -10.0866761 -36.4561706
C 1.3158525 -9.1847377 -35.975059
H 1.6594669 -8.1812815 -36.1674815
N -2.9508894 -8.7602365 -37.8162996
H -3.5180262 -8.6013591 -36.9897401
C -3.5164419 -9.5705426 -38.9055008
H -2.8830213 -10.4489155 -39.0412821
C -3.4884375 -8.8191223 -40.2441416
O -3.0854456 -9.4032043 -41.2444796
C -4.9353343 -10.0606912 -38.5229828
H -5.4930771 -9.2156787 -38.1147458
C -5.7199256 -10.6008875 -39.7362459
H -5.1748012 -11.428586 -40.1934969
H -5.8704642 -9.8203191 -40.4808494
H -6.7059161 -10.950001 -39.4339102
C -4.8187313 -11.1576382 -37.4374046
H -4.3660146 -12.0503218 -37.8713733
H -4.1598068 -10.814504 -36.6407699
C -6.1553173 -11.5460725 -36.7923586
H -6.7955629 -12.0580817 -37.5094256
H -6.6616767 -10.6550823 -36.4198857
H -5.9674939 -12.2254155 -35.9610021
N -3.8323925 -7.5248185 -40.2879394
H -4.0656676 -7.0556824 -39.4218744
C -3.7691065 -6.745843 -41.5391746
H -4.3005425 -7.2994218 -42.3139513
C -2.3393446 -6.5519942 -42.0526116
O -2.1145754 -6.6247537 -43.2621331
C -4.4332741 -5.3718712 -41.3703427
H -3.8537605 -4.6109815 -41.8970195

H -4.4733346 -5.1006908 -40.3138927
O -5.7412933 -5.3987764 -41.9212897
H -5.6390313 -5.3843889 -42.8827614
N -1.3861787 -6.3126124 -41.1495095
H -1.6547347 -6.2826085 -40.1714205
C 0.0158965 -6.0425797 -41.481886
H 0.0577829 -5.3112942 -42.2894989
C 0.740751 -7.3012983 -41.9896076
O 1.4825599 -7.2298511 -42.9719905
C 0.6993255 -5.4537121 -40.2332656
H 0.5759614 -6.161702 -39.4119538
H 1.768021 -5.3583632 -40.4270006
C 0.1460188 -4.0790425 -39.7977291
H -0.9410754 -4.0968931 -39.8068985
C 0.5881962 -3.7787531 -38.3651539
H 1.6688825 -3.8436922 -38.2817429
H 0.1358835 -4.4987693 -37.6855946
H 0.2728934 -2.7773283 -38.0815491
C 0.5838749 -2.9467471 -40.7300837
H 1.6679734 -2.8669288 -40.751094
H 0.1714852 -2.0035095 -40.3724266
H 0.2150466 -3.1247907 -41.7383577
N 0.4550266 -8.4611916 -41.3890336
H -0.1379686 -8.4416052 -40.564342
C 0.9646168 -9.7595951 -41.8364496
H 2.0196674 -9.6433663 -42.0892642
C 0.2485841 -10.2596498 -43.1035624
O 0.8987943 -10.7616169 -44.0158858
C 0.8455888 -10.7511466 -40.6638834
H 1.3523783 -10.326376 -39.7955445
H -0.210257 -10.8640917 -40.4110157
C 1.4357562 -12.1477194 -40.9424942
H 0.9147976 -12.6038002 -41.7839374
C 2.9345315 -12.1074372 -41.2461209
H 3.4682867 -11.5756565 -40.4587843
H 3.1048157 -11.6059846 -42.1990644
H 3.3258448 -13.1209115 -41.3293987
C 1.228386 -13.036948 -39.7157139
H 1.7534838 -12.6171902 -38.8578697
H 1.6078131 -14.0384338 -39.9180822
H 0.1647686 -13.1057515 -39.4881301
N -1.0698244 -10.0583188 -43.2209953
H -1.5784126 -9.6705319 -42.4337335
C -1.8288138 -10.4543792 -44.4133578
H -1.6406428 -11.512455 -44.6026739
C -1.3632127 -9.6903915 -45.6534104

O -1.0336879 -10.3147171 -46.6612399
C -3.3368109 -10.2801862 -44.1613091
H -3.5384081 -9.2595103 -43.8397539
H -3.6148275 -10.9493552 -43.3461491
C -4.2354917 -10.6192243 -45.3629775
H -5.2387681 -10.8120393 -44.981554
H -3.8764807 -11.5469091 -45.8110439
S -4.390968 -9.3657168 -46.6781462
C -5.1799099 -8.0079527 -45.7729399
H -5.4383667 -7.2116522 -46.4701612
H -4.4931662 -7.6138861 -45.0242914
H -6.0870682 -8.3705426 -45.2881865
N -1.2836702 -8.3547994 -45.597801
H -1.5351508 -7.8696527 -44.7427603
C -0.9788531 -7.5879528 -46.8043397
H -1.457325 -8.1034868 -47.6360249
C 0.521404 -7.5756896 -47.1451478
O 0.8637811 -7.5102657 -48.3264741
C -1.6430715 -6.2032923 -46.748782
H -1.1355764 -5.5871202 -46.0089988
H -2.6802938 -6.3144597 -46.4385527
C -1.6394516 -5.5151375 -48.1094585
N -0.9709235 -4.389742 -48.2157069
H -0.4685428 -4.0199304 -47.4270163
H -0.9501478 -3.9346266 -49.1103779
O -2.2486604 -5.9590541 -49.0785727
N 1.416904 -7.7516498 -46.1678263
H 1.0980991 -7.8154289 -45.2078644
C 2.8375119 -7.9866019 -46.4363406
H 3.1969886 -7.2710589 -47.1758242
H 3.3911814 -7.8211393 -45.5225839
C 3.1279278 -9.4043741 -46.945777
O 3.8298141 -9.5493483 -47.9453507
N 2.447086 -10.4225332 -46.4101552
H 1.9315207 -10.2624063 -45.5490469
C 2.4854058 -11.7979805 -46.9139969
H 3.5152832 -12.1562071 -46.9087821
H 1.8931677 -12.4278665 -46.2504679
C 1.9239234 -11.9295401 -48.3328251
O 2.5932635 -12.4826701 -49.2022689
N 0.7929469 -11.2777152 -48.628854
H 0.267872 -10.8632915 -47.8641561
C 0.2683398 -11.1228987 -49.9938683
H 0.0573556 -12.1067264 -50.4128921
C 1.2826057 -10.4351154 -50.919821
O 1.4660857 -10.8427349 -52.0678355

C -1.03995 -10.3126457 -49.9511539
H -1.8060414 -10.9034882 -49.4476874
H -0.8743164 -9.4136616 -49.3588149
C -1.5543657 -9.8836418 -51.2950987
C -1.1187229 -8.7991881 -51.9758444
H -0.3563124 -8.1147227 -51.6133052
N -1.6791194 -8.7882528 -53.2363242
H -1.3736528 -8.1625098 -53.9676252
C -2.5438482 -9.8457334 -53.4186151
C -3.3411695 -10.2526051 -54.4992947
H -3.3476281 -9.6931163 -55.422582
C -4.1539985 -11.3894695 -54.358604
H -4.801853 -11.6994674 -55.1695271
C -4.1314936 -12.1172815 -53.1550939
H -4.7589197 -12.9925633 -53.044334
C -3.3101688 -11.7105559 -52.0842815
H -3.3046579 -12.2804389 -51.1652787
C -2.499787 -10.5592805 -52.1831049
N 1.96428 -9.3993704 -50.4253499
H 1.7809878 -9.1156849 -49.4709382
C 2.9636726 -8.654149 -51.2011413
H 2.5137098 -8.3680451 -52.1510126
C 4.1751778 -9.5252683 -51.5380323
O 4.584636 -9.5468988 -52.6991712
C 3.3562456 -7.3613529 -50.4714756
H 3.7076416 -7.5770001 -49.4645344
C 4.4220107 -6.5580398 -51.2039862
H 4.0784879 -6.3762013 -52.2179027
H 5.3645424 -7.1059384 -51.2128172
H 4.574357 -5.6062331 -50.6915057
O 2.2203371 -6.5288334 -50.4111686
H 1.6945377 -6.8265429 -49.6442254
N 4.6517677 -10.3483214 -50.5991615
H 4.2697926 -10.2894075 -49.6593257
C 5.6772822 -11.3580867 -50.8568759
H 6.5451977 -10.8700353 -51.3041686
C 5.1923812 -12.4341222 -51.8490049
O 5.8968642 -12.7354913 -52.8110289
C 6.1067911 -11.9647842 -49.5145566
H 5.2580115 -12.4435631 -49.0255728
H 6.499734 -11.1841373 -48.8617151
H 6.8863492 -12.7084471 -49.6781218
N 3.9534366 -12.9249302 -51.7086818
H 3.4267844 -12.6496469 -50.8836684
C 3.334672 -13.8997941 -52.6291921
H 3.9737587 -14.7791788 -52.6745089

C 3.2311635 -13.3581777 -54.0601941
O 3.501972 -14.0879971 -55.0132972
C 1.9611062 -14.3607064 -52.0920771
H 1.4005589 -13.4924383 -51.7508018
C 1.0987965 -15.094003 -53.1308964
H 1.6582642 -15.9270397 -53.5595551
H 0.8015677 -14.4086898 -53.9226587
H 0.1946828 -15.4764729 -52.6554527
C 2.1541222 -15.3188572 -50.9094302
H 2.6359165 -16.2380737 -51.2437664
H 1.18814 -15.559973 -50.4658729
H 2.7765934 -14.8574841 -50.1444956
N 2.9105554 -12.073782 -54.2549198
H 2.6628376 -11.510579 -53.4444772
C 2.8382215 -11.4848562 -55.603466
H 2.5477263 -12.2731829 -56.294507
C 4.1932573 -11.0523229 -56.1735234
O 4.3050569 -11.0014922 -57.3976465
C 1.7500749 -10.3972881 -55.6684607
H 1.973677 -9.6224873 -54.932472
H 1.7766916 -9.9369756 -56.657886
C 0.3181127 -10.9321047 -55.4242165
H 0.2277581 -11.265099 -54.390261
H -0.3748739 -10.1024204 -55.5677658
C -0.0987512 -12.0894509 -56.3560436
H 0.544746 -12.9544503 -56.17014
H 0.0153415 -11.7777132 -57.39766
C -1.5577073 -12.5187353 -56.1029819
H -2.2269318 -11.6802266 -56.3325773
H -1.6736438 -12.7561101 -55.0366384
N -1.920309 -13.7026939 -56.930816
H -1.8494992 -13.5031153 -57.9228767
H -2.903941 -13.9547134 -56.8042702
H -1.3493329 -14.5134468 -56.71273
N 5.239108 -10.8811623 -55.358782
H 5.0832237 -10.9063656 -54.3572045
C 6.6252368 -10.7490509 -55.8568326
H 6.6136699 -10.1725473 -56.7835268
C 7.23075 -12.111499 -56.2120809
O 7.8163342 -12.237301 -57.2830009
C 7.5248285 -9.9796415 -54.8686851
H 7.4639628 -10.4501565 -53.8877875
C 8.9905939 -9.944826 -55.3227672
H 9.0568977 -9.5997377 -56.3555774
H 9.4372889 -10.9357012 -55.2428839
H 9.5579733 -9.2635562 -54.6896495

C 7.0793244 -8.5186274 -54.7449023
H 7.2214826 -8.0117064 -55.6995769
H 7.6884329 -8.0243809 -53.9867521
H 6.0354079 -8.4688468 -54.4529373
N 7.0278598 -13.1595392 -55.4024952
H 6.5409429 -13.011564 -54.5230944
C 7.6271667 -14.4858677 -55.6559459
H 8.6634613 -14.3031979 -55.9438593
C 6.9906641 -15.2279986 -56.8419336
O 7.6716931 -15.9879257 -57.5225639
C 7.6575082 -15.3317962 -54.3638388
H 7.9124323 -14.6589608 -53.5431137
C 6.2996944 -15.9707711 -54.0400428
H 6.0816508 -16.7851927 -54.7321286
H 5.5262014 -15.2171777 -54.1221082
H 6.2972443 -16.3543042 -53.0201916
C 8.777694 -16.3911983 -54.4438692
H 8.5331507 -17.1303691 -55.2074872
H 9.7068405 -15.8989223 -54.7355244
C 9.0351373 -17.1189259 -53.1182629
H 8.173149 -17.7267722 -52.8436591
H 9.2387219 -16.3944539 -52.3289923
H 9.8999306 -17.7735446 -53.2299662
N 5.7387061 -14.9039989 -57.1868191
H 5.2127234 -14.3222172 -56.5485866
C 5.1062204 -15.3009733 -58.4534771
H 5.3874356 -16.3318692 -58.6680816
C 5.6030433 -14.4874707 -59.672003
O 5.0849158 -14.6599443 -60.773668
C 3.5735904 -15.2270815 -58.2905951
H 3.3168007 -14.2373792 -57.910768
H 3.1057086 -15.3358337 -59.269651
C 2.9775319 -16.3018904 -57.3585176
H 3.4899355 -16.2918303 -56.3990126
C 1.4957103 -15.9962792 -57.1210999
H 0.9610944 -15.9934025 -58.0710052
H 1.4152946 -15.0272253 -56.6348142
H 1.0717324 -16.755584 -56.4636815
C 3.0815974 -17.7101515 -57.947436
H 2.616133 -17.7444842 -58.9324024
H 2.5847047 -18.4241014 -57.2906702
H 4.1275589 -18.0041337 -58.0324987
N 6.5584693 -13.5695965 -59.4840437
H 6.9635998 -13.4805897 -58.559394
C 7.1419561 -12.7294521 -60.533019
H 8.0300305 -12.2428227 -60.1296168

H 7.4428545 -13.3604889 -61.3694312
C 6.2113295 -11.6372743 -61.0664694
O 6.4668133 -11.1007314 -62.1414297
N 5.0989407 -11.3521821 -60.3807085
H 4.9813281 -11.7997437 -59.4822739
C 4.011173 -10.5016823 -60.8819061
H 4.0372055 -10.488733 -61.9692609
C 4.1458654 -9.0360507 -60.4550618
O 4.0311999 -8.1487806 -61.2999311
C 2.6640571 -11.1114873 -60.4638078
H 1.8926477 -10.351034 -60.4842377
H 2.730934 -11.4831747 -59.4414438
C 2.2535011 -12.2657448 -61.3868724
H 1.4591474 -12.8339579 -60.902344
H 3.1069454 -12.932034 -61.5231857
S 1.6498686 -11.7327541 -63.0139356
C 1.6214996 -13.3280329 -63.8756168
H 1.2174771 -13.1904539 -64.8799789
H 0.9970689 -14.0358844 -63.3303468
H 2.6341481 -13.7267153 -63.9450518
N 4.4403578 -8.7747006 -59.1755103
H 4.5741277 -9.5745046 -58.565276
C 4.7721047 -7.4432695 -58.6461105
H 4.2329189 -6.6929209 -59.2287529
C 6.2628089 -7.1513591 -58.8255713
O 7.1132365 -8.0286716 -58.7190631
C 4.3124856 -7.3225627 -57.1776623
H 3.2218766 -7.3201566 -57.1538076
H 4.664482 -8.1940433 -56.6248092
C 4.8413101 -6.0586761 -56.4669405
H 5.9269408 -6.1201353 -56.3940316
H 4.5835999 -5.1738691 -57.0510368
C 4.2767258 -5.8952971 -55.0518431
H 4.2724669 -6.8599056 -54.5417714
H 3.2469937 -5.5465683 -55.1283597
C 5.0870934 -4.8828455 -54.2236432
H 4.4787623 -4.580427 -53.3658758
H 5.2958632 -3.991815 -54.8235343
N 6.3573003 -5.466491 -53.7188249
H 6.2395348 -6.3113772 -53.1868018
H 6.9070721 -4.8226841 -53.1439003
H 7.0461657 -5.6510471 -54.4521823
N 6.5831059 -5.8796803 -59.0166479
H 5.8235267 -5.2105063 -59.113986
C 7.9375657 -5.3227778 -59.0310328
H 8.6143732 -5.9856498 -58.4939189

C 7.9299697 -3.9753104 -58.3016906
O 6.8849011 -3.3299543 -58.2056804
C 8.3940536 -5.2114097 -60.498702
H 7.5989185 -4.7461053 -61.083704
C 9.6867695 -4.4393521 -60.7534294
H 10.461601 -4.7634189 -60.0624864
H 9.5092652 -3.3712309 -60.637248
H 10.0261984 -4.6212524 -61.7728531
O 8.6164189 -6.5095718 -60.9963761
H 8.1255677 -7.1366062 -60.4489059
N 9.0868513 -3.5097747 -57.8288001
H 9.9133854 -4.0836863 -57.9019445
C 9.2842826 -2.0970461 -57.484942
H 8.3344924 -1.5820776 -57.6283965
C 10.2397767 -1.4588863 -58.488381
O 11.1822076 -2.0878065 -58.9748673
C 9.6723067 -1.8988096 -55.9998239
H 9.4745394 -2.8358148 -55.4851521
C 11.1589555 -1.5434698 -55.8111287
H 11.3819425 -0.5661663 -56.2441685
H 11.7740098 -2.3086833 -56.2769605
H 11.4226248 -1.4985324 -54.7557414
C 8.7636256 -0.8305709 -55.3496209
H 8.9602225 0.1445593 -55.7991688
H 7.7226656 -1.0931424 -55.5430241
C 8.9349449 -0.7232145 -53.8274753
H 9.9006709 -0.2819557 -53.5826193
H 8.8562241 -1.7099889 -53.3708842
H 8.1532925 -0.0869728 -53.4164581
N 9.9517901 -0.2103244 -58.8094482
H 9.1682783 0.231535 -58.3373574
C 10.654951 0.6360141 -59.7608788
H 11.4393476 0.0802286 -60.2761536
C 11.3137825 1.7707113 -58.9834636
O 10.6744127 2.3280577 -58.0948429
C 9.5994166 1.0937026 -60.7929687
H 8.6089785 1.028997 -60.3467226
H 9.6158493 0.3827572 -61.6188321
C 9.6756245 2.4856417 -61.3678588
N 9.6560584 2.7683857 -62.7304314
C 9.6304442 4.1041051 -62.8320208
H 9.6677495 4.647484 -63.7669766
N 9.599696 4.6686446 -61.6145544
H 9.6754936 5.6576488 -61.4159251
C 9.6037052 3.6646771 -60.6808175
H 9.5692624 3.7963986 -59.6106405

N 12.5267095 2.1509732 -59.3842939
H 13.0058659 1.5797279 -60.0714761
C 13.1036285 3.4786336 -59.1419166
H 12.3791542 4.0801703 -58.6065819
C 13.4003518 4.137936 -60.4980748
O 13.9804095 3.4895145 -61.3751488
C 14.3486196 3.3947989 -58.2404563
H 14.0355429 3.0345998 -57.2602812
H 15.0443904 2.6671256 -58.661884
C 15.0998792 4.7290328 -58.0561606
H 15.5972644 4.9762276 -58.9978091
H 15.8861092 4.5829474 -57.3125827
C 14.2028056 5.9034645 -57.6227446
O 14.1451209 6.8963968 -58.3833069
O 13.5576779 5.8054743 -56.5593021
N 13.0453446 5.4126846 -60.6712948
H 12.6425479 5.9066621 -59.8759317
C 13.3632583 6.2062371 -61.8533931
H 14.1139036 5.6662568 -62.4167197
C 13.9496037 7.5757594 -61.5001384
O 13.3110616 8.3851175 -60.8299668
C 12.1357501 6.3519771 -62.752082
H 11.3121412 6.7960327 -62.1946084
H 11.8710934 5.3727228 -63.1411174
H 12.3807769 6.9994993 -63.5956822
N 15.109189 7.8745898 -62.0873356
H 15.4773862 7.2053572 -62.7563538
C 15.8432781 9.1316037 -61.9126432
H 15.2448618 9.8123746 -61.30726
C 16.0561624 9.7692783 -63.2824116
O 16.4188304 9.0839867 -64.2349318
C 17.1881392 8.8969502 -61.188147
H 17.8076121 8.2561194 -61.8169794
C 16.9764192 8.1765845 -59.8366834
H 16.3622953 8.7964619 -59.1838709
H 16.4432474 7.2414061 -60.0042166
C 17.9261826 10.2389898 -60.9959842
H 17.3004281 10.929227 -60.4298202
H 18.1645092 10.6855659 -61.9607414
H 18.8686102 10.0894265 -60.4738383
C 18.2749524 7.8099776 -59.110778
H 18.7708445 8.7035771 -58.7336403
H 18.9412565 7.2714474 -59.7855677
H 18.0370505 7.1684199 -58.2616609
N 15.8801458 11.0823937 -63.3805037
H 15.5331748 11.5872138 -62.5688742

C 15.9623305 11.817571 -64.6442913
H 16.7952455 11.4316431 -65.2351664
C 16.2306405 13.3158319 -64.4223685
O 16.001996 13.8407697 -63.3256233
C 14.6780471 11.5721577 -65.4405948
H 14.5614297 12.3592306 -66.1828294
H 14.8143697 10.6397859 -65.9771394
C 13.3981278 11.4233831 -64.5967226
H 13.2409175 12.3213791 -64.0054631
H 13.4843556 10.5777558 -63.912386
C 12.2010246 11.1445692 -65.486179
O 12.4220538 10.9836834 -66.7062945
O 11.0711677 11.0577901 -64.952999
N 16.7127389 14.0282412 -65.4533237
H 16.8577185 13.566903 -66.349382
C 16.9273486 15.4814323 -65.3898288
H 16.3871383 15.8476574 -64.5193038
C 16.3447654 16.2645858 -66.5817988
O 16.1558116 15.7419953 -67.6835179
C 18.4109936 15.8096423 -65.1287159
H 18.8261429 15.0930979 -64.4212154
H 18.4804463 16.7986531 -64.677849
C 19.2719235 15.817902 -66.3805842
O 19.9812442 14.8726451 -66.670495
N 19.2629312 16.8803734 -67.1552989
H 19.7115882 16.7659892 -68.0471681
H 18.6938739 17.6945154 -66.9492704
N 16.1171737 17.5560555 -66.3461034
H 16.3244087 17.9218972 -65.4216912
C 15.6409335 18.5213459 -67.3311437
H 15.1563779 17.9880668 -68.1515813
C 16.8207639 19.3019001 -67.9050657
O 17.814395 19.5196466 -67.2156087
C 14.6025547 19.470826 -66.7156745
H 15.0457816 19.9951369 -65.8663646
H 13.7532703 18.8943344 -66.3687152
C 14.1049765 20.4776596 -67.7549781
O 14.0497 21.6808959 -67.4357038
O 13.8939207 20.0301459 -68.9036003
N 16.6932574 19.7127438 -69.1587056
H 15.7361606 19.6535177 -69.5221682
C 17.6417525 20.4891035 -69.9664808
H 18.1709276 19.7718774 -70.5775385
C 18.7397635 21.2279062 -69.1911158
O 19.6163392 20.5303848 -68.6231238
O 18.7905354 22.4280729 -69.0989548

C 16.8603575 21.4153103 -70.9236658
H 16.3055296 22.1240006 -70.3041713
H 17.5800612 21.9809911 -71.516076
C 15.8643249 20.7213124 -71.8783228
H 15.1037594 20.1933291 -71.2956912
C 15.1458514 21.7861056 -72.7164382
H 15.8579213 22.3362843 -73.3312233
H 14.6219113 22.4788679 -72.0561909
H 14.4058411 21.3071409 -73.3609419
C 16.5435476 19.7199673 -72.8232848
H 17.329409 20.2094041 -73.398468
H 15.8002973 19.3095617 -73.5106352
H 16.9602655 18.8885803 -72.2557724

Supplementary Data 5. Cartesian coordinates for TePeNMT homology model with bound SAM.

N -2.1385667 21.7753992 -15.9844882
H -2.8881317 22.3451992 -15.612073
H -2.4187043 21.4412796 -16.898962
H -1.952445 20.9940613 -15.3721405
C -0.8983927 22.6008534 -16.1048954
H -1.0805973 23.3575681 -16.8709468
C 0.33849 21.7897622 -16.5659561
O 1.2553163 22.327476 -17.1874333
C -0.6053818 23.3116885 -14.7771474
H 0.3668547 23.80077 -14.8479421
H -0.5567763 22.5711725 -13.9778747
C -1.639947 24.3835703 -14.4160168
H -1.396285 24.7758699 -13.426708
H -2.6273748 23.9290305 -14.3587376
S -1.7285502 25.7740286 -15.5733039
C -2.9534829 26.7844932 -14.6928753
H -3.1342375 27.7045542 -15.2488035
H -2.5798462 27.0332981 -13.6984196
H -3.8891334 26.2323095 -14.5953778
N 0.3673335 20.4886197 -16.2657559
H -0.4109708 20.1080689 -15.7405706
C 1.3938537 19.5859753 -16.7771625
H 1.3580658 19.6103407 -17.8672226
H 2.3753087 19.9422459 -16.4680576
C 1.2496557 18.1385969 -16.3224611
O 0.7986647 17.8537127 -15.2154758
N 1.7628938 17.2390447 -17.1522057
H 2.1121366 17.5432004 -18.0501647
C 2.0288584 15.8382482 -16.8338954
H 1.9028599 15.7027836 -15.7604402
C 3.4957816 15.5076051 -17.1394012
O 4.119702 16.1812749 -17.9609391
C 0.9990926 14.9223526 -17.5032517
H 1.2572628 13.8928438 -17.2749979
H 0.0423459 15.1358366 -17.026009
C 0.8149397 15.0455348 -19.020348
H 1.6157817 14.5050359 -19.5288645
H 0.8699863 16.0954136 -19.3176114
C -0.5554566 14.4754363 -19.4180847
O -1.297864 15.2053148 -20.1119062
O -0.875195 13.3478418 -18.9747065
N 4.1061184 14.7016468 -16.2632362
H 3.5442127 14.1102083 -15.6715657

C 5.5629684 14.6122266 -16.0983634
H 6.0446756 14.8186955 -17.0600251
C 5.9695982 13.2129132 -15.6736854
O 5.3463653 12.639753 -14.786269
C 6.0594875 15.5900786 -15.0182406
H 5.5966651 15.3285324 -14.0641537
H 7.1389711 15.4636292 -14.915048
C 5.7688522 17.0627619 -15.3210483
H 6.199365 17.3109927 -16.2919241
H 4.6914859 17.2284646 -15.351451
C 6.3694499 17.9615862 -14.2310701
H 7.4516987 17.8220036 -14.192695
H 5.9514537 17.6743696 -13.2638997
C 6.0413128 19.4366002 -14.4861744
H 6.4275873 20.029751 -13.6516003
H 4.9522862 19.5499365 -14.5026758
N 6.6253332 19.9107507 -15.7656037
H 7.6331593 19.818804 -15.7546612
H 6.3843249 20.8792368 -15.933991
H 6.2683797 19.3550584 -16.5356419
N 7.106455 12.7593095 -16.1705568
H 7.4956956 13.2759729 -16.9574346
C 7.5639831 11.383838 -16.027934
H 6.7220861 10.7521143 -15.7410336
C 8.6256294 11.3080201 -14.9245199
O 9.6775993 11.9537076 -14.9983873
C 8.0743326 10.8706348 -17.3888252
H 8.9570019 11.4416081 -17.6791626
H 8.3787829 9.8302572 -17.2664366
C 7.0169986 10.9370419 -18.5172246
H 6.1405201 10.371064 -18.1914962
H 7.409044 10.4393792 -19.4038394
C 6.5974706 12.3719046 -18.896483
O 7.4785217 13.2681533 -18.9084406
O 5.3745041 12.6072441 -18.9936412
N 8.3366279 10.5700175 -13.8495991
H 7.428984 10.1185224 -13.7997984
C 9.2537472 10.4628666 -12.7165948
H 9.411715 11.4704627 -12.3299929
C 10.6243327 9.9036971 -13.153139
O 10.6986872 8.9385328 -13.9069108
C 8.6037136 9.6350831 -11.6036772
H 8.4204313 8.619799 -11.9600852
H 7.655759 10.0896643 -11.3114457
H 9.2647124 9.595986 -10.737355
N 11.703524 10.5367378 -12.6721267

H 11.5296608 11.3030954 -12.0418231
C 13.1142625 10.3142897 -13.0702562
H 13.6516325 11.146085 -12.6173586
C 13.4243279 10.5119185 -14.5675031
O 14.4681525 10.0599575 -15.0339048
C 13.7765064 9.0619071 -12.4395625
H 14.845682 9.122573 -12.6445486
C 13.6265139 9.0675566 -10.9118445
H 12.5803642 8.9423089 -10.6318339
H 14.0018322 10.0070241 -10.506304
H 14.2061243 8.2486758 -10.485005
C 13.2962742 7.7038875 -12.9652798
H 12.2890587 7.4911381 -12.6080123
H 13.9637118 6.9192992 -12.6112727
H 13.2950871 7.7094786 -14.0550769
N 12.605294 11.3349009 -15.2378421
H 11.7369915 11.5814565 -14.7805248
C 12.7314974 11.833814 -16.6111805
H 11.8724211 12.4921825 -16.7495073
C 12.6154246 10.7757957 -17.7237248
O 13.1625965 9.6728211 -17.644985
C 13.9716254 12.728998 -16.7542726
H 14.876663 12.1276992 -16.6566322
H 13.9650252 13.5017634 -15.9855672
H 13.9695782 13.2008787 -17.7373648
N 11.9102878 11.1457732 -18.7984009
H 11.4901366 12.0606529 -18.8311174
C 11.6113386 10.2161504 -19.8849453
H 11.4683386 9.2575284 -19.3922061
C 12.7697246 10.041093 -20.8749029
O 13.4841971 10.9883786 -21.2103005
C 10.2956217 10.5834444 -20.6018401
H 9.5642602 10.8737195 -19.8541547
H 10.4613373 11.4376453 -21.2598264
C 9.7172444 9.4047201 -21.4196476
H 10.350257 9.2350742 -22.2929309
H 8.7362979 9.7002982 -21.7986705
C 9.5899082 8.089336 -20.6183328
O 8.4815808 7.7830257 -20.1380566
O 10.6205886 7.3866881 -20.4498584
N 12.8901662 8.8305339 -21.4204041
H 12.2017952 8.1247666 -21.1470783
C 13.7998552 8.4972353 -22.5092701
H 14.1771403 9.4236569 -22.9434863
C 13.0399482 7.741936 -23.6088019
O 12.2666108 6.8259826 -23.3460136

C 14.9936922 7.7225667 -21.9245386
H 15.5173435 8.3745069 -21.2235556
H 14.6001376 6.8863099 -21.3570609
C 16.0029798 7.189281 -22.9591753
H 15.4878043 6.568367 -23.6890142
C 16.7306364 8.3210255 -23.6896215
H 17.2504768 8.9574053 -22.9726436
H 16.0235264 8.9250527 -24.2541046
H 17.4549132 7.90003 -24.3877077
C 17.0509741 6.3236455 -22.2584349
H 17.5894864 6.9110609 -21.5149428
H 17.7563171 5.9395483 -22.9960493
H 16.5630007 5.4766885 -21.778768
N 13.2962296 8.1160457 -24.8611726
H 13.9627613 8.8548464 -25.0049569
C 12.7297022 7.4720004 -26.0467334
H 11.6552424 7.6561114 -26.0668372
C 12.963355 5.9483119 -26.0268958
O 14.0825531 5.4964434 -25.7932219
C 13.3669937 8.1475173 -27.2671617
H 13.1403425 9.2139322 -27.2266732
H 14.4513269 8.049724 -27.187207
C 12.9656811 7.6189319 -28.6307211
C 12.1494424 8.3971049 -29.4761888
H 11.769383 9.3526089 -29.1454154
C 11.8565891 7.9520163 -30.7798108
H 11.2562024 8.555452 -31.4472005
C 12.3673248 6.7178848 -31.2374061
O 12.1160781 6.2857259 -32.4969706
H 11.5991475 6.9488647 -33.0185918
C 13.1697677 5.9340538 -30.3847744
H 13.5492799 4.9896701 -30.7459872
C 13.4827043 6.3907892 -29.091865
H 14.1273567 5.791667 -28.4621886
N 11.8951892 5.1788313 -26.2599184
H 11.0304658 5.6427895 -26.4800882
C 11.8426092 3.7036502 -26.2137578
H 10.7811114 3.4599108 -26.2631227
C 12.3180866 3.0493723 -24.893297
O 12.588105 1.8486882 -24.8229072
C 12.4769853 3.1028593 -27.4818833
H 13.5610504 3.0598399 -27.3602348
H 12.2582179 3.7523311 -28.3311342
C 11.9188234 1.7098089 -27.8129528
O 10.6734795 1.5608478 -27.7641107
O 12.7303627 0.8270589 -28.169355

N 12.3786583 3.8077385 -23.7892188
H 12.1567416 4.7960764 -23.8565606
C 12.7236866 3.2518266 -22.4780661
H 13.5786598 2.5902866 -22.6181944
C 11.5733328 2.4149249 -21.9209309
O 10.5345585 2.9233827 -21.5059758
C 13.1586237 4.3717878 -21.5322934
H 14.0067916 4.8753226 -21.9927235
H 12.3398627 5.0838206 -21.4074609
C 13.5708913 3.8411374 -20.1519667
H 12.6903613 3.4453154 -19.6452956
H 14.3110593 3.0473288 -20.2626016
C 14.1595742 4.9730826 -19.3074994
H 13.477531 5.8273082 -19.3386531
H 15.1255757 5.265841 -19.7198963
C 14.3246592 4.5251755 -17.8558798
H 14.9647772 3.6393135 -17.8237746
H 13.3343658 4.2459127 -17.4769636
N 14.894404 5.6206571 -17.034387
H 15.8026652 5.9022239 -17.3743342
H 14.9585333 5.3479973 -16.0632593
H 14.281514 6.4323719 -17.0835563
N 11.8145313 1.1129027 -21.796752
H 12.636759 0.7428888 -22.2469702
C 10.915173 0.206206 -21.075752
H 9.9261211 0.3116122 -21.5233934
C 10.7879049 0.6022152 -19.5986625
O 11.7565775 1.0128309 -18.9601126
C 11.3345322 -1.270057 -21.22834
H 10.6296722 -1.8783407 -20.6606041
C 11.2418011 -1.7098514 -22.6941182
H 11.9608367 -1.1650701 -23.3087444
H 10.2395993 -1.5174528 -23.0780593
H 11.4468521 -2.7773516 -22.7753339
C 12.7458793 -1.5768076 -20.7095885
H 13.4907476 -1.0077613 -21.2656222
H 12.9570051 -2.6395233 -20.8284199
H 12.8214824 -1.3296274 -19.6506744
N 9.6090573 0.3641778 -19.0190877
H 8.8636369 0.042936 -19.61778
C 9.3030641 0.4995601 -17.5765515
H 8.2498576 0.2378134 -17.4888618
C 9.3464409 1.9102799 -16.9589934
O 9.2888891 2.018324 -15.7386553
C 10.0341763 -0.545724 -16.7016046
H 9.6693134 -0.4676256 -15.6770594

C 9.7794235 -1.9797159 -17.169852
H 10.215899 -2.1450359 -18.1540049
H 8.7079161 -2.1740235 -17.2032576
H 10.239578 -2.6699839 -16.4624216
O 11.4299275 -0.3613229 -16.691399
H 11.6667592 0.1678767 -17.4727227
N 9.2779939 2.9909397 -17.7450031
H 9.3809 2.8980378 -18.7455964
C 8.8924366 4.3186524 -17.2226461
H 9.484174 4.5459203 -16.3361664
C 7.3950813 4.3363773 -16.8420819
O 6.54833 4.1563548 -17.7157283
C 9.1737252 5.4060425 -18.2724269
H 8.8412563 6.3707971 -17.8859208
H 8.6096819 5.1895781 -19.1816057
O 10.5517975 5.4957348 -18.5958262
H 10.5894134 6.1754387 -19.3178923
N 7.0367162 4.4791013 -15.5515566
H 7.7637049 4.4744686 -14.8485576
C 5.6335974 4.372326 -15.0850094
H 5.0070584 4.6318068 -15.9400818
C 5.0839267 5.3356751 -13.9496445
O 4.1802507 4.8921545 -13.2441249
C 5.3501324 2.8742188 -14.7954481
H 5.6601937 2.2734319 -15.6495461
H 4.2770818 2.7385852 -14.6680957
C 6.0481724 2.3356799 -13.5563096
O 6.9355157 2.9397106 -12.9802825
N 5.6839717 1.1536912 -13.1130988
H 6.1721809 0.8278604 -12.2984982
H 4.9434468 0.6468731 -13.5561149
N 5.4209234 6.6581869 -13.7954432
H 6.1313111 7.0377768 -14.4037847
C 4.6727308 7.6690857 -12.8730958
H 3.6722803 7.2716519 -12.7031805
H 5.1665915 7.6425688 -11.902207
C 4.4605241 9.2667223 -13.2399755
O 5.4811072 9.6663177 -13.812121
N 3.2536409 10.1109433 -12.9872607
H 2.6474233 9.6614132 -12.3206553
C 2.3771526 11.4343133 -13.6922496
H 3.1658733 12.0715482 -14.0792353
C 1.0505375 12.8198719 -12.978889
O 0.738086 12.1140384 -12.0071596
C 1.9540762 10.4352165 -14.9544058
H 0.9329988 10.1097298 -14.746017

C 2.7417697 9.1177858 -15.5124699
H 3.7821969 9.3932306 -15.6797871
H 2.6711718 8.3582525 -14.7352443
C 1.9181844 11.302737 -16.1856385
H 2.9290002 11.590086 -16.4899791
H 1.3394983 12.1952761 -15.9637221
H 1.4171187 10.8031861 -17.0150758
C 2.3461044 8.3062146 -16.7841532
H 1.2838958 8.043668 -16.7411571
H 2.9411495 7.3919643 -16.8207085
H 2.5328117 8.8854332 -17.695951
N 0.2333932 14.4003043 -13.1834479
H 0.7437184 14.7653437 -13.9704046
C -1.2123799 15.955379 -12.9597387
H -1.0028175 16.3292374 -13.960482
C -2.5312399 15.1695335 -13.1010222
H -2.3258053 14.1092915 -13.2484796
H -3.0618878 15.2689053 -12.1555772
C -3.4175086 15.6977339 -14.2585728
H -3.2432872 16.7657087 -14.3985759
C -3.0783778 14.9714564 -15.5675978
H -3.2811317 13.903087 -15.4809576
H -2.0269104 15.1037593 -15.8111044
H -3.6757972 15.3801559 -16.3812018
C -4.9110054 15.512951 -13.9555661
H -5.1400087 14.4586235 -13.8201664
H -5.5040244 15.9052164 -14.7807417
H -5.172629 16.0607214 -13.0540348
C -2.0596333 17.9254177 -12.3430272
O -1.3832877 18.2475852 -11.374691
N -3.271554 19.2249776 -12.5340521
H -3.8017849 18.9469697 -13.3433045
C -4.1853409 20.9390259 -11.9780666
H -4.1201797 20.8378689 -10.8941409
C -5.8142116 21.9798425 -12.0685304
O -6.4577917 21.990306 -13.1238488
C -3.3553264 22.2522131 -12.3368714
H -2.7508435 21.9810248 -13.199742
H -3.9431639 23.1195955 -12.6376299
C -2.3935577 22.6639575 -11.2141059
H -1.5875927 23.2551301 -11.6539897
H -1.9417954 21.7737149 -10.7710014
C -3.1027948 23.4921229 -10.1365626
O -4.1338848 23.0043489 -9.6189633
O -2.6283562 24.6174618 -9.8712537
N -6.4475046 23.0719258 -11.2698861

H -5.8681384 23.316696 -10.4654281
C -7.9445496 23.8116708 -11.2481781
H -8.5313975 23.1447577 -11.8786809
C -8.4505552 25.3183528 -11.7282815
O -7.9562339 26.3541144 -11.2608184
C -8.5827225 23.7319299 -9.8142739
H -8.3098143 22.7644516 -9.3910452
H -8.1237068 24.4929663 -9.1820639
C -10.1128287 23.8553926 -9.699581
H -10.4295997 24.8665053 -9.9608487
H -10.3884539 23.7049408 -8.6526486
C -10.8425833 22.8299823 -10.5754585
O -10.9583405 23.1023724 -11.793048
O -11.2574013 21.7859035 -10.0265513
N -9.602907 25.5151302 -12.4609812
H -10.1970189 24.6947314 -12.6038089
C -10.0093518 26.7968804 -13.139427
H -9.6033406 27.6153906 -12.5465371
C -11.5373224 27.0770783 -13.3129338
O -12.3430523 26.1660776 -13.4651874
C -9.3158021 26.7963794 -14.5218177
H -8.2652178 26.5407625 -14.3723088
H -9.7616841 25.9988297 -15.1187812
C -9.3656862 28.1006173 -15.3396433
H -10.3980501 28.3527717 -15.5746801
C -8.7151959 29.2767986 -14.6053596
H -7.6855865 29.0272619 -14.3458923
H -9.2616884 29.5103425 -13.6946444
H -8.721151 30.1580805 -15.2468748
C -8.6202634 27.902131 -16.6598573
H -7.5714266 27.6723762 -16.4704162
H -8.6904418 28.808201 -17.2612677
H -9.0736317 27.0808258 -17.214682
N -11.9412789 28.3572898 -13.4297045
H -11.2467902 29.0828588 -13.3712445
C -13.3321225 28.7589476 -13.708797
H -13.9922764 28.0787705 -13.1669539
C -13.6973559 28.668285 -15.20173
O -13.2212429 29.4599915 -16.0166546
C -13.5976231 30.1744778 -13.1694646
H -13.3339687 30.2032292 -12.1112647
H -12.9526566 30.8857408 -13.6884859
C -15.0439728 30.6091374 -13.3172145
C -16.0007793 30.2075446 -12.3654889
H -15.7026398 29.6095012 -11.5164327
C -17.3497155 30.5734271 -12.5229045

H -18.084102 30.2600018 -11.7942258
C -17.7459906 31.3401123 -13.632773
H -18.784204 31.6156339 -13.7569758
C -16.7930155 31.742311 -14.5848431
H -17.0984245 32.326439 -15.4416514
C -15.4440982 31.3771052 -14.4276294
H -14.7165289 31.6734974 -15.1713742
N -14.6326318 27.780819 -15.5396291
H -14.9521194 27.1150618 -14.8471413
C -15.2390964 27.6886577 -16.8666391
H -15.735075 28.6312197 -17.0994544
H -14.4624243 27.5134405 -17.6111349
C -16.2666536 26.5596064 -16.957317
O -16.3953891 25.7524785 -16.0401148
N -16.9764725 26.4878918 -18.0819378
H -16.8289854 27.176356 -18.8044725
C -17.9575033 25.4408391 -18.3831007
H -17.7899232 24.5827215 -17.7304426
C -17.7792012 24.9927546 -19.8397808
O -17.7959146 25.8188285 -20.750926
C -19.3742105 25.9740925 -18.1133224
H -19.4072767 26.3626889 -17.0943744
H -19.5911057 26.7896238 -18.8053619
C -20.4418095 24.8784784 -18.2588574
H -20.2170614 24.0779836 -17.5490798
H -20.3816661 24.4589508 -19.2655484
C -21.873747 25.3850101 -18.0153021
O -22.0329252 26.4946208 -17.4568844
O -22.8044396 24.6367793 -18.3888393
N -17.5576422 23.6953514 -20.0649298
H -17.6282623 23.0479037 -19.293315
C -17.3269048 23.1256682 -21.396853
H -17.6295227 23.8524687 -22.1539443
C -18.173276 21.8705255 -21.6133325
O -18.4492201 21.1344351 -20.6665456
C -15.8303326 22.833808 -21.5971231
H -15.5031065 22.1074362 -20.8521036
H -15.2664698 23.755845 -21.4523805
C -15.5235885 22.3038595 -22.9769314
N -16.0573017 22.8083599 -24.1656877
C -15.6125519 22.0028993 -25.140073
H -15.854249 22.1181749 -26.1888167
N -14.8449621 21.02673 -24.6277346
H -14.429788 20.2755673 -25.1579691
C -14.7816493 21.1953241 -23.2621255
H -14.2791179 20.561661 -22.5447457

N -18.5185024 21.5818415 -22.8701808
H -18.1432063 22.1738025 -23.6009505
C -19.2288161 20.3661674 -23.257331
H -19.9116933 20.1067755 -22.4457962
C -18.2431347 19.2015468 -23.4437471
O -17.5034608 19.1367934 -24.4310812
C -20.0753688 20.6536823 -24.5101802
H -20.7622502 21.4721288 -24.2876809
H -19.4111275 20.9769363 -25.3134978
C -20.8899068 19.4416545 -25.0031907
H -20.2061397 18.632868 -25.252551
C -21.8900833 18.9305006 -23.9653095
H -22.5664824 19.7301243 -23.6638583
H -21.3716057 18.543429 -23.0898099
H -22.4721546 18.1090986 -24.387707
C -21.6558409 19.8178908 -26.2704486
H -22.3670983 20.6156827 -26.0549008
H -22.1966695 18.9460329 -26.6408658
H -20.9593117 20.149503 -27.0399621
N -18.305586 18.2264973 -22.5363312
H -18.9848484 18.3084584 -21.7879638
C -17.4987295 17.0022879 -22.56464
H -16.4445225 17.2767669 -22.5352472
C -17.7136686 16.1851165 -23.8479637
O -16.7324037 15.7061853 -24.4089253
C -17.8183622 16.1676056 -21.3118248
H -17.6392762 16.7679502 -20.4189096
H -17.1541192 15.3031549 -21.279748
C -19.2469521 15.6902349 -21.2993199
N -20.3555822 16.5291505 -21.1770985
C -21.409785 15.8002387 -21.5780685
H -22.404937 16.1941265 -21.7444035
N -21.0189659 14.5690479 -21.940586
H -21.5461555 13.999855 -22.611493
C -19.6585309 14.4792161 -21.7690455
H -19.0246639 13.6887216 -22.1457658
N -18.9342631 16.1416122 -24.3967885
H -19.7139306 16.4659898 -23.8383305
C -19.2306073 15.4622993 -25.6698397
H -18.9191364 14.4191048 -25.5943322
C -18.4740459 16.0915514 -26.8531044
O -17.994577 15.3816032 -27.732199
C -20.7422481 15.4827005 -25.9605596
H -20.9140981 15.0341016 -26.9402223
H -21.0845436 16.5175317 -26.0055241
C -21.5715667 14.7083567 -24.9337949

O -21.1731487 13.5796127 -24.57134
O -22.5721805 15.2767049 -24.4473625
N -18.2496141 17.4112082 -26.8323886
H -18.5716361 17.9329869 -26.0288096
C -17.4531297 18.1047217 -27.8489587
H -17.7977212 17.8135617 -28.8424323
H -17.5879439 19.1789559 -27.7304308
C -15.956921 17.7942072 -27.7454374
O -15.2888172 17.6280449 -28.7627028
N -15.439025 17.6201509 -26.5247796
H -16.0609943 17.7043393 -25.7294654
C -14.0743246 17.1266898 -26.3031017
H -13.3826248 17.7038566 -26.9186169
C -13.9256189 15.6534948 -26.724003
O -12.9564171 15.2990751 -27.3953963
C -13.702554 17.3422062 -24.8291262
H -13.7609572 18.4063683 -24.6038623
H -14.431762 16.8391893 -24.1953113
C -12.3186415 16.8515113 -24.4543145
C -11.189853 17.6482056 -24.7279804
H -11.3040053 18.609059 -25.2098121
C -9.9051194 17.1911998 -24.3777829
H -9.0312386 17.792005 -24.5808524
C -9.7460546 15.9368061 -23.7535044
O -8.5019852 15.5094553 -23.4137942
H -8.5099345 14.6569228 -22.9779248
C -10.8775819 15.1370117 -23.4808996
H -10.7593813 14.1747477 -23.0085044
C -12.161335 15.5959257 -23.8337612
H -13.0275541 14.9793858 -23.6353157
N -14.9268359 14.8201123 -26.423841
H -15.7014839 15.176822 -25.8741997
C -14.9828394 13.4197593 -26.8408227
H -14.0769896 12.9181517 -26.4986893
C -15.0310294 13.2711907 -28.3685764
O -14.316041 12.4302505 -28.9112801
C -16.1878629 12.7409781 -26.1724631
H -16.0987507 12.8425083 -25.0898935
H -17.1015123 13.2506904 -26.4752072
C -16.3194695 11.2716038 -26.5208461
C -15.679925 10.2960364 -25.7306851
H -15.1296517 10.5903453 -24.8487413
C -15.7427155 8.9385754 -26.1018896
H -15.2470437 8.1817352 -25.5143806
C -16.4327316 8.5582085 -27.2723703
O -16.4601685 7.2539103 -27.6505408

H -16.8895214 7.1591939 -28.5014005
C -17.087672 9.5346431 -28.0532165
H -17.603424 9.2540987 -28.9595907
C -17.035941 10.887789 -27.6716777
H -17.5222426 11.6367727 -28.2851494
N -15.7781968 14.1209507 -29.0785084
H -16.4213335 14.735133 -28.58639
C -15.8302485 14.0991621 -30.5458371
H -16.023302 13.0723458 -30.857743
C -14.4935853 14.5210283 -31.1785911
O -13.967663 13.81329 -32.0333803
C -16.994066 14.9750367 -31.0424011
H -17.90762 14.6865268 -30.519727
H -16.7819786 16.0183928 -30.8031416
C -17.238316 14.8508303 -32.5555673
H -16.3290961 15.1177248 -33.0992785
H -18.0038016 15.5779021 -32.8375267
C -17.6986865 13.4395358 -32.9540066
O -18.9264795 13.2539991 -33.097649
O -16.8225467 12.5548429 -33.0888592
N -13.837982 15.5729289 -30.6701081
H -14.2892367 16.1201696 -29.9440593
C -12.4898729 15.9648231 -31.1351682
H -12.5346458 16.1337827 -32.2113024
C -11.4694612 14.8360737 -30.9194148
O -10.6875397 14.5295424 -31.8238719
C -12.0510367 17.2817301 -30.4618222
H -12.2135606 17.2075124 -29.385962
C -10.5745282 17.6261432 -30.7001145
H -10.360449 17.6415137 -31.7693089
H -9.9326989 16.8899406 -30.2164525
H -10.3471552 18.6032195 -30.2737645
C -12.8768666 18.454303 -31.009272
H -12.6844 18.5843449 -32.0745506
H -12.6143381 19.3701809 -30.4802161
H -13.9403605 18.2682826 -30.8668087
N -11.5418689 14.1369177 -29.7810758
H -12.2068198 14.4377171 -29.0741423
C -10.7457172 12.934077 -29.5229422
H -9.6880299 13.1653715 -29.6509979
H -10.9128238 12.6231638 -28.4919327
C -11.1015855 11.7534031 -30.4368387
O -10.2052046 11.0525872 -30.9091058
N -12.3806552 11.5703557 -30.7665667
H -13.0669852 12.1981408 -30.3596135
C -12.8830576 10.4904933 -31.6335773

H -12.4536585 9.5465298 -31.299618
C -12.4546029 10.6930136 -33.0856704
O -11.8379689 9.7967565 -33.662114
C -14.4115139 10.3664975 -31.5108397
H -14.8837716 11.3195435 -31.7430037
C -15.0075931 9.281508 -32.405127
H -14.497612 8.3332624 -32.2385487
H -14.9184434 9.5764776 -33.4497101
H -16.0681325 9.1738969 -32.1753859
O -14.7191285 9.9990169 -30.1858573
H -14.6611214 10.8041515 -29.6445041
N -12.6061399 11.9050074 -33.6278135
H -13.1277568 12.5991368 -33.0943308
C -12.1108057 12.2899864 -34.96069
H -12.5626445 11.6330904 -35.7039491
C -10.5924574 12.1086371 -35.0606404
O -10.1118234 11.5017037 -36.0194828
C -12.5253388 13.7419652 -35.2846723
H -12.2047179 14.3950538 -34.4716908
C -11.9014053 14.2583629 -36.5896139
H -12.1485647 13.587198 -37.4129321
H -10.8182307 14.3273054 -36.4922998
H -12.2846337 15.2548982 -36.8119629
C -14.0473735 13.8569901 -35.4397281
H -14.390893 13.2481522 -36.2760117
H -14.3288191 14.8958381 -35.6094773
H -14.5551193 13.5168148 -34.5364626
N -9.8327613 12.5183483 -34.0376294
H -10.2691124 13.0158676 -33.2677802
C -8.3866883 12.2947826 -33.9964652
H -7.94803 12.713808 -34.9032965
C -8.0253687 10.7951613 -33.964585
O -7.1750255 10.3535353 -34.7385592
C -7.808095 13.0498299 -32.7940855
H -8.2322251 12.6640796 -31.8665257
H -8.0425363 14.1119177 -32.8768195
H -6.7249933 12.9258191 -32.7693519
N -8.7383063 9.9928143 -33.1690346
H -9.4399586 10.4216383 -32.5757625
C -8.5261026 8.5373236 -33.0519002
H -7.476906 8.3564513 -32.8190178
C -8.836892 7.7959867 -34.3557814
O -8.0721417 6.9214099 -34.7582541
C -9.3671494 7.960609 -31.9009351
H -10.4270834 8.1175921 -32.1029293
C -9.1210937 6.4714297 -31.6602582

H -8.0569718 6.2924769 -31.5037895
H -9.4663804 5.8883818 -32.5134126
H -9.6722286 6.1524028 -30.7752524
O -9.0287859 8.6085484 -30.6970051
H -9.4300372 9.4946216 -30.711502
N -9.8765834 8.2071453 -35.0894348
H -10.4751493 8.9342993 -34.7066042
C -10.218649 7.6484065 -36.4087586
H -10.3078933 6.565189 -36.3149444
C -9.0961985 7.9031619 -37.4291875
O -8.8107229 7.0319327 -38.2457573
C -11.5883493 8.2058925 -36.8724934
H -11.5800876 9.2895272 -36.7418409
C -11.8649231 7.9080697 -38.3605028
H -11.8182208 6.8336763 -38.5433383
H -11.1330206 8.4106419 -38.9928817
H -12.8465962 8.2794081 -38.6492608
C -12.7194695 7.6055139 -36.002287
H -12.8725628 6.5606242 -36.275951
H -12.4291906 7.6247014 -34.9527788
C -14.0547499 8.3529914 -36.1158443
H -14.469557 8.2558855 -37.1180749
H -13.9111854 9.4081356 -35.8800284
H -14.765007 7.9268607 -35.4065953
N -8.3937191 9.0388911 -37.3367375
H -8.6846259 9.7176446 -36.6427142
C -7.265239 9.3812153 -38.2229573
H -7.5533014 9.1179589 -39.2408252
C -5.9497281 8.6286735 -37.9283472
O -4.9741865 8.7465544 -38.6840646
C -7.0390962 10.899473 -38.1899562
H -8.0014138 11.4136505 -38.2093944
H -6.4800146 11.1924024 -39.0771632
O -6.2990547 11.3175345 -37.05805
H -6.6658562 10.914714 -36.2473093
N -5.8900761 7.8921141 -36.8100548
H -6.7249579 7.8243468 -36.2397125
C -4.6565738 7.3302429 -36.256341
H -3.8362244 8.0007177 -36.5123651
C -4.2800623 5.9408626 -36.8074929
O -3.1352983 5.5205919 -36.6491747
C -4.7709993 7.3240925 -34.7269169
H -5.5236596 6.6026775 -34.4107831
H -5.0474193 8.3167635 -34.3677705
H -3.8122191 7.0434425 -34.2890576
N -5.1800992 5.2659515 -37.5310566

H -6.0659794 5.7270073 -37.7010793
C -4.9863126 3.9392569 -38.1512939
H -5.0038412 3.1815968 -37.3671395
C -3.6467069 3.7571432 -38.8886477
O -2.9731921 2.7386014 -38.7264792
C -6.1697208 3.6740353 -39.1048049
H -7.0402153 3.4328757 -38.4935022
H -5.9499646 2.7970377 -39.7150907
C -6.5637775 4.8197352 -40.0201111
N -5.7699764 5.9207787 -40.3641479
C -6.5674691 6.752385 -41.049963
H -6.277414 7.7318812 -41.4028678
N -7.7926363 6.2244398 -41.1826337
H -8.5854946 6.6964794 -41.5940588
C -7.8129371 5.0107498 -40.5332027
H -8.6720512 4.3747877 -40.3736965
N -3.1845526 4.7824287 -39.6094995
H -3.8397485 5.5490973 -39.7402001
C -1.9094941 4.7790629 -40.3503428
H -1.9058746 3.9210138 -41.0259016
C -0.6755178 4.6000095 -39.4590681
O 0.3500762 4.1523355 -39.9634635
C -1.8108003 6.0655976 -41.1818839
H -1.9802055 6.9149351 -40.5182176
H -0.8101594 6.155225 -41.6088882
C -2.8366963 6.0737128 -42.3295833
H -3.7805566 5.6467642 -41.9998432
H -2.4675716 5.4691415 -43.158521
C -3.090666 7.5062857 -42.7975055
H -2.21557 7.8834898 -43.328123
H -3.2604295 8.1357064 -41.9227019
N -4.2898586 7.6146451 -43.6353146
H -5.0985667 8.0752973 -43.2163844
C -4.3632322 7.5927761 -44.9465972
N -3.4118636 7.0991039 -45.680707
H -3.2172373 7.5904414 -46.5573116
H -2.6560279 6.644648 -45.2205878
N -5.4028833 8.1065841 -45.5234574
H -6.0523492 8.6180683 -44.9202338
H -5.4423454 8.1830156 -46.5185052
N -0.7546542 4.8641941 -38.1528525
H -1.6478001 5.1514024 -37.7665882
C 0.3441051 4.6164829 -37.2169392
H 1.2323201 5.1264964 -37.5885598
C 0.6996833 3.1210416 -37.0906775
O 1.8637562 2.7984179 -36.8587498

C -0.0133202 5.2297426 -35.858726
H -0.9017486 4.748926 -35.4486878
H -0.2065543 6.2971976 -35.9704315
H 0.8169899 5.0909097 -35.1647239
N -0.2423564 2.2117153 -37.3765593
H -1.1831707 2.5200405 -37.5942453
C 0.0441412 0.7786215 -37.4595675
H 0.5673747 0.4724402 -36.5515695
C 0.9552045 0.4341165 -38.6550826
O 1.7906982 -0.4590169 -38.5465271
C -1.2872208 0.0221727 -37.522223
H -1.8167315 0.2672544 -38.4447155
H -1.9079066 0.2944651 -36.668599
H -1.1024213 -1.0504495 -37.4916568
N 0.8781965 1.1977947 -39.7544587
H 0.2102942 1.9555281 -39.7611379
C 1.8112123 1.0873253 -40.8905783
H 2.0039892 0.0301488 -41.0804667
C 3.1532747 1.7347408 -40.5479506
O 4.1918602 1.1211358 -40.7786454
C 1.2242149 1.7004207 -42.1796402
H 1.0712918 2.7691655 -42.0362597
C 2.1680245 1.5163423 -43.3761676
H 2.3959256 0.458749 -43.5151264
H 3.0968456 2.0616945 -43.2111974
H 1.7047064 1.9050683 -44.2829471
C -0.1270984 1.0684292 -42.5415657
H -0.0134202 -0.0094525 -42.6618157
H -0.4995357 1.4948188 -43.4727939
H -0.8607535 1.2658962 -41.7604837
N 3.1497115 2.9075864 -39.8983128
H 2.2584563 3.3646896 -39.7477886
C 4.3829764 3.5769137 -39.4271644
H 5.0316617 3.7532182 -40.287047
C 5.1714679 2.6870662 -38.4572727
O 6.3914124 2.6466122 -38.5342007
C 4.0854947 4.9447377 -38.7734971
H 3.4492565 4.7868126 -37.9038288
C 5.3579813 5.667574 -38.3077099
H 6.0473034 5.7700222 -39.1479344
H 5.8520541 5.0988091 -37.5198768
H 5.1091859 6.6515776 -37.9116408
C 3.3657123 5.9094878 -39.7292705
H 4.0142422 6.146524 -40.5715909
H 3.1117469 6.8290265 -39.2030367
H 2.4524687 5.4645623 -40.1099666

N 4.5099699 1.8691594 -37.6261136
H 3.501018 1.9661057 -37.5679173
C 5.1768327 0.8861925 -36.7517641
H 5.9189905 1.4157616 -36.1524491
C 5.9655105 -0.1805465 -37.5278367
O 6.9655235 -0.6580613 -37.005066
C 4.1151826 0.2654933 -35.821984
H 3.6872668 1.0584642 -35.2070059
H 3.3115988 -0.1409395 -36.4366679
C 4.6026419 -0.8663175 -34.8987054
H 4.9701494 -1.6946528 -35.5041773
H 3.7425868 -1.2253198 -34.3369632
C 5.691348 -0.4487287 -33.8979481
H 5.305648 0.3329929 -33.2411631
H 6.5347315 -0.0299299 -34.4515579
N 6.1852786 -1.5988657 -33.1065998
H 7.0302672 -2.023504 -33.4565587
C 5.6931212 -2.0929878 -31.9796119
N 4.6137617 -1.6238221 -31.4121932
H 4.2496792 -2.0315972 -30.5644291
H 4.1500759 -0.8388203 -31.8268032
N 6.2826626 -3.0960025 -31.392016
H 7.158683 -3.4460252 -31.7437805
H 5.9220268 -3.4384356 -30.5210906
N 5.5130667 -0.5786026 -38.7196586
H 4.6989518 -0.1098716 -39.0946508
C 6.155844 -1.6215128 -39.5477068
H 6.7171868 -2.2989085 -38.9044692
C 7.1870424 -1.0471198 -40.5141252
O 8.1672071 -1.7115576 -40.8379074
C 5.0729423 -2.429844 -40.2970744
H 4.4719049 -1.7392024 -40.8919679
C 5.6505611 -3.4778044 -41.2668926
H 6.3652394 -4.1194898 -40.7503349
H 6.1577817 -2.9777053 -42.0922651
H 4.8537515 -4.0898952 -41.6888069
C 4.1272498 -3.1381766 -39.3001663
H 3.4521743 -2.3981917 -38.8745679
H 3.519856 -3.8567523 -39.8472324
C 4.8022508 -3.8790266 -38.1377464
H 5.1620025 -3.1674568 -37.3956499
H 5.6444629 -4.4611102 -38.5038849
H 4.0797422 -4.5405924 -37.6627072
N 6.9634562 0.1827065 -40.9660073
H 6.1059518 0.645179 -40.6903566
C 7.8918005 0.9019409 -41.8307362

H 8.3560989 0.1968688 -42.5199967
C 9.0541694 1.5083209 -41.0392823
O 10.1751688 1.4175308 -41.5113396
C 7.0910835 1.9372281 -42.6425295
H 6.4755174 2.5141194 -41.9494156
C 8.015055 2.9405889 -43.3353853
H 8.8170607 2.4130698 -43.8517664
H 8.4436482 3.6079035 -42.5859543
H 7.4537262 3.5413332 -44.0376991
C 6.1329575 1.2547909 -43.6511673
H 5.3388 0.7519175 -43.1009127
H 5.6559528 2.0240523 -44.2518133
C 6.7607953 0.2206656 -44.5975052
H 7.0017951 -0.6915304 -44.0477814
H 7.6692296 0.6123557 -45.0487245
H 6.0481605 -0.0291608 -45.3837918
N 8.798992 1.9819869 -39.8195457
H 7.8269675 1.9509433 -39.5448266
C 9.6149082 2.8267209 -38.9312898
H 9.2170332 2.5797365 -37.9459199
C 9.3250434 4.3409519 -39.0306445
O 8.5238253 4.7993794 -39.8542185
C 11.099113 2.4020543 -38.8279642
H 11.7082873 3.1408157 -39.3434734
H 11.2510327 1.4269978 -39.2904088
C 11.5965131 2.3038259 -37.3833269
O 11.4492724 3.3205405 -36.6703128
O 12.1249463 1.2330349 -37.0094698
N 9.8588958 5.1058401 -38.0754761
H 10.516748 4.6466534 -37.4396315
C 9.3325992 6.4041998 -37.6512809
H 8.3526531 6.2042132 -37.2199303
C 9.1395831 7.4234129 -38.7896545
O 10.0494829 7.7492656 -39.5515223
C 10.2024572 6.9760756 -36.5200309
H 10.3373002 6.2059069 -35.7593743
H 11.1814432 7.2466722 -36.9189787
C 9.5601829 8.2096529 -35.8657287
H 8.5933201 7.9247972 -35.443284
H 9.3792816 8.9717041 -36.6261681
C 10.4478246 8.8008571 -34.7647909
O 10.8020372 8.0456266 -33.8314376
O 10.7428137 10.012847 -34.8410345
N 7.9356494 8.0039627 -38.8202964
H 7.2747294 7.6949909 -38.1276181
C 7.4979327 9.066309 -39.7246913

H 6.4475177 9.2219085 -39.4784392
C 7.4607978 8.7250306 -41.2328113
O 7.2182063 9.6195186 -42.0501456
C 8.189382 10.3821855 -39.339387
H 9.2533861 10.3291555 -39.5703739
H 8.0772996 10.5585665 -38.2684137
H 7.7299772 11.2135753 -39.8729389
N 7.5456185 7.4423073 -41.6155564
H 7.7541 6.7354562 -40.9135248
C 7.344726 6.9801426 -42.997353
H 6.9802142 7.8289962 -43.5714683
C 6.2228111 5.9398072 -43.1423331
O 5.8136015 5.298277 -42.1811172
C 8.7040589 6.5417867 -43.5937623
H 9.3769214 6.2789443 -42.781539
H 8.5857406 5.6690598 -44.2357264
C 9.2911549 7.7100855 -44.425368
H 9.1907816 8.6410518 -43.8675988
C 10.7676023 7.5379856 -44.7641775
H 10.9094523 6.6899523 -45.4326336
H 11.347273 7.4053009 -43.8535032
H 11.1408564 8.4293545 -45.2677901
C 8.5546316 7.8484516 -45.7653518
H 8.6226347 6.9085588 -46.3141622
H 9.0244539 8.6267692 -46.3653165
H 7.5122898 8.121164 -45.6183874
N 5.6747873 5.8134553 -44.3622881
H 6.051826 6.3868802 -45.1032308
C 4.5891328 4.8649351 -44.6980872
H 4.5631062 4.1139725 -43.9051263
C 4.8447935 4.0459821 -45.973334
O 3.9017478 3.6498304 -46.6520897
C 3.2105171 5.5603428 -44.666108
H 2.4533252 4.7738203 -44.6831793
H 3.1054279 6.0864467 -43.7153432
C 2.9161759 6.5454592 -45.8179066
H 3.1261552 6.0697121 -46.7735315
H 3.5448542 7.4315362 -45.7241825
C 1.4308664 6.9484155 -45.7776237
H 1.2590212 7.5693356 -44.8966521
H 0.8212512 6.0482975 -45.6740764
N 0.981203 7.6811794 -46.9780385
H 0.7524881 8.6673509 -46.8923419
C 0.6228584 7.177358 -48.1443321
N 0.8186153 5.929421 -48.4685675
H 0.5752193 5.6132175 -49.3870961

H 1.388143 5.3577715 -47.8677134
N 0.0406873 7.949872 -49.0092621
H -0.2933387 8.855375 -48.6610897
H -0.2623347 7.6163466 -49.9005672
N 6.1190758 3.8578277 -46.3209588
H 6.8301691 4.1363819 -45.6635574
C 6.5958218 3.2428633 -47.5626859
H 5.830005 2.5719029 -47.9542416
C 7.8916732 2.4367347 -47.343168
O 8.5668267 2.64766 -46.3401455
C 6.8674228 4.3491416 -48.5851797
H 7.7970464 4.8464069 -48.304794
H 7.032437 3.8770175 -49.5533758
C 5.793543 5.4093771 -48.7466088
C 4.5694425 5.071977 -49.3430479
H 4.3617759 4.0434651 -49.5976387
C 3.6563468 6.0786253 -49.6922932
H 2.7837729 5.8310304 -50.2772817
C 3.9157208 7.4151039 -49.3467661
H 3.2349086 8.196869 -49.6514519
C 5.0976506 7.7468283 -48.6644446
H 5.3156042 8.7854837 -48.4512446
C 6.0470079 6.7471097 -48.3865945
H 6.9997683 7.0245668 -47.966154
N 8.2414517 1.5676536 -48.3001291
H 7.6597218 1.5224626 -49.118932
C 9.3979198 0.6578697 -48.2914649
H 9.1016447 -0.2646165 -47.791358
C 10.6602547 1.1969945 -47.5742744
O 11.1786285 2.245083 -47.9427162
C 9.7087369 0.3224341 -49.75895
H 9.967396 1.234773 -50.3010873
H 8.8483582 -0.1504811 -50.2328802
H 10.5608699 -0.3531544 -49.8204047
N 11.1058984 0.4683517 -46.5404187
H 10.6020477 -0.3756197 -46.3181145
C 12.3498292 0.6411544 -45.7620579
H 12.0842348 0.2321049 -44.78769
C 12.7677959 2.0582833 -45.3295639
O 13.9257997 2.2966503 -44.965395
C 13.488643 -0.2542563 -46.2966031
H 14.4106292 0.3125754 -46.3885813
H 13.233996 -0.5667301 -47.3062372
C 13.7404825 -1.5012609 -45.4212878
O 12.782663 -1.9446545 -44.7479203
O 14.8558588 -2.0616043 -45.4835954

N 11.7676098 2.9221305 -45.1175289
H 10.8663841 2.6705479 -45.5032949
C 11.8434355 4.1137479 -44.2565654
H 10.9702733 4.7131008 -44.4953178
C 13.0474841 5.0013958 -44.5690478
O 13.2566867 5.4146228 -45.7033659
C 11.6839069 3.7215239 -42.7654516
H 10.7042933 3.2772 -42.7433744
C 12.7172875 2.6995277 -42.243958
H 13.7411237 3.021697 -42.4103113
H 12.5627012 1.7344082 -42.7242798
H 12.5786212 2.5252549 -41.1832717
C 11.6078686 4.8907969 -41.7666777
H 12.5088229 4.963613 -41.1569353
H 10.7748965 4.7152793 -41.0967139
H 11.4403098 5.837781 -42.2673589
N 13.7997814 5.3551081 -43.5357305
H 13.5427582 4.9757027 -42.6392564
C 14.9722896 6.1999847 -43.5773004
H 14.7513394 7.1316738 -44.1038787
C 16.1325397 5.5277022 -44.3124657
O 16.971215 6.2121722 -44.8791594
C 15.3363167 6.5059358 -42.1167684
H 14.5993989 7.1982355 -41.7073855
H 15.2826306 5.5845827 -41.5328418
C 16.7149966 7.0988042 -41.9408267
C 16.9780411 8.3984896 -42.403626
H 16.1908624 8.9809401 -42.8654804
C 17.7536204 6.3218351 -41.3924162
H 17.5600729 5.3123153 -41.0611771
C 18.2759221 8.9240865 -42.299878
H 18.4802673 9.9218835 -42.6403416
C 19.0546992 6.8452967 -41.3078177
H 19.8555147 6.2433117 -40.90308
C 19.314826 8.149128 -41.7601691
H 20.3159865 8.5514257 -41.6963891
N 16.2058513 4.196207 -44.3268838
H 15.3924863 3.6449569 -44.0809636
C 17.3365519 3.5317345 -44.9880782
H 18.247824 4.0148928 -44.6328091
C 17.3362498 3.7162578 -46.5202865
O 18.411362 3.6889561 -47.1212283
C 17.4139431 2.0551039 -44.544089
H 17.2640812 2.0186564 -43.4640312
C 18.7685605 1.4097662 -44.8475106
H 18.7772956 0.3945679 -44.4540022

H 19.5699411 1.9849547 -44.3903993
H 18.9200973 1.3641078 -45.9264033
O 16.4573901 1.2286298 -45.1599023
H 15.5577683 1.5969757 -45.0914068
N 16.1818698 4.0690221 -47.1078517
H 15.3465728 4.1192239 -46.5422669
C 16.0387294 4.3659216 -48.539581
H 16.9320402 3.9392428 -48.9945931
C 16.0907108 5.8966401 -49.0803814
O 16.0794458 5.9767522 -50.2908172
C 14.8556147 3.4957892 -49.0773819
H 13.9798023 4.1366509 -49.1933825
H 14.5968292 2.7346661 -48.3378472
C 15.1275989 2.7525254 -50.4024108
O 14.1400315 2.2286328 -50.965854
O 16.307982 2.6299838 -50.8096799
N 16.2900343 7.1238414 -48.4203981
H 16.4497442 7.0131143 -47.4277553
C 16.1613041 8.688626 -48.8951202
H 15.5643498 8.6270493 -49.8051702
C 17.4543756 9.8556596 -49.3389581
O 18.4787587 9.2741032 -49.0634247
C 15.1839224 9.2297703 -47.7945255
H 14.6070166 8.4267525 -47.3302822
H 14.4664933 9.8869038 -48.2885934
C 15.9020718 10.0680588 -46.713811
O 16.3872989 11.1886415 -47.009515
O 15.9809798 9.6471257 -45.537687
N 17.6582507 11.2710311 -50.0187799
H 16.7432841 11.6473397 -50.215925
C 18.9196021 12.4999217 -50.4996121
H 19.4239745 12.7072473 -49.557024
C 19.8910586 11.6284874 -51.3214552
H 19.3040143 10.926469 -51.916006
H 20.4598406 12.2416738 -52.0211191
C 20.8996192 10.8577388 -50.4511839
H 20.4908512 10.6758339 -49.4595413
H 21.799341 11.4600084 -50.3269145
C 21.2793501 9.5187183 -51.0747255
O 22.2065281 9.4215954 -51.8603748
N 20.5592735 8.459898 -50.7803827
H 20.794535 7.5910496 -51.2218482
H 19.7724068 8.5434667 -50.1355936
C 19.092366 14.182846 -51.3913657
O 18.0971829 14.350783 -52.0856275
N 20.1085432 15.3856543 -51.5493382

H 20.9260687 15.212857 -50.986138
C 20.2948739 16.9461427 -52.3131094
H 20.2227503 16.7196393 -53.3770916
C 21.4078328 18.3179152 -52.2406162
O 22.5468109 18.183726 -51.7988277
C 19.0686253 17.7464106 -51.9697146
H 19.2338989 18.2728335 -51.0391949
H 18.2005998 17.0932304 -51.9204808
H 18.8667864 18.4802587 -52.7438545
N 21.1702238 19.6749261 -52.6514902
H 20.2345851 19.8379585 -52.9935248
C 22.0024176 21.0115075 -52.4479166
H 22.8683875 20.7431257 -51.8441611
C 21.2832516 22.1579803 -51.6588977
O 20.098186 22.3880363 -51.8856444
C 22.5113401 21.7661131 -53.7349204
H 21.6390627 22.0773934 -54.3111731
H 23.0283835 22.6774737 -53.4283394
C 23.4507222 21.0203275 -54.6889031
H 22.9115143 20.1707727 -55.1106251
H 23.7236569 21.6915994 -55.5045992
C 24.7266258 20.5101083 -54.0066815
H 24.4530278 19.8220364 -53.2042071
H 25.2791937 21.3544152 -53.5879837
C 25.5984771 19.7681775 -55.0268131
H 25.909443 20.4728439 -55.8051529
H 24.9888066 18.9920389 -55.5006992
N 26.7854235 19.1515691 -54.3842775
H 27.3653918 19.8580064 -53.9512649
H 27.3408378 18.6543916 -55.0695705
H 26.4940335 18.4923601 -53.6723818
N 22.0141776 22.9790716 -50.8776924
H 22.9848334 22.7561847 -50.7481297
C 21.4363151 24.0595014 -50.0406279
H 20.6651534 23.5880877 -49.4217957
C 20.6932301 25.1571828 -50.8434591
O 21.1123859 25.4928957 -51.9501055
C 22.4954675 24.6021902 -49.0576117
H 21.9681595 25.0697176 -48.2281703
H 23.0446883 23.7611733 -48.6298199
C 23.4967813 25.6136439 -49.6395768
H 24.1308012 25.1185841 -50.3761876
H 22.9616084 26.426131 -50.1294037
C 24.3600993 26.2058559 -48.5122502
H 25.0695733 25.4548231 -48.159759
H 23.7129745 26.4715583 -47.6719088

C 25.1007745 27.47991 -48.9414417
H 25.6487684 27.8643149 -48.0751497
H 24.3538345 28.231996 -49.2207631
N 26.033051 27.2429212 -50.0717632
H 26.729265 26.5557402 -49.8134378
H 26.5058074 28.1019389 -50.3270792
H 25.5216444 26.9054137 -50.8778729
N 19.6653497 25.7902966 -50.2502701
C 18.5260935 26.4199977 -50.9302971
H 17.683172 26.2611953 -50.25573
C 17.9666354 25.9230463 -52.2825018
O 17.2120042 26.6858309 -52.8855268
C 18.8245867 27.9163968 -50.8994079
H 19.6064095 28.1600803 -51.6210341
H 17.9311673 28.5198536 -51.0658878
C 19.9982801 26.7186724 -49.1631419
H 19.6497361 26.3469128 -48.204326
H 21.0706298 26.8855675 -49.103357
C 19.3411503 28.0678737 -49.4726293
H 18.5032263 28.2293829 -48.7937525
H 20.0551531 28.8885143 -49.395854
N 18.1444302 24.6585658 -52.7170806
H 18.7575415 24.0313991 -52.2081714
C 17.2497154 24.0944877 -53.7603078
H 17.1809009 24.8305569 -54.5644565
C 15.8108259 23.9536811 -53.2184155
O 15.6116559 23.7733122 -52.0168994
C 17.7905208 22.7718561 -54.367871
H 18.2164828 22.1394345 -53.5915213
H 16.9504542 22.2274091 -54.799801
C 18.8275157 22.9966047 -55.490398
H 19.7490875 23.3914824 -55.0618205
H 18.4292468 23.7618966 -56.1540239
C 19.1471149 21.7322105 -56.331149
H 18.3107319 21.0347568 -56.2604309
H 20.0375569 21.2460875 -55.9308658
N 19.3493069 22.0762304 -57.7577649
H 19.2235844 23.0639289 -58.0004847
C 19.3902273 21.3013719 -58.8329725
N 19.5688706 20.0105413 -58.789513
H 19.5868354 19.4947518 -59.6476506
H 19.6328429 19.5190873 -57.9186205
N 19.2411043 21.8219077 -60.01574
H 18.7989704 22.7503794 -60.07748
H 19.2658129 21.2691169 -60.8598066
N 14.821947 23.9635607 -54.1171727

H 15.0640462 24.1295175 -55.0795782
C 13.4070457 23.7053603 -53.8023649
H 13.2318405 23.8314889 -52.7303655
C 13.0148305 22.2690653 -54.1901151
O 13.2650034 21.8554559 -55.3268438
C 12.5042714 24.70102 -54.554355
H 11.4657565 24.4018961 -54.4176821
H 12.7363243 24.6603459 -55.6166903
C 12.5918951 26.14774 -54.1111584
O 13.1869008 26.5106661 -53.1113018
N 11.9857542 27.0281791 -54.8750885
H 12.0663734 27.990201 -54.6088372
H 11.474667 26.7304553 -55.7012412
N 12.3104354 21.5618859 -53.3039725
H 12.1319232 21.9882772 -52.3959269
C 11.7798987 20.2088839 -53.5218785
H 12.1354009 19.8236533 -54.4750295
C 10.2499869 20.2006078 -53.5304971
O 9.636071 20.6928764 -52.5837385
C 12.2939867 19.2814334 -52.4104324
H 12.0003572 19.7111831 -51.4550013
H 13.3811827 19.2399207 -52.459261
C 11.7331357 17.8531895 -52.451972
H 10.6473428 17.8812916 -52.5280063
H 11.9562163 17.3702826 -51.5054196
S 12.3827862 16.813984 -53.7779941
C 13.8931229 16.2105975 -52.9728887
H 14.4627188 15.602152 -53.6752431
H 13.6285852 15.6018071 -52.1075691
H 14.5036321 17.0516099 -52.6454799
N 9.6561252 19.476022 -54.4810838
H 10.2356146 19.1014098 -55.228018
C 8.2618632 19.0294284 -54.4110718
H 7.7432147 19.5950109 -53.6411374
C 8.2012683 17.5367186 -54.0391612
O 8.6091906 16.6916482 -54.8315145
C 7.5848191 19.3377223 -55.7563497
H 7.6533027 20.404727 -55.9472126
H 8.1407942 18.8235216 -56.5354205
C 6.1094319 18.9161937 -55.8471269
H 6.0223168 17.8621598 -55.5987058
C 5.2008171 19.7105303 -54.9111302
H 5.2842959 20.7773402 -55.1160026
H 5.4789999 19.5191252 -53.8783082
H 4.1706631 19.3882301 -55.0433692
C 5.5958432 19.1157678 -57.2735893

H 5.6369121 20.1715894 -57.5396105
H 4.5696247 18.7588489 -57.3505647
H 6.2159663 18.5490014 -57.9673902
N 7.650806 17.207511 -52.8705797
H 7.2809473 17.9542867 -52.2913677
C 7.3710433 15.8279812 -52.4464654
H 8.0060151 15.1508264 -53.0126345
C 5.9027388 15.4793458 -52.7611271
O 4.9709185 16.075372 -52.2065813
C 7.75986 15.6405981 -50.9667627
H 7.1203425 16.2573012 -50.3370099
H 8.7862314 15.9905422 -50.8370111
C 7.6824443 14.1863395 -50.4760692
O 6.8708591 13.4090086 -51.019936
O 8.4087115 13.8653462 -49.5055914
N 5.6864177 14.5711102 -53.7189983
H 6.4974395 14.0713565 -54.0777662
C 4.3447916 14.1842873 -54.1843736
H 3.6345714 14.9349283 -53.8406842
C 3.9351687 12.8544228 -53.5605881
O 4.3232699 11.7831128 -54.0192536
C 4.2336867 14.1575708 -55.7201635
H 4.8568936 13.3737071 -56.1278179
C 2.7974381 13.8629456 -56.1479142
H 2.1426664 14.6095088 -55.703318
H 2.4969621 12.8683791 -55.8186253
H 2.7229068 13.8922377 -57.2343704
C 4.6461988 15.4961607 -56.3443168
H 4.0765308 16.3124174 -55.897042
H 4.4772019 15.482627 -57.4198195
H 5.7094427 15.6583488 -56.1699027
N 3.0257182 12.9205146 -52.5909151
H 2.717312 13.8404901 -52.2952782
C 2.6511322 11.7941466 -51.7378099
H 1.5919382 11.8736081 -51.495824
H 2.826334 10.8515972 -52.2505211
C 3.4378837 11.764253 -50.4292475
O 3.7783718 10.6859284 -49.9559292
N 3.6725557 12.9262538 -49.8125931
H 3.3218807 13.7634739 -50.2589932
C 4.5639542 13.1282033 -48.6574106
H 5.5763021 12.8435635 -48.9560907
C 4.2394702 12.317265 -47.379758
O 4.9944892 12.3378076 -46.4012804
C 4.557135 14.6320349 -48.3562011
H 4.9178299 15.1702423 -49.2356233

H 5.2327209 14.8285762 -47.5267004
S 2.8894722 15.2074225 -47.9074698
H 2.7061254 14.4032963 -46.8479299
N 3.0995178 11.6227276 -47.3420058
H 2.5537956 11.6005599 -48.1895828
C 2.6973692 10.7695608 -46.2284903
H 1.7296098 10.3306215 -46.4382888
H 3.4252333 9.964224 -46.1301078
C 2.5964225 11.5335775 -44.9096616
O 2.0756425 12.6458024 -44.8617237
N 3.1475683 10.9405853 -43.8409104
H 3.5995258 10.050913 -43.9813798
C 3.2644505 11.5676352 -42.5110625
H 2.5363332 12.3721322 -42.4922009
C 4.6024855 12.3215117 -42.2989558
O 4.9330438 12.7035656 -41.1837733
C 2.7685976 10.6219858 -41.3876643
H 3.329892 9.6866489 -41.3966698
H 2.9110664 11.1133101 -40.4234859
C 1.2482459 10.3497499 -41.5881163
H 0.7919812 11.3134804 -41.7977461
H 1.1127473 9.7337692 -42.4784474
C 0.4227669 9.7161131 -40.4391496
H 0.7314226 10.1512193 -39.4901884
H 0.5960085 8.639396 -40.4025918
C -1.0758004 10.0217816 -40.7095582
H -1.41602 9.4220592 -41.5561837
H -1.1388727 11.0716698 -41.0127608
N -2.0107233 9.8625097 -39.5598994
H -2.0996365 8.9127489 -39.2334567
H -2.9406933 10.1949484 -39.8135329
H -1.7309402 10.4529809 -38.7839952
N 5.3030928 12.6456133 -43.3932679
H 4.9509657 12.3158047 -44.2839729
C 6.263606 13.7539353 -43.4685514
H 6.339384 14.0373157 -44.5168193
H 5.8664254 14.6025745 -42.9108326
C 7.6966603 13.5217271 -42.9844836
O 8.5229441 14.4189115 -43.1446022
N 8.052298 12.3272621 -42.5032093
H 7.3432049 11.6119669 -42.3818921
C 9.4128024 12.0200965 -42.040962
H 9.6646061 12.6654394 -41.1981862
H 9.4474178 10.9834761 -41.7123813
C 10.481707 12.1834795 -43.1192423
O 11.5222019 12.7773601 -42.8556136

N 10.1750316 11.7843984 -44.3589289
H 9.2846575 11.3332788 -44.4955374
C 11.0186339 12.0413754 -45.5425943
H 11.9980506 11.588156 -45.3790969
C 11.2701527 13.5389522 -45.7486745
O 12.4236188 13.9518113 -45.8260238
C 10.4258214 11.3832015 -46.8083101
H 10.5905208 10.3099959 -46.7269139
C 8.9312833 11.6033541 -47.0463739
H 8.7099762 12.6632397 -47.1442538
H 8.3393666 11.1827496 -46.2344072
H 8.6361123 11.1131843 -47.9758533
O 11.0895781 11.8306891 -47.9597574
H 10.484578 12.3844576 -48.4701324
N 10.2252136 14.371186 -45.663101
H 9.3074535 13.9722939 -45.5446904
C 10.2857366 15.8236971 -45.8547703
H 10.7235008 16.0265557 -46.8268567
C 11.1106349 16.537419 -44.7650356
O 11.8863183 17.4526382 -45.0535196
C 8.8657689 16.3970441 -45.8856242
H 8.8986116 17.4030429 -46.3069571
H 8.4867539 16.4648006 -44.8668808
S 7.7163401 15.3759861 -46.8510702
H 8.4533831 15.1086649 -47.9512639
N 10.9632892 16.0999518 -43.508704
H 10.2825801 15.3646959 -43.3383485
C 11.7666912 16.5933009 -42.3777083
H 11.7210304 17.682167 -42.3558897
C 13.2337723 16.2149077 -42.5646649
O 14.1041332 17.0794444 -42.4631432
C 11.2181531 16.0596538 -41.0385742
H 11.0788588 14.9803609 -41.1088303
C 12.1583111 16.3454275 -39.8608403
H 12.413162 17.4044263 -39.8365978
H 13.0713173 15.7583224 -39.9544092
H 11.6747709 16.0658247 -38.9240883
C 9.8619149 16.7101419 -40.7311641
H 9.9738266 17.7906836 -40.6408162
H 9.465485 16.313601 -39.7953368
H 9.147258 16.4904463 -41.5236473
N 13.5086787 14.9554922 -42.9189931
H 12.7374487 14.300883 -43.0121126
C 14.8616324 14.4600437 -43.1763215
H 15.4605874 14.5795315 -42.2714439
C 15.5717593 15.257738 -44.2626702

O 16.6984701 15.6980688 -44.0520232
C 14.8119003 12.9696725 -43.5454865
H 15.47017 12.778969 -44.3885111
H 13.8159203 12.6800994 -43.8734669
C 15.2350372 12.1081566 -42.3887418
N 16.5503927 11.9810507 -41.9354251
C 16.4753185 11.2196844 -40.8299497
H 17.3253826 10.9192111 -40.2306378
N 15.2029599 10.8627996 -40.5836198
H 14.890693 10.2906702 -39.8085624
C 14.4037633 11.4233475 -41.5538219
H 13.3257702 11.3738953 -41.6274226
N 14.9078435 15.5075468 -45.387301
H 13.989292 15.091668 -45.5152898
C 15.5112271 16.2350314 -46.5011762
H 16.4944499 15.8005517 -46.6810838
C 15.788866 17.702243 -46.1455768
O 16.9395239 18.1374501 -46.2012692
C 14.6897345 16.0179734 -47.7882799
H 15.1327128 16.6236197 -48.5603665
C 14.7908711 14.5234165 -48.182398
H 14.1962195 13.8987777 -47.5198436
H 15.8220997 14.1781548 -48.1262874
H 14.4372692 14.3716629 -49.2011021
C 13.2366266 16.4975189 -47.7320752
H 12.7646062 16.0195718 -46.8976035
H 13.2059957 17.5711979 -47.5585925
C 12.4407379 16.1837724 -49.0033466
H 12.2244151 15.1164961 -49.062049
H 13.0189562 16.48687 -49.8741816
H 11.4970485 16.7230964 -48.9888032
N 14.8187234 18.4245126 -45.5793613
H 13.9008259 18.0120409 -45.4473034
C 15.0314858 19.8172963 -45.1857533
H 15.4248899 20.3646793 -46.0422485
C 16.0654803 19.9815299 -44.052507
O 16.775709 20.9872676 -44.0197683
C 13.6739593 20.4215669 -44.8288207
H 13.2487701 19.8884107 -43.9775321
H 12.9997992 20.3346726 -45.682005
H 13.7950387 21.4777533 -44.588073
N 16.1945128 19.0031707 -43.1417661
H 15.5550477 18.2125874 -43.1960906
C 17.1426889 19.0652184 -42.0165262
H 17.2682905 20.115006 -41.7540704
C 18.5555244 18.5806105 -42.3633946

O 19.5022617 19.2022987 -41.8920977
C 16.5356863 18.3408313 -40.8014493
H 15.5546198 18.7709715 -40.5929376
H 16.3868088 17.2899072 -41.0560764
C 17.3783728 18.4041283 -39.5137768
H 18.2761985 17.8007418 -39.655826
H 16.803368 17.9420041 -38.7110996
C 17.8159365 19.8120997 -39.0658109
H 18.4907804 20.2290409 -39.816017
H 18.3932875 19.7217441 -38.1440225
N 16.6897875 20.7491832 -38.8609668
H 16.5665419 21.4502419 -39.5716769
C 15.8807065 20.8193544 -37.8169131
N 14.9853566 21.7589121 -37.7435133
H 14.3645694 21.8061651 -36.956886
H 14.8726153 22.4170928 -38.4946404
N 15.9472023 19.9843426 -36.8189475
H 16.6241288 19.2455779 -36.8502505
H 15.2949912 20.0477172 -36.0589826
N 18.7300829 17.5290883 -43.1764286
H 17.9068943 17.0488226 -43.5306661
C 20.0684893 17.0390531 -43.5746573
H 20.7065707 16.9291063 -42.6964889
C 20.7865705 18.0002679 -44.5366372
O 22.0134416 18.0053269 -44.5586972
C 19.9813346 15.699411 -44.3219547
H 19.304658 15.8747004 -45.1616404
H 20.9671976 15.5209637 -44.7569981
C 19.5628823 14.3578569 -43.6784448
H 20.3290906 14.0366348 -42.971993
H 18.6129878 14.4437749 -43.1562454
C 19.4543242 13.3380914 -44.844005
H 20.418487 13.3271226 -45.354375
H 18.7167883 13.694725 -45.5669937
C 19.1362141 11.8730891 -44.4980398
H 19.490443 11.6480878 -43.4892399
H 19.686472 11.2395421 -45.2001729
N 17.6955314 11.5486037 -44.6343123
H 17.1272324 12.0169392 -43.9401613
H 17.4806865 10.5531329 -44.5600195
H 17.3202713 11.7308229 -45.5705452
N 20.0478271 18.6624719 -45.4364741
H 19.046923 18.5093921 -45.436498
C 20.6358853 19.3309775 -46.6085273
H 21.6749389 19.5607893 -46.3684505
C 20.0541746 20.7091617 -46.9622581

O 20.3152965 21.2210728 -48.0515675
C 20.6868561 18.354292 -47.7957786
H 21.0473326 18.879063 -48.6814314
H 21.4566581 17.6177888 -47.5613333
C 19.4373287 17.602644 -48.1667651
C 19.394892 16.1940631 -48.1271936
H 20.196105 15.6434021 -47.6560693
C 18.3451897 15.5113149 -48.7728262
H 18.2923324 14.4372359 -48.7484014
C 17.3896775 16.2497781 -49.5044677
O 16.5031357 15.6745726 -50.3589183
H 16.8175994 14.8393012 -50.723428
C 17.3695605 17.6457992 -49.4114359
H 16.6398896 18.1806343 -49.9827419
C 18.3848271 18.32481 -48.7295873
H 18.4231516 19.3990316 -48.7319403
N 19.3517895 21.3539171 -46.024161
H 19.1651048 20.8775033 -45.1539639
C 19.0384741 22.7911583 -46.0750765
H 18.3539854 22.9771147 -45.2457682
C 18.2660689 23.2374827 -47.3416898
O 18.3498463 24.3844502 -47.7803859
C 20.3254224 23.5929801 -45.7682459
H 21.0190659 23.4837652 -46.6030665
H 20.8007875 23.1682607 -44.8823023
C 20.0831759 25.0814136 -45.500918
O 19.0655738 25.4065578 -44.8420731
O 20.8655128 25.9146396 -46.0124582
N 17.4548563 22.3556176 -47.9295338
H 17.4238769 21.424189 -47.5465261
C 16.4823397 22.7281121 -48.9710723
H 16.9464183 23.4477338 -49.6473765
C 15.24281 23.3977265 -48.3725054
O 14.9522023 23.2457856 -47.1836795
C 16.0422905 21.5162724 -49.8130752
H 15.2603537 21.8499514 -50.4920367
C 17.2011516 21.0558416 -50.7125561
H 18.1143336 20.9147098 -50.1404907
H 17.4026366 21.8357731 -51.4439333
H 16.943333 20.1436236 -51.2459316
C 15.398919 20.4182088 -48.9405732
H 16.1665696 19.8238888 -48.4571155
H 14.7955406 20.8598812 -48.151009
C 14.4493492 19.5350595 -49.7478417
H 14.8862927 19.2531794 -50.7036183
H 13.5268763 20.0795328 -49.9194258

H 14.2201392 18.6408149 -49.1834476
N 14.4360211 24.010578 -49.2375354
H 14.7402083 24.0446147 -50.2085445
C 12.9993631 24.158699 -49.0076405
H 12.8175786 24.2573771 -47.9397338
C 12.2662528 22.9046082 -49.5103378
O 12.6107608 22.3634176 -50.5604784
C 12.5234879 25.4517901 -49.6859483
H 13.0406288 26.283309 -49.2048236
H 12.830272 25.44303 -50.731898
C 11.0112581 25.7338392 -49.6079951
H 10.8575365 26.7986213 -49.7828324
H 10.658697 25.5126149 -48.6002629
C 10.1424015 24.9839405 -50.6247752
O 10.5934761 24.3664138 -51.5757024
N 8.8434225 24.9954737 -50.4307784
H 8.2650384 24.4828781 -51.0906918
H 8.4331033 25.5005896 -49.6701504
N 11.2359635 22.4621827 -48.7844512
H 11.0578115 22.9049529 -47.8891113
C 10.3429926 21.383246 -49.214284
H 10.5949631 21.0920705 -50.235015
C 8.8711568 21.8264889 -49.2272665
O 8.3637704 22.3460571 -48.2286926
C 10.5732244 20.1618264 -48.309774
H 10.3332883 20.4190762 -47.2777976
H 11.6227969 19.8672222 -48.3609254
S 9.5400098 18.7650291 -48.8429061
H 8.3402798 19.3259352 -48.6493615
N 8.1565084 21.4877271 -50.299685
H 8.6580875 21.1050509 -51.0997332
C 6.6915622 21.4723115 -50.3796701
H 6.2622286 21.8728319 -49.4665579
C 6.2201094 20.031677 -50.5303368
O 6.4399128 19.4158451 -51.5664035
C 6.1943466 22.3420754 -51.5434893
H 6.7074003 22.060723 -52.4640156
C 4.6826819 22.2560971 -51.7615391
H 4.1511206 22.4652032 -50.8329504
H 4.408085 21.2625488 -52.117419
H 4.3832472 22.9787312 -52.5207642
O 6.4597851 23.693421 -51.2481149
H 6.0035097 23.908674 -50.423727
N 5.5750189 19.4858299 -49.5003679
H 5.4489048 20.0421054 -48.6609263
C 4.8934822 18.1915638 -49.5707935

H 5.3805521 17.5583986 -50.3075649
H 4.9561912 17.6920084 -48.6044006
C 3.4178491 18.3381219 -49.9451647
O 2.7499778 19.2457229 -49.4508098
N 2.8759735 17.4192718 -50.7447628
H 3.489359 16.728848 -51.172119
C 1.4218424 17.2768095 -50.9304672
H 0.9352328 17.8212694 -50.1261785
C 0.9560668 15.8305307 -50.7697592
O 1.6406314 14.8966804 -51.1867693
C 0.9136146 17.8905378 -52.2565359
H -0.1448096 17.6360649 -52.3435319
C 1.6387051 17.3248981 -53.5004349
H 2.6843131 17.6363338 -53.4916327
H 1.6106378 16.2363657 -53.4623999
C 0.9961788 19.4249524 -52.1858548
H 2.0365676 19.7521869 -52.1926341
H 0.5247676 19.7783184 -51.266841
H 0.4642133 19.8755679 -53.0218273
C 1.0052803 17.7519525 -54.8316522
H 1.1088672 18.8260369 -54.9781292
H -0.0515642 17.481976 -54.8436029
H 1.510511 17.2397663 -55.65089
N -0.2477491 15.6495421 -50.2260329
H -0.7465903 16.4668413 -49.8863459
C -0.9656272 14.3704334 -50.212057
H -0.6470092 13.784003 -51.0743246
C -2.4736961 14.6046495 -50.3361702
O -2.9923013 15.6108318 -49.855645
C -0.6345655 13.5626088 -48.9469876
H -1.035592 14.063649 -48.0667914
H 0.4483293 13.4718768 -48.8486547
O -1.2039403 12.2647033 -49.0661658
H -0.8230701 11.6633302 -48.3915009
N -3.189996 13.6624306 -50.9545782
H -2.693636 12.8593069 -51.3085801
C -4.6570956 13.7038033 -51.1109514
H -4.9453094 14.6681594 -51.5324737
C -5.3659256 13.5807981 -49.7505522
O -6.4750307 14.0756237 -49.5707605
C -5.0838402 12.5810381 -52.0946599
H -4.5868338 11.656628 -51.7934535
C -6.60297 12.3174183 -52.0927149
H -7.1427278 13.2367823 -52.3255887
H -6.9278242 11.951214 -51.1188279
H -6.8636599 11.5560362 -52.8261546

C -4.6265523 12.9470809 -53.5247128
H -5.2699191 13.744181 -53.8956398
H -3.6030739 13.3215718 -53.5000957
C -4.6555746 11.788384 -54.5300713
H -5.6777838 11.458972 -54.7113472
H -4.0625365 10.9539342 -54.1543668
H -4.2321241 12.1260136 -55.4769672
N -4.7271601 12.913471 -48.788543
H -3.775143 12.6272275 -48.9723444
C -5.2999645 12.5558781 -47.4919011
H -6.3581933 12.3426238 -47.6238231
C -5.12327 13.6827605 -46.4548777
O -3.9915082 13.9538372 -46.0403971
C -4.6452439 11.2446981 -47.0565748
H -3.587913 11.2809028 -47.3174804
H -5.1004324 10.4159779 -47.6001787
O -4.7366918 11.0128571 -45.6723862
H -5.6758702 11.0025864 -45.3613888
N -6.2181431 14.2734505 -45.9265974
C -6.1448261 15.2535177 -44.8392723
H -5.5037262 16.0768951 -45.1469994
C -5.5994107 14.6593758 -43.5365247
O -4.990551 15.3639187 -42.7414758
C -7.5767455 15.7683423 -44.6357585
H -8.056633 15.2366621 -43.8112928
H -7.5919532 16.8425494 -44.4489233
C -7.6005661 14.1249832 -46.3585443
H -7.6892693 14.0050037 -47.4364082
H -8.051605 13.2737373 -45.845865
C -8.2932888 15.4153499 -45.9362787
H -8.111043 16.1900229 -46.680453
H -9.3633916 15.2698172 -45.7853592
N -5.798928 13.3603836 -43.3244477
H -6.3334307 12.8472974 -44.0199305
C -5.3819279 12.5909009 -42.1533687
H -5.5086175 13.2194111 -41.2710266
C -3.889537 12.1953296 -42.1819728
O -3.2267231 12.2061379 -41.1417984
C -6.3412377 11.3905832 -41.9922851
H -7.3113502 11.7703061 -41.6675729
H -5.9575394 10.7498656 -41.1971473
C -6.5663179 10.5388814 -43.2538189
O -6.908747 11.078591 -44.3329274
O -6.3508923 9.3107568 -43.1812859
N -3.3008267 11.9683216 -43.3619274
H -3.8866016 11.9470974 -44.1933319

C -1.8430178 11.9831623 -43.5618261
H -1.3723892 11.257173 -42.9150736
C -1.2618897 13.3502526 -43.1982805
O -0.4067809 13.4546671 -42.3135894
C -1.4776396 11.6187214 -45.0103698
H -2.1218696 12.1563712 -45.7031098
H -0.4492902 11.9202012 -45.2048059
C -1.5826308 10.1109484 -45.2530965
H -2.542401 9.7508069 -44.8866851
H -0.8029225 9.6052696 -44.6769365
C -1.4265517 9.7523301 -46.7342513
O -2.1887154 8.9014817 -47.2378949
O -0.4387212 10.1870761 -47.3704002
N -1.8238071 14.3981459 -43.7998905
H -2.5652821 14.2183388 -44.4716944
C -1.3629664 15.7743681 -43.644951
H -0.2966211 15.7971953 -43.8619811
C -1.5011144 16.2671664 -42.1931932
O -0.5669668 16.8774303 -41.6842395
C -2.0937697 16.6432986 -44.6956286
H -3.1616424 16.4331521 -44.6208048
C -1.6453572 16.3062192 -46.1415492
H -1.8060816 15.2459139 -46.3228022
H -2.2857106 16.8442621 -46.8391984
C -1.9169274 18.1412204 -44.4519846
H -0.8624135 18.3648704 -44.4776171
H -2.351247 18.4235725 -43.4927015
H -2.4173335 18.7036014 -45.2397571
C -0.1850993 16.6247081 -46.5047295
H 0.502714 16.0852862 -45.8550906
H 0.005957 17.6948585 -46.4390428
H -0.0012286 16.3148047 -47.5317343
N -2.5671139 15.9256739 -41.4689525
H -3.3267024 15.4447242 -41.9411177
C -2.7735512 16.3191514 -40.0675664
H -2.7786111 17.4067291 -40.0129945
C -1.6436206 15.8404344 -39.1348382
O -1.1884872 16.6049745 -38.2795479
C -4.1712491 15.8244989 -39.6523631
H -4.8964345 16.3669418 -40.2600162
H -4.276831 14.7663523 -39.8970248
C -4.5547229 16.0502673 -38.1793378
H -5.6347657 16.1922723 -38.1337433
H -4.0852274 16.9595111 -37.8033673
C -4.2106249 14.87867 -37.2602295
O -3.478791 13.9624486 -37.5900405

N -4.7524033 14.838826 -36.0651956
H -4.5608134 14.0108061 -35.5281391
H -5.3835633 15.5549238 -35.7602172
N -1.1004638 14.6424705 -39.3708469
H -1.5013084 14.0764096 -40.106404
C 0.0418472 14.1322373 -38.6116889
H -0.0436926 14.4501244 -37.5713118
C 1.3850099 14.6518156 -39.1239408
O 2.2660308 14.8908551 -38.3035319
C 0.0435491 12.6104591 -38.6527647
H 1.0465421 12.2488474 -38.4138007
H -0.2262749 12.3017169 -39.6598596
S -1.1117245 11.9445062 -37.4241918
H -2.0986728 12.845829 -37.6105998
N 1.5575739 14.8170369 -40.4417058
H 0.8054631 14.5690666 -41.0762493
C 2.7701594 15.422793 -40.987119
H 3.6444812 14.9045317 -40.5883586
C 2.8696392 16.8863013 -40.5636503
O 3.5847836 17.2487937 -39.6283336
C 2.7907183 15.3258836 -42.5238653
H 1.9156234 15.7985274 -42.9702768
H 2.7822393 14.2994419 -42.8465516
H 3.7036186 15.7788831 -42.9060338
N 2.1002966 17.7180647 -41.2791163
H 1.1970245 17.3057366 -41.4945638
C 2.6117307 18.5517644 -42.3861192
H 3.1341734 17.8723516 -43.0516016
C 1.3970127 19.0600589 -43.2242848
H 1.358215 18.4262558 -44.0974844
H 0.5018309 18.6692998 -42.7733033
C 1.1166488 20.5727843 -43.5444475
H 1.2915895 21.151315 -42.6635274
H 1.8411256 20.9728381 -44.2381746
C -0.3230066 20.9360951 -43.9914732
H -1.0227159 20.3720331 -43.3859128
H -0.4699022 20.6178342 -45.0179328
C -0.6963571 22.4298742 -43.8025919
H 0.1995555 22.9755746 -43.4898631
H -1.4260614 22.4979107 -42.9928553
N -1.2635441 23.0865246 -45.012343
H -0.5895041 23.0906335 -45.7804691
H -1.4992876 24.0597524 -44.8902245
H -2.0834365 22.6400856 -45.4285437
C 3.7642777 19.4606364 -41.9383207
O 4.6934606 18.9814363 -41.3095185

N 3.6311882 20.7771193 -41.9989438
H 2.8876535 21.1417414 -42.5604266
C 4.0788258 21.6000937 -40.8865894
H 5.1237588 21.3834285 -40.6673247
C 3.9999911 23.0820359 -41.3327063
H 3.0493725 23.2473995 -41.8409777
H 4.7944901 23.2717171 -42.0553756
C 4.1026739 24.1179475 -40.2399803
N 3.0918861 25.0331692 -39.9409579
C 3.4223691 25.5589047 -38.7527186
H 2.8010558 26.2448663 -38.192991
N 4.5752577 25.0358408 -38.3042514
H 4.8930887 25.1113105 -37.3429746
C 5.0364284 24.1390869 -39.2428601
H 5.8685618 23.4582498 -39.1352582
C 3.2420288 21.199852 -39.6385361
O 3.0797663 22.025223 -38.7500341
N 2.530232 20.0383135 -39.640792
H 2.865367 19.2416506 -40.1623869
C 1.4823966 19.8353562 -38.7041374
H 1.2481924 20.7922532 -38.2384218
C 2.0858318 19.0933861 -37.5361898
O 3.067777 19.6165891 -37.0050026
C 0.1413169 19.4928001 -39.3442083
H 0.1442248 18.4588372 -39.6817178
H -0.6141341 19.569777 -38.5611991
C -0.267998 20.4123699 -40.5175301
H 0.1183068 19.9509151 -41.3678312
C -1.7776922 20.4353344 -40.70414
H -2.2492346 20.8376265 -39.8066394
H -2.1313518 19.4171078 -40.8620015
H -2.0806715 21.041301 -41.5528518
C 0.1944014 21.8747087 -40.5369619
H 1.2344464 22.003532 -40.7787416
H 0.035205 22.3050513 -39.5470494
H -0.3843236 22.4742456 -41.2283432
N 1.4878269 18.020571 -37.0275607
H 0.680745 17.619781 -37.490007
C 1.8370793 17.567107 -35.682473
H 1.6480608 18.3923588 -34.994206
C 3.3255548 17.1909504 -35.5105984
O 3.975249 17.6990186 -34.5966676
C 0.897452 16.4265003 -35.2938252
H 1.0844826 15.5580817 -35.9233497
H -0.1416476 16.7397962 -35.4098183
H 1.0723946 16.150676 -34.2533891

N 3.895938 16.3935091 -36.4204664
H 3.3273487 16.0340792 -37.1822979
C 5.2891756 15.9425756 -36.3358269
H 5.4806631 15.6036171 -35.3165186
C 6.3276383 17.0397494 -36.6405866
O 7.4910534 16.9063308 -36.2670223
C 5.4618095 14.744717 -37.274927
H 5.2733635 15.0489023 -38.3063018
H 4.7617187 13.9547081 -37.0015401
H 6.47971 14.3605827 -37.1961688
N 5.9285687 18.1328717 -37.2982429
H 4.962632 18.1795496 -37.6016175
C 6.8314934 19.2418052 -37.635999
H 7.8429605 18.8505207 -37.6832907
C 6.844892 20.3829303 -36.6121256
O 7.5699517 21.3645417 -36.8076774
C 6.4883151 19.7654549 -39.0249614
H 5.4763035 20.1549587 -38.9918722
H 6.5452826 18.940506 -39.7349417
O 7.3909096 20.7802827 -39.4271918
H 7.5897174 21.3108912 -38.6363837
N 6.0301942 20.3258281 -35.5583977
H 5.4633655 19.4990513 -35.4131305
C 5.9102691 21.4436743 -34.6242259
H 5.8569889 22.3420848 -35.2375565
C 7.1639527 21.593333 -33.7492034
O 7.6308825 20.6298769 -33.149975
C 4.6035516 21.3149437 -33.8160339
H 3.9610644 20.5586151 -34.2705869
H 4.8261419 20.983383 -32.8007128
C 3.8146552 22.6339158 -33.7630863
H 4.3761121 23.361526 -33.1761685
H 2.8597389 22.454182 -33.2696363
C 3.5503843 23.2164719 -35.1512038
O 4.0029425 24.3035214 -35.4804174
N 2.9488639 22.4629638 -36.0411139
H 2.950617 22.7735293 -37.0102779
H 2.7918841 21.4817724 -35.8719266
N 7.7210904 22.8081187 -33.6914249
H 7.2987774 23.5567177 -34.2190106
C 8.9972279 23.0788989 -33.0131334
H 9.0701395 24.1482844 -32.8145984
H 9.0214978 22.5526099 -32.0586359
C 10.2493908 22.6746477 -33.805537
O 11.3286693 22.5814544 -33.2268775
N 10.1271567 22.3925806 -35.1093067

H 9.2142375 22.4390954 -35.5407744
C 11.2828804 22.1739636 -35.9765919
H 12.1470555 21.9182832 -35.3639204
C 11.6311631 23.4425166 -36.7703543
O 10.8468171 23.8739616 -37.6062429
C 11.0248787 21.0051895 -36.9419587
H 10.2282802 21.2982624 -37.627223
H 11.9359448 20.8776523 -37.5163873
C 10.6521745 19.6372191 -36.3484215
H 9.6886744 19.7031776 -35.8480046
C 10.554009 18.6256642 -37.4922608
H 11.5421073 18.4392663 -37.908598
H 9.9033941 19.0025286 -38.2786216
H 10.1402736 17.6890333 -37.1141196
C 11.700169 19.1190278 -35.363519
H 12.6761902 19.0615675 -35.8409176
H 11.4049478 18.1266817 -35.0192001
H 11.7434391 19.7788958 -34.4960605
N 12.8948299 23.8661966 -36.7091582
H 13.3352564 23.7065076 -35.812184
C 13.4155394 25.0179326 -37.4790076
H 12.6681205 25.8145446 -37.4282954
C 13.6732908 24.7387133 -38.9836209
O 14.3679382 25.4938641 -39.6582383
C 14.7076514 25.5493859 -36.8171534
H 15.5330047 24.8870948 -37.0856383
H 14.9254605 26.5347873 -37.2302733
C 14.686988 25.6625318 -35.2845744
H 15.5866248 26.1902592 -34.9596257
H 14.7320807 24.6561241 -34.8586402
C 13.4378158 26.3886491 -34.7758366
O 13.4280188 27.6368107 -34.819975
O 12.4900572 25.6633847 -34.4019596
N 13.2648524 23.5772005 -39.5105643
H 12.5354699 23.085907 -39.0068225
C 13.5570177 23.1795007 -40.8976494
H 14.5254876 23.6009869 -41.1761233
C 12.542468 23.7627457 -41.8860466
O 11.3445727 23.7642826 -41.6133993
C 13.6717175 21.6505914 -41.0511484
H 14.6737933 21.3409764 -40.7633607
H 13.5409867 21.3814689 -42.0977122
C 12.6807254 20.8409029 -40.234541
O 13.0819143 19.9571612 -39.4998137
N 11.4063889 21.1652132 -40.2593715
H 10.7663929 20.6171536 -39.7086994

H 11.0835354 21.9909526 -40.7547637
N 13.0133136 24.1123106 -43.0888082
H 14.003369 23.9861169 -43.252692
C 12.2559559 24.8063065 -44.14478
H 11.634147 25.5703974 -43.6725806
C 11.2900693 23.8636724 -44.8836361
O 11.4794884 23.5296958 -46.0528063
C 13.2402964 25.5207442 -45.0935189
H 13.7974318 24.7585126 -45.6299273
H 12.6747639 26.1075278 -45.8195301
C 14.2367 26.4457212 -44.363787
H 13.6782029 27.2076148 -43.8182901
H 14.8081668 25.8651489 -43.6389009
C 15.2530414 27.1445263 -45.2802328
H 15.9662655 27.6665972 -44.6388995
H 14.7410762 27.8844194 -45.8974409
C 16.0017683 26.1590706 -46.1895801
H 15.4383225 26.0365783 -47.1185131
H 16.0485386 25.1791219 -45.7028254
N 17.3833513 26.6013149 -46.4784672
H 17.4501689 27.5257558 -46.8646949
H 17.8394313 25.9286781 -47.0934372
H 17.9672793 26.5200239 -45.6378488
N 10.2586788 23.4106932 -44.1767312
H 10.1841574 23.7178249 -43.2124954
C 9.2046284 22.5279462 -44.6902692
H 9.4646982 22.2318761 -45.7066837
C 7.8664666 23.2561846 -44.773848
O 7.5173305 24.0732073 -43.9268036
C 9.0728533 21.2239852 -43.8778855
H 8.316634 20.6091786 -44.3642886
C 10.3766276 20.4230278 -43.8853184
H 11.1563743 20.9672999 -43.3545985
H 10.6901088 20.2389319 -44.9118237
H 10.2204614 19.4614051 -43.3955968
C 8.6444678 21.4437578 -42.4225151
H 9.3296493 22.1190396 -41.9159669
H 8.6253233 20.491972 -41.8909169
H 7.6444986 21.8751438 -42.3818422
N 7.0848025 22.9020958 -45.7835561
H 7.4542566 22.2468272 -46.4660422
C 5.688535 23.2901281 -45.9603944
H 5.2592379 23.5642908 -44.9971347
C 4.9368154 22.090787 -46.5162855
O 5.5175932 21.2799915 -47.2402207
C 5.572196 24.4861637 -46.9093264

H 6.1315988 25.3254024 -46.4927753
H 4.5253632 24.7775338 -47.0057321
O 6.0920421 24.166681 -48.1936715
H 6.9019288 23.6412406 -48.069159
N 3.6649475 21.9328174 -46.1572492
H 3.1924113 22.6509743 -45.6290061
C 2.8540294 20.891737 -46.7718369
H 3.2603949 20.7420939 -47.768523
C 1.4232672 21.3469321 -46.9993605
O 0.9655742 22.2461308 -46.2958749
C 2.8723023 19.5311039 -46.0727033
H 1.8998189 19.3593649 -45.6756641
H 2.831625 18.8431272 -46.9167144
C 4.0238134 18.9905664 -45.2169582
C 4.3071869 17.6253821 -45.3860567
H 3.6888653 17.0224533 -46.0309537
C 4.8919789 19.744078 -44.3979068
H 4.7530479 20.7996534 -44.2590226
C 5.3992881 17.0235059 -44.752768
H 5.5957283 15.9801922 -44.9285487
C 5.9619852 19.1264943 -43.7177794
H 6.5828412 19.700868 -43.0462872
C 6.2154413 17.761582 -43.8888187
H 7.0295482 17.2877361 -43.3583586
N 0.7049211 20.6843602 -47.9071269
H 1.1580329 19.9917536 -48.4999263
C -0.7426341 20.8387991 -48.0115702
H -1.0824204 20.9641876 -46.9898499
C -1.5361428 19.617803 -48.4852348
O -0.9924186 18.6595985 -49.0384563
C -1.11455 22.1121096 -48.8003637
H -1.7026334 21.8618073 -49.6844732
H -0.2187602 22.6483505 -49.119513
C -1.9431406 22.9948283 -47.8556361
O -2.8487189 22.4398679 -47.1824651
O -1.4945848 24.1167787 -47.540539
N -2.8489941 19.6723543 -48.2388324
H -3.2118883 20.5386921 -47.8451067
C -3.8093695 18.7281656 -48.8164363
H -3.3973647 17.7249245 -48.7310293
C -3.9734597 19.0752603 -50.2936677
O -4.3973474 20.175993 -50.6415666
C -5.1732061 18.7435119 -48.0992768
H -5.6730347 19.6950951 -48.2825975
C -6.0637773 17.6068255 -48.6193746
H -5.6052813 16.639463 -48.4108445

H -6.2151491 17.7031978 -49.6940242
H -7.0395154 17.6576898 -48.1394967
C -5.0359728 18.5600012 -46.5826229
H -4.4763312 17.6507279 -46.373113
H -6.0235974 18.4938287 -46.1264681
H -4.5200769 19.4177832 -46.1531065
N -3.6215795 18.1436875 -51.1741785
H -3.2879382 17.2507786 -50.8282856
C -3.6115106 18.3903012 -52.6077545
H -4.5005105 18.9612831 -52.879691
H -2.7342712 18.9845956 -52.864576
C -3.5764643 17.1062873 -53.4192378
O -2.760003 16.2192524 -53.1788795
N -4.4347018 17.0516582 -54.4326559
H -5.0732818 17.8204822 -54.5581563
C -4.3864658 16.05468 -55.4956002
H -4.0915978 15.0912157 -55.0883004
C -3.3390088 16.4662952 -56.5310674
O -3.4495113 17.5140222 -57.164842
C -5.7890218 15.9175663 -56.0942179
H -6.1264015 16.9003061 -56.4288134
H -6.4684305 15.5787826 -55.3090581
C -5.884318 14.9468145 -57.2730016
O -4.8383242 14.4918336 -57.7921059
O -7.0322555 14.7422459 -57.7062923
N -2.3237965 15.6294866 -56.7222922
H -2.3348741 14.7515165 -56.2290766
C -1.1959614 15.917138 -57.5968154
H -0.8042434 16.9026035 -57.3349828
C -1.5477935 15.9520276 -59.0940706
O -0.7986989 16.5414193 -59.8719076
C -0.1261968 14.8713849 -57.2991538
H -0.4332321 13.8964363 -57.6832249
H 0.0211865 14.8034629 -56.2217764
H 0.8044678 15.171372 -57.7763393
N -2.6894224 15.3899282 -59.5099429
H -3.3057519 14.9434825 -58.8268714
C -3.1680469 15.5081525 -60.8889374
H -2.2977069 15.4764396 -61.5426236
C -3.8714604 16.8578481 -61.1353749
O -3.8881519 17.3427345 -62.2717246
C -4.0589673 14.2899356 -61.2019821
H -3.5800509 13.3944349 -60.8022451
H -5.0094949 14.4145144 -60.6792547
C -4.3336983 14.054691 -62.7013104
H -4.8066073 14.9365606 -63.1323418

C -3.0607759 13.732287 -63.4923323
H -2.5395826 12.8897156 -63.037984
H -2.3987076 14.5952794 -63.5160859
H -3.3198034 13.4669686 -64.5171777
C -5.2831101 12.8667808 -62.868187
H -4.8269732 11.9632398 -62.4652122
H -5.5106869 12.7195113 -63.9246257
H -6.2135557 13.0676634 -62.3361046
N -4.3438173 17.5317856 -60.0767708
H -4.239743 17.0913676 -59.1689351
C -5.1392953 18.7670931 -60.1508346
H -5.3101043 19.014173 -61.1954728
C -4.4652452 20.0192241 -59.553186
O -4.7247452 21.1197762 -60.036646
C -6.5275032 18.4864972 -59.5562995
H -7.1041324 19.4099779 -59.5149897
H -6.4332717 18.0886791 -58.546576
C -7.2894454 17.5033969 -60.4354308
O -7.5563357 17.7986585 -61.5883171
N -7.5704357 16.2932746 -60.0008698
H -8.0536807 15.6856149 -60.632247
H -7.3300548 15.9419432 -59.0695682
N -3.5291447 19.8666245 -58.6129897
H -3.3778296 18.9328279 -58.2480178
C -2.7161973 20.948246 -58.0426512
H -3.3974747 21.6988151 -57.6398965
C -1.8381347 21.6244684 -59.108357
O -1.3603305 20.9679296 -60.0376461
C -1.8708616 20.3801509 -56.8857777
H -1.2155043 19.5960797 -57.2688558
H -2.5403692 19.9339514 -56.1488536
C -1.0113161 21.4298223 -56.1713507
H -0.2443703 21.7912918 -56.8567282
H -0.5059511 20.9491715 -55.3327881
S -1.9402459 22.8506813 -55.5394919
C -0.5701828 23.8292679 -54.8690719
H -0.9613234 24.74235 -54.4197375
H 0.1193026 24.0926016 -55.6715556
H -0.0410525 23.252321 -54.1098706
N -1.6015408 22.9369568 -58.9811233
H -1.9423219 23.4054305 -58.1486339
C -0.8192637 23.7390643 -59.9395053
H -0.2576772 23.064157 -60.5851844
C 0.2282464 24.5957342 -59.2270807
O 0.004776 25.0706294 -58.1183271
C -1.7509353 24.6002576 -60.822006

H -2.2485006 25.3361505 -60.1875853
H -1.1470927 25.1432575 -61.5512182
C -2.8355023 23.8041089 -61.576147
H -3.4692309 24.5095959 -62.1148217
H -3.4670243 23.293783 -60.85025
C -2.285634 22.7794283 -62.5838831
H -1.469829 22.2166229 -62.1362081
H -1.8727529 23.3160784 -63.440655
N -3.3480238 21.8600974 -63.0454371
H -4.1592275 22.2924263 -63.4494333
C -3.3712553 20.539867 -62.9367282
N -4.3632524 19.8552986 -63.436589
H -4.394121 18.8635073 -63.2050823
H -5.2093213 20.3214733 -63.7056537
N -2.4470361 19.8412767 -62.3456859
H -1.8072108 20.3142319 -61.7116381
H -2.5589807 18.8412342 -62.2636225
N 1.3477479 24.8147152 -59.9086339
H 1.3980397 24.4714973 -60.8561952
C 2.4801481 25.6382775 -59.4817666
H 2.171737 26.310206 -58.6796175
C 2.9540436 26.4820188 -60.6742369
O 2.5493109 26.2124622 -61.8048482
C 3.6101897 24.7291168 -58.9715134
H 3.9556652 24.0994097 -59.7921348
H 4.4519802 25.3525014 -58.6710504
C 3.2414508 23.8502424 -57.790578
C 3.2255769 24.3988016 -56.4940977
H 3.4578802 25.4439975 -56.3432923
C 2.944317 22.484486 -57.9805326
H 2.957988 22.0603603 -58.9749413
C 2.9118273 23.5871558 -55.3886793
H 2.893409 23.9989979 -54.390721
C 2.6376701 21.6672874 -56.8746023
H 2.4174114 20.6212776 -57.0198648
C 2.6196309 22.2218223 -55.5761608
O 2.3179465 21.4526177 -54.4997302
H 2.18435 20.5319836 -54.724826
N 3.8334992 27.4628261 -60.4509498
H 4.2082822 27.584594 -59.5219213
C 4.4939213 28.1620494 -61.5616058
H 3.7309422 28.5488971 -62.2384471
C 5.3882736 27.2020676 -62.3485732
O 6.0176283 26.3114741 -61.7616727
C 5.3217809 29.3477141 -61.0436929
H 6.091829 28.9980095 -60.3569351

H 4.6612574 30.029898 -60.5071673
O 5.9393037 30.0538829 -62.1032955
H 6.7243398 29.5613144 -62.4093461
N 5.5820288 27.4940815 -63.6317522
H 5.0106585 28.1958685 -64.0746187
C 6.7307802 26.9874338 -64.3760478
H 6.6366855 25.9085016 -64.4254029
C 8.028407 27.3558119 -63.6304401
O 8.1187891 28.4400932 -63.0417593
C 6.7479946 27.5450176 -65.8100117
H 7.719706 27.3149662 -66.2505881
H 6.6485008 28.6315395 -65.7731873
C 5.6672195 26.9697377 -66.7358325
O 4.772257 26.2412801 -66.2508656
O 5.7522914 27.2543315 -67.9504335
N 9.0046514 26.4438149 -63.5827792
H 8.8767 25.5757138 -64.0999271
C 10.2928452 26.6834173 -62.9168859
H 10.9628395 25.863104 -63.1596956
H 10.726691 27.5994604 -63.3176861
C 10.2581107 26.8168125 -61.3836782
O 11.1929088 27.3706979 -60.812188
N 9.2068629 26.3463782 -60.7000323
H 8.4713915 25.8971 -61.2280263
C 9.0731089 26.4374344 -59.2283582
H 9.1851045 27.4791121 -58.9258279
C 10.1069491 25.6136815 -58.4432941
O 10.4836519 25.994552 -57.3288833
C 7.6897912 25.9495096 -58.7796666
H 7.6527518 25.9341602 -57.6888603
H 7.5223822 24.9374057 -59.148062
O 6.6580577 26.7898418 -59.2502181
H 6.5249451 26.5892104 -60.1998395
N 10.5092653 24.4553591 -58.9775317
H 10.148396 24.2047745 -59.8925566
C 11.2703098 23.4359545 -58.2511685
H 11.53296 23.831252 -57.2725145
C 12.5826537 23.0539197 -58.9343996
O 12.7237566 23.0892687 -60.153064
C 10.4016771 22.1945978 -58.0069762
H 10.1678104 21.7295215 -58.9665107
H 10.9725225 21.4720044 -57.4234963
C 9.1174901 22.4918067 -57.2616053
C 9.1440574 22.7882535 -55.8861933
H 10.0770687 22.7663086 -55.3446341
C 7.8965917 22.5106125 -57.9559699

H 7.8822044 22.2996273 -59.0114969
C 7.9532069 23.1130515 -55.2127899
H 7.9771022 23.3415634 -54.1554797
C 6.7052395 22.8277978 -57.284616
H 5.7704859 22.841606 -57.8236995
C 6.7337942 23.135257 -55.9133376
H 5.8189192 23.3828901 -55.3936062
N 13.517583 22.5839715 -58.1188466
H 13.3072881 22.5350507 -57.1278463
C 14.8179288 22.0601079 -58.5444263
H 15.0314648 22.3351386 -59.5799209
C 14.8820061 20.5283609 -58.4571595
O 15.7122647 19.8905221 -59.1081171
C 15.861854 22.6970743 -57.6264017
H 15.4958654 22.6849266 -56.6000881
H 16.7664742 22.1004187 -57.6568063
C 16.1785143 24.1454572 -58.0345006
H 16.4935678 24.6931106 -57.143149
H 15.2907343 24.6425045 -58.4312527
C 17.305602 24.1476694 -59.067901
O 17.1190339 23.5958259 -60.1692975
O 18.44763 24.4781666 -58.6847995
N 14.0008482 19.9431131 -57.6415347
H 13.375073 20.5442361 -57.11928
C 13.8557196 18.5135042 -57.3961515
H 14.3000178 17.9505765 -58.2132188
C 12.3562485 18.1809218 -57.2916426
O 11.6055412 18.9392478 -56.6695756
C 14.5613807 18.1437631 -56.0764476
H 13.9703084 18.5856413 -55.2756477
H 14.5331341 17.0604963 -55.9537592
C 16.0075358 18.6382877 -55.8749913
H 16.0774433 19.7022195 -56.0961422
C 16.4272853 18.4705187 -54.4170553
H 16.4803628 17.4129 -54.1558347
H 15.7119752 18.972793 -53.7650525
H 17.3987076 18.9361346 -54.2814031
C 17.0207935 17.8886108 -56.7338335
H 16.9321508 16.814618 -56.5697651
H 18.0321975 18.2073195 -56.4892937
H 16.8337156 18.1120355 -57.7796173
N 11.9268476 17.0254245 -57.802589
H 12.5667476 16.4679935 -58.3580903
C 10.6134922 16.4408486 -57.4727742
H 10.200647 16.9817986 -56.6240596
C 10.8460628 14.9974708 -57.0338666

O 11.4968357 14.2196056 -57.7343886
C 9.584129 16.5453119 -58.623281
H 9.8891 15.8453796 -59.384544
C 9.5544193 17.9798466 -59.2141413
H 9.2019235 18.6905423 -58.4697116
H 10.5636784 18.2729503 -59.500348
C 8.1844474 16.0604285 -58.2049168
H 7.7911132 16.6907992 -57.4099126
H 8.2346799 15.0332518 -57.8434264
H 7.5072373 16.083636 -59.0554359
C 8.7221937 18.1222675 -60.4791129
H 7.6661354 18.1114382 -60.2270789
H 8.9737782 17.3097286 -61.1536164
H 8.9770545 19.0592215 -60.9698104
N 10.3564225 14.6574374 -55.8469035
H 9.7941865 15.3397182 -55.3446681
C 10.5610505 13.361721 -55.2088406
H 11.1527113 12.721799 -55.8623133
C 9.2265463 12.6740554 -54.9607565
O 8.2716464 13.3041168 -54.5053329
C 11.33476 13.5316808 -53.9000093
H 12.3374437 13.8914798 -54.1303004
H 10.8329836 14.2807638 -53.2847405
C 11.4560679 12.2414929 -53.107116
C 12.2584577 11.1893237 -53.5913009
H 12.8093437 11.3079028 -54.5115507
C 12.3285074 9.9712448 -52.892086
H 12.935959 9.1595666 -53.2719667
C 11.6002187 9.80203 -51.7022888
H 11.6597189 8.8679635 -51.1608939
C 10.7885537 10.8448729 -51.2227153
H 10.2173261 10.7141468 -50.3149552
C 10.7115946 12.0603749 -51.9250526
H 10.0647863 12.8469327 -51.5572318
N 9.1602094 11.3777451 -55.2613215
H 9.967209 10.9333375 -55.6892098
C 7.9328766 10.5948167 -55.1189933
H 7.301889 11.0701356 -54.3646456
C 8.255057 9.1831792 -54.6477118
O 9.0891373 8.509012 -55.2435789
C 7.1597415 10.5440188 -56.4471444
H 7.7114147 9.9222563 -57.1494066
C 5.8095002 9.8629732 -56.2113163
H 5.4896513 10.0212756 -55.1882349
H 5.9140703 8.7885214 -56.3956002
H 5.0585275 10.2781186 -56.8705113

C 7.0334598 11.9183157 -57.1168015
H 6.7794035 12.6694729 -56.3779092
H 6.2773547 11.9109892 -57.8926942
H 7.9869524 12.1928344 -57.5734035
N 7.5304832 8.6922801 -53.6430702
H 6.8760449 9.3036927 -53.1726615
C 7.6681163 7.3252057 -53.1358409
H 8.2068826 6.7406371 -53.8801015
C 6.2876119 6.6673452 -52.9862792
O 5.4097723 7.1751835 -52.2984167
C 8.5623854 7.3067255 -51.8731256
H 9.5579647 7.6240205 -52.1890165
C 8.094785 8.2976582 -50.7891014
H 7.0661867 8.0877915 -50.5071326
H 8.1462975 9.3195321 -51.1659953
H 8.7354529 8.2405992 -49.9112774
C 8.6896144 5.865025 -51.3446308
H 7.7397634 5.5693468 -50.9079072
H 8.8984866 5.198318 -52.1818034
C 9.804598 5.6643442 -50.3096542
H 9.5898272 6.2220642 -49.3977556
H 10.7584064 5.9929064 -50.7211156
H 9.8773923 4.6049462 -50.0589894
N 6.0735872 5.5791504 -53.7322323
H 6.8322139 5.3057652 -54.3480851
C 4.8587987 4.7465541 -53.7745885
H 4.9169297 4.1923917 -54.7089597
C 3.516118 5.5057891 -53.8614048
O 2.5191161 5.1118456 -53.2532807
C 4.8607276 3.7088065 -52.6438668
H 4.6254463 4.2273965 -51.7263386
H 4.0580549 2.9937707 -52.8329263
C 6.1573545 2.9237375 -52.4338794
H 5.9667513 2.1123083 -51.7318169
H 6.9089625 3.5794581 -51.9982039
C 6.7097361 2.3381834 -53.7201912
O 7.8204849 2.6367907 -54.124086
N 5.9596121 1.5408023 -54.4434662
H 6.3860314 1.1601171 -55.2659575
H 4.9443976 1.5731702 -54.3602218
N 3.4922299 6.609794 -54.6151327
H 4.3734201 6.9183661 -54.9925454
C 2.3102518 7.4619524 -54.7883182
H 1.523502 7.1422596 -54.1042785
C 1.7272705 7.3825057 -56.209906
O 0.5213259 7.2247492 -56.3878293

C 2.7044591 8.890572 -54.3960494
H 3.4554001 9.2689719 -55.0839756
H 3.1257726 8.8895343 -53.3873539
S 1.267988 9.9956678 -54.455393
H 1.9581013 11.1116626 -54.1764562
N 2.5785643 7.3956426 -57.2408166
H 3.5667076 7.4519611 -57.0465178
C 2.1412431 7.4998195 -58.6453044
H 1.3706166 8.2684934 -58.6961616
C 1.455654 6.224998 -59.1639819
O 0.6846533 6.2812467 -60.1185169
C 3.3271499 7.9979964 -59.498981
H 4.2271993 7.4604443 -59.1935633
C 3.5120954 9.5030321 -59.2234801
H 2.7433385 10.070109 -59.7525158
H 3.3888159 9.7067547 -58.1618482
C 3.1360484 7.7830679 -61.0060698
H 2.1735433 8.1823495 -61.3276595
H 3.2063335 6.7193164 -61.2171017
H 3.9295564 8.2650958 -61.5743967
C 4.8911054 10.0097662 -59.6504475
H 5.0099305 9.9628237 -60.7314375
H 5.6735898 9.4236341 -59.1685517
H 4.9797716 11.0455733 -59.3443017
N 1.5717582 5.1041732 -58.4564899
H 2.1827045 5.1206104 -57.6529454
C 0.7328439 3.9149034 -58.651388
H 0.8947388 3.5462889 -59.6639284
C -0.7823536 4.1812482 -58.5331897
O -1.5480582 3.5308124 -59.2486143
C 1.1982313 2.8047712 -57.6992863
H 0.5310809 1.9524817 -57.8022436
H 2.1807305 2.4791663 -58.0396875
C 1.2976814 3.1948038 -56.2119626
H 0.3012642 3.1688227 -55.7657597
H 1.6944627 4.205053 -56.0979221
C 2.2384522 2.2254211 -55.4939914
O 3.4720441 2.4194285 -55.590334
O 1.7426194 1.2099946 -54.9540701
N -1.2002262 5.2068174 -57.7761104
H -0.5129244 5.7123035 -57.2254629
C -2.598939 5.6605511 -57.6700557
H -3.2650489 4.7962077 -57.7183515
C -3.0249164 6.6303356 -58.7861373
O -4.212663 6.7435325 -59.0897442
C -2.8084363 6.3407486 -56.3074555

H -3.8525083 6.6461592 -56.2269157
H -2.1951503 7.2411331 -56.2540386
C -2.494111 5.4648105 -55.1222584
N -3.2748369 4.3995001 -54.6813414
C -1.402258 5.5724542 -54.3123075
H -0.6083186 6.2963122 -54.4092074
C -2.6439835 3.8919381 -53.61534
H -2.9894624 3.0311948 -53.0587202
N -1.5076655 4.5702295 -53.3754836
H -0.8044458 4.3204002 -52.6953078
N -2.0794667 7.3391896 -59.4136958
H -1.1158875 7.1556197 -59.1719981
C -2.3616997 8.3287098 -60.4667779
H -3.2276859 8.9173499 -60.1602128
C -2.7464924 7.5875773 -61.7548098
O -2.001621 6.7304906 -62.2333305
C -1.169923 9.3091347 -60.6337605
H -0.2399088 8.7535995 -60.5132491
C -1.1908147 10.461597 -59.5993055
H -0.3002541 11.0742561 -59.749503
H -2.0610367 11.0934907 -59.7853356
C -1.1248122 9.968303 -62.0256172
H -2.0519598 10.5058078 -62.2273489
H -0.9656117 9.221869 -62.8030621
H -0.2929621 10.6711498 -62.0761009
C -1.2165483 10.0459058 -58.1225757
H -0.4028053 9.3553923 -57.9105932
H -2.1699243 9.5788426 -57.8756608
H -1.0975652 10.9321865 -57.4986943
N -3.9191623 7.9002296 -62.3131057
H -4.4943561 8.5906527 -61.8567087
C -4.4811881 7.1647627 -63.4544377
H -4.2521094 6.1048541 -63.3300885
C -3.8496852 7.5951677 -64.7889643
O -3.2727482 6.7640869 -65.4867586
C -6.0132403 7.3088003 -63.4796484
H -6.2694267 8.3537053 -63.6622205
H -6.4039576 6.7184002 -64.3096062
C -6.7166646 6.8608593 -62.1861027
H -7.7887787 7.0203155 -62.3018223
H -6.3872767 7.4812947 -61.3521355
C -6.4970237 5.3889815 -61.8459751
O -6.9995508 4.4963847 -62.5114945
N -5.7572628 5.0902401 -60.8042215
H -5.833909 4.14822 -60.4307188
H -5.3482376 5.8097848 -60.2156199

N -3.8569681 8.8981386 -65.0946658
H -4.3202539 9.5439063 -64.4785248
C -3.1401507 9.4583446 -66.2477274
H -3.0411782 8.6833109 -67.0107969
C -1.7236527 9.8698317 -65.8282051
O -1.4618108 10.9868417 -65.364135
C -3.9305454 10.5951731 -66.9132344
H -4.0322571 11.4261952 -66.2131145
H -4.9304066 10.2295566 -67.1538309
C -3.2659619 11.0967388 -68.2087631
O -2.0957811 10.7292587 -68.4770125
O -3.9030893 11.919111 -68.8986723
N -0.803794 8.9111941 -65.9612056
H -1.116004 8.0117478 -66.302686
C 0.6148988 9.1220086 -65.6716635
H 0.701243 9.6227611 -64.7065084
C 1.2656105 10.0741342 -66.6689175
O 2.098196 10.8648421 -66.2441968
C 1.3529267 7.7757301 -65.5926566
H 1.1126767 7.1689474 -66.4679944
H 2.4297305 7.955249 -65.5762918
C 0.9574017 7.0366377 -64.3062496
H 1.2856483 7.6316669 -63.4540904
H -0.1280695 6.9419732 -64.2625537
C 1.5717334 5.633152 -64.2128231
H 2.6407791 5.6991469 -64.4166036
H 1.1128656 4.9710833 -64.9484462
C 1.3819517 5.0770151 -62.7971826
H 1.7874912 5.8138986 -62.1066302
H 1.9538723 4.1524789 -62.6816544
N -0.0351696 4.8430507 -62.4374898
H -0.6226182 5.656999 -62.6128357
H -0.1159741 4.6848988 -61.4413045
H -0.410129 4.0257107 -62.9061462
N 0.857613 10.1083443 -67.9375912
H 0.0306158 9.5949737 -68.2147783
C 1.4627276 11.0419099 -68.8908006
H 2.5495178 10.9536548 -68.8456466
C 1.0955327 12.4971915 -68.5945729
O 1.9570158 13.368256 -68.6695282
C 1.0240044 10.7936445 -70.3246517
H -0.0383867 11.0268319 -70.4208665
H 1.5987591 11.5133366 -70.9013528
C 1.2870139 9.4334093 -70.9591885
H 0.6207275 8.6875652 -70.5204886
H 2.321262 9.1555211 -70.7495981

C 1.0670261 9.5267931 -72.4818624
O 1.5837226 8.6346019 -73.1863309
O 0.449561 10.5278113 -72.9382914
N -0.1651418 12.7909911 -68.2489103
H -0.8538143 12.0316837 -68.2624408
C -0.597046 14.1400191 -67.8500139
H -0.3606733 14.8368522 -68.6493023
C 0.1788132 14.6128788 -66.6331437
O 0.6875716 15.7306213 -66.6664212
C -2.1126775 14.1466946 -67.5941196
H -2.6227997 14.0571914 -68.5530931
H -2.3576696 13.272807 -66.9910062
C -2.6896842 15.3612165 -66.8459386
H -3.7652708 15.2087699 -66.7430743
H -2.2667664 15.3935762 -65.8416272
C -2.4521123 16.7168604 -67.5216241
H -2.901966 16.7154243 -68.5155877
H -1.3820194 16.9074539 -67.6060141
C -3.0951271 17.8042082 -66.6541248
H -2.7560098 17.6658543 -65.6223159
H -4.1813943 17.6762267 -66.6795216
N -2.71647 19.159312 -67.1134731
H -1.700728 19.2804625 -67.0277463
H -3.1123092 19.8764223 -66.5258363
H -2.9676407 19.3212056 -68.075571
N 0.3204954 13.766832 -65.6119406
H -0.1301998 12.8600258 -65.6626692
C 1.1372004 14.0988161 -64.4489276
H 0.7681291 15.0311553 -64.0186157
C 2.6010132 14.3488103 -64.8440332
O 3.1203401 15.4231067 -64.5515805
C 1.0048208 13.0052398 -63.3833035
H -0.0274297 12.9715737 -63.0338871
H 1.2416141 12.0362933 -63.8251743
C 1.9196494 13.2544928 -62.2026704
C 1.5902525 14.2330469 -61.2449909
H 0.6507624 14.7645696 -61.3091331
C 2.5054578 14.5589645 -60.2283914
H 2.2645 15.3361693 -59.5185997
C 3.7477584 13.9046379 -60.163313
H 4.4610224 14.1635952 -59.3939693
C 4.075634 12.9324819 -61.1222156
H 5.0420339 12.4538226 -61.0965527
C 3.1634757 12.6011592 -62.1355357
H 3.4384687 11.8742898 -62.8856969
N 3.2219607 13.4431795 -65.6129092

H 2.7236004 12.5935768 -65.8627977
C 4.6285742 13.5613066 -66.0272132
H 5.2190656 13.6913037 -65.136371
C 4.8554892 14.8103973 -66.9071792
O 5.7538651 15.5982238 -66.6248052
C 5.1469566 12.2423806 -66.6375441
H 4.5264298 12.0049949 -67.503892
C 6.6112232 12.3569321 -67.0848273
H 7.294004 12.1712733 -66.2581363
H 6.8394987 13.3368053 -67.4929083
H 6.78462 11.6171695 -67.8603109
C 5.0988351 11.0728698 -65.6156212
H 5.8917595 11.1882859 -64.8758281
H 4.1709873 11.0890031 -65.0529447
C 5.2309691 9.6913749 -66.2729081
H 6.195366 9.5950969 -66.7695518
H 4.4343643 9.5515472 -67.0036455
H 5.1519131 8.9179254 -65.5089451
N 3.9636351 15.1292163 -67.8520424
H 3.2265652 14.4600239 -68.0547949
C 4.0218868 16.3871815 -68.6226211
H 5.0042765 16.4596209 -69.0901891
C 3.8797531 17.6361744 -67.751329
O 4.3853571 18.6900604 -68.1393617
C 2.9423549 16.3991231 -69.7126516
H 1.9845357 16.1193492 -69.2705717
H 2.8543825 17.4158555 -70.0989016
C 3.2701847 15.4763868 -70.8965743
H 3.2834725 14.434007 -70.5792135
H 4.2600221 15.7274747 -71.2810722
C 2.2462379 15.6651832 -72.0260354
H 2.5661878 15.0769262 -72.8877034
H 2.2537104 16.7118842 -72.3344896
N 0.873631 15.2917612 -71.613203
H 0.3736673 15.9475456 -71.0440993
C 0.2606047 14.1451865 -71.8617678
N 0.8324717 13.2109762 -72.562001
H 0.4215544 12.2750905 -72.7048818
H 1.7817286 13.3170273 -72.8573687
N -0.9394563 13.9204432 -71.4047869
H -1.4254517 14.5628763 -70.815964
H -1.3235025 12.9947997 -71.5411287
N 3.1468849 17.5726836 -66.640555
H 2.7147858 16.6893069 -66.3908726
C 3.0422072 18.6861717 -65.6891141
H 2.9536692 19.6115987 -66.2544648

C 4.3172776 18.8331359 -64.8542892
O 4.818539 19.9522103 -64.7316767
C 1.7891423 18.5422215 -64.8007602
H 1.0555546 17.9044931 -65.2924164
H 2.0612306 18.0606817 -63.8602092
C 1.1195113 19.8957002 -64.5028273
H 1.8473703 20.5638183 -64.0369374
H 0.3048766 19.7346467 -63.7929669
C 0.5491297 20.5310302 -65.7790337
O -0.2282403 19.8629453 -66.4986209
O 0.9457575 21.6510811 -66.1609826
N 4.94132 17.725144 -64.4234338
H 4.4998686 16.8199543 -64.5638902
C 6.2140599 17.8019982 -63.6909489
H 6.0524835 18.5815138 -62.9562327
C 7.3890477 18.3481159 -64.518059
O 8.2732855 18.9822581 -63.9445966
C 6.5604161 16.5360761 -62.8697259
H 7.6036421 16.612572 -62.5826921
C 5.687129 16.5745079 -61.5928064
H 4.6438896 16.3754152 -61.8451636
H 5.7360653 17.5498829 -61.1123979
H 6.0256541 15.83006 -60.8757306
C 6.3611885 15.1622226 -63.5084856
H 5.298497 14.9474787 -63.5301493
H 6.74844 15.1843201 -64.5161972
C 7.0610066 14.0140301 -62.7739185
H 6.6684855 13.8981371 -61.7667884
H 8.122155 14.2216184 -62.7084251
H 6.9110273 13.0840706 -63.3228865
N 7.30301 18.2991724 -65.852446
H 6.594574 17.6978391 -66.2541671
C 8.2430857 18.9945263 -66.7521705
H 9.256973 18.7089679 -66.4899572
C 8.1690656 20.51075 -66.5818082
O 9.1990222 21.1611148 -66.460308
C 7.977772 18.6399987 -68.229651
H 6.9892592 19.0220523 -68.484297
C 8.9845327 19.2914653 -69.1874602
H 10.0014391 18.9979181 -68.9194354
H 8.9126701 20.3778295 -69.1491886
H 8.7886603 18.966429 -70.2109033
C 7.96589 17.1408008 -68.5243393
H 8.943109 16.8146886 -68.8779901
H 7.2357508 16.9438971 -69.3046957
H 7.7171778 16.551408 -67.6508645

N 6.9615765 21.0893303 -66.532496
H 6.1488449 20.486938 -66.5022801
C 6.780615 22.5474602 -66.4135413
H 7.4330732 23.0541204 -67.1286945
C 7.2202667 23.0717325 -65.0569848
O 7.912679 24.0815246 -64.9821131
C 5.3159302 22.9327156 -66.6760503
H 4.6831159 22.049465 -66.5844177
H 4.9780527 23.653042 -65.9286919
C 5.1585947 23.5769347 -68.0589042
H 5.5905341 22.9257093 -68.8152424
H 5.7275276 24.5057287 -68.0837541
C 3.6905468 23.8966561 -68.3952165
H 3.6380314 24.2920585 -69.4108383
H 3.3606373 24.6917945 -67.7218206
N 2.7656957 22.7454938 -68.2527494
H 2.0283473 22.845749 -67.5579178
C 2.88091 21.5274568 -68.7454514
N 3.8693124 21.140862 -69.4991559
H 4.0949378 20.1565639 -69.4957941
H 4.5709032 21.8214323 -69.7161739
N 1.9903342 20.6334323 -68.4586807
H 1.2955849 20.8980357 -67.7519289
H 2.1477213 19.6774308 -68.6872359
N 6.8385406 22.3850171 -63.9793491
H 6.2678414 21.5594362 -64.1188139
C 7.0762139 22.8882716 -62.6154744
H 6.7536171 23.9302454 -62.5989197
C 8.5529194 22.901266 -62.209056
O 8.8936518 23.5075295 -61.1960446
C 6.2302789 22.1490035 -61.5659052
H 6.3002834 22.7158685 -60.6375336
C 4.7406745 22.1370212 -61.9312552
H 4.5608851 21.5072413 -62.7992222
H 4.4104188 23.1524728 -62.155835
H 4.1599278 21.7513496 -61.0951958
C 6.7187884 20.740197 -61.2639461
H 6.946144 20.2365441 -62.1944734
H 5.9697426 20.1820863 -60.7022476
H 7.6299397 20.7966649 -60.6732389
N 9.4330305 22.2598839 -62.9727844
H 9.1190088 21.8290885 -63.831774
C 10.8586508 22.2157185 -62.6987607
H 11.0205601 22.3896924 -61.6385775
C 11.6587618 23.2613323 -63.4943908
O 11.250341 23.686733 -64.571033

C 11.3162717 20.8126336 -63.009183
H 11.2222436 20.673128 -64.0870845
H 10.6948542 20.0962774 -62.4706286
H 12.34619 20.6925253 -62.698318
N 12.8048385 23.6814376 -62.9622398
H 13.0708624 23.3246349 -62.0518115
C 13.7495271 24.5578181 -63.6509041
H 13.1778 25.2862108 -64.2238724
C 14.6719354 23.7794981 -64.6223379
O 14.975169 22.6149417 -64.3663321
C 14.5534374 25.3133525 -62.584314
H 15.1349368 24.6050227 -61.9900518
H 13.8784704 25.8645982 -61.9278724
H 15.2402197 26.0126728 -63.0612602
N 15.2562672 24.4346856 -65.6428965
C 16.3457623 23.8565942 -66.4329207
H 15.9645131 23.0084207 -66.9972777
C 17.5084646 23.3942187 -65.5437926
O 17.9666931 24.1420647 -64.6815619
C 16.7604415 24.9539896 -67.41675
H 17.5396302 25.5804047 -66.9808329
H 17.0916402 24.5356973 -68.368371
C 15.4800201 25.7739039 -67.5748403
H 14.8102338 25.2746548 -68.2775496
H 15.6876092 26.7949285 -67.8961341
C 14.8755827 25.7311526 -66.1737705
H 13.7917232 25.8368128 -66.2352599
H 15.3069372 26.5190763 -65.555338
N 17.9761557 22.1537945 -65.7184936
H 17.5578649 21.5750566 -66.4370437
C 18.9691881 21.5330127 -64.828799
H 19.4528712 20.7173447 -65.3653614
H 19.7268063 22.271958 -64.5676816
C 18.3945524 20.9579177 -63.5231969
O 19.1431088 20.4234825 -62.6929978
N 17.0763422 20.9995032 -63.325819
H 16.4871733 21.5126475 -63.9742723
C 16.4200515 20.282351 -62.2438022
H 16.8992307 20.5608404 -61.3042546
C 16.5430015 18.7673145 -62.4295642
O 16.5979498 18.2344241 -63.5433115
C 14.9511917 20.6876016 -62.1594518
H 14.4759675 20.4721613 -63.1160417
H 14.876111 21.7519627 -61.935609
H 14.4412873 20.1255796 -61.3775577
N 16.4551063 18.0541021 -61.3125826

H 16.2855776 18.5690471 -60.4511779
C 16.3207142 16.6066863 -61.2672556
H 16.667364 16.1436285 -62.1925943
C 14.8430527 16.4876811 -61.1714432
O 14.2703098 16.4918358 -60.0760996
C 17.1025975 16.0055349 -60.0967373
H 16.7267382 16.4254048 -59.1674263
H 16.9332383 14.927969 -60.0696969
C 18.6151246 16.2789271 -60.2035818
H 18.7934009 17.3000794 -60.5397025
H 19.0498173 15.6006874 -60.9384452
C 19.3309473 16.1098317 -58.8681021
O 19.8209138 17.063498 -58.2767724
N 19.4280626 14.9016131 -58.3528361
H 19.9160454 14.8206799 -57.47876
H 19.0269415 14.1074667 -58.8221429
N 14.2900884 16.7928196 -62.3621341
H 14.9082487 16.9578584 -63.1449686
C 12.8660165 16.8921841 -62.5786515
H 12.50313 17.8129391 -62.118628
C 12.3945187 16.8195691 -64.0583579
H 11.5182164 16.1806944 -64.1205188
C 11.927267 18.2080647 -64.4477732
H 12.7140434 18.9289267 -64.2293846
H 11.0102521 18.4103486 -63.8975376
H 11.6871596 18.2879955 -65.5028421
C 13.3973882 16.3601632 -65.1198995
H 13.1242213 16.743578 -66.1014841
H 14.3632553 16.7589904 -64.8585091
C 13.4802412 14.8541873 -65.2635705
H 12.5137973 14.4524802 -65.5470749
H 13.7913259 14.4259101 -64.3196536
H 14.1809111 14.6061911 -66.0551717
C 12.2832975 15.764129 -61.7971147
O 11.2494559 16.0014477 -61.1925983
N 12.9953431 14.6160479 -61.6922139
H 13.8487719 14.4634771 -62.2140222
C 12.4409562 13.5648977 -60.9104669
H 11.9661348 14.0429408 -60.056893
C 13.3950247 12.5287825 -60.3251963
O 14.3898634 12.1321749 -60.9337501
C 11.3641755 12.9163869 -61.7760491
H 11.69877 11.9263678 -62.0871113
C 10.1448767 12.7675339 -60.8818847
H 9.7797381 13.7482455 -60.5753726
H 10.389334 12.200937 -59.985975

H 9.3515675 12.2659959 -61.4236928
C 10.8142608 13.647437 -63.0167973
H 10.2651247 14.5505195 -62.7809551
H 10.1246692 12.9691349 -63.5158578
H 11.6027534 13.8396563 -63.7244018
N 12.992268 12.0624941 -59.1407394
H 12.2290494 12.5696896 -58.7005972
C 13.3517678 10.7991302 -58.4875107
H 13.8691189 10.1454328 -59.1901295
C 12.022295 10.1409558 -58.0722694
O 11.2124517 10.7801555 -57.3905926
C 14.2732964 11.0619765 -57.2714117
H 13.7556098 11.7467959 -56.5983282
C 14.5539489 9.7552096 -56.5040559
H 15.098085 9.0547942 -57.1393494
H 13.6255968 9.2720965 -56.1977078
H 15.1326487 9.9473315 -55.6028041
C 15.5967541 11.7351388 -57.7104154
H 16.1322448 11.0671286 -58.3834904
H 15.3704105 12.6510699 -58.2557262
C 16.5299331 12.1281646 -56.5575202
H 16.9353557 11.2398739 -56.0737671
H 15.9862613 12.7290053 -55.8275692
H 17.3598219 12.7148318 -56.9523203
N 11.7580335 8.9046503 -58.5018071
H 12.4971308 8.4049395 -59.0006782
C 10.4983895 8.1867901 -58.2471085
H 9.9675434 8.6814518 -57.4396159
C 10.7986262 6.7492106 -57.8252037
O 11.102295 5.9443846 -58.7003625
C 9.5576584 8.1569015 -59.488233
H 10.0657489 7.6118162 -60.2850846
C 9.2280089 9.5527545 -60.0531933
H 8.7489912 10.1650888 -59.2892799
H 10.1684049 10.018405 -60.3410172
C 8.2538598 7.3944316 -59.1507915
H 7.707404 7.913199 -58.3643917
H 8.4738782 6.3822778 -58.8197286
H 7.6197814 7.2880813 -60.0276344
C 8.3419087 9.5094014 -61.311817
H 7.3388524 9.1658198 -61.0701747
H 8.7868381 8.8466241 -62.055071
H 8.2449684 10.4990386 -61.7479271
N 10.3778145 6.3647875 -56.6173089
H 10.0867171 7.0875804 -55.969359
C 10.0771669 4.965996 -56.2829504

H 10.6161371 4.3342597 -56.978693
C 8.5735844 4.707958 -56.4325046
O 7.7547345 5.5273144 -56.0055968
C 10.6004787 4.5910992 -54.8968489
H 10.1061673 5.1945444 -54.1366728
H 11.6735419 4.7889482 -54.8607758
O 10.3828632 3.2140938 -54.6418497
H 9.4472056 3.0366627 -54.4338089
N 8.1607001 3.6309068 -57.1055167
H 8.8769937 2.9804449 -57.4273788
C 6.7371884 3.300688 -57.3620578
H 6.1596131 3.5083897 -56.4621738
C 6.5491433 1.8064488 -57.6597888
O 7.4787363 1.1227106 -58.0834072
C 6.1822375 4.201798 -58.4902398
H 6.9381585 4.3099942 -59.2694911
C 4.8723056 3.766805 -59.1432156
H 4.1171477 3.6139869 -58.3752925
H 5.0235349 2.8460121 -59.7068042
H 4.5357146 4.5394803 -59.8340945
O 5.8902408 5.4700017 -57.9372644
H 6.6083844 5.6589023 -57.3062582
N 5.3715908 1.2506011 -57.3572631
H 4.6553821 1.8125038 -56.8983762
C 5.0533527 -0.1448405 -57.6414244
H 5.9175855 -0.7539901 -57.3692877
C 4.7686066 -0.3651727 -59.1348527
O 3.9382459 0.3149866 -59.7426248
C 3.8790949 -0.6020956 -56.7719286
H 2.9852171 -0.0491227 -57.0590648
H 4.0992285 -0.4156114 -55.7198245
H 3.7121333 -1.6687299 -56.9224242
N 5.384449 -1.4132231 -59.6710028
H 5.9615485 -1.978817 -59.0585404
C 5.274073 -1.8707675 -61.0481607
H 4.52063 -1.2819514 -61.5709594
C 4.819635 -3.3368143 -61.111532
O 4.71484 -4.0327033 -60.0985856
C 6.6290324 -1.6468094 -61.7413333
H 6.7247861 -2.2962304 -62.6122492
H 7.4480343 -1.8571985 -61.0513851
S 6.7161264 0.0744788 -62.2988248
H 5.7073454 -0.0156779 -63.1767116
N 4.5804398 -3.8163232 -62.3306187
H 4.6770828 -3.1691214 -63.1064769
C 4.346574 -5.2294842 -62.6551505

H 4.459447 -5.8264837 -61.7510928
C 5.4032849 -5.7429896 -63.6329606
O 6.1897972 -4.9572822 -64.1649364
C 2.9059161 -5.4309908 -63.1662228
H 2.7672556 -6.4849372 -63.4143421
H 2.2061259 -5.1920836 -62.3647088
C 2.5629917 -4.5759187 -64.3962885
H 3.3538419 -4.6507815 -65.1381943
H 2.4774119 -3.5317996 -64.1082129
C 1.239358 -5.0199314 -65.0200982
H 0.4409992 -4.8935195 -64.2851521
H 1.2911458 -6.0804497 -65.2787751
N 0.9258565 -4.2126199 -66.212061
H 0.4081314 -3.3590145 -66.0351975
C 1.2259418 -4.5210722 -67.4631425
N 1.9688723 -5.5468659 -67.7776155
H 2.1152903 -5.8721481 -68.7219343
H 2.3570204 -6.1389378 -67.0480665
N 0.7801215 -3.779182 -68.4391839
H 0.1596895 -3.0162654 -68.2328983
H 0.9754474 -4.0537471 -69.3841574
N 5.3899017 -7.0384504 -63.929528
H 4.7383203 -7.6344439 -63.4363782
C 6.0942102 -7.5596355 -65.1036889
H 7.0977374 -7.1300138 -65.1006469
C 5.4232967 -7.0925274 -66.4226341
O 4.3354509 -6.5106139 -66.4170726
C 6.2677109 -9.0838745 -64.9594063
H 6.6936781 -9.2945219 -63.9792089
H 6.9785677 -9.4402086 -65.7029615
C 4.9912565 -9.885612 -65.1365704
O 4.2025192 -9.6370408 -66.0222303
N 4.7808317 -10.9165378 -64.3629691
H 3.901636 -11.390675 -64.4666077
H 5.4341878 -11.1196392 -63.6117621
N 6.087026 -7.301176 -67.5654737
H 6.97492 -7.7768733 -67.5279906
C 5.5686416 -6.8960675 -68.8824775
H 5.3268371 -5.8354456 -68.847767
C 4.2717675 -7.636028 -69.2757134
O 3.3960169 -7.0361793 -69.9063768
C 6.6966829 -7.1057223 -69.9118019
H 7.5465829 -6.4850685 -69.622889
H 7.0144693 -8.1489414 -69.8602459
C 6.3428838 -6.7968035 -71.3816833
H 5.5274226 -7.4439008 -71.7033055

C 5.9359276 -5.3387014 -71.5982807
H 6.7312629 -4.6712647 -71.263949
H 5.0185384 -5.1252485 -71.0539862
H 5.7487945 -5.1681951 -72.6580208
C 7.5553389 -7.0841982 -72.2684394
H 8.3866897 -6.4341584 -71.9947723
H 7.2949337 -6.9136733 -73.313153
H 7.8578121 -8.1251123 -72.1530906
N 4.1334798 -8.8979656 -68.8636108
H 4.8250579 -9.2749777 -68.2335885
C 3.0282568 -9.7990911 -69.2066128
H 2.994302 -9.8630138 -70.2915907
C 1.6602333 -9.3119172 -68.6927965
O 1.5945623 -8.516802 -67.7502102
C 3.3072741 -11.2092038 -68.6610331
H 2.5364612 -11.8929918 -69.0166306
H 3.2739869 -11.2045757 -67.5732222
O 4.5717364 -11.6737482 -69.1032695
H 4.7435367 -12.5260357 -68.6915725
N 0.5433211 -9.7783737 -69.2905212
C -0.7985269 -9.4881579 -68.7920022
H -0.974344 -8.4156383 -68.878059
C -0.9754439 -9.9212677 -67.3331253
O -0.527133 -10.9988821 -66.9390965
C -1.7659872 -10.2366409 -69.7171588
H -1.976375 -11.2318292 -69.3196156
H -2.693753 -9.6827823 -69.8650191
C 0.4554123 -10.5666366 -70.5124142
H 1.1583618 -10.2258453 -71.2721388
H 0.6242898 -11.6193873 -70.280253
C -0.973233 -10.3730318 -71.0152853
H -1.0393598 -9.4455678 -71.586019
H -1.3115491 -11.2200922 -71.6130074
N -1.6717958 -9.0991233 -66.547554
H -1.9774122 -8.2113845 -66.9104634
C -1.9426282 -9.3712957 -65.1363939
H -0.9869589 -9.4906116 -64.6280691
C -2.7599268 -10.6578647 -64.9656591
O -3.8400503 -10.7898855 -65.5414905
C -2.701309 -8.1901542 -64.5041285
H -2.7718945 -8.3380187 -63.4261289
H -3.7101012 -8.1606276 -64.9151862
O -2.0772418 -6.945159 -64.7763668
H -2.4140318 -6.2669389 -64.1668781
N -2.292544 -11.5662814 -64.1043462
H -1.3627723 -11.44079 -63.7365719

C -3.0207712 -12.7921241 -63.7457168
H -3.2244875 -13.323451 -64.6781217
C -4.3966937 -12.4684447 -63.1343671
O -4.6230048 -11.3825377 -62.579092
C -2.1096583 -13.72106 -62.9038127
H -1.910148 -14.6234283 -63.4834728
H -1.1418491 -13.2284864 -62.8004951
C -2.5148567 -14.1252411 -61.4840284
H -2.7946617 -13.2267165 -60.9280213
H -1.6416138 -14.553518 -60.9864657
C -3.6505368 -15.1492933 -61.4302847
O -4.2452488 -15.22482 -60.332582
O -4.0275463 -15.7087837 -62.4791995
N -5.3275999 -13.4242844 -63.2208885
H -5.0081732 -14.3620026 -63.4746751
C -6.7373853 -13.2558538 -62.8708644
H -7.1275333 -12.4809128 -63.5271843
C -6.946757 -12.7679238 -61.4410492
O -7.9117529 -12.0322895 -61.2615722
C -7.4812086 -14.5788163 -63.1367407
H -7.2969487 -14.8900304 -64.1668939
H -7.0568127 -15.3459934 -62.4846586
C -8.9995942 -14.5392484 -62.9048429
H -9.3885239 -15.5543231 -62.9959171
H -9.1947796 -14.2219061 -61.8805407
C -9.7553918 -13.6445641 -63.9063237
H -10.1326223 -14.2766743 -64.7127237
H -9.0952164 -12.9031723 -64.3565096
C -10.9343519 -12.935928 -63.2296966
H -11.4774207 -13.6713244 -62.6279936
H -11.6147183 -12.563259 -64.0023007
N -10.4659423 -11.8111498 -62.3804404
H -9.7675482 -12.1340372 -61.710333
H -11.2343898 -11.3862407 -61.8810379
H -10.0067443 -11.1094506 -62.9439105
N -6.0816248 -13.0886981 -60.4718269
H -5.3969747 -13.8110332 -60.6825751
C -6.0968226 -12.5397581 -59.1017552
H -7.1172031 -12.2357542 -58.8791371
C -5.2212816 -11.2922417 -58.8815129
O -5.4720267 -10.5585664 -57.9262851
C -5.7549487 -13.6167123 -58.0640669
H -6.3952429 -14.4869167 -58.2181221
H -5.9482444 -13.2182487 -57.0671658
O -4.3992676 -14.0098058 -58.1432093
H -4.343102 -14.5866227 -58.960205

N -4.2784466 -10.9636136 -59.7796263
H -4.191226 -11.5356199 -60.6116894
C -3.5645761 -9.675878 -59.7459851
H -3.2982635 -9.4612025 -58.710529
C -4.4764878 -8.5391038 -60.2170946
O -4.6424323 -7.5619692 -59.492832
C -2.2637066 -9.7208699 -60.5782299
H -1.5860132 -10.4576694 -60.1459091
H -2.5060962 -10.0411425 -61.5913691
C -1.54592 -8.348949 -60.6594275
H -2.2252017 -7.6004366 -61.0640231
C -1.049715 -7.869789 -59.2926931
H -0.3925492 -8.6162577 -58.8481829
H -1.895953 -7.6778135 -58.6345231
H -0.5004847 -6.9352805 -59.4146506
C -0.3461376 -8.4080241 -61.5969519
H 0.3865714 -9.1103831 -61.226032
H 0.1108929 -7.4212003 -61.6794127
H -0.6523785 -8.7230191 -62.5882788
N -5.1169134 -8.689943 -61.3849318
H -4.9457027 -9.5459942 -61.90868
C -6.0412299 -7.6860189 -61.9416269
H -5.4551489 -6.7857532 -62.1437637
C -7.1240799 -7.231914 -60.9375162
O -7.2297424 -6.0346225 -60.7273625
C -6.5934143 -8.1928127 -63.2907024
H -5.7661369 -8.2380254 -63.9984981
H -6.9541082 -9.2147834 -63.16253
C -7.7329938 -7.3712305 -63.9310273
H -7.8971692 -7.7500898 -64.9408023
H -8.6442331 -7.5659483 -63.3636415
C -7.5575872 -5.8442729 -64.0094301
H -7.4980538 -5.4352241 -63.0077227
H -8.4615509 -5.4163366 -64.4482493
C -6.3614685 -5.3390559 -64.8205827
H -6.662531 -5.2804452 -65.8695286
H -5.5195257 -6.0309988 -64.7300999
N -5.9571078 -4.0034871 -64.3263948
H -6.7679802 -3.395136 -64.17414
H -5.2630821 -3.5587778 -64.9038722
H -5.4837897 -4.0957187 -63.4295262
N -7.871645 -8.0931999 -60.2239082
C -8.873936 -7.6616363 -59.2573345
H -9.5234761 -6.9139405 -59.7159941
C -8.2649088 -7.0320422 -57.9992117
O -8.9337415 -6.198365 -57.3998078

C -9.7099499 -8.8997193 -58.9176722
H -10.0869565 -8.8805224 -57.8938592
H -10.5345133 -8.9914458 -59.6264198
C -7.9351771 -9.5147877 -60.3439147
H -8.4774898 -9.7379089 -61.262144
H -6.9490279 -9.9651637 -60.3563997
C -8.720291 -10.0246495 -59.1344312
H -9.2281338 -10.9685741 -59.3297095
H -8.0659944 -10.10908 -58.2666461
N -7.021786 -7.3606245 -57.6043131
H -6.4998668 -8.0322734 -58.1535313
C -6.3056253 -6.6353359 -56.5348897
H -6.9728801 -6.5111878 -55.6824392
C -5.9322881 -5.2270172 -57.0036012
O -6.1602659 -4.2700824 -56.2681912
C -5.0687729 -7.4390979 -56.0860549
H -5.3895788 -8.424192 -55.7418211
H -4.4305065 -7.5948128 -56.9556649
C -4.1992388 -6.7757217 -54.993123
H -3.2115868 -7.2341931 -55.0481208
H -4.0668317 -5.7140503 -55.2046993
C -4.7169933 -6.9550987 -53.5545555
H -4.8133604 -8.0239603 -53.351868
H -5.6999526 -6.4874112 -53.4551922
C -3.7314371 -6.3211879 -52.5553124
H -3.9467867 -5.2535484 -52.4822222
H -2.7197203 -6.4281609 -52.9491867
N -3.7880118 -6.9492504 -51.2117734
H -4.7008678 -6.8646728 -50.7950431
H -3.1294465 -6.5095671 -50.5629086
H -3.5314121 -7.9249485 -51.2652042
N -5.4101965 -5.0928218 -58.2204721
H -5.2513331 -5.9347831 -58.7634563
C -5.1139113 -3.8079675 -58.8626221
H -4.4172266 -3.2441876 -58.2414818
C -6.3827766 -2.9486339 -58.9977917
O -6.424878 -1.8461333 -58.4601641
C -4.4306352 -4.0925634 -60.2147685
H -3.4889227 -4.612916 -60.0251308
H -5.0681263 -4.750292 -60.8032861
C -4.1402352 -2.8329003 -61.0402102
H -3.5223312 -2.1630568 -60.4391233
H -5.0881717 -2.3339607 -61.2560169
C -3.4537445 -3.1310734 -62.3813936
O -3.6879035 -4.2298107 -62.9487496
O -2.7668995 -2.224337 -62.9038172

N -7.4664462 -3.5002571 -59.5499567
H -7.3734285 -4.4240226 -59.9575726
C -8.7116426 -2.7639545 -59.7964255
H -8.4353106 -1.8095062 -60.2480485
C -9.4638687 -2.4089424 -58.5074308
O -9.9528278 -1.288221 -58.3755535
C -9.6118555 -3.5466591 -60.7821039
H -9.0607949 -4.3693492 -61.2330581
H -10.4668038 -3.9705644 -60.2532929
C -10.1111674 -2.6392604 -61.9230753
H -10.7214885 -1.8416841 -61.4932013
H -10.7558622 -3.2309231 -62.5768496
C -8.9667387 -2.0313695 -62.7636765
O -8.2371866 -2.7994259 -63.4391515
O -8.8066446 -0.7847794 -62.7441526
N -9.47042 -3.2962327 -57.4999447
H -9.0417097 -4.20374 -57.6524768
C -10.1010431 -3.0170332 -56.1973756
H -11.0288266 -2.4764864 -56.3973745
C -9.2769901 -2.0761537 -55.3142795
O -9.8498703 -1.4157787 -54.4554675
C -10.4660358 -4.334596 -55.4944689
H -11.0058283 -4.9560219 -56.2112972
H -9.5566922 -4.8544857 -55.1890845
C -11.3704398 -4.1053174 -54.2696322
H -10.7558442 -3.807233 -53.4188719
H -12.0692675 -3.2953097 -54.4855386
C -12.2007088 -5.3376816 -53.8874421
H -12.8839548 -5.5640901 -54.7082912
H -12.7938671 -5.0865768 -53.0059178
C -11.3301939 -6.5636396 -53.5898349
H -10.6293076 -6.3072624 -52.7892185
H -10.7480368 -6.8020651 -54.4859351
N -12.1634946 -7.726805 -53.1936491
H -12.701381 -7.5095402 -52.3633894
H -11.5843428 -8.534113 -52.9993255
H -12.8073944 -7.9650457 -53.9371857
N -7.9716141 -1.9444161 -55.5661454
H -7.5758423 -2.5040646 -56.3110106
C -7.1488087 -0.8533673 -55.0040867
H -7.6222074 -0.5067725 -54.0856447
C -7.1064176 0.3871534 -55.9041949
O -6.4532318 1.363562 -55.5514579
C -5.7327836 -1.3132419 -54.6059369
H -5.1704231 -0.4493004 -54.2512706
C -5.7686133 -2.3392788 -53.4714731

H -6.3200593 -3.2274988 -53.7777969
H -6.2540956 -1.8980821 -52.6008782
H -4.7497511 -2.6174525 -53.2046511
O -5.0121386 -1.9015539 -55.6648057
H -5.5081724 -2.690785 -55.9458878
N -7.8166632 0.3654646 -57.0417343
H -8.3462446 -0.4726061 -57.2427193
C -7.8502309 1.3899778 -58.0923619
H -8.3461036 0.901136 -58.9292334
C -6.4594769 1.7892235 -58.633805
O -6.3001095 2.8344891 -59.2640218
C -8.7526115 2.5574542 -57.6322694
H -9.6187161 2.1305136 -57.1235023
H -8.1999693 3.1478449 -56.8997247
C -9.2803135 3.5032468 -58.7320415
H -8.4579137 4.0436594 -59.1952806
C -10.0659324 2.771923 -59.8264709
H -10.8596408 2.1741863 -59.3761475
H -9.4021933 2.1159166 -60.3858728
H -10.5024011 3.4948423 -60.5147045
C -10.2132689 4.5376623 -58.0986059
H -11.0746174 4.0426197 -57.649767
H -10.5543428 5.2422816 -58.85601
H -9.6730952 5.0878871 -57.3275553
N -5.4426723 0.9434196 -58.4481881
H -5.6678236 0.0355238 -58.0548346
C -4.0798935 1.185912 -58.9357536
H -3.8150141 2.2211407 -58.7113507
C -4.0051982 1.032849 -60.4518383
O -4.7678273 0.27695 -61.0434649
C -3.0949649 0.2568287 -58.2130025
H -3.5138127 -0.7490093 -58.1422002
H -2.1678002 0.2159683 -58.784929
C -2.795052 0.8141 -56.8097441
H -2.3119699 1.7866437 -56.9208895
H -3.7301621 0.9656837 -56.2690902
C -1.8893985 -0.0773955 -55.9502131
H -2.4545609 -0.9480272 -55.6154601
H -1.5950424 0.5033683 -55.0736083
C -0.6439782 -0.5463608 -56.7108241
H -0.3018319 0.268945 -57.3493463
H -0.9215663 -1.3830817 -57.3566641
N 0.4467047 -0.9339892 -55.7916971
H 0.7465728 -0.112789 -55.2498367
H 1.2748543 -1.2105249 -56.2979961
H 0.1611309 -1.6486113 -55.1440047

N -3.0487572 1.7262357 -61.0774219
H -2.4915023 2.3509615 -60.5015827
C -2.7384924 1.6213578 -62.5167704
H -3.2764944 0.767828 -62.9341887
C -1.2710996 1.2723612 -62.7035448
O -0.4066791 2.1504201 -62.6343866
C -3.1835379 2.9095297 -63.2545982
H -3.4195988 3.6894009 -62.5300726
H -2.3764105 3.2816492 -63.8842477
C -4.4157079 2.6815394 -64.1627795
H -4.7723658 3.6569256 -64.4963477
H -4.1117891 2.1108096 -65.0407656
C -5.5713926 1.9356428 -63.4710507
H -5.670715 2.3288555 -62.460506
H -5.3424209 0.8725846 -63.4125411
C -6.9189723 2.0674559 -64.1837241
H -6.9312356 1.427326 -65.0694601
H -7.0421595 3.108668 -64.4916204
N -8.0097774 1.7033435 -63.2510456
H -7.9605382 0.719054 -62.960874
H -8.919042 1.7965361 -63.6756161
H -7.9574248 2.2846703 -62.4272009
N -0.9851324 -0.0152724 -62.8685472
H -1.7507671 -0.6921228 -62.9343551
C 0.3622951 -0.5630426 -62.7563147
H 0.9805693 0.166144 -62.2307181
C 0.9623679 -0.7635467 -64.1569443
O 0.4536773 -1.5034089 -65.0035394
C 0.3662204 -1.8384641 -61.8795023
H -0.0630003 -2.6610733 -62.4548948
C 1.819163 -2.1735927 -61.5191769
H 2.2062048 -1.4454116 -60.8056847
H 2.4410637 -2.152216 -62.4072739
H 1.8767011 -3.1699076 -61.08193
C -0.4305112 -1.6738873 -60.5577608
H 0.0351023 -0.9039866 -59.9410057
H -1.442414 -1.3440835 -60.7786126
C -0.5557201 -2.9669112 -59.7395202
H 0.4082644 -3.2467547 -59.3150915
H -0.9233073 -3.7726035 -60.3764866
H -1.265722 -2.8133331 -58.927048
N 2.0820627 -0.0851478 -64.4029156
H 2.4655832 0.475942 -63.6575331
C 2.9068083 -0.2770199 -65.5962123
H 2.3018658 -0.6952979 -66.4020926
C 4.0396154 -1.2643317 -65.292497

O 4.4307098 -1.4269569 -64.1332412
C 3.4495725 1.0814523 -66.061202
H 4.0958151 0.9525892 -66.9322466
H 4.0432311 1.5292122 -65.261646
S 2.0709612 2.1846743 -66.4876026
H 1.3003227 1.8921115 -65.4342484
N 4.6448883 -1.8495044 -66.3268338
H 4.3466771 -1.6176306 -67.261478
C 5.9932657 -2.3856146 -66.1595621
H 6.0491473 -2.8706147 -65.1886549
C 7.0088041 -1.2347704 -66.1249314
O 6.737833 -0.132612 -66.6086652
C 6.3073177 -3.4524623 -67.2163512
H 5.4957053 -4.1766962 -67.2517385
H 7.2191217 -3.9815768 -66.9375472
C 6.5230252 -2.8364934 -68.5773457
O 7.5678347 -2.280934 -68.8635157
N 5.5086591 -2.8222407 -69.403895
H 5.6807276 -2.3748843 -70.3032807
H 4.6772054 -3.3460722 -69.2191437
N 8.1801732 -1.5131329 -65.5618081
H 8.3470506 -2.4508453 -65.2320084
C 9.2452561 -0.5331003 -65.3748175
H 8.8589516 0.286887 -64.7647094
C 9.6957767 0.098359 -66.7072817
O 9.7789872 1.3222783 -66.8014925
C 10.3873618 -1.2029495 -64.5780293
H 11.2574185 -0.549599 -64.5671677
H 10.0590078 -1.2929866 -63.5414857
C 10.8177317 -2.5865022 -65.0490904
C 11.9181375 -2.7365552 -65.9169673
H 12.4819757 -1.8755661 -66.2390297
C 12.3053206 -4.0136478 -66.363435
H 13.1452796 -4.1187607 -67.0335377
C 11.597087 -5.1547648 -65.9319127
O 11.9592972 -6.3881262 -66.3699191
H 12.7274721 -6.3595775 -66.9421564
C 10.5129368 -5.0118722 -65.040923
H 9.9908157 -5.8930459 -64.6965091
C 10.1282528 -3.7322316 -64.5973171
H 9.308215 -3.6379075 -63.8983316
N 9.914116 -0.6953223 -67.7660937
H 9.7185 -1.6813804 -67.6780474
C 10.4258322 -0.1969978 -69.0526211
H 11.3531195 0.3334907 -68.8684677
C 9.4744436 0.8147866 -69.7068052

O 9.9050969 1.903903 -70.0918449
C 10.7555701 -1.3866897 -69.9704476
H 11.4799712 -2.0264374 -69.4643361
H 9.8468928 -1.968167 -70.1290859
C 11.319076 -0.9912917 -71.3499645
H 10.5886703 -0.3825456 -71.8825983
C 12.633967 -0.2142643 -71.2488822
H 13.3625122 -0.779567 -70.6682336
H 12.462991 0.7532524 -70.7772734
H 13.0319279 -0.0350601 -72.2476385
C 11.5735498 -2.2547509 -72.1719436
H 12.3160219 -2.8816896 -71.6783682
H 11.9344282 -1.9816748 -73.1636924
H 10.6437644 -2.8131246 -72.2827093
N 8.1721243 0.5219023 -69.7300654
H 7.8781855 -0.3931025 -69.4008782
C 7.1627271 1.4694778 -70.216607
H 7.465206 1.8258281 -71.2019721
C 7.0485684 2.7218684 -69.344085
O 6.7977963 3.7974389 -69.8845881
C 5.8037654 0.7721156 -70.3591403
H 5.0142317 1.5249066 -70.3314356
H 5.6424419 0.0910384 -69.5229777
C 5.6722935 0.0300481 -71.6647418
N 5.9532087 -1.3209948 -71.8725283
C 5.7499517 -1.545272 -73.178947
H 5.897551 -2.4993019 -73.6695688
N 5.3500598 -0.4176791 -73.790252
H 5.1471925 -0.3273254 -74.7758818
C 5.2980715 0.5887433 -72.851667
H 5.0341347 1.6258243 -73.0140616
N 7.3164396 2.6332608 -68.0360767
H 7.5599259 1.7314697 -67.6420576
C 7.2614763 3.7968527 -67.1509479
H 6.3620564 4.3393293 -67.4291629
C 8.4261241 4.7738874 -67.3880553
O 8.1695259 5.9696373 -67.5199989
C 7.0948289 3.3363918 -65.6891448
H 6.3288467 2.5601209 -65.6683109
H 8.0271337 2.8928056 -65.3390609
C 6.6681347 4.4549309 -64.7117129
H 7.5189438 5.109165 -64.5258907
C 5.4997377 5.3156355 -65.2074584
H 4.6757884 4.679795 -65.5306189
H 5.8301918 5.9439793 -66.0348046
H 5.1677863 5.9791257 -64.4099764

C 6.2145039 3.8387536 -63.3851094
H 5.3016056 3.2618588 -63.5260214
H 6.0445249 4.6237662 -62.6487135
H 6.9882306 3.1722436 -63.0041928
N 9.6480168 4.2850584 -67.6533181
H 9.7997559 3.2846604 -67.5630426
C 10.7262591 5.1507473 -68.1801541
H 10.8557976 5.9933419 -67.499146
C 10.3952471 5.6900104 -69.5631661
O 10.7418516 6.824877 -69.8713061
C 12.0605472 4.4240518 -68.3016877
H 12.1166467 3.8610945 -69.2350101
H 12.121121 3.7293563 -67.4803064
O 13.1491082 5.3247477 -68.1948499
H 13.269569 5.8510611 -69.0056855
N 9.6793356 4.9112594 -70.3797769
H 9.4739327 3.9631262 -70.084374
C 9.1529553 5.3651188 -71.6612905
H 9.9800387 5.6169965 -72.3253587
H 8.5721268 4.5597261 -72.1096179
C 8.2436502 6.5889815 -71.5206075
O 8.4964877 7.5881455 -72.1829673
N 7.271716 6.5802667 -70.6017955
H 7.1008963 5.7179054 -70.0934582
C 6.3753098 7.7248641 -70.3564864
H 5.913338 8.0175862 -71.2986071
C 7.1064366 8.967594 -69.8279953
O 6.7901523 10.0960769 -70.2162708
C 5.2706065 7.3106618 -69.3736671
H 5.7289851 6.9026424 -68.4718638
H 4.7203521 8.2063148 -69.0832355
C 4.2527546 6.3259864 -69.9274463
C 3.9569052 5.1377987 -69.2312677
H 4.470828 4.90886 -68.3126257
C 2.9868958 4.2463839 -69.7247382
H 2.7656118 3.3367646 -69.1875547
C 2.2932897 4.5473782 -70.9100298
H 1.5351606 3.8746796 -71.2849157
C 2.5749518 5.7375725 -71.6014384
H 2.0297721 5.995317 -72.5016579
C 3.5527959 6.6201892 -71.1146242
H 3.7383022 7.5375435 -71.6559592
N 8.1333138 8.7500029 -69.0098282
H 8.2811018 7.8069615 -68.6678179
C 9.07562 9.7716512 -68.5730746
H 8.5547353 10.5922679 -68.0912188

C 9.8605926 10.3853852 -69.7513712
O 9.7566054 11.5847949 -70.0065046
C 9.9597189 9.1076336 -67.5219712
H 10.8339721 9.7058895 -67.3759088
H 10.2895408 8.1279684 -67.8552867
S 9.0625811 8.9471298 -65.9503501
H 9.8351265 9.8474364 -65.3019252
N 10.4805271 9.5504548 -70.5898632
H 10.5030738 8.5688311 -70.3340113
C 11.2121939 9.9579073 -71.8004392
H 12.0155281 10.6371849 -71.5108146
C 10.3189662 10.6842638 -72.8220458
O 10.6706195 11.7496569 -73.3218699
C 11.8287769 8.7012708 -72.4308687
H 11.0787146 8.1829872 -73.0301747
H 12.1581597 8.0264929 -71.641054
O 12.9535704 9.016732 -73.2287619
H 13.2151123 8.2260534 -73.7115009
N 9.0917743 10.1931924 -73.0341901
H 8.8462209 9.3288767 -72.56349
C 8.0758771 10.7997598 -73.908273
H 8.5230395 10.9933282 -74.8827677
C 7.5601152 12.1572996 -73.4059811
O 6.9709603 12.9057875 -74.1813275
C 6.8980951 9.8177845 -74.0665242
H 6.5963806 9.4797408 -73.0746319
H 6.0469815 10.3468555 -74.4990079
C 7.2010356 8.596359 -74.9559661
H 8.1359891 8.1311667 -74.6495739
C 6.078985 7.5667695 -74.8172612
H 5.1265064 8.0041828 -75.1217947
H 6.002105 7.244648 -73.7790986
H 6.2918725 6.6974123 -75.4373065
C 7.3195826 8.9722824 -76.4354788
H 6.4050387 9.4644876 -76.7684964
H 7.4802548 8.0744735 -77.031481
H 8.1643764 9.6422728 -76.586479
N 7.8083199 12.5056056 -72.1428918
H 8.3267403 11.8644839 -71.5540116
C 7.4700463 13.8201725 -71.5844624
H 6.6249218 14.2248713 -72.140381
C 8.5965015 14.8537852 -71.7283394
O 8.4412966 15.9715581 -71.2569983
C 7.0096966 13.6702722 -70.1392581
H 6.7278152 14.646009 -69.7435907
H 7.8241777 13.2648047 -69.5378654

O 5.8818799 12.8084382 -70.0899695
H 6.1934362 11.8939971 -70.2199382
N 9.6527789 14.5194905 -72.4726668
H 9.6609405 13.583641 -72.8564542
C 10.8450282 15.3307806 -72.7823003
H 11.3215018 14.7681748 -73.5857787
C 11.9223128 15.3449434 -71.6844862
O 12.7208264 16.2703293 -71.5460031
C 10.4885248 16.6983789 -73.4123986
H 10.3052822 17.4216129 -72.6154718
H 9.5630553 16.5886969 -73.9810148
C 11.5483248 17.2610639 -74.3780375
O 12.3958398 16.4764423 -74.8659835
O 11.4371665 18.4657644 -74.7049311
N 11.9834183 14.26542 -70.8998863
H 11.310854 13.5258562 -71.0438426
C 13.0028759 14.0912496 -69.8714545
H 13.306885 15.0728525 -69.5156226
C 14.2829326 13.4339092 -70.3807713
O 14.2769208 12.3465484 -70.963321
C 12.4106606 13.3349922 -68.6923727
H 12.0504773 12.3665639 -69.0383334
H 13.215576 13.1470367 -67.9823596
C 11.3089208 14.0511588 -67.9372688
C 10.4143586 13.2895275 -67.1652494
H 10.4207411 12.2129036 -67.2452246
C 9.622269 13.9020251 -66.183757
H 9.0420531 13.2912381 -65.5123696
C 9.6896914 15.2939169 -66.0117228
O 9.0688287 15.8810816 -64.9659394
H 8.6517885 15.2197967 -64.4135997
C 10.476267 16.0688853 -66.8777947
H 10.5008894 17.1334103 -66.7732363
C 11.2869235 15.4568867 -67.8389113
H 11.9309688 16.084664 -68.4366982
N 15.413457 14.0666489 -70.0648519
H 15.3470006 14.8954175 -69.4865486
C 16.7466287 13.6072225 -70.4479379
H 16.6439033 13.0148765 -71.3578561
C 17.3845581 12.7089696 -69.3861672
O 17.0498046 12.785335 -68.2019845
C 17.6340751 14.8021464 -70.8021027
H 17.0888067 15.4656922 -71.4759625
H 18.5250258 14.4458703 -71.3186039
O 18.0387931 15.5263655 -69.6592734
H 17.3011331 15.5682376 -69.0191956

N 18.3085085 11.8526167 -69.839663
H 18.540374 11.8840546 -70.8182419
C 19.0346307 10.8940314 -69.0057114
H 19.4675616 10.1472293 -69.6713046
C 18.1121144 10.1118048 -68.0476025
O 18.3664568 10.0593789 -66.8463913
C 20.2060694 11.6112569 -68.3167241
H 19.8233584 12.3411857 -67.6012615
H 20.822286 12.1241624 -69.055329
H 20.8190484 10.8836954 -67.7848943
N 17.0082147 9.5547067 -68.5680509
H 16.8205954 9.6340163 -69.5538031
C 16.1258953 8.7216485 -67.7571872
H 16.0455573 9.2076642 -66.7871337
C 16.7726785 7.3640399 -67.4612841
O 17.037384 6.5613184 -68.3551552
C 14.6792575 8.6592675 -68.293561
H 14.2122908 9.6269136 -68.1101076
H 14.1203773 7.931987 -67.704949
C 14.479784 8.3334479 -69.7606206
O 14.1690558 7.2130685 -70.1375173
N 14.5042034 9.3331716 -70.6191546
H 14.1908225 9.1433714 -71.5603483
H 14.5384129 10.2922772 -70.2963576
N 17.0392665 7.1424113 -66.177116
H 16.830673 7.8928146 -65.5251381
C 17.5157217 5.8978433 -65.5954028
H 17.9177789 5.2616605 -66.384763
C 16.3357946 5.207524 -64.9288189
O 15.6159122 5.8225069 -64.1466295
C 18.6212937 6.1737034 -64.5672921
H 18.1711266 6.6156535 -63.6769974
H 19.0702786 5.2249355 -64.2716113
C 19.7093446 7.0913046 -65.0380035
C 19.7931824 8.4044157 -64.7311366
H 19.0720161 8.9404149 -64.124824
N 20.8504143 8.9783652 -65.4059943
H 20.9953366 9.9773354 -65.4322483
C 21.4912532 8.0630289 -66.2111008
C 22.5607112 8.1678796 -67.1126483
H 23.0547657 9.1163029 -67.2628505
C 22.976687 7.0234162 -67.8144749
H 23.7994497 7.0845023 -68.5131482
C 22.3164877 5.7977862 -67.6094385
H 22.6337933 4.9207785 -68.1562422
C 21.2368826 5.7069224 -66.707767

H 20.7248034 4.766601 -66.5741431
C 20.7952953 6.8365602 -65.9837824
N 16.1848563 3.912817 -65.176291
H 16.821799 3.469261 -65.8205527
C 15.0796393 3.1104436 -64.6775838
H 14.54308 3.6333506 -63.8833365
C 15.6247004 1.7985474 -64.1325112
O 16.0577958 0.9402323 -64.9020713
C 14.131694 2.8887033 -65.8487103
H 13.64995 3.8395378 -66.0623428
H 14.7289034 2.6128478 -66.7202805
C 13.0901348 1.8002141 -65.5654381
H 13.4487714 1.0489044 -64.8679001
C 11.8698551 2.4330043 -64.9143906
H 11.3898081 3.1481945 -65.575671
H 12.1699034 2.9421886 -63.9979327
H 11.1508884 1.6662489 -64.6483251
C 12.8433832 1.0456084 -66.8611406
H 12.3756177 1.6885177 -67.5980209
H 12.2143535 0.1981272 -66.6495824
H 13.7858171 0.6635186 -67.2549475
N 15.5022455 1.6123315 -62.8227831
H 15.0176951 2.3212374 -62.2749974
C 15.9819101 0.4156113 -62.1354336
H 16.5767406 -0.1688088 -62.8309248
C 14.7948239 -0.412656 -61.638592
O 14.0877462 0.0314062 -60.7298387
C 16.9228566 0.7713672 -60.9737509
H 16.3487296 1.1324943 -60.119959
C 17.7625409 -0.435787 -60.5611816
H 18.3692173 -0.7740933 -61.4018301
H 17.1180753 -1.2500821 -60.2345302
H 18.4224701 -0.1512396 -59.7425426
O 17.83844 1.7718527 -61.3585353
H 17.3314862 2.5714806 -61.5236629
N 14.5757734 -1.6381667 -62.1522123
C 13.7832369 -2.6243789 -61.4326093
H 12.8078798 -2.2156713 -61.1651937
C 14.5459745 -3.0172483 -60.1610187
O 15.6270337 -3.5983735 -60.2304277
C 13.5987399 -3.7952014 -62.405327
H 13.5382697 -4.7567764 -61.8952256
H 12.6985773 -3.6284725 -62.9974839
C 15.1494966 -2.2202696 -63.3610773
H 14.6833669 -1.7644417 -64.236083
H 16.2312348 -2.0961457 -63.4033807

C 14.823558 -3.7137458 -63.3158808
H 14.6132543 -4.1093829 -64.3096971
H 15.6551597 -4.2543635 -62.8618106
N 13.9662979 -2.746838 -58.9908767
H 13.0661721 -2.2754632 -59.0090904
C 14.5182136 -3.1102436 -57.6789229
H 15.4998097 -3.5591397 -57.8229977
C 13.7014467 -4.2044761 -56.9428997
O 13.6238671 -4.1604091 -55.7146881
C 14.714941 -1.81596 -56.8514598
H 13.7756341 -1.2643591 -56.8431172
H 14.9534945 -2.0737327 -55.819355
C 15.8229594 -0.873091 -57.3541501
H 15.6404761 -0.6146515 -58.3959849
C 15.8120489 0.4127376 -56.5303236
H 16.028931 0.1980959 -55.4826737
H 14.8309305 0.8833728 -56.593059
H 16.5554212 1.1109018 -56.9145674
C 17.2136708 -1.5018124 -57.2217831
H 17.3755269 -1.8389114 -56.1975999
H 17.9750578 -0.7631874 -57.470298
H 17.3151205 -2.3433035 -57.9050428
N 13.0948036 -5.214763 -57.6151661
C 12.2053006 -6.1721577 -56.9514439
H 11.3888186 -5.644905 -56.4617025
C 12.9249789 -7.0386965 -55.9075064
O 12.3195788 -7.4085346 -54.9111168
C 11.6162382 -7.0363963 -58.0682371
H 11.3764525 -8.0460584 -57.7312578
H 10.7270492 -6.5549761 -58.4702904
C 13.2765494 -5.6211828 -59.0043124
H 12.7053484 -4.9577645 -59.6524598
H 14.33053 -5.6213029 -59.2835882
C 12.7120005 -7.0340446 -59.1282169
H 12.3119336 -7.2235629 -60.1252091
H 13.4796364 -7.7662537 -58.8743209
N 14.2193922 -7.3309605 -56.0875591
H 14.6758111 -6.9951267 -56.9187628
C 15.0103271 -8.0990672 -55.112049
H 14.434692 -8.9910839 -54.8655987
C 15.2070223 -7.332927 -53.7964256
O 15.0766997 -7.9116657 -52.717904
C 16.3497863 -8.552893 -55.7413196
H 16.1067111 -9.1238207 -56.6393262
C 17.2457079 -7.3784555 -56.1790683
H 17.6104389 -6.833258 -55.3064419

H 16.7096291 -6.695959 -56.8363059
H 18.1109972 -7.7622999 -56.7213065
C 17.1758962 -9.4774166 -54.8192359
H 18.0864798 -9.7670527 -55.3455807
H 17.4716894 -8.935527 -53.9193684
C 16.4479887 -10.7630839 -54.4053729
H 16.0929498 -11.2919093 -55.2902479
H 15.605773 -10.5327737 -53.7532482
H 17.1389073 -11.4070984 -53.8607548
N 15.3935547 -6.0141343 -53.87863
H 15.4044782 -5.57695 -54.785056
C 15.4989416 -5.1573755 -52.7005961
H 16.1260958 -5.6549509 -51.9609007
C 14.1275042 -4.979588 -52.0493859
O 14.0041547 -5.2410058 -50.8625342
C 16.1529791 -3.8128398 -53.0612849
H 15.5453294 -3.3096024 -53.8136461
H 16.1749842 -3.1849001 -52.1697456
C 17.5888162 -3.9565456 -53.5969701
H 17.9604463 -2.9636573 -53.8583359
H 17.5825816 -4.5500643 -54.513358
C 18.5239075 -4.5941443 -52.560969
O 18.5957494 -5.8428049 -52.4662396
O 19.14147 -3.8633975 -51.7551244
N 13.0718701 -4.7526557 -52.834066
H 13.246795 -4.5814834 -53.8192455
C 11.6674641 -4.7573474 -52.3915481
H 11.5110988 -3.9424476 -51.6838844
C 11.2638044 -6.055375 -51.6705936
O 10.7000723 -5.9916155 -50.5815018
C 10.8225721 -4.4658085 -53.6373247
H 11.2703018 -4.9725609 -54.4847155
H 10.8980451 -3.3996945 -53.8162933
C 9.3400869 -4.8422281 -53.6032018
O 8.5960027 -4.4336681 -52.6834481
O 8.8625046 -5.4577929 -54.5871172
N 11.6917214 -7.2238873 -52.1577427
H 12.1378338 -7.2256039 -53.0701622
C 11.4803921 -8.5084706 -51.4804795
H 10.4192842 -8.5902366 -51.2439924
C 12.2581264 -8.616735 -50.1576347
O 11.7173718 -9.1125472 -49.1729519
C 11.8590517 -9.6523727 -52.4351888
H 12.9441046 -9.6885066 -52.5437256
H 11.4310558 -9.4628766 -53.4176041
C 11.3583046 -11.0199242 -51.9562242

H 11.7482812 -11.2164564 -50.9570363
H 11.7634607 -11.7769659 -52.6281464
S 9.5513028 -11.2041661 -51.9296248
C 9.4280423 -12.9497713 -51.463499
H 8.3802362 -13.2482151 -51.4521366
H 9.8585708 -13.0948903 -50.4720088
H 9.9653893 -13.5614913 -52.1881289
N 13.4992124 -8.1155487 -50.1013757
H 13.8751445 -7.7085546 -50.9531837
C 14.3462607 -8.0992723 -48.8917458
H 14.3261654 -9.0880359 -48.4302966
C 13.8414349 -7.109273 -47.8321094
O 13.8696885 -7.4088854 -46.6438106
C 15.7803139 -7.7771225 -49.3386629
H 16.0728206 -8.4953921 -50.1072518
H 15.78842 -6.7853287 -49.7877521
C 16.8146607 -7.8333564 -48.2019743
H 16.5459291 -7.1366515 -47.4067893
H 16.8280532 -8.8437172 -47.7908348
C 18.2187971 -7.4873246 -48.7193819
H 18.4184365 -8.0829343 -49.6128579
H 18.9536731 -7.7499724 -47.9570584
C 18.3487833 -5.992413 -49.0458139
H 18.4319706 -5.4217815 -48.1165061
H 17.4451803 -5.6561907 -49.5619324
N 19.5031294 -5.7293883 -49.9309239
H 20.3954323 -5.9966135 -49.5549232
H 19.5174901 -4.749167 -50.2346865
H 19.3464276 -6.1609146 -50.848426
N 13.3322727 -5.9630881 -48.2710839
H 13.3916351 -5.7966786 -49.2700395
C 12.6752334 -4.9259791 -47.4652083
H 13.3066394 -4.639705 -46.6204001
C 11.3514425 -5.4659331 -46.907204
O 11.0801258 -5.3692947 -45.708615
C 12.463281 -3.6936637 -48.3766892
H 12.0638674 -4.0566426 -49.3241162
C 11.3939495 -2.735481 -47.8251092
H 11.5699158 -2.5482408 -46.7698325
H 10.4027027 -3.1721763 -47.9439616
H 11.4167651 -1.795219 -48.3641339
C 13.8090055 -3.0011842 -48.7056933
H 14.1943818 -2.5034339 -47.8276267
H 14.5553025 -3.7476582 -48.9678682
C 13.7415405 -1.9940272 -49.8643963
H 13.1388901 -1.128049 -49.5957665

H 13.3172396 -2.4665957 -50.7493734
H 14.7489847 -1.6485579 -50.0979748
N 10.5569209 -6.1251239 -47.7575478
H 10.8058184 -6.1502801 -48.7417588
C 9.3406062 -6.8155937 -47.3499945
H 8.687436 -6.0980664 -46.8515223
C 9.6451199 -7.9320667 -46.3426887
O 8.9789309 -7.9857565 -45.3181334
C 8.6090851 -7.3439192 -48.5900398
H 9.2347282 -8.0646011 -49.1166765
H 8.3743305 -6.5167723 -49.2610325
H 7.6825766 -7.8315087 -48.286617
N 10.6967849 -8.7327052 -46.5459848
H 11.1877227 -8.6812436 -47.4322827
C 11.1410652 -9.7413254 -45.5766499
H 10.3095113 -10.4188421 -45.3765601
C 11.5417736 -9.1181235 -44.2316211
O 11.0027769 -9.5243077 -43.2065835
C 12.2879134 -10.5821685 -46.1574739
H 13.1383181 -9.9410307 -46.3900585
H 11.9539012 -11.054893 -47.0827182
C 12.7241101 -11.6616207 -45.1622198
O 13.5224159 -11.3337599 -44.252894
O 12.2018212 -12.793503 -45.265959
N 12.3829782 -8.0769071 -44.2118859
H 12.8033958 -7.7736289 -45.0842588
C 12.8418208 -7.4756884 -42.9545116
H 13.20788 -8.2795145 -42.315361
C 11.7105569 -6.7843906 -42.179033
O 11.5105007 -7.0762136 -40.995856
C 14.0107534 -6.5158331 -43.2086705
H 14.8199205 -7.0620265 -43.6953675
H 13.6867322 -5.7238326 -43.8863098
C 14.5352889 -5.889822 -41.9505213
C 15.3513898 -6.4920806 -41.0560262
H 15.7532765 -7.4912347 -41.1718495
N 15.5335855 -5.6654725 -39.9629168
H 16.0897329 -5.9158626 -39.160673
C 14.8148192 -4.49376 -40.0872147
C 14.6321954 -3.3866221 -39.242576
H 15.1032791 -3.3500841 -38.2735638
C 13.819259 -2.3236695 -39.676404
H 13.6551516 -1.4597462 -39.0434416
C 13.2231146 -2.3758491 -40.9490175
H 12.61592 -1.5480294 -41.2963996
C 13.413713 -3.4925454 -41.785488

H 12.9690569 -3.4997165 -42.7718436
C 14.1985453 -4.5894945 -41.3712467
N 10.9043925 -5.9527161 -42.8492866
H 11.1134384 -5.7630804 -43.8240366
C 9.7438538 -5.2921988 -42.2212211
H 10.0844229 -4.773692 -41.3238945
C 8.6828352 -6.2978319 -41.7797415
O 8.1475363 -6.1684389 -40.6765001
C 9.0796136 -4.2487583 -43.1304959
H 8.177974 -3.8895772 -42.6371761
C 9.9720763 -3.0467225 -43.4104666
H 10.8669468 -3.3593763 -43.9445606
H 10.2661708 -2.5763767 -42.4714854
H 9.4314004 -2.3171273 -44.0117579
O 8.7181591 -4.8066588 -44.3726664
H 9.5496823 -4.9479732 -44.8577988
N 8.4358032 -7.3581704 -42.5633335
H 8.8963046 -7.4314515 -43.466883
C 7.5723311 -8.4495715 -42.1268535
H 6.6350766 -8.0155664 -41.7795424
C 8.1700975 -9.1517994 -40.9247922
O 7.4758181 -9.174459 -39.9330543
C 7.2448461 -9.4620492 -43.2330206
H 8.1639771 -9.7685181 -43.7320877
H 6.8107539 -10.3548548 -42.779457
C 6.239774 -8.9211213 -44.2597927
H 6.2060887 -9.6067299 -45.1067549
H 6.5649357 -7.9487869 -44.6272582
C 4.8301142 -8.7907822 -43.6962677
O 4.0185217 -9.6936336 -43.7800733
N 4.4373901 -7.6329512 -43.2092787
H 3.4549186 -7.5555601 -42.9669857
H 5.0762198 -6.8618807 -43.140931
N 9.4273277 -9.6006942 -40.9284614
H 9.9686587 -9.4839231 -41.780828
C 10.0214635 -10.412134 -39.8567427
H 9.3084547 -11.1990181 -39.609992
C 10.253828 -9.6601678 -38.5291229
O 10.2250454 -10.2893742 -37.4666527
C 11.2944858 -11.0964365 -40.3945858
H 11.8618845 -11.5134649 -39.5643313
H 11.9244235 -10.3674132 -40.904203
C 10.9694015 -12.2521054 -41.3360884
O 10.0165345 -12.9890257 -41.1245311
N 11.7728694 -12.4937022 -42.343769
H 11.4995019 -13.1733068 -43.0362986

H 12.499172 -11.8467642 -42.6449554
N 10.2929531 -8.3240194 -38.537167
H 10.3624872 -7.8437779 -39.4304161
C 10.2075646 -7.5261957 -37.311539
H 10.9370343 -7.9083139 -36.5955343
C 8.8173012 -7.6078404 -36.6366541
O 8.7224682 -7.6976754 -35.4119872
C 10.5911494 -6.0827475 -37.6571958
H 9.8848154 -5.6694188 -38.3793169
H 11.5923603 -6.0574983 -38.0909011
H 10.5793001 -5.471428 -36.7549126
N 7.7281362 -7.648853 -37.4093658
H 7.8464977 -7.6620426 -38.4175533
C 6.3615845 -7.7065981 -36.8837774
H 6.2525433 -6.8732437 -36.1891368
C 5.9991926 -8.9928891 -36.0905116
O 5.3798625 -8.8602907 -35.0312186
C 5.3875801 -7.4605357 -38.0417105
H 5.317507 -8.3466364 -38.6717451
H 5.7360643 -6.6360806 -38.6607618
H 4.3964641 -7.2320804 -37.651936
N 6.3537059 -10.227742 -36.5201682
C 6.0921003 -11.4546801 -35.7992746
H 5.0595046 -11.4728627 -35.4552056
C 7.0141917 -11.5868196 -34.5825291
O 6.5442037 -12.022288 -33.5387212
C 6.2944393 -12.5878507 -36.8162142
H 6.5796654 -13.5239315 -36.3340225
H 5.3790469 -12.7278175 -37.3944718
C 6.9710996 -10.6108314 -37.7595143
H 6.2311127 -10.4906961 -38.55036
H 7.8621115 -10.0271148 -37.9027458
C 7.3852351 -12.0701274 -37.7139111
H 7.3632158 -12.5333561 -38.7012058
H 8.3603036 -12.1870356 -37.2408137
N 8.2600128 -11.0993172 -34.6473682
H 8.5974122 -10.7351963 -35.529587
C 9.1638845 -11.0324667 -33.493428
H 9.2559856 -12.0264496 -33.0531509
C 8.6246533 -10.1030979 -32.3918366
O 8.5898426 -10.4804776 -31.2234502
C 10.5463395 -10.5906214 -33.9929249
H 10.9400176 -11.3588206 -34.6604347
H 10.4349591 -9.6795107 -34.5821898
C 11.5645544 -10.3336599 -32.8982995
C 12.1676299 -11.4139774 -32.226674

H 11.9007571 -12.4282594 -32.4853161
C 13.115578 -11.1764532 -31.2147571
H 13.5750579 -12.007349 -30.6985902
C 13.464218 -9.8578058 -30.873536
H 14.194 -9.6750549 -30.0974929
C 12.8624226 -8.7769348 -31.541472
H 13.1324783 -7.7636193 -31.281157
C 11.9107974 -9.0133361 -32.5496745
H 11.4482941 -8.1777601 -33.0568941
N 8.0603085 -8.9495652 -32.7631566
H 8.1434311 -8.665565 -33.7337643
C 7.4168677 -8.0211198 -31.8271501
H 7.9214617 -8.1082216 -30.8649975
C 5.9315139 -8.326438 -31.5456683
O 5.2535744 -7.4801421 -30.961817
C 7.5941536 -6.574922 -32.324633
H 7.124499 -6.4941971 -33.3064549
H 7.0538484 -5.9068789 -31.6550215
C 9.0136829 -6.0386909 -32.3987931
C 9.4463363 -5.359517 -33.5561945
H 8.7912065 -5.2787379 -34.4129759
C 10.7433545 -4.8150995 -33.6203321
H 11.075635 -4.3075551 -34.5142454
C 11.6148391 -4.9453172 -32.5180674
O 12.8717441 -4.435554 -32.5709646
H 13.0345853 -3.9454995 -33.3800472
C 11.174962 -5.6029107 -31.3511048
H 11.8453005 -5.6829437 -30.507972
C 9.8769556 -6.144496 -31.2893049
H 9.5563376 -6.6474406 -30.3872537
N 5.3871766 -9.481238 -31.956426
H 6.0033601 -10.1538206 -32.4006634
C 3.9274016 -9.6951489 -32.0875758
H 3.5788743 -9.0167038 -32.8656313
C 3.101727 -9.3795924 -30.8315989
O 1.9946272 -8.8568 -30.9597617
C 3.6184426 -11.1199467 -32.5819641
H 4.1373467 -11.2775613 -33.5237867
C 4.0262775 -12.229422 -31.6076246
H 3.4924745 -12.148523 -30.6645446
H 5.0959923 -12.1726016 -31.405786
H 3.820187 -13.1996915 -32.0601955
O 2.2341245 -11.2757929 -32.8256162
H 2.1031661 -11.0808149 -33.7675107
N 3.6312656 -9.6287895 -29.6257628
H 4.5649006 -10.0097299 -29.5872965

C 2.9405287 -9.3177676 -28.3687039
H 1.9221669 -9.6969241 -28.445873
C 2.8276563 -7.8017421 -28.1507249
O 1.7167741 -7.2833488 -28.1555535
C 3.6362754 -10.0368499 -27.1973246
H 3.6353806 -11.1098669 -27.3948457
H 4.6745595 -9.7054275 -27.1474315
C 2.978396 -9.7839054 -25.8233671
H 2.9966347 -8.7178673 -25.6005677
C 1.5310448 -10.2812004 -25.7697653
H 1.4817257 -11.3377576 -26.0293787
H 0.9096754 -9.6938931 -26.4446131
H 1.1425322 -10.1385847 -24.7604909
C 3.7720642 -10.514862 -24.7416685
H 3.7563083 -11.5903824 -24.9153329
H 3.3368301 -10.2987848 -23.7649057
H 4.8026104 -10.1599394 -24.7388168
N 3.9616382 -7.0915666 -28.1298216
H 4.8305866 -7.5964343 -28.1841824
C 4.0327294 -5.6286734 -27.9990707
H 3.5475118 -5.3373421 -27.066531
C 3.3223149 -4.9052738 -29.1588863
O 2.7887892 -3.8112358 -29.0000078
C 5.522688 -5.2420034 -27.9073454
H 5.9417744 -5.7125461 -27.0159418
H 6.0387796 -5.6508386 -28.77724
C 5.8148372 -3.7290084 -27.839953
H 5.4302001 -3.2507583 -28.740477
C 5.2052008 -3.0369301 -26.6265279
H 5.5270028 -3.5308572 -25.7079944
H 4.1165442 -3.0902357 -26.6670599
H 5.5002705 -1.9890255 -26.5938106
C 7.3263404 -3.5054407 -27.8182893
H 7.7487717 -3.9367071 -26.9075049
H 7.5460048 -2.4366408 -27.8259105
H 7.7977514 -3.9704279 -28.681937
N 3.3240981 -5.4922525 -30.3565642
H 3.8200164 -6.3736162 -30.4481514
C 2.6217824 -4.9657189 -31.5266407
H 2.8835245 -3.9148142 -31.6417372
C 1.0943839 -5.029999 -31.3514762
O 0.4108227 -4.0473604 -31.6319395
C 3.127867 -5.7370606 -32.7582961
H 4.209855 -5.607543 -32.8206349
H 2.9292394 -6.7975108 -32.6056175
C 2.5111026 -5.3318787 -34.1077532

H 1.4532277 -5.5947615 -34.1197899
C 2.6686143 -3.8435188 -34.4097948
H 3.7203921 -3.5715168 -34.3494023
H 2.0853599 -3.2554452 -33.704076
H 2.2981698 -3.6320755 -35.4139741
C 3.226156 -6.095398 -35.2194507
H 4.2560045 -5.7514367 -35.3113525
H 2.7083162 -5.937219 -36.1650524
H 3.2404669 -7.1588567 -34.9842874
N 0.5617721 -6.1321914 -30.8100106
H 1.1854655 -6.8955181 -30.565335
C -0.8774119 -6.2932057 -30.5294087
H -1.4450707 -5.8133694 -31.3273322
C -1.3387857 -5.5989339 -29.2472027
O -2.4972833 -5.2107496 -29.1799714
C -1.2259879 -7.7857952 -30.5059816
H -0.5395652 -8.3161481 -29.8425807
H -2.2423577 -7.9080194 -30.1304602
C -1.1550235 -8.3653732 -31.9235204
H -0.1595575 -8.2283015 -32.3457716
H -1.8592111 -7.825137 -32.5503861
C -1.517416 -9.8499764 -31.9535904
H -1.7789138 -10.1101266 -32.9786297
H -2.3926915 -10.0247935 -31.3275004
N -0.3815104 -10.6888054 -31.5472774
H 0.4686067 -10.5891001 -32.0922565
C -0.2819976 -11.5284697 -30.5397689
N 0.7987728 -12.2399241 -30.4453776
H 0.9213052 -12.9191248 -29.7195572
H 1.4926744 -12.1243647 -31.1788985
N -1.2140364 -11.691179 -29.6413828
H -2.0524106 -11.1461476 -29.7056909
H -1.0837421 -12.3425586 -28.8909044
N -0.4359914 -5.4137744 -28.291648
H 0.4565919 -5.878876 -28.4106519
C -0.6121594 -4.6637386 -27.0405899
H -1.5484464 -4.9448234 -26.5574101
C -0.6419177 -3.1442925 -27.2699944
O -1.4719593 -2.454789 -26.6849815
C 0.5604857 -5.0831334 -26.1452238
H 0.4619065 -6.1502756 -25.9371324
H 1.4675594 -4.941078 -26.7285774
C 0.7664583 -4.3591921 -24.8161269
H -0.1072872 -4.5150072 -24.1788573
H 0.8875321 -3.2879945 -24.9917974
C 2.035639 -4.9226683 -24.1581527

O 1.8929473 -5.6917077 -23.1831504
O 3.1384206 -4.6239782 -24.6732221
N 0.1917402 -2.6286023 -28.1822229
H 0.9142711 -3.2259607 -28.5641261
C 0.1832659 -1.2150978 -28.5658876
H 0.0037262 -0.6137299 -27.6722889
C -0.9246664 -0.8558517 -29.5768898
O -1.3427934 0.2986789 -29.6289793
C 1.5712314 -0.8568396 -29.111109
H 1.7755088 -1.4407739 -30.0082925
H 2.3275326 -1.0683264 -28.3535512
H 1.5991124 0.2052893 -29.3567835
N -1.3690744 -1.8151607 -30.399589
H -0.9570034 -2.7323259 -30.3107115
C -2.4122034 -1.6197109 -31.4130676
H -2.8401389 -0.6237023 -31.2850044
C -3.5838278 -2.5891446 -31.1989809
O -4.4984085 -2.2893246 -30.4387788
C -1.8158924 -1.6685056 -32.833723
H -1.3145361 -2.624073 -32.987596
H -2.63415 -1.6141187 -33.553035
C -0.8409829 -0.5505884 -33.1512774
C -1.3096435 0.649862 -33.7150379
H -2.3647469 0.7833783 -33.9051926
C 0.531843 -0.7025696 -32.8802269
H 0.8864084 -1.6149005 -32.4260483
C -0.4110316 1.6879453 -34.01348
H -0.7806818 2.6123909 -34.4308109
C 1.4300197 0.3388147 -33.1757978
H 2.4760809 0.2347951 -32.9481857
C 0.9602995 1.5313944 -33.7512922
H 1.6466328 2.3356076 -33.9776088
N -3.6205628 -3.7132496 -31.9236746
H -2.8479066 -3.9034345 -32.5436994
C -4.6046085 -4.7861899 -31.7255437
H -4.6477881 -5.0219769 -30.6606922
C -4.2176418 -6.0541322 -32.4919207
O -3.4466607 -6.006089 -33.4557384
C -6.0082382 -4.3507814 -32.1811528
H -6.3304691 -3.4699273 -31.624698
H -6.7175425 -5.152142 -31.9705056
O -6.0325689 -4.0692292 -33.5679786
H -5.899994 -3.1163384 -33.6518697
N -4.8741413 -7.1737827 -32.163381
H -5.4716506 -7.1487112 -31.3494907
C -4.8665038 -8.4037354 -32.9751168

H -3.8421984 -8.7622554 -33.024553
C -5.296619 -8.1459118 -34.4291164
O -4.6251069 -8.5946963 -35.3565651
C -5.7191938 -9.4992887 -32.2851475
H -5.2617698 -9.6962688 -31.3135578
C -7.1749464 -9.0643969 -32.0189038
H -7.7180113 -8.9252308 -32.9539572
H -7.2157387 -8.1483551 -31.4309694
H -7.6857446 -9.8419113 -31.4478146
C -5.7591062 -10.8389807 -33.0521625
H -6.3486961 -11.5529072 -32.4750014
H -6.2623358 -10.6958331 -34.0075932
C -4.3868889 -11.4732058 -33.3065014
H -3.86465 -11.6184485 -32.3614838
H -3.7929475 -10.8453952 -33.9693974
H -4.5241808 -12.4437534 -33.7840225
N -6.3197801 -7.3081212 -34.6459337
H -6.7867577 -6.9174776 -33.8407924
C -6.8108968 -6.9429317 -35.9822531
H -6.9295796 -7.8697976 -36.5438468
C -5.8010042 -6.0946841 -36.7703672
O -5.7534856 -6.2049544 -37.9993011
C -8.1929232 -6.2678168 -35.828368
H -8.8936588 -6.9935093 -35.4113768
H -8.1035006 -5.4462128 -35.1147961
C -8.795135 -5.6932521 -37.1238768
H -9.7559562 -5.2332397 -36.8894435
H -8.1411857 -4.8942535 -37.4703831
C -9.0162358 -6.7421972 -38.2346595
H -9.9843958 -7.2211919 -38.076221
H -8.2596339 -7.5233973 -38.1830747
C -8.9707821 -6.1540308 -39.6519625
H -9.7832162 -5.4324325 -39.7803459
H -9.1312743 -6.9749907 -40.3575952
N -7.6582374 -5.5172426 -39.9201159
H -7.5238449 -4.6979612 -39.3466649
H -7.5232324 -5.260878 -40.8933727
H -6.8930147 -6.1433565 -39.6825251
N -4.9831557 -5.2914209 -36.0868715
H -5.1114733 -5.2316311 -35.0833861
C -3.8621657 -4.5557308 -36.6755995
H -4.2125805 -3.9476157 -37.5101316
H -3.4303927 -3.8987074 -35.9202956
C -2.7652385 -5.4947951 -37.177779
O -2.4814048 -5.5085537 -38.373279
N -2.2677174 -6.3806718 -36.3070245

H -2.5945535 -6.342489 -35.3479413
C -1.2607822 -7.3958332 -36.6512525
H -0.3445124 -6.8925585 -36.961776
C -1.6939054 -8.2984128 -37.8176158
O -0.9311845 -8.4717807 -38.7673839
C -0.9587692 -8.2239316 -35.3945171
H -0.5126998 -7.5591821 -34.6545479
H -1.9003645 -8.5918536 -34.9852958
C -0.0205586 -9.403772 -35.5957312
C -0.476939 -10.7237244 -35.4080804
H -1.509788 -10.9114615 -35.1526382
C 0.4018223 -11.8087914 -35.5828742
H 0.0411055 -12.8215519 -35.4605813
C 1.7396798 -11.5814567 -35.9510346
H 2.4073452 -12.4177367 -36.1126058
C 2.197147 -10.2669613 -36.1448257
H 3.2167878 -10.0866761 -36.4561706
C 1.3158525 -9.1847377 -35.975059
H 1.6594669 -8.1812815 -36.1674815
N -2.9508894 -8.7602365 -37.8162996
H -3.5180262 -8.6013591 -36.9897401
C -3.5164419 -9.5705426 -38.9055008
H -2.8830213 -10.4489155 -39.0412821
C -3.4884375 -8.8191223 -40.2441416
O -3.0854456 -9.4032043 -41.2444796
C -4.9353343 -10.0606912 -38.5229828
H -5.4930771 -9.2156787 -38.1147458
C -5.7199256 -10.6008875 -39.7362459
H -5.1748012 -11.428586 -40.1934969
H -5.8704642 -9.8203191 -40.4808494
H -6.7059161 -10.950001 -39.4339102
C -4.8187313 -11.1576382 -37.4374046
H -4.3660146 -12.0503218 -37.8713733
H -4.1598068 -10.814504 -36.6407699
C -6.1553173 -11.5460725 -36.7923586
H -6.7955629 -12.0580817 -37.5094256
H -6.6616767 -10.6550823 -36.4198857
H -5.9674939 -12.2254155 -35.9610021
N -3.8323925 -7.5248185 -40.2879394
H -4.0656676 -7.0556824 -39.4218744
C -3.7691065 -6.745843 -41.5391746
H -4.3005425 -7.2994218 -42.3139513
C -2.3393446 -6.5519942 -42.0526116
O -2.1145754 -6.6247537 -43.2621331
C -4.4332741 -5.3718712 -41.3703427
H -3.8537605 -4.6109815 -41.8970195

H -4.4733346 -5.1006908 -40.3138927
O -5.7412933 -5.3987764 -41.9212897
H -5.6390313 -5.3843889 -42.8827614
N -1.3861787 -6.3126124 -41.1495095
H -1.6547347 -6.2826085 -40.1714205
C 0.0158965 -6.0425797 -41.481886
H 0.0577829 -5.3112942 -42.2894989
C 0.740751 -7.3012983 -41.9896076
O 1.4825599 -7.2298511 -42.9719905
C 0.6993255 -5.4537121 -40.2332656
H 0.5759614 -6.161702 -39.4119538
H 1.768021 -5.3583632 -40.4270006
C 0.1460188 -4.0790425 -39.7977291
H -0.9410754 -4.0968931 -39.8068985
C 0.5881962 -3.7787531 -38.3651539
H 1.6688825 -3.8436922 -38.2817429
H 0.1358835 -4.4987693 -37.6855946
H 0.2728934 -2.7773283 -38.0815491
C 0.5838749 -2.9467471 -40.7300837
H 1.6679734 -2.8669288 -40.751094
H 0.1714852 -2.0035095 -40.3724266
H 0.2150466 -3.1247907 -41.7383577
N 0.4550266 -8.4611916 -41.3890336
H -0.1379686 -8.4416052 -40.564342
C 0.9646168 -9.7595951 -41.8364496
H 2.0196674 -9.6433663 -42.0892642
C 0.2485841 -10.2596498 -43.1035624
O 0.8987943 -10.7616169 -44.0158858
C 0.8455888 -10.7511466 -40.6638834
H 1.3523783 -10.326376 -39.7955445
H -0.210257 -10.8640917 -40.4110157
C 1.4357562 -12.1477194 -40.9424942
H 0.9147976 -12.6038002 -41.7839374
C 2.9345315 -12.1074372 -41.2461209
H 3.4682867 -11.5756565 -40.4587843
H 3.1048157 -11.6059846 -42.1990644
H 3.3258448 -13.1209115 -41.3293987
C 1.228386 -13.036948 -39.7157139
H 1.7534838 -12.6171902 -38.8578697
H 1.6078131 -14.0384338 -39.9180822
H 0.1647686 -13.1057515 -39.4881301
N -1.0698244 -10.0583188 -43.2209953
H -1.5784126 -9.6705319 -42.4337335
C -1.8288138 -10.4543792 -44.4133578
H -1.6406428 -11.512455 -44.6026739
C -1.3632127 -9.6903915 -45.6534104

O -1.0336879 -10.3147171 -46.6612399
C -3.3368109 -10.2801862 -44.1613091
H -3.5384081 -9.2595103 -43.8397539
H -3.6148275 -10.9493552 -43.3461491
C -4.2354917 -10.6192243 -45.3629775
H -5.2387681 -10.8120393 -44.981554
H -3.8764807 -11.5469091 -45.8110439
S -4.390968 -9.3657168 -46.6781462
C -5.1799099 -8.0079527 -45.7729399
H -5.4383667 -7.2116522 -46.4701612
H -4.4931662 -7.6138861 -45.0242914
H -6.0870682 -8.3705426 -45.2881865
N -1.2836702 -8.3547994 -45.597801
H -1.5351508 -7.8696527 -44.7427603
C -0.9788531 -7.5879528 -46.8043397
H -1.457325 -8.1034868 -47.6360249
C 0.521404 -7.5756896 -47.1451478
O 0.8637811 -7.5102657 -48.3264741
C -1.6430715 -6.2032923 -46.748782
H -1.1355764 -5.5871202 -46.0089988
H -2.6802938 -6.3144597 -46.4385527
C -1.6394516 -5.5151375 -48.1094585
N -0.9709235 -4.389742 -48.2157069
H -0.4685428 -4.0199304 -47.4270163
H -0.9501478 -3.9346266 -49.1103779
O -2.2486604 -5.9590541 -49.0785727
N 1.416904 -7.7516498 -46.1678263
H 1.0980991 -7.8154289 -45.2078644
C 2.8375119 -7.9866019 -46.4363406
H 3.1969886 -7.2710589 -47.1758242
H 3.3911814 -7.8211393 -45.5225839
C 3.1279278 -9.4043741 -46.945777
O 3.8298141 -9.5493483 -47.9453507
N 2.447086 -10.4225332 -46.4101552
H 1.9315207 -10.2624063 -45.5490469
C 2.4854058 -11.7979805 -46.9139969
H 3.5152832 -12.1562071 -46.9087821
H 1.8931677 -12.4278665 -46.2504679
C 1.9239234 -11.9295401 -48.3328251
O 2.5932635 -12.4826701 -49.2022689
N 0.7929469 -11.2777152 -48.628854
H 0.267872 -10.8632915 -47.8641561
C 0.2683398 -11.1228987 -49.9938683
H 0.0573556 -12.1067264 -50.4128921
C 1.2826057 -10.4351154 -50.919821
O 1.4660857 -10.8427349 -52.0678355

C -1.03995 -10.3126457 -49.9511539
H -1.8060414 -10.9034882 -49.4476874
H -0.8743164 -9.4136616 -49.3588149
C -1.5543657 -9.8836418 -51.2950987
C -1.1187229 -8.7991881 -51.9758444
H -0.3563124 -8.1147227 -51.6133052
N -1.6791194 -8.7882528 -53.2363242
H -1.3736528 -8.1625098 -53.9676252
C -2.5438482 -9.8457334 -53.4186151
C -3.3411695 -10.2526051 -54.4992947
H -3.3476281 -9.6931163 -55.422582
C -4.1539985 -11.3894695 -54.358604
H -4.801853 -11.6994674 -55.1695271
C -4.1314936 -12.1172815 -53.1550939
H -4.7589197 -12.9925633 -53.044334
C -3.3101688 -11.7105559 -52.0842815
H -3.3046579 -12.2804389 -51.1652787
C -2.499787 -10.5592805 -52.1831049
N 1.96428 -9.3993704 -50.4253499
H 1.7809878 -9.1156849 -49.4709382
C 2.9636726 -8.654149 -51.2011413
H 2.5137098 -8.3680451 -52.1510126
C 4.1751778 -9.5252683 -51.5380323
O 4.584636 -9.5468988 -52.6991712
C 3.3562456 -7.3613529 -50.4714756
H 3.7076416 -7.5770001 -49.4645344
C 4.4220107 -6.5580398 -51.2039862
H 4.0784879 -6.3762013 -52.2179027
H 5.3645424 -7.1059384 -51.2128172
H 4.574357 -5.6062331 -50.6915057
O 2.2203371 -6.5288334 -50.4111686
H 1.6945377 -6.8265429 -49.6442254
N 4.6517677 -10.3483214 -50.5991615
H 4.2697926 -10.2894075 -49.6593257
C 5.6772822 -11.3580867 -50.8568759
H 6.5451977 -10.8700353 -51.3041686
C 5.1923812 -12.4341222 -51.8490049
O 5.8968642 -12.7354913 -52.8110289
C 6.1067911 -11.9647842 -49.5145566
H 5.2580115 -12.4435631 -49.0255728
H 6.499734 -11.1841373 -48.8617151
H 6.8863492 -12.7084471 -49.6781218
N 3.9534366 -12.9249302 -51.7086818
H 3.4267844 -12.6496469 -50.8836684
C 3.334672 -13.8997941 -52.6291921
H 3.9737587 -14.7791788 -52.6745089

C 3.2311635 -13.3581777 -54.0601941
O 3.501972 -14.0879971 -55.0132972
C 1.9611062 -14.3607064 -52.0920771
H 1.4005589 -13.4924383 -51.7508018
C 1.0987965 -15.094003 -53.1308964
H 1.6582642 -15.9270397 -53.5595551
H 0.8015677 -14.4086898 -53.9226587
H 0.1946828 -15.4764729 -52.6554527
C 2.1541222 -15.3188572 -50.9094302
H 2.6359165 -16.2380737 -51.2437664
H 1.18814 -15.559973 -50.4658729
H 2.7765934 -14.8574841 -50.1444956
N 2.9105554 -12.073782 -54.2549198
H 2.6628376 -11.510579 -53.4444772
C 2.8382215 -11.4848562 -55.603466
H 2.5477263 -12.2731829 -56.294507
C 4.1932573 -11.0523229 -56.1735234
O 4.3050569 -11.0014922 -57.3976465
C 1.7500749 -10.3972881 -55.6684607
H 1.973677 -9.6224873 -54.932472
H 1.7766916 -9.9369756 -56.657886
C 0.3181127 -10.9321047 -55.4242165
H 0.2277581 -11.265099 -54.390261
H -0.3748739 -10.1024204 -55.5677658
C -0.0987512 -12.0894509 -56.3560436
H 0.544746 -12.9544503 -56.17014
H 0.0153415 -11.7777132 -57.39766
C -1.5577073 -12.5187353 -56.1029819
H -2.2269318 -11.6802266 -56.3325773
H -1.6736438 -12.7561101 -55.0366384
N -1.920309 -13.7026939 -56.930816
H -1.8494992 -13.5031153 -57.9228767
H -2.903941 -13.9547134 -56.8042702
H -1.3493329 -14.5134468 -56.71273
N 5.239108 -10.8811623 -55.358782
H 5.0832237 -10.9063656 -54.3572045
C 6.6252368 -10.7490509 -55.8568326
H 6.6136699 -10.1725473 -56.7835268
C 7.23075 -12.111499 -56.2120809
O 7.8163342 -12.237301 -57.2830009
C 7.5248285 -9.9796415 -54.8686851
H 7.4639628 -10.4501565 -53.8877875
C 8.9905939 -9.944826 -55.3227672
H 9.0568977 -9.5997377 -56.3555774
H 9.4372889 -10.9357012 -55.2428839
H 9.5579733 -9.2635562 -54.6896495

C 7.0793244 -8.5186274 -54.7449023
H 7.2214826 -8.0117064 -55.6995769
H 7.6884329 -8.0243809 -53.9867521
H 6.0354079 -8.4688468 -54.4529373
N 7.0278598 -13.1595392 -55.4024952
H 6.5409429 -13.011564 -54.5230944
C 7.6271667 -14.4858677 -55.6559459
H 8.6634613 -14.3031979 -55.9438593
C 6.9906641 -15.2279986 -56.8419336
O 7.6716931 -15.9879257 -57.5225639
C 7.6575082 -15.3317962 -54.3638388
H 7.9124323 -14.6589608 -53.5431137
C 6.2996944 -15.9707711 -54.0400428
H 6.0816508 -16.7851927 -54.7321286
H 5.5262014 -15.2171777 -54.1221082
H 6.2972443 -16.3543042 -53.0201916
C 8.777694 -16.3911983 -54.4438692
H 8.5331507 -17.1303691 -55.2074872
H 9.7068405 -15.8989223 -54.7355244
C 9.0351373 -17.1189259 -53.1182629
H 8.173149 -17.7267722 -52.8436591
H 9.2387219 -16.3944539 -52.3289923
H 9.8999306 -17.7735446 -53.2299662
N 5.7387061 -14.9039989 -57.1868191
H 5.2127234 -14.3222172 -56.5485866
C 5.1062204 -15.3009733 -58.4534771
H 5.3874356 -16.3318692 -58.6680816
C 5.6030433 -14.4874707 -59.672003
O 5.0849158 -14.6599443 -60.773668
C 3.5735904 -15.2270815 -58.2905951
H 3.3168007 -14.2373792 -57.910768
H 3.1057086 -15.3358337 -59.269651
C 2.9775319 -16.3018904 -57.3585176
H 3.4899355 -16.2918303 -56.3990126
C 1.4957103 -15.9962792 -57.1210999
H 0.9610944 -15.9934025 -58.0710052
H 1.4152946 -15.0272253 -56.6348142
H 1.0717324 -16.755584 -56.4636815
C 3.0815974 -17.7101515 -57.947436
H 2.616133 -17.7444842 -58.9324024
H 2.5847047 -18.4241014 -57.2906702
H 4.1275589 -18.0041337 -58.0324987
N 6.5584693 -13.5695965 -59.4840437
H 6.9635998 -13.4805897 -58.559394
C 7.1419561 -12.7294521 -60.533019
H 8.0300305 -12.2428227 -60.1296168

H 7.4428545 -13.3604889 -61.3694312
C 6.2113295 -11.6372743 -61.0664694
O 6.4668133 -11.1007314 -62.1414297
N 5.0989407 -11.3521821 -60.3807085
H 4.9813281 -11.7997437 -59.4822739
C 4.011173 -10.5016823 -60.8819061
H 4.0372055 -10.488733 -61.9692609
C 4.1458654 -9.0360507 -60.4550618
O 4.0311999 -8.1487806 -61.2999311
C 2.6640571 -11.1114873 -60.4638078
H 1.8926477 -10.351034 -60.4842377
H 2.730934 -11.4831747 -59.4414438
C 2.2535011 -12.2657448 -61.3868724
H 1.4591474 -12.8339579 -60.902344
H 3.1069454 -12.932034 -61.5231857
S 1.6498686 -11.7327541 -63.0139356
C 1.6214996 -13.3280329 -63.8756168
H 1.2174771 -13.1904539 -64.8799789
H 0.9970689 -14.0358844 -63.3303468
H 2.6341481 -13.7267153 -63.9450518
N 4.4403578 -8.7747006 -59.1755103
H 4.5741277 -9.5745046 -58.565276
C 4.7721047 -7.4432695 -58.6461105
H 4.2329189 -6.6929209 -59.2287529
C 6.2628089 -7.1513591 -58.8255713
O 7.1132365 -8.0286716 -58.7190631
C 4.3124856 -7.3225627 -57.1776623
H 3.2218766 -7.3201566 -57.1538076
H 4.664482 -8.1940433 -56.6248092
C 4.8413101 -6.0586761 -56.4669405
H 5.9269408 -6.1201353 -56.3940316
H 4.5835999 -5.1738691 -57.0510368
C 4.2767258 -5.8952971 -55.0518431
H 4.2724669 -6.8599056 -54.5417714
H 3.2469937 -5.5465683 -55.1283597
C 5.0870934 -4.8828455 -54.2236432
H 4.4787623 -4.580427 -53.3658758
H 5.2958632 -3.991815 -54.8235343
N 6.3573003 -5.466491 -53.7188249
H 6.2395348 -6.3113772 -53.1868018
H 6.9070721 -4.8226841 -53.1439003
H 7.0461657 -5.6510471 -54.4521823
N 6.5831059 -5.8796803 -59.0166479
H 5.8235267 -5.2105063 -59.113986
C 7.9375657 -5.3227778 -59.0310328
H 8.6143732 -5.9856498 -58.4939189

C 7.9299697 -3.9753104 -58.3016906
O 6.8849011 -3.3299543 -58.2056804
C 8.3940536 -5.2114097 -60.498702
H 7.5989185 -4.7461053 -61.083704
C 9.6867695 -4.4393521 -60.7534294
H 10.461601 -4.7634189 -60.0624864
H 9.5092652 -3.3712309 -60.637248
H 10.0261984 -4.6212524 -61.7728531
O 8.6164189 -6.5095718 -60.9963761
H 8.1255677 -7.1366062 -60.4489059
N 9.0868513 -3.5097747 -57.8288001
H 9.9133854 -4.0836863 -57.9019445
C 9.2842826 -2.0970461 -57.484942
H 8.3344924 -1.5820776 -57.6283965
C 10.2397767 -1.4588863 -58.488381
O 11.1822076 -2.0878065 -58.9748673
C 9.6723067 -1.8988096 -55.9998239
H 9.4745394 -2.8358148 -55.4851521
C 11.1589555 -1.5434698 -55.8111287
H 11.3819425 -0.5661663 -56.2441685
H 11.7740098 -2.3086833 -56.2769605
H 11.4226248 -1.4985324 -54.7557414
C 8.7636256 -0.8305709 -55.3496209
H 8.9602225 0.1445593 -55.7991688
H 7.7226656 -1.0931424 -55.5430241
C 8.9349449 -0.7232145 -53.8274753
H 9.9006709 -0.2819557 -53.5826193
H 8.8562241 -1.7099889 -53.3708842
H 8.1532925 -0.0869728 -53.4164581
N 9.9517901 -0.2103244 -58.8094482
H 9.1682783 0.231535 -58.3373574
C 10.654951 0.6360141 -59.7608788
H 11.4393476 0.0802286 -60.2761536
C 11.3137825 1.7707113 -58.9834636
O 10.6744127 2.3280577 -58.0948429
C 9.5994166 1.0937026 -60.7929687
H 8.6089785 1.028997 -60.3467226
H 9.6158493 0.3827572 -61.6188321
C 9.6756245 2.4856417 -61.3678588
N 9.6560584 2.7683857 -62.7304314
C 9.6304442 4.1041051 -62.8320208
H 9.6677495 4.647484 -63.7669766
N 9.599696 4.6686446 -61.6145544
H 9.6754936 5.6576488 -61.4159251
C 9.6037052 3.6646771 -60.6808175
H 9.5692624 3.7963986 -59.6106405

N 12.5267095 2.1509732 -59.3842939
H 13.0058659 1.5797279 -60.0714761
C 13.1036285 3.4786336 -59.1419166
H 12.3791542 4.0801703 -58.6065819
C 13.4003518 4.137936 -60.4980748
O 13.9804095 3.4895145 -61.3751488
C 14.3486196 3.3947989 -58.2404563
H 14.0355429 3.0345998 -57.2602812
H 15.0443904 2.6671256 -58.661884
C 15.0998792 4.7290328 -58.0561606
H 15.5972644 4.9762276 -58.9978091
H 15.8861092 4.5829474 -57.3125827
C 14.2028056 5.9034645 -57.6227446
O 14.1451209 6.8963968 -58.3833069
O 13.5576779 5.8054743 -56.5593021
N 13.0453446 5.4126846 -60.6712948
H 12.6425479 5.9066621 -59.8759317
C 13.3632583 6.2062371 -61.8533931
H 14.1139036 5.6662568 -62.4167197
C 13.9496037 7.5757594 -61.5001384
O 13.3110616 8.3851175 -60.8299668
C 12.1357501 6.3519771 -62.752082
H 11.3121412 6.7960327 -62.1946084
H 11.8710934 5.3727228 -63.1411174
H 12.3807769 6.9994993 -63.5956822
N 15.109189 7.8745898 -62.0873356
H 15.4773862 7.2053572 -62.7563538
C 15.8432781 9.1316037 -61.9126432
H 15.2448618 9.8123746 -61.30726
C 16.0561624 9.7692783 -63.2824116
O 16.4188304 9.0839867 -64.2349318
C 17.1881392 8.8969502 -61.188147
H 17.8076121 8.2561194 -61.8169794
C 16.9764192 8.1765845 -59.8366834
H 16.3622953 8.7964619 -59.1838709
H 16.4432474 7.2414061 -60.0042166
C 17.9261826 10.2389898 -60.9959842
H 17.3004281 10.929227 -60.4298202
H 18.1645092 10.6855659 -61.9607414
H 18.8686102 10.0894265 -60.4738383
C 18.2749524 7.8099776 -59.110778
H 18.7708445 8.7035771 -58.7336403
H 18.9412565 7.2714474 -59.7855677
H 18.0370505 7.1684199 -58.2616609
N 15.8801458 11.0823937 -63.3805037
H 15.5331748 11.5872138 -62.5688742

C 15.9623305 11.817571 -64.6442913
H 16.7952455 11.4316431 -65.2351664
C 16.2306405 13.3158319 -64.4223685
O 16.001996 13.8407697 -63.3256233
C 14.6780471 11.5721577 -65.4405948
H 14.5614297 12.3592306 -66.1828294
H 14.8143697 10.6397859 -65.9771394
C 13.3981278 11.4233831 -64.5967226
H 13.2409175 12.3213791 -64.0054631
H 13.4843556 10.5777558 -63.912386
C 12.2010246 11.1445692 -65.486179
O 12.4220538 10.9836834 -66.7062945
O 11.0711677 11.0577901 -64.952999
N 16.7127389 14.0282412 -65.4533237
H 16.8577185 13.566903 -66.349382
C 16.9273486 15.4814323 -65.3898288
H 16.3871383 15.8476574 -64.5193038
C 16.3447654 16.2645858 -66.5817988
O 16.1558116 15.7419953 -67.6835179
C 18.4109936 15.8096423 -65.1287159
H 18.8261429 15.0930979 -64.4212154
H 18.4804463 16.7986531 -64.677849
C 19.2719235 15.817902 -66.3805842
O 19.9812442 14.8726451 -66.670495
N 19.2629312 16.8803734 -67.1552989
H 19.7115882 16.7659892 -68.0471681
H 18.6938739 17.6945154 -66.9492704
N 16.1171737 17.5560555 -66.3461034
H 16.3244087 17.9218972 -65.4216912
C 15.6409335 18.5213459 -67.3311437
H 15.1563779 17.9880668 -68.1515813
C 16.8207639 19.3019001 -67.9050657
O 17.814395 19.5196466 -67.2156087
C 14.6025547 19.470826 -66.7156745
H 15.0457816 19.9951369 -65.8663646
H 13.7532703 18.8943344 -66.3687152
C 14.1049765 20.4776596 -67.7549781
O 14.0497 21.6808959 -67.4357038
O 13.8939207 20.0301459 -68.9036003
N 16.6932574 19.7127438 -69.1587056
H 15.7361606 19.6535177 -69.5221682
C 17.6417525 20.4891035 -69.9664808
H 18.1709276 19.7718774 -70.5775385
C 18.7397635 21.2279062 -69.1911158
O 19.6163392 20.5303848 -68.6231238
O 18.7905354 22.4280729 -69.0989548

C 16.8603575 21.4153103 -70.9236658
H 16.3055296 22.1240006 -70.3041713
H 17.5800612 21.9809911 -71.516076
C 15.8643249 20.7213124 -71.8783228
H 15.1037594 20.1933291 -71.2956912
C 15.1458514 21.7861056 -72.7164382
H 15.8579213 22.3362843 -73.3312233
H 14.6219113 22.4788679 -72.0561909
H 14.4058411 21.3071409 -73.3609419
C 16.5435476 19.7199673 -72.8232848
H 17.329409 20.2094041 -73.398468
H 15.8002973 19.3095617 -73.5106352
H 16.9602655 18.8885803 -72.2557724

Supplementary Data 6. Cartesian coordinates for perivine docked into CrPeNMT+SAM.

N -2.1385667 21.7753992 -15.9844882
H -2.8881317 22.3451992 -15.612073
H -2.4187043 21.4412796 -16.898962
H -1.952445 20.9940613 -15.3721405
C -0.8983927 22.6008534 -16.1048954
H -1.0805973 23.3575681 -16.8709468
C 0.33849 21.7897622 -16.5659561
O 1.2553163 22.327476 -17.1874333
C -0.6053818 23.3116885 -14.7771474
H 0.3668547 23.80077 -14.8479421
H -0.5567763 22.5711725 -13.9778747
C -1.639947 24.3835703 -14.4160168
H -1.396285 24.7758699 -13.426708
H -2.6273748 23.9290305 -14.3587376
S -1.7285502 25.7740286 -15.5733039
C -2.9534829 26.7844932 -14.6928753
H -3.1342375 27.7045542 -15.2488035
H -2.5798462 27.0332981 -13.6984196
H -3.8891334 26.2323095 -14.5953778
N 0.3673335 20.4886197 -16.2657559
H -0.4109708 20.1080689 -15.7405706
C 1.3938537 19.5859753 -16.7771625
H 1.3580658 19.6103407 -17.8672226
H 2.3753087 19.9422459 -16.4680576
C 1.2496557 18.1385969 -16.3224611
O 0.7986647 17.8537127 -15.2154758
N 1.7628938 17.2390447 -17.1522057
H 2.1121366 17.5432004 -18.0501647
C 2.0288584 15.8382482 -16.8338954
H 1.9028599 15.7027836 -15.7604402
C 3.4957816 15.5076051 -17.1394012
O 4.119702 16.1812749 -17.9609391
C 0.9990926 14.9223526 -17.5032517
H 1.2572628 13.8928438 -17.2749979
H 0.0423459 15.1358366 -17.026009
C 0.8149397 15.0455348 -19.020348
H 1.6157817 14.5050359 -19.5288645
H 0.8699863 16.0954136 -19.3176114
C -0.5554566 14.4754363 -19.4180847
O -1.297864 15.2053148 -20.1119062
O -0.875195 13.3478418 -18.9747065
N 4.1061184 14.7016468 -16.2632362
H 3.5442127 14.1102083 -15.6715657
C 5.5629684 14.6122266 -16.0983634

H 6.0446756 14.8186955 -17.0600251
C 5.9695982 13.2129132 -15.6736854
O 5.3463653 12.639753 -14.786269
C 6.0594875 15.5900786 -15.0182406
H 5.5966651 15.3285324 -14.0641537
H 7.1389711 15.4636292 -14.915048
C 5.7688522 17.0627619 -15.3210483
H 6.199365 17.3109927 -16.2919241
H 4.6914859 17.2284646 -15.351451
C 6.3694499 17.9615862 -14.2310701
H 7.4516987 17.8220036 -14.192695
H 5.9514537 17.6743696 -13.2638997
C 6.0413128 19.4366002 -14.4861744
H 6.4275873 20.029751 -13.6516003
H 4.9522862 19.5499365 -14.5026758
N 6.6253332 19.9107507 -15.7656037
H 7.6331593 19.818804 -15.7546612
H 6.3843249 20.8792368 -15.933991
H 6.2683797 19.3550584 -16.5356419
N 7.106455 12.7593095 -16.1705568
H 7.4956956 13.2759729 -16.9574346
C 7.5639831 11.383838 -16.027934
H 6.7220861 10.7521143 -15.7410336
C 8.6256294 11.3080201 -14.9245199
O 9.6775993 11.9537076 -14.9983873
C 8.0743326 10.8706348 -17.3888252
H 8.9570019 11.4416081 -17.6791626
H 8.3787829 9.8302572 -17.2664366
C 7.0169986 10.9370419 -18.5172246
H 6.1405201 10.371064 -18.1914962
H 7.409044 10.4393792 -19.4038394
C 6.5974706 12.3719046 -18.896483
O 7.4785217 13.2681533 -18.9084406
O 5.3745041 12.6072441 -18.9936412
N 8.3366279 10.5700175 -13.8495991
H 7.428984 10.1185224 -13.7997984
C 9.2537472 10.4628666 -12.7165948
H 9.411715 11.4704627 -12.3299929
C 10.6243327 9.9036971 -13.153139
O 10.6986872 8.9385328 -13.9069108
C 8.6037136 9.6350831 -11.6036772
H 8.4204313 8.619799 -11.9600852
H 7.655759 10.0896643 -11.3114457
H 9.2647124 9.595986 -10.737355
N 11.703524 10.5367378 -12.6721267
H 11.5296608 11.3030954 -12.0418231

C 13.1142625 10.3142897 -13.0702562
H 13.6516325 11.146085 -12.6173586
C 13.4243279 10.5119185 -14.5675031
O 14.4681525 10.0599575 -15.0339048
C 13.7765064 9.0619071 -12.4395625
H 14.845682 9.122573 -12.6445486
C 13.6265139 9.0675566 -10.9118445
H 12.5803642 8.9423089 -10.6318339
H 14.0018322 10.0070241 -10.506304
H 14.2061243 8.2486758 -10.485005
C 13.2962742 7.7038875 -12.9652798
H 12.2890587 7.4911381 -12.6080123
H 13.9637118 6.9192992 -12.6112727
H 13.2950871 7.7094786 -14.0550769
N 12.605294 11.3349009 -15.2378421
H 11.7369915 11.5814565 -14.7805248
C 12.7314974 11.833814 -16.6111805
H 11.8724211 12.4921825 -16.7495073
C 12.6154246 10.7757957 -17.7237248
O 13.1625965 9.6728211 -17.644985
C 13.9716254 12.728998 -16.7542726
H 14.876663 12.1276992 -16.6566322
H 13.9650252 13.5017634 -15.9855672
H 13.9695782 13.2008787 -17.7373648
N 11.9102878 11.1457732 -18.7984009
H 11.4901366 12.0606529 -18.8311174
C 11.6113386 10.2161504 -19.8849453
H 11.4683386 9.2575284 -19.3922061
C 12.7697246 10.041093 -20.8749029
O 13.4841971 10.9883786 -21.2103005
C 10.2956217 10.5834444 -20.6018401
H 9.5642602 10.8737195 -19.8541547
H 10.4613373 11.4376453 -21.2598264
C 9.7172444 9.4047201 -21.4196476
H 10.350257 9.2350742 -22.2929309
H 8.7362979 9.7002982 -21.7986705
C 9.5899082 8.089336 -20.6183328
O 8.4815808 7.7830257 -20.1380566
O 10.6205886 7.3866881 -20.4498584
N 12.8901662 8.8305339 -21.4204041
H 12.2017952 8.1247666 -21.1470783
C 13.7998552 8.4972353 -22.5092701
H 14.1771403 9.4236569 -22.9434863
C 13.0399482 7.741936 -23.6088019
O 12.2666108 6.8259826 -23.3460136
C 14.9936922 7.7225667 -21.9245386

H 15.5173435 8.3745069 -21.2235556
H 14.6001376 6.8863099 -21.3570609
C 16.0029798 7.189281 -22.9591753
H 15.4878043 6.568367 -23.6890142
C 16.7306364 8.3210255 -23.6896215
H 17.2504768 8.9574053 -22.9726436
H 16.0235264 8.9250527 -24.2541046
H 17.4549132 7.90003 -24.3877077
C 17.0509741 6.3236455 -22.2584349
H 17.5894864 6.9110609 -21.5149428
H 17.7563171 5.9395483 -22.9960493
H 16.5630007 5.4766885 -21.778768
N 13.2962296 8.1160457 -24.8611726
H 13.9627613 8.8548464 -25.0049569
C 12.7297022 7.4720004 -26.0467334
H 11.6552424 7.6561114 -26.0668372
C 12.963355 5.9483119 -26.0268958
O 14.0825531 5.4964434 -25.7932219
C 13.3669937 8.1475173 -27.2671617
H 13.1403425 9.2139322 -27.2266732
H 14.4513269 8.049724 -27.187207
C 12.9656811 7.6189319 -28.6307211
C 12.1494424 8.3971049 -29.4761888
H 11.769383 9.3526089 -29.1454154
C 11.8565891 7.9520163 -30.7798108
H 11.2562024 8.555452 -31.4472005
C 12.3673248 6.7178848 -31.2374061
O 12.1160781 6.2857259 -32.4969706
H 11.5991475 6.9488647 -33.0185918
C 13.1697677 5.9340538 -30.3847744
H 13.5492799 4.9896701 -30.7459872
C 13.4827043 6.3907892 -29.091865
H 14.1273567 5.791667 -28.4621886
N 11.8951892 5.1788313 -26.2599184
H 11.0304658 5.6427895 -26.4800882
C 11.8426092 3.7036502 -26.2137578
H 10.7811114 3.4599108 -26.2631227
C 12.3180866 3.0493723 -24.893297
O 12.588105 1.8486882 -24.8229072
C 12.4769853 3.1028593 -27.4818833
H 13.5610504 3.0598399 -27.3602348
H 12.2582179 3.7523311 -28.3311342
C 11.9188234 1.7098089 -27.8129528
O 10.6734795 1.5608478 -27.7641107
O 12.7303627 0.8270589 -28.169355
N 12.3786583 3.8077385 -23.7892188

H 12.1567416 4.7960764 -23.8565606
C 12.7236866 3.2518266 -22.4780661
H 13.5786598 2.5902866 -22.6181944
C 11.5733328 2.4149249 -21.9209309
O 10.5345585 2.9233827 -21.5059758
C 13.1586237 4.3717878 -21.5322934
H 14.0067916 4.8753226 -21.9927235
H 12.3398627 5.0838206 -21.4074609
C 13.5708913 3.8411374 -20.1519667
H 12.6903613 3.4453154 -19.6452956
H 14.3110593 3.0473288 -20.2626016
C 14.1595742 4.9730826 -19.3074994
H 13.477531 5.8273082 -19.3386531
H 15.1255757 5.265841 -19.7198963
C 14.3246592 4.5251755 -17.8558798
H 14.9647772 3.6393135 -17.8237746
H 13.3343658 4.2459127 -17.4769636
N 14.894404 5.6206571 -17.034387
H 15.8026652 5.9022239 -17.3743342
H 14.9585333 5.3479973 -16.0632593
H 14.281514 6.4323719 -17.0835563
N 11.8145313 1.1129027 -21.796752
H 12.636759 0.7428888 -22.2469702
C 10.915173 0.206206 -21.075752
H 9.9261211 0.3116122 -21.5233934
C 10.7879049 0.6022152 -19.5986625
O 11.7565775 1.0128309 -18.9601126
C 11.3345322 -1.270057 -21.22834
H 10.6296722 -1.8783407 -20.6606041
C 11.2418011 -1.7098514 -22.6941182
H 11.9608367 -1.1650701 -23.3087444
H 10.2395993 -1.5174528 -23.0780593
H 11.4468521 -2.7773516 -22.7753339
C 12.7458793 -1.5768076 -20.7095885
H 13.4907476 -1.0077613 -21.2656222
H 12.9570051 -2.6395233 -20.8284199
H 12.8214824 -1.3296274 -19.6506744
N 9.6090573 0.3641778 -19.0190877
H 8.8636369 0.042936 -19.61778
C 9.3030641 0.4995601 -17.5765515
H 8.2498576 0.2378134 -17.4888618
C 9.3464409 1.9102799 -16.9589934
O 9.2888891 2.018324 -15.7386553
C 10.0341763 -0.545724 -16.7016046
H 9.6693134 -0.4676256 -15.6770594
C 9.7794235 -1.9797159 -17.169852

H 10.215899 -2.1450359 -18.1540049
H 8.7079161 -2.1740235 -17.2032576
H 10.239578 -2.6699839 -16.4624216
O 11.4299275 -0.3613229 -16.691399
H 11.6667592 0.1678767 -17.4727227
N 9.2779939 2.9909397 -17.7450031
H 9.3809 2.8980378 -18.7455964
C 8.8924366 4.3186524 -17.2226461
H 9.484174 4.5459203 -16.3361664
C 7.3950813 4.3363773 -16.8420819
O 6.54833 4.1563548 -17.7157283
C 9.1737252 5.4060425 -18.2724269
H 8.8412563 6.3707971 -17.8859208
H 8.6096819 5.1895781 -19.1816057
O 10.5517975 5.4957348 -18.5958262
H 10.5894134 6.1754387 -19.3178923
N 7.0367162 4.4791013 -15.5515566
H 7.7637049 4.4744686 -14.8485576
C 5.6335974 4.372326 -15.0850094
H 5.0070584 4.6318068 -15.9400818
C 5.0839267 5.3356751 -13.9496445
O 4.1802507 4.8921545 -13.2441249
C 5.3501324 2.8742188 -14.7954481
H 5.6601937 2.2734319 -15.6495461
H 4.2770818 2.7385852 -14.6680957
C 6.0481724 2.3356799 -13.5563096
O 6.9355157 2.9397106 -12.9802825
N 5.6839717 1.1536912 -13.1130988
H 6.1721809 0.8278604 -12.2984982
H 4.9434468 0.6468731 -13.5561149
N 5.4209234 6.6581869 -13.7954432
H 6.1313111 7.0377768 -14.4037847
C 4.6727308 7.6690857 -12.8730958
H 3.6722803 7.2716519 -12.7031805
H 5.1665915 7.6425688 -11.902207
C 4.4605241 9.2667223 -13.2399755
O 5.4811072 9.6663177 -13.812121
N 3.2536409 10.1109433 -12.9872607
H 2.6474233 9.6614132 -12.3206553
C 2.3771526 11.4343133 -13.6922496
H 3.1658733 12.0715482 -14.0792353
C 1.0505375 12.8198719 -12.978889
O 0.738086 12.1140384 -12.0071596
C 1.9540762 10.4352165 -14.9544058
H 0.9329988 10.1097298 -14.746017
C 2.7417697 9.1177858 -15.5124699

H 3.7821969 9.3932306 -15.6797871
H 2.6711718 8.3582525 -14.7352443
C 1.9181844 11.302737 -16.1856385
H 2.9290002 11.590086 -16.4899791
H 1.3394983 12.1952761 -15.9637221
H 1.4171187 10.8031861 -17.0150758
C 2.3461044 8.3062146 -16.7841532
H 1.2838958 8.043668 -16.7411571
H 2.9411495 7.3919643 -16.8207085
H 2.5328117 8.8854332 -17.695951
N 0.2333932 14.4003043 -13.1834479
H 0.7437184 14.7653437 -13.9704046
C -1.2123799 15.955379 -12.9597387
H -1.0028175 16.3292374 -13.960482
C -2.5312399 15.1695335 -13.1010222
H -2.3258053 14.1092915 -13.2484796
H -3.0618878 15.2689053 -12.1555772
C -3.4175086 15.6977339 -14.2585728
H -3.2432872 16.7657087 -14.3985759
C -3.0783778 14.9714564 -15.5675978
H -3.2811317 13.903087 -15.4809576
H -2.0269104 15.1037593 -15.8111044
H -3.6757972 15.3801559 -16.3812018
C -4.9110054 15.512951 -13.9555661
H -5.1400087 14.4586235 -13.8201664
H -5.5040244 15.9052164 -14.7807417
H -5.172629 16.0607214 -13.0540348
C -2.0596333 17.9254177 -12.3430272
O -1.3832877 18.2475852 -11.374691
N -3.271554 19.2249776 -12.5340521
H -3.8017849 18.9469697 -13.3433045
C -4.1853409 20.9390259 -11.9780666
H -4.1201797 20.8378689 -10.8941409
C -5.8142116 21.9798425 -12.0685304
O -6.4577917 21.990306 -13.1238488
C -3.3553264 22.2522131 -12.3368714
H -2.7508435 21.9810248 -13.199742
H -3.9431639 23.1195955 -12.6376299
C -2.3935577 22.6639575 -11.2141059
H -1.5875927 23.2551301 -11.6539897
H -1.9417954 21.7737149 -10.7710014
C -3.1027948 23.4921229 -10.1365626
O -4.1338848 23.0043489 -9.6189633
O -2.6283562 24.6174618 -9.8712537
N -6.4475046 23.0719258 -11.2698861
H -5.8681384 23.316696 -10.4654281

C -7.9445496 23.8116708 -11.2481781
H -8.5313975 23.1447577 -11.8786809
C -8.4505552 25.3183528 -11.7282815
O -7.9562339 26.3541144 -11.2608184
C -8.5827225 23.7319299 -9.8142739
H -8.3098143 22.7644516 -9.3910452
H -8.1237068 24.4929663 -9.1820639
C -10.1128287 23.8553926 -9.699581
H -10.4295997 24.8665053 -9.9608487
H -10.3884539 23.7049408 -8.6526486
C -10.8425833 22.8299823 -10.5754585
O -10.9583405 23.1023724 -11.793048
O -11.2574013 21.7859035 -10.0265513
N -9.602907 25.5151302 -12.4609812
H -10.1970189 24.6947314 -12.6038089
C -10.0093518 26.7968804 -13.139427
H -9.6033406 27.6153906 -12.5465371
C -11.5373224 27.0770783 -13.3129338
O -12.3430523 26.1660776 -13.4651874
C -9.3158021 26.7963794 -14.5218177
H -8.2652178 26.5407625 -14.3723088
H -9.7616841 25.9988297 -15.1187812
C -9.3656862 28.1006173 -15.3396433
H -10.3980501 28.3527717 -15.5746801
C -8.7151959 29.2767986 -14.6053596
H -7.6855865 29.0272619 -14.3458923
H -9.2616884 29.5103425 -13.6946444
H -8.721151 30.1580805 -15.2468748
C -8.6202634 27.902131 -16.6598573
H -7.5714266 27.6723762 -16.4704162
H -8.6904418 28.808201 -17.2612677
H -9.0736317 27.0808258 -17.214682
N -11.9412789 28.3572898 -13.4297045
H -11.2467902 29.0828588 -13.3712445
C -13.3321225 28.7589476 -13.708797
H -13.9922764 28.0787705 -13.1669539
C -13.6973559 28.668285 -15.20173
O -13.2212429 29.4599915 -16.0166546
C -13.5976231 30.1744778 -13.1694646
H -13.3339687 30.2032292 -12.1112647
H -12.9526566 30.8857408 -13.6884859
C -15.0439728 30.6091374 -13.3172145
C -16.0007793 30.2075446 -12.3654889
H -15.7026398 29.6095012 -11.5164327
C -17.3497155 30.5734271 -12.5229045
H -18.084102 30.2600018 -11.7942258

C -17.7459906 31.3401123 -13.632773
H -18.784204 31.6156339 -13.7569758
C -16.7930155 31.742311 -14.5848431
H -17.0984245 32.326439 -15.4416514
C -15.4440982 31.3771052 -14.4276294
H -14.7165289 31.6734974 -15.1713742
N -14.6326318 27.780819 -15.5396291
H -14.9521194 27.1150618 -14.8471413
C -15.2390964 27.6886577 -16.8666391
H -15.735075 28.6312197 -17.0994544
H -14.4624243 27.5134405 -17.6111349
C -16.2666536 26.5596064 -16.957317
O -16.3953891 25.7524785 -16.0401148
N -16.9764725 26.4878918 -18.0819378
H -16.8289854 27.176356 -18.8044725
C -17.9575033 25.4408391 -18.3831007
H -17.7899232 24.5827215 -17.7304426
C -17.7792012 24.9927546 -19.8397808
O -17.7959146 25.8188285 -20.750926
C -19.3742105 25.9740925 -18.1133224
H -19.4072767 26.3626889 -17.0943744
H -19.5911057 26.7896238 -18.8053619
C -20.4418095 24.8784784 -18.2588574
H -20.2170614 24.0779836 -17.5490798
H -20.3816661 24.4589508 -19.2655484
C -21.873747 25.3850101 -18.0153021
O -22.0329252 26.4946208 -17.4568844
O -22.8044396 24.6367793 -18.3888393
N -17.5576422 23.6953514 -20.0649298
H -17.6282623 23.0479037 -19.293315
C -17.3269048 23.1256682 -21.396853
H -17.6295227 23.8524687 -22.1539443
C -18.173276 21.8705255 -21.6133325
O -18.4492201 21.1344351 -20.6665456
C -15.8303326 22.833808 -21.5971231
H -15.5031065 22.1074362 -20.8521036
H -15.2664698 23.755845 -21.4523805
C -15.5235885 22.3038595 -22.9769314
N -16.0573017 22.8083599 -24.1656877
C -15.6125519 22.0028993 -25.140073
H -15.854249 22.1181749 -26.1888167
N -14.8449621 21.02673 -24.6277346
H -14.429788 20.2755673 -25.1579691
C -14.7816493 21.1953241 -23.2621255
H -14.2791179 20.561661 -22.5447457
N -18.5185024 21.5818415 -22.8701808

H -18.1432063 22.1738025 -23.6009505
C -19.2288161 20.3661674 -23.257331
H -19.9116933 20.1067755 -22.4457962
C -18.2431347 19.2015468 -23.4437471
O -17.5034608 19.1367934 -24.4310812
C -20.0753688 20.6536823 -24.5101802
H -20.7622502 21.4721288 -24.2876809
H -19.4111275 20.9769363 -25.3134978
C -20.8899068 19.4416545 -25.0031907
H -20.2061397 18.632868 -25.252551
C -21.8900833 18.9305006 -23.9653095
H -22.5664824 19.7301243 -23.6638583
H -21.3716057 18.543429 -23.0898099
H -22.4721546 18.1090986 -24.387707
C -21.6558409 19.8178908 -26.2704486
H -22.3670983 20.6156827 -26.0549008
H -22.1966695 18.9460329 -26.6408658
H -20.9593117 20.149503 -27.0399621
N -18.305586 18.2264973 -22.5363312
H -18.9848484 18.3084584 -21.7879638
C -17.4987295 17.0022879 -22.56464
H -16.4445225 17.2767669 -22.5352472
C -17.7136686 16.1851165 -23.8479637
O -16.7324037 15.7061853 -24.4089253
C -17.8183622 16.1676056 -21.3118248
H -17.6392762 16.7679502 -20.4189096
H -17.1541192 15.3031549 -21.279748
C -19.2469521 15.6902349 -21.2993199
N -20.3555822 16.5291505 -21.1770985
C -21.409785 15.8002387 -21.5780685
H -22.404937 16.1941265 -21.7444035
N -21.0189659 14.5690479 -21.940586
H -21.5461555 13.999855 -22.611493
C -19.6585309 14.4792161 -21.7690455
H -19.0246639 13.6887216 -22.1457658
N -18.9342631 16.1416122 -24.3967885
H -19.7139306 16.4659898 -23.8383305
C -19.2306073 15.4622993 -25.6698397
H -18.9191364 14.4191048 -25.5943322
C -18.4740459 16.0915514 -26.8531044
O -17.994577 15.3816032 -27.732199
C -20.7422481 15.4827005 -25.9605596
H -20.9140981 15.0341016 -26.9402223
H -21.0845436 16.5175317 -26.0055241
C -21.5715667 14.7083567 -24.9337949
O -21.1731487 13.5796127 -24.57134

O -22.5721805 15.2767049 -24.4473625
N -18.2496141 17.4112082 -26.8323886
H -18.5716361 17.9329869 -26.0288096
C -17.4531297 18.1047217 -27.8489587
H -17.7977212 17.8135617 -28.8424323
H -17.5879439 19.1789559 -27.7304308
C -15.956921 17.7942072 -27.7454374
O -15.2888172 17.6280449 -28.7627028
N -15.439025 17.6201509 -26.5247796
H -16.0609943 17.7043393 -25.7294654
C -14.0743246 17.1266898 -26.3031017
H -13.3826248 17.7038566 -26.9186169
C -13.9256189 15.6534948 -26.724003
O -12.9564171 15.2990751 -27.3953963
C -13.702554 17.3422062 -24.8291262
H -13.7609572 18.4063683 -24.6038623
H -14.431762 16.8391893 -24.1953113
C -12.3186415 16.8515113 -24.4543145
C -11.189853 17.6482056 -24.7279804
H -11.3040053 18.609059 -25.2098121
C -9.9051194 17.1911998 -24.3777829
H -9.0312386 17.792005 -24.5808524
C -9.7460546 15.9368061 -23.7535044
O -8.5019852 15.5094553 -23.4137942
H -8.5099345 14.6569228 -22.9779248
C -10.8775819 15.1370117 -23.4808996
H -10.7593813 14.1747477 -23.0085044
C -12.161335 15.5959257 -23.8337612
H -13.0275541 14.9793858 -23.6353157
N -14.9268359 14.8201123 -26.423841
H -15.7014839 15.176822 -25.8741997
C -14.9828394 13.4197593 -26.8408227
H -14.0769896 12.9181517 -26.4986893
C -15.0310294 13.2711907 -28.3685764
O -14.316041 12.4302505 -28.9112801
C -16.1878629 12.7409781 -26.1724631
H -16.0987507 12.8425083 -25.0898935
H -17.1015123 13.2506904 -26.4752072
C -16.3194695 11.2716038 -26.5208461
C -15.679925 10.2960364 -25.7306851
H -15.1296517 10.5903453 -24.8487413
C -15.7427155 8.9385754 -26.1018896
H -15.2470437 8.1817352 -25.5143806
C -16.4327316 8.5582085 -27.2723703
O -16.4601685 7.2539103 -27.6505408
H -16.8895214 7.1591939 -28.5014005

C -17.087672 9.5346431 -28.0532165
H -17.603424 9.2540987 -28.9595907
C -17.035941 10.887789 -27.6716777
H -17.5222426 11.6367727 -28.2851494
N -15.7781968 14.1209507 -29.0785084
H -16.4213335 14.735133 -28.58639
C -15.8302485 14.0991621 -30.5458371
H -16.023302 13.0723458 -30.857743
C -14.4935853 14.5210283 -31.1785911
O -13.967663 13.81329 -32.0333803
C -16.994066 14.9750367 -31.0424011
H -17.90762 14.6865268 -30.519727
H -16.7819786 16.0183928 -30.8031416
C -17.238316 14.8508303 -32.5555673
H -16.3290961 15.1177248 -33.0992785
H -18.0038016 15.5779021 -32.8375267
C -17.6986865 13.4395358 -32.9540066
O -18.9264795 13.2539991 -33.097649
O -16.8225467 12.5548429 -33.0888592
N -13.837982 15.5729289 -30.6701081
H -14.2892367 16.1201696 -29.9440593
C -12.4898729 15.9648231 -31.1351682
H -12.5346458 16.1337827 -32.2113024
C -11.4694612 14.8360737 -30.9194148
O -10.6875397 14.5295424 -31.8238719
C -12.0510367 17.2817301 -30.4618222
H -12.2135606 17.2075124 -29.385962
C -10.5745282 17.6261432 -30.7001145
H -10.360449 17.6415137 -31.7693089
H -9.9326989 16.8899406 -30.2164525
H -10.3471552 18.6032195 -30.2737645
C -12.8768666 18.454303 -31.009272
H -12.6844 18.5843449 -32.0745506
H -12.6143381 19.3701809 -30.4802161
H -13.9403605 18.2682826 -30.8668087
N -11.5418689 14.1369177 -29.7810758
H -12.2068198 14.4377171 -29.0741423
C -10.7457172 12.934077 -29.5229422
H -9.6880299 13.1653715 -29.6509979
H -10.9128238 12.6231638 -28.4919327
C -11.1015855 11.7534031 -30.4368387
O -10.2052046 11.0525872 -30.9091058
N -12.3806552 11.5703557 -30.7665667
H -13.0669852 12.1981408 -30.3596135
C -12.8830576 10.4904933 -31.6335773
H -12.4536585 9.5465298 -31.299618

C -12.4546029 10.6930136 -33.0856704
O -11.8379689 9.7967565 -33.662114
C -14.4115139 10.3664975 -31.5108397
H -14.8837716 11.3195435 -31.7430037
C -15.0075931 9.281508 -32.405127
H -14.497612 8.3332624 -32.2385487
H -14.9184434 9.5764776 -33.4497101
H -16.0681325 9.1738969 -32.1753859
O -14.7191285 9.9990169 -30.1858573
H -14.6611214 10.8041515 -29.6445041
N -12.6061399 11.9050074 -33.6278135
H -13.1277568 12.5991368 -33.0943308
C -12.1108057 12.2899864 -34.96069
H -12.5626445 11.6330904 -35.7039491
C -10.5924574 12.1086371 -35.0606404
O -10.1118234 11.5017037 -36.0194828
C -12.5253388 13.7419652 -35.2846723
H -12.2047179 14.3950538 -34.4716908
C -11.9014053 14.2583629 -36.5896139
H -12.1485647 13.587198 -37.4129321
H -10.8182307 14.3273054 -36.4922998
H -12.2846337 15.2548982 -36.8119629
C -14.0473735 13.8569901 -35.4397281
H -14.390893 13.2481522 -36.2760117
H -14.3288191 14.8958381 -35.6094773
H -14.5551193 13.5168148 -34.5364626
N -9.8327613 12.5183483 -34.0376294
H -10.2691124 13.0158676 -33.2677802
C -8.3866883 12.2947826 -33.9964652
H -7.94803 12.713808 -34.9032965
C -8.0253687 10.7951613 -33.964585
O -7.1750255 10.3535353 -34.7385592
C -7.808095 13.0498299 -32.7940855
H -8.2322251 12.6640796 -31.8665257
H -8.0425363 14.1119177 -32.8768195
H -6.7249933 12.9258191 -32.7693519
N -8.7383063 9.9928143 -33.1690346
H -9.4399586 10.4216383 -32.5757625
C -8.5261026 8.5373236 -33.0519002
H -7.476906 8.3564513 -32.8190178
C -8.836892 7.7959867 -34.3557814
O -8.0721417 6.9214099 -34.7582541
C -9.3671494 7.960609 -31.9009351
H -10.4270834 8.1175921 -32.1029293
C -9.1210937 6.4714297 -31.6602582
H -8.0569718 6.2924769 -31.5037895

H -9.4663804 5.8883818 -32.5134126
H -9.6722286 6.1524028 -30.7752524
O -9.0287859 8.6085484 -30.6970051
H -9.4300372 9.4946216 -30.711502
N -9.8765834 8.2071453 -35.0894348
H -10.4751493 8.9342993 -34.7066042
C -10.218649 7.6484065 -36.4087586
H -10.3078933 6.565189 -36.3149444
C -9.0961985 7.9031619 -37.4291875
O -8.8107229 7.0319327 -38.2457573
C -11.5883493 8.2058925 -36.8724934
H -11.5800876 9.2895272 -36.7418409
C -11.8649231 7.9080697 -38.3605028
H -11.8182208 6.8336763 -38.5433383
H -11.1330206 8.4106419 -38.9928817
H -12.8465962 8.2794081 -38.6492608
C -12.7194695 7.6055139 -36.002287
H -12.8725628 6.5606242 -36.275951
H -12.4291906 7.6247014 -34.9527788
C -14.0547499 8.3529914 -36.1158443
H -14.469557 8.2558855 -37.1180749
H -13.9111854 9.4081356 -35.8800284
H -14.765007 7.9268607 -35.4065953
N -8.3937191 9.0388911 -37.3367375
H -8.6846259 9.7176446 -36.6427142
C -7.265239 9.3812153 -38.2229573
H -7.5533014 9.1179589 -39.2408252
C -5.9497281 8.6286735 -37.9283472
O -4.9741865 8.7465544 -38.6840646
C -7.0390962 10.899473 -38.1899562
H -8.0014138 11.4136505 -38.2093944
H -6.4800146 11.1924024 -39.0771632
O -6.2990547 11.3175345 -37.05805
H -6.6658562 10.914714 -36.2473093
N -5.8900761 7.8921141 -36.8100548
H -6.7249579 7.8243468 -36.2397125
C -4.6565738 7.3302429 -36.256341
H -3.8362244 8.0007177 -36.5123651
C -4.2800623 5.9408626 -36.8074929
O -3.1352983 5.5205919 -36.6491747
C -4.7709993 7.3240925 -34.7269169
H -5.5236596 6.6026775 -34.4107831
H -5.0474193 8.3167635 -34.3677705
H -3.8122191 7.0434425 -34.2890576
N -5.1800992 5.2659515 -37.5310566
H -6.0659794 5.7270073 -37.7010793

C -4.9863126 3.9392569 -38.1512939
H -5.0038412 3.1815968 -37.3671395
C -3.6467069 3.7571432 -38.8886477
O -2.9731921 2.7386014 -38.7264792
C -6.1697208 3.6740353 -39.1048049
H -7.0402153 3.4328757 -38.4935022
H -5.9499646 2.7970377 -39.7150907
C -6.5637775 4.8197352 -40.0201111
N -5.7699764 5.9207787 -40.3641479
C -6.5674691 6.752385 -41.049963
H -6.277414 7.7318812 -41.4028678
N -7.7926363 6.2244398 -41.1826337
H -8.5854946 6.6964794 -41.5940588
C -7.8129371 5.0107498 -40.5332027
H -8.6720512 4.3747877 -40.3736965
N -3.1845526 4.7824287 -39.6094995
H -3.8397485 5.5490973 -39.7402001
C -1.9094941 4.7790629 -40.3503428
H -1.9058746 3.9210138 -41.0259016
C -0.6755178 4.6000095 -39.4590681
O 0.3500762 4.1523355 -39.9634635
C -1.8108003 6.0655976 -41.1818839
H -1.9802055 6.9149351 -40.5182176
H -0.8101594 6.155225 -41.6088882
C -2.8366963 6.0737128 -42.3295833
H -3.7805566 5.6467642 -41.9998432
H -2.4675716 5.4691415 -43.158521
C -3.090666 7.5062857 -42.7975055
H -2.21557 7.8834898 -43.328123
H -3.2604295 8.1357064 -41.9227019
N -4.2898586 7.6146451 -43.6353146
H -5.0985667 8.0752973 -43.2163844
C -4.3632322 7.5927761 -44.9465972
N -3.4118636 7.0991039 -45.680707
H -3.2172373 7.5904414 -46.5573116
H -2.6560279 6.644648 -45.2205878
N -5.4028833 8.1065841 -45.5234574
H -6.0523492 8.6180683 -44.9202338
H -5.4423454 8.1830156 -46.5185052
N -0.7546542 4.8641941 -38.1528525
H -1.6478001 5.1514024 -37.7665882
C 0.3441051 4.6164829 -37.2169392
H 1.2323201 5.1264964 -37.5885598
C 0.6996833 3.1210416 -37.0906775
O 1.8637562 2.7984179 -36.8587498
C -0.0133202 5.2297426 -35.858726

H -0.9017486 4.748926 -35.4486878
H -0.2065543 6.2971976 -35.9704315
H 0.8169899 5.0909097 -35.1647239
N -0.2423564 2.2117153 -37.3765593
H -1.1831707 2.5200405 -37.5942453
C 0.0441412 0.7786215 -37.4595675
H 0.5673747 0.4724402 -36.5515695
C 0.9552045 0.4341165 -38.6550826
O 1.7906982 -0.4590169 -38.5465271
C -1.2872208 0.0221727 -37.522223
H -1.8167315 0.2672544 -38.4447155
H -1.9079066 0.2944651 -36.668599
H -1.1024213 -1.0504495 -37.4916568
N 0.8781965 1.1977947 -39.7544587
H 0.2102942 1.9555281 -39.7611379
C 1.8112123 1.0873253 -40.8905783
H 2.0039892 0.0301488 -41.0804667
C 3.1532747 1.7347408 -40.5479506
O 4.1918602 1.1211358 -40.7786454
C 1.2242149 1.7004207 -42.1796402
H 1.0712918 2.7691655 -42.0362597
C 2.1680245 1.5163423 -43.3761676
H 2.3959256 0.458749 -43.5151264
H 3.0968456 2.0616945 -43.2111974
H 1.7047064 1.9050683 -44.2829471
C -0.1270984 1.0684292 -42.5415657
H -0.0134202 -0.0094525 -42.6618157
H -0.4995357 1.4948188 -43.4727939
H -0.8607535 1.2658962 -41.7604837
N 3.1497115 2.9075864 -39.8983128
H 2.2584563 3.3646896 -39.7477886
C 4.3829764 3.5769137 -39.4271644
H 5.0316617 3.7532182 -40.287047
C 5.1714679 2.6870662 -38.4572727
O 6.3914124 2.6466122 -38.5342007
C 4.0854947 4.9447377 -38.7734971
H 3.4492565 4.7868126 -37.9038288
C 5.3579813 5.667574 -38.3077099
H 6.0473034 5.7700222 -39.1479344
H 5.8520541 5.0988091 -37.5198768
H 5.1091859 6.6515776 -37.9116408
C 3.3657123 5.9094878 -39.7292705
H 4.0142422 6.146524 -40.5715909
H 3.1117469 6.8290265 -39.2030367
H 2.4524687 5.4645623 -40.1099666
N 4.5099699 1.8691594 -37.6261136

H 3.501018 1.9661057 -37.5679173
C 5.1768327 0.8861925 -36.7517641
H 5.9189905 1.4157616 -36.1524491
C 5.9655105 -0.1805465 -37.5278367
O 6.9655235 -0.6580613 -37.005066
C 4.1151826 0.2654933 -35.821984
H 3.6872668 1.0584642 -35.2070059
H 3.3115988 -0.1409395 -36.4366679
C 4.6026419 -0.8663175 -34.8987054
H 4.9701494 -1.6946528 -35.5041773
H 3.7425868 -1.2253198 -34.3369632
C 5.691348 -0.4487287 -33.8979481
H 5.305648 0.3329929 -33.2411631
H 6.5347315 -0.0299299 -34.4515579
N 6.1852786 -1.5988657 -33.1065998
H 7.0302672 -2.023504 -33.4565587
C 5.6931212 -2.0929878 -31.9796119
N 4.6137617 -1.6238221 -31.4121932
H 4.2496792 -2.0315972 -30.5644291
H 4.1500759 -0.8388203 -31.8268032
N 6.2826626 -3.0960025 -31.392016
H 7.158683 -3.4460252 -31.7437805
H 5.9220268 -3.4384356 -30.5210906
N 5.5130667 -0.5786026 -38.7196586
H 4.6989518 -0.1098716 -39.0946508
C 6.155844 -1.6215128 -39.5477068
H 6.7171868 -2.2989085 -38.9044692
C 7.1870424 -1.0471198 -40.5141252
O 8.1672071 -1.7115576 -40.8379074
C 5.0729423 -2.429844 -40.2970744
H 4.4719049 -1.7392024 -40.8919679
C 5.6505611 -3.4778044 -41.2668926
H 6.3652394 -4.1194898 -40.7503349
H 6.1577817 -2.9777053 -42.0922651
H 4.8537515 -4.0898952 -41.6888069
C 4.1272498 -3.1381766 -39.3001663
H 3.4521743 -2.3981917 -38.8745679
H 3.519856 -3.8567523 -39.8472324
C 4.8022508 -3.8790266 -38.1377464
H 5.1620025 -3.1674568 -37.3956499
H 5.6444629 -4.4611102 -38.5038849
H 4.0797422 -4.5405924 -37.6627072
N 6.9634562 0.1827065 -40.9660073
H 6.1059518 0.645179 -40.6903566
C 7.8918005 0.9019409 -41.8307362
H 8.3560989 0.1968688 -42.5199967

C 9.0541694 1.5083209 -41.0392823
O 10.1751688 1.4175308 -41.5113396
C 7.0910835 1.9372281 -42.6425295
H 6.4755174 2.5141194 -41.9494156
C 8.015055 2.9405889 -43.3353853
H 8.8170607 2.4130698 -43.8517664
H 8.4436482 3.6079035 -42.5859543
H 7.4537262 3.5413332 -44.0376991
C 6.1329575 1.2547909 -43.6511673
H 5.3388 0.7519175 -43.1009127
H 5.6559528 2.0240523 -44.2518133
C 6.7607953 0.2206656 -44.5975052
H 7.0017951 -0.6915304 -44.0477814
H 7.6692296 0.6123557 -45.0487245
H 6.0481605 -0.0291608 -45.3837918
N 8.798992 1.9819869 -39.8195457
H 7.8269675 1.9509433 -39.5448266
C 9.6149082 2.8267209 -38.9312898
H 9.2170332 2.5797365 -37.9459199
C 9.3250434 4.3409519 -39.0306445
O 8.5238253 4.7993794 -39.8542185
C 11.099113 2.4020543 -38.8279642
H 11.7082873 3.1408157 -39.3434734
H 11.2510327 1.4269978 -39.2904088
C 11.5965131 2.3038259 -37.3833269
O 11.4492724 3.3205405 -36.6703128
O 12.1249463 1.2330349 -37.0094698
N 9.8588958 5.1058401 -38.0754761
H 10.516748 4.6466534 -37.4396315
C 9.3325992 6.4041998 -37.6512809
H 8.3526531 6.2042132 -37.2199303
C 9.1395831 7.4234129 -38.7896545
O 10.0494829 7.7492656 -39.5515223
C 10.2024572 6.9760756 -36.5200309
H 10.3373002 6.2059069 -35.7593743
H 11.1814432 7.2466722 -36.9189787
C 9.5601829 8.2096529 -35.8657287
H 8.5933201 7.9247972 -35.443284
H 9.3792816 8.9717041 -36.6261681
C 10.4478246 8.8008571 -34.7647909
O 10.8020372 8.0456266 -33.8314376
O 10.7428137 10.012847 -34.8410345
N 7.9356494 8.0039627 -38.8202964
H 7.2747294 7.6949909 -38.1276181
C 7.4979327 9.066309 -39.7246913
H 6.4475177 9.2219085 -39.4784392

C 7.4607978 8.7250306 -41.2328113
O 7.2182063 9.6195186 -42.0501456
C 8.189382 10.3821855 -39.339387
H 9.2533861 10.3291555 -39.5703739
H 8.0772996 10.5585665 -38.2684137
H 7.7299772 11.2135753 -39.8729389
N 7.5456185 7.4423073 -41.6155564
H 7.7541 6.7354562 -40.9135248
C 7.344726 6.9801426 -42.997353
H 6.9802142 7.8289962 -43.5714683
C 6.2228111 5.9398072 -43.1423331
O 5.8136015 5.298277 -42.1811172
C 8.7040589 6.5417867 -43.5937623
H 9.3769214 6.2789443 -42.781539
H 8.5857406 5.6690598 -44.2357264
C 9.2911549 7.7100855 -44.425368
H 9.1907816 8.6410518 -43.8675988
C 10.7676023 7.5379856 -44.7641775
H 10.9094523 6.6899523 -45.4326336
H 11.347273 7.4053009 -43.8535032
H 11.1408564 8.4293545 -45.2677901
C 8.5546316 7.8484516 -45.7653518
H 8.6226347 6.9085588 -46.3141622
H 9.0244539 8.6267692 -46.3653165
H 7.5122898 8.121164 -45.6183874
N 5.6747873 5.8134553 -44.3622881
H 6.051826 6.3868802 -45.1032308
C 4.5891328 4.8649351 -44.6980872
H 4.5631062 4.1139725 -43.9051263
C 4.8447935 4.0459821 -45.973334
O 3.9017478 3.6498304 -46.6520897
C 3.2105171 5.5603428 -44.666108
H 2.4533252 4.7738203 -44.6831793
H 3.1054279 6.0864467 -43.7153432
C 2.9161759 6.5454592 -45.8179066
H 3.1261552 6.0697121 -46.7735315
H 3.5448542 7.4315362 -45.7241825
C 1.4308664 6.9484155 -45.7776237
H 1.2590212 7.5693356 -44.8966521
H 0.8212512 6.0482975 -45.6740764
N 0.981203 7.6811794 -46.9780385
H 0.7524881 8.6673509 -46.8923419
C 0.6228584 7.177358 -48.1443321
N 0.8186153 5.929421 -48.4685675
H 0.5752193 5.6132175 -49.3870961
H 1.388143 5.3577715 -47.8677134

N 0.0406873 7.949872 -49.0092621
H -0.2933387 8.855375 -48.6610897
H -0.2623347 7.6163466 -49.9005672
N 6.1190758 3.8578277 -46.3209588
H 6.8301691 4.1363819 -45.6635574
C 6.5958218 3.2428633 -47.5626859
H 5.830005 2.5719029 -47.9542416
C 7.8916732 2.4367347 -47.343168
O 8.5668267 2.64766 -46.3401455
C 6.8674228 4.3491416 -48.5851797
H 7.7970464 4.8464069 -48.304794
H 7.032437 3.8770175 -49.5533758
C 5.793543 5.4093771 -48.7466088
C 4.5694425 5.071977 -49.3430479
H 4.3617759 4.0434651 -49.5976387
C 3.6563468 6.0786253 -49.6922932
H 2.7837729 5.8310304 -50.2772817
C 3.9157208 7.4151039 -49.3467661
H 3.2349086 8.196869 -49.6514519
C 5.0976506 7.7468283 -48.6644446
H 5.3156042 8.7854837 -48.4512446
C 6.0470079 6.7471097 -48.3865945
H 6.9997683 7.0245668 -47.966154
N 8.2414517 1.5676536 -48.3001291
H 7.6597218 1.5224626 -49.118932
C 9.3979198 0.6578697 -48.2914649
H 9.1016447 -0.2646165 -47.791358
C 10.6602547 1.1969945 -47.5742744
O 11.1786285 2.245083 -47.9427162
C 9.7087369 0.3224341 -49.75895
H 9.967396 1.234773 -50.3010873
H 8.8483582 -0.1504811 -50.2328802
H 10.5608699 -0.3531544 -49.8204047
N 11.1058984 0.4683517 -46.5404187
H 10.6020477 -0.3756197 -46.3181145
C 12.3498292 0.6411544 -45.7620579
H 12.0842348 0.2321049 -44.78769
C 12.7677959 2.0582833 -45.3295639
O 13.9257997 2.2966503 -44.965395
C 13.488643 -0.2542563 -46.2966031
H 14.4106292 0.3125754 -46.3885813
H 13.233996 -0.5667301 -47.3062372
C 13.7404825 -1.5012609 -45.4212878
O 12.782663 -1.9446545 -44.7479203
O 14.8558588 -2.0616043 -45.4835954
N 11.7676098 2.9221305 -45.1175289

H 10.8663841 2.6705479 -45.5032949
C 11.8434355 4.1137479 -44.2565654
H 10.9702733 4.7131008 -44.4953178
C 13.0474841 5.0013958 -44.5690478
O 13.2566867 5.4146228 -45.7033659
C 11.6839069 3.7215239 -42.7654516
H 10.7042933 3.2772 -42.7433744
C 12.7172875 2.6995277 -42.243958
H 13.7411237 3.021697 -42.4103113
H 12.5627012 1.7344082 -42.7242798
H 12.5786212 2.5252549 -41.1832717
C 11.6078686 4.8907969 -41.7666777
H 12.5088229 4.963613 -41.1569353
H 10.7748965 4.7152793 -41.0967139
H 11.4403098 5.837781 -42.2673589
N 13.7997814 5.3551081 -43.5357305
H 13.5427582 4.9757027 -42.6392564
C 14.9722896 6.1999847 -43.5773004
H 14.7513394 7.1316738 -44.1038787
C 16.1325397 5.5277022 -44.3124657
O 16.971215 6.2121722 -44.8791594
C 15.3363167 6.5059358 -42.1167684
H 14.5993989 7.1982355 -41.7073855
H 15.2826306 5.5845827 -41.5328418
C 16.7149966 7.0988042 -41.9408267
C 16.9780411 8.3984896 -42.403626
H 16.1908624 8.9809401 -42.8654804
C 17.7536204 6.3218351 -41.3924162
H 17.5600729 5.3123153 -41.0611771
C 18.2759221 8.9240865 -42.299878
H 18.4802673 9.9218835 -42.6403416
C 19.0546992 6.8452967 -41.3078177
H 19.8555147 6.2433117 -40.90308
C 19.314826 8.149128 -41.7601691
H 20.3159865 8.5514257 -41.6963891
N 16.2058513 4.196207 -44.3268838
H 15.3924863 3.6449569 -44.0809636
C 17.3365519 3.5317345 -44.9880782
H 18.247824 4.0148928 -44.6328091
C 17.3362498 3.7162578 -46.5202865
O 18.411362 3.6889561 -47.1212283
C 17.4139431 2.0551039 -44.544089
H 17.2640812 2.0186564 -43.4640312
C 18.7685605 1.4097662 -44.8475106
H 18.7772956 0.3945679 -44.4540022
H 19.5699411 1.9849547 -44.3903993

H 18.9200973 1.3641078 -45.9264033
O 16.4573901 1.2286298 -45.1599023
H 15.5577683 1.5969757 -45.0914068
N 16.1818698 4.0690221 -47.1078517
H 15.3465728 4.1192239 -46.5422669
C 16.0387294 4.3659216 -48.539581
H 16.9320402 3.9392428 -48.9945931
C 16.0907108 5.8966401 -49.0803814
O 16.0794458 5.9767522 -50.2908172
C 14.8556147 3.4957892 -49.0773819
H 13.9798023 4.1366509 -49.1933825
H 14.5968292 2.7346661 -48.3378472
C 15.1275989 2.7525254 -50.4024108
O 14.1400315 2.2286328 -50.965854
O 16.307982 2.6299838 -50.8096799
N 16.2900343 7.1238414 -48.4203981
H 16.4497442 7.0131143 -47.4277553
C 16.1613041 8.688626 -48.8951202
H 15.5643498 8.6270493 -49.8051702
C 17.4543756 9.8556596 -49.3389581
O 18.4787587 9.2741032 -49.0634247
C 15.1839224 9.2297703 -47.7945255
H 14.6070166 8.4267525 -47.3302822
H 14.4664933 9.8869038 -48.2885934
C 15.9020718 10.0680588 -46.713811
O 16.3872989 11.1886415 -47.009515
O 15.9809798 9.6471257 -45.537687
N 17.6582507 11.2710311 -50.0187799
H 16.7432841 11.6473397 -50.215925
C 18.9196021 12.4999217 -50.4996121
H 19.4239745 12.7072473 -49.557024
C 19.8910586 11.6284874 -51.3214552
H 19.3040143 10.926469 -51.916006
H 20.4598406 12.2416738 -52.0211191
C 20.8996192 10.8577388 -50.4511839
H 20.4908512 10.6758339 -49.4595413
H 21.799341 11.4600084 -50.3269145
C 21.2793501 9.5187183 -51.0747255
O 22.2065281 9.4215954 -51.8603748
N 20.5592735 8.459898 -50.7803827
H 20.794535 7.5910496 -51.2218482
H 19.7724068 8.5434667 -50.1355936
C 19.092366 14.182846 -51.3913657
O 18.0971829 14.350783 -52.0856275
N 20.1085432 15.3856543 -51.5493382
H 20.9260687 15.212857 -50.986138

C 20.2948739 16.9461427 -52.3131094
H 20.2227503 16.7196393 -53.3770916
C 21.4078328 18.3179152 -52.2406162
O 22.5468109 18.183726 -51.7988277
C 19.0686253 17.7464106 -51.9697146
H 19.2338989 18.2728335 -51.0391949
H 18.2005998 17.0932304 -51.9204808
H 18.8667864 18.4802587 -52.7438545
N 21.1702238 19.6749261 -52.6514902
H 20.2345851 19.8379585 -52.9935248
C 22.0024176 21.0115075 -52.4479166
H 22.8683875 20.7431257 -51.8441611
C 21.2832516 22.1579803 -51.6588977
O 20.098186 22.3880363 -51.8856444
C 22.5113401 21.7661131 -53.7349204
H 21.6390627 22.0773934 -54.3111731
H 23.0283835 22.6774737 -53.4283394
C 23.4507222 21.0203275 -54.6889031
H 22.9115143 20.1707727 -55.1106251
H 23.7236569 21.6915994 -55.5045992
C 24.7266258 20.5101083 -54.0066815
H 24.4530278 19.8220364 -53.2042071
H 25.2791937 21.3544152 -53.5879837
C 25.5984771 19.7681775 -55.0268131
H 25.909443 20.4728439 -55.8051529
H 24.9888066 18.9920389 -55.5006992
N 26.7854235 19.1515691 -54.3842775
H 27.3653918 19.8580064 -53.9512649
H 27.3408378 18.6543916 -55.0695705
H 26.4940335 18.4923601 -53.6723818
N 22.0141776 22.9790716 -50.8776924
H 22.9848334 22.7561847 -50.7481297
C 21.4363151 24.0595014 -50.0406279
H 20.6651534 23.5880877 -49.4217957
C 20.6932301 25.1571828 -50.8434591
O 21.1123859 25.4928957 -51.9501055
C 22.4954675 24.6021902 -49.0576117
H 21.9681595 25.0697176 -48.2281703
H 23.0446883 23.7611733 -48.6298199
C 23.4967813 25.6136439 -49.6395768
H 24.1308012 25.1185841 -50.3761876
H 22.9616084 26.426131 -50.1294037
C 24.3600993 26.2058559 -48.5122502
H 25.0695733 25.4548231 -48.159759
H 23.7129745 26.4715583 -47.6719088
C 25.1007745 27.47991 -48.9414417

H 25.6487684 27.8643149 -48.0751497
H 24.3538345 28.231996 -49.2207631
N 26.033051 27.2429212 -50.0717632
H 26.729265 26.5557402 -49.8134378
H 26.5058074 28.1019389 -50.3270792
H 25.5216444 26.9054137 -50.8778729
N 19.6653497 25.7902966 -50.2502701
C 18.5260935 26.4199977 -50.9302971
H 17.683172 26.2611953 -50.25573
C 17.9666354 25.9230463 -52.2825018
O 17.2120042 26.6858309 -52.8855268
C 18.8245867 27.9163968 -50.8994079
H 19.6064095 28.1600803 -51.6210341
H 17.9311673 28.5198536 -51.0658878
C 19.9982801 26.7186724 -49.1631419
H 19.6497361 26.3469128 -48.204326
H 21.0706298 26.8855675 -49.103357
C 19.3411503 28.0678737 -49.4726293
H 18.5032263 28.2293829 -48.7937525
H 20.0551531 28.8885143 -49.395854
N 18.1444302 24.6585658 -52.7170806
H 18.7575415 24.0313991 -52.2081714
C 17.2497154 24.0944877 -53.7603078
H 17.1809009 24.8305569 -54.5644565
C 15.8108259 23.9536811 -53.2184155
O 15.6116559 23.7733122 -52.0168994
C 17.7905208 22.7718561 -54.367871
H 18.2164828 22.1394345 -53.5915213
H 16.9504542 22.2274091 -54.799801
C 18.8275157 22.9966047 -55.490398
H 19.7490875 23.3914824 -55.0618205
H 18.4292468 23.7618966 -56.1540239
C 19.1471149 21.7322105 -56.331149
H 18.3107319 21.0347568 -56.2604309
H 20.0375569 21.2460875 -55.9308658
N 19.3493069 22.0762304 -57.7577649
H 19.2235844 23.0639289 -58.0004847
C 19.3902273 21.3013719 -58.8329725
N 19.5688706 20.0105413 -58.789513
H 19.5868354 19.4947518 -59.6476506
H 19.6328429 19.5190873 -57.9186205
N 19.2411043 21.8219077 -60.01574
H 18.7989704 22.7503794 -60.07748
H 19.2658129 21.2691169 -60.8598066
N 14.821947 23.9635607 -54.1171727
H 15.0640462 24.1295175 -55.0795782

C 13.4070457 23.7053603 -53.8023649
H 13.2318405 23.8314889 -52.7303655
C 13.0148305 22.2690653 -54.1901151
O 13.2650034 21.8554559 -55.3268438
C 12.5042714 24.70102 -54.554355
H 11.4657565 24.4018961 -54.4176821
H 12.7363243 24.6603459 -55.6166903
C 12.5918951 26.14774 -54.1111584
O 13.1869008 26.5106661 -53.1113018
N 11.9857542 27.0281791 -54.8750885
H 12.0663734 27.990201 -54.6088372
H 11.474667 26.7304553 -55.7012412
N 12.3104354 21.5618859 -53.3039725
H 12.1319232 21.9882772 -52.3959269
C 11.7798987 20.2088839 -53.5218785
H 12.1354009 19.8236533 -54.4750295
C 10.2499869 20.2006078 -53.5304971
O 9.636071 20.6928764 -52.5837385
C 12.2939867 19.2814334 -52.4104324
H 12.0003572 19.7111831 -51.4550013
H 13.3811827 19.2399207 -52.459261
C 11.7331357 17.8531895 -52.451972
H 10.6473428 17.8812916 -52.5280063
H 11.9562163 17.3702826 -51.5054196
S 12.3827862 16.813984 -53.7779941
C 13.8931229 16.2105975 -52.9728887
H 14.4627188 15.602152 -53.6752431
H 13.6285852 15.6018071 -52.1075691
H 14.5036321 17.0516099 -52.6454799
N 9.6561252 19.476022 -54.4810838
H 10.2356146 19.1014098 -55.228018
C 8.2618632 19.0294284 -54.4110718
H 7.7432147 19.5950109 -53.6411374
C 8.2012683 17.5367186 -54.0391612
O 8.6091906 16.6916482 -54.8315145
C 7.5848191 19.3377223 -55.7563497
H 7.6533027 20.404727 -55.9472126
H 8.1407942 18.8235216 -56.5354205
C 6.1094319 18.9161937 -55.8471269
H 6.0223168 17.8621598 -55.5987058
C 5.2008171 19.7105303 -54.9111302
H 5.2842959 20.7773402 -55.1160026
H 5.4789999 19.5191252 -53.8783082
H 4.1706631 19.3882301 -55.0433692
C 5.5958432 19.1157678 -57.2735893
H 5.6369121 20.1715894 -57.5396105

H 4.5696247 18.7588489 -57.3505647
H 6.2159663 18.5490014 -57.9673902
N 7.650806 17.207511 -52.8705797
H 7.2809473 17.9542867 -52.2913677
C 7.3710433 15.8279812 -52.4464654
H 8.0060151 15.1508264 -53.0126345
C 5.9027388 15.4793458 -52.7611271
O 4.9709185 16.075372 -52.2065813
C 7.75986 15.6405981 -50.9667627
H 7.1203425 16.2573012 -50.3370099
H 8.7862314 15.9905422 -50.8370111
C 7.6824443 14.1863395 -50.4760692
O 6.8708591 13.4090086 -51.019936
O 8.4087115 13.8653462 -49.5055914
N 5.6864177 14.5711102 -53.7189983
H 6.4974395 14.0713565 -54.0777662
C 4.3447916 14.1842873 -54.1843736
H 3.6345714 14.9349283 -53.8406842
C 3.9351687 12.8544228 -53.5605881
O 4.3232699 11.7831128 -54.0192536
C 4.2336867 14.1575708 -55.7201635
H 4.8568936 13.3737071 -56.1278179
C 2.7974381 13.8629456 -56.1479142
H 2.1426664 14.6095088 -55.703318
H 2.4969621 12.8683791 -55.8186253
H 2.7229068 13.8922377 -57.2343704
C 4.6461988 15.4961607 -56.3443168
H 4.0765308 16.3124174 -55.897042
H 4.4772019 15.482627 -57.4198195
H 5.7094427 15.6583488 -56.1699027
N 3.0257182 12.9205146 -52.5909151
H 2.717312 13.8404901 -52.2952782
C 2.6511322 11.7941466 -51.7378099
H 1.5919382 11.8736081 -51.495824
H 2.826334 10.8515972 -52.2505211
C 3.4378837 11.764253 -50.4292475
O 3.7783718 10.6859284 -49.9559292
N 3.6725557 12.9262538 -49.8125931
H 3.3218807 13.7634739 -50.2589932
C 4.5639542 13.1282033 -48.6574106
H 5.5763021 12.8435635 -48.9560907
C 4.2394702 12.317265 -47.379758
O 4.9944892 12.3378076 -46.4012804
C 4.557135 14.6320349 -48.3562011
H 4.9178299 15.1702423 -49.2356233
H 5.2327209 14.8285762 -47.5267004

S 2.8894722 15.2074225 -47.9074698
H 2.7061254 14.4032963 -46.8479299
N 3.0995178 11.6227276 -47.3420058
H 2.5537956 11.6005599 -48.1895828
C 2.6973692 10.7695608 -46.2284903
H 1.7296098 10.3306215 -46.4382888
H 3.4252333 9.964224 -46.1301078
C 2.5964225 11.5335775 -44.9096616
O 2.0756425 12.6458024 -44.8617237
N 3.1475683 10.9405853 -43.8409104
H 3.5995258 10.050913 -43.9813798
C 3.2644505 11.5676352 -42.5110625
H 2.5363332 12.3721322 -42.4922009
C 4.6024855 12.3215117 -42.2989558
O 4.9330438 12.7035656 -41.1837733
C 2.7685976 10.6219858 -41.3876643
H 3.329892 9.6866489 -41.3966698
H 2.9110664 11.1133101 -40.4234859
C 1.2482459 10.3497499 -41.5881163
H 0.7919812 11.3134804 -41.7977461
H 1.1127473 9.7337692 -42.4784474
C 0.4227669 9.7161131 -40.4391496
H 0.7314226 10.1512193 -39.4901884
H 0.5960085 8.639396 -40.4025918
C -1.0758004 10.0217816 -40.7095582
H -1.41602 9.4220592 -41.5561837
H -1.1388727 11.0716698 -41.0127608
N -2.0107233 9.8625097 -39.5598994
H -2.0996365 8.9127489 -39.2334567
H -2.9406933 10.1949484 -39.8135329
H -1.7309402 10.4529809 -38.7839952
N 5.3030928 12.6456133 -43.3932679
H 4.9509657 12.3158047 -44.2839729
C 6.263606 13.7539353 -43.4685514
H 6.339384 14.0373157 -44.5168193
H 5.8664254 14.6025745 -42.9108326
C 7.6966603 13.5217271 -42.9844836
O 8.5229441 14.4189115 -43.1446022
N 8.052298 12.3272621 -42.5032093
H 7.3432049 11.6119669 -42.3818921
C 9.4128024 12.0200965 -42.040962
H 9.6646061 12.6654394 -41.1981862
H 9.4474178 10.9834761 -41.7123813
C 10.481707 12.1834795 -43.1192423
O 11.5222019 12.7773601 -42.8556136
N 10.1750316 11.7843984 -44.3589289

H 9.2846575 11.3332788 -44.4955374
C 11.0186339 12.0413754 -45.5425943
H 11.9980506 11.588156 -45.3790969
C 11.2701527 13.5389522 -45.7486745
O 12.4236188 13.9518113 -45.8260238
C 10.4258214 11.3832015 -46.8083101
H 10.5905208 10.3099959 -46.7269139
C 8.9312833 11.6033541 -47.0463739
H 8.7099762 12.6632397 -47.1442538
H 8.3393666 11.1827496 -46.2344072
H 8.6361123 11.1131843 -47.9758533
O 11.0895781 11.8306891 -47.9597574
H 10.484578 12.3844576 -48.4701324
N 10.2252136 14.371186 -45.663101
H 9.3074535 13.9722939 -45.5446904
C 10.2857366 15.8236971 -45.8547703
H 10.7235008 16.0265557 -46.8268567
C 11.1106349 16.537419 -44.7650356
O 11.8863183 17.4526382 -45.0535196
C 8.8657689 16.3970441 -45.8856242
H 8.8986116 17.4030429 -46.3069571
H 8.4867539 16.4648006 -44.8668808
S 7.7163401 15.3759861 -46.8510702
H 8.4533831 15.1086649 -47.9512639
N 10.9632892 16.0999518 -43.508704
H 10.2825801 15.3646959 -43.3383485
C 11.7666912 16.5933009 -42.3777083
H 11.7210304 17.682167 -42.3558897
C 13.2337723 16.2149077 -42.5646649
O 14.1041332 17.0794444 -42.4631432
C 11.2181531 16.0596538 -41.0385742
H 11.0788588 14.9803609 -41.1088303
C 12.1583111 16.3454275 -39.8608403
H 12.413162 17.4044263 -39.8365978
H 13.0713173 15.7583224 -39.9544092
H 11.6747709 16.0658247 -38.9240883
C 9.8619149 16.7101419 -40.7311641
H 9.9738266 17.7906836 -40.6408162
H 9.465485 16.313601 -39.7953368
H 9.147258 16.4904463 -41.5236473
N 13.5086787 14.9554922 -42.9189931
H 12.7374487 14.300883 -43.0121126
C 14.8616324 14.4600437 -43.1763215
H 15.4605874 14.5795315 -42.2714439
C 15.5717593 15.257738 -44.2626702
O 16.6984701 15.6980688 -44.0520232

C 14.8119003 12.9696725 -43.5454865
H 15.47017 12.778969 -44.3885111
H 13.8159203 12.6800994 -43.8734669
C 15.2350372 12.1081566 -42.3887418
N 16.5503927 11.9810507 -41.9354251
C 16.4753185 11.2196844 -40.8299497
H 17.3253826 10.9192111 -40.2306378
N 15.2029599 10.8627996 -40.5836198
H 14.890693 10.2906702 -39.8085624
C 14.4037633 11.4233475 -41.5538219
H 13.3257702 11.3738953 -41.6274226
N 14.9078435 15.5075468 -45.387301
H 13.989292 15.091668 -45.5152898
C 15.5112271 16.2350314 -46.5011762
H 16.4944499 15.8005517 -46.6810838
C 15.788866 17.702243 -46.1455768
O 16.9395239 18.1374501 -46.2012692
C 14.6897345 16.0179734 -47.7882799
H 15.1327128 16.6236197 -48.5603665
C 14.7908711 14.5234165 -48.182398
H 14.1962195 13.8987777 -47.5198436
H 15.8220997 14.1781548 -48.1262874
H 14.4372692 14.3716629 -49.2011021
C 13.2366266 16.4975189 -47.7320752
H 12.7646062 16.0195718 -46.8976035
H 13.2059957 17.5711979 -47.5585925
C 12.4407379 16.1837724 -49.0033466
H 12.2244151 15.1164961 -49.062049
H 13.0189562 16.48687 -49.8741816
H 11.4970485 16.7230964 -48.9888032
N 14.8187234 18.4245126 -45.5793613
H 13.9008259 18.0120409 -45.4473034
C 15.0314858 19.8172963 -45.1857533
H 15.4248899 20.3646793 -46.0422485
C 16.0654803 19.9815299 -44.052507
O 16.775709 20.9872676 -44.0197683
C 13.6739593 20.4215669 -44.8288207
H 13.2487701 19.8884107 -43.9775321
H 12.9997992 20.3346726 -45.682005
H 13.7950387 21.4777533 -44.588073
N 16.1945128 19.0031707 -43.1417661
H 15.5550477 18.2125874 -43.1960906
C 17.1426889 19.0652184 -42.0165262
H 17.2682905 20.115006 -41.7540704
C 18.5555244 18.5806105 -42.3633946
O 19.5022617 19.2022987 -41.8920977

C 16.5356863 18.3408313 -40.8014493
H 15.5546198 18.7709715 -40.5929376
H 16.3868088 17.2899072 -41.0560764
C 17.3783728 18.4041283 -39.5137768
H 18.2761985 17.8007418 -39.655826
H 16.803368 17.9420041 -38.7110996
C 17.8159365 19.8120997 -39.0658109
H 18.4907804 20.2290409 -39.816017
H 18.3932875 19.7217441 -38.1440225
N 16.6897875 20.7491832 -38.8609668
H 16.5665419 21.4502419 -39.5716769
C 15.8807065 20.8193544 -37.8169131
N 14.9853566 21.7589121 -37.7435133
H 14.3645694 21.8061651 -36.956886
H 14.8726153 22.4170928 -38.4946404
N 15.9472023 19.9843426 -36.8189475
H 16.6241288 19.2455779 -36.8502505
H 15.2949912 20.0477172 -36.0589826
N 18.7300829 17.5290883 -43.1764286
H 17.9068943 17.0488226 -43.5306661
C 20.0684893 17.0390531 -43.5746573
H 20.7065707 16.9291063 -42.6964889
C 20.7865705 18.0002679 -44.5366372
O 22.0134416 18.0053269 -44.5586972
C 19.9813346 15.699411 -44.3219547
H 19.304658 15.8747004 -45.1616404
H 20.9671976 15.5209637 -44.7569981
C 19.5628823 14.3578569 -43.6784448
H 20.3290906 14.0366348 -42.971993
H 18.6129878 14.4437749 -43.1562454
C 19.4543242 13.3380914 -44.844005
H 20.418487 13.3271226 -45.354375
H 18.7167883 13.694725 -45.5669937
C 19.1362141 11.8730891 -44.4980398
H 19.490443 11.6480878 -43.4892399
H 19.686472 11.2395421 -45.2001729
N 17.6955314 11.5486037 -44.6343123
H 17.1272324 12.0169392 -43.9401613
H 17.4806865 10.5531329 -44.5600195
H 17.3202713 11.7308229 -45.5705452
N 20.0478271 18.6624719 -45.4364741
H 19.046923 18.5093921 -45.436498
C 20.6358853 19.3309775 -46.6085273
H 21.6749389 19.5607893 -46.3684505
C 20.0541746 20.7091617 -46.9622581
O 20.3152965 21.2210728 -48.0515675

C 20.6868561 18.354292 -47.7957786
H 21.0473326 18.879063 -48.6814314
H 21.4566581 17.6177888 -47.5613333
C 19.4373287 17.602644 -48.1667651
C 19.394892 16.1940631 -48.1271936
H 20.196105 15.6434021 -47.6560693
C 18.3451897 15.5113149 -48.7728262
H 18.2923324 14.4372359 -48.7484014
C 17.3896775 16.2497781 -49.5044677
O 16.5031357 15.6745726 -50.3589183
H 16.8175994 14.8393012 -50.723428
C 17.3695605 17.6457992 -49.4114359
H 16.6398896 18.1806343 -49.9827419
C 18.3848271 18.32481 -48.7295873
H 18.4231516 19.3990316 -48.7319403
N 19.3517895 21.3539171 -46.024161
H 19.1651048 20.8775033 -45.1539639
C 19.0384741 22.7911583 -46.0750765
H 18.3539854 22.9771147 -45.2457682
C 18.2660689 23.2374827 -47.3416898
O 18.3498463 24.3844502 -47.7803859
C 20.3254224 23.5929801 -45.7682459
H 21.0190659 23.4837652 -46.6030665
H 20.8007875 23.1682607 -44.8823023
C 20.0831759 25.0814136 -45.500918
O 19.0655738 25.4065578 -44.8420731
O 20.8655128 25.9146396 -46.0124582
N 17.4548563 22.3556176 -47.9295338
H 17.4238769 21.424189 -47.5465261
C 16.4823397 22.7281121 -48.9710723
H 16.9464183 23.4477338 -49.6473765
C 15.24281 23.3977265 -48.3725054
O 14.9522023 23.2457856 -47.1836795
C 16.0422905 21.5162724 -49.8130752
H 15.2603537 21.8499514 -50.4920367
C 17.2011516 21.0558416 -50.7125561
H 18.1143336 20.9147098 -50.1404907
H 17.4026366 21.8357731 -51.4439333
H 16.943333 20.1436236 -51.2459316
C 15.398919 20.4182088 -48.9405732
H 16.1665696 19.8238888 -48.4571155
H 14.7955406 20.8598812 -48.151009
C 14.4493492 19.5350595 -49.7478417
H 14.8862927 19.2531794 -50.7036183
H 13.5268763 20.0795328 -49.9194258
H 14.2201392 18.6408149 -49.1834476

N 14.4360211 24.010578 -49.2375354
H 14.7402083 24.0446147 -50.2085445
C 12.9993631 24.158699 -49.0076405
H 12.8175786 24.2573771 -47.9397338
C 12.2662528 22.9046082 -49.5103378
O 12.6107608 22.3634176 -50.5604784
C 12.5234879 25.4517901 -49.6859483
H 13.0406288 26.283309 -49.2048236
H 12.830272 25.44303 -50.731898
C 11.0112581 25.7338392 -49.6079951
H 10.8575365 26.7986213 -49.7828324
H 10.658697 25.5126149 -48.6002629
C 10.1424015 24.9839405 -50.6247752
O 10.5934761 24.3664138 -51.5757024
N 8.8434225 24.9954737 -50.4307784
H 8.2650384 24.4828781 -51.0906918
H 8.4331033 25.5005896 -49.6701504
N 11.2359635 22.4621827 -48.7844512
H 11.0578115 22.9049529 -47.8891113
C 10.3429926 21.383246 -49.214284
H 10.5949631 21.0920705 -50.235015
C 8.8711568 21.8264889 -49.2272665
O 8.3637704 22.3460571 -48.2286926
C 10.5732244 20.1618264 -48.309774
H 10.3332883 20.4190762 -47.2777976
H 11.6227969 19.8672222 -48.3609254
S 9.5400098 18.7650291 -48.8429061
H 8.3402798 19.3259352 -48.6493615
N 8.1565084 21.4877271 -50.299685
H 8.6580875 21.1050509 -51.0997332
C 6.6915622 21.4723115 -50.3796701
H 6.2622286 21.8728319 -49.4665579
C 6.2201094 20.031677 -50.5303368
O 6.4399128 19.4158451 -51.5664035
C 6.1943466 22.3420754 -51.5434893
H 6.7074003 22.060723 -52.4640156
C 4.6826819 22.2560971 -51.7615391
H 4.1511206 22.4652032 -50.8329504
H 4.408085 21.2625488 -52.117419
H 4.3832472 22.9787312 -52.5207642
O 6.4597851 23.693421 -51.2481149
H 6.0035097 23.908674 -50.423727
N 5.5750189 19.4858299 -49.5003679
H 5.4489048 20.0421054 -48.6609263
C 4.8934822 18.1915638 -49.5707935
H 5.3805521 17.5583986 -50.3075649

H 4.9561912 17.6920084 -48.6044006
C 3.4178491 18.3381219 -49.9451647
O 2.7499778 19.2457229 -49.4508098
N 2.8759735 17.4192718 -50.7447628
H 3.489359 16.728848 -51.172119
C 1.4218424 17.2768095 -50.9304672
H 0.9352328 17.8212694 -50.1261785
C 0.9560668 15.8305307 -50.7697592
O 1.6406314 14.8966804 -51.1867693
C 0.9136146 17.8905378 -52.2565359
H -0.1448096 17.6360649 -52.3435319
C 1.6387051 17.3248981 -53.5004349
H 2.6843131 17.6363338 -53.4916327
H 1.6106378 16.2363657 -53.4623999
C 0.9961788 19.4249524 -52.1858548
H 2.0365676 19.7521869 -52.1926341
H 0.5247676 19.7783184 -51.266841
H 0.4642133 19.8755679 -53.0218273
C 1.0052803 17.7519525 -54.8316522
H 1.1088672 18.8260369 -54.9781292
H -0.0515642 17.481976 -54.8436029
H 1.510511 17.2397663 -55.65089
N -0.2477491 15.6495421 -50.2260329
H -0.7465903 16.4668413 -49.8863459
C -0.9656272 14.3704334 -50.212057
H -0.6470092 13.784003 -51.0743246
C -2.4736961 14.6046495 -50.3361702
O -2.9923013 15.6108318 -49.855645
C -0.6345655 13.5626088 -48.9469876
H -1.035592 14.063649 -48.0667914
H 0.4483293 13.4718768 -48.8486547
O -1.2039403 12.2647033 -49.0661658
H -0.8230701 11.6633302 -48.3915009
N -3.189996 13.6624306 -50.9545782
H -2.693636 12.8593069 -51.3085801
C -4.6570956 13.7038033 -51.1109514
H -4.9453094 14.6681594 -51.5324737
C -5.3659256 13.5807981 -49.7505522
O -6.4750307 14.0756237 -49.5707605
C -5.0838402 12.5810381 -52.0946599
H -4.5868338 11.656628 -51.7934535
C -6.60297 12.3174183 -52.0927149
H -7.1427278 13.2367823 -52.3255887
H -6.9278242 11.951214 -51.1188279
H -6.8636599 11.5560362 -52.8261546
C -4.6265523 12.9470809 -53.5247128

H -5.2699191 13.744181 -53.8956398
H -3.6030739 13.3215718 -53.5000957
C -4.6555746 11.788384 -54.5300713
H -5.6777838 11.458972 -54.7113472
H -4.0625365 10.9539342 -54.1543668
H -4.2321241 12.1260136 -55.4769672
N -4.7271601 12.913471 -48.788543
H -3.775143 12.6272275 -48.9723444
C -5.2999645 12.5558781 -47.4919011
H -6.3581933 12.3426238 -47.6238231
C -5.12327 13.6827605 -46.4548777
O -3.9915082 13.9538372 -46.0403971
C -4.6452439 11.2446981 -47.0565748
H -3.587913 11.2809028 -47.3174804
H -5.1004324 10.4159779 -47.6001787
O -4.7366918 11.0128571 -45.6723862
H -5.6758702 11.0025864 -45.3613888
N -6.2181431 14.2734505 -45.9265974
C -6.1448261 15.2535177 -44.8392723
H -5.5037262 16.0768951 -45.1469994
C -5.5994107 14.6593758 -43.5365247
O -4.990551 15.3639187 -42.7414758
C -7.5767455 15.7683423 -44.6357585
H -8.056633 15.2366621 -43.8112928
H -7.5919532 16.8425494 -44.4489233
C -7.6005661 14.1249832 -46.3585443
H -7.6892693 14.0050037 -47.4364082
H -8.051605 13.2737373 -45.845865
C -8.2932888 15.4153499 -45.9362787
H -8.111043 16.1900229 -46.680453
H -9.3633916 15.2698172 -45.7853592
N -5.798928 13.3603836 -43.3244477
H -6.3334307 12.8472974 -44.0199305
C -5.3819279 12.5909009 -42.1533687
H -5.5086175 13.2194111 -41.2710266
C -3.889537 12.1953296 -42.1819728
O -3.2267231 12.2061379 -41.1417984
C -6.3412377 11.3905832 -41.9922851
H -7.3113502 11.7703061 -41.6675729
H -5.9575394 10.7498656 -41.1971473
C -6.5663179 10.5388814 -43.2538189
O -6.908747 11.078591 -44.3329274
O -6.3508923 9.3107568 -43.1812859
N -3.3008267 11.9683216 -43.3619274
H -3.8866016 11.9470974 -44.1933319
C -1.8430178 11.9831623 -43.5618261

H -1.3723892 11.257173 -42.9150736
C -1.2618897 13.3502526 -43.1982805
O -0.4067809 13.4546671 -42.3135894
C -1.4776396 11.6187214 -45.0103698
H -2.1218696 12.1563712 -45.7031098
H -0.4492902 11.9202012 -45.2048059
C -1.5826308 10.1109484 -45.2530965
H -2.542401 9.7508069 -44.8866851
H -0.8029225 9.6052696 -44.6769365
C -1.4265517 9.7523301 -46.7342513
O -2.1887154 8.9014817 -47.2378949
O -0.4387212 10.1870761 -47.3704002
N -1.8238071 14.3981459 -43.7998905
H -2.5652821 14.2183388 -44.4716944
C -1.3629664 15.7743681 -43.644951
H -0.2966211 15.7971953 -43.8619811
C -1.5011144 16.2671664 -42.1931932
O -0.5669668 16.8774303 -41.6842395
C -2.0937697 16.6432986 -44.6956286
H -3.1616424 16.4331521 -44.6208048
C -1.6453572 16.3062192 -46.1415492
H -1.8060816 15.2459139 -46.3228022
H -2.2857106 16.8442621 -46.8391984
C -1.9169274 18.1412204 -44.4519846
H -0.8624135 18.3648704 -44.4776171
H -2.351247 18.4235725 -43.4927015
H -2.4173335 18.7036014 -45.2397571
C -0.1850993 16.6247081 -46.5047295
H 0.502714 16.0852862 -45.8550906
H 0.005957 17.6948585 -46.4390428
H -0.0012286 16.3148047 -47.5317343
N -2.5671139 15.9256739 -41.4689525
H -3.3267024 15.4447242 -41.9411177
C -2.7735512 16.3191514 -40.0675664
H -2.7786111 17.4067291 -40.0129945
C -1.6436206 15.8404344 -39.1348382
O -1.1884872 16.6049745 -38.2795479
C -4.1712491 15.8244989 -39.6523631
H -4.8964345 16.3669418 -40.2600162
H -4.276831 14.7663523 -39.8970248
C -4.5547229 16.0502673 -38.1793378
H -5.6347657 16.1922723 -38.1337433
H -4.0852274 16.9595111 -37.8033673
C -4.2106249 14.87867 -37.2602295
O -3.478791 13.9624486 -37.5900405
N -4.7524033 14.838826 -36.0651956

H -4.5608134 14.0108061 -35.5281391
H -5.3835633 15.5549238 -35.7602172
N -1.1004638 14.6424705 -39.3708469
H -1.5013084 14.0764096 -40.106404
C 0.0418472 14.1322373 -38.6116889
H -0.0436926 14.4501244 -37.5713118
C 1.3850099 14.6518156 -39.1239408
O 2.2660308 14.8908551 -38.3035319
C 0.0435491 12.6104591 -38.6527647
H 1.0465421 12.2488474 -38.4138007
H -0.2262749 12.3017169 -39.6598596
S -1.1117245 11.9445062 -37.4241918
H -2.0986728 12.845829 -37.6105998
N 1.5575739 14.8170369 -40.4417058
H 0.8054631 14.5690666 -41.0762493
C 2.7701594 15.422793 -40.987119
H 3.6444812 14.9045317 -40.5883586
C 2.8696392 16.8863013 -40.5636503
O 3.5847836 17.2487937 -39.6283336
C 2.7907183 15.3258836 -42.5238653
H 1.9156234 15.7985274 -42.9702768
H 2.7822393 14.2994419 -42.8465516
H 3.7036186 15.7788831 -42.9060338
N 2.1002966 17.7180647 -41.2791163
H 1.1970245 17.3057366 -41.4945638
C 2.6117307 18.5517644 -42.3861192
H 3.1341734 17.8723516 -43.0516016
C 1.3970127 19.0600589 -43.2242848
H 1.358215 18.4262558 -44.0974844
H 0.5018309 18.6692998 -42.7733033
C 1.1166488 20.5727843 -43.5444475
H 1.2915895 21.151315 -42.6635274
H 1.8411256 20.9728381 -44.2381746
C -0.3230066 20.9360951 -43.9914732
H -1.0227159 20.3720331 -43.3859128
H -0.4699022 20.6178342 -45.0179328
C -0.6963571 22.4298742 -43.8025919
H 0.1995555 22.9755746 -43.4898631
H -1.4260614 22.4979107 -42.9928553
N -1.2635441 23.0865246 -45.012343
H -0.5895041 23.0906335 -45.7804691
H -1.4992876 24.0597524 -44.8902245
H -2.0834365 22.6400856 -45.4285437
C 3.7642777 19.4606364 -41.9383207
O 4.6934606 18.9814363 -41.3095185
N 3.6311882 20.7771193 -41.9989438

H 2.8876535 21.1417414 -42.5604266
C 4.0788258 21.6000937 -40.8865894
H 5.1237588 21.3834285 -40.6673247
C 3.9999911 23.0820359 -41.3327063
H 3.0493725 23.2473995 -41.8409777
H 4.7944901 23.2717171 -42.0553756
C 4.1026739 24.1179475 -40.2399803
N 3.0918861 25.0331692 -39.9409579
C 3.4223691 25.5589047 -38.7527186
H 2.8010558 26.2448663 -38.192991
N 4.5752577 25.0358408 -38.3042514
H 4.8930887 25.1113105 -37.3429746
C 5.0364284 24.1390869 -39.2428601
H 5.8685618 23.4582498 -39.1352582
C 3.2420288 21.199852 -39.6385361
O 3.0797663 22.025223 -38.7500341
N 2.530232 20.0383135 -39.640792
H 2.865367 19.2416506 -40.1623869
C 1.4823966 19.8353562 -38.7041374
H 1.2481924 20.7922532 -38.2384218
C 2.0858318 19.0933861 -37.5361898
O 3.067777 19.6165891 -37.0050026
C 0.1413169 19.4928001 -39.3442083
H 0.1442248 18.4588372 -39.6817178
H -0.6141341 19.569777 -38.5611991
C -0.267998 20.4123699 -40.5175301
H 0.1183068 19.9509151 -41.3678312
C -1.7776922 20.4353344 -40.70414
H -2.2492346 20.8376265 -39.8066394
H -2.1313518 19.4171078 -40.8620015
H -2.0806715 21.041301 -41.5528518
C 0.1944014 21.8747087 -40.5369619
H 1.2344464 22.003532 -40.7787416
H 0.035205 22.3050513 -39.5470494
H -0.3843236 22.4742456 -41.2283432
N 1.4878269 18.020571 -37.0275607
H 0.680745 17.619781 -37.490007
C 1.8370793 17.567107 -35.682473
H 1.6480608 18.3923588 -34.994206
C 3.3255548 17.1909504 -35.5105984
O 3.975249 17.6990186 -34.5966676
C 0.897452 16.4265003 -35.2938252
H 1.0844826 15.5580817 -35.9233497
H -0.1416476 16.7397962 -35.4098183
H 1.0723946 16.150676 -34.2533891
N 3.895938 16.3935091 -36.4204664

H 3.3273487 16.0340792 -37.1822979
C 5.2891756 15.9425756 -36.3358269
H 5.4806631 15.6036171 -35.3165186
C 6.3276383 17.0397494 -36.6405866
O 7.4910534 16.9063308 -36.2670223
C 5.4618095 14.744717 -37.274927
H 5.2733635 15.0489023 -38.3063018
H 4.7617187 13.9547081 -37.0015401
H 6.47971 14.3605827 -37.1961688
N 5.9285687 18.1328717 -37.2982429
H 4.962632 18.1795496 -37.6016175
C 6.8314934 19.2418052 -37.635999
H 7.8429605 18.8505207 -37.6832907
C 6.844892 20.3829303 -36.6121256
O 7.5699517 21.3645417 -36.8076774
C 6.4883151 19.7654549 -39.0249614
H 5.4763035 20.1549587 -38.9918722
H 6.5452826 18.940506 -39.7349417
O 7.3909096 20.7802827 -39.4271918
H 7.5897174 21.3108912 -38.6363837
N 6.0301942 20.3258281 -35.5583977
H 5.4633655 19.4990513 -35.4131305
C 5.9102691 21.4436743 -34.6242259
H 5.8569889 22.3420848 -35.2375565
C 7.1639527 21.593333 -33.7492034
O 7.6308825 20.6298769 -33.149975
C 4.6035516 21.3149437 -33.8160339
H 3.9610644 20.5586151 -34.2705869
H 4.8261419 20.983383 -32.8007128
C 3.8146552 22.6339158 -33.7630863
H 4.3761121 23.361526 -33.1761685
H 2.8597389 22.454182 -33.2696363
C 3.5503843 23.2164719 -35.1512038
O 4.0029425 24.3035214 -35.4804174
N 2.9488639 22.4629638 -36.0411139
H 2.950617 22.7735293 -37.0102779
H 2.7918841 21.4817724 -35.8719266
N 7.7210904 22.8081187 -33.6914249
H 7.2987774 23.5567177 -34.2190106
C 8.9972279 23.0788989 -33.0131334
H 9.0701395 24.1482844 -32.8145984
H 9.0214978 22.5526099 -32.0586359
C 10.2493908 22.6746477 -33.805537
O 11.3286693 22.5814544 -33.2268775
N 10.1271567 22.3925806 -35.1093067
H 9.2142375 22.4390954 -35.5407744

C 11.2828804 22.1739636 -35.9765919
H 12.1470555 21.9182832 -35.3639204
C 11.6311631 23.4425166 -36.7703543
O 10.8468171 23.8739616 -37.6062429
C 11.0248787 21.0051895 -36.9419587
H 10.2282802 21.2982624 -37.627223
H 11.9359448 20.8776523 -37.5163873
C 10.6521745 19.6372191 -36.3484215
H 9.6886744 19.7031776 -35.8480046
C 10.554009 18.6256642 -37.4922608
H 11.5421073 18.4392663 -37.908598
H 9.9033941 19.0025286 -38.2786216
H 10.1402736 17.6890333 -37.1141196
C 11.700169 19.1190278 -35.363519
H 12.6761902 19.0615675 -35.8409176
H 11.4049478 18.1266817 -35.0192001
H 11.7434391 19.7788958 -34.4960605
N 12.8948299 23.8661966 -36.7091582
H 13.3352564 23.7065076 -35.812184
C 13.4155394 25.0179326 -37.4790076
H 12.6681205 25.8145446 -37.4282954
C 13.6732908 24.7387133 -38.9836209
O 14.3679382 25.4938641 -39.6582383
C 14.7076514 25.5493859 -36.8171534
H 15.5330047 24.8870948 -37.0856383
H 14.9254605 26.5347873 -37.2302733
C 14.686988 25.6625318 -35.2845744
H 15.5866248 26.1902592 -34.9596257
H 14.7320807 24.6561241 -34.8586402
C 13.4378158 26.3886491 -34.7758366
O 13.4280188 27.6368107 -34.819975
O 12.4900572 25.6633847 -34.4019596
N 13.2648524 23.5772005 -39.5105643
H 12.5354699 23.085907 -39.0068225
C 13.5570177 23.1795007 -40.8976494
H 14.5254876 23.6009869 -41.1761233
C 12.542468 23.7627457 -41.8860466
O 11.3445727 23.7642826 -41.6133993
C 13.6717175 21.6505914 -41.0511484
H 14.6737933 21.3409764 -40.7633607
H 13.5409867 21.3814689 -42.0977122
C 12.6807254 20.8409029 -40.234541
O 13.0819143 19.9571612 -39.4998137
N 11.4063889 21.1652132 -40.2593715
H 10.7663929 20.6171536 -39.7086994
H 11.0835354 21.9909526 -40.7547637

N 13.0133136 24.1123106 -43.0888082
H 14.003369 23.9861169 -43.252692
C 12.2559559 24.8063065 -44.14478
H 11.634147 25.5703974 -43.6725806
C 11.2900693 23.8636724 -44.8836361
O 11.4794884 23.5296958 -46.0528063
C 13.2402964 25.5207442 -45.0935189
H 13.7974318 24.7585126 -45.6299273
H 12.6747639 26.1075278 -45.8195301
C 14.2367 26.4457212 -44.363787
H 13.6782029 27.2076148 -43.8182901
H 14.8081668 25.8651489 -43.6389009
C 15.2530414 27.1445263 -45.2802328
H 15.9662655 27.6665972 -44.6388995
H 14.7410762 27.8844194 -45.8974409
C 16.0017683 26.1590706 -46.1895801
H 15.4383225 26.0365783 -47.1185131
H 16.0485386 25.1791219 -45.7028254
N 17.3833513 26.6013149 -46.4784672
H 17.4501689 27.5257558 -46.8646949
H 17.8394313 25.9286781 -47.0934372
H 17.9672793 26.5200239 -45.6378488
N 10.2586788 23.4106932 -44.1767312
H 10.1841574 23.7178249 -43.2124954
C 9.2046284 22.5279462 -44.6902692
H 9.4646982 22.2318761 -45.7066837
C 7.8664666 23.2561846 -44.773848
O 7.5173305 24.0732073 -43.9268036
C 9.0728533 21.2239852 -43.8778855
H 8.316634 20.6091786 -44.3642886
C 10.3766276 20.4230278 -43.8853184
H 11.1563743 20.9672999 -43.3545985
H 10.6901088 20.2389319 -44.9118237
H 10.2204614 19.4614051 -43.3955968
C 8.6444678 21.4437578 -42.4225151
H 9.3296493 22.1190396 -41.9159669
H 8.6253233 20.491972 -41.8909169
H 7.6444986 21.8751438 -42.3818422
N 7.0848025 22.9020958 -45.7835561
H 7.4542566 22.2468272 -46.4660422
C 5.688535 23.2901281 -45.9603944
H 5.2592379 23.5642908 -44.9971347
C 4.9368154 22.090787 -46.5162855
O 5.5175932 21.2799915 -47.2402207
C 5.572196 24.4861637 -46.9093264
H 6.1315988 25.3254024 -46.4927753

H 4.5253632 24.7775338 -47.0057321
O 6.0920421 24.166681 -48.1936715
H 6.9019288 23.6412406 -48.069159
N 3.6649475 21.9328174 -46.1572492
H 3.1924113 22.6509743 -45.6290061
C 2.8540294 20.891737 -46.7718369
H 3.2603949 20.7420939 -47.768523
C 1.4232672 21.3469321 -46.9993605
O 0.9655742 22.2461308 -46.2958749
C 2.8723023 19.5311039 -46.0727033
H 1.8998189 19.3593649 -45.6756641
H 2.831625 18.8431272 -46.9167144
C 4.0238134 18.9905664 -45.2169582
C 4.3071869 17.6253821 -45.3860567
H 3.6888653 17.0224533 -46.0309537
C 4.8919789 19.744078 -44.3979068
H 4.7530479 20.7996534 -44.2590226
C 5.3992881 17.0235059 -44.752768
H 5.5957283 15.9801922 -44.9285487
C 5.9619852 19.1264943 -43.7177794
H 6.5828412 19.700868 -43.0462872
C 6.2154413 17.761582 -43.8888187
H 7.0295482 17.2877361 -43.3583586
N 0.7049211 20.6843602 -47.9071269
H 1.1580329 19.9917536 -48.4999263
C -0.7426341 20.8387991 -48.0115702
H -1.0824204 20.9641876 -46.9898499
C -1.5361428 19.617803 -48.4852348
O -0.9924186 18.6595985 -49.0384563
C -1.11455 22.1121096 -48.8003637
H -1.7026334 21.8618073 -49.6844732
H -0.2187602 22.6483505 -49.119513
C -1.9431406 22.9948283 -47.8556361
O -2.8487189 22.4398679 -47.1824651
O -1.4945848 24.1167787 -47.540539
N -2.8489941 19.6723543 -48.2388324
H -3.2118883 20.5386921 -47.8451067
C -3.8093695 18.7281656 -48.8164363
H -3.3973647 17.7249245 -48.7310293
C -3.9734597 19.0752603 -50.2936677
O -4.3973474 20.175993 -50.6415666
C -5.1732061 18.7435119 -48.0992768
H -5.6730347 19.6950951 -48.2825975
C -6.0637773 17.6068255 -48.6193746
H -5.6052813 16.639463 -48.4108445
H -6.2151491 17.7031978 -49.6940242

H -7.0395154 17.6576898 -48.1394967
C -5.0359728 18.5600012 -46.5826229
H -4.4763312 17.6507279 -46.373113
H -6.0235974 18.4938287 -46.1264681
H -4.5200769 19.4177832 -46.1531065
N -3.6215795 18.1436875 -51.1741785
H -3.2879382 17.2507786 -50.8282856
C -3.6115106 18.3903012 -52.6077545
H -4.5005105 18.9612831 -52.879691
H -2.7342712 18.9845956 -52.864576
C -3.5764643 17.1062873 -53.4192378
O -2.760003 16.2192524 -53.1788795
N -4.4347018 17.0516582 -54.4326559
H -5.0732818 17.8204822 -54.5581563
C -4.3864658 16.05468 -55.4956002
H -4.0915978 15.0912157 -55.0883004
C -3.3390088 16.4662952 -56.5310674
O -3.4495113 17.5140222 -57.164842
C -5.7890218 15.9175663 -56.0942179
H -6.1264015 16.9003061 -56.4288134
H -6.4684305 15.5787826 -55.3090581
C -5.884318 14.9468145 -57.2730016
O -4.8383242 14.4918336 -57.7921059
O -7.0322555 14.7422459 -57.7062923
N -2.3237965 15.6294866 -56.7222922
H -2.3348741 14.7515165 -56.2290766
C -1.1959614 15.917138 -57.5968154
H -0.8042434 16.9026035 -57.3349828
C -1.5477935 15.9520276 -59.0940706
O -0.7986989 16.5414193 -59.8719076
C -0.1261968 14.8713849 -57.2991538
H -0.4332321 13.8964363 -57.6832249
H 0.0211865 14.8034629 -56.2217764
H 0.8044678 15.171372 -57.7763393
N -2.6894224 15.3899282 -59.5099429
H -3.3057519 14.9434825 -58.8268714
C -3.1680469 15.5081525 -60.8889374
H -2.2977069 15.4764396 -61.5426236
C -3.8714604 16.8578481 -61.1353749
O -3.8881519 17.3427345 -62.2717246
C -4.0589673 14.2899356 -61.2019821
H -3.5800509 13.3944349 -60.8022451
H -5.0094949 14.4145144 -60.6792547
C -4.3336983 14.054691 -62.7013104
H -4.8066073 14.9365606 -63.1323418
C -3.0607759 13.732287 -63.4923323

H -2.5395826 12.8897156 -63.037984
H -2.3987076 14.5952794 -63.5160859
H -3.3198034 13.4669686 -64.5171777
C -5.2831101 12.8667808 -62.868187
H -4.8269732 11.9632398 -62.4652122
H -5.5106869 12.7195113 -63.9246257
H -6.2135557 13.0676634 -62.3361046
N -4.3438173 17.5317856 -60.0767708
H -4.239743 17.0913676 -59.1689351
C -5.1392953 18.7670931 -60.1508346
H -5.3101043 19.014173 -61.1954728
C -4.4652452 20.0192241 -59.553186
O -4.7247452 21.1197762 -60.036646
C -6.5275032 18.4864972 -59.5562995
H -7.1041324 19.4099779 -59.5149897
H -6.4332717 18.0886791 -58.546576
C -7.2894454 17.5033969 -60.4354308
O -7.5563357 17.7986585 -61.5883171
N -7.5704357 16.2932746 -60.0008698
H -8.0536807 15.6856149 -60.632247
H -7.3300548 15.9419432 -59.0695682
N -3.5291447 19.8666245 -58.6129897
H -3.3778296 18.9328279 -58.2480178
C -2.7161973 20.948246 -58.0426512
H -3.3974747 21.6988151 -57.6398965
C -1.8381347 21.6244684 -59.108357
O -1.3603305 20.9679296 -60.0376461
C -1.8708616 20.3801509 -56.8857777
H -1.2155043 19.5960797 -57.2688558
H -2.5403692 19.9339514 -56.1488536
C -1.0113161 21.4298223 -56.1713507
H -0.2443703 21.7912918 -56.8567282
H -0.5059511 20.9491715 -55.3327881
S -1.9402459 22.8506813 -55.5394919
C -0.5701828 23.8292679 -54.8690719
H -0.9613234 24.74235 -54.4197375
H 0.1193026 24.0926016 -55.6715556
H -0.0410525 23.252321 -54.1098706
N -1.6015408 22.9369568 -58.9811233
H -1.9423219 23.4054305 -58.1486339
C -0.8192637 23.7390643 -59.9395053
H -0.2576772 23.064157 -60.5851844
C 0.2282464 24.5957342 -59.2270807
O 0.004776 25.0706294 -58.1183271
C -1.7509353 24.6002576 -60.822006
H -2.2485006 25.3361505 -60.1875853

H -1.1470927 25.1432575 -61.5512182
C -2.8355023 23.8041089 -61.576147
H -3.4692309 24.5095959 -62.1148217
H -3.4670243 23.293783 -60.85025
C -2.285634 22.7794283 -62.5838831
H -1.469829 22.2166229 -62.1362081
H -1.8727529 23.3160784 -63.440655
N -3.3480238 21.8600974 -63.0454371
H -4.1592275 22.2924263 -63.4494333
C -3.3712553 20.539867 -62.9367282
N -4.3632524 19.8552986 -63.436589
H -4.394121 18.8635073 -63.2050823
H -5.2093213 20.3214733 -63.7056537
N -2.4470361 19.8412767 -62.3456859
H -1.8072108 20.3142319 -61.7116381
H -2.5589807 18.8412342 -62.2636225
N 1.3477479 24.8147152 -59.9086339
H 1.3980397 24.4714973 -60.8561952
C 2.4801481 25.6382775 -59.4817666
H 2.171737 26.310206 -58.6796175
C 2.9540436 26.4820188 -60.6742369
O 2.5493109 26.2124622 -61.8048482
C 3.6101897 24.7291168 -58.9715134
H 3.9556652 24.0994097 -59.7921348
H 4.4519802 25.3525014 -58.6710504
C 3.2414508 23.8502424 -57.790578
C 3.2255769 24.3988016 -56.4940977
H 3.4578802 25.4439975 -56.3432923
C 2.944317 22.484486 -57.9805326
H 2.957988 22.0603603 -58.9749413
C 2.9118273 23.5871558 -55.3886793
H 2.893409 23.9989979 -54.390721
C 2.6376701 21.6672874 -56.8746023
H 2.4174114 20.6212776 -57.0198648
C 2.6196309 22.2218223 -55.5761608
O 2.3179465 21.4526177 -54.4997302
H 2.18435 20.5319836 -54.724826
N 3.8334992 27.4628261 -60.4509498
H 4.2082822 27.584594 -59.5219213
C 4.4939213 28.1620494 -61.5616058
H 3.7309422 28.5488971 -62.2384471
C 5.3882736 27.2020676 -62.3485732
O 6.0176283 26.3114741 -61.7616727
C 5.3217809 29.3477141 -61.0436929
H 6.091829 28.9980095 -60.3569351
H 4.6612574 30.029898 -60.5071673

O 5.9393037 30.0538829 -62.1032955
H 6.7243398 29.5613144 -62.4093461
N 5.5820288 27.4940815 -63.6317522
H 5.0106585 28.1958685 -64.0746187
C 6.7307802 26.9874338 -64.3760478
H 6.6366855 25.9085016 -64.4254029
C 8.028407 27.3558119 -63.6304401
O 8.1187891 28.4400932 -63.0417593
C 6.7479946 27.5450176 -65.8100117
H 7.719706 27.3149662 -66.2505881
H 6.6485008 28.6315395 -65.7731873
C 5.6672195 26.9697377 -66.7358325
O 4.772257 26.2412801 -66.2508656
O 5.7522914 27.2543315 -67.9504335
N 9.0046514 26.4438149 -63.5827792
H 8.8767 25.5757138 -64.0999271
C 10.2928452 26.6834173 -62.9168859
H 10.9628395 25.863104 -63.1596956
H 10.726691 27.5994604 -63.3176861
C 10.2581107 26.8168125 -61.3836782
O 11.1929088 27.3706979 -60.812188
N 9.2068629 26.3463782 -60.7000323
H 8.4713915 25.8971 -61.2280263
C 9.0731089 26.4374344 -59.2283582
H 9.1851045 27.4791121 -58.9258279
C 10.1069491 25.6136815 -58.4432941
O 10.4836519 25.994552 -57.3288833
C 7.6897912 25.9495096 -58.7796666
H 7.6527518 25.9341602 -57.6888603
H 7.5223822 24.9374057 -59.148062
O 6.6580577 26.7898418 -59.2502181
H 6.5249451 26.5892104 -60.1998395
N 10.5092653 24.4553591 -58.9775317
H 10.148396 24.2047745 -59.8925566
C 11.2703098 23.4359545 -58.2511685
H 11.53296 23.831252 -57.2725145
C 12.5826537 23.0539197 -58.9343996
O 12.7237566 23.0892687 -60.153064
C 10.4016771 22.1945978 -58.0069762
H 10.1678104 21.7295215 -58.9665107
H 10.9725225 21.4720044 -57.4234963
C 9.1174901 22.4918067 -57.2616053
C 9.1440574 22.7882535 -55.8861933
H 10.0770687 22.7663086 -55.3446341
C 7.8965917 22.5106125 -57.9559699
H 7.8822044 22.2996273 -59.0114969

C 7.9532069 23.1130515 -55.2127899
H 7.9771022 23.3415634 -54.1554797
C 6.7052395 22.8277978 -57.284616
H 5.7704859 22.841606 -57.8236995
C 6.7337942 23.135257 -55.9133376
H 5.8189192 23.3828901 -55.3936062
N 13.517583 22.5839715 -58.1188466
H 13.3072881 22.5350507 -57.1278463
C 14.8179288 22.0601079 -58.5444263
H 15.0314648 22.3351386 -59.5799209
C 14.8820061 20.5283609 -58.4571595
O 15.7122647 19.8905221 -59.1081171
C 15.861854 22.6970743 -57.6264017
H 15.4958654 22.6849266 -56.6000881
H 16.7664742 22.1004187 -57.6568063
C 16.1785143 24.1454572 -58.0345006
H 16.4935678 24.6931106 -57.143149
H 15.2907343 24.6425045 -58.4312527
C 17.305602 24.1476694 -59.067901
O 17.1190339 23.5958259 -60.1692975
O 18.44763 24.4781666 -58.6847995
N 14.0008482 19.9431131 -57.6415347
H 13.375073 20.5442361 -57.11928
C 13.8557196 18.5135042 -57.3961515
H 14.3000178 17.9505765 -58.2132188
C 12.3562485 18.1809218 -57.2916426
O 11.6055412 18.9392478 -56.6695756
C 14.5613807 18.1437631 -56.0764476
H 13.9703084 18.5856413 -55.2756477
H 14.5331341 17.0604963 -55.9537592
C 16.0075358 18.6382877 -55.8749913
H 16.0774433 19.7022195 -56.0961422
C 16.4272853 18.4705187 -54.4170553
H 16.4803628 17.4129 -54.1558347
H 15.7119752 18.972793 -53.7650525
H 17.3987076 18.9361346 -54.2814031
C 17.0207935 17.8886108 -56.7338335
H 16.9321508 16.814618 -56.5697651
H 18.0321975 18.2073195 -56.4892937
H 16.8337156 18.1120355 -57.7796173
N 11.9268476 17.0254245 -57.802589
H 12.5667476 16.4679935 -58.3580903
C 10.6134922 16.4408486 -57.4727742
H 10.200647 16.9817986 -56.6240596
C 10.8460628 14.9974708 -57.0338666
O 11.4968357 14.2196056 -57.7343886

C 9.584129 16.5453119 -58.623281
H 9.8891 15.8453796 -59.384544
C 9.5544193 17.9798466 -59.2141413
H 9.2019235 18.6905423 -58.4697116
H 10.5636784 18.2729503 -59.500348
C 8.1844474 16.0604285 -58.2049168
H 7.7911132 16.6907992 -57.4099126
H 8.2346799 15.0332518 -57.8434264
H 7.5072373 16.083636 -59.0554359
C 8.7221937 18.1222675 -60.4791129
H 7.6661354 18.1114382 -60.2270789
H 8.9737782 17.3097286 -61.1536164
H 8.9770545 19.0592215 -60.9698104
N 10.3564225 14.6574374 -55.8469035
H 9.7941865 15.3397182 -55.3446681
C 10.5610505 13.361721 -55.2088406
H 11.1527113 12.721799 -55.8623133
C 9.2265463 12.6740554 -54.9607565
O 8.2716464 13.3041168 -54.5053329
C 11.33476 13.5316808 -53.9000093
H 12.3374437 13.8914798 -54.1303004
H 10.8329836 14.2807638 -53.2847405
C 11.4560679 12.2414929 -53.107116
C 12.2584577 11.1893237 -53.5913009
H 12.8093437 11.3079028 -54.5115507
C 12.3285074 9.9712448 -52.892086
H 12.935959 9.1595666 -53.2719667
C 11.6002187 9.80203 -51.7022888
H 11.6597189 8.8679635 -51.1608939
C 10.7885537 10.8448729 -51.2227153
H 10.2173261 10.7141468 -50.3149552
C 10.7115946 12.0603749 -51.9250526
H 10.0647863 12.8469327 -51.5572318
N 9.1602094 11.3777451 -55.2613215
H 9.967209 10.9333375 -55.6892098
C 7.9328766 10.5948167 -55.1189933
H 7.301889 11.0701356 -54.3646456
C 8.255057 9.1831792 -54.6477118
O 9.0891373 8.509012 -55.2435789
C 7.1597415 10.5440188 -56.4471444
H 7.7114147 9.9222563 -57.1494066
C 5.8095002 9.8629732 -56.2113163
H 5.4896513 10.0212756 -55.1882349
H 5.9140703 8.7885214 -56.3956002
H 5.0585275 10.2781186 -56.8705113
C 7.0334598 11.9183157 -57.1168015

H 6.7794035 12.6694729 -56.3779092
H 6.2773547 11.9109892 -57.8926942
H 7.9869524 12.1928344 -57.5734035
N 7.5304832 8.6922801 -53.6430702
H 6.8760449 9.3036927 -53.1726615
C 7.6681163 7.3252057 -53.1358409
H 8.2068826 6.7406371 -53.8801015
C 6.2876119 6.6673452 -52.9862792
O 5.4097723 7.1751835 -52.2984167
C 8.5623854 7.3067255 -51.8731256
H 9.5579647 7.6240205 -52.1890165
C 8.094785 8.2976582 -50.7891014
H 7.0661867 8.0877915 -50.5071326
H 8.1462975 9.3195321 -51.1659953
H 8.7354529 8.2405992 -49.9112774
C 8.6896144 5.865025 -51.3446308
H 7.7397634 5.5693468 -50.9079072
H 8.8984866 5.198318 -52.1818034
C 9.804598 5.6643442 -50.3096542
H 9.5898272 6.2220642 -49.3977556
H 10.7584064 5.9929064 -50.7211156
H 9.8773923 4.6049462 -50.0589894
N 6.0735872 5.5791504 -53.7322323
H 6.8322139 5.3057652 -54.3480851
C 4.8587987 4.7465541 -53.7745885
H 4.9169297 4.1923917 -54.7089597
C 3.516118 5.5057891 -53.8614048
O 2.5191161 5.1118456 -53.2532807
C 4.8607276 3.7088065 -52.6438668
H 4.6254463 4.2273965 -51.7263386
H 4.0580549 2.9937707 -52.8329263
C 6.1573545 2.9237375 -52.4338794
H 5.9667513 2.1123083 -51.7318169
H 6.9089625 3.5794581 -51.9982039
C 6.7097361 2.3381834 -53.7201912
O 7.8204849 2.6367907 -54.124086
N 5.9596121 1.5408023 -54.4434662
H 6.3860314 1.1601171 -55.2659575
H 4.9443976 1.5731702 -54.3602218
N 3.4922299 6.609794 -54.6151327
H 4.3734201 6.9183661 -54.9925454
C 2.3102518 7.4619524 -54.7883182
H 1.523502 7.1422596 -54.1042785
C 1.7272705 7.3825057 -56.209906
O 0.5213259 7.2247492 -56.3878293
C 2.7044591 8.890572 -54.3960494

H 3.4554001 9.2689719 -55.0839756
H 3.1257726 8.8895343 -53.3873539
S 1.267988 9.9956678 -54.455393
H 1.9581013 11.1116626 -54.1764562
N 2.5785643 7.3956426 -57.2408166
H 3.5667076 7.4519611 -57.0465178
C 2.1412431 7.4998195 -58.6453044
H 1.3706166 8.2684934 -58.6961616
C 1.455654 6.224998 -59.1639819
O 0.6846533 6.2812467 -60.1185169
C 3.3271499 7.9979964 -59.498981
H 4.2271993 7.4604443 -59.1935633
C 3.5120954 9.5030321 -59.2234801
H 2.7433385 10.070109 -59.7525158
H 3.3888159 9.7067547 -58.1618482
C 3.1360484 7.7830679 -61.0060698
H 2.1735433 8.1823495 -61.3276595
H 3.2063335 6.7193164 -61.2171017
H 3.9295564 8.2650958 -61.5743967
C 4.8911054 10.0097662 -59.6504475
H 5.0099305 9.9628237 -60.7314375
H 5.6735898 9.4236341 -59.1685517
H 4.9797716 11.0455733 -59.3443017
N 1.5717582 5.1041732 -58.4564899
H 2.1827045 5.1206104 -57.6529454
C 0.7328439 3.9149034 -58.651388
H 0.8947388 3.5462889 -59.6639284
C -0.7823536 4.1812482 -58.5331897
O -1.5480582 3.5308124 -59.2486143
C 1.1982313 2.8047712 -57.6992863
H 0.5310809 1.9524817 -57.8022436
H 2.1807305 2.4791663 -58.0396875
C 1.2976814 3.1948038 -56.2119626
H 0.3012642 3.1688227 -55.7657597
H 1.6944627 4.205053 -56.0979221
C 2.2384522 2.2254211 -55.4939914
O 3.4720441 2.4194285 -55.590334
O 1.7426194 1.2099946 -54.9540701
N -1.2002262 5.2068174 -57.7761104
H -0.5129244 5.7123035 -57.2254629
C -2.598939 5.6605511 -57.6700557
H -3.2650489 4.7962077 -57.7183515
C -3.0249164 6.6303356 -58.7861373
O -4.212663 6.7435325 -59.0897442
C -2.8084363 6.3407486 -56.3074555
H -3.8525083 6.6461592 -56.2269157

H -2.1951503 7.2411331 -56.2540386
C -2.494111 5.4648105 -55.1222584
N -3.2748369 4.3995001 -54.6813414
C -1.402258 5.5724542 -54.3123075
H -0.6083186 6.2963122 -54.4092074
C -2.6439835 3.8919381 -53.61534
H -2.9894624 3.0311948 -53.0587202
N -1.5076655 4.5702295 -53.3754836
H -0.8044458 4.3204002 -52.6953078
N -2.0794667 7.3391896 -59.4136958
H -1.1158875 7.1556197 -59.1719981
C -2.3616997 8.3287098 -60.4667779
H -3.2276859 8.9173499 -60.1602128
C -2.7464924 7.5875773 -61.7548098
O -2.001621 6.7304906 -62.2333305
C -1.169923 9.3091347 -60.6337605
H -0.2399088 8.7535995 -60.5132491
C -1.1908147 10.461597 -59.5993055
H -0.3002541 11.0742561 -59.749503
H -2.0610367 11.0934907 -59.7853356
C -1.1248122 9.968303 -62.0256172
H -2.0519598 10.5058078 -62.2273489
H -0.9656117 9.221869 -62.8030621
H -0.2929621 10.6711498 -62.0761009
C -1.2165483 10.0459058 -58.1225757
H -0.4028053 9.3553923 -57.9105932
H -2.1699243 9.5788426 -57.8756608
H -1.0975652 10.9321865 -57.4986943
N -3.9191623 7.9002296 -62.3131057
H -4.4943561 8.5906527 -61.8567087
C -4.4811881 7.1647627 -63.4544377
H -4.2521094 6.1048541 -63.3300885
C -3.8496852 7.5951677 -64.7889643
O -3.2727482 6.7640869 -65.4867586
C -6.0132403 7.3088003 -63.4796484
H -6.2694267 8.3537053 -63.6622205
H -6.4039576 6.7184002 -64.3096062
C -6.7166646 6.8608593 -62.1861027
H -7.7887787 7.0203155 -62.3018223
H -6.3872767 7.4812947 -61.3521355
C -6.4970237 5.3889815 -61.8459751
O -6.9995508 4.4963847 -62.5114945
N -5.7572628 5.0902401 -60.8042215
H -5.833909 4.14822 -60.4307188
H -5.3482376 5.8097848 -60.2156199
N -3.8569681 8.8981386 -65.0946658

H -4.3202539 9.5439063 -64.4785248
C -3.1401507 9.4583446 -66.2477274
H -3.0411782 8.6833109 -67.0107969
C -1.7236527 9.8698317 -65.8282051
O -1.4618108 10.9868417 -65.364135
C -3.9305454 10.5951731 -66.9132344
H -4.0322571 11.4261952 -66.2131145
H -4.9304066 10.2295566 -67.1538309
C -3.2659619 11.0967388 -68.2087631
O -2.0957811 10.7292587 -68.4770125
O -3.9030893 11.919111 -68.8986723
N -0.803794 8.9111941 -65.9612056
H -1.116004 8.0117478 -66.302686
C 0.6148988 9.1220086 -65.6716635
H 0.701243 9.6227611 -64.7065084
C 1.2656105 10.0741342 -66.6689175
O 2.098196 10.8648421 -66.2441968
C 1.3529267 7.7757301 -65.5926566
H 1.1126767 7.1689474 -66.4679944
H 2.4297305 7.955249 -65.5762918
C 0.9574017 7.0366377 -64.3062496
H 1.2856483 7.6316669 -63.4540904
H -0.1280695 6.9419732 -64.2625537
C 1.5717334 5.633152 -64.2128231
H 2.6407791 5.6991469 -64.4166036
H 1.1128656 4.9710833 -64.9484462
C 1.3819517 5.0770151 -62.7971826
H 1.7874912 5.8138986 -62.1066302
H 1.9538723 4.1524789 -62.6816544
N -0.0351696 4.8430507 -62.4374898
H -0.6226182 5.656999 -62.6128357
H -0.1159741 4.6848988 -61.4413045
H -0.410129 4.0257107 -62.9061462
N 0.857613 10.1083443 -67.9375912
H 0.0306158 9.5949737 -68.2147783
C 1.4627276 11.0419099 -68.8908006
H 2.5495178 10.9536548 -68.8456466
C 1.0955327 12.4971915 -68.5945729
O 1.9570158 13.368256 -68.6695282
C 1.0240044 10.7936445 -70.3246517
H -0.0383867 11.0268319 -70.4208665
H 1.5987591 11.5133366 -70.9013528
C 1.2870139 9.4334093 -70.9591885
H 0.6207275 8.6875652 -70.5204886
H 2.321262 9.1555211 -70.7495981
C 1.0670261 9.5267931 -72.4818624

O 1.5837226 8.6346019 -73.1863309
O 0.449561 10.5278113 -72.9382914
N -0.1651418 12.7909911 -68.2489103
H -0.8538143 12.0316837 -68.2624408
C -0.597046 14.1400191 -67.8500139
H -0.3606733 14.8368522 -68.6493023
C 0.1788132 14.6128788 -66.6331437
O 0.6875716 15.7306213 -66.6664212
C -2.1126775 14.1466946 -67.5941196
H -2.6227997 14.0571914 -68.5530931
H -2.3576696 13.272807 -66.9910062
C -2.6896842 15.3612165 -66.8459386
H -3.7652708 15.2087699 -66.7430743
H -2.2667664 15.3935762 -65.8416272
C -2.4521123 16.7168604 -67.5216241
H -2.901966 16.7154243 -68.5155877
H -1.3820194 16.9074539 -67.6060141
C -3.0951271 17.8042082 -66.6541248
H -2.7560098 17.6658543 -65.6223159
H -4.1813943 17.6762267 -66.6795216
N -2.71647 19.159312 -67.1134731
H -1.700728 19.2804625 -67.0277463
H -3.1123092 19.8764223 -66.5258363
H -2.9676407 19.3212056 -68.075571
N 0.3204954 13.766832 -65.6119406
H -0.1301998 12.8600258 -65.6626692
C 1.1372004 14.0988161 -64.4489276
H 0.7681291 15.0311553 -64.0186157
C 2.6010132 14.3488103 -64.8440332
O 3.1203401 15.4231067 -64.5515805
C 1.0048208 13.0052398 -63.3833035
H -0.0274297 12.9715737 -63.0338871
H 1.2416141 12.0362933 -63.8251743
C 1.9196494 13.2544928 -62.2026704
C 1.5902525 14.2330469 -61.2449909
H 0.6507624 14.7645696 -61.3091331
C 2.5054578 14.5589645 -60.2283914
H 2.2645 15.3361693 -59.5185997
C 3.7477584 13.9046379 -60.163313
H 4.4610224 14.1635952 -59.3939693
C 4.075634 12.9324819 -61.1222156
H 5.0420339 12.4538226 -61.0965527
C 3.1634757 12.6011592 -62.1355357
H 3.4384687 11.8742898 -62.8856969
N 3.2219607 13.4431795 -65.6129092
H 2.7236004 12.5935768 -65.8627977

C 4.6285742 13.5613066 -66.0272132
H 5.2190656 13.6913037 -65.136371
C 4.8554892 14.8103973 -66.9071792
O 5.7538651 15.5982238 -66.6248052
C 5.1469566 12.2423806 -66.6375441
H 4.5264298 12.0049949 -67.503892
C 6.6112232 12.3569321 -67.0848273
H 7.294004 12.1712733 -66.2581363
H 6.8394987 13.3368053 -67.4929083
H 6.78462 11.6171695 -67.8603109
C 5.0988351 11.0728698 -65.6156212
H 5.8917595 11.1882859 -64.8758281
H 4.1709873 11.0890031 -65.0529447
C 5.2309691 9.6913749 -66.2729081
H 6.195366 9.5950969 -66.7695518
H 4.4343643 9.5515472 -67.0036455
H 5.1519131 8.9179254 -65.5089451
N 3.9636351 15.1292163 -67.8520424
H 3.2265652 14.4600239 -68.0547949
C 4.0218868 16.3871815 -68.6226211
H 5.0042765 16.4596209 -69.0901891
C 3.8797531 17.6361744 -67.751329
O 4.3853571 18.6900604 -68.1393617
C 2.9423549 16.3991231 -69.7126516
H 1.9845357 16.1193492 -69.2705717
H 2.8543825 17.4158555 -70.0989016
C 3.2701847 15.4763868 -70.8965743
H 3.2834725 14.434007 -70.5792135
H 4.2600221 15.7274747 -71.2810722
C 2.2462379 15.6651832 -72.0260354
H 2.5661878 15.0769262 -72.8877034
H 2.2537104 16.7118842 -72.3344896
N 0.873631 15.2917612 -71.613203
H 0.3736673 15.9475456 -71.0440993
C 0.2606047 14.1451865 -71.8617678
N 0.8324717 13.2109762 -72.562001
H 0.4215544 12.2750905 -72.7048818
H 1.7817286 13.3170273 -72.8573687
N -0.9394563 13.9204432 -71.4047869
H -1.4254517 14.5628763 -70.815964
H -1.3235025 12.9947997 -71.5411287
N 3.1468849 17.5726836 -66.640555
H 2.7147858 16.6893069 -66.3908726
C 3.0422072 18.6861717 -65.6891141
H 2.9536692 19.6115987 -66.2544648
C 4.3172776 18.8331359 -64.8542892

O 4.818539 19.9522103 -64.7316767
C 1.7891423 18.5422215 -64.8007602
H 1.0555546 17.9044931 -65.2924164
H 2.0612306 18.0606817 -63.8602092
C 1.1195113 19.8957002 -64.5028273
H 1.8473703 20.5638183 -64.0369374
H 0.3048766 19.7346467 -63.7929669
C 0.5491297 20.5310302 -65.7790337
O -0.2282403 19.8629453 -66.4986209
O 0.9457575 21.6510811 -66.1609826
N 4.94132 17.725144 -64.4234338
H 4.4998686 16.8199543 -64.5638902
C 6.2140599 17.8019982 -63.6909489
H 6.0524835 18.5815138 -62.9562327
C 7.3890477 18.3481159 -64.518059
O 8.2732855 18.9822581 -63.9445966
C 6.5604161 16.5360761 -62.8697259
H 7.6036421 16.612572 -62.5826921
C 5.687129 16.5745079 -61.5928064
H 4.6438896 16.3754152 -61.8451636
H 5.7360653 17.5498829 -61.1123979
H 6.0256541 15.83006 -60.8757306
C 6.3611885 15.1622226 -63.5084856
H 5.298497 14.9474787 -63.5301493
H 6.74844 15.1843201 -64.5161972
C 7.0610066 14.0140301 -62.7739185
H 6.6684855 13.8981371 -61.7667884
H 8.122155 14.2216184 -62.7084251
H 6.9110273 13.0840706 -63.3228865
N 7.30301 18.2991724 -65.852446
H 6.594574 17.6978391 -66.2541671
C 8.2430857 18.9945263 -66.7521705
H 9.256973 18.7089679 -66.4899572
C 8.1690656 20.51075 -66.5818082
O 9.1990222 21.1611148 -66.460308
C 7.977772 18.6399987 -68.229651
H 6.9892592 19.0220523 -68.484297
C 8.9845327 19.2914653 -69.1874602
H 10.0014391 18.9979181 -68.9194354
H 8.9126701 20.3778295 -69.1491886
H 8.7886603 18.966429 -70.2109033
C 7.96589 17.1408008 -68.5243393
H 8.943109 16.8146886 -68.8779901
H 7.2357508 16.9438971 -69.3046957
H 7.7171778 16.551408 -67.6508645
N 6.9615765 21.0893303 -66.532496

H 6.1488449 20.486938 -66.5022801
C 6.780615 22.5474602 -66.4135413
H 7.4330732 23.0541204 -67.1286945
C 7.2202667 23.0717325 -65.0569848
O 7.912679 24.0815246 -64.9821131
C 5.3159302 22.9327156 -66.6760503
H 4.6831159 22.049465 -66.5844177
H 4.9780527 23.653042 -65.9286919
C 5.1585947 23.5769347 -68.0589042
H 5.5905341 22.9257093 -68.8152424
H 5.7275276 24.5057287 -68.0837541
C 3.6905468 23.8966561 -68.3952165
H 3.6380314 24.2920585 -69.4108383
H 3.3606373 24.6917945 -67.7218206
N 2.7656957 22.7454938 -68.2527494
H 2.0283473 22.845749 -67.5579178
C 2.88091 21.5274568 -68.7454514
N 3.8693124 21.140862 -69.4991559
H 4.0949378 20.1565639 -69.4957941
H 4.5709032 21.8214323 -69.7161739
N 1.9903342 20.6334323 -68.4586807
H 1.2955849 20.8980357 -67.7519289
H 2.1477213 19.6774308 -68.6872359
N 6.8385406 22.3850171 -63.9793491
H 6.2678414 21.5594362 -64.1188139
C 7.0762139 22.8882716 -62.6154744
H 6.7536171 23.9302454 -62.5989197
C 8.5529194 22.901266 -62.209056
O 8.8936518 23.5075295 -61.1960446
C 6.2302789 22.1490035 -61.5659052
H 6.3002834 22.7158685 -60.6375336
C 4.7406745 22.1370212 -61.9312552
H 4.5608851 21.5072413 -62.7992222
H 4.4104188 23.1524728 -62.155835
H 4.1599278 21.7513496 -61.0951958
C 6.7187884 20.740197 -61.2639461
H 6.946144 20.2365441 -62.1944734
H 5.9697426 20.1820863 -60.7022476
H 7.6299397 20.7966649 -60.6732389
N 9.4330305 22.2598839 -62.9727844
H 9.1190088 21.8290885 -63.831774
C 10.8586508 22.2157185 -62.6987607
H 11.0205601 22.3896924 -61.6385775
C 11.6587618 23.2613323 -63.4943908
O 11.250341 23.686733 -64.571033
C 11.3162717 20.8126336 -63.009183

H 11.2222436 20.673128 -64.0870845
H 10.6948542 20.0962774 -62.4706286
H 12.34619 20.6925253 -62.698318
N 12.8048385 23.6814376 -62.9622398
H 13.0708624 23.3246349 -62.0518115
C 13.7495271 24.5578181 -63.6509041
H 13.1778 25.2862108 -64.2238724
C 14.6719354 23.7794981 -64.6223379
O 14.975169 22.6149417 -64.3663321
C 14.5534374 25.3133525 -62.584314
H 15.1349368 24.6050227 -61.9900518
H 13.8784704 25.8645982 -61.9278724
H 15.2402197 26.0126728 -63.0612602
N 15.2562672 24.4346856 -65.6428965
C 16.3457623 23.8565942 -66.4329207
H 15.9645131 23.0084207 -66.9972777
C 17.5084646 23.3942187 -65.5437926
O 17.9666931 24.1420647 -64.6815619
C 16.7604415 24.9539896 -67.41675
H 17.5396302 25.5804047 -66.9808329
H 17.0916402 24.5356973 -68.368371
C 15.4800201 25.7739039 -67.5748403
H 14.8102338 25.2746548 -68.2775496
H 15.6876092 26.7949285 -67.8961341
C 14.8755827 25.7311526 -66.1737705
H 13.7917232 25.8368128 -66.2352599
H 15.3069372 26.5190763 -65.555338
N 17.9761557 22.1537945 -65.7184936
H 17.5578649 21.5750566 -66.4370437
C 18.9691881 21.5330127 -64.828799
H 19.4528712 20.7173447 -65.3653614
H 19.7268063 22.271958 -64.5676816
C 18.3945524 20.9579177 -63.5231969
O 19.1431088 20.4234825 -62.6929978
N 17.0763422 20.9995032 -63.325819
H 16.4871733 21.5126475 -63.9742723
C 16.4200515 20.282351 -62.2438022
H 16.8992307 20.5608404 -61.3042546
C 16.5430015 18.7673145 -62.4295642
O 16.5979498 18.2344241 -63.5433115
C 14.9511917 20.6876016 -62.1594518
H 14.4759675 20.4721613 -63.1160417
H 14.876111 21.7519627 -61.935609
H 14.4412873 20.1255796 -61.3775577
N 16.4551063 18.0541021 -61.3125826
H 16.2855776 18.5690471 -60.4511779

C 16.3207142 16.6066863 -61.2672556
H 16.667364 16.1436285 -62.1925943
C 14.8430527 16.4876811 -61.1714432
O 14.2703098 16.4918358 -60.0760996
C 17.1025975 16.0055349 -60.0967373
H 16.7267382 16.4254048 -59.1674263
H 16.9332383 14.927969 -60.0696969
C 18.6151246 16.2789271 -60.2035818
H 18.7934009 17.3000794 -60.5397025
H 19.0498173 15.6006874 -60.9384452
C 19.3309473 16.1098317 -58.8681021
O 19.8209138 17.063498 -58.2767724
N 19.4280626 14.9016131 -58.3528361
H 19.9160454 14.8206799 -57.47876
H 19.0269415 14.1074667 -58.8221429
N 14.2900884 16.7928196 -62.3621341
H 14.9082487 16.9578584 -63.1449686
C 12.8660165 16.8921841 -62.5786515
H 12.50313 17.8129391 -62.118628
C 12.3945187 16.8195691 -64.0583579
H 11.5182164 16.1806944 -64.1205188
C 11.927267 18.2080647 -64.4477732
H 12.7140434 18.9289267 -64.2293846
H 11.0102521 18.4103486 -63.8975376
H 11.6871596 18.2879955 -65.5028421
C 13.3973882 16.3601632 -65.1198995
H 13.1242213 16.743578 -66.1014841
H 14.3632553 16.7589904 -64.8585091
C 13.4802412 14.8541873 -65.2635705
H 12.5137973 14.4524802 -65.5470749
H 13.7913259 14.4259101 -64.3196536
H 14.1809111 14.6061911 -66.0551717
C 12.2832975 15.764129 -61.7971147
O 11.2494559 16.0014477 -61.1925983
N 12.9953431 14.6160479 -61.6922139
H 13.8487719 14.4634771 -62.2140222
C 12.4409562 13.5648977 -60.9104669
H 11.9661348 14.0429408 -60.056893
C 13.3950247 12.5287825 -60.3251963
O 14.3898634 12.1321749 -60.9337501
C 11.3641755 12.9163869 -61.7760491
H 11.69877 11.9263678 -62.0871113
C 10.1448767 12.7675339 -60.8818847
H 9.7797381 13.7482455 -60.5753726
H 10.389334 12.200937 -59.985975
H 9.3515675 12.2659959 -61.4236928

C 10.8142608 13.647437 -63.0167973
H 10.2651247 14.5505195 -62.7809551
H 10.1246692 12.9691349 -63.5158578
H 11.6027534 13.8396563 -63.7244018
N 12.992268 12.0624941 -59.1407394
H 12.2290494 12.5696896 -58.7005972
C 13.3517678 10.7991302 -58.4875107
H 13.8691189 10.1454328 -59.1901295
C 12.022295 10.1409558 -58.0722694
O 11.2124517 10.7801555 -57.3905926
C 14.2732964 11.0619765 -57.2714117
H 13.7556098 11.7467959 -56.5983282
C 14.5539489 9.7552096 -56.5040559
H 15.098085 9.0547942 -57.1393494
H 13.6255968 9.2720965 -56.1977078
H 15.1326487 9.9473315 -55.6028041
C 15.5967541 11.7351388 -57.7104154
H 16.1322448 11.0671286 -58.3834904
H 15.3704105 12.6510699 -58.2557262
C 16.5299331 12.1281646 -56.5575202
H 16.9353557 11.2398739 -56.0737671
H 15.9862613 12.7290053 -55.8275692
H 17.3598219 12.7148318 -56.9523203
N 11.7580335 8.9046503 -58.5018071
H 12.4971308 8.4049395 -59.0006782
C 10.4983895 8.1867901 -58.2471085
H 9.9675434 8.6814518 -57.4396159
C 10.7986262 6.7492106 -57.8252037
O 11.102295 5.9443846 -58.7003625
C 9.5576584 8.1569015 -59.488233
H 10.0657489 7.6118162 -60.2850846
C 9.2280089 9.5527545 -60.0531933
H 8.7489912 10.1650888 -59.2892799
H 10.1684049 10.018405 -60.3410172
C 8.2538598 7.3944316 -59.1507915
H 7.707404 7.913199 -58.3643917
H 8.4738782 6.3822778 -58.8197286
H 7.6197814 7.2880813 -60.0276344
C 8.3419087 9.5094014 -61.311817
H 7.3388524 9.1658198 -61.0701747
H 8.7868381 8.8466241 -62.055071
H 8.2449684 10.4990386 -61.7479271
N 10.3778145 6.3647875 -56.6173089
H 10.0867171 7.0875804 -55.969359
C 10.0771669 4.965996 -56.2829504
H 10.6161371 4.3342597 -56.978693

C 8.5735844 4.707958 -56.4325046
O 7.7547345 5.5273144 -56.0055968
C 10.6004787 4.5910992 -54.8968489
H 10.1061673 5.1945444 -54.1366728
H 11.6735419 4.7889482 -54.8607758
O 10.3828632 3.2140938 -54.6418497
H 9.4472056 3.0366627 -54.4338089
N 8.1607001 3.6309068 -57.1055167
H 8.8769937 2.9804449 -57.4273788
C 6.7371884 3.300688 -57.3620578
H 6.1596131 3.5083897 -56.4621738
C 6.5491433 1.8064488 -57.6597888
O 7.4787363 1.1227106 -58.0834072
C 6.1822375 4.201798 -58.4902398
H 6.9381585 4.3099942 -59.2694911
C 4.8723056 3.766805 -59.1432156
H 4.1171477 3.6139869 -58.3752925
H 5.0235349 2.8460121 -59.7068042
H 4.5357146 4.5394803 -59.8340945
O 5.8902408 5.4700017 -57.9372644
H 6.6083844 5.6589023 -57.3062582
N 5.3715908 1.2506011 -57.3572631
H 4.6553821 1.8125038 -56.8983762
C 5.0533527 -0.1448405 -57.6414244
H 5.9175855 -0.7539901 -57.3692877
C 4.7686066 -0.3651727 -59.1348527
O 3.9382459 0.3149866 -59.7426248
C 3.8790949 -0.6020956 -56.7719286
H 2.9852171 -0.0491227 -57.0590648
H 4.0992285 -0.4156114 -55.7198245
H 3.7121333 -1.6687299 -56.9224242
N 5.384449 -1.4132231 -59.6710028
H 5.9615485 -1.978817 -59.0585404
C 5.274073 -1.8707675 -61.0481607
H 4.52063 -1.2819514 -61.5709594
C 4.819635 -3.3368143 -61.111532
O 4.71484 -4.0327033 -60.0985856
C 6.6290324 -1.6468094 -61.7413333
H 6.7247861 -2.2962304 -62.6122492
H 7.4480343 -1.8571985 -61.0513851
S 6.7161264 0.0744788 -62.2988248
H 5.7073454 -0.0156779 -63.1767116
N 4.5804398 -3.8163232 -62.3306187
H 4.6770828 -3.1691214 -63.1064769
C 4.346574 -5.2294842 -62.6551505
H 4.459447 -5.8264837 -61.7510928

C 5.4032849 -5.7429896 -63.6329606
O 6.1897972 -4.9572822 -64.1649364
C 2.9059161 -5.4309908 -63.1662228
H 2.7672556 -6.4849372 -63.4143421
H 2.2061259 -5.1920836 -62.3647088
C 2.5629917 -4.5759187 -64.3962885
H 3.3538419 -4.6507815 -65.1381943
H 2.4774119 -3.5317996 -64.1082129
C 1.239358 -5.0199314 -65.0200982
H 0.4409992 -4.8935195 -64.2851521
H 1.2911458 -6.0804497 -65.2787751
N 0.9258565 -4.2126199 -66.212061
H 0.4081314 -3.3590145 -66.0351975
C 1.2259418 -4.5210722 -67.4631425
N 1.9688723 -5.5468659 -67.7776155
H 2.1152903 -5.8721481 -68.7219343
H 2.3570204 -6.1389378 -67.0480665
N 0.7801215 -3.779182 -68.4391839
H 0.1596895 -3.0162654 -68.2328983
H 0.9754474 -4.0537471 -69.3841574
N 5.3899017 -7.0384504 -63.929528
H 4.7383203 -7.6344439 -63.4363782
C 6.0942102 -7.5596355 -65.1036889
H 7.0977374 -7.1300138 -65.1006469
C 5.4232967 -7.0925274 -66.4226341
O 4.3354509 -6.5106139 -66.4170726
C 6.2677109 -9.0838745 -64.9594063
H 6.6936781 -9.2945219 -63.9792089
H 6.9785677 -9.4402086 -65.7029615
C 4.9912565 -9.885612 -65.1365704
O 4.2025192 -9.6370408 -66.0222303
N 4.7808317 -10.9165378 -64.3629691
H 3.901636 -11.390675 -64.4666077
H 5.4341878 -11.1196392 -63.6117621
N 6.087026 -7.301176 -67.5654737
H 6.97492 -7.7768733 -67.5279906
C 5.5686416 -6.8960675 -68.8824775
H 5.3268371 -5.8354456 -68.847767
C 4.2717675 -7.636028 -69.2757134
O 3.3960169 -7.0361793 -69.9063768
C 6.6966829 -7.1057223 -69.9118019
H 7.5465829 -6.4850685 -69.622889
H 7.0144693 -8.1489414 -69.8602459
C 6.3428838 -6.7968035 -71.3816833
H 5.5274226 -7.4439008 -71.7033055
C 5.9359276 -5.3387014 -71.5982807

H 6.7312629 -4.6712647 -71.263949
H 5.0185384 -5.1252485 -71.0539862
H 5.7487945 -5.1681951 -72.6580208
C 7.5553389 -7.0841982 -72.2684394
H 8.3866897 -6.4341584 -71.9947723
H 7.2949337 -6.9136733 -73.313153
H 7.8578121 -8.1251123 -72.1530906
N 4.1334798 -8.8979656 -68.8636108
H 4.8250579 -9.2749777 -68.2335885
C 3.0282568 -9.7990911 -69.2066128
H 2.994302 -9.8630138 -70.2915907
C 1.6602333 -9.3119172 -68.6927965
O 1.5945623 -8.516802 -67.7502102
C 3.3072741 -11.2092038 -68.6610331
H 2.5364612 -11.8929918 -69.0166306
H 3.2739869 -11.2045757 -67.5732222
O 4.5717364 -11.6737482 -69.1032695
H 4.7435367 -12.5260357 -68.6915725
N 0.5433211 -9.7783737 -69.2905212
C -0.7985269 -9.4881579 -68.7920022
H -0.974344 -8.4156383 -68.878059
C -0.9754439 -9.9212677 -67.3331253
O -0.527133 -10.9988821 -66.9390965
C -1.7659872 -10.2366409 -69.7171588
H -1.976375 -11.2318292 -69.3196156
H -2.693753 -9.6827823 -69.8650191
C 0.4554123 -10.5666366 -70.5124142
H 1.1583618 -10.2258453 -71.2721388
H 0.6242898 -11.6193873 -70.280253
C -0.973233 -10.3730318 -71.0152853
H -1.0393598 -9.4455678 -71.586019
H -1.3115491 -11.2200922 -71.6130074
N -1.6717958 -9.0991233 -66.547554
H -1.9774122 -8.2113845 -66.9104634
C -1.9426282 -9.3712957 -65.1363939
H -0.9869589 -9.4906116 -64.6280691
C -2.7599268 -10.6578647 -64.9656591
O -3.8400503 -10.7898855 -65.5414905
C -2.701309 -8.1901542 -64.5041285
H -2.7718945 -8.3380187 -63.4261289
H -3.7101012 -8.1606276 -64.9151862
O -2.0772418 -6.945159 -64.7763668
H -2.4140318 -6.2669389 -64.1668781
N -2.292544 -11.5662814 -64.1043462
H -1.3627723 -11.44079 -63.7365719
C -3.0207712 -12.7921241 -63.7457168

H -3.2244875 -13.323451 -64.6781217
C -4.3966937 -12.4684447 -63.1343671
O -4.6230048 -11.3825377 -62.579092
C -2.1096583 -13.72106 -62.9038127
H -1.910148 -14.6234283 -63.4834728
H -1.1418491 -13.2284864 -62.8004951
C -2.5148567 -14.1252411 -61.4840284
H -2.7946617 -13.2267165 -60.9280213
H -1.6416138 -14.553518 -60.9864657
C -3.6505368 -15.1492933 -61.4302847
O -4.2452488 -15.22482 -60.332582
O -4.0275463 -15.7087837 -62.4791995
N -5.3275999 -13.4242844 -63.2208885
H -5.0081732 -14.3620026 -63.4746751
C -6.7373853 -13.2558538 -62.8708644
H -7.1275333 -12.4809128 -63.5271843
C -6.946757 -12.7679238 -61.4410492
O -7.9117529 -12.0322895 -61.2615722
C -7.4812086 -14.5788163 -63.1367407
H -7.2969487 -14.8900304 -64.1668939
H -7.0568127 -15.3459934 -62.4846586
C -8.9995942 -14.5392484 -62.9048429
H -9.3885239 -15.5543231 -62.9959171
H -9.1947796 -14.2219061 -61.8805407
C -9.7553918 -13.6445641 -63.9063237
H -10.1326223 -14.2766743 -64.7127237
H -9.0952164 -12.9031723 -64.3565096
C -10.9343519 -12.935928 -63.2296966
H -11.4774207 -13.6713244 -62.6279936
H -11.6147183 -12.563259 -64.0023007
N -10.4659423 -11.8111498 -62.3804404
H -9.7675482 -12.1340372 -61.710333
H -11.2343898 -11.3862407 -61.8810379
H -10.0067443 -11.1094506 -62.9439105
N -6.0816248 -13.0886981 -60.4718269
H -5.3969747 -13.8110332 -60.6825751
C -6.0968226 -12.5397581 -59.1017552
H -7.1172031 -12.2357542 -58.8791371
C -5.2212816 -11.2922417 -58.8815129
O -5.4720267 -10.5585664 -57.9262851
C -5.7549487 -13.6167123 -58.0640669
H -6.3952429 -14.4869167 -58.2181221
H -5.9482444 -13.2182487 -57.0671658
O -4.3992676 -14.0098058 -58.1432093
H -4.343102 -14.5866227 -58.960205
N -4.2784466 -10.9636136 -59.7796263

H -4.191226 -11.5356199 -60.6116894
C -3.5645761 -9.675878 -59.7459851
H -3.2982635 -9.4612025 -58.710529
C -4.4764878 -8.5391038 -60.2170946
O -4.6424323 -7.5619692 -59.492832
C -2.2637066 -9.7208699 -60.5782299
H -1.5860132 -10.4576694 -60.1459091
H -2.5060962 -10.0411425 -61.5913691
C -1.54592 -8.348949 -60.6594275
H -2.2252017 -7.6004366 -61.0640231
C -1.049715 -7.869789 -59.2926931
H -0.3925492 -8.6162577 -58.8481829
H -1.895953 -7.6778135 -58.6345231
H -0.5004847 -6.9352805 -59.4146506
C -0.3461376 -8.4080241 -61.5969519
H 0.3865714 -9.1103831 -61.226032
H 0.1108929 -7.4212003 -61.6794127
H -0.6523785 -8.7230191 -62.5882788
N -5.1169134 -8.689943 -61.3849318
H -4.9457027 -9.5459942 -61.90868
C -6.0412299 -7.6860189 -61.9416269
H -5.4551489 -6.7857532 -62.1437637
C -7.1240799 -7.231914 -60.9375162
O -7.2297424 -6.0346225 -60.7273625
C -6.5934143 -8.1928127 -63.2907024
H -5.7661369 -8.2380254 -63.9984981
H -6.9541082 -9.2147834 -63.16253
C -7.7329938 -7.3712305 -63.9310273
H -7.8971692 -7.7500898 -64.9408023
H -8.6442331 -7.5659483 -63.3636415
C -7.5575872 -5.8442729 -64.0094301
H -7.4980538 -5.4352241 -63.0077227
H -8.4615509 -5.4163366 -64.4482493
C -6.3614685 -5.3390559 -64.8205827
H -6.662531 -5.2804452 -65.8695286
H -5.5195257 -6.0309988 -64.7300999
N -5.9571078 -4.0034871 -64.3263948
H -6.7679802 -3.395136 -64.17414
H -5.2630821 -3.5587778 -64.9038722
H -5.4837897 -4.0957187 -63.4295262
N -7.871645 -8.0931999 -60.2239082
C -8.873936 -7.6616363 -59.2573345
H -9.5234761 -6.9139405 -59.7159941
C -8.2649088 -7.0320422 -57.9992117
O -8.9337415 -6.198365 -57.3998078
C -9.7099499 -8.8997193 -58.9176722

H -10.0869565 -8.8805224 -57.8938592
H -10.5345133 -8.9914458 -59.6264198
C -7.9351771 -9.5147877 -60.3439147
H -8.4774898 -9.7379089 -61.262144
H -6.9490279 -9.9651637 -60.3563997
C -8.720291 -10.0246495 -59.1344312
H -9.2281338 -10.9685741 -59.3297095
H -8.0659944 -10.10908 -58.2666461
N -7.021786 -7.3606245 -57.6043131
H -6.4998668 -8.0322734 -58.1535313
C -6.3056253 -6.6353359 -56.5348897
H -6.9728801 -6.5111878 -55.6824392
C -5.9322881 -5.2270172 -57.0036012
O -6.1602659 -4.2700824 -56.2681912
C -5.0687729 -7.4390979 -56.0860549
H -5.3895788 -8.424192 -55.7418211
H -4.4305065 -7.5948128 -56.9556649
C -4.1992388 -6.7757217 -54.993123
H -3.2115868 -7.2341931 -55.0481208
H -4.0668317 -5.7140503 -55.2046993
C -4.7169933 -6.9550987 -53.5545555
H -4.8133604 -8.0239603 -53.351868
H -5.6999526 -6.4874112 -53.4551922
C -3.7314371 -6.3211879 -52.5553124
H -3.9467867 -5.2535484 -52.4822222
H -2.7197203 -6.4281609 -52.9491867
N -3.7880118 -6.9492504 -51.2117734
H -4.7008678 -6.8646728 -50.7950431
H -3.1294465 -6.5095671 -50.5629086
H -3.5314121 -7.9249485 -51.2652042
N -5.4101965 -5.0928218 -58.2204721
H -5.2513331 -5.9347831 -58.7634563
C -5.1139113 -3.8079675 -58.8626221
H -4.4172266 -3.2441876 -58.2414818
C -6.3827766 -2.9486339 -58.9977917
O -6.424878 -1.8461333 -58.4601641
C -4.4306352 -4.0925634 -60.2147685
H -3.4889227 -4.612916 -60.0251308
H -5.0681263 -4.750292 -60.8032861
C -4.1402352 -2.8329003 -61.0402102
H -3.5223312 -2.1630568 -60.4391233
H -5.0881717 -2.3339607 -61.2560169
C -3.4537445 -3.1310734 -62.3813936
O -3.6879035 -4.2298107 -62.9487496
O -2.7668995 -2.224337 -62.9038172
N -7.4664462 -3.5002571 -59.5499567

H -7.3734285 -4.4240226 -59.9575726
C -8.7116426 -2.7639545 -59.7964255
H -8.4353106 -1.8095062 -60.2480485
C -9.4638687 -2.4089424 -58.5074308
O -9.9528278 -1.288221 -58.3755535
C -9.6118555 -3.5466591 -60.7821039
H -9.0607949 -4.3693492 -61.2330581
H -10.4668038 -3.9705644 -60.2532929
C -10.1111674 -2.6392604 -61.9230753
H -10.7214885 -1.8416841 -61.4932013
H -10.7558622 -3.2309231 -62.5768496
C -8.9667387 -2.0313695 -62.7636765
O -8.2371866 -2.7994259 -63.4391515
O -8.8066446 -0.7847794 -62.7441526
N -9.47042 -3.2962327 -57.4999447
H -9.0417097 -4.20374 -57.6524768
C -10.1010431 -3.0170332 -56.1973756
H -11.0288266 -2.4764864 -56.3973745
C -9.2769901 -2.0761537 -55.3142795
O -9.8498703 -1.4157787 -54.4554675
C -10.4660358 -4.334596 -55.4944689
H -11.0058283 -4.9560219 -56.2112972
H -9.5566922 -4.8544857 -55.1890845
C -11.3704398 -4.1053174 -54.2696322
H -10.7558442 -3.807233 -53.4188719
H -12.0692675 -3.2953097 -54.4855386
C -12.2007088 -5.3376816 -53.8874421
H -12.8839548 -5.5640901 -54.7082912
H -12.7938671 -5.0865768 -53.0059178
C -11.3301939 -6.5636396 -53.5898349
H -10.6293076 -6.3072624 -52.7892185
H -10.7480368 -6.8020651 -54.4859351
N -12.1634946 -7.726805 -53.1936491
H -12.701381 -7.5095402 -52.3633894
H -11.5843428 -8.534113 -52.9993255
H -12.8073944 -7.9650457 -53.9371857
N -7.9716141 -1.9444161 -55.5661454
H -7.5758423 -2.5040646 -56.3110106
C -7.1488087 -0.8533673 -55.0040867
H -7.6222074 -0.5067725 -54.0856447
C -7.1064176 0.3871534 -55.9041949
O -6.4532318 1.363562 -55.5514579
C -5.7327836 -1.3132419 -54.6059369
H -5.1704231 -0.4493004 -54.2512706
C -5.7686133 -2.3392788 -53.4714731
H -6.3200593 -3.2274988 -53.7777969

H -6.2540956 -1.8980821 -52.6008782
H -4.7497511 -2.6174525 -53.2046511
O -5.0121386 -1.9015539 -55.6648057
H -5.5081724 -2.690785 -55.9458878
N -7.8166632 0.3654646 -57.0417343
H -8.3462446 -0.4726061 -57.2427193
C -7.8502309 1.3899778 -58.0923619
H -8.3461036 0.901136 -58.9292334
C -6.4594769 1.7892235 -58.633805
O -6.3001095 2.8344891 -59.2640218
C -8.7526115 2.5574542 -57.6322694
H -9.6187161 2.1305136 -57.1235023
H -8.1999693 3.1478449 -56.8997247
C -9.2803135 3.5032468 -58.7320415
H -8.4579137 4.0436594 -59.1952806
C -10.0659324 2.771923 -59.8264709
H -10.8596408 2.1741863 -59.3761475
H -9.4021933 2.1159166 -60.3858728
H -10.5024011 3.4948423 -60.5147045
C -10.2132689 4.5376623 -58.0986059
H -11.0746174 4.0426197 -57.649767
H -10.5543428 5.2422816 -58.85601
H -9.6730952 5.0878871 -57.3275553
N -5.4426723 0.9434196 -58.4481881
H -5.6678236 0.0355238 -58.0548346
C -4.0798935 1.185912 -58.9357536
H -3.8150141 2.2211407 -58.7113507
C -4.0051982 1.032849 -60.4518383
O -4.7678273 0.27695 -61.0434649
C -3.0949649 0.2568287 -58.2130025
H -3.5138127 -0.7490093 -58.1422002
H -2.1678002 0.2159683 -58.784929
C -2.795052 0.8141 -56.8097441
H -2.3119699 1.7866437 -56.9208895
H -3.7301621 0.9656837 -56.2690902
C -1.8893985 -0.0773955 -55.9502131
H -2.4545609 -0.9480272 -55.6154601
H -1.5950424 0.5033683 -55.0736083
C -0.6439782 -0.5463608 -56.7108241
H -0.3018319 0.268945 -57.3493463
H -0.9215663 -1.3830817 -57.3566641
N 0.4467047 -0.9339892 -55.7916971
H 0.7465728 -0.112789 -55.2498367
H 1.2748543 -1.2105249 -56.2979961
H 0.1611309 -1.6486113 -55.1440047
N -3.0487572 1.7262357 -61.0774219

H -2.4915023 2.3509615 -60.5015827
C -2.7384924 1.6213578 -62.5167704
H -3.2764944 0.767828 -62.9341887
C -1.2710996 1.2723612 -62.7035448
O -0.4066791 2.1504201 -62.6343866
C -3.1835379 2.9095297 -63.2545982
H -3.4195988 3.6894009 -62.5300726
H -2.3764105 3.2816492 -63.8842477
C -4.4157079 2.6815394 -64.1627795
H -4.7723658 3.6569256 -64.4963477
H -4.1117891 2.1108096 -65.0407656
C -5.5713926 1.9356428 -63.4710507
H -5.670715 2.3288555 -62.460506
H -5.3424209 0.8725846 -63.4125411
C -6.9189723 2.0674559 -64.1837241
H -6.9312356 1.427326 -65.0694601
H -7.0421595 3.108668 -64.4916204
N -8.0097774 1.7033435 -63.2510456
H -7.9605382 0.719054 -62.960874
H -8.919042 1.7965361 -63.6756161
H -7.9574248 2.2846703 -62.4272009
N -0.9851324 -0.0152724 -62.8685472
H -1.7507671 -0.6921228 -62.9343551
C 0.3622951 -0.5630426 -62.7563147
H 0.9805693 0.166144 -62.2307181
C 0.9623679 -0.7635467 -64.1569443
O 0.4536773 -1.5034089 -65.0035394
C 0.3662204 -1.8384641 -61.8795023
H -0.0630003 -2.6610733 -62.4548948
C 1.819163 -2.1735927 -61.5191769
H 2.2062048 -1.4454116 -60.8056847
H 2.4410637 -2.152216 -62.4072739
H 1.8767011 -3.1699076 -61.08193
C -0.4305112 -1.6738873 -60.5577608
H 0.0351023 -0.9039866 -59.9410057
H -1.442414 -1.3440835 -60.7786126
C -0.5557201 -2.9669112 -59.7395202
H 0.4082644 -3.2467547 -59.3150915
H -0.9233073 -3.7726035 -60.3764866
H -1.265722 -2.8133331 -58.927048
N 2.0820627 -0.0851478 -64.4029156
H 2.4655832 0.475942 -63.6575331
C 2.9068083 -0.2770199 -65.5962123
H 2.3018658 -0.6952979 -66.4020926
C 4.0396154 -1.2643317 -65.292497
O 4.4307098 -1.4269569 -64.1332412

C 3.4495725 1.0814523 -66.061202
H 4.0958151 0.9525892 -66.9322466
H 4.0432311 1.5292122 -65.261646
S 2.0709612 2.1846743 -66.4876026
H 1.3003227 1.8921115 -65.4342484
N 4.6448883 -1.8495044 -66.3268338
H 4.3466771 -1.6176306 -67.261478
C 5.9932657 -2.3856146 -66.1595621
H 6.0491473 -2.8706147 -65.1886549
C 7.0088041 -1.2347704 -66.1249314
O 6.737833 -0.132612 -66.6086652
C 6.3073177 -3.4524623 -67.2163512
H 5.4957053 -4.1766962 -67.2517385
H 7.2191217 -3.9815768 -66.9375472
C 6.5230252 -2.8364934 -68.5773457
O 7.5678347 -2.280934 -68.8635157
N 5.5086591 -2.8222407 -69.403895
H 5.6807276 -2.3748843 -70.3032807
H 4.6772054 -3.3460722 -69.2191437
N 8.1801732 -1.5131329 -65.5618081
H 8.3470506 -2.4508453 -65.2320084
C 9.2452561 -0.5331003 -65.3748175
H 8.8589516 0.286887 -64.7647094
C 9.6957767 0.098359 -66.7072817
O 9.7789872 1.3222783 -66.8014925
C 10.3873618 -1.2029495 -64.5780293
H 11.2574185 -0.549599 -64.5671677
H 10.0590078 -1.2929866 -63.5414857
C 10.8177317 -2.5865022 -65.0490904
C 11.9181375 -2.7365552 -65.9169673
H 12.4819757 -1.8755661 -66.2390297
C 12.3053206 -4.0136478 -66.363435
H 13.1452796 -4.1187607 -67.0335377
C 11.597087 -5.1547648 -65.9319127
O 11.9592972 -6.3881262 -66.3699191
H 12.7274721 -6.3595775 -66.9421564
C 10.5129368 -5.0118722 -65.040923
H 9.9908157 -5.8930459 -64.6965091
C 10.1282528 -3.7322316 -64.5973171
H 9.308215 -3.6379075 -63.8983316
N 9.914116 -0.6953223 -67.7660937
H 9.7185 -1.6813804 -67.6780474
C 10.4258322 -0.1969978 -69.0526211
H 11.3531195 0.3334907 -68.8684677
C 9.4744436 0.8147866 -69.7068052
O 9.9050969 1.903903 -70.0918449

C 10.7555701 -1.3866897 -69.9704476
H 11.4799712 -2.0264374 -69.4643361
H 9.8468928 -1.968167 -70.1290859
C 11.319076 -0.9912917 -71.3499645
H 10.5886703 -0.3825456 -71.8825983
C 12.633967 -0.2142643 -71.2488822
H 13.3625122 -0.779567 -70.6682336
H 12.462991 0.7532524 -70.7772734
H 13.0319279 -0.0350601 -72.2476385
C 11.5735498 -2.2547509 -72.1719436
H 12.3160219 -2.8816896 -71.6783682
H 11.9344282 -1.9816748 -73.1636924
H 10.6437644 -2.8131246 -72.2827093
N 8.1721243 0.5219023 -69.7300654
H 7.8781855 -0.3931025 -69.4008782
C 7.1627271 1.4694778 -70.216607
H 7.465206 1.8258281 -71.2019721
C 7.0485684 2.7218684 -69.344085
O 6.7977963 3.7974389 -69.8845881
C 5.8037654 0.7721156 -70.3591403
H 5.0142317 1.5249066 -70.3314356
H 5.6424419 0.0910384 -69.5229777
C 5.6722935 0.0300481 -71.6647418
N 5.9532087 -1.3209948 -71.8725283
C 5.7499517 -1.545272 -73.178947
H 5.897551 -2.4993019 -73.6695688
N 5.3500598 -0.4176791 -73.790252
H 5.1471925 -0.3273254 -74.7758818
C 5.2980715 0.5887433 -72.851667
H 5.0341347 1.6258243 -73.0140616
N 7.3164396 2.6332608 -68.0360767
H 7.5599259 1.7314697 -67.6420576
C 7.2614763 3.7968527 -67.1509479
H 6.3620564 4.3393293 -67.4291629
C 8.4261241 4.7738874 -67.3880553
O 8.1695259 5.9696373 -67.5199989
C 7.0948289 3.3363918 -65.6891448
H 6.3288467 2.5601209 -65.6683109
H 8.0271337 2.8928056 -65.3390609
C 6.6681347 4.4549309 -64.7117129
H 7.5189438 5.109165 -64.5258907
C 5.4997377 5.3156355 -65.2074584
H 4.6757884 4.679795 -65.5306189
H 5.8301918 5.9439793 -66.0348046
H 5.1677863 5.9791257 -64.4099764
C 6.2145039 3.8387536 -63.3851094

H 5.3016056 3.2618588 -63.5260214
H 6.0445249 4.6237662 -62.6487135
H 6.9882306 3.1722436 -63.0041928
N 9.6480168 4.2850584 -67.6533181
H 9.7997559 3.2846604 -67.5630426
C 10.7262591 5.1507473 -68.1801541
H 10.8557976 5.9933419 -67.499146
C 10.3952471 5.6900104 -69.5631661
O 10.7418516 6.824877 -69.8713061
C 12.0605472 4.4240518 -68.3016877
H 12.1166467 3.8610945 -69.2350101
H 12.121121 3.7293563 -67.4803064
O 13.1491082 5.3247477 -68.1948499
H 13.269569 5.8510611 -69.0056855
N 9.6793356 4.9112594 -70.3797769
H 9.4739327 3.9631262 -70.084374
C 9.1529553 5.3651188 -71.6612905
H 9.9800387 5.6169965 -72.3253587
H 8.5721268 4.5597261 -72.1096179
C 8.2436502 6.5889815 -71.5206075
O 8.4964877 7.5881455 -72.1829673
N 7.271716 6.5802667 -70.6017955
H 7.1008963 5.7179054 -70.0934582
C 6.3753098 7.7248641 -70.3564864
H 5.913338 8.0175862 -71.2986071
C 7.1064366 8.967594 -69.8279953
O 6.7901523 10.0960769 -70.2162708
C 5.2706065 7.3106618 -69.3736671
H 5.7289851 6.9026424 -68.4718638
H 4.7203521 8.2063148 -69.0832355
C 4.2527546 6.3259864 -69.9274463
C 3.9569052 5.1377987 -69.2312677
H 4.470828 4.90886 -68.3126257
C 2.9868958 4.2463839 -69.7247382
H 2.7656118 3.3367646 -69.1875547
C 2.2932897 4.5473782 -70.9100298
H 1.5351606 3.8746796 -71.2849157
C 2.5749518 5.7375725 -71.6014384
H 2.0297721 5.995317 -72.5016579
C 3.5527959 6.6201892 -71.1146242
H 3.7383022 7.5375435 -71.6559592
N 8.1333138 8.7500029 -69.0098282
H 8.2811018 7.8069615 -68.6678179
C 9.07562 9.7716512 -68.5730746
H 8.5547353 10.5922679 -68.0912188
C 9.8605926 10.3853852 -69.7513712

O 9.7566054 11.5847949 -70.0065046
C 9.9597189 9.1076336 -67.5219712
H 10.8339721 9.7058895 -67.3759088
H 10.2895408 8.1279684 -67.8552867
S 9.0625811 8.9471298 -65.9503501
H 9.8351265 9.8474364 -65.3019252
N 10.4805271 9.5504548 -70.5898632
H 10.5030738 8.5688311 -70.3340113
C 11.2121939 9.9579073 -71.8004392
H 12.0155281 10.6371849 -71.5108146
C 10.3189662 10.6842638 -72.8220458
O 10.6706195 11.7496569 -73.3218699
C 11.8287769 8.7012708 -72.4308687
H 11.0787146 8.1829872 -73.0301747
H 12.1581597 8.0264929 -71.641054
O 12.9535704 9.016732 -73.2287619
H 13.2151123 8.2260534 -73.7115009
N 9.0917743 10.1931924 -73.0341901
H 8.8462209 9.3288767 -72.56349
C 8.0758771 10.7997598 -73.908273
H 8.5230395 10.9933282 -74.8827677
C 7.5601152 12.1572996 -73.4059811
O 6.9709603 12.9057875 -74.1813275
C 6.8980951 9.8177845 -74.0665242
H 6.5963806 9.4797408 -73.0746319
H 6.0469815 10.3468555 -74.4990079
C 7.2010356 8.596359 -74.9559661
H 8.1359891 8.1311667 -74.6495739
C 6.078985 7.5667695 -74.8172612
H 5.1265064 8.0041828 -75.1217947
H 6.002105 7.244648 -73.7790986
H 6.2918725 6.6974123 -75.4373065
C 7.3195826 8.9722824 -76.4354788
H 6.4050387 9.4644876 -76.7684964
H 7.4802548 8.0744735 -77.031481
H 8.1643764 9.6422728 -76.586479
N 7.8083199 12.5056056 -72.1428918
H 8.3267403 11.8644839 -71.5540116
C 7.4700463 13.8201725 -71.5844624
H 6.6249218 14.2248713 -72.140381
C 8.5965015 14.8537852 -71.7283394
O 8.4412966 15.9715581 -71.2569983
C 7.0096966 13.6702722 -70.1392581
H 6.7278152 14.646009 -69.7435907
H 7.8241777 13.2648047 -69.5378654
O 5.8818799 12.8084382 -70.0899695

H 6.1934362 11.8939971 -70.2199382
N 9.6527789 14.5194905 -72.4726668
H 9.6609405 13.583641 -72.8564542
C 10.8450282 15.3307806 -72.7823003
H 11.3215018 14.7681748 -73.5857787
C 11.9223128 15.3449434 -71.6844862
O 12.7208264 16.2703293 -71.5460031
C 10.4885248 16.6983789 -73.4123986
H 10.3052822 17.4216129 -72.6154718
H 9.5630553 16.5886969 -73.9810148
C 11.5483248 17.2610639 -74.3780375
O 12.3958398 16.4764423 -74.8659835
O 11.4371665 18.4657644 -74.7049311
N 11.9834183 14.26542 -70.8998863
H 11.310854 13.5258562 -71.0438426
C 13.0028759 14.0912496 -69.8714545
H 13.306885 15.0728525 -69.5156226
C 14.2829326 13.4339092 -70.3807713
O 14.2769208 12.3465484 -70.963321
C 12.4106606 13.3349922 -68.6923727
H 12.0504773 12.3665639 -69.0383334
H 13.215576 13.1470367 -67.9823596
C 11.3089208 14.0511588 -67.9372688
C 10.4143586 13.2895275 -67.1652494
H 10.4207411 12.2129036 -67.2452246
C 9.622269 13.9020251 -66.183757
H 9.0420531 13.2912381 -65.5123696
C 9.6896914 15.2939169 -66.0117228
O 9.0688287 15.8810816 -64.9659394
H 8.6517885 15.2197967 -64.4135997
C 10.476267 16.0688853 -66.8777947
H 10.5008894 17.1334103 -66.7732363
C 11.2869235 15.4568867 -67.8389113
H 11.9309688 16.084664 -68.4366982
N 15.413457 14.0666489 -70.0648519
H 15.3470006 14.8954175 -69.4865486
C 16.7466287 13.6072225 -70.4479379
H 16.6439033 13.0148765 -71.3578561
C 17.3845581 12.7089696 -69.3861672
O 17.0498046 12.785335 -68.2019845
C 17.6340751 14.8021464 -70.8021027
H 17.0888067 15.4656922 -71.4759625
H 18.5250258 14.4458703 -71.3186039
O 18.0387931 15.5263655 -69.6592734
H 17.3011331 15.5682376 -69.0191956
N 18.3085085 11.8526167 -69.839663

H 18.540374 11.8840546 -70.8182419
C 19.0346307 10.8940314 -69.0057114
H 19.4675616 10.1472293 -69.6713046
C 18.1121144 10.1118048 -68.0476025
O 18.3664568 10.0593789 -66.8463913
C 20.2060694 11.6112569 -68.3167241
H 19.8233584 12.3411857 -67.6012615
H 20.822286 12.1241624 -69.055329
H 20.8190484 10.8836954 -67.7848943
N 17.0082147 9.5547067 -68.5680509
H 16.8205954 9.6340163 -69.5538031
C 16.1258953 8.7216485 -67.7571872
H 16.0455573 9.2076642 -66.7871337
C 16.7726785 7.3640399 -67.4612841
O 17.037384 6.5613184 -68.3551552
C 14.6792575 8.6592675 -68.293561
H 14.2122908 9.6269136 -68.1101076
H 14.1203773 7.931987 -67.704949
C 14.479784 8.3334479 -69.7606206
O 14.1690558 7.2130685 -70.1375173
N 14.5042034 9.3331716 -70.6191546
H 14.1908225 9.1433714 -71.5603483
H 14.5384129 10.2922772 -70.2963576
N 17.0392665 7.1424113 -66.177116
H 16.830673 7.8928146 -65.5251381
C 17.5157217 5.8978433 -65.5954028
H 17.9177789 5.2616605 -66.384763
C 16.3357946 5.207524 -64.9288189
O 15.6159122 5.8225069 -64.1466295
C 18.6212937 6.1737034 -64.5672921
H 18.1711266 6.6156535 -63.6769974
H 19.0702786 5.2249355 -64.2716113
C 19.7093446 7.0913046 -65.0380035
C 19.7931824 8.4044157 -64.7311366
H 19.0720161 8.9404149 -64.124824
N 20.8504143 8.9783652 -65.4059943
H 20.9953366 9.9773354 -65.4322483
C 21.4912532 8.0630289 -66.2111008
C 22.5607112 8.1678796 -67.1126483
H 23.0547657 9.1163029 -67.2628505
C 22.976687 7.0234162 -67.8144749
H 23.7994497 7.0845023 -68.5131482
C 22.3164877 5.7977862 -67.6094385
H 22.6337933 4.9207785 -68.1562422
C 21.2368826 5.7069224 -66.707767
H 20.7248034 4.766601 -66.5741431

C 20.7952953 6.8365602 -65.9837824
N 16.1848563 3.912817 -65.176291
H 16.821799 3.469261 -65.8205527
C 15.0796393 3.1104436 -64.6775838
H 14.54308 3.6333506 -63.8833365
C 15.6247004 1.7985474 -64.1325112
O 16.0577958 0.9402323 -64.9020713
C 14.131694 2.8887033 -65.8487103
H 13.64995 3.8395378 -66.0623428
H 14.7289034 2.6128478 -66.7202805
C 13.0901348 1.8002141 -65.5654381
H 13.4487714 1.0489044 -64.8679001
C 11.8698551 2.4330043 -64.9143906
H 11.3898081 3.1481945 -65.575671
H 12.1699034 2.9421886 -63.9979327
H 11.1508884 1.6662489 -64.6483251
C 12.8433832 1.0456084 -66.8611406
H 12.3756177 1.6885177 -67.5980209
H 12.2143535 0.1981272 -66.6495824
H 13.7858171 0.6635186 -67.2549475
N 15.5022455 1.6123315 -62.8227831
H 15.0176951 2.3212374 -62.2749974
C 15.9819101 0.4156113 -62.1354336
H 16.5767406 -0.1688088 -62.8309248
C 14.7948239 -0.412656 -61.638592
O 14.0877462 0.0314062 -60.7298387
C 16.9228566 0.7713672 -60.9737509
H 16.3487296 1.1324943 -60.119959
C 17.7625409 -0.435787 -60.5611816
H 18.3692173 -0.7740933 -61.4018301
H 17.1180753 -1.2500821 -60.2345302
H 18.4224701 -0.1512396 -59.7425426
O 17.83844 1.7718527 -61.3585353
H 17.3314862 2.5714806 -61.5236629
N 14.5757734 -1.6381667 -62.1522123
C 13.7832369 -2.6243789 -61.4326093
H 12.8078798 -2.2156713 -61.1651937
C 14.5459745 -3.0172483 -60.1610187
O 15.6270337 -3.5983735 -60.2304277
C 13.5987399 -3.7952014 -62.405327
H 13.5382697 -4.7567764 -61.8952256
H 12.6985773 -3.6284725 -62.9974839
C 15.1494966 -2.2202696 -63.3610773
H 14.6833669 -1.7644417 -64.236083
H 16.2312348 -2.0961457 -63.4033807
C 14.823558 -3.7137458 -63.3158808

H 14.6132543 -4.1093829 -64.3096971
H 15.6551597 -4.2543635 -62.8618106
N 13.9662979 -2.746838 -58.9908767
H 13.0661721 -2.2754632 -59.0090904
C 14.5182136 -3.1102436 -57.6789229
H 15.4998097 -3.5591397 -57.8229977
C 13.7014467 -4.2044761 -56.9428997
O 13.6238671 -4.1604091 -55.7146881
C 14.714941 -1.81596 -56.8514598
H 13.7756341 -1.2643591 -56.8431172
H 14.9534945 -2.0737327 -55.819355
C 15.8229594 -0.873091 -57.3541501
H 15.6404761 -0.6146515 -58.3959849
C 15.8120489 0.4127376 -56.5303236
H 16.028931 0.1980959 -55.4826737
H 14.8309305 0.8833728 -56.593059
H 16.5554212 1.1109018 -56.9145674
C 17.2136708 -1.5018124 -57.2217831
H 17.3755269 -1.8389114 -56.1975999
H 17.9750578 -0.7631874 -57.470298
H 17.3151205 -2.3433035 -57.9050428
N 13.0948036 -5.214763 -57.6151661
C 12.2053006 -6.1721577 -56.9514439
H 11.3888186 -5.644905 -56.4617025
C 12.9249789 -7.0386965 -55.9075064
O 12.3195788 -7.4085346 -54.9111168
C 11.6162382 -7.0363963 -58.0682371
H 11.3764525 -8.0460584 -57.7312578
H 10.7270492 -6.5549761 -58.4702904
C 13.2765494 -5.6211828 -59.0043124
H 12.7053484 -4.9577645 -59.6524598
H 14.33053 -5.6213029 -59.2835882
C 12.7120005 -7.0340446 -59.1282169
H 12.3119336 -7.2235629 -60.1252091
H 13.4796364 -7.7662537 -58.8743209
N 14.2193922 -7.3309605 -56.0875591
H 14.6758111 -6.9951267 -56.9187628
C 15.0103271 -8.0990672 -55.112049
H 14.434692 -8.9910839 -54.8655987
C 15.2070223 -7.332927 -53.7964256
O 15.0766997 -7.9116657 -52.717904
C 16.3497863 -8.552893 -55.7413196
H 16.1067111 -9.1238207 -56.6393262
C 17.2457079 -7.3784555 -56.1790683
H 17.6104389 -6.833258 -55.3064419
H 16.7096291 -6.695959 -56.8363059

H 18.1109972 -7.7622999 -56.7213065
C 17.1758962 -9.4774166 -54.8192359
H 18.0864798 -9.7670527 -55.3455807
H 17.4716894 -8.935527 -53.9193684
C 16.4479887 -10.7630839 -54.4053729
H 16.0929498 -11.2919093 -55.2902479
H 15.605773 -10.5327737 -53.7532482
H 17.1389073 -11.4070984 -53.8607548
N 15.3935547 -6.0141343 -53.87863
H 15.4044782 -5.57695 -54.785056
C 15.4989416 -5.1573755 -52.7005961
H 16.1260958 -5.6549509 -51.9609007
C 14.1275042 -4.979588 -52.0493859
O 14.0041547 -5.2410058 -50.8625342
C 16.1529791 -3.8128398 -53.0612849
H 15.5453294 -3.3096024 -53.8136461
H 16.1749842 -3.1849001 -52.1697456
C 17.5888162 -3.9565456 -53.5969701
H 17.9604463 -2.9636573 -53.8583359
H 17.5825816 -4.5500643 -54.513358
C 18.5239075 -4.5941443 -52.560969
O 18.5957494 -5.8428049 -52.4662396
O 19.14147 -3.8633975 -51.7551244
N 13.0718701 -4.7526557 -52.834066
H 13.246795 -4.5814834 -53.8192455
C 11.6674641 -4.7573474 -52.3915481
H 11.5110988 -3.9424476 -51.6838844
C 11.2638044 -6.055375 -51.6705936
O 10.7000723 -5.9916155 -50.5815018
C 10.8225721 -4.4658085 -53.6373247
H 11.2703018 -4.9725609 -54.4847155
H 10.8980451 -3.3996945 -53.8162933
C 9.3400869 -4.8422281 -53.6032018
O 8.5960027 -4.4336681 -52.6834481
O 8.8625046 -5.4577929 -54.5871172
N 11.6917214 -7.2238873 -52.1577427
H 12.1378338 -7.2256039 -53.0701622
C 11.4803921 -8.5084706 -51.4804795
H 10.4192842 -8.5902366 -51.2439924
C 12.2581264 -8.616735 -50.1576347
O 11.7173718 -9.1125472 -49.1729519
C 11.8590517 -9.6523727 -52.4351888
H 12.9441046 -9.6885066 -52.5437256
H 11.4310558 -9.4628766 -53.4176041
C 11.3583046 -11.0199242 -51.9562242
H 11.7482812 -11.2164564 -50.9570363

H 11.7634607 -11.7769659 -52.6281464
S 9.5513028 -11.2041661 -51.9296248
C 9.4280423 -12.9497713 -51.463499
H 8.3802362 -13.2482151 -51.4521366
H 9.8585708 -13.0948903 -50.4720088
H 9.9653893 -13.5614913 -52.1881289
N 13.4992124 -8.1155487 -50.1013757
H 13.8751445 -7.7085546 -50.9531837
C 14.3462607 -8.0992723 -48.8917458
H 14.3261654 -9.0880359 -48.4302966
C 13.8414349 -7.109273 -47.8321094
O 13.8696885 -7.4088854 -46.6438106
C 15.7803139 -7.7771225 -49.3386629
H 16.0728206 -8.4953921 -50.1072518
H 15.78842 -6.7853287 -49.7877521
C 16.8146607 -7.8333564 -48.2019743
H 16.5459291 -7.1366515 -47.4067893
H 16.8280532 -8.8437172 -47.7908348
C 18.2187971 -7.4873246 -48.7193819
H 18.4184365 -8.0829343 -49.6128579
H 18.9536731 -7.7499724 -47.9570584
C 18.3487833 -5.992413 -49.0458139
H 18.4319706 -5.4217815 -48.1165061
H 17.4451803 -5.6561907 -49.5619324
N 19.5031294 -5.7293883 -49.9309239
H 20.3954323 -5.9966135 -49.5549232
H 19.5174901 -4.749167 -50.2346865
H 19.3464276 -6.1609146 -50.848426
N 13.3322727 -5.9630881 -48.2710839
H 13.3916351 -5.7966786 -49.2700395
C 12.6752334 -4.9259791 -47.4652083
H 13.3066394 -4.639705 -46.6204001
C 11.3514425 -5.4659331 -46.907204
O 11.0801258 -5.3692947 -45.708615
C 12.463281 -3.6936637 -48.3766892
H 12.0638674 -4.0566426 -49.3241162
C 11.3939495 -2.735481 -47.8251092
H 11.5699158 -2.5482408 -46.7698325
H 10.4027027 -3.1721763 -47.9439616
H 11.4167651 -1.795219 -48.3641339
C 13.8090055 -3.0011842 -48.7056933
H 14.1943818 -2.5034339 -47.8276267
H 14.5553025 -3.7476582 -48.9678682
C 13.7415405 -1.9940272 -49.8643963
H 13.1388901 -1.128049 -49.5957665
H 13.3172396 -2.4665957 -50.7493734

H 14.7489847 -1.6485579 -50.0979748
N 10.5569209 -6.1251239 -47.7575478
H 10.8058184 -6.1502801 -48.7417588
C 9.3406062 -6.8155937 -47.3499945
H 8.687436 -6.0980664 -46.8515223
C 9.6451199 -7.9320667 -46.3426887
O 8.9789309 -7.9857565 -45.3181334
C 8.6090851 -7.3439192 -48.5900398
H 9.2347282 -8.0646011 -49.1166765
H 8.3743305 -6.5167723 -49.2610325
H 7.6825766 -7.8315087 -48.286617
N 10.6967849 -8.7327052 -46.5459848
H 11.1877227 -8.6812436 -47.4322827
C 11.1410652 -9.7413254 -45.5766499
H 10.3095113 -10.4188421 -45.3765601
C 11.5417736 -9.1181235 -44.2316211
O 11.0027769 -9.5243077 -43.2065835
C 12.2879134 -10.5821685 -46.1574739
H 13.1383181 -9.9410307 -46.3900585
H 11.9539012 -11.054893 -47.0827182
C 12.7241101 -11.6616207 -45.1622198
O 13.5224159 -11.3337599 -44.252894
O 12.2018212 -12.793503 -45.265959
N 12.3829782 -8.0769071 -44.2118859
H 12.8033958 -7.7736289 -45.0842588
C 12.8418208 -7.4756884 -42.9545116
H 13.20788 -8.2795145 -42.315361
C 11.7105569 -6.7843906 -42.179033
O 11.5105007 -7.0762136 -40.995856
C 14.0107534 -6.5158331 -43.2086705
H 14.8199205 -7.0620265 -43.6953675
H 13.6867322 -5.7238326 -43.8863098
C 14.5352889 -5.889822 -41.9505213
C 15.3513898 -6.4920806 -41.0560262
H 15.7532765 -7.4912347 -41.1718495
N 15.5335855 -5.6654725 -39.9629168
H 16.0897329 -5.9158626 -39.160673
C 14.8148192 -4.49376 -40.0872147
C 14.6321954 -3.3866221 -39.242576
H 15.1032791 -3.3500841 -38.2735638
C 13.819259 -2.3236695 -39.676404
H 13.6551516 -1.4597462 -39.0434416
C 13.2231146 -2.3758491 -40.9490175
H 12.61592 -1.5480294 -41.2963996
C 13.413713 -3.4925454 -41.785488
H 12.9690569 -3.4997165 -42.7718436

C 14.1985453 -4.5894945 -41.3712467
N 10.9043925 -5.9527161 -42.8492866
H 11.1134384 -5.7630804 -43.8240366
C 9.7438538 -5.2921988 -42.2212211
H 10.0844229 -4.773692 -41.3238945
C 8.6828352 -6.2978319 -41.7797415
O 8.1475363 -6.1684389 -40.6765001
C 9.0796136 -4.2487583 -43.1304959
H 8.177974 -3.8895772 -42.6371761
C 9.9720763 -3.0467225 -43.4104666
H 10.8669468 -3.3593763 -43.9445606
H 10.2661708 -2.5763767 -42.4714854
H 9.4314004 -2.3171273 -44.0117579
O 8.7181591 -4.8066588 -44.3726664
H 9.5496823 -4.9479732 -44.8577988
N 8.4358032 -7.3581704 -42.5633335
H 8.8963046 -7.4314515 -43.466883
C 7.5723311 -8.4495715 -42.1268535
H 6.6350766 -8.0155664 -41.7795424
C 8.1700975 -9.1517994 -40.9247922
O 7.4758181 -9.174459 -39.9330543
C 7.2448461 -9.4620492 -43.2330206
H 8.1639771 -9.7685181 -43.7320877
H 6.8107539 -10.3548548 -42.779457
C 6.239774 -8.9211213 -44.2597927
H 6.2060887 -9.6067299 -45.1067549
H 6.5649357 -7.9487869 -44.6272582
C 4.8301142 -8.7907822 -43.6962677
O 4.0185217 -9.6936336 -43.7800733
N 4.4373901 -7.6329512 -43.2092787
H 3.4549186 -7.5555601 -42.9669857
H 5.0762198 -6.8618807 -43.140931
N 9.4273277 -9.6006942 -40.9284614
H 9.9686587 -9.4839231 -41.780828
C 10.0214635 -10.412134 -39.8567427
H 9.3084547 -11.1990181 -39.609992
C 10.253828 -9.6601678 -38.5291229
O 10.2250454 -10.2893742 -37.4666527
C 11.2944858 -11.0964365 -40.3945858
H 11.8618845 -11.5134649 -39.5643313
H 11.9244235 -10.3674132 -40.904203
C 10.9694015 -12.2521054 -41.3360884
O 10.0165345 -12.9890257 -41.1245311
N 11.7728694 -12.4937022 -42.343769
H 11.4995019 -13.1733068 -43.0362986
H 12.499172 -11.8467642 -42.6449554

N 10.2929531 -8.3240194 -38.537167
H 10.3624872 -7.8437779 -39.4304161
C 10.2075646 -7.5261957 -37.311539
H 10.9370343 -7.9083139 -36.5955343
C 8.8173012 -7.6078404 -36.6366541
O 8.7224682 -7.6976754 -35.4119872
C 10.5911494 -6.0827475 -37.6571958
H 9.8848154 -5.6694188 -38.3793169
H 11.5923603 -6.0574983 -38.0909011
H 10.5793001 -5.471428 -36.7549126
N 7.7281362 -7.648853 -37.4093658
H 7.8464977 -7.6620426 -38.4175533
C 6.3615845 -7.7065981 -36.8837774
H 6.2525433 -6.8732437 -36.1891368
C 5.9991926 -8.9928891 -36.0905116
O 5.3798625 -8.8602907 -35.0312186
C 5.3875801 -7.4605357 -38.0417105
H 5.317507 -8.3466364 -38.6717451
H 5.7360643 -6.6360806 -38.6607618
H 4.3964641 -7.2320804 -37.651936
N 6.3537059 -10.227742 -36.5201682
C 6.0921003 -11.4546801 -35.7992746
H 5.0595046 -11.4728627 -35.4552056
C 7.0141917 -11.5868196 -34.5825291
O 6.5442037 -12.022288 -33.5387212
C 6.2944393 -12.5878507 -36.8162142
H 6.5796654 -13.5239315 -36.3340225
H 5.3790469 -12.7278175 -37.3944718
C 6.9710996 -10.6108314 -37.7595143
H 6.2311127 -10.4906961 -38.55036
H 7.8621115 -10.0271148 -37.9027458
C 7.3852351 -12.0701274 -37.7139111
H 7.3632158 -12.5333561 -38.7012058
H 8.3603036 -12.1870356 -37.2408137
N 8.2600128 -11.0993172 -34.6473682
H 8.5974122 -10.7351963 -35.529587
C 9.1638845 -11.0324667 -33.493428
H 9.2559856 -12.0264496 -33.0531509
C 8.6246533 -10.1030979 -32.3918366
O 8.5898426 -10.4804776 -31.2234502
C 10.5463395 -10.5906214 -33.9929249
H 10.9400176 -11.3588206 -34.6604347
H 10.4349591 -9.6795107 -34.5821898
C 11.5645544 -10.3336599 -32.8982995
C 12.1676299 -11.4139774 -32.226674
H 11.9007571 -12.4282594 -32.4853161

C 13.115578 -11.1764532 -31.2147571
H 13.5750579 -12.007349 -30.6985902
C 13.464218 -9.8578058 -30.873536
H 14.194 -9.6750549 -30.0974929
C 12.8624226 -8.7769348 -31.541472
H 13.1324783 -7.7636193 -31.281157
C 11.9107974 -9.0133361 -32.5496745
H 11.4482941 -8.1777601 -33.0568941
N 8.0603085 -8.9495652 -32.7631566
H 8.1434311 -8.665565 -33.7337643
C 7.4168677 -8.0211198 -31.8271501
H 7.9214617 -8.1082216 -30.8649975
C 5.9315139 -8.326438 -31.5456683
O 5.2535744 -7.4801421 -30.961817
C 7.5941536 -6.574922 -32.324633
H 7.124499 -6.4941971 -33.3064549
H 7.0538484 -5.9068789 -31.6550215
C 9.0136829 -6.0386909 -32.3987931
C 9.4463363 -5.359517 -33.5561945
H 8.7912065 -5.2787379 -34.4129759
C 10.7433545 -4.8150995 -33.6203321
H 11.075635 -4.3075551 -34.5142454
C 11.6148391 -4.9453172 -32.5180674
O 12.8717441 -4.435554 -32.5709646
H 13.0345853 -3.9454995 -33.3800472
C 11.174962 -5.6029107 -31.3511048
H 11.8453005 -5.6829437 -30.507972
C 9.8769556 -6.144496 -31.2893049
H 9.5563376 -6.6474406 -30.3872537
N 5.3871766 -9.481238 -31.956426
H 6.0033601 -10.1538206 -32.4006634
C 3.9274016 -9.6951489 -32.0875758
H 3.5788743 -9.0167038 -32.8656313
C 3.101727 -9.3795924 -30.8315989
O 1.9946272 -8.8568 -30.9597617
C 3.6184426 -11.1199467 -32.5819641
H 4.1373467 -11.2775613 -33.5237867
C 4.0262775 -12.229422 -31.6076246
H 3.4924745 -12.148523 -30.6645446
H 5.0959923 -12.1726016 -31.405786
H 3.820187 -13.1996915 -32.0601955
O 2.2341245 -11.2757929 -32.8256162
H 2.1031661 -11.0808149 -33.7675107
N 3.6312656 -9.6287895 -29.6257628
H 4.5649006 -10.0097299 -29.5872965
C 2.9405287 -9.3177676 -28.3687039

H 1.9221669 -9.6969241 -28.445873
C 2.8276563 -7.8017421 -28.1507249
O 1.7167741 -7.2833488 -28.1555535
C 3.6362754 -10.0368499 -27.1973246
H 3.6353806 -11.1098669 -27.3948457
H 4.6745595 -9.7054275 -27.1474315
C 2.978396 -9.7839054 -25.8233671
H 2.9966347 -8.7178673 -25.6005677
C 1.5310448 -10.2812004 -25.7697653
H 1.4817257 -11.3377576 -26.0293787
H 0.9096754 -9.6938931 -26.4446131
H 1.1425322 -10.1385847 -24.7604909
C 3.7720642 -10.514862 -24.7416685
H 3.7563083 -11.5903824 -24.9153329
H 3.3368301 -10.2987848 -23.7649057
H 4.8026104 -10.1599394 -24.7388168
N 3.9616382 -7.0915666 -28.1298216
H 4.8305866 -7.5964343 -28.1841824
C 4.0327294 -5.6286734 -27.9990707
H 3.5475118 -5.3373421 -27.066531
C 3.3223149 -4.9052738 -29.1588863
O 2.7887892 -3.8112358 -29.0000078
C 5.522688 -5.2420034 -27.9073454
H 5.9417744 -5.7125461 -27.0159418
H 6.0387796 -5.6508386 -28.77724
C 5.8148372 -3.7290084 -27.839953
H 5.4302001 -3.2507583 -28.740477
C 5.2052008 -3.0369301 -26.6265279
H 5.5270028 -3.5308572 -25.7079944
H 4.1165442 -3.0902357 -26.6670599
H 5.5002705 -1.9890255 -26.5938106
C 7.3263404 -3.5054407 -27.8182893
H 7.7487717 -3.9367071 -26.9075049
H 7.5460048 -2.4366408 -27.8259105
H 7.7977514 -3.9704279 -28.681937
N 3.3240981 -5.4922525 -30.3565642
H 3.8200164 -6.3736162 -30.4481514
C 2.6217824 -4.9657189 -31.5266407
H 2.8835245 -3.9148142 -31.6417372
C 1.0943839 -5.029999 -31.3514762
O 0.4108227 -4.0473604 -31.6319395
C 3.127867 -5.7370606 -32.7582961
H 4.209855 -5.607543 -32.8206349
H 2.9292394 -6.7975108 -32.6056175
C 2.5111026 -5.3318787 -34.1077532
H 1.4532277 -5.5947615 -34.1197899

C 2.6686143 -3.8435188 -34.4097948
H 3.7203921 -3.5715168 -34.3494023
H 2.0853599 -3.2554452 -33.704076
H 2.2981698 -3.6320755 -35.4139741
C 3.226156 -6.095398 -35.2194507
H 4.2560045 -5.7514367 -35.3113525
H 2.7083162 -5.937219 -36.1650524
H 3.2404669 -7.1588567 -34.9842874
N 0.5617721 -6.1321914 -30.8100106
H 1.1854655 -6.8955181 -30.565335
C -0.8774119 -6.2932057 -30.5294087
H -1.4450707 -5.8133694 -31.3273322
C -1.3387857 -5.5989339 -29.2472027
O -2.4972833 -5.2107496 -29.1799714
C -1.2259879 -7.7857952 -30.5059816
H -0.5395652 -8.3161481 -29.8425807
H -2.2423577 -7.9080194 -30.1304602
C -1.1550235 -8.3653732 -31.9235204
H -0.1595575 -8.2283015 -32.3457716
H -1.8592111 -7.825137 -32.5503861
C -1.517416 -9.8499764 -31.9535904
H -1.7789138 -10.1101266 -32.9786297
H -2.3926915 -10.0247935 -31.3275004
N -0.3815104 -10.6888054 -31.5472774
H 0.4686067 -10.5891001 -32.0922565
C -0.2819976 -11.5284697 -30.5397689
N 0.7987728 -12.2399241 -30.4453776
H 0.9213052 -12.9191248 -29.7195572
H 1.4926744 -12.1243647 -31.1788985
N -1.2140364 -11.691179 -29.6413828
H -2.0524106 -11.1461476 -29.7056909
H -1.0837421 -12.3425586 -28.8909044
N -0.4359914 -5.4137744 -28.291648
H 0.4565919 -5.878876 -28.4106519
C -0.6121594 -4.6637386 -27.0405899
H -1.5484464 -4.9448234 -26.5574101
C -0.6419177 -3.1442925 -27.2699944
O -1.4719593 -2.454789 -26.6849815
C 0.5604857 -5.0831334 -26.1452238
H 0.4619065 -6.1502756 -25.9371324
H 1.4675594 -4.941078 -26.7285774
C 0.7664583 -4.3591921 -24.8161269
H -0.1072872 -4.5150072 -24.1788573
H 0.8875321 -3.2879945 -24.9917974
C 2.035639 -4.9226683 -24.1581527
O 1.8929473 -5.6917077 -23.1831504

O 3.1384206 -4.6239782 -24.6732221
N 0.1917402 -2.6286023 -28.1822229
H 0.9142711 -3.2259607 -28.5641261
C 0.1832659 -1.2150978 -28.5658876
H 0.0037262 -0.6137299 -27.6722889
C -0.9246664 -0.8558517 -29.5768898
O -1.3427934 0.2986789 -29.6289793
C 1.5712314 -0.8568396 -29.111109
H 1.7755088 -1.4407739 -30.0082925
H 2.3275326 -1.0683264 -28.3535512
H 1.5991124 0.2052893 -29.3567835
N -1.3690744 -1.8151607 -30.399589
H -0.9570034 -2.7323259 -30.3107115
C -2.4122034 -1.6197109 -31.4130676
H -2.8401389 -0.6237023 -31.2850044
C -3.5838278 -2.5891446 -31.1989809
O -4.4984085 -2.2893246 -30.4387788
C -1.8158924 -1.6685056 -32.833723
H -1.3145361 -2.624073 -32.987596
H -2.63415 -1.6141187 -33.553035
C -0.8409829 -0.5505884 -33.1512774
C -1.3096435 0.649862 -33.7150379
H -2.3647469 0.7833783 -33.9051926
C 0.531843 -0.7025696 -32.8802269
H 0.8864084 -1.6149005 -32.4260483
C -0.4110316 1.6879453 -34.01348
H -0.7806818 2.6123909 -34.4308109
C 1.4300197 0.3388147 -33.1757978
H 2.4760809 0.2347951 -32.9481857
C 0.9602995 1.5313944 -33.7512922
H 1.6466328 2.3356076 -33.9776088
N -3.6205628 -3.7132496 -31.9236746
H -2.8479066 -3.9034345 -32.5436994
C -4.6046085 -4.7861899 -31.7255437
H -4.6477881 -5.0219769 -30.6606922
C -4.2176418 -6.0541322 -32.4919207
O -3.4466607 -6.006089 -33.4557384
C -6.0082382 -4.3507814 -32.1811528
H -6.3304691 -3.4699273 -31.624698
H -6.7175425 -5.152142 -31.9705056
O -6.0325689 -4.0692292 -33.5679786
H -5.899994 -3.1163384 -33.6518697
N -4.8741413 -7.1737827 -32.163381
H -5.4716506 -7.1487112 -31.3494907
C -4.8665038 -8.4037354 -32.9751168
H -3.8421984 -8.7622554 -33.024553

C -5.296619 -8.1459118 -34.4291164
O -4.6251069 -8.5946963 -35.3565651
C -5.7191938 -9.4992887 -32.2851475
H -5.2617698 -9.6962688 -31.3135578
C -7.1749464 -9.0643969 -32.0189038
H -7.7180113 -8.9252308 -32.9539572
H -7.2157387 -8.1483551 -31.4309694
H -7.6857446 -9.8419113 -31.4478146
C -5.7591062 -10.8389807 -33.0521625
H -6.3486961 -11.5529072 -32.4750014
H -6.2623358 -10.6958331 -34.0075932
C -4.3868889 -11.4732058 -33.3065014
H -3.86465 -11.6184485 -32.3614838
H -3.7929475 -10.8453952 -33.9693974
H -4.5241808 -12.4437534 -33.7840225
N -6.3197801 -7.3081212 -34.6459337
H -6.7867577 -6.9174776 -33.8407924
C -6.8108968 -6.9429317 -35.9822531
H -6.9295796 -7.8697976 -36.5438468
C -5.8010042 -6.0946841 -36.7703672
O -5.7534856 -6.2049544 -37.9993011
C -8.1929232 -6.2678168 -35.828368
H -8.8936588 -6.9935093 -35.4113768
H -8.1035006 -5.4462128 -35.1147961
C -8.795135 -5.6932521 -37.1238768
H -9.7559562 -5.2332397 -36.8894435
H -8.1411857 -4.8942535 -37.4703831
C -9.0162358 -6.7421972 -38.2346595
H -9.9843958 -7.2211919 -38.076221
H -8.2596339 -7.5233973 -38.1830747
C -8.9707821 -6.1540308 -39.6519625
H -9.7832162 -5.4324325 -39.7803459
H -9.1312743 -6.9749907 -40.3575952
N -7.6582374 -5.5172426 -39.9201159
H -7.5238449 -4.6979612 -39.3466649
H -7.5232324 -5.260878 -40.8933727
H -6.8930147 -6.1433565 -39.6825251
N -4.9831557 -5.2914209 -36.0868715
H -5.1114733 -5.2316311 -35.0833861
C -3.8621657 -4.5557308 -36.6755995
H -4.2125805 -3.9476157 -37.5101316
H -3.4303927 -3.8987074 -35.9202956
C -2.7652385 -5.4947951 -37.177779
O -2.4814048 -5.5085537 -38.373279
N -2.2677174 -6.3806718 -36.3070245
H -2.5945535 -6.342489 -35.3479413

C -1.2607822 -7.3958332 -36.6512525
H -0.3445124 -6.8925585 -36.961776
C -1.6939054 -8.2984128 -37.8176158
O -0.9311845 -8.4717807 -38.7673839
C -0.9587692 -8.2239316 -35.3945171
H -0.5126998 -7.5591821 -34.6545479
H -1.9003645 -8.5918536 -34.9852958
C -0.0205586 -9.403772 -35.5957312
C -0.476939 -10.7237244 -35.4080804
H -1.509788 -10.9114615 -35.1526382
C 0.4018223 -11.8087914 -35.5828742
H 0.0411055 -12.8215519 -35.4605813
C 1.7396798 -11.5814567 -35.9510346
H 2.4073452 -12.4177367 -36.1126058
C 2.197147 -10.2669613 -36.1448257
H 3.2167878 -10.0866761 -36.4561706
C 1.3158525 -9.1847377 -35.975059
H 1.6594669 -8.1812815 -36.1674815
N -2.9508894 -8.7602365 -37.8162996
H -3.5180262 -8.6013591 -36.9897401
C -3.5164419 -9.5705426 -38.9055008
H -2.8830213 -10.4489155 -39.0412821
C -3.4884375 -8.8191223 -40.2441416
O -3.0854456 -9.4032043 -41.2444796
C -4.9353343 -10.0606912 -38.5229828
H -5.4930771 -9.2156787 -38.1147458
C -5.7199256 -10.6008875 -39.7362459
H -5.1748012 -11.428586 -40.1934969
H -5.8704642 -9.8203191 -40.4808494
H -6.7059161 -10.950001 -39.4339102
C -4.8187313 -11.1576382 -37.4374046
H -4.3660146 -12.0503218 -37.8713733
H -4.1598068 -10.814504 -36.6407699
C -6.1553173 -11.5460725 -36.7923586
H -6.7955629 -12.0580817 -37.5094256
H -6.6616767 -10.6550823 -36.4198857
H -5.9674939 -12.2254155 -35.9610021
N -3.8323925 -7.5248185 -40.2879394
H -4.0656676 -7.0556824 -39.4218744
C -3.7691065 -6.745843 -41.5391746
H -4.3005425 -7.2994218 -42.3139513
C -2.3393446 -6.5519942 -42.0526116
O -2.1145754 -6.6247537 -43.2621331
C -4.4332741 -5.3718712 -41.3703427
H -3.8537605 -4.6109815 -41.8970195
H -4.4733346 -5.1006908 -40.3138927

O -5.7412933 -5.3987764 -41.9212897
H -5.6390313 -5.3843889 -42.8827614
N -1.3861787 -6.3126124 -41.1495095
H -1.6547347 -6.2826085 -40.1714205
C 0.0158965 -6.0425797 -41.481886
H 0.0577829 -5.3112942 -42.2894989
C 0.740751 -7.3012983 -41.9896076
O 1.4825599 -7.2298511 -42.9719905
C 0.6993255 -5.4537121 -40.2332656
H 0.5759614 -6.161702 -39.4119538
H 1.768021 -5.3583632 -40.4270006
C 0.1460188 -4.0790425 -39.7977291
H -0.9410754 -4.0968931 -39.8068985
C 0.5881962 -3.7787531 -38.3651539
H 1.6688825 -3.8436922 -38.2817429
H 0.1358835 -4.4987693 -37.6855946
H 0.2728934 -2.7773283 -38.0815491
C 0.5838749 -2.9467471 -40.7300837
H 1.6679734 -2.8669288 -40.751094
H 0.1714852 -2.0035095 -40.3724266
H 0.2150466 -3.1247907 -41.7383577
N 0.4550266 -8.4611916 -41.3890336
H -0.1379686 -8.4416052 -40.564342
C 0.9646168 -9.7595951 -41.8364496
H 2.0196674 -9.6433663 -42.0892642
C 0.2485841 -10.2596498 -43.1035624
O 0.8987943 -10.7616169 -44.0158858
C 0.8455888 -10.7511466 -40.6638834
H 1.3523783 -10.326376 -39.7955445
H -0.210257 -10.8640917 -40.4110157
C 1.4357562 -12.1477194 -40.9424942
H 0.9147976 -12.6038002 -41.7839374
C 2.9345315 -12.1074372 -41.2461209
H 3.4682867 -11.5756565 -40.4587843
H 3.1048157 -11.6059846 -42.1990644
H 3.3258448 -13.1209115 -41.3293987
C 1.228386 -13.036948 -39.7157139
H 1.7534838 -12.6171902 -38.8578697
H 1.6078131 -14.0384338 -39.9180822
H 0.1647686 -13.1057515 -39.4881301
N -1.0698244 -10.0583188 -43.2209953
H -1.5784126 -9.6705319 -42.4337335
C -1.8288138 -10.4543792 -44.4133578
H -1.6406428 -11.512455 -44.6026739
C -1.3632127 -9.6903915 -45.6534104
O -1.0336879 -10.3147171 -46.6612399

C -3.3368109 -10.2801862 -44.1613091
H -3.5384081 -9.2595103 -43.8397539
H -3.6148275 -10.9493552 -43.3461491
C -4.2354917 -10.6192243 -45.3629775
H -5.2387681 -10.8120393 -44.981554
H -3.8764807 -11.5469091 -45.8110439
S -4.390968 -9.3657168 -46.6781462
C -5.1799099 -8.0079527 -45.7729399
H -5.4383667 -7.2116522 -46.4701612
H -4.4931662 -7.6138861 -45.0242914
H -6.0870682 -8.3705426 -45.2881865
N -1.2836702 -8.3547994 -45.597801
H -1.5351508 -7.8696527 -44.7427603
C -0.9788531 -7.5879528 -46.8043397
H -1.457325 -8.1034868 -47.6360249
C 0.521404 -7.5756896 -47.1451478
O 0.8637811 -7.5102657 -48.3264741
C -1.6430715 -6.2032923 -46.748782
H -1.1355764 -5.5871202 -46.0089988
H -2.6802938 -6.3144597 -46.4385527
C -1.6394516 -5.5151375 -48.1094585
N -0.9709235 -4.389742 -48.2157069
H -0.4685428 -4.0199304 -47.4270163
H -0.9501478 -3.9346266 -49.1103779
O -2.2486604 -5.9590541 -49.0785727
N 1.416904 -7.7516498 -46.1678263
H 1.0980991 -7.8154289 -45.2078644
C 2.8375119 -7.9866019 -46.4363406
H 3.1969886 -7.2710589 -47.1758242
H 3.3911814 -7.8211393 -45.5225839
C 3.1279278 -9.4043741 -46.945777
O 3.8298141 -9.5493483 -47.9453507
N 2.447086 -10.4225332 -46.4101552
H 1.9315207 -10.2624063 -45.5490469
C 2.4854058 -11.7979805 -46.9139969
H 3.5152832 -12.1562071 -46.9087821
H 1.8931677 -12.4278665 -46.2504679
C 1.9239234 -11.9295401 -48.3328251
O 2.5932635 -12.4826701 -49.2022689
N 0.7929469 -11.2777152 -48.628854
H 0.267872 -10.8632915 -47.8641561
C 0.2683398 -11.1228987 -49.9938683
H 0.0573556 -12.1067264 -50.4128921
C 1.2826057 -10.4351154 -50.919821
O 1.4660857 -10.8427349 -52.0678355
C -1.03995 -10.3126457 -49.9511539

H -1.8060414 -10.9034882 -49.4476874
H -0.8743164 -9.4136616 -49.3588149
C -1.5543657 -9.8836418 -51.2950987
C -1.1187229 -8.7991881 -51.9758444
H -0.3563124 -8.1147227 -51.6133052
N -1.6791194 -8.7882528 -53.2363242
H -1.3736528 -8.1625098 -53.9676252
C -2.5438482 -9.8457334 -53.4186151
C -3.3411695 -10.2526051 -54.4992947
H -3.3476281 -9.6931163 -55.422582
C -4.1539985 -11.3894695 -54.358604
H -4.801853 -11.6994674 -55.1695271
C -4.1314936 -12.1172815 -53.1550939
H -4.7589197 -12.9925633 -53.044334
C -3.3101688 -11.7105559 -52.0842815
H -3.3046579 -12.2804389 -51.1652787
C -2.499787 -10.5592805 -52.1831049
N 1.96428 -9.3993704 -50.4253499
H 1.7809878 -9.1156849 -49.4709382
C 2.9636726 -8.654149 -51.2011413
H 2.5137098 -8.3680451 -52.1510126
C 4.1751778 -9.5252683 -51.5380323
O 4.584636 -9.5468988 -52.6991712
C 3.3562456 -7.3613529 -50.4714756
H 3.7076416 -7.5770001 -49.4645344
C 4.4220107 -6.5580398 -51.2039862
H 4.0784879 -6.3762013 -52.2179027
H 5.3645424 -7.1059384 -51.2128172
H 4.574357 -5.6062331 -50.6915057
O 2.2203371 -6.5288334 -50.4111686
H 1.6945377 -6.8265429 -49.6442254
N 4.6517677 -10.3483214 -50.5991615
H 4.2697926 -10.2894075 -49.6593257
C 5.6772822 -11.3580867 -50.8568759
H 6.5451977 -10.8700353 -51.3041686
C 5.1923812 -12.4341222 -51.8490049
O 5.8968642 -12.7354913 -52.8110289
C 6.1067911 -11.9647842 -49.5145566
H 5.2580115 -12.4435631 -49.0255728
H 6.499734 -11.1841373 -48.8617151
H 6.8863492 -12.7084471 -49.6781218
N 3.9534366 -12.9249302 -51.7086818
H 3.4267844 -12.6496469 -50.8836684
C 3.334672 -13.8997941 -52.6291921
H 3.9737587 -14.7791788 -52.6745089
C 3.2311635 -13.3581777 -54.0601941

O 3.501972 -14.0879971 -55.0132972
C 1.9611062 -14.3607064 -52.0920771
H 1.4005589 -13.4924383 -51.7508018
C 1.0987965 -15.094003 -53.1308964
H 1.6582642 -15.9270397 -53.5595551
H 0.8015677 -14.4086898 -53.9226587
H 0.1946828 -15.4764729 -52.6554527
C 2.1541222 -15.3188572 -50.9094302
H 2.6359165 -16.2380737 -51.2437664
H 1.18814 -15.559973 -50.4658729
H 2.7765934 -14.8574841 -50.1444956
N 2.9105554 -12.073782 -54.2549198
H 2.6628376 -11.510579 -53.4444772
C 2.8382215 -11.4848562 -55.603466
H 2.5477263 -12.2731829 -56.294507
C 4.1932573 -11.0523229 -56.1735234
O 4.3050569 -11.0014922 -57.3976465
C 1.7500749 -10.3972881 -55.6684607
H 1.973677 -9.6224873 -54.932472
H 1.7766916 -9.9369756 -56.657886
C 0.3181127 -10.9321047 -55.4242165
H 0.2277581 -11.265099 -54.390261
H -0.3748739 -10.1024204 -55.5677658
C -0.0987512 -12.0894509 -56.3560436
H 0.544746 -12.9544503 -56.17014
H 0.0153415 -11.7777132 -57.39766
C -1.5577073 -12.5187353 -56.1029819
H -2.2269318 -11.6802266 -56.3325773
H -1.6736438 -12.7561101 -55.0366384
N -1.920309 -13.7026939 -56.930816
H -1.8494992 -13.5031153 -57.9228767
H -2.903941 -13.9547134 -56.8042702
H -1.3493329 -14.5134468 -56.71273
N 5.239108 -10.8811623 -55.358782
H 5.0832237 -10.9063656 -54.3572045
C 6.6252368 -10.7490509 -55.8568326
H 6.6136699 -10.1725473 -56.7835268
C 7.23075 -12.111499 -56.2120809
O 7.8163342 -12.237301 -57.2830009
C 7.5248285 -9.9796415 -54.8686851
H 7.4639628 -10.4501565 -53.8877875
C 8.9905939 -9.944826 -55.3227672
H 9.0568977 -9.5997377 -56.3555774
H 9.4372889 -10.9357012 -55.2428839
H 9.5579733 -9.2635562 -54.6896495
C 7.0793244 -8.5186274 -54.7449023

H 7.2214826 -8.0117064 -55.6995769
H 7.6884329 -8.0243809 -53.9867521
H 6.0354079 -8.4688468 -54.4529373
N 7.0278598 -13.1595392 -55.4024952
H 6.5409429 -13.011564 -54.5230944
C 7.6271667 -14.4858677 -55.6559459
H 8.6634613 -14.3031979 -55.9438593
C 6.9906641 -15.2279986 -56.8419336
O 7.6716931 -15.9879257 -57.5225639
C 7.6575082 -15.3317962 -54.3638388
H 7.9124323 -14.6589608 -53.5431137
C 6.2996944 -15.9707711 -54.0400428
H 6.0816508 -16.7851927 -54.7321286
H 5.5262014 -15.2171777 -54.1221082
H 6.2972443 -16.3543042 -53.0201916
C 8.777694 -16.3911983 -54.4438692
H 8.5331507 -17.1303691 -55.2074872
H 9.7068405 -15.8989223 -54.7355244
C 9.0351373 -17.1189259 -53.1182629
H 8.173149 -17.7267722 -52.8436591
H 9.2387219 -16.3944539 -52.3289923
H 9.8999306 -17.7735446 -53.2299662
N 5.7387061 -14.9039989 -57.1868191
H 5.2127234 -14.3222172 -56.5485866
C 5.1062204 -15.3009733 -58.4534771
H 5.3874356 -16.3318692 -58.6680816
C 5.6030433 -14.4874707 -59.672003
O 5.0849158 -14.6599443 -60.773668
C 3.5735904 -15.2270815 -58.2905951
H 3.3168007 -14.2373792 -57.910768
H 3.1057086 -15.3358337 -59.269651
C 2.9775319 -16.3018904 -57.3585176
H 3.4899355 -16.2918303 -56.3990126
C 1.4957103 -15.9962792 -57.1210999
H 0.9610944 -15.9934025 -58.0710052
H 1.4152946 -15.0272253 -56.6348142
H 1.0717324 -16.755584 -56.4636815
C 3.0815974 -17.7101515 -57.947436
H 2.616133 -17.7444842 -58.9324024
H 2.5847047 -18.4241014 -57.2906702
H 4.1275589 -18.0041337 -58.0324987
N 6.5584693 -13.5695965 -59.4840437
H 6.9635998 -13.4805897 -58.559394
C 7.1419561 -12.7294521 -60.533019
H 8.0300305 -12.2428227 -60.1296168
H 7.4428545 -13.3604889 -61.3694312

C 6.2113295 -11.6372743 -61.0664694
O 6.4668133 -11.1007314 -62.1414297
N 5.0989407 -11.3521821 -60.3807085
H 4.9813281 -11.7997437 -59.4822739
C 4.011173 -10.5016823 -60.8819061
H 4.0372055 -10.488733 -61.9692609
C 4.1458654 -9.0360507 -60.4550618
O 4.0311999 -8.1487806 -61.2999311
C 2.6640571 -11.1114873 -60.4638078
H 1.8926477 -10.351034 -60.4842377
H 2.730934 -11.4831747 -59.4414438
C 2.2535011 -12.2657448 -61.3868724
H 1.4591474 -12.8339579 -60.902344
H 3.1069454 -12.932034 -61.5231857
S 1.6498686 -11.7327541 -63.0139356
C 1.6214996 -13.3280329 -63.8756168
H 1.2174771 -13.1904539 -64.8799789
H 0.9970689 -14.0358844 -63.3303468
H 2.6341481 -13.7267153 -63.9450518
N 4.4403578 -8.7747006 -59.1755103
H 4.5741277 -9.5745046 -58.565276
C 4.7721047 -7.4432695 -58.6461105
H 4.2329189 -6.6929209 -59.2287529
C 6.2628089 -7.1513591 -58.8255713
O 7.1132365 -8.0286716 -58.7190631
C 4.3124856 -7.3225627 -57.1776623
H 3.2218766 -7.3201566 -57.1538076
H 4.664482 -8.1940433 -56.6248092
C 4.8413101 -6.0586761 -56.4669405
H 5.9269408 -6.1201353 -56.3940316
H 4.5835999 -5.1738691 -57.0510368
C 4.2767258 -5.8952971 -55.0518431
H 4.2724669 -6.8599056 -54.5417714
H 3.2469937 -5.5465683 -55.1283597
C 5.0870934 -4.8828455 -54.2236432
H 4.4787623 -4.580427 -53.3658758
H 5.2958632 -3.991815 -54.8235343
N 6.3573003 -5.466491 -53.7188249
H 6.2395348 -6.3113772 -53.1868018
H 6.9070721 -4.8226841 -53.1439003
H 7.0461657 -5.6510471 -54.4521823
N 6.5831059 -5.8796803 -59.0166479
H 5.8235267 -5.2105063 -59.113986
C 7.9375657 -5.3227778 -59.0310328
H 8.6143732 -5.9856498 -58.4939189
C 7.9299697 -3.9753104 -58.3016906

O 6.8849011 -3.3299543 -58.2056804
C 8.3940536 -5.2114097 -60.498702
H 7.5989185 -4.7461053 -61.083704
C 9.6867695 -4.4393521 -60.7534294
H 10.461601 -4.7634189 -60.0624864
H 9.5092652 -3.3712309 -60.637248
H 10.0261984 -4.6212524 -61.7728531
O 8.6164189 -6.5095718 -60.9963761
H 8.1255677 -7.1366062 -60.4489059
N 9.0868513 -3.5097747 -57.8288001
H 9.9133854 -4.0836863 -57.9019445
C 9.2842826 -2.0970461 -57.484942
H 8.3344924 -1.5820776 -57.6283965
C 10.2397767 -1.4588863 -58.488381
O 11.1822076 -2.0878065 -58.9748673
C 9.6723067 -1.8988096 -55.9998239
H 9.4745394 -2.8358148 -55.4851521
C 11.1589555 -1.5434698 -55.8111287
H 11.3819425 -0.5661663 -56.2441685
H 11.7740098 -2.3086833 -56.2769605
H 11.4226248 -1.4985324 -54.7557414
C 8.7636256 -0.8305709 -55.3496209
H 8.9602225 0.1445593 -55.7991688
H 7.7226656 -1.0931424 -55.5430241
C 8.9349449 -0.7232145 -53.8274753
H 9.9006709 -0.2819557 -53.5826193
H 8.8562241 -1.7099889 -53.3708842
H 8.1532925 -0.0869728 -53.4164581
N 9.9517901 -0.2103244 -58.8094482
H 9.1682783 0.231535 -58.3373574
C 10.654951 0.6360141 -59.7608788
H 11.4393476 0.0802286 -60.2761536
C 11.3137825 1.7707113 -58.9834636
O 10.6744127 2.3280577 -58.0948429
C 9.5994166 1.0937026 -60.7929687
H 8.6089785 1.028997 -60.3467226
H 9.6158493 0.3827572 -61.6188321
C 9.6756245 2.4856417 -61.3678588
N 9.6560584 2.7683857 -62.7304314
C 9.6304442 4.1041051 -62.8320208
H 9.6677495 4.647484 -63.7669766
N 9.599696 4.6686446 -61.6145544
H 9.6754936 5.6576488 -61.4159251
C 9.6037052 3.6646771 -60.6808175
H 9.5692624 3.7963986 -59.6106405
N 12.5267095 2.1509732 -59.3842939

H 13.0058659 1.5797279 -60.0714761
C 13.1036285 3.4786336 -59.1419166
H 12.3791542 4.0801703 -58.6065819
C 13.4003518 4.137936 -60.4980748
O 13.9804095 3.4895145 -61.3751488
C 14.3486196 3.3947989 -58.2404563
H 14.0355429 3.0345998 -57.2602812
H 15.0443904 2.6671256 -58.661884
C 15.0998792 4.7290328 -58.0561606
H 15.5972644 4.9762276 -58.9978091
H 15.8861092 4.5829474 -57.3125827
C 14.2028056 5.9034645 -57.6227446
O 14.1451209 6.8963968 -58.3833069
O 13.5576779 5.8054743 -56.5593021
N 13.0453446 5.4126846 -60.6712948
H 12.6425479 5.9066621 -59.8759317
C 13.3632583 6.2062371 -61.8533931
H 14.1139036 5.6662568 -62.4167197
C 13.9496037 7.5757594 -61.5001384
O 13.3110616 8.3851175 -60.8299668
C 12.1357501 6.3519771 -62.752082
H 11.3121412 6.7960327 -62.1946084
H 11.8710934 5.3727228 -63.1411174
H 12.3807769 6.9994993 -63.5956822
N 15.109189 7.8745898 -62.0873356
H 15.4773862 7.2053572 -62.7563538
C 15.8432781 9.1316037 -61.9126432
H 15.2448618 9.8123746 -61.30726
C 16.0561624 9.7692783 -63.2824116
O 16.4188304 9.0839867 -64.2349318
C 17.1881392 8.8969502 -61.188147
H 17.8076121 8.2561194 -61.8169794
C 16.9764192 8.1765845 -59.8366834
H 16.3622953 8.7964619 -59.1838709
H 16.4432474 7.2414061 -60.0042166
C 17.9261826 10.2389898 -60.9959842
H 17.3004281 10.929227 -60.4298202
H 18.1645092 10.6855659 -61.9607414
H 18.8686102 10.0894265 -60.4738383
C 18.2749524 7.8099776 -59.110778
H 18.7708445 8.7035771 -58.7336403
H 18.9412565 7.2714474 -59.7855677
H 18.0370505 7.1684199 -58.2616609
N 15.8801458 11.0823937 -63.3805037
H 15.5331748 11.5872138 -62.5688742
C 15.9623305 11.817571 -64.6442913

H 16.7952455 11.4316431 -65.2351664
C 16.2306405 13.3158319 -64.4223685
O 16.001996 13.8407697 -63.3256233
C 14.6780471 11.5721577 -65.4405948
H 14.5614297 12.3592306 -66.1828294
H 14.8143697 10.6397859 -65.9771394
C 13.3981278 11.4233831 -64.5967226
H 13.2409175 12.3213791 -64.0054631
H 13.4843556 10.5777558 -63.912386
C 12.2010246 11.1445692 -65.486179
O 12.4220538 10.9836834 -66.7062945
O 11.0711677 11.0577901 -64.952999
N 16.7127389 14.0282412 -65.4533237
H 16.8577185 13.566903 -66.349382
C 16.9273486 15.4814323 -65.3898288
H 16.3871383 15.8476574 -64.5193038
C 16.3447654 16.2645858 -66.5817988
O 16.1558116 15.7419953 -67.6835179
C 18.4109936 15.8096423 -65.1287159
H 18.8261429 15.0930979 -64.4212154
H 18.4804463 16.7986531 -64.677849
C 19.2719235 15.817902 -66.3805842
O 19.9812442 14.8726451 -66.670495
N 19.2629312 16.8803734 -67.1552989
H 19.7115882 16.7659892 -68.0471681
H 18.6938739 17.6945154 -66.9492704
N 16.1171737 17.5560555 -66.3461034
H 16.3244087 17.9218972 -65.4216912
C 15.6409335 18.5213459 -67.3311437
H 15.1563779 17.9880668 -68.1515813
C 16.8207639 19.3019001 -67.9050657
O 17.814395 19.5196466 -67.2156087
C 14.6025547 19.470826 -66.7156745
H 15.0457816 19.9951369 -65.8663646
H 13.7532703 18.8943344 -66.3687152
C 14.1049765 20.4776596 -67.7549781
O 14.0497 21.6808959 -67.4357038
O 13.8939207 20.0301459 -68.9036003
N 16.6932574 19.7127438 -69.1587056
H 15.7361606 19.6535177 -69.5221682
C 17.6417525 20.4891035 -69.9664808
H 18.1709276 19.7718774 -70.5775385
C 18.7397635 21.2279062 -69.1911158
O 19.6163392 20.5303848 -68.6231238
O 18.7905354 22.4280729 -69.0989548
C 16.8603575 21.4153103 -70.9236658

H 16.3055296 22.1240006 -70.3041713
H 17.5800612 21.9809911 -71.516076
C 15.8643249 20.7213124 -71.8783228
H 15.1037594 20.1933291 -71.2956912
C 15.1458514 21.7861056 -72.7164382
H 15.8579213 22.3362843 -73.3312233
H 14.6219113 22.4788679 -72.0561909
H 14.4058411 21.3071409 -73.3609419
C 16.5435476 19.7199673 -72.8232848
H 17.329409 20.2094041 -73.398468
H 15.8002973 19.3095617 -73.5106352
H 16.9602655 18.8885803 -72.2557724

Supplementary Data 7. Cartesian coordinates for perivine docked into TePeNMT+SAM.

N -2.1385667 21.7753992 -15.9844882
H -2.8881317 22.3451992 -15.612073
H -2.4187043 21.4412796 -16.898962
H -1.952445 20.9940613 -15.3721405
C -0.8983927 22.6008534 -16.1048954
H -1.0805973 23.3575681 -16.8709468
C 0.33849 21.7897622 -16.5659561
O 1.2553163 22.327476 -17.1874333
C -0.6053818 23.3116885 -14.7771474
H 0.3668547 23.80077 -14.8479421
H -0.5567763 22.5711725 -13.9778747
C -1.639947 24.3835703 -14.4160168
H -1.396285 24.7758699 -13.426708
H -2.6273748 23.9290305 -14.3587376
S -1.7285502 25.7740286 -15.5733039
C -2.9534829 26.7844932 -14.6928753
H -3.1342375 27.7045542 -15.2488035
H -2.5798462 27.0332981 -13.6984196
H -3.8891334 26.2323095 -14.5953778
N 0.3673335 20.4886197 -16.2657559
H -0.4109708 20.1080689 -15.7405706
C 1.3938537 19.5859753 -16.7771625
H 1.3580658 19.6103407 -17.8672226
H 2.3753087 19.9422459 -16.4680576
C 1.2496557 18.1385969 -16.3224611
O 0.7986647 17.8537127 -15.2154758
N 1.7628938 17.2390447 -17.1522057
H 2.1121366 17.5432004 -18.0501647
C 2.0288584 15.8382482 -16.8338954
H 1.9028599 15.7027836 -15.7604402
C 3.4957816 15.5076051 -17.1394012
O 4.119702 16.1812749 -17.9609391
C 0.9990926 14.9223526 -17.5032517
H 1.2572628 13.8928438 -17.2749979
H 0.0423459 15.1358366 -17.026009
C 0.8149397 15.0455348 -19.020348
H 1.6157817 14.5050359 -19.5288645
H 0.8699863 16.0954136 -19.3176114
C -0.5554566 14.4754363 -19.4180847
O -1.297864 15.2053148 -20.1119062
O -0.875195 13.3478418 -18.9747065
N 4.1061184 14.7016468 -16.2632362
H 3.5442127 14.1102083 -15.6715657
C 5.5629684 14.6122266 -16.0983634

H 6.0446756 14.8186955 -17.0600251
C 5.9695982 13.2129132 -15.6736854
O 5.3463653 12.639753 -14.786269
C 6.0594875 15.5900786 -15.0182406
H 5.5966651 15.3285324 -14.0641537
H 7.1389711 15.4636292 -14.915048
C 5.7688522 17.0627619 -15.3210483
H 6.199365 17.3109927 -16.2919241
H 4.6914859 17.2284646 -15.351451
C 6.3694499 17.9615862 -14.2310701
H 7.4516987 17.8220036 -14.192695
H 5.9514537 17.6743696 -13.2638997
C 6.0413128 19.4366002 -14.4861744
H 6.4275873 20.029751 -13.6516003
H 4.9522862 19.5499365 -14.5026758
N 6.6253332 19.9107507 -15.7656037
H 7.6331593 19.818804 -15.7546612
H 6.3843249 20.8792368 -15.933991
H 6.2683797 19.3550584 -16.5356419
N 7.106455 12.7593095 -16.1705568
H 7.4956956 13.2759729 -16.9574346
C 7.5639831 11.383838 -16.027934
H 6.7220861 10.7521143 -15.7410336
C 8.6256294 11.3080201 -14.9245199
O 9.6775993 11.9537076 -14.9983873
C 8.0743326 10.8706348 -17.3888252
H 8.9570019 11.4416081 -17.6791626
H 8.3787829 9.8302572 -17.2664366
C 7.0169986 10.9370419 -18.5172246
H 6.1405201 10.371064 -18.1914962
H 7.409044 10.4393792 -19.4038394
C 6.5974706 12.3719046 -18.896483
O 7.4785217 13.2681533 -18.9084406
O 5.3745041 12.6072441 -18.9936412
N 8.3366279 10.5700175 -13.8495991
H 7.428984 10.1185224 -13.7997984
C 9.2537472 10.4628666 -12.7165948
H 9.411715 11.4704627 -12.3299929
C 10.6243327 9.9036971 -13.153139
O 10.6986872 8.9385328 -13.9069108
C 8.6037136 9.6350831 -11.6036772
H 8.4204313 8.619799 -11.9600852
H 7.655759 10.0896643 -11.3114457
H 9.2647124 9.595986 -10.737355
N 11.703524 10.5367378 -12.6721267
H 11.5296608 11.3030954 -12.0418231

C 13.1142625 10.3142897 -13.0702562
H 13.6516325 11.146085 -12.6173586
C 13.4243279 10.5119185 -14.5675031
O 14.4681525 10.0599575 -15.0339048
C 13.7765064 9.0619071 -12.4395625
H 14.845682 9.122573 -12.6445486
C 13.6265139 9.0675566 -10.9118445
H 12.5803642 8.9423089 -10.6318339
H 14.0018322 10.0070241 -10.506304
H 14.2061243 8.2486758 -10.485005
C 13.2962742 7.7038875 -12.9652798
H 12.2890587 7.4911381 -12.6080123
H 13.9637118 6.9192992 -12.6112727
H 13.2950871 7.7094786 -14.0550769
N 12.605294 11.3349009 -15.2378421
H 11.7369915 11.5814565 -14.7805248
C 12.7314974 11.833814 -16.6111805
H 11.8724211 12.4921825 -16.7495073
C 12.6154246 10.7757957 -17.7237248
O 13.1625965 9.6728211 -17.644985
C 13.9716254 12.728998 -16.7542726
H 14.876663 12.1276992 -16.6566322
H 13.9650252 13.5017634 -15.9855672
H 13.9695782 13.2008787 -17.7373648
N 11.9102878 11.1457732 -18.7984009
H 11.4901366 12.0606529 -18.8311174
C 11.6113386 10.2161504 -19.8849453
H 11.4683386 9.2575284 -19.3922061
C 12.7697246 10.041093 -20.8749029
O 13.4841971 10.9883786 -21.2103005
C 10.2956217 10.5834444 -20.6018401
H 9.5642602 10.8737195 -19.8541547
H 10.4613373 11.4376453 -21.2598264
C 9.7172444 9.4047201 -21.4196476
H 10.350257 9.2350742 -22.2929309
H 8.7362979 9.7002982 -21.7986705
C 9.5899082 8.089336 -20.6183328
O 8.4815808 7.7830257 -20.1380566
O 10.6205886 7.3866881 -20.4498584
N 12.8901662 8.8305339 -21.4204041
H 12.2017952 8.1247666 -21.1470783
C 13.7998552 8.4972353 -22.5092701
H 14.1771403 9.4236569 -22.9434863
C 13.0399482 7.741936 -23.6088019
O 12.2666108 6.8259826 -23.3460136
C 14.9936922 7.7225667 -21.9245386

H 15.5173435 8.3745069 -21.2235556
H 14.6001376 6.8863099 -21.3570609
C 16.0029798 7.189281 -22.9591753
H 15.4878043 6.568367 -23.6890142
C 16.7306364 8.3210255 -23.6896215
H 17.2504768 8.9574053 -22.9726436
H 16.0235264 8.9250527 -24.2541046
H 17.4549132 7.90003 -24.3877077
C 17.0509741 6.3236455 -22.2584349
H 17.5894864 6.9110609 -21.5149428
H 17.7563171 5.9395483 -22.9960493
H 16.5630007 5.4766885 -21.778768
N 13.2962296 8.1160457 -24.8611726
H 13.9627613 8.8548464 -25.0049569
C 12.7297022 7.4720004 -26.0467334
H 11.6552424 7.6561114 -26.0668372
C 12.963355 5.9483119 -26.0268958
O 14.0825531 5.4964434 -25.7932219
C 13.3669937 8.1475173 -27.2671617
H 13.1403425 9.2139322 -27.2266732
H 14.4513269 8.049724 -27.187207
C 12.9656811 7.6189319 -28.6307211
C 12.1494424 8.3971049 -29.4761888
H 11.769383 9.3526089 -29.1454154
C 11.8565891 7.9520163 -30.7798108
H 11.2562024 8.555452 -31.4472005
C 12.3673248 6.7178848 -31.2374061
O 12.1160781 6.2857259 -32.4969706
H 11.5991475 6.9488647 -33.0185918
C 13.1697677 5.9340538 -30.3847744
H 13.5492799 4.9896701 -30.7459872
C 13.4827043 6.3907892 -29.091865
H 14.1273567 5.791667 -28.4621886
N 11.8951892 5.1788313 -26.2599184
H 11.0304658 5.6427895 -26.4800882
C 11.8426092 3.7036502 -26.2137578
H 10.7811114 3.4599108 -26.2631227
C 12.3180866 3.0493723 -24.893297
O 12.588105 1.8486882 -24.8229072
C 12.4769853 3.1028593 -27.4818833
H 13.5610504 3.0598399 -27.3602348
H 12.2582179 3.7523311 -28.3311342
C 11.9188234 1.7098089 -27.8129528
O 10.6734795 1.5608478 -27.7641107
O 12.7303627 0.8270589 -28.169355
N 12.3786583 3.8077385 -23.7892188

H 12.1567416 4.7960764 -23.8565606
C 12.7236866 3.2518266 -22.4780661
H 13.5786598 2.5902866 -22.6181944
C 11.5733328 2.4149249 -21.9209309
O 10.5345585 2.9233827 -21.5059758
C 13.1586237 4.3717878 -21.5322934
H 14.0067916 4.8753226 -21.9927235
H 12.3398627 5.0838206 -21.4074609
C 13.5708913 3.8411374 -20.1519667
H 12.6903613 3.4453154 -19.6452956
H 14.3110593 3.0473288 -20.2626016
C 14.1595742 4.9730826 -19.3074994
H 13.477531 5.8273082 -19.3386531
H 15.1255757 5.265841 -19.7198963
C 14.3246592 4.5251755 -17.8558798
H 14.9647772 3.6393135 -17.8237746
H 13.3343658 4.2459127 -17.4769636
N 14.894404 5.6206571 -17.034387
H 15.8026652 5.9022239 -17.3743342
H 14.9585333 5.3479973 -16.0632593
H 14.281514 6.4323719 -17.0835563
N 11.8145313 1.1129027 -21.796752
H 12.636759 0.7428888 -22.2469702
C 10.915173 0.206206 -21.075752
H 9.9261211 0.3116122 -21.5233934
C 10.7879049 0.6022152 -19.5986625
O 11.7565775 1.0128309 -18.9601126
C 11.3345322 -1.270057 -21.22834
H 10.6296722 -1.8783407 -20.6606041
C 11.2418011 -1.7098514 -22.6941182
H 11.9608367 -1.1650701 -23.3087444
H 10.2395993 -1.5174528 -23.0780593
H 11.4468521 -2.7773516 -22.7753339
C 12.7458793 -1.5768076 -20.7095885
H 13.4907476 -1.0077613 -21.2656222
H 12.9570051 -2.6395233 -20.8284199
H 12.8214824 -1.3296274 -19.6506744
N 9.6090573 0.3641778 -19.0190877
H 8.8636369 0.042936 -19.61778
C 9.3030641 0.4995601 -17.5765515
H 8.2498576 0.2378134 -17.4888618
C 9.3464409 1.9102799 -16.9589934
O 9.2888891 2.018324 -15.7386553
C 10.0341763 -0.545724 -16.7016046
H 9.6693134 -0.4676256 -15.6770594
C 9.7794235 -1.9797159 -17.169852

H 10.215899 -2.1450359 -18.1540049
H 8.7079161 -2.1740235 -17.2032576
H 10.239578 -2.6699839 -16.4624216
O 11.4299275 -0.3613229 -16.691399
H 11.6667592 0.1678767 -17.4727227
N 9.2779939 2.9909397 -17.7450031
H 9.3809 2.8980378 -18.7455964
C 8.8924366 4.3186524 -17.2226461
H 9.484174 4.5459203 -16.3361664
C 7.3950813 4.3363773 -16.8420819
O 6.54833 4.1563548 -17.7157283
C 9.1737252 5.4060425 -18.2724269
H 8.8412563 6.3707971 -17.8859208
H 8.6096819 5.1895781 -19.1816057
O 10.5517975 5.4957348 -18.5958262
H 10.5894134 6.1754387 -19.3178923
N 7.0367162 4.4791013 -15.5515566
H 7.7637049 4.4744686 -14.8485576
C 5.6335974 4.372326 -15.0850094
H 5.0070584 4.6318068 -15.9400818
C 5.0839267 5.3356751 -13.9496445
O 4.1802507 4.8921545 -13.2441249
C 5.3501324 2.8742188 -14.7954481
H 5.6601937 2.2734319 -15.6495461
H 4.2770818 2.7385852 -14.6680957
C 6.0481724 2.3356799 -13.5563096
O 6.9355157 2.9397106 -12.9802825
N 5.6839717 1.1536912 -13.1130988
H 6.1721809 0.8278604 -12.2984982
H 4.9434468 0.6468731 -13.5561149
N 5.4209234 6.6581869 -13.7954432
H 6.1313111 7.0377768 -14.4037847
C 4.6727308 7.6690857 -12.8730958
H 3.6722803 7.2716519 -12.7031805
H 5.1665915 7.6425688 -11.902207
C 4.4605241 9.2667223 -13.2399755
O 5.4811072 9.6663177 -13.812121
N 3.2536409 10.1109433 -12.9872607
H 2.6474233 9.6614132 -12.3206553
C 2.3771526 11.4343133 -13.6922496
H 3.1658733 12.0715482 -14.0792353
C 1.0505375 12.8198719 -12.978889
O 0.738086 12.1140384 -12.0071596
C 1.9540762 10.4352165 -14.9544058
H 0.9329988 10.1097298 -14.746017
C 2.7417697 9.1177858 -15.5124699

H 3.7821969 9.3932306 -15.6797871
H 2.6711718 8.3582525 -14.7352443
C 1.9181844 11.302737 -16.1856385
H 2.9290002 11.590086 -16.4899791
H 1.3394983 12.1952761 -15.9637221
H 1.4171187 10.8031861 -17.0150758
C 2.3461044 8.3062146 -16.7841532
H 1.2838958 8.043668 -16.7411571
H 2.9411495 7.3919643 -16.8207085
H 2.5328117 8.8854332 -17.695951
N 0.2333932 14.4003043 -13.1834479
H 0.7437184 14.7653437 -13.9704046
C -1.2123799 15.955379 -12.9597387
H -1.0028175 16.3292374 -13.960482
C -2.5312399 15.1695335 -13.1010222
H -2.3258053 14.1092915 -13.2484796
H -3.0618878 15.2689053 -12.1555772
C -3.4175086 15.6977339 -14.2585728
H -3.2432872 16.7657087 -14.3985759
C -3.0783778 14.9714564 -15.5675978
H -3.2811317 13.903087 -15.4809576
H -2.0269104 15.1037593 -15.8111044
H -3.6757972 15.3801559 -16.3812018
C -4.9110054 15.512951 -13.9555661
H -5.1400087 14.4586235 -13.8201664
H -5.5040244 15.9052164 -14.7807417
H -5.172629 16.0607214 -13.0540348
C -2.0596333 17.9254177 -12.3430272
O -1.3832877 18.2475852 -11.374691
N -3.271554 19.2249776 -12.5340521
H -3.8017849 18.9469697 -13.3433045
C -4.1853409 20.9390259 -11.9780666
H -4.1201797 20.8378689 -10.8941409
C -5.8142116 21.9798425 -12.0685304
O -6.4577917 21.990306 -13.1238488
C -3.3553264 22.2522131 -12.3368714
H -2.7508435 21.9810248 -13.199742
H -3.9431639 23.1195955 -12.6376299
C -2.3935577 22.6639575 -11.2141059
H -1.5875927 23.2551301 -11.6539897
H -1.9417954 21.7737149 -10.7710014
C -3.1027948 23.4921229 -10.1365626
O -4.1338848 23.0043489 -9.6189633
O -2.6283562 24.6174618 -9.8712537
N -6.4475046 23.0719258 -11.2698861
H -5.8681384 23.316696 -10.4654281

C -7.9445496 23.8116708 -11.2481781
H -8.5313975 23.1447577 -11.8786809
C -8.4505552 25.3183528 -11.7282815
O -7.9562339 26.3541144 -11.2608184
C -8.5827225 23.7319299 -9.8142739
H -8.3098143 22.7644516 -9.3910452
H -8.1237068 24.4929663 -9.1820639
C -10.1128287 23.8553926 -9.699581
H -10.4295997 24.8665053 -9.9608487
H -10.3884539 23.7049408 -8.6526486
C -10.8425833 22.8299823 -10.5754585
O -10.9583405 23.1023724 -11.793048
O -11.2574013 21.7859035 -10.0265513
N -9.602907 25.5151302 -12.4609812
H -10.1970189 24.6947314 -12.6038089
C -10.0093518 26.7968804 -13.139427
H -9.6033406 27.6153906 -12.5465371
C -11.5373224 27.0770783 -13.3129338
O -12.3430523 26.1660776 -13.4651874
C -9.3158021 26.7963794 -14.5218177
H -8.2652178 26.5407625 -14.3723088
H -9.7616841 25.9988297 -15.1187812
C -9.3656862 28.1006173 -15.3396433
H -10.3980501 28.3527717 -15.5746801
C -8.7151959 29.2767986 -14.6053596
H -7.6855865 29.0272619 -14.3458923
H -9.2616884 29.5103425 -13.6946444
H -8.721151 30.1580805 -15.2468748
C -8.6202634 27.902131 -16.6598573
H -7.5714266 27.6723762 -16.4704162
H -8.6904418 28.808201 -17.2612677
H -9.0736317 27.0808258 -17.214682
N -11.9412789 28.3572898 -13.4297045
H -11.2467902 29.0828588 -13.3712445
C -13.3321225 28.7589476 -13.708797
H -13.9922764 28.0787705 -13.1669539
C -13.6973559 28.668285 -15.20173
O -13.2212429 29.4599915 -16.0166546
C -13.5976231 30.1744778 -13.1694646
H -13.3339687 30.2032292 -12.1112647
H -12.9526566 30.8857408 -13.6884859
C -15.0439728 30.6091374 -13.3172145
C -16.0007793 30.2075446 -12.3654889
H -15.7026398 29.6095012 -11.5164327
C -17.3497155 30.5734271 -12.5229045
H -18.084102 30.2600018 -11.7942258

C -17.7459906 31.3401123 -13.632773
H -18.784204 31.6156339 -13.7569758
C -16.7930155 31.742311 -14.5848431
H -17.0984245 32.326439 -15.4416514
C -15.4440982 31.3771052 -14.4276294
H -14.7165289 31.6734974 -15.1713742
N -14.6326318 27.780819 -15.5396291
H -14.9521194 27.1150618 -14.8471413
C -15.2390964 27.6886577 -16.8666391
H -15.735075 28.6312197 -17.0994544
H -14.4624243 27.5134405 -17.6111349
C -16.2666536 26.5596064 -16.957317
O -16.3953891 25.7524785 -16.0401148
N -16.9764725 26.4878918 -18.0819378
H -16.8289854 27.176356 -18.8044725
C -17.9575033 25.4408391 -18.3831007
H -17.7899232 24.5827215 -17.7304426
C -17.7792012 24.9927546 -19.8397808
O -17.7959146 25.8188285 -20.750926
C -19.3742105 25.9740925 -18.1133224
H -19.4072767 26.3626889 -17.0943744
H -19.5911057 26.7896238 -18.8053619
C -20.4418095 24.8784784 -18.2588574
H -20.2170614 24.0779836 -17.5490798
H -20.3816661 24.4589508 -19.2655484
C -21.873747 25.3850101 -18.0153021
O -22.0329252 26.4946208 -17.4568844
O -22.8044396 24.6367793 -18.3888393
N -17.5576422 23.6953514 -20.0649298
H -17.6282623 23.0479037 -19.293315
C -17.3269048 23.1256682 -21.396853
H -17.6295227 23.8524687 -22.1539443
C -18.173276 21.8705255 -21.6133325
O -18.4492201 21.1344351 -20.6665456
C -15.8303326 22.833808 -21.5971231
H -15.5031065 22.1074362 -20.8521036
H -15.2664698 23.755845 -21.4523805
C -15.5235885 22.3038595 -22.9769314
N -16.0573017 22.8083599 -24.1656877
C -15.6125519 22.0028993 -25.140073
H -15.854249 22.1181749 -26.1888167
N -14.8449621 21.02673 -24.6277346
H -14.429788 20.2755673 -25.1579691
C -14.7816493 21.1953241 -23.2621255
H -14.2791179 20.561661 -22.5447457
N -18.5185024 21.5818415 -22.8701808

H -18.1432063 22.1738025 -23.6009505
C -19.2288161 20.3661674 -23.257331
H -19.9116933 20.1067755 -22.4457962
C -18.2431347 19.2015468 -23.4437471
O -17.5034608 19.1367934 -24.4310812
C -20.0753688 20.6536823 -24.5101802
H -20.7622502 21.4721288 -24.2876809
H -19.4111275 20.9769363 -25.3134978
C -20.8899068 19.4416545 -25.0031907
H -20.2061397 18.632868 -25.252551
C -21.8900833 18.9305006 -23.9653095
H -22.5664824 19.7301243 -23.6638583
H -21.3716057 18.543429 -23.0898099
H -22.4721546 18.1090986 -24.387707
C -21.6558409 19.8178908 -26.2704486
H -22.3670983 20.6156827 -26.0549008
H -22.1966695 18.9460329 -26.6408658
H -20.9593117 20.149503 -27.0399621
N -18.305586 18.2264973 -22.5363312
H -18.9848484 18.3084584 -21.7879638
C -17.4987295 17.0022879 -22.56464
H -16.4445225 17.2767669 -22.5352472
C -17.7136686 16.1851165 -23.8479637
O -16.7324037 15.7061853 -24.4089253
C -17.8183622 16.1676056 -21.3118248
H -17.6392762 16.7679502 -20.4189096
H -17.1541192 15.3031549 -21.279748
C -19.2469521 15.6902349 -21.2993199
N -20.3555822 16.5291505 -21.1770985
C -21.409785 15.8002387 -21.5780685
H -22.404937 16.1941265 -21.7444035
N -21.0189659 14.5690479 -21.940586
H -21.5461555 13.999855 -22.611493
C -19.6585309 14.4792161 -21.7690455
H -19.0246639 13.6887216 -22.1457658
N -18.9342631 16.1416122 -24.3967885
H -19.7139306 16.4659898 -23.8383305
C -19.2306073 15.4622993 -25.6698397
H -18.9191364 14.4191048 -25.5943322
C -18.4740459 16.0915514 -26.8531044
O -17.994577 15.3816032 -27.732199
C -20.7422481 15.4827005 -25.9605596
H -20.9140981 15.0341016 -26.9402223
H -21.0845436 16.5175317 -26.0055241
C -21.5715667 14.7083567 -24.9337949
O -21.1731487 13.5796127 -24.57134

O -22.5721805 15.2767049 -24.4473625
N -18.2496141 17.4112082 -26.8323886
H -18.5716361 17.9329869 -26.0288096
C -17.4531297 18.1047217 -27.8489587
H -17.7977212 17.8135617 -28.8424323
H -17.5879439 19.1789559 -27.7304308
C -15.956921 17.7942072 -27.7454374
O -15.2888172 17.6280449 -28.7627028
N -15.439025 17.6201509 -26.5247796
H -16.0609943 17.7043393 -25.7294654
C -14.0743246 17.1266898 -26.3031017
H -13.3826248 17.7038566 -26.9186169
C -13.9256189 15.6534948 -26.724003
O -12.9564171 15.2990751 -27.3953963
C -13.702554 17.3422062 -24.8291262
H -13.7609572 18.4063683 -24.6038623
H -14.431762 16.8391893 -24.1953113
C -12.3186415 16.8515113 -24.4543145
C -11.189853 17.6482056 -24.7279804
H -11.3040053 18.609059 -25.2098121
C -9.9051194 17.1911998 -24.3777829
H -9.0312386 17.792005 -24.5808524
C -9.7460546 15.9368061 -23.7535044
O -8.5019852 15.5094553 -23.4137942
H -8.5099345 14.6569228 -22.9779248
C -10.8775819 15.1370117 -23.4808996
H -10.7593813 14.1747477 -23.0085044
C -12.161335 15.5959257 -23.8337612
H -13.0275541 14.9793858 -23.6353157
N -14.9268359 14.8201123 -26.423841
H -15.7014839 15.176822 -25.8741997
C -14.9828394 13.4197593 -26.8408227
H -14.0769896 12.9181517 -26.4986893
C -15.0310294 13.2711907 -28.3685764
O -14.316041 12.4302505 -28.9112801
C -16.1878629 12.7409781 -26.1724631
H -16.0987507 12.8425083 -25.0898935
H -17.1015123 13.2506904 -26.4752072
C -16.3194695 11.2716038 -26.5208461
C -15.679925 10.2960364 -25.7306851
H -15.1296517 10.5903453 -24.8487413
C -15.7427155 8.9385754 -26.1018896
H -15.2470437 8.1817352 -25.5143806
C -16.4327316 8.5582085 -27.2723703
O -16.4601685 7.2539103 -27.6505408
H -16.8895214 7.1591939 -28.5014005

C -17.087672 9.5346431 -28.0532165
H -17.603424 9.2540987 -28.9595907
C -17.035941 10.887789 -27.6716777
H -17.5222426 11.6367727 -28.2851494
N -15.7781968 14.1209507 -29.0785084
H -16.4213335 14.735133 -28.58639
C -15.8302485 14.0991621 -30.5458371
H -16.023302 13.0723458 -30.857743
C -14.4935853 14.5210283 -31.1785911
O -13.967663 13.81329 -32.0333803
C -16.994066 14.9750367 -31.0424011
H -17.90762 14.6865268 -30.519727
H -16.7819786 16.0183928 -30.8031416
C -17.238316 14.8508303 -32.5555673
H -16.3290961 15.1177248 -33.0992785
H -18.0038016 15.5779021 -32.8375267
C -17.6986865 13.4395358 -32.9540066
O -18.9264795 13.2539991 -33.097649
O -16.8225467 12.5548429 -33.0888592
N -13.837982 15.5729289 -30.6701081
H -14.2892367 16.1201696 -29.9440593
C -12.4898729 15.9648231 -31.1351682
H -12.5346458 16.1337827 -32.2113024
C -11.4694612 14.8360737 -30.9194148
O -10.6875397 14.5295424 -31.8238719
C -12.0510367 17.2817301 -30.4618222
H -12.2135606 17.2075124 -29.385962
C -10.5745282 17.6261432 -30.7001145
H -10.360449 17.6415137 -31.7693089
H -9.9326989 16.8899406 -30.2164525
H -10.3471552 18.6032195 -30.2737645
C -12.8768666 18.454303 -31.009272
H -12.6844 18.5843449 -32.0745506
H -12.6143381 19.3701809 -30.4802161
H -13.9403605 18.2682826 -30.8668087
N -11.5418689 14.1369177 -29.7810758
H -12.2068198 14.4377171 -29.0741423
C -10.7457172 12.934077 -29.5229422
H -9.6880299 13.1653715 -29.6509979
H -10.9128238 12.6231638 -28.4919327
C -11.1015855 11.7534031 -30.4368387
O -10.2052046 11.0525872 -30.9091058
N -12.3806552 11.5703557 -30.7665667
H -13.0669852 12.1981408 -30.3596135
C -12.8830576 10.4904933 -31.6335773
H -12.4536585 9.5465298 -31.299618

C -12.4546029 10.6930136 -33.0856704
O -11.8379689 9.7967565 -33.662114
C -14.4115139 10.3664975 -31.5108397
H -14.8837716 11.3195435 -31.7430037
C -15.0075931 9.281508 -32.405127
H -14.497612 8.3332624 -32.2385487
H -14.9184434 9.5764776 -33.4497101
H -16.0681325 9.1738969 -32.1753859
O -14.7191285 9.9990169 -30.1858573
H -14.6611214 10.8041515 -29.6445041
N -12.6061399 11.9050074 -33.6278135
H -13.1277568 12.5991368 -33.0943308
C -12.1108057 12.2899864 -34.96069
H -12.5626445 11.6330904 -35.7039491
C -10.5924574 12.1086371 -35.0606404
O -10.1118234 11.5017037 -36.0194828
C -12.5253388 13.7419652 -35.2846723
H -12.2047179 14.3950538 -34.4716908
C -11.9014053 14.2583629 -36.5896139
H -12.1485647 13.587198 -37.4129321
H -10.8182307 14.3273054 -36.4922998
H -12.2846337 15.2548982 -36.8119629
C -14.0473735 13.8569901 -35.4397281
H -14.390893 13.2481522 -36.2760117
H -14.3288191 14.8958381 -35.6094773
H -14.5551193 13.5168148 -34.5364626
N -9.8327613 12.5183483 -34.0376294
H -10.2691124 13.0158676 -33.2677802
C -8.3866883 12.2947826 -33.9964652
H -7.94803 12.713808 -34.9032965
C -8.0253687 10.7951613 -33.964585
O -7.1750255 10.3535353 -34.7385592
C -7.808095 13.0498299 -32.7940855
H -8.2322251 12.6640796 -31.8665257
H -8.0425363 14.1119177 -32.8768195
H -6.7249933 12.9258191 -32.7693519
N -8.7383063 9.9928143 -33.1690346
H -9.4399586 10.4216383 -32.5757625
C -8.5261026 8.5373236 -33.0519002
H -7.476906 8.3564513 -32.8190178
C -8.836892 7.7959867 -34.3557814
O -8.0721417 6.9214099 -34.7582541
C -9.3671494 7.960609 -31.9009351
H -10.4270834 8.1175921 -32.1029293
C -9.1210937 6.4714297 -31.6602582
H -8.0569718 6.2924769 -31.5037895

H -9.4663804 5.8883818 -32.5134126
H -9.6722286 6.1524028 -30.7752524
O -9.0287859 8.6085484 -30.6970051
H -9.4300372 9.4946216 -30.711502
N -9.8765834 8.2071453 -35.0894348
H -10.4751493 8.9342993 -34.7066042
C -10.218649 7.6484065 -36.4087586
H -10.3078933 6.565189 -36.3149444
C -9.0961985 7.9031619 -37.4291875
O -8.8107229 7.0319327 -38.2457573
C -11.5883493 8.2058925 -36.8724934
H -11.5800876 9.2895272 -36.7418409
C -11.8649231 7.9080697 -38.3605028
H -11.8182208 6.8336763 -38.5433383
H -11.1330206 8.4106419 -38.9928817
H -12.8465962 8.2794081 -38.6492608
C -12.7194695 7.6055139 -36.002287
H -12.8725628 6.5606242 -36.275951
H -12.4291906 7.6247014 -34.9527788
C -14.0547499 8.3529914 -36.1158443
H -14.469557 8.2558855 -37.1180749
H -13.9111854 9.4081356 -35.8800284
H -14.765007 7.9268607 -35.4065953
N -8.3937191 9.0388911 -37.3367375
H -8.6846259 9.7176446 -36.6427142
C -7.265239 9.3812153 -38.2229573
H -7.5533014 9.1179589 -39.2408252
C -5.9497281 8.6286735 -37.9283472
O -4.9741865 8.7465544 -38.6840646
C -7.0390962 10.899473 -38.1899562
H -8.0014138 11.4136505 -38.2093944
H -6.4800146 11.1924024 -39.0771632
O -6.2990547 11.3175345 -37.05805
H -6.6658562 10.914714 -36.2473093
N -5.8900761 7.8921141 -36.8100548
H -6.7249579 7.8243468 -36.2397125
C -4.6565738 7.3302429 -36.256341
H -3.8362244 8.0007177 -36.5123651
C -4.2800623 5.9408626 -36.8074929
O -3.1352983 5.5205919 -36.6491747
C -4.7709993 7.3240925 -34.7269169
H -5.5236596 6.6026775 -34.4107831
H -5.0474193 8.3167635 -34.3677705
H -3.8122191 7.0434425 -34.2890576
N -5.1800992 5.2659515 -37.5310566
H -6.0659794 5.7270073 -37.7010793

C -4.9863126 3.9392569 -38.1512939
H -5.0038412 3.1815968 -37.3671395
C -3.6467069 3.7571432 -38.8886477
O -2.9731921 2.7386014 -38.7264792
C -6.1697208 3.6740353 -39.1048049
H -7.0402153 3.4328757 -38.4935022
H -5.9499646 2.7970377 -39.7150907
C -6.5637775 4.8197352 -40.0201111
N -5.7699764 5.9207787 -40.3641479
C -6.5674691 6.752385 -41.049963
H -6.277414 7.7318812 -41.4028678
N -7.7926363 6.2244398 -41.1826337
H -8.5854946 6.6964794 -41.5940588
C -7.8129371 5.0107498 -40.5332027
H -8.6720512 4.3747877 -40.3736965
N -3.1845526 4.7824287 -39.6094995
H -3.8397485 5.5490973 -39.7402001
C -1.9094941 4.7790629 -40.3503428
H -1.9058746 3.9210138 -41.0259016
C -0.6755178 4.6000095 -39.4590681
O 0.3500762 4.1523355 -39.9634635
C -1.8108003 6.0655976 -41.1818839
H -1.9802055 6.9149351 -40.5182176
H -0.8101594 6.155225 -41.6088882
C -2.8366963 6.0737128 -42.3295833
H -3.7805566 5.6467642 -41.9998432
H -2.4675716 5.4691415 -43.158521
C -3.090666 7.5062857 -42.7975055
H -2.21557 7.8834898 -43.328123
H -3.2604295 8.1357064 -41.9227019
N -4.2898586 7.6146451 -43.6353146
H -5.0985667 8.0752973 -43.2163844
C -4.3632322 7.5927761 -44.9465972
N -3.4118636 7.0991039 -45.680707
H -3.2172373 7.5904414 -46.5573116
H -2.6560279 6.644648 -45.2205878
N -5.4028833 8.1065841 -45.5234574
H -6.0523492 8.6180683 -44.9202338
H -5.4423454 8.1830156 -46.5185052
N -0.7546542 4.8641941 -38.1528525
H -1.6478001 5.1514024 -37.7665882
C 0.3441051 4.6164829 -37.2169392
H 1.2323201 5.1264964 -37.5885598
C 0.6996833 3.1210416 -37.0906775
O 1.8637562 2.7984179 -36.8587498
C -0.0133202 5.2297426 -35.858726

H -0.9017486 4.748926 -35.4486878
H -0.2065543 6.2971976 -35.9704315
H 0.8169899 5.0909097 -35.1647239
N -0.2423564 2.2117153 -37.3765593
H -1.1831707 2.5200405 -37.5942453
C 0.0441412 0.7786215 -37.4595675
H 0.5673747 0.4724402 -36.5515695
C 0.9552045 0.4341165 -38.6550826
O 1.7906982 -0.4590169 -38.5465271
C -1.2872208 0.0221727 -37.522223
H -1.8167315 0.2672544 -38.4447155
H -1.9079066 0.2944651 -36.668599
H -1.1024213 -1.0504495 -37.4916568
N 0.8781965 1.1977947 -39.7544587
H 0.2102942 1.9555281 -39.7611379
C 1.8112123 1.0873253 -40.8905783
H 2.0039892 0.0301488 -41.0804667
C 3.1532747 1.7347408 -40.5479506
O 4.1918602 1.1211358 -40.7786454
C 1.2242149 1.7004207 -42.1796402
H 1.0712918 2.7691655 -42.0362597
C 2.1680245 1.5163423 -43.3761676
H 2.3959256 0.458749 -43.5151264
H 3.0968456 2.0616945 -43.2111974
H 1.7047064 1.9050683 -44.2829471
C -0.1270984 1.0684292 -42.5415657
H -0.0134202 -0.0094525 -42.6618157
H -0.4995357 1.4948188 -43.4727939
H -0.8607535 1.2658962 -41.7604837
N 3.1497115 2.9075864 -39.8983128
H 2.2584563 3.3646896 -39.7477886
C 4.3829764 3.5769137 -39.4271644
H 5.0316617 3.7532182 -40.287047
C 5.1714679 2.6870662 -38.4572727
O 6.3914124 2.6466122 -38.5342007
C 4.0854947 4.9447377 -38.7734971
H 3.4492565 4.7868126 -37.9038288
C 5.3579813 5.667574 -38.3077099
H 6.0473034 5.7700222 -39.1479344
H 5.8520541 5.0988091 -37.5198768
H 5.1091859 6.6515776 -37.9116408
C 3.3657123 5.9094878 -39.7292705
H 4.0142422 6.146524 -40.5715909
H 3.1117469 6.8290265 -39.2030367
H 2.4524687 5.4645623 -40.1099666
N 4.5099699 1.8691594 -37.6261136

H 3.501018 1.9661057 -37.5679173
C 5.1768327 0.8861925 -36.7517641
H 5.9189905 1.4157616 -36.1524491
C 5.9655105 -0.1805465 -37.5278367
O 6.9655235 -0.6580613 -37.005066
C 4.1151826 0.2654933 -35.821984
H 3.6872668 1.0584642 -35.2070059
H 3.3115988 -0.1409395 -36.4366679
C 4.6026419 -0.8663175 -34.8987054
H 4.9701494 -1.6946528 -35.5041773
H 3.7425868 -1.2253198 -34.3369632
C 5.691348 -0.4487287 -33.8979481
H 5.305648 0.3329929 -33.2411631
H 6.5347315 -0.0299299 -34.4515579
N 6.1852786 -1.5988657 -33.1065998
H 7.0302672 -2.023504 -33.4565587
C 5.6931212 -2.0929878 -31.9796119
N 4.6137617 -1.6238221 -31.4121932
H 4.2496792 -2.0315972 -30.5644291
H 4.1500759 -0.8388203 -31.8268032
N 6.2826626 -3.0960025 -31.392016
H 7.158683 -3.4460252 -31.7437805
H 5.9220268 -3.4384356 -30.5210906
N 5.5130667 -0.5786026 -38.7196586
H 4.6989518 -0.1098716 -39.0946508
C 6.155844 -1.6215128 -39.5477068
H 6.7171868 -2.2989085 -38.9044692
C 7.1870424 -1.0471198 -40.5141252
O 8.1672071 -1.7115576 -40.8379074
C 5.0729423 -2.429844 -40.2970744
H 4.4719049 -1.7392024 -40.8919679
C 5.6505611 -3.4778044 -41.2668926
H 6.3652394 -4.1194898 -40.7503349
H 6.1577817 -2.9777053 -42.0922651
H 4.8537515 -4.0898952 -41.6888069
C 4.1272498 -3.1381766 -39.3001663
H 3.4521743 -2.3981917 -38.8745679
H 3.519856 -3.8567523 -39.8472324
C 4.8022508 -3.8790266 -38.1377464
H 5.1620025 -3.1674568 -37.3956499
H 5.6444629 -4.4611102 -38.5038849
H 4.0797422 -4.5405924 -37.6627072
N 6.9634562 0.1827065 -40.9660073
H 6.1059518 0.645179 -40.6903566
C 7.8918005 0.9019409 -41.8307362
H 8.3560989 0.1968688 -42.5199967

C 9.0541694 1.5083209 -41.0392823
O 10.1751688 1.4175308 -41.5113396
C 7.0910835 1.9372281 -42.6425295
H 6.4755174 2.5141194 -41.9494156
C 8.015055 2.9405889 -43.3353853
H 8.8170607 2.4130698 -43.8517664
H 8.4436482 3.6079035 -42.5859543
H 7.4537262 3.5413332 -44.0376991
C 6.1329575 1.2547909 -43.6511673
H 5.3388 0.7519175 -43.1009127
H 5.6559528 2.0240523 -44.2518133
C 6.7607953 0.2206656 -44.5975052
H 7.0017951 -0.6915304 -44.0477814
H 7.6692296 0.6123557 -45.0487245
H 6.0481605 -0.0291608 -45.3837918
N 8.798992 1.9819869 -39.8195457
H 7.8269675 1.9509433 -39.5448266
C 9.6149082 2.8267209 -38.9312898
H 9.2170332 2.5797365 -37.9459199
C 9.3250434 4.3409519 -39.0306445
O 8.5238253 4.7993794 -39.8542185
C 11.099113 2.4020543 -38.8279642
H 11.7082873 3.1408157 -39.3434734
H 11.2510327 1.4269978 -39.2904088
C 11.5965131 2.3038259 -37.3833269
O 11.4492724 3.3205405 -36.6703128
O 12.1249463 1.2330349 -37.0094698
N 9.8588958 5.1058401 -38.0754761
H 10.516748 4.6466534 -37.4396315
C 9.3325992 6.4041998 -37.6512809
H 8.3526531 6.2042132 -37.2199303
C 9.1395831 7.4234129 -38.7896545
O 10.0494829 7.7492656 -39.5515223
C 10.2024572 6.9760756 -36.5200309
H 10.3373002 6.2059069 -35.7593743
H 11.1814432 7.2466722 -36.9189787
C 9.5601829 8.2096529 -35.8657287
H 8.5933201 7.9247972 -35.443284
H 9.3792816 8.9717041 -36.6261681
C 10.4478246 8.8008571 -34.7647909
O 10.8020372 8.0456266 -33.8314376
O 10.7428137 10.012847 -34.8410345
N 7.9356494 8.0039627 -38.8202964
H 7.2747294 7.6949909 -38.1276181
C 7.4979327 9.066309 -39.7246913
H 6.4475177 9.2219085 -39.4784392

C 7.4607978 8.7250306 -41.2328113
O 7.2182063 9.6195186 -42.0501456
C 8.189382 10.3821855 -39.339387
H 9.2533861 10.3291555 -39.5703739
H 8.0772996 10.5585665 -38.2684137
H 7.7299772 11.2135753 -39.8729389
N 7.5456185 7.4423073 -41.6155564
H 7.7541 6.7354562 -40.9135248
C 7.344726 6.9801426 -42.997353
H 6.9802142 7.8289962 -43.5714683
C 6.2228111 5.9398072 -43.1423331
O 5.8136015 5.298277 -42.1811172
C 8.7040589 6.5417867 -43.5937623
H 9.3769214 6.2789443 -42.781539
H 8.5857406 5.6690598 -44.2357264
C 9.2911549 7.7100855 -44.425368
H 9.1907816 8.6410518 -43.8675988
C 10.7676023 7.5379856 -44.7641775
H 10.9094523 6.6899523 -45.4326336
H 11.347273 7.4053009 -43.8535032
H 11.1408564 8.4293545 -45.2677901
C 8.5546316 7.8484516 -45.7653518
H 8.6226347 6.9085588 -46.3141622
H 9.0244539 8.6267692 -46.3653165
H 7.5122898 8.121164 -45.6183874
N 5.6747873 5.8134553 -44.3622881
H 6.051826 6.3868802 -45.1032308
C 4.5891328 4.8649351 -44.6980872
H 4.5631062 4.1139725 -43.9051263
C 4.8447935 4.0459821 -45.973334
O 3.9017478 3.6498304 -46.6520897
C 3.2105171 5.5603428 -44.666108
H 2.4533252 4.7738203 -44.6831793
H 3.1054279 6.0864467 -43.7153432
C 2.9161759 6.5454592 -45.8179066
H 3.1261552 6.0697121 -46.7735315
H 3.5448542 7.4315362 -45.7241825
C 1.4308664 6.9484155 -45.7776237
H 1.2590212 7.5693356 -44.8966521
H 0.8212512 6.0482975 -45.6740764
N 0.981203 7.6811794 -46.9780385
H 0.7524881 8.6673509 -46.8923419
C 0.6228584 7.177358 -48.1443321
N 0.8186153 5.929421 -48.4685675
H 0.5752193 5.6132175 -49.3870961
H 1.388143 5.3577715 -47.8677134

N 0.0406873 7.949872 -49.0092621
H -0.2933387 8.855375 -48.6610897
H -0.2623347 7.6163466 -49.9005672
N 6.1190758 3.8578277 -46.3209588
H 6.8301691 4.1363819 -45.6635574
C 6.5958218 3.2428633 -47.5626859
H 5.830005 2.5719029 -47.9542416
C 7.8916732 2.4367347 -47.343168
O 8.5668267 2.64766 -46.3401455
C 6.8674228 4.3491416 -48.5851797
H 7.7970464 4.8464069 -48.304794
H 7.032437 3.8770175 -49.5533758
C 5.793543 5.4093771 -48.7466088
C 4.5694425 5.071977 -49.3430479
H 4.3617759 4.0434651 -49.5976387
C 3.6563468 6.0786253 -49.6922932
H 2.7837729 5.8310304 -50.2772817
C 3.9157208 7.4151039 -49.3467661
H 3.2349086 8.196869 -49.6514519
C 5.0976506 7.7468283 -48.6644446
H 5.3156042 8.7854837 -48.4512446
C 6.0470079 6.7471097 -48.3865945
H 6.9997683 7.0245668 -47.966154
N 8.2414517 1.5676536 -48.3001291
H 7.6597218 1.5224626 -49.118932
C 9.3979198 0.6578697 -48.2914649
H 9.1016447 -0.2646165 -47.791358
C 10.6602547 1.1969945 -47.5742744
O 11.1786285 2.245083 -47.9427162
C 9.7087369 0.3224341 -49.75895
H 9.967396 1.234773 -50.3010873
H 8.8483582 -0.1504811 -50.2328802
H 10.5608699 -0.3531544 -49.8204047
N 11.1058984 0.4683517 -46.5404187
H 10.6020477 -0.3756197 -46.3181145
C 12.3498292 0.6411544 -45.7620579
H 12.0842348 0.2321049 -44.78769
C 12.7677959 2.0582833 -45.3295639
O 13.9257997 2.2966503 -44.965395
C 13.488643 -0.2542563 -46.2966031
H 14.4106292 0.3125754 -46.3885813
H 13.233996 -0.5667301 -47.3062372
C 13.7404825 -1.5012609 -45.4212878
O 12.782663 -1.9446545 -44.7479203
O 14.8558588 -2.0616043 -45.4835954
N 11.7676098 2.9221305 -45.1175289

H 10.8663841 2.6705479 -45.5032949
C 11.8434355 4.1137479 -44.2565654
H 10.9702733 4.7131008 -44.4953178
C 13.0474841 5.0013958 -44.5690478
O 13.2566867 5.4146228 -45.7033659
C 11.6839069 3.7215239 -42.7654516
H 10.7042933 3.2772 -42.7433744
C 12.7172875 2.6995277 -42.243958
H 13.7411237 3.021697 -42.4103113
H 12.5627012 1.7344082 -42.7242798
H 12.5786212 2.5252549 -41.1832717
C 11.6078686 4.8907969 -41.7666777
H 12.5088229 4.963613 -41.1569353
H 10.7748965 4.7152793 -41.0967139
H 11.4403098 5.837781 -42.2673589
N 13.7997814 5.3551081 -43.5357305
H 13.5427582 4.9757027 -42.6392564
C 14.9722896 6.1999847 -43.5773004
H 14.7513394 7.1316738 -44.1038787
C 16.1325397 5.5277022 -44.3124657
O 16.971215 6.2121722 -44.8791594
C 15.3363167 6.5059358 -42.1167684
H 14.5993989 7.1982355 -41.7073855
H 15.2826306 5.5845827 -41.5328418
C 16.7149966 7.0988042 -41.9408267
C 16.9780411 8.3984896 -42.403626
H 16.1908624 8.9809401 -42.8654804
C 17.7536204 6.3218351 -41.3924162
H 17.5600729 5.3123153 -41.0611771
C 18.2759221 8.9240865 -42.299878
H 18.4802673 9.9218835 -42.6403416
C 19.0546992 6.8452967 -41.3078177
H 19.8555147 6.2433117 -40.90308
C 19.314826 8.149128 -41.7601691
H 20.3159865 8.5514257 -41.6963891
N 16.2058513 4.196207 -44.3268838
H 15.3924863 3.6449569 -44.0809636
C 17.3365519 3.5317345 -44.9880782
H 18.247824 4.0148928 -44.6328091
C 17.3362498 3.7162578 -46.5202865
O 18.411362 3.6889561 -47.1212283
C 17.4139431 2.0551039 -44.544089
H 17.2640812 2.0186564 -43.4640312
C 18.7685605 1.4097662 -44.8475106
H 18.7772956 0.3945679 -44.4540022
H 19.5699411 1.9849547 -44.3903993

H 18.9200973 1.3641078 -45.9264033
O 16.4573901 1.2286298 -45.1599023
H 15.5577683 1.5969757 -45.0914068
N 16.1818698 4.0690221 -47.1078517
H 15.3465728 4.1192239 -46.5422669
C 16.0387294 4.3659216 -48.539581
H 16.9320402 3.9392428 -48.9945931
C 16.0907108 5.8966401 -49.0803814
O 16.0794458 5.9767522 -50.2908172
C 14.8556147 3.4957892 -49.0773819
H 13.9798023 4.1366509 -49.1933825
H 14.5968292 2.7346661 -48.3378472
C 15.1275989 2.7525254 -50.4024108
O 14.1400315 2.2286328 -50.965854
O 16.307982 2.6299838 -50.8096799
N 16.2900343 7.1238414 -48.4203981
H 16.4497442 7.0131143 -47.4277553
C 16.1613041 8.688626 -48.8951202
H 15.5643498 8.6270493 -49.8051702
C 17.4543756 9.8556596 -49.3389581
O 18.4787587 9.2741032 -49.0634247
C 15.1839224 9.2297703 -47.7945255
H 14.6070166 8.4267525 -47.3302822
H 14.4664933 9.8869038 -48.2885934
C 15.9020718 10.0680588 -46.713811
O 16.3872989 11.1886415 -47.009515
O 15.9809798 9.6471257 -45.537687
N 17.6582507 11.2710311 -50.0187799
H 16.7432841 11.6473397 -50.215925
C 18.9196021 12.4999217 -50.4996121
H 19.4239745 12.7072473 -49.557024
C 19.8910586 11.6284874 -51.3214552
H 19.3040143 10.926469 -51.916006
H 20.4598406 12.2416738 -52.0211191
C 20.8996192 10.8577388 -50.4511839
H 20.4908512 10.6758339 -49.4595413
H 21.799341 11.4600084 -50.3269145
C 21.2793501 9.5187183 -51.0747255
O 22.2065281 9.4215954 -51.8603748
N 20.5592735 8.459898 -50.7803827
H 20.794535 7.5910496 -51.2218482
H 19.7724068 8.5434667 -50.1355936
C 19.092366 14.182846 -51.3913657
O 18.0971829 14.350783 -52.0856275
N 20.1085432 15.3856543 -51.5493382
H 20.9260687 15.212857 -50.986138

C 20.2948739 16.9461427 -52.3131094
H 20.2227503 16.7196393 -53.3770916
C 21.4078328 18.3179152 -52.2406162
O 22.5468109 18.183726 -51.7988277
C 19.0686253 17.7464106 -51.9697146
H 19.2338989 18.2728335 -51.0391949
H 18.2005998 17.0932304 -51.9204808
H 18.8667864 18.4802587 -52.7438545
N 21.1702238 19.6749261 -52.6514902
H 20.2345851 19.8379585 -52.9935248
C 22.0024176 21.0115075 -52.4479166
H 22.8683875 20.7431257 -51.8441611
C 21.2832516 22.1579803 -51.6588977
O 20.098186 22.3880363 -51.8856444
C 22.5113401 21.7661131 -53.7349204
H 21.6390627 22.0773934 -54.3111731
H 23.0283835 22.6774737 -53.4283394
C 23.4507222 21.0203275 -54.6889031
H 22.9115143 20.1707727 -55.1106251
H 23.7236569 21.6915994 -55.5045992
C 24.7266258 20.5101083 -54.0066815
H 24.4530278 19.8220364 -53.2042071
H 25.2791937 21.3544152 -53.5879837
C 25.5984771 19.7681775 -55.0268131
H 25.909443 20.4728439 -55.8051529
H 24.9888066 18.9920389 -55.5006992
N 26.7854235 19.1515691 -54.3842775
H 27.3653918 19.8580064 -53.9512649
H 27.3408378 18.6543916 -55.0695705
H 26.4940335 18.4923601 -53.6723818
N 22.0141776 22.9790716 -50.8776924
H 22.9848334 22.7561847 -50.7481297
C 21.4363151 24.0595014 -50.0406279
H 20.6651534 23.5880877 -49.4217957
C 20.6932301 25.1571828 -50.8434591
O 21.1123859 25.4928957 -51.9501055
C 22.4954675 24.6021902 -49.0576117
H 21.9681595 25.0697176 -48.2281703
H 23.0446883 23.7611733 -48.6298199
C 23.4967813 25.6136439 -49.6395768
H 24.1308012 25.1185841 -50.3761876
H 22.9616084 26.426131 -50.1294037
C 24.3600993 26.2058559 -48.5122502
H 25.0695733 25.4548231 -48.159759
H 23.7129745 26.4715583 -47.6719088
C 25.1007745 27.47991 -48.9414417

H 25.6487684 27.8643149 -48.0751497
H 24.3538345 28.231996 -49.2207631
N 26.033051 27.2429212 -50.0717632
H 26.729265 26.5557402 -49.8134378
H 26.5058074 28.1019389 -50.3270792
H 25.5216444 26.9054137 -50.8778729
N 19.6653497 25.7902966 -50.2502701
C 18.5260935 26.4199977 -50.9302971
H 17.683172 26.2611953 -50.25573
C 17.9666354 25.9230463 -52.2825018
O 17.2120042 26.6858309 -52.8855268
C 18.8245867 27.9163968 -50.8994079
H 19.6064095 28.1600803 -51.6210341
H 17.9311673 28.5198536 -51.0658878
C 19.9982801 26.7186724 -49.1631419
H 19.6497361 26.3469128 -48.204326
H 21.0706298 26.8855675 -49.103357
C 19.3411503 28.0678737 -49.4726293
H 18.5032263 28.2293829 -48.7937525
H 20.0551531 28.8885143 -49.395854
N 18.1444302 24.6585658 -52.7170806
H 18.7575415 24.0313991 -52.2081714
C 17.2497154 24.0944877 -53.7603078
H 17.1809009 24.8305569 -54.5644565
C 15.8108259 23.9536811 -53.2184155
O 15.6116559 23.7733122 -52.0168994
C 17.7905208 22.7718561 -54.367871
H 18.2164828 22.1394345 -53.5915213
H 16.9504542 22.2274091 -54.799801
C 18.8275157 22.9966047 -55.490398
H 19.7490875 23.3914824 -55.0618205
H 18.4292468 23.7618966 -56.1540239
C 19.1471149 21.7322105 -56.331149
H 18.3107319 21.0347568 -56.2604309
H 20.0375569 21.2460875 -55.9308658
N 19.3493069 22.0762304 -57.7577649
H 19.2235844 23.0639289 -58.0004847
C 19.3902273 21.3013719 -58.8329725
N 19.5688706 20.0105413 -58.789513
H 19.5868354 19.4947518 -59.6476506
H 19.6328429 19.5190873 -57.9186205
N 19.2411043 21.8219077 -60.01574
H 18.7989704 22.7503794 -60.07748
H 19.2658129 21.2691169 -60.8598066
N 14.821947 23.9635607 -54.1171727
H 15.0640462 24.1295175 -55.0795782

C 13.4070457 23.7053603 -53.8023649
H 13.2318405 23.8314889 -52.7303655
C 13.0148305 22.2690653 -54.1901151
O 13.2650034 21.8554559 -55.3268438
C 12.5042714 24.70102 -54.554355
H 11.4657565 24.4018961 -54.4176821
H 12.7363243 24.6603459 -55.6166903
C 12.5918951 26.14774 -54.1111584
O 13.1869008 26.5106661 -53.1113018
N 11.9857542 27.0281791 -54.8750885
H 12.0663734 27.990201 -54.6088372
H 11.474667 26.7304553 -55.7012412
N 12.3104354 21.5618859 -53.3039725
H 12.1319232 21.9882772 -52.3959269
C 11.7798987 20.2088839 -53.5218785
H 12.1354009 19.8236533 -54.4750295
C 10.2499869 20.2006078 -53.5304971
O 9.636071 20.6928764 -52.5837385
C 12.2939867 19.2814334 -52.4104324
H 12.0003572 19.7111831 -51.4550013
H 13.3811827 19.2399207 -52.459261
C 11.7331357 17.8531895 -52.451972
H 10.6473428 17.8812916 -52.5280063
H 11.9562163 17.3702826 -51.5054196
S 12.3827862 16.813984 -53.7779941
C 13.8931229 16.2105975 -52.9728887
H 14.4627188 15.602152 -53.6752431
H 13.6285852 15.6018071 -52.1075691
H 14.5036321 17.0516099 -52.6454799
N 9.6561252 19.476022 -54.4810838
H 10.2356146 19.1014098 -55.228018
C 8.2618632 19.0294284 -54.4110718
H 7.7432147 19.5950109 -53.6411374
C 8.2012683 17.5367186 -54.0391612
O 8.6091906 16.6916482 -54.8315145
C 7.5848191 19.3377223 -55.7563497
H 7.6533027 20.404727 -55.9472126
H 8.1407942 18.8235216 -56.5354205
C 6.1094319 18.9161937 -55.8471269
H 6.0223168 17.8621598 -55.5987058
C 5.2008171 19.7105303 -54.9111302
H 5.2842959 20.7773402 -55.1160026
H 5.4789999 19.5191252 -53.8783082
H 4.1706631 19.3882301 -55.0433692
C 5.5958432 19.1157678 -57.2735893
H 5.6369121 20.1715894 -57.5396105

H 4.5696247 18.7588489 -57.3505647
H 6.2159663 18.5490014 -57.9673902
N 7.650806 17.207511 -52.8705797
H 7.2809473 17.9542867 -52.2913677
C 7.3710433 15.8279812 -52.4464654
H 8.0060151 15.1508264 -53.0126345
C 5.9027388 15.4793458 -52.7611271
O 4.9709185 16.075372 -52.2065813
C 7.75986 15.6405981 -50.9667627
H 7.1203425 16.2573012 -50.3370099
H 8.7862314 15.9905422 -50.8370111
C 7.6824443 14.1863395 -50.4760692
O 6.8708591 13.4090086 -51.019936
O 8.4087115 13.8653462 -49.5055914
N 5.6864177 14.5711102 -53.7189983
H 6.4974395 14.0713565 -54.0777662
C 4.3447916 14.1842873 -54.1843736
H 3.6345714 14.9349283 -53.8406842
C 3.9351687 12.8544228 -53.5605881
O 4.3232699 11.7831128 -54.0192536
C 4.2336867 14.1575708 -55.7201635
H 4.8568936 13.3737071 -56.1278179
C 2.7974381 13.8629456 -56.1479142
H 2.1426664 14.6095088 -55.703318
H 2.4969621 12.8683791 -55.8186253
H 2.7229068 13.8922377 -57.2343704
C 4.6461988 15.4961607 -56.3443168
H 4.0765308 16.3124174 -55.897042
H 4.4772019 15.482627 -57.4198195
H 5.7094427 15.6583488 -56.1699027
N 3.0257182 12.9205146 -52.5909151
H 2.717312 13.8404901 -52.2952782
C 2.6511322 11.7941466 -51.7378099
H 1.5919382 11.8736081 -51.495824
H 2.826334 10.8515972 -52.2505211
C 3.4378837 11.764253 -50.4292475
O 3.7783718 10.6859284 -49.9559292
N 3.6725557 12.9262538 -49.8125931
H 3.3218807 13.7634739 -50.2589932
C 4.5639542 13.1282033 -48.6574106
H 5.5763021 12.8435635 -48.9560907
C 4.2394702 12.317265 -47.379758
O 4.9944892 12.3378076 -46.4012804
C 4.557135 14.6320349 -48.3562011
H 4.9178299 15.1702423 -49.2356233
H 5.2327209 14.8285762 -47.5267004

S 2.8894722 15.2074225 -47.9074698
H 2.7061254 14.4032963 -46.8479299
N 3.0995178 11.6227276 -47.3420058
H 2.5537956 11.6005599 -48.1895828
C 2.6973692 10.7695608 -46.2284903
H 1.7296098 10.3306215 -46.4382888
H 3.4252333 9.964224 -46.1301078
C 2.5964225 11.5335775 -44.9096616
O 2.0756425 12.6458024 -44.8617237
N 3.1475683 10.9405853 -43.8409104
H 3.5995258 10.050913 -43.9813798
C 3.2644505 11.5676352 -42.5110625
H 2.5363332 12.3721322 -42.4922009
C 4.6024855 12.3215117 -42.2989558
O 4.9330438 12.7035656 -41.1837733
C 2.7685976 10.6219858 -41.3876643
H 3.329892 9.6866489 -41.3966698
H 2.9110664 11.1133101 -40.4234859
C 1.2482459 10.3497499 -41.5881163
H 0.7919812 11.3134804 -41.7977461
H 1.1127473 9.7337692 -42.4784474
C 0.4227669 9.7161131 -40.4391496
H 0.7314226 10.1512193 -39.4901884
H 0.5960085 8.639396 -40.4025918
C -1.0758004 10.0217816 -40.7095582
H -1.41602 9.4220592 -41.5561837
H -1.1388727 11.0716698 -41.0127608
N -2.0107233 9.8625097 -39.5598994
H -2.0996365 8.9127489 -39.2334567
H -2.9406933 10.1949484 -39.8135329
H -1.7309402 10.4529809 -38.7839952
N 5.3030928 12.6456133 -43.3932679
H 4.9509657 12.3158047 -44.2839729
C 6.263606 13.7539353 -43.4685514
H 6.339384 14.0373157 -44.5168193
H 5.8664254 14.6025745 -42.9108326
C 7.6966603 13.5217271 -42.9844836
O 8.5229441 14.4189115 -43.1446022
N 8.052298 12.3272621 -42.5032093
H 7.3432049 11.6119669 -42.3818921
C 9.4128024 12.0200965 -42.040962
H 9.6646061 12.6654394 -41.1981862
H 9.4474178 10.9834761 -41.7123813
C 10.481707 12.1834795 -43.1192423
O 11.5222019 12.7773601 -42.8556136
N 10.1750316 11.7843984 -44.3589289

H 9.2846575 11.3332788 -44.4955374
C 11.0186339 12.0413754 -45.5425943
H 11.9980506 11.588156 -45.3790969
C 11.2701527 13.5389522 -45.7486745
O 12.4236188 13.9518113 -45.8260238
C 10.4258214 11.3832015 -46.8083101
H 10.5905208 10.3099959 -46.7269139
C 8.9312833 11.6033541 -47.0463739
H 8.7099762 12.6632397 -47.1442538
H 8.3393666 11.1827496 -46.2344072
H 8.6361123 11.1131843 -47.9758533
O 11.0895781 11.8306891 -47.9597574
H 10.484578 12.3844576 -48.4701324
N 10.2252136 14.371186 -45.663101
H 9.3074535 13.9722939 -45.5446904
C 10.2857366 15.8236971 -45.8547703
H 10.7235008 16.0265557 -46.8268567
C 11.1106349 16.537419 -44.7650356
O 11.8863183 17.4526382 -45.0535196
C 8.8657689 16.3970441 -45.8856242
H 8.8986116 17.4030429 -46.3069571
H 8.4867539 16.4648006 -44.8668808
S 7.7163401 15.3759861 -46.8510702
H 8.4533831 15.1086649 -47.9512639
N 10.9632892 16.0999518 -43.508704
H 10.2825801 15.3646959 -43.3383485
C 11.7666912 16.5933009 -42.3777083
H 11.7210304 17.682167 -42.3558897
C 13.2337723 16.2149077 -42.5646649
O 14.1041332 17.0794444 -42.4631432
C 11.2181531 16.0596538 -41.0385742
H 11.0788588 14.9803609 -41.1088303
C 12.1583111 16.3454275 -39.8608403
H 12.413162 17.4044263 -39.8365978
H 13.0713173 15.7583224 -39.9544092
H 11.6747709 16.0658247 -38.9240883
C 9.8619149 16.7101419 -40.7311641
H 9.9738266 17.7906836 -40.6408162
H 9.465485 16.313601 -39.7953368
H 9.147258 16.4904463 -41.5236473
N 13.5086787 14.9554922 -42.9189931
H 12.7374487 14.300883 -43.0121126
C 14.8616324 14.4600437 -43.1763215
H 15.4605874 14.5795315 -42.2714439
C 15.5717593 15.257738 -44.2626702
O 16.6984701 15.6980688 -44.0520232

C 14.8119003 12.9696725 -43.5454865
H 15.47017 12.778969 -44.3885111
H 13.8159203 12.6800994 -43.8734669
C 15.2350372 12.1081566 -42.3887418
N 16.5503927 11.9810507 -41.9354251
C 16.4753185 11.2196844 -40.8299497
H 17.3253826 10.9192111 -40.2306378
N 15.2029599 10.8627996 -40.5836198
H 14.890693 10.2906702 -39.8085624
C 14.4037633 11.4233475 -41.5538219
H 13.3257702 11.3738953 -41.6274226
N 14.9078435 15.5075468 -45.387301
H 13.989292 15.091668 -45.5152898
C 15.5112271 16.2350314 -46.5011762
H 16.4944499 15.8005517 -46.6810838
C 15.788866 17.702243 -46.1455768
O 16.9395239 18.1374501 -46.2012692
C 14.6897345 16.0179734 -47.7882799
H 15.1327128 16.6236197 -48.5603665
C 14.7908711 14.5234165 -48.182398
H 14.1962195 13.8987777 -47.5198436
H 15.8220997 14.1781548 -48.1262874
H 14.4372692 14.3716629 -49.2011021
C 13.2366266 16.4975189 -47.7320752
H 12.7646062 16.0195718 -46.8976035
H 13.2059957 17.5711979 -47.5585925
C 12.4407379 16.1837724 -49.0033466
H 12.2244151 15.1164961 -49.062049
H 13.0189562 16.48687 -49.8741816
H 11.4970485 16.7230964 -48.9888032
N 14.8187234 18.4245126 -45.5793613
H 13.9008259 18.0120409 -45.4473034
C 15.0314858 19.8172963 -45.1857533
H 15.4248899 20.3646793 -46.0422485
C 16.0654803 19.9815299 -44.052507
O 16.775709 20.9872676 -44.0197683
C 13.6739593 20.4215669 -44.8288207
H 13.2487701 19.8884107 -43.9775321
H 12.9997992 20.3346726 -45.682005
H 13.7950387 21.4777533 -44.588073
N 16.1945128 19.0031707 -43.1417661
H 15.5550477 18.2125874 -43.1960906
C 17.1426889 19.0652184 -42.0165262
H 17.2682905 20.115006 -41.7540704
C 18.5555244 18.5806105 -42.3633946
O 19.5022617 19.2022987 -41.8920977

C 16.5356863 18.3408313 -40.8014493
H 15.5546198 18.7709715 -40.5929376
H 16.3868088 17.2899072 -41.0560764
C 17.3783728 18.4041283 -39.5137768
H 18.2761985 17.8007418 -39.655826
H 16.803368 17.9420041 -38.7110996
C 17.8159365 19.8120997 -39.0658109
H 18.4907804 20.2290409 -39.816017
H 18.3932875 19.7217441 -38.1440225
N 16.6897875 20.7491832 -38.8609668
H 16.5665419 21.4502419 -39.5716769
C 15.8807065 20.8193544 -37.8169131
N 14.9853566 21.7589121 -37.7435133
H 14.3645694 21.8061651 -36.956886
H 14.8726153 22.4170928 -38.4946404
N 15.9472023 19.9843426 -36.8189475
H 16.6241288 19.2455779 -36.8502505
H 15.2949912 20.0477172 -36.0589826
N 18.7300829 17.5290883 -43.1764286
H 17.9068943 17.0488226 -43.5306661
C 20.0684893 17.0390531 -43.5746573
H 20.7065707 16.9291063 -42.6964889
C 20.7865705 18.0002679 -44.5366372
O 22.0134416 18.0053269 -44.5586972
C 19.9813346 15.699411 -44.3219547
H 19.304658 15.8747004 -45.1616404
H 20.9671976 15.5209637 -44.7569981
C 19.5628823 14.3578569 -43.6784448
H 20.3290906 14.0366348 -42.971993
H 18.6129878 14.4437749 -43.1562454
C 19.4543242 13.3380914 -44.844005
H 20.418487 13.3271226 -45.354375
H 18.7167883 13.694725 -45.5669937
C 19.1362141 11.8730891 -44.4980398
H 19.490443 11.6480878 -43.4892399
H 19.686472 11.2395421 -45.2001729
N 17.6955314 11.5486037 -44.6343123
H 17.1272324 12.0169392 -43.9401613
H 17.4806865 10.5531329 -44.5600195
H 17.3202713 11.7308229 -45.5705452
N 20.0478271 18.6624719 -45.4364741
H 19.046923 18.5093921 -45.436498
C 20.6358853 19.3309775 -46.6085273
H 21.6749389 19.5607893 -46.3684505
C 20.0541746 20.7091617 -46.9622581
O 20.3152965 21.2210728 -48.0515675

C 20.6868561 18.354292 -47.7957786
H 21.0473326 18.879063 -48.6814314
H 21.4566581 17.6177888 -47.5613333
C 19.4373287 17.602644 -48.1667651
C 19.394892 16.1940631 -48.1271936
H 20.196105 15.6434021 -47.6560693
C 18.3451897 15.5113149 -48.7728262
H 18.2923324 14.4372359 -48.7484014
C 17.3896775 16.2497781 -49.5044677
O 16.5031357 15.6745726 -50.3589183
H 16.8175994 14.8393012 -50.723428
C 17.3695605 17.6457992 -49.4114359
H 16.6398896 18.1806343 -49.9827419
C 18.3848271 18.32481 -48.7295873
H 18.4231516 19.3990316 -48.7319403
N 19.3517895 21.3539171 -46.024161
H 19.1651048 20.8775033 -45.1539639
C 19.0384741 22.7911583 -46.0750765
H 18.3539854 22.9771147 -45.2457682
C 18.2660689 23.2374827 -47.3416898
O 18.3498463 24.3844502 -47.7803859
C 20.3254224 23.5929801 -45.7682459
H 21.0190659 23.4837652 -46.6030665
H 20.8007875 23.1682607 -44.8823023
C 20.0831759 25.0814136 -45.500918
O 19.0655738 25.4065578 -44.8420731
O 20.8655128 25.9146396 -46.0124582
N 17.4548563 22.3556176 -47.9295338
H 17.4238769 21.424189 -47.5465261
C 16.4823397 22.7281121 -48.9710723
H 16.9464183 23.4477338 -49.6473765
C 15.24281 23.3977265 -48.3725054
O 14.9522023 23.2457856 -47.1836795
C 16.0422905 21.5162724 -49.8130752
H 15.2603537 21.8499514 -50.4920367
C 17.2011516 21.0558416 -50.7125561
H 18.1143336 20.9147098 -50.1404907
H 17.4026366 21.8357731 -51.4439333
H 16.943333 20.1436236 -51.2459316
C 15.398919 20.4182088 -48.9405732
H 16.1665696 19.8238888 -48.4571155
H 14.7955406 20.8598812 -48.151009
C 14.4493492 19.5350595 -49.7478417
H 14.8862927 19.2531794 -50.7036183
H 13.5268763 20.0795328 -49.9194258
H 14.2201392 18.6408149 -49.1834476

N 14.4360211 24.010578 -49.2375354
H 14.7402083 24.0446147 -50.2085445
C 12.9993631 24.158699 -49.0076405
H 12.8175786 24.2573771 -47.9397338
C 12.2662528 22.9046082 -49.5103378
O 12.6107608 22.3634176 -50.5604784
C 12.5234879 25.4517901 -49.6859483
H 13.0406288 26.283309 -49.2048236
H 12.830272 25.44303 -50.731898
C 11.0112581 25.7338392 -49.6079951
H 10.8575365 26.7986213 -49.7828324
H 10.658697 25.5126149 -48.6002629
C 10.1424015 24.9839405 -50.6247752
O 10.5934761 24.3664138 -51.5757024
N 8.8434225 24.9954737 -50.4307784
H 8.2650384 24.4828781 -51.0906918
H 8.4331033 25.5005896 -49.6701504
N 11.2359635 22.4621827 -48.7844512
H 11.0578115 22.9049529 -47.8891113
C 10.3429926 21.383246 -49.214284
H 10.5949631 21.0920705 -50.235015
C 8.8711568 21.8264889 -49.2272665
O 8.3637704 22.3460571 -48.2286926
C 10.5732244 20.1618264 -48.309774
H 10.3332883 20.4190762 -47.2777976
H 11.6227969 19.8672222 -48.3609254
S 9.5400098 18.7650291 -48.8429061
H 8.3402798 19.3259352 -48.6493615
N 8.1565084 21.4877271 -50.299685
H 8.6580875 21.1050509 -51.0997332
C 6.6915622 21.4723115 -50.3796701
H 6.2622286 21.8728319 -49.4665579
C 6.2201094 20.031677 -50.5303368
O 6.4399128 19.4158451 -51.5664035
C 6.1943466 22.3420754 -51.5434893
H 6.7074003 22.060723 -52.4640156
C 4.6826819 22.2560971 -51.7615391
H 4.1511206 22.4652032 -50.8329504
H 4.408085 21.2625488 -52.117419
H 4.3832472 22.9787312 -52.5207642
O 6.4597851 23.693421 -51.2481149
H 6.0035097 23.908674 -50.423727
N 5.5750189 19.4858299 -49.5003679
H 5.4489048 20.0421054 -48.6609263
C 4.8934822 18.1915638 -49.5707935
H 5.3805521 17.5583986 -50.3075649

H 4.9561912 17.6920084 -48.6044006
C 3.4178491 18.3381219 -49.9451647
O 2.7499778 19.2457229 -49.4508098
N 2.8759735 17.4192718 -50.7447628
H 3.489359 16.728848 -51.172119
C 1.4218424 17.2768095 -50.9304672
H 0.9352328 17.8212694 -50.1261785
C 0.9560668 15.8305307 -50.7697592
O 1.6406314 14.8966804 -51.1867693
C 0.9136146 17.8905378 -52.2565359
H -0.1448096 17.6360649 -52.3435319
C 1.6387051 17.3248981 -53.5004349
H 2.6843131 17.6363338 -53.4916327
H 1.6106378 16.2363657 -53.4623999
C 0.9961788 19.4249524 -52.1858548
H 2.0365676 19.7521869 -52.1926341
H 0.5247676 19.7783184 -51.266841
H 0.4642133 19.8755679 -53.0218273
C 1.0052803 17.7519525 -54.8316522
H 1.1088672 18.8260369 -54.9781292
H -0.0515642 17.481976 -54.8436029
H 1.510511 17.2397663 -55.65089
N -0.2477491 15.6495421 -50.2260329
H -0.7465903 16.4668413 -49.8863459
C -0.9656272 14.3704334 -50.212057
H -0.6470092 13.784003 -51.0743246
C -2.4736961 14.6046495 -50.3361702
O -2.9923013 15.6108318 -49.855645
C -0.6345655 13.5626088 -48.9469876
H -1.035592 14.063649 -48.0667914
H 0.4483293 13.4718768 -48.8486547
O -1.2039403 12.2647033 -49.0661658
H -0.8230701 11.6633302 -48.3915009
N -3.189996 13.6624306 -50.9545782
H -2.693636 12.8593069 -51.3085801
C -4.6570956 13.7038033 -51.1109514
H -4.9453094 14.6681594 -51.5324737
C -5.3659256 13.5807981 -49.7505522
O -6.4750307 14.0756237 -49.5707605
C -5.0838402 12.5810381 -52.0946599
H -4.5868338 11.656628 -51.7934535
C -6.60297 12.3174183 -52.0927149
H -7.1427278 13.2367823 -52.3255887
H -6.9278242 11.951214 -51.1188279
H -6.8636599 11.5560362 -52.8261546
C -4.6265523 12.9470809 -53.5247128

H -5.2699191 13.744181 -53.8956398
H -3.6030739 13.3215718 -53.5000957
C -4.6555746 11.788384 -54.5300713
H -5.6777838 11.458972 -54.7113472
H -4.0625365 10.9539342 -54.1543668
H -4.2321241 12.1260136 -55.4769672
N -4.7271601 12.913471 -48.788543
H -3.775143 12.6272275 -48.9723444
C -5.2999645 12.5558781 -47.4919011
H -6.3581933 12.3426238 -47.6238231
C -5.12327 13.6827605 -46.4548777
O -3.9915082 13.9538372 -46.0403971
C -4.6452439 11.2446981 -47.0565748
H -3.587913 11.2809028 -47.3174804
H -5.1004324 10.4159779 -47.6001787
O -4.7366918 11.0128571 -45.6723862
H -5.6758702 11.0025864 -45.3613888
N -6.2181431 14.2734505 -45.9265974
C -6.1448261 15.2535177 -44.8392723
H -5.5037262 16.0768951 -45.1469994
C -5.5994107 14.6593758 -43.5365247
O -4.990551 15.3639187 -42.7414758
C -7.5767455 15.7683423 -44.6357585
H -8.056633 15.2366621 -43.8112928
H -7.5919532 16.8425494 -44.4489233
C -7.6005661 14.1249832 -46.3585443
H -7.6892693 14.0050037 -47.4364082
H -8.051605 13.2737373 -45.845865
C -8.2932888 15.4153499 -45.9362787
H -8.111043 16.1900229 -46.680453
H -9.3633916 15.2698172 -45.7853592
N -5.798928 13.3603836 -43.3244477
H -6.3334307 12.8472974 -44.0199305
C -5.3819279 12.5909009 -42.1533687
H -5.5086175 13.2194111 -41.2710266
C -3.889537 12.1953296 -42.1819728
O -3.2267231 12.2061379 -41.1417984
C -6.3412377 11.3905832 -41.9922851
H -7.3113502 11.7703061 -41.6675729
H -5.9575394 10.7498656 -41.1971473
C -6.5663179 10.5388814 -43.2538189
O -6.908747 11.078591 -44.3329274
O -6.3508923 9.3107568 -43.1812859
N -3.3008267 11.9683216 -43.3619274
H -3.8866016 11.9470974 -44.1933319
C -1.8430178 11.9831623 -43.5618261

H -1.3723892 11.257173 -42.9150736
C -1.2618897 13.3502526 -43.1982805
O -0.4067809 13.4546671 -42.3135894
C -1.4776396 11.6187214 -45.0103698
H -2.1218696 12.1563712 -45.7031098
H -0.4492902 11.9202012 -45.2048059
C -1.5826308 10.1109484 -45.2530965
H -2.542401 9.7508069 -44.8866851
H -0.8029225 9.6052696 -44.6769365
C -1.4265517 9.7523301 -46.7342513
O -2.1887154 8.9014817 -47.2378949
O -0.4387212 10.1870761 -47.3704002
N -1.8238071 14.3981459 -43.7998905
H -2.5652821 14.2183388 -44.4716944
C -1.3629664 15.7743681 -43.644951
H -0.2966211 15.7971953 -43.8619811
C -1.5011144 16.2671664 -42.1931932
O -0.5669668 16.8774303 -41.6842395
C -2.0937697 16.6432986 -44.6956286
H -3.1616424 16.4331521 -44.6208048
C -1.6453572 16.3062192 -46.1415492
H -1.8060816 15.2459139 -46.3228022
H -2.2857106 16.8442621 -46.8391984
C -1.9169274 18.1412204 -44.4519846
H -0.8624135 18.3648704 -44.4776171
H -2.351247 18.4235725 -43.4927015
H -2.4173335 18.7036014 -45.2397571
C -0.1850993 16.6247081 -46.5047295
H 0.502714 16.0852862 -45.8550906
H 0.005957 17.6948585 -46.4390428
H -0.0012286 16.3148047 -47.5317343
N -2.5671139 15.9256739 -41.4689525
H -3.3267024 15.4447242 -41.9411177
C -2.7735512 16.3191514 -40.0675664
H -2.7786111 17.4067291 -40.0129945
C -1.6436206 15.8404344 -39.1348382
O -1.1884872 16.6049745 -38.2795479
C -4.1712491 15.8244989 -39.6523631
H -4.8964345 16.3669418 -40.2600162
H -4.276831 14.7663523 -39.8970248
C -4.5547229 16.0502673 -38.1793378
H -5.6347657 16.1922723 -38.1337433
H -4.0852274 16.9595111 -37.8033673
C -4.2106249 14.87867 -37.2602295
O -3.478791 13.9624486 -37.5900405
N -4.7524033 14.838826 -36.0651956

H -4.5608134 14.0108061 -35.5281391
H -5.3835633 15.5549238 -35.7602172
N -1.1004638 14.6424705 -39.3708469
H -1.5013084 14.0764096 -40.106404
C 0.0418472 14.1322373 -38.6116889
H -0.0436926 14.4501244 -37.5713118
C 1.3850099 14.6518156 -39.1239408
O 2.2660308 14.8908551 -38.3035319
C 0.0435491 12.6104591 -38.6527647
H 1.0465421 12.2488474 -38.4138007
H -0.2262749 12.3017169 -39.6598596
S -1.1117245 11.9445062 -37.4241918
H -2.0986728 12.845829 -37.6105998
N 1.5575739 14.8170369 -40.4417058
H 0.8054631 14.5690666 -41.0762493
C 2.7701594 15.422793 -40.987119
H 3.6444812 14.9045317 -40.5883586
C 2.8696392 16.8863013 -40.5636503
O 3.5847836 17.2487937 -39.6283336
C 2.7907183 15.3258836 -42.5238653
H 1.9156234 15.7985274 -42.9702768
H 2.7822393 14.2994419 -42.8465516
H 3.7036186 15.7788831 -42.9060338
N 2.1002966 17.7180647 -41.2791163
H 1.1970245 17.3057366 -41.4945638
C 2.6117307 18.5517644 -42.3861192
H 3.1341734 17.8723516 -43.0516016
C 1.3970127 19.0600589 -43.2242848
H 1.358215 18.4262558 -44.0974844
H 0.5018309 18.6692998 -42.7733033
C 1.1166488 20.5727843 -43.5444475
H 1.2915895 21.151315 -42.6635274
H 1.8411256 20.9728381 -44.2381746
C -0.3230066 20.9360951 -43.9914732
H -1.0227159 20.3720331 -43.3859128
H -0.4699022 20.6178342 -45.0179328
C -0.6963571 22.4298742 -43.8025919
H 0.1995555 22.9755746 -43.4898631
H -1.4260614 22.4979107 -42.9928553
N -1.2635441 23.0865246 -45.012343
H -0.5895041 23.0906335 -45.7804691
H -1.4992876 24.0597524 -44.8902245
H -2.0834365 22.6400856 -45.4285437
C 3.7642777 19.4606364 -41.9383207
O 4.6934606 18.9814363 -41.3095185
N 3.6311882 20.7771193 -41.9989438

H 2.8876535 21.1417414 -42.5604266
C 4.0788258 21.6000937 -40.8865894
H 5.1237588 21.3834285 -40.6673247
C 3.9999911 23.0820359 -41.3327063
H 3.0493725 23.2473995 -41.8409777
H 4.7944901 23.2717171 -42.0553756
C 4.1026739 24.1179475 -40.2399803
N 3.0918861 25.0331692 -39.9409579
C 3.4223691 25.5589047 -38.7527186
H 2.8010558 26.2448663 -38.192991
N 4.5752577 25.0358408 -38.3042514
H 4.8930887 25.1113105 -37.3429746
C 5.0364284 24.1390869 -39.2428601
H 5.8685618 23.4582498 -39.1352582
C 3.2420288 21.199852 -39.6385361
O 3.0797663 22.025223 -38.7500341
N 2.530232 20.0383135 -39.640792
H 2.865367 19.2416506 -40.1623869
C 1.4823966 19.8353562 -38.7041374
H 1.2481924 20.7922532 -38.2384218
C 2.0858318 19.0933861 -37.5361898
O 3.067777 19.6165891 -37.0050026
C 0.1413169 19.4928001 -39.3442083
H 0.1442248 18.4588372 -39.6817178
H -0.6141341 19.569777 -38.5611991
C -0.267998 20.4123699 -40.5175301
H 0.1183068 19.9509151 -41.3678312
C -1.7776922 20.4353344 -40.70414
H -2.2492346 20.8376265 -39.8066394
H -2.1313518 19.4171078 -40.8620015
H -2.0806715 21.041301 -41.5528518
C 0.1944014 21.8747087 -40.5369619
H 1.2344464 22.003532 -40.7787416
H 0.035205 22.3050513 -39.5470494
H -0.3843236 22.4742456 -41.2283432
N 1.4878269 18.020571 -37.0275607
H 0.680745 17.619781 -37.490007
C 1.8370793 17.567107 -35.682473
H 1.6480608 18.3923588 -34.994206
C 3.3255548 17.1909504 -35.5105984
O 3.975249 17.6990186 -34.5966676
C 0.897452 16.4265003 -35.2938252
H 1.0844826 15.5580817 -35.9233497
H -0.1416476 16.7397962 -35.4098183
H 1.0723946 16.150676 -34.2533891
N 3.895938 16.3935091 -36.4204664

H 3.3273487 16.0340792 -37.1822979
C 5.2891756 15.9425756 -36.3358269
H 5.4806631 15.6036171 -35.3165186
C 6.3276383 17.0397494 -36.6405866
O 7.4910534 16.9063308 -36.2670223
C 5.4618095 14.744717 -37.274927
H 5.2733635 15.0489023 -38.3063018
H 4.7617187 13.9547081 -37.0015401
H 6.47971 14.3605827 -37.1961688
N 5.9285687 18.1328717 -37.2982429
H 4.962632 18.1795496 -37.6016175
C 6.8314934 19.2418052 -37.635999
H 7.8429605 18.8505207 -37.6832907
C 6.844892 20.3829303 -36.6121256
O 7.5699517 21.3645417 -36.8076774
C 6.4883151 19.7654549 -39.0249614
H 5.4763035 20.1549587 -38.9918722
H 6.5452826 18.940506 -39.7349417
O 7.3909096 20.7802827 -39.4271918
H 7.5897174 21.3108912 -38.6363837
N 6.0301942 20.3258281 -35.5583977
H 5.4633655 19.4990513 -35.4131305
C 5.9102691 21.4436743 -34.6242259
H 5.8569889 22.3420848 -35.2375565
C 7.1639527 21.593333 -33.7492034
O 7.6308825 20.6298769 -33.149975
C 4.6035516 21.3149437 -33.8160339
H 3.9610644 20.5586151 -34.2705869
H 4.8261419 20.983383 -32.8007128
C 3.8146552 22.6339158 -33.7630863
H 4.3761121 23.361526 -33.1761685
H 2.8597389 22.454182 -33.2696363
C 3.5503843 23.2164719 -35.1512038
O 4.0029425 24.3035214 -35.4804174
N 2.9488639 22.4629638 -36.0411139
H 2.950617 22.7735293 -37.0102779
H 2.7918841 21.4817724 -35.8719266
N 7.7210904 22.8081187 -33.6914249
H 7.2987774 23.5567177 -34.2190106
C 8.9972279 23.0788989 -33.0131334
H 9.0701395 24.1482844 -32.8145984
H 9.0214978 22.5526099 -32.0586359
C 10.2493908 22.6746477 -33.805537
O 11.3286693 22.5814544 -33.2268775
N 10.1271567 22.3925806 -35.1093067
H 9.2142375 22.4390954 -35.5407744

C 11.2828804 22.1739636 -35.9765919
H 12.1470555 21.9182832 -35.3639204
C 11.6311631 23.4425166 -36.7703543
O 10.8468171 23.8739616 -37.6062429
C 11.0248787 21.0051895 -36.9419587
H 10.2282802 21.2982624 -37.627223
H 11.9359448 20.8776523 -37.5163873
C 10.6521745 19.6372191 -36.3484215
H 9.6886744 19.7031776 -35.8480046
C 10.554009 18.6256642 -37.4922608
H 11.5421073 18.4392663 -37.908598
H 9.9033941 19.0025286 -38.2786216
H 10.1402736 17.6890333 -37.1141196
C 11.700169 19.1190278 -35.363519
H 12.6761902 19.0615675 -35.8409176
H 11.4049478 18.1266817 -35.0192001
H 11.7434391 19.7788958 -34.4960605
N 12.8948299 23.8661966 -36.7091582
H 13.3352564 23.7065076 -35.812184
C 13.4155394 25.0179326 -37.4790076
H 12.6681205 25.8145446 -37.4282954
C 13.6732908 24.7387133 -38.9836209
O 14.3679382 25.4938641 -39.6582383
C 14.7076514 25.5493859 -36.8171534
H 15.5330047 24.8870948 -37.0856383
H 14.9254605 26.5347873 -37.2302733
C 14.686988 25.6625318 -35.2845744
H 15.5866248 26.1902592 -34.9596257
H 14.7320807 24.6561241 -34.8586402
C 13.4378158 26.3886491 -34.7758366
O 13.4280188 27.6368107 -34.819975
O 12.4900572 25.6633847 -34.4019596
N 13.2648524 23.5772005 -39.5105643
H 12.5354699 23.085907 -39.0068225
C 13.5570177 23.1795007 -40.8976494
H 14.5254876 23.6009869 -41.1761233
C 12.542468 23.7627457 -41.8860466
O 11.3445727 23.7642826 -41.6133993
C 13.6717175 21.6505914 -41.0511484
H 14.6737933 21.3409764 -40.7633607
H 13.5409867 21.3814689 -42.0977122
C 12.6807254 20.8409029 -40.234541
O 13.0819143 19.9571612 -39.4998137
N 11.4063889 21.1652132 -40.2593715
H 10.7663929 20.6171536 -39.7086994
H 11.0835354 21.9909526 -40.7547637

N 13.0133136 24.1123106 -43.0888082
H 14.003369 23.9861169 -43.252692
C 12.2559559 24.8063065 -44.14478
H 11.634147 25.5703974 -43.6725806
C 11.2900693 23.8636724 -44.8836361
O 11.4794884 23.5296958 -46.0528063
C 13.2402964 25.5207442 -45.0935189
H 13.7974318 24.7585126 -45.6299273
H 12.6747639 26.1075278 -45.8195301
C 14.2367 26.4457212 -44.363787
H 13.6782029 27.2076148 -43.8182901
H 14.8081668 25.8651489 -43.6389009
C 15.2530414 27.1445263 -45.2802328
H 15.9662655 27.6665972 -44.6388995
H 14.7410762 27.8844194 -45.8974409
C 16.0017683 26.1590706 -46.1895801
H 15.4383225 26.0365783 -47.1185131
H 16.0485386 25.1791219 -45.7028254
N 17.3833513 26.6013149 -46.4784672
H 17.4501689 27.5257558 -46.8646949
H 17.8394313 25.9286781 -47.0934372
H 17.9672793 26.5200239 -45.6378488
N 10.2586788 23.4106932 -44.1767312
H 10.1841574 23.7178249 -43.2124954
C 9.2046284 22.5279462 -44.6902692
H 9.4646982 22.2318761 -45.7066837
C 7.8664666 23.2561846 -44.773848
O 7.5173305 24.0732073 -43.9268036
C 9.0728533 21.2239852 -43.8778855
H 8.316634 20.6091786 -44.3642886
C 10.3766276 20.4230278 -43.8853184
H 11.1563743 20.9672999 -43.3545985
H 10.6901088 20.2389319 -44.9118237
H 10.2204614 19.4614051 -43.3955968
C 8.6444678 21.4437578 -42.4225151
H 9.3296493 22.1190396 -41.9159669
H 8.6253233 20.491972 -41.8909169
H 7.6444986 21.8751438 -42.3818422
N 7.0848025 22.9020958 -45.7835561
H 7.4542566 22.2468272 -46.4660422
C 5.688535 23.2901281 -45.9603944
H 5.2592379 23.5642908 -44.9971347
C 4.9368154 22.090787 -46.5162855
O 5.5175932 21.2799915 -47.2402207
C 5.572196 24.4861637 -46.9093264
H 6.1315988 25.3254024 -46.4927753

H 4.5253632 24.7775338 -47.0057321
O 6.0920421 24.166681 -48.1936715
H 6.9019288 23.6412406 -48.069159
N 3.6649475 21.9328174 -46.1572492
H 3.1924113 22.6509743 -45.6290061
C 2.8540294 20.891737 -46.7718369
H 3.2603949 20.7420939 -47.768523
C 1.4232672 21.3469321 -46.9993605
O 0.9655742 22.2461308 -46.2958749
C 2.8723023 19.5311039 -46.0727033
H 1.8998189 19.3593649 -45.6756641
H 2.831625 18.8431272 -46.9167144
C 4.0238134 18.9905664 -45.2169582
C 4.3071869 17.6253821 -45.3860567
H 3.6888653 17.0224533 -46.0309537
C 4.8919789 19.744078 -44.3979068
H 4.7530479 20.7996534 -44.2590226
C 5.3992881 17.0235059 -44.752768
H 5.5957283 15.9801922 -44.9285487
C 5.9619852 19.1264943 -43.7177794
H 6.5828412 19.700868 -43.0462872
C 6.2154413 17.761582 -43.8888187
H 7.0295482 17.2877361 -43.3583586
N 0.7049211 20.6843602 -47.9071269
H 1.1580329 19.9917536 -48.4999263
C -0.7426341 20.8387991 -48.0115702
H -1.0824204 20.9641876 -46.9898499
C -1.5361428 19.617803 -48.4852348
O -0.9924186 18.6595985 -49.0384563
C -1.11455 22.1121096 -48.8003637
H -1.7026334 21.8618073 -49.6844732
H -0.2187602 22.6483505 -49.119513
C -1.9431406 22.9948283 -47.8556361
O -2.8487189 22.4398679 -47.1824651
O -1.4945848 24.1167787 -47.540539
N -2.8489941 19.6723543 -48.2388324
H -3.2118883 20.5386921 -47.8451067
C -3.8093695 18.7281656 -48.8164363
H -3.3973647 17.7249245 -48.7310293
C -3.9734597 19.0752603 -50.2936677
O -4.3973474 20.175993 -50.6415666
C -5.1732061 18.7435119 -48.0992768
H -5.6730347 19.6950951 -48.2825975
C -6.0637773 17.6068255 -48.6193746
H -5.6052813 16.639463 -48.4108445
H -6.2151491 17.7031978 -49.6940242

H -7.0395154 17.6576898 -48.1394967
C -5.0359728 18.5600012 -46.5826229
H -4.4763312 17.6507279 -46.373113
H -6.0235974 18.4938287 -46.1264681
H -4.5200769 19.4177832 -46.1531065
N -3.6215795 18.1436875 -51.1741785
H -3.2879382 17.2507786 -50.8282856
C -3.6115106 18.3903012 -52.6077545
H -4.5005105 18.9612831 -52.879691
H -2.7342712 18.9845956 -52.864576
C -3.5764643 17.1062873 -53.4192378
O -2.760003 16.2192524 -53.1788795
N -4.4347018 17.0516582 -54.4326559
H -5.0732818 17.8204822 -54.5581563
C -4.3864658 16.05468 -55.4956002
H -4.0915978 15.0912157 -55.0883004
C -3.3390088 16.4662952 -56.5310674
O -3.4495113 17.5140222 -57.164842
C -5.7890218 15.9175663 -56.0942179
H -6.1264015 16.9003061 -56.4288134
H -6.4684305 15.5787826 -55.3090581
C -5.884318 14.9468145 -57.2730016
O -4.8383242 14.4918336 -57.7921059
O -7.0322555 14.7422459 -57.7062923
N -2.3237965 15.6294866 -56.7222922
H -2.3348741 14.7515165 -56.2290766
C -1.1959614 15.917138 -57.5968154
H -0.8042434 16.9026035 -57.3349828
C -1.5477935 15.9520276 -59.0940706
O -0.7986989 16.5414193 -59.8719076
C -0.1261968 14.8713849 -57.2991538
H -0.4332321 13.8964363 -57.6832249
H 0.0211865 14.8034629 -56.2217764
H 0.8044678 15.171372 -57.7763393
N -2.6894224 15.3899282 -59.5099429
H -3.3057519 14.9434825 -58.8268714
C -3.1680469 15.5081525 -60.8889374
H -2.2977069 15.4764396 -61.5426236
C -3.8714604 16.8578481 -61.1353749
O -3.8881519 17.3427345 -62.2717246
C -4.0589673 14.2899356 -61.2019821
H -3.5800509 13.3944349 -60.8022451
H -5.0094949 14.4145144 -60.6792547
C -4.3336983 14.054691 -62.7013104
H -4.8066073 14.9365606 -63.1323418
C -3.0607759 13.732287 -63.4923323

H -2.5395826 12.8897156 -63.037984
H -2.3987076 14.5952794 -63.5160859
H -3.3198034 13.4669686 -64.5171777
C -5.2831101 12.8667808 -62.868187
H -4.8269732 11.9632398 -62.4652122
H -5.5106869 12.7195113 -63.9246257
H -6.2135557 13.0676634 -62.3361046
N -4.3438173 17.5317856 -60.0767708
H -4.239743 17.0913676 -59.1689351
C -5.1392953 18.7670931 -60.1508346
H -5.3101043 19.014173 -61.1954728
C -4.4652452 20.0192241 -59.553186
O -4.7247452 21.1197762 -60.036646
C -6.5275032 18.4864972 -59.5562995
H -7.1041324 19.4099779 -59.5149897
H -6.4332717 18.0886791 -58.546576
C -7.2894454 17.5033969 -60.4354308
O -7.5563357 17.7986585 -61.5883171
N -7.5704357 16.2932746 -60.0008698
H -8.0536807 15.6856149 -60.632247
H -7.3300548 15.9419432 -59.0695682
N -3.5291447 19.8666245 -58.6129897
H -3.3778296 18.9328279 -58.2480178
C -2.7161973 20.948246 -58.0426512
H -3.3974747 21.6988151 -57.6398965
C -1.8381347 21.6244684 -59.108357
O -1.3603305 20.9679296 -60.0376461
C -1.8708616 20.3801509 -56.8857777
H -1.2155043 19.5960797 -57.2688558
H -2.5403692 19.9339514 -56.1488536
C -1.0113161 21.4298223 -56.1713507
H -0.2443703 21.7912918 -56.8567282
H -0.5059511 20.9491715 -55.3327881
S -1.9402459 22.8506813 -55.5394919
C -0.5701828 23.8292679 -54.8690719
H -0.9613234 24.74235 -54.4197375
H 0.1193026 24.0926016 -55.6715556
H -0.0410525 23.252321 -54.1098706
N -1.6015408 22.9369568 -58.9811233
H -1.9423219 23.4054305 -58.1486339
C -0.8192637 23.7390643 -59.9395053
H -0.2576772 23.064157 -60.5851844
C 0.2282464 24.5957342 -59.2270807
O 0.004776 25.0706294 -58.1183271
C -1.7509353 24.6002576 -60.822006
H -2.2485006 25.3361505 -60.1875853

H -1.1470927 25.1432575 -61.5512182
C -2.8355023 23.8041089 -61.576147
H -3.4692309 24.5095959 -62.1148217
H -3.4670243 23.293783 -60.85025
C -2.285634 22.7794283 -62.5838831
H -1.469829 22.2166229 -62.1362081
H -1.8727529 23.3160784 -63.440655
N -3.3480238 21.8600974 -63.0454371
H -4.1592275 22.2924263 -63.4494333
C -3.3712553 20.539867 -62.9367282
N -4.3632524 19.8552986 -63.436589
H -4.394121 18.8635073 -63.2050823
H -5.2093213 20.3214733 -63.7056537
N -2.4470361 19.8412767 -62.3456859
H -1.8072108 20.3142319 -61.7116381
H -2.5589807 18.8412342 -62.2636225
N 1.3477479 24.8147152 -59.9086339
H 1.3980397 24.4714973 -60.8561952
C 2.4801481 25.6382775 -59.4817666
H 2.171737 26.310206 -58.6796175
C 2.9540436 26.4820188 -60.6742369
O 2.5493109 26.2124622 -61.8048482
C 3.6101897 24.7291168 -58.9715134
H 3.9556652 24.0994097 -59.7921348
H 4.4519802 25.3525014 -58.6710504
C 3.2414508 23.8502424 -57.790578
C 3.2255769 24.3988016 -56.4940977
H 3.4578802 25.4439975 -56.3432923
C 2.944317 22.484486 -57.9805326
H 2.957988 22.0603603 -58.9749413
C 2.9118273 23.5871558 -55.3886793
H 2.893409 23.9989979 -54.390721
C 2.6376701 21.6672874 -56.8746023
H 2.4174114 20.6212776 -57.0198648
C 2.6196309 22.2218223 -55.5761608
O 2.3179465 21.4526177 -54.4997302
H 2.18435 20.5319836 -54.724826
N 3.8334992 27.4628261 -60.4509498
H 4.2082822 27.584594 -59.5219213
C 4.4939213 28.1620494 -61.5616058
H 3.7309422 28.5488971 -62.2384471
C 5.3882736 27.2020676 -62.3485732
O 6.0176283 26.3114741 -61.7616727
C 5.3217809 29.3477141 -61.0436929
H 6.091829 28.9980095 -60.3569351
H 4.6612574 30.029898 -60.5071673

O 5.9393037 30.0538829 -62.1032955
H 6.7243398 29.5613144 -62.4093461
N 5.5820288 27.4940815 -63.6317522
H 5.0106585 28.1958685 -64.0746187
C 6.7307802 26.9874338 -64.3760478
H 6.6366855 25.9085016 -64.4254029
C 8.028407 27.3558119 -63.6304401
O 8.1187891 28.4400932 -63.0417593
C 6.7479946 27.5450176 -65.8100117
H 7.719706 27.3149662 -66.2505881
H 6.6485008 28.6315395 -65.7731873
C 5.6672195 26.9697377 -66.7358325
O 4.772257 26.2412801 -66.2508656
O 5.7522914 27.2543315 -67.9504335
N 9.0046514 26.4438149 -63.5827792
H 8.8767 25.5757138 -64.0999271
C 10.2928452 26.6834173 -62.9168859
H 10.9628395 25.863104 -63.1596956
H 10.726691 27.5994604 -63.3176861
C 10.2581107 26.8168125 -61.3836782
O 11.1929088 27.3706979 -60.812188
N 9.2068629 26.3463782 -60.7000323
H 8.4713915 25.8971 -61.2280263
C 9.0731089 26.4374344 -59.2283582
H 9.1851045 27.4791121 -58.9258279
C 10.1069491 25.6136815 -58.4432941
O 10.4836519 25.994552 -57.3288833
C 7.6897912 25.9495096 -58.7796666
H 7.6527518 25.9341602 -57.6888603
H 7.5223822 24.9374057 -59.148062
O 6.6580577 26.7898418 -59.2502181
H 6.5249451 26.5892104 -60.1998395
N 10.5092653 24.4553591 -58.9775317
H 10.148396 24.2047745 -59.8925566
C 11.2703098 23.4359545 -58.2511685
H 11.53296 23.831252 -57.2725145
C 12.5826537 23.0539197 -58.9343996
O 12.7237566 23.0892687 -60.153064
C 10.4016771 22.1945978 -58.0069762
H 10.1678104 21.7295215 -58.9665107
H 10.9725225 21.4720044 -57.4234963
C 9.1174901 22.4918067 -57.2616053
C 9.1440574 22.7882535 -55.8861933
H 10.0770687 22.7663086 -55.3446341
C 7.8965917 22.5106125 -57.9559699
H 7.8822044 22.2996273 -59.0114969

C 7.9532069 23.1130515 -55.2127899
H 7.9771022 23.3415634 -54.1554797
C 6.7052395 22.8277978 -57.284616
H 5.7704859 22.841606 -57.8236995
C 6.7337942 23.135257 -55.9133376
H 5.8189192 23.3828901 -55.3936062
N 13.517583 22.5839715 -58.1188466
H 13.3072881 22.5350507 -57.1278463
C 14.8179288 22.0601079 -58.5444263
H 15.0314648 22.3351386 -59.5799209
C 14.8820061 20.5283609 -58.4571595
O 15.7122647 19.8905221 -59.1081171
C 15.861854 22.6970743 -57.6264017
H 15.4958654 22.6849266 -56.6000881
H 16.7664742 22.1004187 -57.6568063
C 16.1785143 24.1454572 -58.0345006
H 16.4935678 24.6931106 -57.143149
H 15.2907343 24.6425045 -58.4312527
C 17.305602 24.1476694 -59.067901
O 17.1190339 23.5958259 -60.1692975
O 18.44763 24.4781666 -58.6847995
N 14.0008482 19.9431131 -57.6415347
H 13.375073 20.5442361 -57.11928
C 13.8557196 18.5135042 -57.3961515
H 14.3000178 17.9505765 -58.2132188
C 12.3562485 18.1809218 -57.2916426
O 11.6055412 18.9392478 -56.6695756
C 14.5613807 18.1437631 -56.0764476
H 13.9703084 18.5856413 -55.2756477
H 14.5331341 17.0604963 -55.9537592
C 16.0075358 18.6382877 -55.8749913
H 16.0774433 19.7022195 -56.0961422
C 16.4272853 18.4705187 -54.4170553
H 16.4803628 17.4129 -54.1558347
H 15.7119752 18.972793 -53.7650525
H 17.3987076 18.9361346 -54.2814031
C 17.0207935 17.8886108 -56.7338335
H 16.9321508 16.814618 -56.5697651
H 18.0321975 18.2073195 -56.4892937
H 16.8337156 18.1120355 -57.7796173
N 11.9268476 17.0254245 -57.802589
H 12.5667476 16.4679935 -58.3580903
C 10.6134922 16.4408486 -57.4727742
H 10.200647 16.9817986 -56.6240596
C 10.8460628 14.9974708 -57.0338666
O 11.4968357 14.2196056 -57.7343886

C 9.584129 16.5453119 -58.623281
H 9.8891 15.8453796 -59.384544
C 9.5544193 17.9798466 -59.2141413
H 9.2019235 18.6905423 -58.4697116
H 10.5636784 18.2729503 -59.500348
C 8.1844474 16.0604285 -58.2049168
H 7.7911132 16.6907992 -57.4099126
H 8.2346799 15.0332518 -57.8434264
H 7.5072373 16.083636 -59.0554359
C 8.7221937 18.1222675 -60.4791129
H 7.6661354 18.1114382 -60.2270789
H 8.9737782 17.3097286 -61.1536164
H 8.9770545 19.0592215 -60.9698104
N 10.3564225 14.6574374 -55.8469035
H 9.7941865 15.3397182 -55.3446681
C 10.5610505 13.361721 -55.2088406
H 11.1527113 12.721799 -55.8623133
C 9.2265463 12.6740554 -54.9607565
O 8.2716464 13.3041168 -54.5053329
C 11.33476 13.5316808 -53.9000093
H 12.3374437 13.8914798 -54.1303004
H 10.8329836 14.2807638 -53.2847405
C 11.4560679 12.2414929 -53.107116
C 12.2584577 11.1893237 -53.5913009
H 12.8093437 11.3079028 -54.5115507
C 12.3285074 9.9712448 -52.892086
H 12.935959 9.1595666 -53.2719667
C 11.6002187 9.80203 -51.7022888
H 11.6597189 8.8679635 -51.1608939
C 10.7885537 10.8448729 -51.2227153
H 10.2173261 10.7141468 -50.3149552
C 10.7115946 12.0603749 -51.9250526
H 10.0647863 12.8469327 -51.5572318
N 9.1602094 11.3777451 -55.2613215
H 9.967209 10.9333375 -55.6892098
C 7.9328766 10.5948167 -55.1189933
H 7.301889 11.0701356 -54.3646456
C 8.255057 9.1831792 -54.6477118
O 9.0891373 8.509012 -55.2435789
C 7.1597415 10.5440188 -56.4471444
H 7.7114147 9.9222563 -57.1494066
C 5.8095002 9.8629732 -56.2113163
H 5.4896513 10.0212756 -55.1882349
H 5.9140703 8.7885214 -56.3956002
H 5.0585275 10.2781186 -56.8705113
C 7.0334598 11.9183157 -57.1168015

H 6.7794035 12.6694729 -56.3779092
H 6.2773547 11.9109892 -57.8926942
H 7.9869524 12.1928344 -57.5734035
N 7.5304832 8.6922801 -53.6430702
H 6.8760449 9.3036927 -53.1726615
C 7.6681163 7.3252057 -53.1358409
H 8.2068826 6.7406371 -53.8801015
C 6.2876119 6.6673452 -52.9862792
O 5.4097723 7.1751835 -52.2984167
C 8.5623854 7.3067255 -51.8731256
H 9.5579647 7.6240205 -52.1890165
C 8.094785 8.2976582 -50.7891014
H 7.0661867 8.0877915 -50.5071326
H 8.1462975 9.3195321 -51.1659953
H 8.7354529 8.2405992 -49.9112774
C 8.6896144 5.865025 -51.3446308
H 7.7397634 5.5693468 -50.9079072
H 8.8984866 5.198318 -52.1818034
C 9.804598 5.6643442 -50.3096542
H 9.5898272 6.2220642 -49.3977556
H 10.7584064 5.9929064 -50.7211156
H 9.8773923 4.6049462 -50.0589894
N 6.0735872 5.5791504 -53.7322323
H 6.8322139 5.3057652 -54.3480851
C 4.8587987 4.7465541 -53.7745885
H 4.9169297 4.1923917 -54.7089597
C 3.516118 5.5057891 -53.8614048
O 2.5191161 5.1118456 -53.2532807
C 4.8607276 3.7088065 -52.6438668
H 4.6254463 4.2273965 -51.7263386
H 4.0580549 2.9937707 -52.8329263
C 6.1573545 2.9237375 -52.4338794
H 5.9667513 2.1123083 -51.7318169
H 6.9089625 3.5794581 -51.9982039
C 6.7097361 2.3381834 -53.7201912
O 7.8204849 2.6367907 -54.124086
N 5.9596121 1.5408023 -54.4434662
H 6.3860314 1.1601171 -55.2659575
H 4.9443976 1.5731702 -54.3602218
N 3.4922299 6.609794 -54.6151327
H 4.3734201 6.9183661 -54.9925454
C 2.3102518 7.4619524 -54.7883182
H 1.523502 7.1422596 -54.1042785
C 1.7272705 7.3825057 -56.209906
O 0.5213259 7.2247492 -56.3878293
C 2.7044591 8.890572 -54.3960494

H 3.4554001 9.2689719 -55.0839756
H 3.1257726 8.8895343 -53.3873539
S 1.267988 9.9956678 -54.455393
H 1.9581013 11.1116626 -54.1764562
N 2.5785643 7.3956426 -57.2408166
H 3.5667076 7.4519611 -57.0465178
C 2.1412431 7.4998195 -58.6453044
H 1.3706166 8.2684934 -58.6961616
C 1.455654 6.224998 -59.1639819
O 0.6846533 6.2812467 -60.1185169
C 3.3271499 7.9979964 -59.498981
H 4.2271993 7.4604443 -59.1935633
C 3.5120954 9.5030321 -59.2234801
H 2.7433385 10.070109 -59.7525158
H 3.3888159 9.7067547 -58.1618482
C 3.1360484 7.7830679 -61.0060698
H 2.1735433 8.1823495 -61.3276595
H 3.2063335 6.7193164 -61.2171017
H 3.9295564 8.2650958 -61.5743967
C 4.8911054 10.0097662 -59.6504475
H 5.0099305 9.9628237 -60.7314375
H 5.6735898 9.4236341 -59.1685517
H 4.9797716 11.0455733 -59.3443017
N 1.5717582 5.1041732 -58.4564899
H 2.1827045 5.1206104 -57.6529454
C 0.7328439 3.9149034 -58.651388
H 0.8947388 3.5462889 -59.6639284
C -0.7823536 4.1812482 -58.5331897
O -1.5480582 3.5308124 -59.2486143
C 1.1982313 2.8047712 -57.6992863
H 0.5310809 1.9524817 -57.8022436
H 2.1807305 2.4791663 -58.0396875
C 1.2976814 3.1948038 -56.2119626
H 0.3012642 3.1688227 -55.7657597
H 1.6944627 4.205053 -56.0979221
C 2.2384522 2.2254211 -55.4939914
O 3.4720441 2.4194285 -55.590334
O 1.7426194 1.2099946 -54.9540701
N -1.2002262 5.2068174 -57.7761104
H -0.5129244 5.7123035 -57.2254629
C -2.598939 5.6605511 -57.6700557
H -3.2650489 4.7962077 -57.7183515
C -3.0249164 6.6303356 -58.7861373
O -4.212663 6.7435325 -59.0897442
C -2.8084363 6.3407486 -56.3074555
H -3.8525083 6.6461592 -56.2269157

H -2.1951503 7.2411331 -56.2540386
C -2.494111 5.4648105 -55.1222584
N -3.2748369 4.3995001 -54.6813414
C -1.402258 5.5724542 -54.3123075
H -0.6083186 6.2963122 -54.4092074
C -2.6439835 3.8919381 -53.61534
H -2.9894624 3.0311948 -53.0587202
N -1.5076655 4.5702295 -53.3754836
H -0.8044458 4.3204002 -52.6953078
N -2.0794667 7.3391896 -59.4136958
H -1.1158875 7.1556197 -59.1719981
C -2.3616997 8.3287098 -60.4667779
H -3.2276859 8.9173499 -60.1602128
C -2.7464924 7.5875773 -61.7548098
O -2.001621 6.7304906 -62.2333305
C -1.169923 9.3091347 -60.6337605
H -0.2399088 8.7535995 -60.5132491
C -1.1908147 10.461597 -59.5993055
H -0.3002541 11.0742561 -59.749503
H -2.0610367 11.0934907 -59.7853356
C -1.1248122 9.968303 -62.0256172
H -2.0519598 10.5058078 -62.2273489
H -0.9656117 9.221869 -62.8030621
H -0.2929621 10.6711498 -62.0761009
C -1.2165483 10.0459058 -58.1225757
H -0.4028053 9.3553923 -57.9105932
H -2.1699243 9.5788426 -57.8756608
H -1.0975652 10.9321865 -57.4986943
N -3.9191623 7.9002296 -62.3131057
H -4.4943561 8.5906527 -61.8567087
C -4.4811881 7.1647627 -63.4544377
H -4.2521094 6.1048541 -63.3300885
C -3.8496852 7.5951677 -64.7889643
O -3.2727482 6.7640869 -65.4867586
C -6.0132403 7.3088003 -63.4796484
H -6.2694267 8.3537053 -63.6622205
H -6.4039576 6.7184002 -64.3096062
C -6.7166646 6.8608593 -62.1861027
H -7.7887787 7.0203155 -62.3018223
H -6.3872767 7.4812947 -61.3521355
C -6.4970237 5.3889815 -61.8459751
O -6.9995508 4.4963847 -62.5114945
N -5.7572628 5.0902401 -60.8042215
H -5.833909 4.14822 -60.4307188
H -5.3482376 5.8097848 -60.2156199
N -3.8569681 8.8981386 -65.0946658

H -4.3202539 9.5439063 -64.4785248
C -3.1401507 9.4583446 -66.2477274
H -3.0411782 8.6833109 -67.0107969
C -1.7236527 9.8698317 -65.8282051
O -1.4618108 10.9868417 -65.364135
C -3.9305454 10.5951731 -66.9132344
H -4.0322571 11.4261952 -66.2131145
H -4.9304066 10.2295566 -67.1538309
C -3.2659619 11.0967388 -68.2087631
O -2.0957811 10.7292587 -68.4770125
O -3.9030893 11.919111 -68.8986723
N -0.803794 8.9111941 -65.9612056
H -1.116004 8.0117478 -66.302686
C 0.6148988 9.1220086 -65.6716635
H 0.701243 9.6227611 -64.7065084
C 1.2656105 10.0741342 -66.6689175
O 2.098196 10.8648421 -66.2441968
C 1.3529267 7.7757301 -65.5926566
H 1.1126767 7.1689474 -66.4679944
H 2.4297305 7.955249 -65.5762918
C 0.9574017 7.0366377 -64.3062496
H 1.2856483 7.6316669 -63.4540904
H -0.1280695 6.9419732 -64.2625537
C 1.5717334 5.633152 -64.2128231
H 2.6407791 5.6991469 -64.4166036
H 1.1128656 4.9710833 -64.9484462
C 1.3819517 5.0770151 -62.7971826
H 1.7874912 5.8138986 -62.1066302
H 1.9538723 4.1524789 -62.6816544
N -0.0351696 4.8430507 -62.4374898
H -0.6226182 5.656999 -62.6128357
H -0.1159741 4.6848988 -61.4413045
H -0.410129 4.0257107 -62.9061462
N 0.857613 10.1083443 -67.9375912
H 0.0306158 9.5949737 -68.2147783
C 1.4627276 11.0419099 -68.8908006
H 2.5495178 10.9536548 -68.8456466
C 1.0955327 12.4971915 -68.5945729
O 1.9570158 13.368256 -68.6695282
C 1.0240044 10.7936445 -70.3246517
H -0.0383867 11.0268319 -70.4208665
H 1.5987591 11.5133366 -70.9013528
C 1.2870139 9.4334093 -70.9591885
H 0.6207275 8.6875652 -70.5204886
H 2.321262 9.1555211 -70.7495981
C 1.0670261 9.5267931 -72.4818624

O 1.5837226 8.6346019 -73.1863309
O 0.449561 10.5278113 -72.9382914
N -0.1651418 12.7909911 -68.2489103
H -0.8538143 12.0316837 -68.2624408
C -0.597046 14.1400191 -67.8500139
H -0.3606733 14.8368522 -68.6493023
C 0.1788132 14.6128788 -66.6331437
O 0.6875716 15.7306213 -66.6664212
C -2.1126775 14.1466946 -67.5941196
H -2.6227997 14.0571914 -68.5530931
H -2.3576696 13.272807 -66.9910062
C -2.6896842 15.3612165 -66.8459386
H -3.7652708 15.2087699 -66.7430743
H -2.2667664 15.3935762 -65.8416272
C -2.4521123 16.7168604 -67.5216241
H -2.901966 16.7154243 -68.5155877
H -1.3820194 16.9074539 -67.6060141
C -3.0951271 17.8042082 -66.6541248
H -2.7560098 17.6658543 -65.6223159
H -4.1813943 17.6762267 -66.6795216
N -2.71647 19.159312 -67.1134731
H -1.700728 19.2804625 -67.0277463
H -3.1123092 19.8764223 -66.5258363
H -2.9676407 19.3212056 -68.075571
N 0.3204954 13.766832 -65.6119406
H -0.1301998 12.8600258 -65.6626692
C 1.1372004 14.0988161 -64.4489276
H 0.7681291 15.0311553 -64.0186157
C 2.6010132 14.3488103 -64.8440332
O 3.1203401 15.4231067 -64.5515805
C 1.0048208 13.0052398 -63.3833035
H -0.0274297 12.9715737 -63.0338871
H 1.2416141 12.0362933 -63.8251743
C 1.9196494 13.2544928 -62.2026704
C 1.5902525 14.2330469 -61.2449909
H 0.6507624 14.7645696 -61.3091331
C 2.5054578 14.5589645 -60.2283914
H 2.2645 15.3361693 -59.5185997
C 3.7477584 13.9046379 -60.163313
H 4.4610224 14.1635952 -59.3939693
C 4.075634 12.9324819 -61.1222156
H 5.0420339 12.4538226 -61.0965527
C 3.1634757 12.6011592 -62.1355357
H 3.4384687 11.8742898 -62.8856969
N 3.2219607 13.4431795 -65.6129092
H 2.7236004 12.5935768 -65.8627977

C 4.6285742 13.5613066 -66.0272132
H 5.2190656 13.6913037 -65.136371
C 4.8554892 14.8103973 -66.9071792
O 5.7538651 15.5982238 -66.6248052
C 5.1469566 12.2423806 -66.6375441
H 4.5264298 12.0049949 -67.503892
C 6.6112232 12.3569321 -67.0848273
H 7.294004 12.1712733 -66.2581363
H 6.8394987 13.3368053 -67.4929083
H 6.78462 11.6171695 -67.8603109
C 5.0988351 11.0728698 -65.6156212
H 5.8917595 11.1882859 -64.8758281
H 4.1709873 11.0890031 -65.0529447
C 5.2309691 9.6913749 -66.2729081
H 6.195366 9.5950969 -66.7695518
H 4.4343643 9.5515472 -67.0036455
H 5.1519131 8.9179254 -65.5089451
N 3.9636351 15.1292163 -67.8520424
H 3.2265652 14.4600239 -68.0547949
C 4.0218868 16.3871815 -68.6226211
H 5.0042765 16.4596209 -69.0901891
C 3.8797531 17.6361744 -67.751329
O 4.3853571 18.6900604 -68.1393617
C 2.9423549 16.3991231 -69.7126516
H 1.9845357 16.1193492 -69.2705717
H 2.8543825 17.4158555 -70.0989016
C 3.2701847 15.4763868 -70.8965743
H 3.2834725 14.434007 -70.5792135
H 4.2600221 15.7274747 -71.2810722
C 2.2462379 15.6651832 -72.0260354
H 2.5661878 15.0769262 -72.8877034
H 2.2537104 16.7118842 -72.3344896
N 0.873631 15.2917612 -71.613203
H 0.3736673 15.9475456 -71.0440993
C 0.2606047 14.1451865 -71.8617678
N 0.8324717 13.2109762 -72.562001
H 0.4215544 12.2750905 -72.7048818
H 1.7817286 13.3170273 -72.8573687
N -0.9394563 13.9204432 -71.4047869
H -1.4254517 14.5628763 -70.815964
H -1.3235025 12.9947997 -71.5411287
N 3.1468849 17.5726836 -66.640555
H 2.7147858 16.6893069 -66.3908726
C 3.0422072 18.6861717 -65.6891141
H 2.9536692 19.6115987 -66.2544648
C 4.3172776 18.8331359 -64.8542892

O 4.818539 19.9522103 -64.7316767
C 1.7891423 18.5422215 -64.8007602
H 1.0555546 17.9044931 -65.2924164
H 2.0612306 18.0606817 -63.8602092
C 1.1195113 19.8957002 -64.5028273
H 1.8473703 20.5638183 -64.0369374
H 0.3048766 19.7346467 -63.7929669
C 0.5491297 20.5310302 -65.7790337
O -0.2282403 19.8629453 -66.4986209
O 0.9457575 21.6510811 -66.1609826
N 4.94132 17.725144 -64.4234338
H 4.4998686 16.8199543 -64.5638902
C 6.2140599 17.8019982 -63.6909489
H 6.0524835 18.5815138 -62.9562327
C 7.3890477 18.3481159 -64.518059
O 8.2732855 18.9822581 -63.9445966
C 6.5604161 16.5360761 -62.8697259
H 7.6036421 16.612572 -62.5826921
C 5.687129 16.5745079 -61.5928064
H 4.6438896 16.3754152 -61.8451636
H 5.7360653 17.5498829 -61.1123979
H 6.0256541 15.83006 -60.8757306
C 6.3611885 15.1622226 -63.5084856
H 5.298497 14.9474787 -63.5301493
H 6.74844 15.1843201 -64.5161972
C 7.0610066 14.0140301 -62.7739185
H 6.6684855 13.8981371 -61.7667884
H 8.122155 14.2216184 -62.7084251
H 6.9110273 13.0840706 -63.3228865
N 7.30301 18.2991724 -65.852446
H 6.594574 17.6978391 -66.2541671
C 8.2430857 18.9945263 -66.7521705
H 9.256973 18.7089679 -66.4899572
C 8.1690656 20.51075 -66.5818082
O 9.1990222 21.1611148 -66.460308
C 7.977772 18.6399987 -68.229651
H 6.9892592 19.0220523 -68.484297
C 8.9845327 19.2914653 -69.1874602
H 10.0014391 18.9979181 -68.9194354
H 8.9126701 20.3778295 -69.1491886
H 8.7886603 18.966429 -70.2109033
C 7.96589 17.1408008 -68.5243393
H 8.943109 16.8146886 -68.8779901
H 7.2357508 16.9438971 -69.3046957
H 7.7171778 16.551408 -67.6508645
N 6.9615765 21.0893303 -66.532496

H 6.1488449 20.486938 -66.5022801
C 6.780615 22.5474602 -66.4135413
H 7.4330732 23.0541204 -67.1286945
C 7.2202667 23.0717325 -65.0569848
O 7.912679 24.0815246 -64.9821131
C 5.3159302 22.9327156 -66.6760503
H 4.6831159 22.049465 -66.5844177
H 4.9780527 23.653042 -65.9286919
C 5.1585947 23.5769347 -68.0589042
H 5.5905341 22.9257093 -68.8152424
H 5.7275276 24.5057287 -68.0837541
C 3.6905468 23.8966561 -68.3952165
H 3.6380314 24.2920585 -69.4108383
H 3.3606373 24.6917945 -67.7218206
N 2.7656957 22.7454938 -68.2527494
H 2.0283473 22.845749 -67.5579178
C 2.88091 21.5274568 -68.7454514
N 3.8693124 21.140862 -69.4991559
H 4.0949378 20.1565639 -69.4957941
H 4.5709032 21.8214323 -69.7161739
N 1.9903342 20.6334323 -68.4586807
H 1.2955849 20.8980357 -67.7519289
H 2.1477213 19.6774308 -68.6872359
N 6.8385406 22.3850171 -63.9793491
H 6.2678414 21.5594362 -64.1188139
C 7.0762139 22.8882716 -62.6154744
H 6.7536171 23.9302454 -62.5989197
C 8.5529194 22.901266 -62.209056
O 8.8936518 23.5075295 -61.1960446
C 6.2302789 22.1490035 -61.5659052
H 6.3002834 22.7158685 -60.6375336
C 4.7406745 22.1370212 -61.9312552
H 4.5608851 21.5072413 -62.7992222
H 4.4104188 23.1524728 -62.155835
H 4.1599278 21.7513496 -61.0951958
C 6.7187884 20.740197 -61.2639461
H 6.946144 20.2365441 -62.1944734
H 5.9697426 20.1820863 -60.7022476
H 7.6299397 20.7966649 -60.6732389
N 9.4330305 22.2598839 -62.9727844
H 9.1190088 21.8290885 -63.831774
C 10.8586508 22.2157185 -62.6987607
H 11.0205601 22.3896924 -61.6385775
C 11.6587618 23.2613323 -63.4943908
O 11.250341 23.686733 -64.571033
C 11.3162717 20.8126336 -63.009183

H 11.2222436 20.673128 -64.0870845
H 10.6948542 20.0962774 -62.4706286
H 12.34619 20.6925253 -62.698318
N 12.8048385 23.6814376 -62.9622398
H 13.0708624 23.3246349 -62.0518115
C 13.7495271 24.5578181 -63.6509041
H 13.1778 25.2862108 -64.2238724
C 14.6719354 23.7794981 -64.6223379
O 14.975169 22.6149417 -64.3663321
C 14.5534374 25.3133525 -62.584314
H 15.1349368 24.6050227 -61.9900518
H 13.8784704 25.8645982 -61.9278724
H 15.2402197 26.0126728 -63.0612602
N 15.2562672 24.4346856 -65.6428965
C 16.3457623 23.8565942 -66.4329207
H 15.9645131 23.0084207 -66.9972777
C 17.5084646 23.3942187 -65.5437926
O 17.9666931 24.1420647 -64.6815619
C 16.7604415 24.9539896 -67.41675
H 17.5396302 25.5804047 -66.9808329
H 17.0916402 24.5356973 -68.368371
C 15.4800201 25.7739039 -67.5748403
H 14.8102338 25.2746548 -68.2775496
H 15.6876092 26.7949285 -67.8961341
C 14.8755827 25.7311526 -66.1737705
H 13.7917232 25.8368128 -66.2352599
H 15.3069372 26.5190763 -65.555338
N 17.9761557 22.1537945 -65.7184936
H 17.5578649 21.5750566 -66.4370437
C 18.9691881 21.5330127 -64.828799
H 19.4528712 20.7173447 -65.3653614
H 19.7268063 22.271958 -64.5676816
C 18.3945524 20.9579177 -63.5231969
O 19.1431088 20.4234825 -62.6929978
N 17.0763422 20.9995032 -63.325819
H 16.4871733 21.5126475 -63.9742723
C 16.4200515 20.282351 -62.2438022
H 16.8992307 20.5608404 -61.3042546
C 16.5430015 18.7673145 -62.4295642
O 16.5979498 18.2344241 -63.5433115
C 14.9511917 20.6876016 -62.1594518
H 14.4759675 20.4721613 -63.1160417
H 14.876111 21.7519627 -61.935609
H 14.4412873 20.1255796 -61.3775577
N 16.4551063 18.0541021 -61.3125826
H 16.2855776 18.5690471 -60.4511779

C 16.3207142 16.6066863 -61.2672556
H 16.667364 16.1436285 -62.1925943
C 14.8430527 16.4876811 -61.1714432
O 14.2703098 16.4918358 -60.0760996
C 17.1025975 16.0055349 -60.0967373
H 16.7267382 16.4254048 -59.1674263
H 16.9332383 14.927969 -60.0696969
C 18.6151246 16.2789271 -60.2035818
H 18.7934009 17.3000794 -60.5397025
H 19.0498173 15.6006874 -60.9384452
C 19.3309473 16.1098317 -58.8681021
O 19.8209138 17.063498 -58.2767724
N 19.4280626 14.9016131 -58.3528361
H 19.9160454 14.8206799 -57.47876
H 19.0269415 14.1074667 -58.8221429
N 14.2900884 16.7928196 -62.3621341
H 14.9082487 16.9578584 -63.1449686
C 12.8660165 16.8921841 -62.5786515
H 12.50313 17.8129391 -62.118628
C 12.3945187 16.8195691 -64.0583579
H 11.5182164 16.1806944 -64.1205188
C 11.927267 18.2080647 -64.4477732
H 12.7140434 18.9289267 -64.2293846
H 11.0102521 18.4103486 -63.8975376
H 11.6871596 18.2879955 -65.5028421
C 13.3973882 16.3601632 -65.1198995
H 13.1242213 16.743578 -66.1014841
H 14.3632553 16.7589904 -64.8585091
C 13.4802412 14.8541873 -65.2635705
H 12.5137973 14.4524802 -65.5470749
H 13.7913259 14.4259101 -64.3196536
H 14.1809111 14.6061911 -66.0551717
C 12.2832975 15.764129 -61.7971147
O 11.2494559 16.0014477 -61.1925983
N 12.9953431 14.6160479 -61.6922139
H 13.8487719 14.4634771 -62.2140222
C 12.4409562 13.5648977 -60.9104669
H 11.9661348 14.0429408 -60.056893
C 13.3950247 12.5287825 -60.3251963
O 14.3898634 12.1321749 -60.9337501
C 11.3641755 12.9163869 -61.7760491
H 11.69877 11.9263678 -62.0871113
C 10.1448767 12.7675339 -60.8818847
H 9.7797381 13.7482455 -60.5753726
H 10.389334 12.200937 -59.985975
H 9.3515675 12.2659959 -61.4236928

C 10.8142608 13.647437 -63.0167973
H 10.2651247 14.5505195 -62.7809551
H 10.1246692 12.9691349 -63.5158578
H 11.6027534 13.8396563 -63.7244018
N 12.992268 12.0624941 -59.1407394
H 12.2290494 12.5696896 -58.7005972
C 13.3517678 10.7991302 -58.4875107
H 13.8691189 10.1454328 -59.1901295
C 12.022295 10.1409558 -58.0722694
O 11.2124517 10.7801555 -57.3905926
C 14.2732964 11.0619765 -57.2714117
H 13.7556098 11.7467959 -56.5983282
C 14.5539489 9.7552096 -56.5040559
H 15.098085 9.0547942 -57.1393494
H 13.6255968 9.2720965 -56.1977078
H 15.1326487 9.9473315 -55.6028041
C 15.5967541 11.7351388 -57.7104154
H 16.1322448 11.0671286 -58.3834904
H 15.3704105 12.6510699 -58.2557262
C 16.5299331 12.1281646 -56.5575202
H 16.9353557 11.2398739 -56.0737671
H 15.9862613 12.7290053 -55.8275692
H 17.3598219 12.7148318 -56.9523203
N 11.7580335 8.9046503 -58.5018071
H 12.4971308 8.4049395 -59.0006782
C 10.4983895 8.1867901 -58.2471085
H 9.9675434 8.6814518 -57.4396159
C 10.7986262 6.7492106 -57.8252037
O 11.102295 5.9443846 -58.7003625
C 9.5576584 8.1569015 -59.488233
H 10.0657489 7.6118162 -60.2850846
C 9.2280089 9.5527545 -60.0531933
H 8.7489912 10.1650888 -59.2892799
H 10.1684049 10.018405 -60.3410172
C 8.2538598 7.3944316 -59.1507915
H 7.707404 7.913199 -58.3643917
H 8.4738782 6.3822778 -58.8197286
H 7.6197814 7.2880813 -60.0276344
C 8.3419087 9.5094014 -61.311817
H 7.3388524 9.1658198 -61.0701747
H 8.7868381 8.8466241 -62.055071
H 8.2449684 10.4990386 -61.7479271
N 10.3778145 6.3647875 -56.6173089
H 10.0867171 7.0875804 -55.969359
C 10.0771669 4.965996 -56.2829504
H 10.6161371 4.3342597 -56.978693

C 8.5735844 4.707958 -56.4325046
O 7.7547345 5.5273144 -56.0055968
C 10.6004787 4.5910992 -54.8968489
H 10.1061673 5.1945444 -54.1366728
H 11.6735419 4.7889482 -54.8607758
O 10.3828632 3.2140938 -54.6418497
H 9.4472056 3.0366627 -54.4338089
N 8.1607001 3.6309068 -57.1055167
H 8.8769937 2.9804449 -57.4273788
C 6.7371884 3.300688 -57.3620578
H 6.1596131 3.5083897 -56.4621738
C 6.5491433 1.8064488 -57.6597888
O 7.4787363 1.1227106 -58.0834072
C 6.1822375 4.201798 -58.4902398
H 6.9381585 4.3099942 -59.2694911
C 4.8723056 3.766805 -59.1432156
H 4.1171477 3.6139869 -58.3752925
H 5.0235349 2.8460121 -59.7068042
H 4.5357146 4.5394803 -59.8340945
O 5.8902408 5.4700017 -57.9372644
H 6.6083844 5.6589023 -57.3062582
N 5.3715908 1.2506011 -57.3572631
H 4.6553821 1.8125038 -56.8983762
C 5.0533527 -0.1448405 -57.6414244
H 5.9175855 -0.7539901 -57.3692877
C 4.7686066 -0.3651727 -59.1348527
O 3.9382459 0.3149866 -59.7426248
C 3.8790949 -0.6020956 -56.7719286
H 2.9852171 -0.0491227 -57.0590648
H 4.0992285 -0.4156114 -55.7198245
H 3.7121333 -1.6687299 -56.9224242
N 5.384449 -1.4132231 -59.6710028
H 5.9615485 -1.978817 -59.0585404
C 5.274073 -1.8707675 -61.0481607
H 4.52063 -1.2819514 -61.5709594
C 4.819635 -3.3368143 -61.111532
O 4.71484 -4.0327033 -60.0985856
C 6.6290324 -1.6468094 -61.7413333
H 6.7247861 -2.2962304 -62.6122492
H 7.4480343 -1.8571985 -61.0513851
S 6.7161264 0.0744788 -62.2988248
H 5.7073454 -0.0156779 -63.1767116
N 4.5804398 -3.8163232 -62.3306187
H 4.6770828 -3.1691214 -63.1064769
C 4.346574 -5.2294842 -62.6551505
H 4.459447 -5.8264837 -61.7510928

C 5.4032849 -5.7429896 -63.6329606
O 6.1897972 -4.9572822 -64.1649364
C 2.9059161 -5.4309908 -63.1662228
H 2.7672556 -6.4849372 -63.4143421
H 2.2061259 -5.1920836 -62.3647088
C 2.5629917 -4.5759187 -64.3962885
H 3.3538419 -4.6507815 -65.1381943
H 2.4774119 -3.5317996 -64.1082129
C 1.239358 -5.0199314 -65.0200982
H 0.4409992 -4.8935195 -64.2851521
H 1.2911458 -6.0804497 -65.2787751
N 0.9258565 -4.2126199 -66.212061
H 0.4081314 -3.3590145 -66.0351975
C 1.2259418 -4.5210722 -67.4631425
N 1.9688723 -5.5468659 -67.7776155
H 2.1152903 -5.8721481 -68.7219343
H 2.3570204 -6.1389378 -67.0480665
N 0.7801215 -3.779182 -68.4391839
H 0.1596895 -3.0162654 -68.2328983
H 0.9754474 -4.0537471 -69.3841574
N 5.3899017 -7.0384504 -63.929528
H 4.7383203 -7.6344439 -63.4363782
C 6.0942102 -7.5596355 -65.1036889
H 7.0977374 -7.1300138 -65.1006469
C 5.4232967 -7.0925274 -66.4226341
O 4.3354509 -6.5106139 -66.4170726
C 6.2677109 -9.0838745 -64.9594063
H 6.6936781 -9.2945219 -63.9792089
H 6.9785677 -9.4402086 -65.7029615
C 4.9912565 -9.885612 -65.1365704
O 4.2025192 -9.6370408 -66.0222303
N 4.7808317 -10.9165378 -64.3629691
H 3.901636 -11.390675 -64.4666077
H 5.4341878 -11.1196392 -63.6117621
N 6.087026 -7.301176 -67.5654737
H 6.97492 -7.7768733 -67.5279906
C 5.5686416 -6.8960675 -68.8824775
H 5.3268371 -5.8354456 -68.847767
C 4.2717675 -7.636028 -69.2757134
O 3.3960169 -7.0361793 -69.9063768
C 6.6966829 -7.1057223 -69.9118019
H 7.5465829 -6.4850685 -69.622889
H 7.0144693 -8.1489414 -69.8602459
C 6.3428838 -6.7968035 -71.3816833
H 5.5274226 -7.4439008 -71.7033055
C 5.9359276 -5.3387014 -71.5982807

H 6.7312629 -4.6712647 -71.263949
H 5.0185384 -5.1252485 -71.0539862
H 5.7487945 -5.1681951 -72.6580208
C 7.5553389 -7.0841982 -72.2684394
H 8.3866897 -6.4341584 -71.9947723
H 7.2949337 -6.9136733 -73.313153
H 7.8578121 -8.1251123 -72.1530906
N 4.1334798 -8.8979656 -68.8636108
H 4.8250579 -9.2749777 -68.2335885
C 3.0282568 -9.7990911 -69.2066128
H 2.994302 -9.8630138 -70.2915907
C 1.6602333 -9.3119172 -68.6927965
O 1.5945623 -8.516802 -67.7502102
C 3.3072741 -11.2092038 -68.6610331
H 2.5364612 -11.8929918 -69.0166306
H 3.2739869 -11.2045757 -67.5732222
O 4.5717364 -11.6737482 -69.1032695
H 4.7435367 -12.5260357 -68.6915725
N 0.5433211 -9.7783737 -69.2905212
C -0.7985269 -9.4881579 -68.7920022
H -0.974344 -8.4156383 -68.878059
C -0.9754439 -9.9212677 -67.3331253
O -0.527133 -10.9988821 -66.9390965
C -1.7659872 -10.2366409 -69.7171588
H -1.976375 -11.2318292 -69.3196156
H -2.693753 -9.6827823 -69.8650191
C 0.4554123 -10.5666366 -70.5124142
H 1.1583618 -10.2258453 -71.2721388
H 0.6242898 -11.6193873 -70.280253
C -0.973233 -10.3730318 -71.0152853
H -1.0393598 -9.4455678 -71.586019
H -1.3115491 -11.2200922 -71.6130074
N -1.6717958 -9.0991233 -66.547554
H -1.9774122 -8.2113845 -66.9104634
C -1.9426282 -9.3712957 -65.1363939
H -0.9869589 -9.4906116 -64.6280691
C -2.7599268 -10.6578647 -64.9656591
O -3.8400503 -10.7898855 -65.5414905
C -2.701309 -8.1901542 -64.5041285
H -2.7718945 -8.3380187 -63.4261289
H -3.7101012 -8.1606276 -64.9151862
O -2.0772418 -6.945159 -64.7763668
H -2.4140318 -6.2669389 -64.1668781
N -2.292544 -11.5662814 -64.1043462
H -1.3627723 -11.44079 -63.7365719
C -3.0207712 -12.7921241 -63.7457168

H -3.2244875 -13.323451 -64.6781217
C -4.3966937 -12.4684447 -63.1343671
O -4.6230048 -11.3825377 -62.579092
C -2.1096583 -13.72106 -62.9038127
H -1.910148 -14.6234283 -63.4834728
H -1.1418491 -13.2284864 -62.8004951
C -2.5148567 -14.1252411 -61.4840284
H -2.7946617 -13.2267165 -60.9280213
H -1.6416138 -14.553518 -60.9864657
C -3.6505368 -15.1492933 -61.4302847
O -4.2452488 -15.22482 -60.332582
O -4.0275463 -15.7087837 -62.4791995
N -5.3275999 -13.4242844 -63.2208885
H -5.0081732 -14.3620026 -63.4746751
C -6.7373853 -13.2558538 -62.8708644
H -7.1275333 -12.4809128 -63.5271843
C -6.946757 -12.7679238 -61.4410492
O -7.9117529 -12.0322895 -61.2615722
C -7.4812086 -14.5788163 -63.1367407
H -7.2969487 -14.8900304 -64.1668939
H -7.0568127 -15.3459934 -62.4846586
C -8.9995942 -14.5392484 -62.9048429
H -9.3885239 -15.5543231 -62.9959171
H -9.1947796 -14.2219061 -61.8805407
C -9.7553918 -13.6445641 -63.9063237
H -10.1326223 -14.2766743 -64.7127237
H -9.0952164 -12.9031723 -64.3565096
C -10.9343519 -12.935928 -63.2296966
H -11.4774207 -13.6713244 -62.6279936
H -11.6147183 -12.563259 -64.0023007
N -10.4659423 -11.8111498 -62.3804404
H -9.7675482 -12.1340372 -61.710333
H -11.2343898 -11.3862407 -61.8810379
H -10.0067443 -11.1094506 -62.9439105
N -6.0816248 -13.0886981 -60.4718269
H -5.3969747 -13.8110332 -60.6825751
C -6.0968226 -12.5397581 -59.1017552
H -7.1172031 -12.2357542 -58.8791371
C -5.2212816 -11.2922417 -58.8815129
O -5.4720267 -10.5585664 -57.9262851
C -5.7549487 -13.6167123 -58.0640669
H -6.3952429 -14.4869167 -58.2181221
H -5.9482444 -13.2182487 -57.0671658
O -4.3992676 -14.0098058 -58.1432093
H -4.343102 -14.5866227 -58.960205
N -4.2784466 -10.9636136 -59.7796263

H -4.191226 -11.5356199 -60.6116894
C -3.5645761 -9.675878 -59.7459851
H -3.2982635 -9.4612025 -58.710529
C -4.4764878 -8.5391038 -60.2170946
O -4.6424323 -7.5619692 -59.492832
C -2.2637066 -9.7208699 -60.5782299
H -1.5860132 -10.4576694 -60.1459091
H -2.5060962 -10.0411425 -61.5913691
C -1.54592 -8.348949 -60.6594275
H -2.2252017 -7.6004366 -61.0640231
C -1.049715 -7.869789 -59.2926931
H -0.3925492 -8.6162577 -58.8481829
H -1.895953 -7.6778135 -58.6345231
H -0.5004847 -6.9352805 -59.4146506
C -0.3461376 -8.4080241 -61.5969519
H 0.3865714 -9.1103831 -61.226032
H 0.1108929 -7.4212003 -61.6794127
H -0.6523785 -8.7230191 -62.5882788
N -5.1169134 -8.689943 -61.3849318
H -4.9457027 -9.5459942 -61.90868
C -6.0412299 -7.6860189 -61.9416269
H -5.4551489 -6.7857532 -62.1437637
C -7.1240799 -7.231914 -60.9375162
O -7.2297424 -6.0346225 -60.7273625
C -6.5934143 -8.1928127 -63.2907024
H -5.7661369 -8.2380254 -63.9984981
H -6.9541082 -9.2147834 -63.16253
C -7.7329938 -7.3712305 -63.9310273
H -7.8971692 -7.7500898 -64.9408023
H -8.6442331 -7.5659483 -63.3636415
C -7.5575872 -5.8442729 -64.0094301
H -7.4980538 -5.4352241 -63.0077227
H -8.4615509 -5.4163366 -64.4482493
C -6.3614685 -5.3390559 -64.8205827
H -6.662531 -5.2804452 -65.8695286
H -5.5195257 -6.0309988 -64.7300999
N -5.9571078 -4.0034871 -64.3263948
H -6.7679802 -3.395136 -64.17414
H -5.2630821 -3.5587778 -64.9038722
H -5.4837897 -4.0957187 -63.4295262
N -7.871645 -8.0931999 -60.2239082
C -8.873936 -7.6616363 -59.2573345
H -9.5234761 -6.9139405 -59.7159941
C -8.2649088 -7.0320422 -57.9992117
O -8.9337415 -6.198365 -57.3998078
C -9.7099499 -8.8997193 -58.9176722

H -10.0869565 -8.8805224 -57.8938592
H -10.5345133 -8.9914458 -59.6264198
C -7.9351771 -9.5147877 -60.3439147
H -8.4774898 -9.7379089 -61.262144
H -6.9490279 -9.9651637 -60.3563997
C -8.720291 -10.0246495 -59.1344312
H -9.2281338 -10.9685741 -59.3297095
H -8.0659944 -10.10908 -58.2666461
N -7.021786 -7.3606245 -57.6043131
H -6.4998668 -8.0322734 -58.1535313
C -6.3056253 -6.6353359 -56.5348897
H -6.9728801 -6.5111878 -55.6824392
C -5.9322881 -5.2270172 -57.0036012
O -6.1602659 -4.2700824 -56.2681912
C -5.0687729 -7.4390979 -56.0860549
H -5.3895788 -8.424192 -55.7418211
H -4.4305065 -7.5948128 -56.9556649
C -4.1992388 -6.7757217 -54.993123
H -3.2115868 -7.2341931 -55.0481208
H -4.0668317 -5.7140503 -55.2046993
C -4.7169933 -6.9550987 -53.5545555
H -4.8133604 -8.0239603 -53.351868
H -5.6999526 -6.4874112 -53.4551922
C -3.7314371 -6.3211879 -52.5553124
H -3.9467867 -5.2535484 -52.4822222
H -2.7197203 -6.4281609 -52.9491867
N -3.7880118 -6.9492504 -51.2117734
H -4.7008678 -6.8646728 -50.7950431
H -3.1294465 -6.5095671 -50.5629086
H -3.5314121 -7.9249485 -51.2652042
N -5.4101965 -5.0928218 -58.2204721
H -5.2513331 -5.9347831 -58.7634563
C -5.1139113 -3.8079675 -58.8626221
H -4.4172266 -3.2441876 -58.2414818
C -6.3827766 -2.9486339 -58.9977917
O -6.424878 -1.8461333 -58.4601641
C -4.4306352 -4.0925634 -60.2147685
H -3.4889227 -4.612916 -60.0251308
H -5.0681263 -4.750292 -60.8032861
C -4.1402352 -2.8329003 -61.0402102
H -3.5223312 -2.1630568 -60.4391233
H -5.0881717 -2.3339607 -61.2560169
C -3.4537445 -3.1310734 -62.3813936
O -3.6879035 -4.2298107 -62.9487496
O -2.7668995 -2.224337 -62.9038172
N -7.4664462 -3.5002571 -59.5499567

H -7.3734285 -4.4240226 -59.9575726
C -8.7116426 -2.7639545 -59.7964255
H -8.4353106 -1.8095062 -60.2480485
C -9.4638687 -2.4089424 -58.5074308
O -9.9528278 -1.288221 -58.3755535
C -9.6118555 -3.5466591 -60.7821039
H -9.0607949 -4.3693492 -61.2330581
H -10.4668038 -3.9705644 -60.2532929
C -10.1111674 -2.6392604 -61.9230753
H -10.7214885 -1.8416841 -61.4932013
H -10.7558622 -3.2309231 -62.5768496
C -8.9667387 -2.0313695 -62.7636765
O -8.2371866 -2.7994259 -63.4391515
O -8.8066446 -0.7847794 -62.7441526
N -9.47042 -3.2962327 -57.4999447
H -9.0417097 -4.20374 -57.6524768
C -10.1010431 -3.0170332 -56.1973756
H -11.0288266 -2.4764864 -56.3973745
C -9.2769901 -2.0761537 -55.3142795
O -9.8498703 -1.4157787 -54.4554675
C -10.4660358 -4.334596 -55.4944689
H -11.0058283 -4.9560219 -56.2112972
H -9.5566922 -4.8544857 -55.1890845
C -11.3704398 -4.1053174 -54.2696322
H -10.7558442 -3.807233 -53.4188719
H -12.0692675 -3.2953097 -54.4855386
C -12.2007088 -5.3376816 -53.8874421
H -12.8839548 -5.5640901 -54.7082912
H -12.7938671 -5.0865768 -53.0059178
C -11.3301939 -6.5636396 -53.5898349
H -10.6293076 -6.3072624 -52.7892185
H -10.7480368 -6.8020651 -54.4859351
N -12.1634946 -7.726805 -53.1936491
H -12.701381 -7.5095402 -52.3633894
H -11.5843428 -8.534113 -52.9993255
H -12.8073944 -7.9650457 -53.9371857
N -7.9716141 -1.9444161 -55.5661454
H -7.5758423 -2.5040646 -56.3110106
C -7.1488087 -0.8533673 -55.0040867
H -7.6222074 -0.5067725 -54.0856447
C -7.1064176 0.3871534 -55.9041949
O -6.4532318 1.363562 -55.5514579
C -5.7327836 -1.3132419 -54.6059369
H -5.1704231 -0.4493004 -54.2512706
C -5.7686133 -2.3392788 -53.4714731
H -6.3200593 -3.2274988 -53.7777969

H -6.2540956 -1.8980821 -52.6008782
H -4.7497511 -2.6174525 -53.2046511
O -5.0121386 -1.9015539 -55.6648057
H -5.5081724 -2.690785 -55.9458878
N -7.8166632 0.3654646 -57.0417343
H -8.3462446 -0.4726061 -57.2427193
C -7.8502309 1.3899778 -58.0923619
H -8.3461036 0.901136 -58.9292334
C -6.4594769 1.7892235 -58.633805
O -6.3001095 2.8344891 -59.2640218
C -8.7526115 2.5574542 -57.6322694
H -9.6187161 2.1305136 -57.1235023
H -8.1999693 3.1478449 -56.8997247
C -9.2803135 3.5032468 -58.7320415
H -8.4579137 4.0436594 -59.1952806
C -10.0659324 2.771923 -59.8264709
H -10.8596408 2.1741863 -59.3761475
H -9.4021933 2.1159166 -60.3858728
H -10.5024011 3.4948423 -60.5147045
C -10.2132689 4.5376623 -58.0986059
H -11.0746174 4.0426197 -57.649767
H -10.5543428 5.2422816 -58.85601
H -9.6730952 5.0878871 -57.3275553
N -5.4426723 0.9434196 -58.4481881
H -5.6678236 0.0355238 -58.0548346
C -4.0798935 1.185912 -58.9357536
H -3.8150141 2.2211407 -58.7113507
C -4.0051982 1.032849 -60.4518383
O -4.7678273 0.27695 -61.0434649
C -3.0949649 0.2568287 -58.2130025
H -3.5138127 -0.7490093 -58.1422002
H -2.1678002 0.2159683 -58.784929
C -2.795052 0.8141 -56.8097441
H -2.3119699 1.7866437 -56.9208895
H -3.7301621 0.9656837 -56.2690902
C -1.8893985 -0.0773955 -55.9502131
H -2.4545609 -0.9480272 -55.6154601
H -1.5950424 0.5033683 -55.0736083
C -0.6439782 -0.5463608 -56.7108241
H -0.3018319 0.268945 -57.3493463
H -0.9215663 -1.3830817 -57.3566641
N 0.4467047 -0.9339892 -55.7916971
H 0.7465728 -0.112789 -55.2498367
H 1.2748543 -1.2105249 -56.2979961
H 0.1611309 -1.6486113 -55.1440047
N -3.0487572 1.7262357 -61.0774219

H -2.4915023 2.3509615 -60.5015827
C -2.7384924 1.6213578 -62.5167704
H -3.2764944 0.767828 -62.9341887
C -1.2710996 1.2723612 -62.7035448
O -0.4066791 2.1504201 -62.6343866
C -3.1835379 2.9095297 -63.2545982
H -3.4195988 3.6894009 -62.5300726
H -2.3764105 3.2816492 -63.8842477
C -4.4157079 2.6815394 -64.1627795
H -4.7723658 3.6569256 -64.4963477
H -4.1117891 2.1108096 -65.0407656
C -5.5713926 1.9356428 -63.4710507
H -5.670715 2.3288555 -62.460506
H -5.3424209 0.8725846 -63.4125411
C -6.9189723 2.0674559 -64.1837241
H -6.9312356 1.427326 -65.0694601
H -7.0421595 3.108668 -64.4916204
N -8.0097774 1.7033435 -63.2510456
H -7.9605382 0.719054 -62.960874
H -8.919042 1.7965361 -63.6756161
H -7.9574248 2.2846703 -62.4272009
N -0.9851324 -0.0152724 -62.8685472
H -1.7507671 -0.6921228 -62.9343551
C 0.3622951 -0.5630426 -62.7563147
H 0.9805693 0.166144 -62.2307181
C 0.9623679 -0.7635467 -64.1569443
O 0.4536773 -1.5034089 -65.0035394
C 0.3662204 -1.8384641 -61.8795023
H -0.0630003 -2.6610733 -62.4548948
C 1.819163 -2.1735927 -61.5191769
H 2.2062048 -1.4454116 -60.8056847
H 2.4410637 -2.152216 -62.4072739
H 1.8767011 -3.1699076 -61.08193
C -0.4305112 -1.6738873 -60.5577608
H 0.0351023 -0.9039866 -59.9410057
H -1.442414 -1.3440835 -60.7786126
C -0.5557201 -2.9669112 -59.7395202
H 0.4082644 -3.2467547 -59.3150915
H -0.9233073 -3.7726035 -60.3764866
H -1.265722 -2.8133331 -58.927048
N 2.0820627 -0.0851478 -64.4029156
H 2.4655832 0.475942 -63.6575331
C 2.9068083 -0.2770199 -65.5962123
H 2.3018658 -0.6952979 -66.4020926
C 4.0396154 -1.2643317 -65.292497
O 4.4307098 -1.4269569 -64.1332412

C 3.4495725 1.0814523 -66.061202
H 4.0958151 0.9525892 -66.9322466
H 4.0432311 1.5292122 -65.261646
S 2.0709612 2.1846743 -66.4876026
H 1.3003227 1.8921115 -65.4342484
N 4.6448883 -1.8495044 -66.3268338
H 4.3466771 -1.6176306 -67.261478
C 5.9932657 -2.3856146 -66.1595621
H 6.0491473 -2.8706147 -65.1886549
C 7.0088041 -1.2347704 -66.1249314
O 6.737833 -0.132612 -66.6086652
C 6.3073177 -3.4524623 -67.2163512
H 5.4957053 -4.1766962 -67.2517385
H 7.2191217 -3.9815768 -66.9375472
C 6.5230252 -2.8364934 -68.5773457
O 7.5678347 -2.280934 -68.8635157
N 5.5086591 -2.8222407 -69.403895
H 5.6807276 -2.3748843 -70.3032807
H 4.6772054 -3.3460722 -69.2191437
N 8.1801732 -1.5131329 -65.5618081
H 8.3470506 -2.4508453 -65.2320084
C 9.2452561 -0.5331003 -65.3748175
H 8.8589516 0.286887 -64.7647094
C 9.6957767 0.098359 -66.7072817
O 9.7789872 1.3222783 -66.8014925
C 10.3873618 -1.2029495 -64.5780293
H 11.2574185 -0.549599 -64.5671677
H 10.0590078 -1.2929866 -63.5414857
C 10.8177317 -2.5865022 -65.0490904
C 11.9181375 -2.7365552 -65.9169673
H 12.4819757 -1.8755661 -66.2390297
C 12.3053206 -4.0136478 -66.363435
H 13.1452796 -4.1187607 -67.0335377
C 11.597087 -5.1547648 -65.9319127
O 11.9592972 -6.3881262 -66.3699191
H 12.7274721 -6.3595775 -66.9421564
C 10.5129368 -5.0118722 -65.040923
H 9.9908157 -5.8930459 -64.6965091
C 10.1282528 -3.7322316 -64.5973171
H 9.308215 -3.6379075 -63.8983316
N 9.914116 -0.6953223 -67.7660937
H 9.7185 -1.6813804 -67.6780474
C 10.4258322 -0.1969978 -69.0526211
H 11.3531195 0.3334907 -68.8684677
C 9.4744436 0.8147866 -69.7068052
O 9.9050969 1.903903 -70.0918449

C 10.7555701 -1.3866897 -69.9704476
H 11.4799712 -2.0264374 -69.4643361
H 9.8468928 -1.968167 -70.1290859
C 11.319076 -0.9912917 -71.3499645
H 10.5886703 -0.3825456 -71.8825983
C 12.633967 -0.2142643 -71.2488822
H 13.3625122 -0.779567 -70.6682336
H 12.462991 0.7532524 -70.7772734
H 13.0319279 -0.0350601 -72.2476385
C 11.5735498 -2.2547509 -72.1719436
H 12.3160219 -2.8816896 -71.6783682
H 11.9344282 -1.9816748 -73.1636924
H 10.6437644 -2.8131246 -72.2827093
N 8.1721243 0.5219023 -69.7300654
H 7.8781855 -0.3931025 -69.4008782
C 7.1627271 1.4694778 -70.216607
H 7.465206 1.8258281 -71.2019721
C 7.0485684 2.7218684 -69.344085
O 6.7977963 3.7974389 -69.8845881
C 5.8037654 0.7721156 -70.3591403
H 5.0142317 1.5249066 -70.3314356
H 5.6424419 0.0910384 -69.5229777
C 5.6722935 0.0300481 -71.6647418
N 5.9532087 -1.3209948 -71.8725283
C 5.7499517 -1.545272 -73.178947
H 5.897551 -2.4993019 -73.6695688
N 5.3500598 -0.4176791 -73.790252
H 5.1471925 -0.3273254 -74.7758818
C 5.2980715 0.5887433 -72.851667
H 5.0341347 1.6258243 -73.0140616
N 7.3164396 2.6332608 -68.0360767
H 7.5599259 1.7314697 -67.6420576
C 7.2614763 3.7968527 -67.1509479
H 6.3620564 4.3393293 -67.4291629
C 8.4261241 4.7738874 -67.3880553
O 8.1695259 5.9696373 -67.5199989
C 7.0948289 3.3363918 -65.6891448
H 6.3288467 2.5601209 -65.6683109
H 8.0271337 2.8928056 -65.3390609
C 6.6681347 4.4549309 -64.7117129
H 7.5189438 5.109165 -64.5258907
C 5.4997377 5.3156355 -65.2074584
H 4.6757884 4.679795 -65.5306189
H 5.8301918 5.9439793 -66.0348046
H 5.1677863 5.9791257 -64.4099764
C 6.2145039 3.8387536 -63.3851094

H 5.3016056 3.2618588 -63.5260214
H 6.0445249 4.6237662 -62.6487135
H 6.9882306 3.1722436 -63.0041928
N 9.6480168 4.2850584 -67.6533181
H 9.7997559 3.2846604 -67.5630426
C 10.7262591 5.1507473 -68.1801541
H 10.8557976 5.9933419 -67.499146
C 10.3952471 5.6900104 -69.5631661
O 10.7418516 6.824877 -69.8713061
C 12.0605472 4.4240518 -68.3016877
H 12.1166467 3.8610945 -69.2350101
H 12.121121 3.7293563 -67.4803064
O 13.1491082 5.3247477 -68.1948499
H 13.269569 5.8510611 -69.0056855
N 9.6793356 4.9112594 -70.3797769
H 9.4739327 3.9631262 -70.084374
C 9.1529553 5.3651188 -71.6612905
H 9.9800387 5.6169965 -72.3253587
H 8.5721268 4.5597261 -72.1096179
C 8.2436502 6.5889815 -71.5206075
O 8.4964877 7.5881455 -72.1829673
N 7.271716 6.5802667 -70.6017955
H 7.1008963 5.7179054 -70.0934582
C 6.3753098 7.7248641 -70.3564864
H 5.913338 8.0175862 -71.2986071
C 7.1064366 8.967594 -69.8279953
O 6.7901523 10.0960769 -70.2162708
C 5.2706065 7.3106618 -69.3736671
H 5.7289851 6.9026424 -68.4718638
H 4.7203521 8.2063148 -69.0832355
C 4.2527546 6.3259864 -69.9274463
C 3.9569052 5.1377987 -69.2312677
H 4.470828 4.90886 -68.3126257
C 2.9868958 4.2463839 -69.7247382
H 2.7656118 3.3367646 -69.1875547
C 2.2932897 4.5473782 -70.9100298
H 1.5351606 3.8746796 -71.2849157
C 2.5749518 5.7375725 -71.6014384
H 2.0297721 5.995317 -72.5016579
C 3.5527959 6.6201892 -71.1146242
H 3.7383022 7.5375435 -71.6559592
N 8.1333138 8.7500029 -69.0098282
H 8.2811018 7.8069615 -68.6678179
C 9.07562 9.7716512 -68.5730746
H 8.5547353 10.5922679 -68.0912188
C 9.8605926 10.3853852 -69.7513712

O 9.7566054 11.5847949 -70.0065046
C 9.9597189 9.1076336 -67.5219712
H 10.8339721 9.7058895 -67.3759088
H 10.2895408 8.1279684 -67.8552867
S 9.0625811 8.9471298 -65.9503501
H 9.8351265 9.8474364 -65.3019252
N 10.4805271 9.5504548 -70.5898632
H 10.5030738 8.5688311 -70.3340113
C 11.2121939 9.9579073 -71.8004392
H 12.0155281 10.6371849 -71.5108146
C 10.3189662 10.6842638 -72.8220458
O 10.6706195 11.7496569 -73.3218699
C 11.8287769 8.7012708 -72.4308687
H 11.0787146 8.1829872 -73.0301747
H 12.1581597 8.0264929 -71.641054
O 12.9535704 9.016732 -73.2287619
H 13.2151123 8.2260534 -73.7115009
N 9.0917743 10.1931924 -73.0341901
H 8.8462209 9.3288767 -72.56349
C 8.0758771 10.7997598 -73.908273
H 8.5230395 10.9933282 -74.8827677
C 7.5601152 12.1572996 -73.4059811
O 6.9709603 12.9057875 -74.1813275
C 6.8980951 9.8177845 -74.0665242
H 6.5963806 9.4797408 -73.0746319
H 6.0469815 10.3468555 -74.4990079
C 7.2010356 8.596359 -74.9559661
H 8.1359891 8.1311667 -74.6495739
C 6.078985 7.5667695 -74.8172612
H 5.1265064 8.0041828 -75.1217947
H 6.002105 7.244648 -73.7790986
H 6.2918725 6.6974123 -75.4373065
C 7.3195826 8.9722824 -76.4354788
H 6.4050387 9.4644876 -76.7684964
H 7.4802548 8.0744735 -77.031481
H 8.1643764 9.6422728 -76.586479
N 7.8083199 12.5056056 -72.1428918
H 8.3267403 11.8644839 -71.5540116
C 7.4700463 13.8201725 -71.5844624
H 6.6249218 14.2248713 -72.140381
C 8.5965015 14.8537852 -71.7283394
O 8.4412966 15.9715581 -71.2569983
C 7.0096966 13.6702722 -70.1392581
H 6.7278152 14.646009 -69.7435907
H 7.8241777 13.2648047 -69.5378654
O 5.8818799 12.8084382 -70.0899695

H 6.1934362 11.8939971 -70.2199382
N 9.6527789 14.5194905 -72.4726668
H 9.6609405 13.583641 -72.8564542
C 10.8450282 15.3307806 -72.7823003
H 11.3215018 14.7681748 -73.5857787
C 11.9223128 15.3449434 -71.6844862
O 12.7208264 16.2703293 -71.5460031
C 10.4885248 16.6983789 -73.4123986
H 10.3052822 17.4216129 -72.6154718
H 9.5630553 16.5886969 -73.9810148
C 11.5483248 17.2610639 -74.3780375
O 12.3958398 16.4764423 -74.8659835
O 11.4371665 18.4657644 -74.7049311
N 11.9834183 14.26542 -70.8998863
H 11.310854 13.5258562 -71.0438426
C 13.0028759 14.0912496 -69.8714545
H 13.306885 15.0728525 -69.5156226
C 14.2829326 13.4339092 -70.3807713
O 14.2769208 12.3465484 -70.963321
C 12.4106606 13.3349922 -68.6923727
H 12.0504773 12.3665639 -69.0383334
H 13.215576 13.1470367 -67.9823596
C 11.3089208 14.0511588 -67.9372688
C 10.4143586 13.2895275 -67.1652494
H 10.4207411 12.2129036 -67.2452246
C 9.622269 13.9020251 -66.183757
H 9.0420531 13.2912381 -65.5123696
C 9.6896914 15.2939169 -66.0117228
O 9.0688287 15.8810816 -64.9659394
H 8.6517885 15.2197967 -64.4135997
C 10.476267 16.0688853 -66.8777947
H 10.5008894 17.1334103 -66.7732363
C 11.2869235 15.4568867 -67.8389113
H 11.9309688 16.084664 -68.4366982
N 15.413457 14.0666489 -70.0648519
H 15.3470006 14.8954175 -69.4865486
C 16.7466287 13.6072225 -70.4479379
H 16.6439033 13.0148765 -71.3578561
C 17.3845581 12.7089696 -69.3861672
O 17.0498046 12.785335 -68.2019845
C 17.6340751 14.8021464 -70.8021027
H 17.0888067 15.4656922 -71.4759625
H 18.5250258 14.4458703 -71.3186039
O 18.0387931 15.5263655 -69.6592734
H 17.3011331 15.5682376 -69.0191956
N 18.3085085 11.8526167 -69.839663

H 18.540374 11.8840546 -70.8182419
C 19.0346307 10.8940314 -69.0057114
H 19.4675616 10.1472293 -69.6713046
C 18.1121144 10.1118048 -68.0476025
O 18.3664568 10.0593789 -66.8463913
C 20.2060694 11.6112569 -68.3167241
H 19.8233584 12.3411857 -67.6012615
H 20.822286 12.1241624 -69.055329
H 20.8190484 10.8836954 -67.7848943
N 17.0082147 9.5547067 -68.5680509
H 16.8205954 9.6340163 -69.5538031
C 16.1258953 8.7216485 -67.7571872
H 16.0455573 9.2076642 -66.7871337
C 16.7726785 7.3640399 -67.4612841
O 17.037384 6.5613184 -68.3551552
C 14.6792575 8.6592675 -68.293561
H 14.2122908 9.6269136 -68.1101076
H 14.1203773 7.931987 -67.704949
C 14.479784 8.3334479 -69.7606206
O 14.1690558 7.2130685 -70.1375173
N 14.5042034 9.3331716 -70.6191546
H 14.1908225 9.1433714 -71.5603483
H 14.5384129 10.2922772 -70.2963576
N 17.0392665 7.1424113 -66.177116
H 16.830673 7.8928146 -65.5251381
C 17.5157217 5.8978433 -65.5954028
H 17.9177789 5.2616605 -66.384763
C 16.3357946 5.207524 -64.9288189
O 15.6159122 5.8225069 -64.1466295
C 18.6212937 6.1737034 -64.5672921
H 18.1711266 6.6156535 -63.6769974
H 19.0702786 5.2249355 -64.2716113
C 19.7093446 7.0913046 -65.0380035
C 19.7931824 8.4044157 -64.7311366
H 19.0720161 8.9404149 -64.124824
N 20.8504143 8.9783652 -65.4059943
H 20.9953366 9.9773354 -65.4322483
C 21.4912532 8.0630289 -66.2111008
C 22.5607112 8.1678796 -67.1126483
H 23.0547657 9.1163029 -67.2628505
C 22.976687 7.0234162 -67.8144749
H 23.7994497 7.0845023 -68.5131482
C 22.3164877 5.7977862 -67.6094385
H 22.6337933 4.9207785 -68.1562422
C 21.2368826 5.7069224 -66.707767
H 20.7248034 4.766601 -66.5741431

C 20.7952953 6.8365602 -65.9837824
N 16.1848563 3.912817 -65.176291
H 16.821799 3.469261 -65.8205527
C 15.0796393 3.1104436 -64.6775838
H 14.54308 3.6333506 -63.8833365
C 15.6247004 1.7985474 -64.1325112
O 16.0577958 0.9402323 -64.9020713
C 14.131694 2.8887033 -65.8487103
H 13.64995 3.8395378 -66.0623428
H 14.7289034 2.6128478 -66.7202805
C 13.0901348 1.8002141 -65.5654381
H 13.4487714 1.0489044 -64.8679001
C 11.8698551 2.4330043 -64.9143906
H 11.3898081 3.1481945 -65.575671
H 12.1699034 2.9421886 -63.9979327
H 11.1508884 1.6662489 -64.6483251
C 12.8433832 1.0456084 -66.8611406
H 12.3756177 1.6885177 -67.5980209
H 12.2143535 0.1981272 -66.6495824
H 13.7858171 0.6635186 -67.2549475
N 15.5022455 1.6123315 -62.8227831
H 15.0176951 2.3212374 -62.2749974
C 15.9819101 0.4156113 -62.1354336
H 16.5767406 -0.1688088 -62.8309248
C 14.7948239 -0.412656 -61.638592
O 14.0877462 0.0314062 -60.7298387
C 16.9228566 0.7713672 -60.9737509
H 16.3487296 1.1324943 -60.119959
C 17.7625409 -0.435787 -60.5611816
H 18.3692173 -0.7740933 -61.4018301
H 17.1180753 -1.2500821 -60.2345302
H 18.4224701 -0.1512396 -59.7425426
O 17.83844 1.7718527 -61.3585353
H 17.3314862 2.5714806 -61.5236629
N 14.5757734 -1.6381667 -62.1522123
C 13.7832369 -2.6243789 -61.4326093
H 12.8078798 -2.2156713 -61.1651937
C 14.5459745 -3.0172483 -60.1610187
O 15.6270337 -3.5983735 -60.2304277
C 13.5987399 -3.7952014 -62.405327
H 13.5382697 -4.7567764 -61.8952256
H 12.6985773 -3.6284725 -62.9974839
C 15.1494966 -2.2202696 -63.3610773
H 14.6833669 -1.7644417 -64.236083
H 16.2312348 -2.0961457 -63.4033807
C 14.823558 -3.7137458 -63.3158808

H 14.6132543 -4.1093829 -64.3096971
H 15.6551597 -4.2543635 -62.8618106
N 13.9662979 -2.746838 -58.9908767
H 13.0661721 -2.2754632 -59.0090904
C 14.5182136 -3.1102436 -57.6789229
H 15.4998097 -3.5591397 -57.8229977
C 13.7014467 -4.2044761 -56.9428997
O 13.6238671 -4.1604091 -55.7146881
C 14.714941 -1.81596 -56.8514598
H 13.7756341 -1.2643591 -56.8431172
H 14.9534945 -2.0737327 -55.819355
C 15.8229594 -0.873091 -57.3541501
H 15.6404761 -0.6146515 -58.3959849
C 15.8120489 0.4127376 -56.5303236
H 16.028931 0.1980959 -55.4826737
H 14.8309305 0.8833728 -56.593059
H 16.5554212 1.1109018 -56.9145674
C 17.2136708 -1.5018124 -57.2217831
H 17.3755269 -1.8389114 -56.1975999
H 17.9750578 -0.7631874 -57.470298
H 17.3151205 -2.3433035 -57.9050428
N 13.0948036 -5.214763 -57.6151661
C 12.2053006 -6.1721577 -56.9514439
H 11.3888186 -5.644905 -56.4617025
C 12.9249789 -7.0386965 -55.9075064
O 12.3195788 -7.4085346 -54.9111168
C 11.6162382 -7.0363963 -58.0682371
H 11.3764525 -8.0460584 -57.7312578
H 10.7270492 -6.5549761 -58.4702904
C 13.2765494 -5.6211828 -59.0043124
H 12.7053484 -4.9577645 -59.6524598
H 14.33053 -5.6213029 -59.2835882
C 12.7120005 -7.0340446 -59.1282169
H 12.3119336 -7.2235629 -60.1252091
H 13.4796364 -7.7662537 -58.8743209
N 14.2193922 -7.3309605 -56.0875591
H 14.6758111 -6.9951267 -56.9187628
C 15.0103271 -8.0990672 -55.112049
H 14.434692 -8.9910839 -54.8655987
C 15.2070223 -7.332927 -53.7964256
O 15.0766997 -7.9116657 -52.717904
C 16.3497863 -8.552893 -55.7413196
H 16.1067111 -9.1238207 -56.6393262
C 17.2457079 -7.3784555 -56.1790683
H 17.6104389 -6.833258 -55.3064419
H 16.7096291 -6.695959 -56.8363059

H 18.1109972 -7.7622999 -56.7213065
C 17.1758962 -9.4774166 -54.8192359
H 18.0864798 -9.7670527 -55.3455807
H 17.4716894 -8.935527 -53.9193684
C 16.4479887 -10.7630839 -54.4053729
H 16.0929498 -11.2919093 -55.2902479
H 15.605773 -10.5327737 -53.7532482
H 17.1389073 -11.4070984 -53.8607548
N 15.3935547 -6.0141343 -53.87863
H 15.4044782 -5.57695 -54.785056
C 15.4989416 -5.1573755 -52.7005961
H 16.1260958 -5.6549509 -51.9609007
C 14.1275042 -4.979588 -52.0493859
O 14.0041547 -5.2410058 -50.8625342
C 16.1529791 -3.8128398 -53.0612849
H 15.5453294 -3.3096024 -53.8136461
H 16.1749842 -3.1849001 -52.1697456
C 17.5888162 -3.9565456 -53.5969701
H 17.9604463 -2.9636573 -53.8583359
H 17.5825816 -4.5500643 -54.513358
C 18.5239075 -4.5941443 -52.560969
O 18.5957494 -5.8428049 -52.4662396
O 19.14147 -3.8633975 -51.7551244
N 13.0718701 -4.7526557 -52.834066
H 13.246795 -4.5814834 -53.8192455
C 11.6674641 -4.7573474 -52.3915481
H 11.5110988 -3.9424476 -51.6838844
C 11.2638044 -6.055375 -51.6705936
O 10.7000723 -5.9916155 -50.5815018
C 10.8225721 -4.4658085 -53.6373247
H 11.2703018 -4.9725609 -54.4847155
H 10.8980451 -3.3996945 -53.8162933
C 9.3400869 -4.8422281 -53.6032018
O 8.5960027 -4.4336681 -52.6834481
O 8.8625046 -5.4577929 -54.5871172
N 11.6917214 -7.2238873 -52.1577427
H 12.1378338 -7.2256039 -53.0701622
C 11.4803921 -8.5084706 -51.4804795
H 10.4192842 -8.5902366 -51.2439924
C 12.2581264 -8.616735 -50.1576347
O 11.7173718 -9.1125472 -49.1729519
C 11.8590517 -9.6523727 -52.4351888
H 12.9441046 -9.6885066 -52.5437256
H 11.4310558 -9.4628766 -53.4176041
C 11.3583046 -11.0199242 -51.9562242
H 11.7482812 -11.2164564 -50.9570363

H 11.7634607 -11.7769659 -52.6281464
S 9.5513028 -11.2041661 -51.9296248
C 9.4280423 -12.9497713 -51.463499
H 8.3802362 -13.2482151 -51.4521366
H 9.8585708 -13.0948903 -50.4720088
H 9.9653893 -13.5614913 -52.1881289
N 13.4992124 -8.1155487 -50.1013757
H 13.8751445 -7.7085546 -50.9531837
C 14.3462607 -8.0992723 -48.8917458
H 14.3261654 -9.0880359 -48.4302966
C 13.8414349 -7.109273 -47.8321094
O 13.8696885 -7.4088854 -46.6438106
C 15.7803139 -7.7771225 -49.3386629
H 16.0728206 -8.4953921 -50.1072518
H 15.78842 -6.7853287 -49.7877521
C 16.8146607 -7.8333564 -48.2019743
H 16.5459291 -7.1366515 -47.4067893
H 16.8280532 -8.8437172 -47.7908348
C 18.2187971 -7.4873246 -48.7193819
H 18.4184365 -8.0829343 -49.6128579
H 18.9536731 -7.7499724 -47.9570584
C 18.3487833 -5.992413 -49.0458139
H 18.4319706 -5.4217815 -48.1165061
H 17.4451803 -5.6561907 -49.5619324
N 19.5031294 -5.7293883 -49.9309239
H 20.3954323 -5.9966135 -49.5549232
H 19.5174901 -4.749167 -50.2346865
H 19.3464276 -6.1609146 -50.848426
N 13.3322727 -5.9630881 -48.2710839
H 13.3916351 -5.7966786 -49.2700395
C 12.6752334 -4.9259791 -47.4652083
H 13.3066394 -4.639705 -46.6204001
C 11.3514425 -5.4659331 -46.907204
O 11.0801258 -5.3692947 -45.708615
C 12.463281 -3.6936637 -48.3766892
H 12.0638674 -4.0566426 -49.3241162
C 11.3939495 -2.735481 -47.8251092
H 11.5699158 -2.5482408 -46.7698325
H 10.4027027 -3.1721763 -47.9439616
H 11.4167651 -1.795219 -48.3641339
C 13.8090055 -3.0011842 -48.7056933
H 14.1943818 -2.5034339 -47.8276267
H 14.5553025 -3.7476582 -48.9678682
C 13.7415405 -1.9940272 -49.8643963
H 13.1388901 -1.128049 -49.5957665
H 13.3172396 -2.4665957 -50.7493734

H 14.7489847 -1.6485579 -50.0979748
N 10.5569209 -6.1251239 -47.7575478
H 10.8058184 -6.1502801 -48.7417588
C 9.3406062 -6.8155937 -47.3499945
H 8.687436 -6.0980664 -46.8515223
C 9.6451199 -7.9320667 -46.3426887
O 8.9789309 -7.9857565 -45.3181334
C 8.6090851 -7.3439192 -48.5900398
H 9.2347282 -8.0646011 -49.1166765
H 8.3743305 -6.5167723 -49.2610325
H 7.6825766 -7.8315087 -48.286617
N 10.6967849 -8.7327052 -46.5459848
H 11.1877227 -8.6812436 -47.4322827
C 11.1410652 -9.7413254 -45.5766499
H 10.3095113 -10.4188421 -45.3765601
C 11.5417736 -9.1181235 -44.2316211
O 11.0027769 -9.5243077 -43.2065835
C 12.2879134 -10.5821685 -46.1574739
H 13.1383181 -9.9410307 -46.3900585
H 11.9539012 -11.054893 -47.0827182
C 12.7241101 -11.6616207 -45.1622198
O 13.5224159 -11.3337599 -44.252894
O 12.2018212 -12.793503 -45.265959
N 12.3829782 -8.0769071 -44.2118859
H 12.8033958 -7.7736289 -45.0842588
C 12.8418208 -7.4756884 -42.9545116
H 13.20788 -8.2795145 -42.315361
C 11.7105569 -6.7843906 -42.179033
O 11.5105007 -7.0762136 -40.995856
C 14.0107534 -6.5158331 -43.2086705
H 14.8199205 -7.0620265 -43.6953675
H 13.6867322 -5.7238326 -43.8863098
C 14.5352889 -5.889822 -41.9505213
C 15.3513898 -6.4920806 -41.0560262
H 15.7532765 -7.4912347 -41.1718495
N 15.5335855 -5.6654725 -39.9629168
H 16.0897329 -5.9158626 -39.160673
C 14.8148192 -4.49376 -40.0872147
C 14.6321954 -3.3866221 -39.242576
H 15.1032791 -3.3500841 -38.2735638
C 13.819259 -2.3236695 -39.676404
H 13.6551516 -1.4597462 -39.0434416
C 13.2231146 -2.3758491 -40.9490175
H 12.61592 -1.5480294 -41.2963996
C 13.413713 -3.4925454 -41.785488
H 12.9690569 -3.4997165 -42.7718436

C 14.1985453 -4.5894945 -41.3712467
N 10.9043925 -5.9527161 -42.8492866
H 11.1134384 -5.7630804 -43.8240366
C 9.7438538 -5.2921988 -42.2212211
H 10.0844229 -4.773692 -41.3238945
C 8.6828352 -6.2978319 -41.7797415
O 8.1475363 -6.1684389 -40.6765001
C 9.0796136 -4.2487583 -43.1304959
H 8.177974 -3.8895772 -42.6371761
C 9.9720763 -3.0467225 -43.4104666
H 10.8669468 -3.3593763 -43.9445606
H 10.2661708 -2.5763767 -42.4714854
H 9.4314004 -2.3171273 -44.0117579
O 8.7181591 -4.8066588 -44.3726664
H 9.5496823 -4.9479732 -44.8577988
N 8.4358032 -7.3581704 -42.5633335
H 8.8963046 -7.4314515 -43.466883
C 7.5723311 -8.4495715 -42.1268535
H 6.6350766 -8.0155664 -41.7795424
C 8.1700975 -9.1517994 -40.9247922
O 7.4758181 -9.174459 -39.9330543
C 7.2448461 -9.4620492 -43.2330206
H 8.1639771 -9.7685181 -43.7320877
H 6.8107539 -10.3548548 -42.779457
C 6.239774 -8.9211213 -44.2597927
H 6.2060887 -9.6067299 -45.1067549
H 6.5649357 -7.9487869 -44.6272582
C 4.8301142 -8.7907822 -43.6962677
O 4.0185217 -9.6936336 -43.7800733
N 4.4373901 -7.6329512 -43.2092787
H 3.4549186 -7.5555601 -42.9669857
H 5.0762198 -6.8618807 -43.140931
N 9.4273277 -9.6006942 -40.9284614
H 9.9686587 -9.4839231 -41.780828
C 10.0214635 -10.412134 -39.8567427
H 9.3084547 -11.1990181 -39.609992
C 10.253828 -9.6601678 -38.5291229
O 10.2250454 -10.2893742 -37.4666527
C 11.2944858 -11.0964365 -40.3945858
H 11.8618845 -11.5134649 -39.5643313
H 11.9244235 -10.3674132 -40.904203
C 10.9694015 -12.2521054 -41.3360884
O 10.0165345 -12.9890257 -41.1245311
N 11.7728694 -12.4937022 -42.343769
H 11.4995019 -13.1733068 -43.0362986
H 12.499172 -11.8467642 -42.6449554

N 10.2929531 -8.3240194 -38.537167
H 10.3624872 -7.8437779 -39.4304161
C 10.2075646 -7.5261957 -37.311539
H 10.9370343 -7.9083139 -36.5955343
C 8.8173012 -7.6078404 -36.6366541
O 8.7224682 -7.6976754 -35.4119872
C 10.5911494 -6.0827475 -37.6571958
H 9.8848154 -5.6694188 -38.3793169
H 11.5923603 -6.0574983 -38.0909011
H 10.5793001 -5.471428 -36.7549126
N 7.7281362 -7.648853 -37.4093658
H 7.8464977 -7.6620426 -38.4175533
C 6.3615845 -7.7065981 -36.8837774
H 6.2525433 -6.8732437 -36.1891368
C 5.9991926 -8.9928891 -36.0905116
O 5.3798625 -8.8602907 -35.0312186
C 5.3875801 -7.4605357 -38.0417105
H 5.317507 -8.3466364 -38.6717451
H 5.7360643 -6.6360806 -38.6607618
H 4.3964641 -7.2320804 -37.651936
N 6.3537059 -10.227742 -36.5201682
C 6.0921003 -11.4546801 -35.7992746
H 5.0595046 -11.4728627 -35.4552056
C 7.0141917 -11.5868196 -34.5825291
O 6.5442037 -12.022288 -33.5387212
C 6.2944393 -12.5878507 -36.8162142
H 6.5796654 -13.5239315 -36.3340225
H 5.3790469 -12.7278175 -37.3944718
C 6.9710996 -10.6108314 -37.7595143
H 6.2311127 -10.4906961 -38.55036
H 7.8621115 -10.0271148 -37.9027458
C 7.3852351 -12.0701274 -37.7139111
H 7.3632158 -12.5333561 -38.7012058
H 8.3603036 -12.1870356 -37.2408137
N 8.2600128 -11.0993172 -34.6473682
H 8.5974122 -10.7351963 -35.529587
C 9.1638845 -11.0324667 -33.493428
H 9.2559856 -12.0264496 -33.0531509
C 8.6246533 -10.1030979 -32.3918366
O 8.5898426 -10.4804776 -31.2234502
C 10.5463395 -10.5906214 -33.9929249
H 10.9400176 -11.3588206 -34.6604347
H 10.4349591 -9.6795107 -34.5821898
C 11.5645544 -10.3336599 -32.8982995
C 12.1676299 -11.4139774 -32.226674
H 11.9007571 -12.4282594 -32.4853161

C 13.115578 -11.1764532 -31.2147571
H 13.5750579 -12.007349 -30.6985902
C 13.464218 -9.8578058 -30.873536
H 14.194 -9.6750549 -30.0974929
C 12.8624226 -8.7769348 -31.541472
H 13.1324783 -7.7636193 -31.281157
C 11.9107974 -9.0133361 -32.5496745
H 11.4482941 -8.1777601 -33.0568941
N 8.0603085 -8.9495652 -32.7631566
H 8.1434311 -8.665565 -33.7337643
C 7.4168677 -8.0211198 -31.8271501
H 7.9214617 -8.1082216 -30.8649975
C 5.9315139 -8.326438 -31.5456683
O 5.2535744 -7.4801421 -30.961817
C 7.5941536 -6.574922 -32.324633
H 7.124499 -6.4941971 -33.3064549
H 7.0538484 -5.9068789 -31.6550215
C 9.0136829 -6.0386909 -32.3987931
C 9.4463363 -5.359517 -33.5561945
H 8.7912065 -5.2787379 -34.4129759
C 10.7433545 -4.8150995 -33.6203321
H 11.075635 -4.3075551 -34.5142454
C 11.6148391 -4.9453172 -32.5180674
O 12.8717441 -4.435554 -32.5709646
H 13.0345853 -3.9454995 -33.3800472
C 11.174962 -5.6029107 -31.3511048
H 11.8453005 -5.6829437 -30.507972
C 9.8769556 -6.144496 -31.2893049
H 9.5563376 -6.6474406 -30.3872537
N 5.3871766 -9.481238 -31.956426
H 6.0033601 -10.1538206 -32.4006634
C 3.9274016 -9.6951489 -32.0875758
H 3.5788743 -9.0167038 -32.8656313
C 3.101727 -9.3795924 -30.8315989
O 1.9946272 -8.8568 -30.9597617
C 3.6184426 -11.1199467 -32.5819641
H 4.1373467 -11.2775613 -33.5237867
C 4.0262775 -12.229422 -31.6076246
H 3.4924745 -12.148523 -30.6645446
H 5.0959923 -12.1726016 -31.405786
H 3.820187 -13.1996915 -32.0601955
O 2.2341245 -11.2757929 -32.8256162
H 2.1031661 -11.0808149 -33.7675107
N 3.6312656 -9.6287895 -29.6257628
H 4.5649006 -10.0097299 -29.5872965
C 2.9405287 -9.3177676 -28.3687039

H 1.9221669 -9.6969241 -28.445873
C 2.8276563 -7.8017421 -28.1507249
O 1.7167741 -7.2833488 -28.1555535
C 3.6362754 -10.0368499 -27.1973246
H 3.6353806 -11.1098669 -27.3948457
H 4.6745595 -9.7054275 -27.1474315
C 2.978396 -9.7839054 -25.8233671
H 2.9966347 -8.7178673 -25.6005677
C 1.5310448 -10.2812004 -25.7697653
H 1.4817257 -11.3377576 -26.0293787
H 0.9096754 -9.6938931 -26.4446131
H 1.1425322 -10.1385847 -24.7604909
C 3.7720642 -10.514862 -24.7416685
H 3.7563083 -11.5903824 -24.9153329
H 3.3368301 -10.2987848 -23.7649057
H 4.8026104 -10.1599394 -24.7388168
N 3.9616382 -7.0915666 -28.1298216
H 4.8305866 -7.5964343 -28.1841824
C 4.0327294 -5.6286734 -27.9990707
H 3.5475118 -5.3373421 -27.066531
C 3.3223149 -4.9052738 -29.1588863
O 2.7887892 -3.8112358 -29.0000078
C 5.522688 -5.2420034 -27.9073454
H 5.9417744 -5.7125461 -27.0159418
H 6.0387796 -5.6508386 -28.77724
C 5.8148372 -3.7290084 -27.839953
H 5.4302001 -3.2507583 -28.740477
C 5.2052008 -3.0369301 -26.6265279
H 5.5270028 -3.5308572 -25.7079944
H 4.1165442 -3.0902357 -26.6670599
H 5.5002705 -1.9890255 -26.5938106
C 7.3263404 -3.5054407 -27.8182893
H 7.7487717 -3.9367071 -26.9075049
H 7.5460048 -2.4366408 -27.8259105
H 7.7977514 -3.9704279 -28.681937
N 3.3240981 -5.4922525 -30.3565642
H 3.8200164 -6.3736162 -30.4481514
C 2.6217824 -4.9657189 -31.5266407
H 2.8835245 -3.9148142 -31.6417372
C 1.0943839 -5.029999 -31.3514762
O 0.4108227 -4.0473604 -31.6319395
C 3.127867 -5.7370606 -32.7582961
H 4.209855 -5.607543 -32.8206349
H 2.9292394 -6.7975108 -32.6056175
C 2.5111026 -5.3318787 -34.1077532
H 1.4532277 -5.5947615 -34.1197899

C 2.6686143 -3.8435188 -34.4097948
H 3.7203921 -3.5715168 -34.3494023
H 2.0853599 -3.2554452 -33.704076
H 2.2981698 -3.6320755 -35.4139741
C 3.226156 -6.095398 -35.2194507
H 4.2560045 -5.7514367 -35.3113525
H 2.7083162 -5.937219 -36.1650524
H 3.2404669 -7.1588567 -34.9842874
N 0.5617721 -6.1321914 -30.8100106
H 1.1854655 -6.8955181 -30.565335
C -0.8774119 -6.2932057 -30.5294087
H -1.4450707 -5.8133694 -31.3273322
C -1.3387857 -5.5989339 -29.2472027
O -2.4972833 -5.2107496 -29.1799714
C -1.2259879 -7.7857952 -30.5059816
H -0.5395652 -8.3161481 -29.8425807
H -2.2423577 -7.9080194 -30.1304602
C -1.1550235 -8.3653732 -31.9235204
H -0.1595575 -8.2283015 -32.3457716
H -1.8592111 -7.825137 -32.5503861
C -1.517416 -9.8499764 -31.9535904
H -1.7789138 -10.1101266 -32.9786297
H -2.3926915 -10.0247935 -31.3275004
N -0.3815104 -10.6888054 -31.5472774
H 0.4686067 -10.5891001 -32.0922565
C -0.2819976 -11.5284697 -30.5397689
N 0.7987728 -12.2399241 -30.4453776
H 0.9213052 -12.9191248 -29.7195572
H 1.4926744 -12.1243647 -31.1788985
N -1.2140364 -11.691179 -29.6413828
H -2.0524106 -11.1461476 -29.7056909
H -1.0837421 -12.3425586 -28.8909044
N -0.4359914 -5.4137744 -28.291648
H 0.4565919 -5.878876 -28.4106519
C -0.6121594 -4.6637386 -27.0405899
H -1.5484464 -4.9448234 -26.5574101
C -0.6419177 -3.1442925 -27.2699944
O -1.4719593 -2.454789 -26.6849815
C 0.5604857 -5.0831334 -26.1452238
H 0.4619065 -6.1502756 -25.9371324
H 1.4675594 -4.941078 -26.7285774
C 0.7664583 -4.3591921 -24.8161269
H -0.1072872 -4.5150072 -24.1788573
H 0.8875321 -3.2879945 -24.9917974
C 2.035639 -4.9226683 -24.1581527
O 1.8929473 -5.6917077 -23.1831504

O 3.1384206 -4.6239782 -24.6732221
N 0.1917402 -2.6286023 -28.1822229
H 0.9142711 -3.2259607 -28.5641261
C 0.1832659 -1.2150978 -28.5658876
H 0.0037262 -0.6137299 -27.6722889
C -0.9246664 -0.8558517 -29.5768898
O -1.3427934 0.2986789 -29.6289793
C 1.5712314 -0.8568396 -29.111109
H 1.7755088 -1.4407739 -30.0082925
H 2.3275326 -1.0683264 -28.3535512
H 1.5991124 0.2052893 -29.3567835
N -1.3690744 -1.8151607 -30.399589
H -0.9570034 -2.7323259 -30.3107115
C -2.4122034 -1.6197109 -31.4130676
H -2.8401389 -0.6237023 -31.2850044
C -3.5838278 -2.5891446 -31.1989809
O -4.4984085 -2.2893246 -30.4387788
C -1.8158924 -1.6685056 -32.833723
H -1.3145361 -2.624073 -32.987596
H -2.63415 -1.6141187 -33.553035
C -0.8409829 -0.5505884 -33.1512774
C -1.3096435 0.649862 -33.7150379
H -2.3647469 0.7833783 -33.9051926
C 0.531843 -0.7025696 -32.8802269
H 0.8864084 -1.6149005 -32.4260483
C -0.4110316 1.6879453 -34.01348
H -0.7806818 2.6123909 -34.4308109
C 1.4300197 0.3388147 -33.1757978
H 2.4760809 0.2347951 -32.9481857
C 0.9602995 1.5313944 -33.7512922
H 1.6466328 2.3356076 -33.9776088
N -3.6205628 -3.7132496 -31.9236746
H -2.8479066 -3.9034345 -32.5436994
C -4.6046085 -4.7861899 -31.7255437
H -4.6477881 -5.0219769 -30.6606922
C -4.2176418 -6.0541322 -32.4919207
O -3.4466607 -6.006089 -33.4557384
C -6.0082382 -4.3507814 -32.1811528
H -6.3304691 -3.4699273 -31.624698
H -6.7175425 -5.152142 -31.9705056
O -6.0325689 -4.0692292 -33.5679786
H -5.899994 -3.1163384 -33.6518697
N -4.8741413 -7.1737827 -32.163381
H -5.4716506 -7.1487112 -31.3494907
C -4.8665038 -8.4037354 -32.9751168
H -3.8421984 -8.7622554 -33.024553

C -5.296619 -8.1459118 -34.4291164
O -4.6251069 -8.5946963 -35.3565651
C -5.7191938 -9.4992887 -32.2851475
H -5.2617698 -9.6962688 -31.3135578
C -7.1749464 -9.0643969 -32.0189038
H -7.7180113 -8.9252308 -32.9539572
H -7.2157387 -8.1483551 -31.4309694
H -7.6857446 -9.8419113 -31.4478146
C -5.7591062 -10.8389807 -33.0521625
H -6.3486961 -11.5529072 -32.4750014
H -6.2623358 -10.6958331 -34.0075932
C -4.3868889 -11.4732058 -33.3065014
H -3.86465 -11.6184485 -32.3614838
H -3.7929475 -10.8453952 -33.9693974
H -4.5241808 -12.4437534 -33.7840225
N -6.3197801 -7.3081212 -34.6459337
H -6.7867577 -6.9174776 -33.8407924
C -6.8108968 -6.9429317 -35.9822531
H -6.9295796 -7.8697976 -36.5438468
C -5.8010042 -6.0946841 -36.7703672
O -5.7534856 -6.2049544 -37.9993011
C -8.1929232 -6.2678168 -35.828368
H -8.8936588 -6.9935093 -35.4113768
H -8.1035006 -5.4462128 -35.1147961
C -8.795135 -5.6932521 -37.1238768
H -9.7559562 -5.2332397 -36.8894435
H -8.1411857 -4.8942535 -37.4703831
C -9.0162358 -6.7421972 -38.2346595
H -9.9843958 -7.2211919 -38.076221
H -8.2596339 -7.5233973 -38.1830747
C -8.9707821 -6.1540308 -39.6519625
H -9.7832162 -5.4324325 -39.7803459
H -9.1312743 -6.9749907 -40.3575952
N -7.6582374 -5.5172426 -39.9201159
H -7.5238449 -4.6979612 -39.3466649
H -7.5232324 -5.260878 -40.8933727
H -6.8930147 -6.1433565 -39.6825251
N -4.9831557 -5.2914209 -36.0868715
H -5.1114733 -5.2316311 -35.0833861
C -3.8621657 -4.5557308 -36.6755995
H -4.2125805 -3.9476157 -37.5101316
H -3.4303927 -3.8987074 -35.9202956
C -2.7652385 -5.4947951 -37.177779
O -2.4814048 -5.5085537 -38.373279
N -2.2677174 -6.3806718 -36.3070245
H -2.5945535 -6.342489 -35.3479413

C -1.2607822 -7.3958332 -36.6512525
H -0.3445124 -6.8925585 -36.961776
C -1.6939054 -8.2984128 -37.8176158
O -0.9311845 -8.4717807 -38.7673839
C -0.9587692 -8.2239316 -35.3945171
H -0.5126998 -7.5591821 -34.6545479
H -1.9003645 -8.5918536 -34.9852958
C -0.0205586 -9.403772 -35.5957312
C -0.476939 -10.7237244 -35.4080804
H -1.509788 -10.9114615 -35.1526382
C 0.4018223 -11.8087914 -35.5828742
H 0.0411055 -12.8215519 -35.4605813
C 1.7396798 -11.5814567 -35.9510346
H 2.4073452 -12.4177367 -36.1126058
C 2.197147 -10.2669613 -36.1448257
H 3.2167878 -10.0866761 -36.4561706
C 1.3158525 -9.1847377 -35.975059
H 1.6594669 -8.1812815 -36.1674815
N -2.9508894 -8.7602365 -37.8162996
H -3.5180262 -8.6013591 -36.9897401
C -3.5164419 -9.5705426 -38.9055008
H -2.8830213 -10.4489155 -39.0412821
C -3.4884375 -8.8191223 -40.2441416
O -3.0854456 -9.4032043 -41.2444796
C -4.9353343 -10.0606912 -38.5229828
H -5.4930771 -9.2156787 -38.1147458
C -5.7199256 -10.6008875 -39.7362459
H -5.1748012 -11.428586 -40.1934969
H -5.8704642 -9.8203191 -40.4808494
H -6.7059161 -10.950001 -39.4339102
C -4.8187313 -11.1576382 -37.4374046
H -4.3660146 -12.0503218 -37.8713733
H -4.1598068 -10.814504 -36.6407699
C -6.1553173 -11.5460725 -36.7923586
H -6.7955629 -12.0580817 -37.5094256
H -6.6616767 -10.6550823 -36.4198857
H -5.9674939 -12.2254155 -35.9610021
N -3.8323925 -7.5248185 -40.2879394
H -4.0656676 -7.0556824 -39.4218744
C -3.7691065 -6.745843 -41.5391746
H -4.3005425 -7.2994218 -42.3139513
C -2.3393446 -6.5519942 -42.0526116
O -2.1145754 -6.6247537 -43.2621331
C -4.4332741 -5.3718712 -41.3703427
H -3.8537605 -4.6109815 -41.8970195
H -4.4733346 -5.1006908 -40.3138927

O -5.7412933 -5.3987764 -41.9212897
H -5.6390313 -5.3843889 -42.8827614
N -1.3861787 -6.3126124 -41.1495095
H -1.6547347 -6.2826085 -40.1714205
C 0.0158965 -6.0425797 -41.481886
H 0.0577829 -5.3112942 -42.2894989
C 0.740751 -7.3012983 -41.9896076
O 1.4825599 -7.2298511 -42.9719905
C 0.6993255 -5.4537121 -40.2332656
H 0.5759614 -6.161702 -39.4119538
H 1.768021 -5.3583632 -40.4270006
C 0.1460188 -4.0790425 -39.7977291
H -0.9410754 -4.0968931 -39.8068985
C 0.5881962 -3.7787531 -38.3651539
H 1.6688825 -3.8436922 -38.2817429
H 0.1358835 -4.4987693 -37.6855946
H 0.2728934 -2.7773283 -38.0815491
C 0.5838749 -2.9467471 -40.7300837
H 1.6679734 -2.8669288 -40.751094
H 0.1714852 -2.0035095 -40.3724266
H 0.2150466 -3.1247907 -41.7383577
N 0.4550266 -8.4611916 -41.3890336
H -0.1379686 -8.4416052 -40.564342
C 0.9646168 -9.7595951 -41.8364496
H 2.0196674 -9.6433663 -42.0892642
C 0.2485841 -10.2596498 -43.1035624
O 0.8987943 -10.7616169 -44.0158858
C 0.8455888 -10.7511466 -40.6638834
H 1.3523783 -10.326376 -39.7955445
H -0.210257 -10.8640917 -40.4110157
C 1.4357562 -12.1477194 -40.9424942
H 0.9147976 -12.6038002 -41.7839374
C 2.9345315 -12.1074372 -41.2461209
H 3.4682867 -11.5756565 -40.4587843
H 3.1048157 -11.6059846 -42.1990644
H 3.3258448 -13.1209115 -41.3293987
C 1.228386 -13.036948 -39.7157139
H 1.7534838 -12.6171902 -38.8578697
H 1.6078131 -14.0384338 -39.9180822
H 0.1647686 -13.1057515 -39.4881301
N -1.0698244 -10.0583188 -43.2209953
H -1.5784126 -9.6705319 -42.4337335
C -1.8288138 -10.4543792 -44.4133578
H -1.6406428 -11.512455 -44.6026739
C -1.3632127 -9.6903915 -45.6534104
O -1.0336879 -10.3147171 -46.6612399

C -3.3368109 -10.2801862 -44.1613091
H -3.5384081 -9.2595103 -43.8397539
H -3.6148275 -10.9493552 -43.3461491
C -4.2354917 -10.6192243 -45.3629775
H -5.2387681 -10.8120393 -44.981554
H -3.8764807 -11.5469091 -45.8110439
S -4.390968 -9.3657168 -46.6781462
C -5.1799099 -8.0079527 -45.7729399
H -5.4383667 -7.2116522 -46.4701612
H -4.4931662 -7.6138861 -45.0242914
H -6.0870682 -8.3705426 -45.2881865
N -1.2836702 -8.3547994 -45.597801
H -1.5351508 -7.8696527 -44.7427603
C -0.9788531 -7.5879528 -46.8043397
H -1.457325 -8.1034868 -47.6360249
C 0.521404 -7.5756896 -47.1451478
O 0.8637811 -7.5102657 -48.3264741
C -1.6430715 -6.2032923 -46.748782
H -1.1355764 -5.5871202 -46.0089988
H -2.6802938 -6.3144597 -46.4385527
C -1.6394516 -5.5151375 -48.1094585
N -0.9709235 -4.389742 -48.2157069
H -0.4685428 -4.0199304 -47.4270163
H -0.9501478 -3.9346266 -49.1103779
O -2.2486604 -5.9590541 -49.0785727
N 1.416904 -7.7516498 -46.1678263
H 1.0980991 -7.8154289 -45.2078644
C 2.8375119 -7.9866019 -46.4363406
H 3.1969886 -7.2710589 -47.1758242
H 3.3911814 -7.8211393 -45.5225839
C 3.1279278 -9.4043741 -46.945777
O 3.8298141 -9.5493483 -47.9453507
N 2.447086 -10.4225332 -46.4101552
H 1.9315207 -10.2624063 -45.5490469
C 2.4854058 -11.7979805 -46.9139969
H 3.5152832 -12.1562071 -46.9087821
H 1.8931677 -12.4278665 -46.2504679
C 1.9239234 -11.9295401 -48.3328251
O 2.5932635 -12.4826701 -49.2022689
N 0.7929469 -11.2777152 -48.628854
H 0.267872 -10.8632915 -47.8641561
C 0.2683398 -11.1228987 -49.9938683
H 0.0573556 -12.1067264 -50.4128921
C 1.2826057 -10.4351154 -50.919821
O 1.4660857 -10.8427349 -52.0678355
C -1.03995 -10.3126457 -49.9511539

H -1.8060414 -10.9034882 -49.4476874
H -0.8743164 -9.4136616 -49.3588149
C -1.5543657 -9.8836418 -51.2950987
C -1.1187229 -8.7991881 -51.9758444
H -0.3563124 -8.1147227 -51.6133052
N -1.6791194 -8.7882528 -53.2363242
H -1.3736528 -8.1625098 -53.9676252
C -2.5438482 -9.8457334 -53.4186151
C -3.3411695 -10.2526051 -54.4992947
H -3.3476281 -9.6931163 -55.422582
C -4.1539985 -11.3894695 -54.358604
H -4.801853 -11.6994674 -55.1695271
C -4.1314936 -12.1172815 -53.1550939
H -4.7589197 -12.9925633 -53.044334
C -3.3101688 -11.7105559 -52.0842815
H -3.3046579 -12.2804389 -51.1652787
C -2.499787 -10.5592805 -52.1831049
N 1.96428 -9.3993704 -50.4253499
H 1.7809878 -9.1156849 -49.4709382
C 2.9636726 -8.654149 -51.2011413
H 2.5137098 -8.3680451 -52.1510126
C 4.1751778 -9.5252683 -51.5380323
O 4.584636 -9.5468988 -52.6991712
C 3.3562456 -7.3613529 -50.4714756
H 3.7076416 -7.5770001 -49.4645344
C 4.4220107 -6.5580398 -51.2039862
H 4.0784879 -6.3762013 -52.2179027
H 5.3645424 -7.1059384 -51.2128172
H 4.574357 -5.6062331 -50.6915057
O 2.2203371 -6.5288334 -50.4111686
H 1.6945377 -6.8265429 -49.6442254
N 4.6517677 -10.3483214 -50.5991615
H 4.2697926 -10.2894075 -49.6593257
C 5.6772822 -11.3580867 -50.8568759
H 6.5451977 -10.8700353 -51.3041686
C 5.1923812 -12.4341222 -51.8490049
O 5.8968642 -12.7354913 -52.8110289
C 6.1067911 -11.9647842 -49.5145566
H 5.2580115 -12.4435631 -49.0255728
H 6.499734 -11.1841373 -48.8617151
H 6.8863492 -12.7084471 -49.6781218
N 3.9534366 -12.9249302 -51.7086818
H 3.4267844 -12.6496469 -50.8836684
C 3.334672 -13.8997941 -52.6291921
H 3.9737587 -14.7791788 -52.6745089
C 3.2311635 -13.3581777 -54.0601941

O 3.501972 -14.0879971 -55.0132972
C 1.9611062 -14.3607064 -52.0920771
H 1.4005589 -13.4924383 -51.7508018
C 1.0987965 -15.094003 -53.1308964
H 1.6582642 -15.9270397 -53.5595551
H 0.8015677 -14.4086898 -53.9226587
H 0.1946828 -15.4764729 -52.6554527
C 2.1541222 -15.3188572 -50.9094302
H 2.6359165 -16.2380737 -51.2437664
H 1.18814 -15.559973 -50.4658729
H 2.7765934 -14.8574841 -50.1444956
N 2.9105554 -12.073782 -54.2549198
H 2.6628376 -11.510579 -53.4444772
C 2.8382215 -11.4848562 -55.603466
H 2.5477263 -12.2731829 -56.294507
C 4.1932573 -11.0523229 -56.1735234
O 4.3050569 -11.0014922 -57.3976465
C 1.7500749 -10.3972881 -55.6684607
H 1.973677 -9.6224873 -54.932472
H 1.7766916 -9.9369756 -56.657886
C 0.3181127 -10.9321047 -55.4242165
H 0.2277581 -11.265099 -54.390261
H -0.3748739 -10.1024204 -55.5677658
C -0.0987512 -12.0894509 -56.3560436
H 0.544746 -12.9544503 -56.17014
H 0.0153415 -11.7777132 -57.39766
C -1.5577073 -12.5187353 -56.1029819
H -2.2269318 -11.6802266 -56.3325773
H -1.6736438 -12.7561101 -55.0366384
N -1.920309 -13.7026939 -56.930816
H -1.8494992 -13.5031153 -57.9228767
H -2.903941 -13.9547134 -56.8042702
H -1.3493329 -14.5134468 -56.71273
N 5.239108 -10.8811623 -55.358782
H 5.0832237 -10.9063656 -54.3572045
C 6.6252368 -10.7490509 -55.8568326
H 6.6136699 -10.1725473 -56.7835268
C 7.23075 -12.111499 -56.2120809
O 7.8163342 -12.237301 -57.2830009
C 7.5248285 -9.9796415 -54.8686851
H 7.4639628 -10.4501565 -53.8877875
C 8.9905939 -9.944826 -55.3227672
H 9.0568977 -9.5997377 -56.3555774
H 9.4372889 -10.9357012 -55.2428839
H 9.5579733 -9.2635562 -54.6896495
C 7.0793244 -8.5186274 -54.7449023

H 7.2214826 -8.0117064 -55.6995769
H 7.6884329 -8.0243809 -53.9867521
H 6.0354079 -8.4688468 -54.4529373
N 7.0278598 -13.1595392 -55.4024952
H 6.5409429 -13.011564 -54.5230944
C 7.6271667 -14.4858677 -55.6559459
H 8.6634613 -14.3031979 -55.9438593
C 6.9906641 -15.2279986 -56.8419336
O 7.6716931 -15.9879257 -57.5225639
C 7.6575082 -15.3317962 -54.3638388
H 7.9124323 -14.6589608 -53.5431137
C 6.2996944 -15.9707711 -54.0400428
H 6.0816508 -16.7851927 -54.7321286
H 5.5262014 -15.2171777 -54.1221082
H 6.2972443 -16.3543042 -53.0201916
C 8.777694 -16.3911983 -54.4438692
H 8.5331507 -17.1303691 -55.2074872
H 9.7068405 -15.8989223 -54.7355244
C 9.0351373 -17.1189259 -53.1182629
H 8.173149 -17.7267722 -52.8436591
H 9.2387219 -16.3944539 -52.3289923
H 9.8999306 -17.7735446 -53.2299662
N 5.7387061 -14.9039989 -57.1868191
H 5.2127234 -14.3222172 -56.5485866
C 5.1062204 -15.3009733 -58.4534771
H 5.3874356 -16.3318692 -58.6680816
C 5.6030433 -14.4874707 -59.672003
O 5.0849158 -14.6599443 -60.773668
C 3.5735904 -15.2270815 -58.2905951
H 3.3168007 -14.2373792 -57.910768
H 3.1057086 -15.3358337 -59.269651
C 2.9775319 -16.3018904 -57.3585176
H 3.4899355 -16.2918303 -56.3990126
C 1.4957103 -15.9962792 -57.1210999
H 0.9610944 -15.9934025 -58.0710052
H 1.4152946 -15.0272253 -56.6348142
H 1.0717324 -16.755584 -56.4636815
C 3.0815974 -17.7101515 -57.947436
H 2.616133 -17.7444842 -58.9324024
H 2.5847047 -18.4241014 -57.2906702
H 4.1275589 -18.0041337 -58.0324987
N 6.5584693 -13.5695965 -59.4840437
H 6.9635998 -13.4805897 -58.559394
C 7.1419561 -12.7294521 -60.533019
H 8.0300305 -12.2428227 -60.1296168
H 7.4428545 -13.3604889 -61.3694312

C 6.2113295 -11.6372743 -61.0664694
O 6.4668133 -11.1007314 -62.1414297
N 5.0989407 -11.3521821 -60.3807085
H 4.9813281 -11.7997437 -59.4822739
C 4.011173 -10.5016823 -60.8819061
H 4.0372055 -10.488733 -61.9692609
C 4.1458654 -9.0360507 -60.4550618
O 4.0311999 -8.1487806 -61.2999311
C 2.6640571 -11.1114873 -60.4638078
H 1.8926477 -10.351034 -60.4842377
H 2.730934 -11.4831747 -59.4414438
C 2.2535011 -12.2657448 -61.3868724
H 1.4591474 -12.8339579 -60.902344
H 3.1069454 -12.932034 -61.5231857
S 1.6498686 -11.7327541 -63.0139356
C 1.6214996 -13.3280329 -63.8756168
H 1.2174771 -13.1904539 -64.8799789
H 0.9970689 -14.0358844 -63.3303468
H 2.6341481 -13.7267153 -63.9450518
N 4.4403578 -8.7747006 -59.1755103
H 4.5741277 -9.5745046 -58.565276
C 4.7721047 -7.4432695 -58.6461105
H 4.2329189 -6.6929209 -59.2287529
C 6.2628089 -7.1513591 -58.8255713
O 7.1132365 -8.0286716 -58.7190631
C 4.3124856 -7.3225627 -57.1776623
H 3.2218766 -7.3201566 -57.1538076
H 4.664482 -8.1940433 -56.6248092
C 4.8413101 -6.0586761 -56.4669405
H 5.9269408 -6.1201353 -56.3940316
H 4.5835999 -5.1738691 -57.0510368
C 4.2767258 -5.8952971 -55.0518431
H 4.2724669 -6.8599056 -54.5417714
H 3.2469937 -5.5465683 -55.1283597
C 5.0870934 -4.8828455 -54.2236432
H 4.4787623 -4.580427 -53.3658758
H 5.2958632 -3.991815 -54.8235343
N 6.3573003 -5.466491 -53.7188249
H 6.2395348 -6.3113772 -53.1868018
H 6.9070721 -4.8226841 -53.1439003
H 7.0461657 -5.6510471 -54.4521823
N 6.5831059 -5.8796803 -59.0166479
H 5.8235267 -5.2105063 -59.113986
C 7.9375657 -5.3227778 -59.0310328
H 8.6143732 -5.9856498 -58.4939189
C 7.9299697 -3.9753104 -58.3016906

O 6.8849011 -3.3299543 -58.2056804
C 8.3940536 -5.2114097 -60.498702
H 7.5989185 -4.7461053 -61.083704
C 9.6867695 -4.4393521 -60.7534294
H 10.461601 -4.7634189 -60.0624864
H 9.5092652 -3.3712309 -60.637248
H 10.0261984 -4.6212524 -61.7728531
O 8.6164189 -6.5095718 -60.9963761
H 8.1255677 -7.1366062 -60.4489059
N 9.0868513 -3.5097747 -57.8288001
H 9.9133854 -4.0836863 -57.9019445
C 9.2842826 -2.0970461 -57.484942
H 8.3344924 -1.5820776 -57.6283965
C 10.2397767 -1.4588863 -58.488381
O 11.1822076 -2.0878065 -58.9748673
C 9.6723067 -1.8988096 -55.9998239
H 9.4745394 -2.8358148 -55.4851521
C 11.1589555 -1.5434698 -55.8111287
H 11.3819425 -0.5661663 -56.2441685
H 11.7740098 -2.3086833 -56.2769605
H 11.4226248 -1.4985324 -54.7557414
C 8.7636256 -0.8305709 -55.3496209
H 8.9602225 0.1445593 -55.7991688
H 7.7226656 -1.0931424 -55.5430241
C 8.9349449 -0.7232145 -53.8274753
H 9.9006709 -0.2819557 -53.5826193
H 8.8562241 -1.7099889 -53.3708842
H 8.1532925 -0.0869728 -53.4164581
N 9.9517901 -0.2103244 -58.8094482
H 9.1682783 0.231535 -58.3373574
C 10.654951 0.6360141 -59.7608788
H 11.4393476 0.0802286 -60.2761536
C 11.3137825 1.7707113 -58.9834636
O 10.6744127 2.3280577 -58.0948429
C 9.5994166 1.0937026 -60.7929687
H 8.6089785 1.028997 -60.3467226
H 9.6158493 0.3827572 -61.6188321
C 9.6756245 2.4856417 -61.3678588
N 9.6560584 2.7683857 -62.7304314
C 9.6304442 4.1041051 -62.8320208
H 9.6677495 4.647484 -63.7669766
N 9.599696 4.6686446 -61.6145544
H 9.6754936 5.6576488 -61.4159251
C 9.6037052 3.6646771 -60.6808175
H 9.5692624 3.7963986 -59.6106405
N 12.5267095 2.1509732 -59.3842939

H 13.0058659 1.5797279 -60.0714761
C 13.1036285 3.4786336 -59.1419166
H 12.3791542 4.0801703 -58.6065819
C 13.4003518 4.137936 -60.4980748
O 13.9804095 3.4895145 -61.3751488
C 14.3486196 3.3947989 -58.2404563
H 14.0355429 3.0345998 -57.2602812
H 15.0443904 2.6671256 -58.661884
C 15.0998792 4.7290328 -58.0561606
H 15.5972644 4.9762276 -58.9978091
H 15.8861092 4.5829474 -57.3125827
C 14.2028056 5.9034645 -57.6227446
O 14.1451209 6.8963968 -58.3833069
O 13.5576779 5.8054743 -56.5593021
N 13.0453446 5.4126846 -60.6712948
H 12.6425479 5.9066621 -59.8759317
C 13.3632583 6.2062371 -61.8533931
H 14.1139036 5.6662568 -62.4167197
C 13.9496037 7.5757594 -61.5001384
O 13.3110616 8.3851175 -60.8299668
C 12.1357501 6.3519771 -62.752082
H 11.3121412 6.7960327 -62.1946084
H 11.8710934 5.3727228 -63.1411174
H 12.3807769 6.9994993 -63.5956822
N 15.109189 7.8745898 -62.0873356
H 15.4773862 7.2053572 -62.7563538
C 15.8432781 9.1316037 -61.9126432
H 15.2448618 9.8123746 -61.30726
C 16.0561624 9.7692783 -63.2824116
O 16.4188304 9.0839867 -64.2349318
C 17.1881392 8.8969502 -61.188147
H 17.8076121 8.2561194 -61.8169794
C 16.9764192 8.1765845 -59.8366834
H 16.3622953 8.7964619 -59.1838709
H 16.4432474 7.2414061 -60.0042166
C 17.9261826 10.2389898 -60.9959842
H 17.3004281 10.929227 -60.4298202
H 18.1645092 10.6855659 -61.9607414
H 18.8686102 10.0894265 -60.4738383
C 18.2749524 7.8099776 -59.110778
H 18.7708445 8.7035771 -58.7336403
H 18.9412565 7.2714474 -59.7855677
H 18.0370505 7.1684199 -58.2616609
N 15.8801458 11.0823937 -63.3805037
H 15.5331748 11.5872138 -62.5688742
C 15.9623305 11.817571 -64.6442913

H 16.7952455 11.4316431 -65.2351664
C 16.2306405 13.3158319 -64.4223685
O 16.001996 13.8407697 -63.3256233
C 14.6780471 11.5721577 -65.4405948
H 14.5614297 12.3592306 -66.1828294
H 14.8143697 10.6397859 -65.9771394
C 13.3981278 11.4233831 -64.5967226
H 13.2409175 12.3213791 -64.0054631
H 13.4843556 10.5777558 -63.912386
C 12.2010246 11.1445692 -65.486179
O 12.4220538 10.9836834 -66.7062945
O 11.0711677 11.0577901 -64.952999
N 16.7127389 14.0282412 -65.4533237
H 16.8577185 13.566903 -66.349382
C 16.9273486 15.4814323 -65.3898288
H 16.3871383 15.8476574 -64.5193038
C 16.3447654 16.2645858 -66.5817988
O 16.1558116 15.7419953 -67.6835179
C 18.4109936 15.8096423 -65.1287159
H 18.8261429 15.0930979 -64.4212154
H 18.4804463 16.7986531 -64.677849
C 19.2719235 15.817902 -66.3805842
O 19.9812442 14.8726451 -66.670495
N 19.2629312 16.8803734 -67.1552989
H 19.7115882 16.7659892 -68.0471681
H 18.6938739 17.6945154 -66.9492704
N 16.1171737 17.5560555 -66.3461034
H 16.3244087 17.9218972 -65.4216912
C 15.6409335 18.5213459 -67.3311437
H 15.1563779 17.9880668 -68.1515813
C 16.8207639 19.3019001 -67.9050657
O 17.814395 19.5196466 -67.2156087
C 14.6025547 19.470826 -66.7156745
H 15.0457816 19.9951369 -65.8663646
H 13.7532703 18.8943344 -66.3687152
C 14.1049765 20.4776596 -67.7549781
O 14.0497 21.6808959 -67.4357038
O 13.8939207 20.0301459 -68.9036003
N 16.6932574 19.7127438 -69.1587056
H 15.7361606 19.6535177 -69.5221682
C 17.6417525 20.4891035 -69.9664808
H 18.1709276 19.7718774 -70.5775385
C 18.7397635 21.2279062 -69.1911158
O 19.6163392 20.5303848 -68.6231238
O 18.7905354 22.4280729 -69.0989548
C 16.8603575 21.4153103 -70.9236658

H 16.3055296 22.1240006 -70.3041713
H 17.5800612 21.9809911 -71.516076
C 15.8643249 20.7213124 -71.8783228
H 15.1037594 20.1933291 -71.2956912
C 15.1458514 21.7861056 -72.7164382
H 15.8579213 22.3362843 -73.3312233
H 14.6219113 22.4788679 -72.0561909
H 14.4058411 21.3071409 -73.3609419
C 16.5435476 19.7199673 -72.8232848
H 17.329409 20.2094041 -73.398468
H 15.8002973 19.3095617 -73.5106352
H 16.9602655 18.8885803 -72.2557724

References

1. Pereira, P. S., França, S. de C., Oliveira, P. V. A. de, Breves, C. M. de S., Pereira, S. I. V., Sampaio, S. V., Nomizo, A., and Dias, D. A. (2008) Chemical constituents from Tabernaemontana catharinensis root bark: a brief NMR review of indole alkaloids and in vitro cytotoxicity. *Química Nova*. **31**, 20–24
2. Bennasar, M.-L., Zulaica, E., Solé, D., and Alonso, S. (2009) The first total synthesis of (±)-apparicine. *Chem Commun*. **0**, 3372–3374
3. Kutney, J. P., Horinaka, A., Ward, R. S., and Worth, B. R. (1980) Studies on the total synthesis of bisindole alkaloids within the voacamine family. *Can J Chem*. **58**, 1829–1838
